# Supplementary material for: Regioselective hydroamination of unactivated olefins with diazirines as a diversifiable nitrogen source
Source: Nat Commun. 2024 Jul 18;15:6049. doi: 10.1038/s41467-024-50254-8 (PMC11258257; doi:10.1038/s41467-024-50254-8)
Supplement: Supplementary file 1 — Supplementary Information [file 41467_2024_50254_MOESM1_ESM.pdf]

Supplementary Information for Regioselective Hydroamination of Unactivated Olefins with Diazirines as a Diversifiable Nitrogen Source

This file includes:
Supplementary Methods
Supplementary Discussion
Supplementary Note
Supplementary References

Table of Contents

Supplementary Methods ········ S3

General Experimental ········ S3

Handling of Reagents ········ S4

Synthesis of Diazirine 1 ········ S5

Synthesis of Catalysts ········ S8

Synthesis and Characterization Data of Alkenes ········ S10

Optimization Experiments ········ S35

General Procedure A: Co-catalyzed synthesis of substituted diaziridines ········ S39

General Procedure B: Co-catalyzed synthesis of substituted diaziridines (IPA) ········ S40

General Procedure C: Mn-catalyzed Synthesis of Substituted Diaziridines ········ S43

Supplementary Discussion ········ S46

Characterization Data of Substituted Diaziridines ········ S46

Proposed Mechanism ········ S100

Applications ········ S102

Synthesis of ^{15}*N*-labelled Diazirine (^{15}*N*-1) ········ S119

Synthesis of ^{15}*N*-25 ········ S126

NMR spectra for previously unreported compounds ········ S128

Supplementary Notes ········ S350

Troubleshooting ········ S350

Supplementary References ········ S351

Supplementary Methods:

General Experimental:

Reagents were purchased at the highest commercial quality and used without further purification, unless otherwise stated. Anhydrous methylene chloride (DCM) and ether (Et_2O) were obtained by passing the previously degassed solvent through an activated alumina column (PPT Glass Contour Solvent Purification System) unless otherwise stated. Tetrahydrofuran (THF) was dried over a sodium/benzophenone system and stored over 4 Å MS under argon. Dichloroethane (DCE) was dried over distillation from CaH_2 and stored over 4 Å MS under argon. Isopropanol (IPA) was purchased at the highest commercial quality and further dried over 4 Å MS and stored under argon. Methanol (MeOH) and Ethanol (EtOH) were purchased as highest quality and used without further purification. Yields refer to chromatographically and spectroscopically (^{1}H NMR) homogeneous material, unless otherwise stated. Reactions were monitored by Liquid Chromatography Mass spectrometry (LC/MS) or Thin Layer Chromatography (TLC) carried out on 250 µm SiliCycle SiliaPlates (TLC Glass-Backed TLC Extra Hard Layer, 60 Å), using visualizing agents such as shortwave UV light, iodine, $KMnO_4$, CAM, PMA, ninhydrin or *p*-anisaldehyde with heat as the developing agent. Flash column chromatography was performed with a Biotage Isolera One (ZIP or SNAP Ultra cartridges) or with traditional glass flash columns using SiliCycle SiliaFlash® P60 (particle size 40 - 63 µm). NMR spectra were recorded on a Bruker Ascend^{TM} 500 MHz instrument and were calibrated using residual undeuterated solvent as an internal reference (Chloroform-*d*: 7.26 ppm ^{1}H NMR, 77.16 ppm ^{13}C NMR; DMSO-*d6*: 2.50 ppm ^{1}H NMR, 39.5 ppm ^{13}CNMR). The following abbreviations were used to explain NMR peak multiplicities: s = singlet, d = doublet, t = triplet, q = quartet, dd = doublet of doublet, tt = triplet of triplet, ddt = doublet of doublet of triplet, m = multiplet. High resolution mass spectra (HRMS) were recorded on an Agilent 6230 LC–MS TOF mass spectrometer.

Handling of Reagents:

All diazirines were stored in a vial at -20 °C under argon (although the diazirines are moisture stable, this prevented water from being introduced into subsequent reactions). The vials containing diazirines were covered with aluminum foil to avoid prolonged exposure to direct light. (R,R)SalenCo^{III}OTs, tris(2,2,6,6-tetramethyl-3,5-heptanedionato)Mn^{III} [Mn(dpm)$_3$], and phenylsilane ($PhSiH_3$) were stored in a desiccator. *t*-Butyl hydroperoxide solution (*t*BuOOH) and di-*t*-butyl peroxide (*t*BuOO*t*Bu) were stored at 0-4 °C.

Synthesis of Diazirine 1:

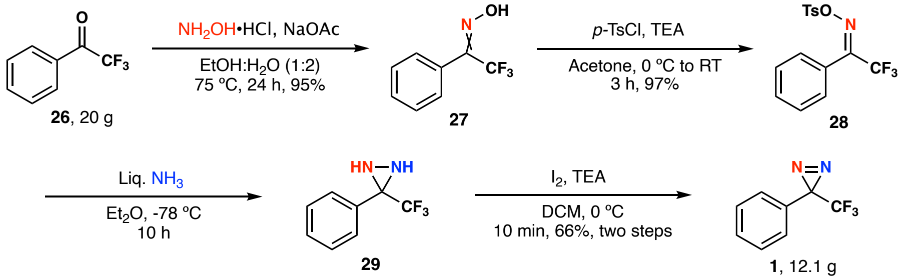

Supplementary Figure 1: 3-(trifluoromethyl)-3-phenyldiazirine (**1**) was synthesized as previously reported with minor modifications.[1]

2,2,2-trifluoro-1-phenylethan-1-one oxime

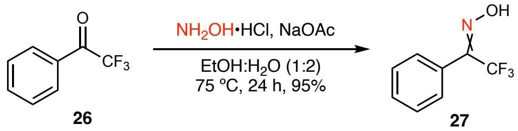

Followed previously reported protocol.[2] To a 100 mL round-bottom flask equipped with magnetic stir bar, 2,2,2-trifluoro-1-phenylethan-1-one (10.0 g, 57.4 mmol, 1 eq) was added followed by 30 mL water and 15 mL ethanol. Hydrochloride hydroxylamine (8.0 g, 114 mmol, 2 eq) and sodium acetate (10.8 g, 132 mmol, 2.3 eq) were added, and the mixture heated at 75 ºC for 24 h. After TLC indicating the disappearance of starting materials, ethanol was removed *in vacuo* and the solid was collected by filtration, washed with water (2 x 50 mL) and dried under reduced pressure offered **27** as white solid (10.3 g, 95%) and used without further purification.

2,2,2-trifluoro-1-phenylethan-1-one *O*-tosyl oxime

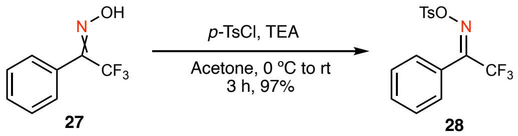

2,2,2-trifluoro-1-phenylethan-1-one *O*-tosyl oxime (**28**) was made following our published procedure with minor modifications.[20] To a 500 mL round-bottom flask equipped with magnetic stir bar, **27** (10.0 g, 52.9 mmol, 1 eq.) was added, followed by 250 mL acetone then cooled to 0 °C with an ice bath. 4-Toluenesulfonyl chloride (12.1 g, 63.5 mmol, 1.2 eq) was added, followed by addition of triethyl amine (22.3 ml, 16.1 g, 159 mmol, 3 eq) dropwise then stirred at room temperature for 3 h until TLC monitoring indicated total consumption of starting material **27**. The reaction mixture slowly transitioned from white to an orange color and solid formation was observed. The suspension was filtered, and the cake washed with acetone (2 x 50 mL). Filtrate was concentrated *in vacuo*, giving a light orange solid, to which ethanol (75 mL) and water (25 mL) were added, then refluxed for 1 h and stirred at room temperature overnight. Another portion of water (100 mL) added and solid was collected by filtration, washed with water (2 x 25 mL), then

dried under reduced pressure to afford **28** as a white solid (17.6 g, 97%) in mostly (>95%) the E form.
The ^{1}H NMR spectrum matched with previously reported spectra.[1]

Physical state: White solid. $\mathbf{R_f}$ = 0.5 (20 % EA in hexanes, vis. UV and iodine).

^{1}H NMR (500 MHz, $CDCl_3$) δ 7.89 (d, J = 8.4 Hz, 2H), 7.57 – 7.51 (m, 1H), 7.47 (ddd, J = 8.5, 7.2, 1.1 Hz, 2H), 7.41 – 7.35 (m, 4H), 2.48 (s, 3H).

3-phenyl-3-(trifluoromethyl)diaziridine

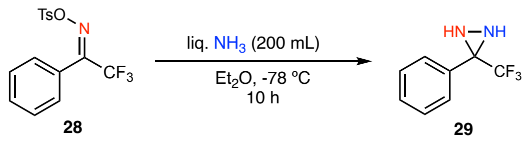

To a flame-dried 1000 mL round-bottom flask equipped with a magnetic stir bar at -78 ºC, liq. NH_3 (approx. 200 mL) was added under argon atmosphere, followed by addition of anhydrous ether (140 mL). **28** (33.5 g, 97.6 mmol, 1 eq.) was added then stirred for 10 hours at -78 ºC. The septum was removed, the reaction slowly warmed to room temperature overnight, and ammonia was evaporated slowly. To the residue was added water (100 mL) and extracted with Et_2O (3 x 100 mL), then washed with brine (100 mL), dried over anhydrous sodium sulfate (Na_2SO_4), filtered and concentrated *in vacuo*, yielding diaziridine **29** (19.0 g, >99% yield). **29** was used in the next step without further purification.

3-phenyl-3-(trifluoromethyl)-3*H*-diazirine

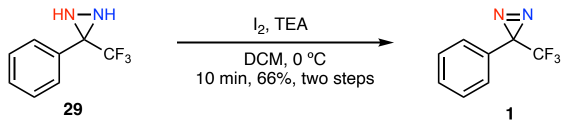

To a flame-dried 1000 mL round-bottom flask equipped with a magnetic stir bar under argon was added diaziridine **29** (18.4 g, 97.8 mmol, 1 eq.), followed by anhydrous dichloromethane (500 mL) then cooled to 0 °C. The round-bottom flask was covered with aluminum foil, then triethyl amine (21.6 g, 30.0 mL, 213 mmol, 2.2 eq.) was added dropwise and stirred for 10 min before iodine (13.4 g, 52.7 mmol, 1.1 eq.) was added portion-wise. With each addition, a dark yellow/brown color developed and then vanished shortly after. Upon completion of the reaction, the brown color persisted for more than 10 minutes at room temperature. The reaction mixture was poured into separatory funnel then was washed with saturated sodium thiosulphate (100 mL) and water (100 mL). The aqueous phases were combined, then extracted with dichloromethane (3 x 50 mL). Then organic phases were combined, dried over anhydrous magnesium sulfate, concentrated *in vacuo* then purified with column chromatography (silica gel, 100% pentane) to afford **1** (12.1 g, 66% yield over two steps) as a colorless oil.

Note: a) extraction must be quickly and protected from light. b) **1** is volatile (high vacuum drying not recommended).

Physical state: colorless oil. $\mathbf{R_f}$ = 0.8 (10 % EA in hexanes, vis. UV).

^{1}H NMR: (500 MHz, $CDCl_3$) δ 7.45 - 7.37 (m, 3H), 7.23 - 7.18 (m, 2H).

^{13}C NMR: (126 MHz, $CDCl_3$) δ 129.8, 129.3, 128.9, 126.6, 122.3 (q, $J_{C–F}$ = 274.7 Hz), 28.6 (q, $J_{C–F}$ = 40.4 Hz).

^{19}F NMR: (471 MHz, $CDCl_3$) δ -65.25.

Synthesis of (*R,R*)-SalenCo^{III}OTs:

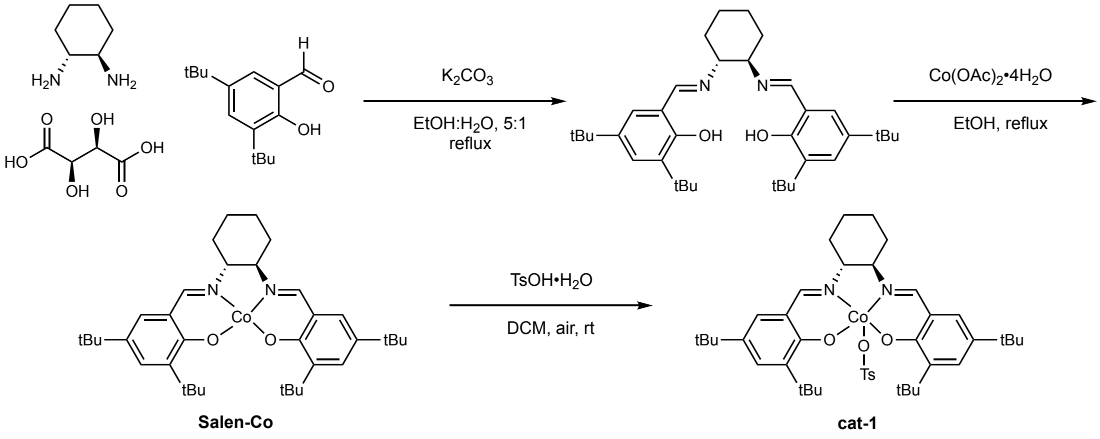

Supplementary Figure 2: Synthesis of **cat-1**.

6,6'-((1*E*,1'*E*)-(((1*R*,2*R*)-cyclohexane-1,2-diyl)bis(azaneylylidene))bis(methaneylylidene)) bis(2,4-di-*tert*-butylphenol)

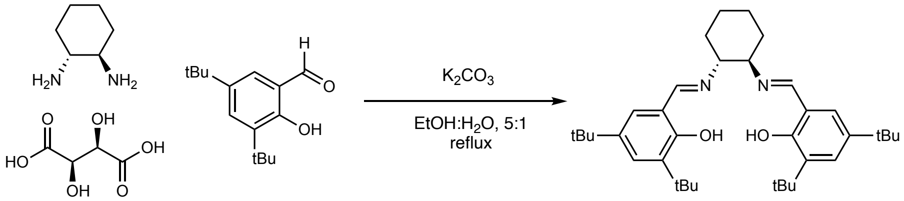

Step 1: Followed previously reported protocol. [3] To a flame-dried 1000 mL round-bottom flask equipped with a magnetic stir bar was added (1*R*,2*R*)-(+)-1,2-diaminocyclohexane L-tartrate (2.00 g, 7.57 mmol, 1 eq) followed by ethanol (50 mL) and water (10 mL). 3,5-Di-*tert*-butyl-2-hydroxybenzaldehyde (3.51 g, 15.0 mmol, 1.98 eq) and potassium carbonate (2.09 g, 15.1 mmol, 2 eq) were added. The reaction mixture was stirred at reflux for 2 h until TLC monitoring indicated the disappearance of 3,5-di-*tert*-butyl-2-hydroxy benzaldehyde. The reaction mixture was cooled to room temperature with stirring and filtered with a Büchner funnel. The yellow solid was washed with a small amount of chilled ethanol and dried on vacuum, yielding (*R,R*)-salenH_2 (3.99 g , 97% yield).

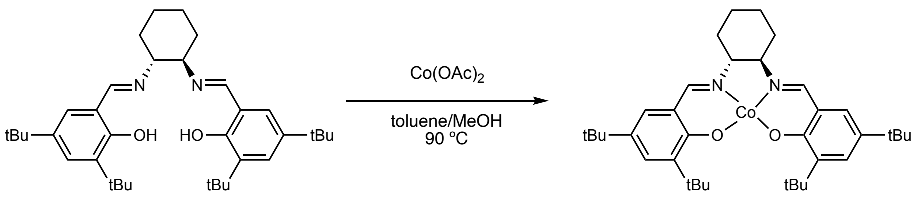

Step 2: Followed previously reported protocol. [3] To a flame-dried 100 mL round-bottom flask equipped with a magnetic stir bar under argon was added (*R,R*)-salenH_2 (1.30 g, 2.40 mmol, 1 eq) followed by dry toluene (10 mL). A solution of $Co(OAc)_2$ (0.42 g, 2.40 mmol, 1 eq) in dry methanol (20 mL) was then added via syringe to afford a red precipitate. The reaction mixture

was stirred at 90 ºC for 1 h. The reaction mixture was cooled, and the red precipitate was collected by filtration and washed with methanol until the filtrate turned colorless. The final product was dried at 40 ºC under vacuum to a constant weight, yielding the product as a red powder (1.20 g, 83% yield).

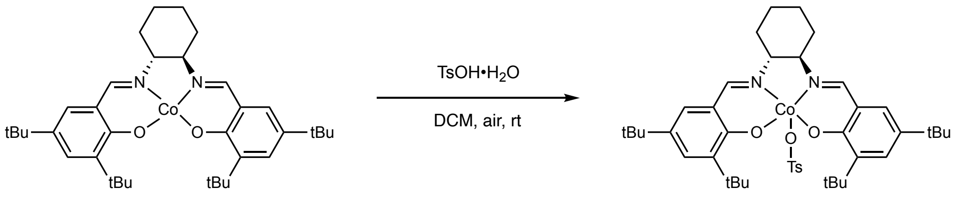

Step 3: Followed previously reported protocol.[3] To a 500 mL round-bottom flask equipped with a magnetic stir bar under argon was added (*R,R*)-salenCo^{II} complex (6.00 g, 9.95 mmol, 1 eq) followed by *p*-toluenesulfonic acid monohydrate (1.90 g, 9.95 mmol, 1 eq) and dichloromethane (200 mL). The reaction mixture was stirred under air atmosphere at room temperature for 3 h. The volatiles were removed *in vacuo* and the remaining solid was suspended in hexane (50 mL), re-collected by filtration, washed with a dichloromethane/hexanes (1:3) mixture (40 mL), and dried at 40 ºC under vacuum to a constant weight (6.61 g, 86 % yield).

The ^{1}H NMR spectrum matched with previously reported spectra.[3]

Physical state: dark green solid.

^{1}H NMR (500 MHz, DMSO) δ 7.81 (s, 2H), 7.51 – 7.42 (m, 6H), 7.11 (d, *J* = 8.0 Hz, 2H), 3.62 (dd, *J* = 7.1, 2.8 Hz, 2H), 3.07 (d, *J* = 11.8 Hz, 2H), 2.29 (s, 3H), 2.06 – 1.97 (m, 2H), 1.97 – 1.86 (m, 2H), 1.75 (s, 18H), 1.63 – 1.54 (m, 2H), 1.31 (s, 18H).

The following catalysts were synthesized by slight modifications to **step 3** of the above procedure: (*R,R*)-SalenCo^{III} (OAc) was synthesized via reaction of **Salen-Co** with acetic acid (AcOH), (*R,R*)-(*R,R*)-SalenCo^{III} (Cl) via reaction of **Salen-Co** with sodium chloride. ^{1}H NMR matched previously reported spectra.

Characterization Data for Alkenes

1-(But-3-en-1-yloxy)-4-methoxybenzene

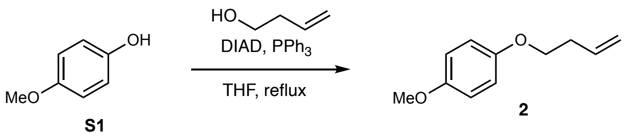

Followed previously reported protocol.[4] To a 50 mL round-bottom flask equipped with a magnetic stir bar under argon was added 3-buten-1-ol (642 mg, 8.91 mmol, 1.05 eq) and anhydrous tetrahydrofuran (25 mL). 4-Methoxyphenol (1.06 g, 8.50 mmol, 1 eq) and triphenylphosphine (2.90 g, 11.1 mmol, 1.3 eq) were added then the reaction mixture was stirred for 5 min at 0 ºC, followed by addition of diisopropyl azodicarboxylate (DIAD) (2.41 g, 2.34 mL, 11.9 mmol, 1.4 eq) via syringe slowly. The mixture was then refluxed for 15 min until TLC monitoring indicated total consumption of the starting material. The crude mixture was concentrated *in vacuo* and purified via flash column chromatography (silica gel, 10% EA in hexanes) yielding **2** (1.42 g, 93% yield).

The ^{1}H NMR spectrum matched with previously reported spectra.[4]

Physical state: colorless oil. $\mathbf{R_f}$ = 0.5 (10% EA in hexanes, vis. UV and $KMnO_4$).

^{1}H NMR (500 MHz, $CDCl_3$) δ 6.87 – 6.81 (m, 4H), 5.91 (ddt, *J* = 17.1, 10.4, 6.7 Hz, 1H), 5.17 (dq, *J* = 17.2, 1.7 Hz, 1H), 5.13 – 5.08 (m, 1H), 3.97 (t, *J* = 6.7 Hz, 2H), 3.77 (s, 3H), 2.52 (qt, *J* = 6.7, 1.4 Hz, 2H).

But-3-en-1-yl(phenyl)sulfane

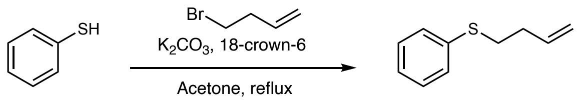

Followed previously reported protocol.[5] To a 50 mL round-bottom flask equipped with a magnetic stir bar under argon was added thiophenol (648 mg, 5.88 mmol, 1 eq), acetone (40 ml), potassium carbonate (1.21 g, 8.82 mmol, 1.5 eq) and 18-crown-6 (155 mg, 0.588 mmol, 0.1 eq). The reaction mixture was stirred for 5 min followed by addition of 4-bromobut-1-ene (795 mg, 5.88 mmol, 1.0 eq). The reaction mixture was stirred under reflux overnight until TLC monitoring indicated total consumption of the starting material (SM and TM have a very close R_f, but stain differently with $KMnO_4$). The reaction mixture was diluted with water (200 mL) and extracted with ethyl acetate (50 mL x 3). The organic layers were combined and washed with 10% sodium hydroxide (50 mL), brine (50 mL), dried over *anhyd.* Na_2SO_4 and concentrated *in vacuo*. The residue was purified via flash column chromatography (silica gel, 100% hexanes) yielding but-3-en-1-yl(phenyl)sulfane (716 mg, 74% yield).

The ^{1}H NMR spectrum matched with previously reported spectra.[5]

Physical state: colorless oil. $\mathbf{R_f}$ = 0.3 (100% hexanes, vis. UV and $KMnO_4$).

^{1}H NMR: (500 MHz, $CDCl_3$) δ 7.38 – 7.32 (m, 2H), 7.31 – 7.26 (m, 2H), 7.21 – 7.15 (m, 1H), 5.86 (ddt, *J* = 16.9, 10.2, 6.6 Hz, 1H), 5.15 – 4.95 (m, 2H), 2.99 (dd, *J* = 7.9, 7.0 Hz, 2H), 2.40 (dtt, *J* = 9.4, 6.7, 1.4 Hz, 2H).

***tert*-Butyldimethyl((3-methylbut-2-en-1-yl)oxy)silane**

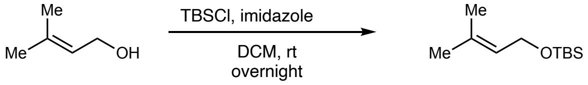

Followed previously reported protocol.[5] To a 25 mL round-bottom flask equipped with a magnetic stir bar was added 3-methylbut-3-en-1-ol (1.00 g, 11.6 mmol, 1 eq) followed by anhydrous dichloromethane (10 mL) and imidazole (1.58 g, 23.2 mmol, 2.0 eq). *tert*-Butyldimethylsilyl chloride (2.62 g, 17.4 mmol, 1.5 eq) was added in one portion then stirred overnight under room temperature until TLC monitoring indicated total consumption of starting material. The reaction mixture was concentrated *in vacuo*, and the residue purified via flash column chromatography (silica gel, 100% hexanes) yielding *tert*-butyldimethyl((3-methylbut-2-en-1-yl)oxy)silane (1.11 g, 48% yield).

The ^{1}H NMR spectrum matched with previously reported spectra.[5]

Physical state: colorless oil. $\mathbf{R_f}$ **=** 0.3, (100% hexanes, vis. PMA and $KMnO_4$).

^{1}H NMR: (500 MHz, $CDCl_3$) δ 5.38 – 5.20 (m, 1H), 4.17 (dt, *J* = 6.5, 1.1 Hz, 2H), 1.71 (q, *J* = 1.4 Hz, 3H), 1.63 (d, *J* = 1.3 Hz, 3H), 0.90 (s, 9H), 0.07 (s, 6H).

3-Methylbut-3-en-1-yl acetate

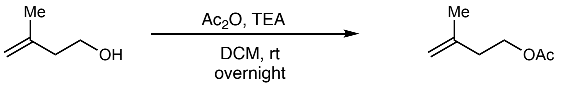

To a 25 mL round-bottom flask equipped with a magnetic stir bar was added 3-methylbut-3-en-1-ol (860 mg, 10.0 mmol, 1 eq) followed by anhydrous dichloromethane (10 mL). Acetic anhydride (1.13 g, 1.05 ml, 11.1 mmol, 1.1 eq) was added in one portion and the reaction mixture was stirred for 10 min at room temperature, then triethylamine (1.19 g, 1.66 ml, 11.8 mmol, 1.2 eq) was added dropwise. The mixture was stirred at room temperature overnight until TLC monitoring indicated total consumption of starting material. The reaction mixture was concentrated *in vacuo*, and the residue purified via flash column chromatography (silica gel, 2% ethyl actate in hexanes) yielding 3-methylbut-3-en-1-yl acetate (430 mg, 34% yield).

The ^{1}H NMR spectrum matched with previously reported spectra. [6]

Physical state: colorless oil. $\mathbf{R_f}$ = 0.8 (10% EA in hexanes vis. PMA and $KMnO_4$).

^{1}H NMR: (500 MHz, $CDCl_3$) δ 4.89 – 4.63 (m, 2H), 4.18 (t, J = 6.9 Hz, 2H), 2.45 – 2.18 (m, 2H), 2.04 (s, 3H), 1.87 – 1.68 (m, 3H).

2-((3-Methylbut-3-en-1-yl)oxy)tetrahydro-2*H*-pyran

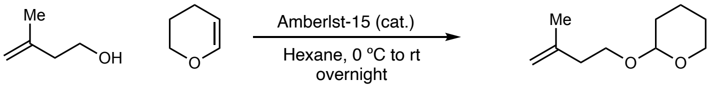

Followed previously reported protocol.[7] To a 25 mL round-bottom flask equipped with a magnetic stir bar was added 3-methylbut-3-en-1-ol (860 mg, 10.0 mmol, 1 eq) and hexane (10 mL), followed by 3,4-dihydro-2*H*-pyran (1.29 g, 1.40 mL, 15.4 mmol, 1.5 eq). The reaction mixture was stirred at 0 ºC then several pellets of Amberlyst-15 was added. The reaction mixture was warmed to room temperature and stirred overnight until TLC monitoring indicated total consumption of the starting material. Amberlyst-15 was removed by filtration and the filtrate was washed with *sat.* $NaHCO_3$ (10 mL). The aqueous layer was extracted with dichloromethane (25 mL x 3), the organic layers combined, then washed with brine, dried over *anhydrous* Na_2SO_4 then concentrated *in vacuo*. The residue was purified via flash column chromatography (silica gel, 14% acetone in hexanes) yielding 2-((3-methylbut-3-en-1-yl)oxy)tetrahydro-2H-pyran (1.30 g, 76% yield).

The ^{1}H NMR spectrum matched with previously reported spectra.[7]

Physical state: colorless oil. $\mathbf{R_f}$ = 0.7 (10% ethyl actate in hexanes, vis. PMA and $KMnO_4$).

^{1}H NMR: (500 MHz, $CDCl_3$) δ 4.80 – 4.69 (m, 2H), 4.59 (dd, *J* = 4.4, 2.9 Hz, 1H), 3.85 (ddd, *J* = 14.3, 10.5, 7.8 Hz, 2H), 3.56 – 3.44 (m, 2H), 2.32 (t, *J* = 7.1 Hz, 2H), 1.83 (td, *J* = 8.7, 3.6 Hz, 1H), 1.77 – 1.74 (m, 3H), 1.68 (t, *J* = 4.4 Hz, 1H), 1.61 – 1.49 (m, 4H).

4-(Methoxymethoxy)-2-methylbut-1-ene

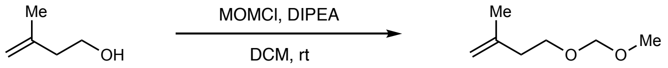

To a 25 mL round-bottom flask equipped with a magnetic stir bar was added 3-methylbut-3-en-1-ol (200 mg, 2.32 mmol, 1 eq) and anhydrous dichloromethane (5 mL), followed by chloro(methoxymethoxy)methane (747 mg, 0.71 mL, 9.28 mmol, 4.0 eq). The reaction mixture was stirred for 10 min under room temperature then *N*-ethyl-*N*-isopropylpropan-2-amine (1.40 g, 1.94 mL, 13.8 mmol, 6.0 eq) was added. The reaction mixture was stirred at room temperature overnight until TLC monitoring indicated total consumption of the starting materials, then the reaction mixture was concentrated *in vacuo*, and the residue purified via flash column chromatography (silica gel, 2% ethyl acetate in hexanes) yielding 4-(methoxymethoxy)-2-methylbut-1-ene (34.2 mg, 11% yield).

Physical state: colorless oil. $\mathbf{R_f}$ = 0.7 (10% ethyl acetate in hexanes, vis. PMA and $KMnO_4$).

^{1}H NMR: (500 MHz, CDCl3) δ 4.87 – 4.70 (m, 2H), 4.63 (s, 2H), 3.65 (t, J = 6.8 Hz, 2H), 3.36 (s, 3H), 2.37 – 2.23 (m, 2H), 1.76 (t, J = 1.2 Hz, 3H).

^{13}C NMR: (126 MHz, $CDCl_3$) δ 142.9, 111.7, 96.6, 66.2, 55.4, 37.9, 22.7.

HRMS: Calculated for $C_7H_{15}O_2^+$ 131.1067 [M+H$^+$]; found 131.1071.

(((3-Methylbut-3-en-1-yl)oxy)methyl)benzene

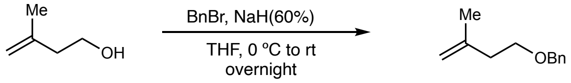

To a 25 mL round-bottom flask equipped with a magnetic stir bar was added 3-methylbut-3-en-1-ol (500 mg, 5.80 mmol, 1 eq) and anhydrous tetrahydrofuran (5 mL). The resulting mixture was cooled to 0 ºC. Sodium hydride (60% in mineral oil, 280 mg, 7.00 mmol, 1.2 eq) was added portion-wise with stirring. After addition, argon was replaced and benzyl bromide (1.09 g, 0.76 mL, 6.39 mmol, 1.1 eq) was added via syringe. The reaction mixture was warmed to room temperature and stirred overnight, until TLC indicated total consumption of starting material. Water (10 mL) was added slowly to quench the reaction, and the mixture was poured into a separatory funnel, followed by addition of water (50 mL) and extracted with ethyl acetate (30 mL x 3). The organic phases were combined, washed with brine (50 mL), dried over *anhyd.* Na_2SO_4, then concentrated *in vacuo*. The residue was purified via flash column chromatography (silica gel, 2% ethyl acetate in hexanes) yielding (((3-methylbut-3-en-1-yl)oxy)methyl)benzene (1.02 g, 99% yield).

The ^{1}H NMR spectrum matched with previously reported spectra. [8]

Physical state: colorless oil. $\mathbf{R_f}$ = 0.9 (10% ethyl acetate in hexanes, vis. PMA and $KMnO_4$).

^{1}H NMR: (500 MHz, $CDCl_3$) δ 7.37 – 7.27 (m, 5H), 4.85 – 4.70 (m, 2H), 4.53 (s, 2H), 3.59 (t, *J* = 6.9 Hz, 2H), 2.35 (tt, *J* = 6.9, 0.9 Hz, 2H), 1.75 (t, *J* = 1.2 Hz, 3H).

Benzyl (3-methylbut-3-en-1-yl) carbonate

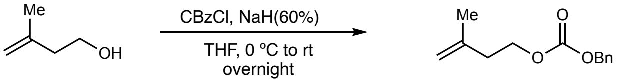

To a 25 mL round-bottom flask equipped with a magnetic stir bar was added 3-methylbut-3-en-1-ol (500 mg, 5.80 mmol, 1 eq) and anhydrous tetrahydrofuran (5 mL). The resulting mixture was cooled to 0 ºC. Sodium hydride (60% in mineral oil, 280 mg, 7.00 mmol, 1.2 eq) was added portion-wise with stirring. After addition, argon was replaced and benzyl chloroformate (1.20 g, 1.00 mL, 7.03 mmol, 1.2 eq) was added via syringe. The reaction mixture was warmed to room temperature and stirred overnight, until TLC indicated total consumption of starting material. Water (10 mL) was added slowly to quench the reaction, and the mixture was poured into a separatory funnel, followed by addition of water (50 mL) and extracted with ethyl acetate (30 mL x 3). The organic phases were combined, washed with brine (50 mL), dried over *anhyd.* Na_2SO_4, then concentrated *in vacuo*. The residue was purified via flash column chromatography (silica gel, 2% ethyl acetate in hexanes) yielding benzyl (3-methylbut-3-en-1-yl) carbonate (854 mg, 67% yield).

The ^{1}H NMR spectrum matched with previously reported spectra. [9]

Physical state: colorless oil. $\mathbf{R_f}$ = 0.7 (10% ethyl acetate in hexanes, vis. PMA and $KMnO_4$).

^{1}H NMR: (500 MHz, $CDCl_3$) δ 7.47 – 7.31 (m, 5H), 5.18 (s, 2H), 4.88 – 4.74 (m, 2H), 4.29 (t, *J* = 7.0 Hz, 2H), 2.41 (td, *J* = 6.9, 1.3 Hz, 2H), 1.78 (t, *J* = 1.2 Hz, 3H).

(1S,4R)-1-isopropyl-4-methyl-2-methylenecyclohexane

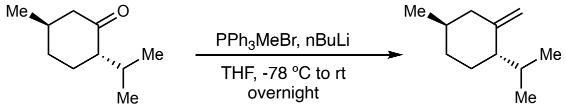

To a 250 mL round-bottom flask equipped with a magnetic stir bar was added triphenylphosphine methyl bromide (6.00 g, 16.8 mmol, 1.3 eq) then the placed under argon. Anhydrous tetrahydrofuran (45 mL) added, followed by slow addition of *n*BuLi (2.5 M, 6.8 mL, 16.8 mmol, 1.3 eq) under -78 ºC with stirring. The reaction mixture was stirred under -78 ºC for 1 h and a yellow solution was formed. Menthone (2.01 g, 13.0 mmol, 1 eq) was added in one portion via a syringe and the reaction mixture warmed to room temperature slowly and stirred overnight. Water (25 mL) was added slowly to quench the reaction, and the mixture was poured into a separatory funnel, followed by addition of water (200 mL) and extracted with ether (100 mL x 3), washed with brine (50 mL), dried over *anhyd.* Na_2SO_4 and concentrated *in vacuo*. The residue purified via flash column chromatography (silica gel, 100% hexanes) yielding **(**1S,4R)-1-isopropyl-4-methyl-2-methylenecyclohexane (883 mg, 45% yield).

The ^{1}H NMR spectrum matched with previously reported spectra. [10]

Physical state: colorless oil. **R_f** = 0.9, (10% ethyl acetate in hexanes, vis. PMA and $KMnO_4$).

^{1}H NMR: (500 MHz, $CDCl_3$) δ 4.70 (dd, *J* = 2.2, 1.1 Hz, 1H), 4.58 (t, *J* = 1.4 Hz, 1H), 2.28 (dddd, *J* = 12.7, 4.1, 1.8, 1.0 Hz, 1H), 1.96 (dq, *J* = 13.3, 6.6 Hz, 1H), 1.84 – 1.72 (m, 2H), 1.70 – 1.63 (m, 2H), 1.62 – 1.51 (m, 1H), 1.21 – 1.03 (m, 2H), 0.93 – 0.87 (m, 9H).

tert-butyl 2,3,6,7-tetrahydro-1_H_-azepine-1-carboxylate (20)

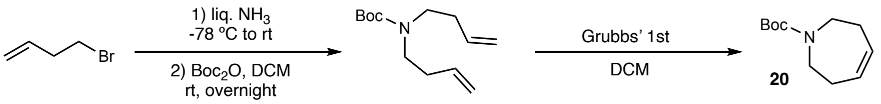

To a flame-dried 25 mL sealed tube equipped with a magnetic stir bar, a rubber stopper was fitted and liq. ammonia (~6 mL) added under -78 ºC, followed by addition of 1-bromobut-3-ene (5.00 g, 37.04 mmol, 1 eq). The reaction mixture was sealed and stirred at 40 ºC overnight then the tube was cooled to -78 ºC, carefully opened and warmed to room temperature until the ammonia evaporated. Water (20 mL) was added, the pH was adjusted to 1 with 3 M HCl and extracted with ether (50 mL x 3). The organic phase was discarded. To the aqueous layer was added a 5% sodium hydroxide solution until pH 14, then extracted with ether (50 mL x 3). The organic layers were combined, dried over *anhyd.* Na_2SO_4, filtered, and concentrated *in vacuo*. The crude product was used in the next step without further purification.

To a 50 mL round-bottom flask equipped with a magnetic stir bar was added the residue (0.96 g) from the previous step and dichloromethane (10 mL). Boc_2O (2.85 g, 3.00 mL, 13.05 mmol, 0.5 eq based on 1-bromobut-3-ene) was added slowly, and the reaction mixture stirred at room temperature overnight, until TLC monitoring confirmed the disappearance of the starting material. The reaction mixture was concentrated *in vacuo* and the residue purified via flash column chromatography (silica gel, 5% ethyl acetate in hexanes) yielding *tert*-butyl di(but-3-en-1-yl)carbamate (1.26 g , 31% yield).

The ^{1}H NMR spectrum matched with previously reported spectra. [11]

Physical state: colorless oil. $\mathbf{R_f}$ = 0.5 (10% ethyl acetate in hexanes, $KMnO_4$).

^{1}H NMR: (500 MHz, $CDCl_3$) δ 5.91 – 5.57 (m, 2H), 5.15 – 4.91 (m, 4H), 3.28 – 3.18 (m, 4H), 2.27 (q, J = 7.2 Hz, 4H), 1.46 (s, 9H).

To a 500 mL round-bottom flask equipped with a magnetic stir bar was added *tert*-butyl di(but-3-en-1-yl)carbamate (380 mg, 1.68 mmol, 1 eq) and dichloromethane (200 mL). Grubbs' 1st generation catalyst (93 mg, 0.11 mmol, 6.5 %) was added, and the reaction placed under argon. The reaction mixture was stirred under reflux for 2 h until TLC monitoring indicated the disappearance of the starting material. The reaction mixture was concentrated on silica gel and purified via flash column chromatography (silica gel, 4% ethyl acetate in hexanes) affording *tert*-butyl 2,3,6,7-tetrahydro-1*H*-azepine-1-carboxylate (**21**) (240 mg, 72% yield).

The ^{1}H NMR spectrum matched with previously reported spectra. [11]

Physical state: colorless oil. $\mathbf{R_f}$ = 0.4 (10% ethyl acetate in hexane, vis. $KMnO_4$).

^{1}H NMR: (500 MHz, $CDCl_3$) δ 5.72 (d, J = 2.6 Hz, 2H), 3.55 – 3.36 (m, 4H), 2.37 – 2.16 (m, 4H), 1.46 (s, 9H).

4-(prop-1-en-2-yl)-1-tosylpiperidine

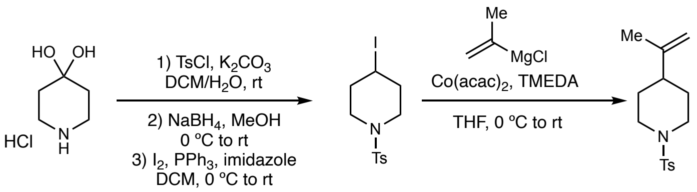

Followed previously reported procedure. [12] To a 250 mL round-bottom flask equipped with a magnetic stir bar was added 4-piperidone monohydrate hydrochloride (5.00 g, 32.5 mmol, 1 eq), dichloromethane (30 mL), water (30 mL) and potassium carbonate (10.8 g, 78.0 mmol, 2.4 eq). The reaction mixture was stirred until all the solid disappeared, followed by addition of 4-toluenesulfonyl chloride (6.50 g, 34.1 mmol, 1.05 eq) portion-wise, then the reaction mixture was stirred overnight at room temperature, until LC-MS indicated disappearance of the starting materials. The reaction mixture poured into a separatory funnel and extracted with dichloromethane (50 mL x 3), washed with brine (50 mL) and concentrated *in vacuo*. A white solid (9.49 g, over 100%) was collected and used without further purification.

The tosylated compound from the previous step was dissolved in methanol (20 mL) in a 50 mL round bottom flask. The mixture was cooled to 0 ºC followed by addition of $NaBH_4$ (1.40 g, 35.0 mmol, 1.1 eq based on 4-piperidone monohydrate hydrochloride) portion-wise. The reaction mixture warmed to room temperature and stirred overnight until LC-MS indicated the disappearance of the starting materials. Most of the methanol (not to dryness) was removed *in vacuo*, then water (100 mL) was added. The resulting suspension was stirred for 1 h and the solid collected by filtration. The yellow solid (7.48 g) was collected and used without further purification.

Followed previously reported protocol. [13] To a 100 mL round bottom flask equipped with a magnetic stir bar was added imidazole (800 mg, 11.8 mmol, 1.5 eq), PPh_3 (3.08 g, 11.8 mmol, 1.5 eq) and dichloromethane (20 mL). Iodine (2.98 g, 11.8 mmol, 1.5 eq) was added portion-wise, then the reaction mixture was stirred for 30 min at room temperature. The reaction mixture was cooled to 0ºC, followed by slow addition of the alcohol from the last step (2.00 g, 7.83 mmol, as a solution in 8 mL dichloromethane), then warmed to room temperature and stirred overnight. After LC-MS indicated disappearance of starting materials, the reaction mixture was concentrated *in vacuo* and purified via flash column chromatography (silica gel, 5% to 10% ethyl acetate in hexane), to afford a white solid (1.68 g, 59% over three steps). The product slowly turned yellow under light.
(Note: changing the order of addition will give no desired product.)

The coupling reaction followed Cossy's reported procedure. [14] To a flame-dried 25 mL round bottom flask equipped with a magnetic stir bar was added 4-iodo-1-tosylpiperidine (1.00 g, 2.74 mmol, 1 eq) and anhydrous tetrahydrofuran (5 mL). $Co(acac)_2$ (24.7 mg, 0.095 mmol, 3.5 mol%) and dried TMEDA (318 mg, 410 μL, 2.74 mmol, 1 eq) were added sequentially, then the reaction placed under argon. The reaction mixture was cooled to 0 ºC, followed by the addition of prop-1-en-2-ylmagnesium chloride (6.85 mL, 0.5 M in THF, 3.42 mmol, 1.25 eq) with a syringe pump at a rate of 1 mL/h with stiring at 0 ºC. After the addition was complete, the reaction mixture was

warmed to room temperature and stirred at room temperature for 7 h until TLC indicated the disappearance of starting materials. The reaction mixture was concentrated *in vacuo* and the residue purified via flash column chromatography (silica gel, 5% ethyl acetate in hexane), yielding the product as a white solid (488 mg, 64%).

The ^{1}H NMR spectrum matched with previously reported spectra. [15]

Physical state: white solid. $\mathbf{R_f}$ = 0.3 (10% ethyl acetate in hexane, vis. UV).

^{1}H NMR: (500 MHz, $CDCl_3$) δ 7.67 (d, *J* = 8.4 Hz, 2H), 7.35 (d, *J* = 7.8 Hz, 1H), 4.83 – 4.61 (m, 2H), 3.87 (dt, *J* = 11.6, 2.5 Hz, 2H), 2.46 (s, 3H), 2.25 (td, *J* = 12.0, 2.5 Hz, 2H), 1.84 – 1.73 (m, 3H), 1.70 (t, *J* = 1.1 Hz, 3H), 1.64 – 1.53 (m, 2H).

tert-butyl (6-methoxyquinolin-8-yl)(pent-4-en-1-yl)carbamate

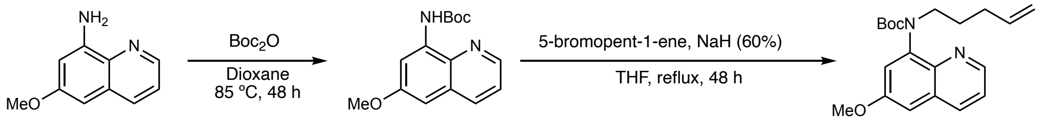

To a flame-dried 25 mL round-bottom flask equipped with a magnetic stir bar was added 8-amino-6-methoxyquinoline (305 mg, 1.75 mmol, 1 eq) and dioxane (6 mL). Boc_2O (764 mg, 804 µL, 3.50 mmol, 2 eq) was added and the reaction mixture was stirred at 85 ºC for 48 h until LC-MS indicated disappearance of the starting materials. The reaction mixture was concentrated *in vacuo* and the residue was purified via flash column chromatography (silica gel, 40% dichloromethane in hexanes), yielding *tert*-butyl (6-methoxyquinolin-8-yl)(pent-4-en-1-yl)carbamate (446 mg, 93% yield) as a white foam.

The ^{1}H NMR spectrum matched with previously reported spectra. [16]

Physical state: white foam. $\mathbf{R_f}$ = 0.4 (20% ethyl acetate in hexanes, vis. UV).

^{1}H NMR (500 MHz, $CDCl_3$) δ 8.97 (s, 1H), 8.62 (dd, *J* = 4.3, 1.7 Hz, 1H), 8.22 – 8.06 (m, 1H), 8.00 (dd, *J* = 8.2, 1.7 Hz, 1H), 7.37 (dd, *J* = 8.2, 4.1 Hz, 1H), 6.71 (d, *J* = 2.6 Hz, 1H), 3.91 (s, 3H), 1.57 (s, 9H).

To a flame-dried 25 mL round-bottom flask equipped with a magnetic stir bar was added *tert*-butyl (6-methoxyquinolin-8-yl) carbamate (160 mg, 0.583 mmol, 1 eq) and anhydrous tetrahydrofuran (5 mL). Sodium hydride (34.8 mg, 60% in mineral oil, 0.875 mmol, 1.5 eq) was added portion-wise at 0 ºC. After addition, the reaction was placed under argon, and the reaction mixture stirred for 1 h at 0 ºC, followed by addition of 5-bromopent-1-ene (869 mg, 691 µL, 5.80 mmol, 10 eq). The mixture was then stirred under reflux for 48 h, until TLC indicated the disappearance of starting materials. The reaction mixture was cooled to room temperature, quenched with aq. NH_4Cl (5 mL), the resulting mixture poured into a separatory funnel. Water (50 mL) was added, and the mixture extracted with ethyl acetate (20 mL x 3), washed with brine (25 mL), dried over *anhyd.* Na_2SO_4, and concentrated *in vacuo*. The residue was purified via flash column chromatography (silica gel, 2% to 10% ethyl acetate in hexanes), affording *tert*-butyl (6-methoxyquinolin-8-yl)(pent-4-en-1-yl) carbamate (146 mg, 73% yield) as a light brown gum-like solid.

Physical state: light brown gum-like solid. $\mathbf{R_f}$ = 0.2 (10% ethyl acetate in hexanes, vis. UV, blue under UV light).

^{1}H NMR (500 MHz, $CDCl_3$) δ 8.78 (dd, *J* = 4.2, 1.8 Hz, 1H), 8.04 (dd, *J* = 8.4, 1.7 Hz, 1H), 7.35 (dd, *J* = 8.2, 4.1 Hz, 1H), 7.23 (s, 1H), 7.01 (d, *J* = 2.7 Hz, 1H), 5.90 – 5.59 (m, 1H), 5.07 – 4.79 (m, 2H), 3.93 (s, 3H), 3.50-4.17 (br, m, 2H), 2.21 – 2.03 (m, 2H), 1.72 – 1.59 (m, 2H), 1.30 (s, 9H).

^{13}C NMR (126 MHz, $CDCl_3$) δ 157.2, 155.6, 147.5, 141.0, 138.2, 134.9, 130.0, 121.6, 114.7, 104.4, 79.7, 55.6, 49.9, 31.1, 28.2, 28.0.

HRMS: Calculated for $C_{20}H_{27}N_2O_3^+$ 343.2016 [M+H$^+$]; found 343.2023.

***tert*-butyl 4-vinylpiperidine-1-carboxylate**

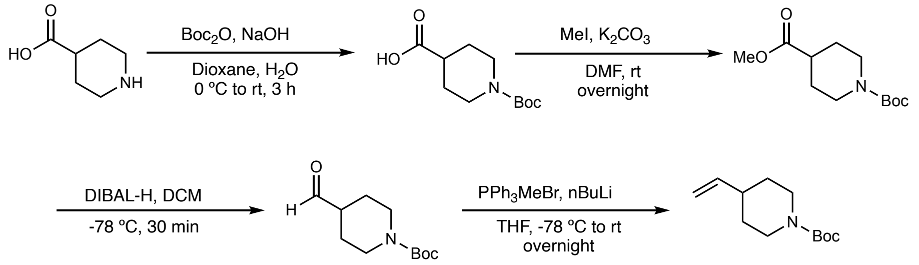

Step 1: Followed previously reported protocol. [17] To a 250 mL round-bottom flask equipped with a magnetic stir bar was added isonipecotic acid (3.00 g, 23.2 mmol, 1 eq), dioxane (46 mL), water (46 mL) and sodium hydroxide (920 mg, 23.0 mmol, 1 eq) sequentially. The reaction mixture was cooled to 0 ºC, followed by addition of Boc_2O (5.32 g, 5.60 mL, 24.39 mmol, 1.05 eq), then warmed up to room temperature and stirred for 3 h at room temperature. Dioxane was removed *in vacuo* and the residue was acidified with 3 N HCl to pH 3. The precipitate was collected by filtration, washed with cold water and dried on vacuum overnight. Obtained 1-(*tert*-butoxycarbonyl) piperidine-4-carboxylic acid (4.45 g, 84% yield) as a white solid.

The ^{1}H NMR spectrum matched with previously reported spectra. [17]

Physical state: white solid.

^{1}H NMR (500 MHz, $CDCl_3$) δ 4.02 (s, 2H), 2.86 (t, *J* = 12.2 Hz, 2H), 2.49 (tt, *J* = 10.9, 3.9 Hz, 1H), 1.98 – 1.86 (m, 2H), 1.64 (dtd, *J* = 13.4, 11.2, 4.3 Hz, 2H), 1.46 (s, 9H).

Step 2: Followed previously reported protocol. [18] To a 100 mL round-bottom flask equipped with a magnetic stir bar was added 1-(*tert*-butoxycarbonyl) piperidine-4-carboxylic acid (1.00 g, 4.36 mmol, 1 eq) and dimethylformamide (20 mL). Potassium carbonate (662 mg, 4.80 mmol, 1.1 eq) was added, followed by methyl iodide (325 µL, 5.23 mmol, 1.2 eq), then the reaction mixture was stirred at room temperature overnight. Water (100 mL) was added and extracted with ethyl acetate (30 mL x 3), washed with brine (50 mL), dried over *anhyd.* Na_2SO_4 and concentrated *in vacuo*. The residue was purified via flash column chromatography (silica gel, 2% to 10% ethyl acetate in hexanes), affording 1-(*tert*-butyl) 4-methyl piperidine-1,4-dicarboxylate (1.12 g, 106% yield, contain solvents) as a light brown oil.

The ^{1}H NMR spectrum matched with previously reported spectra. [18]

Physical state: light brown oil. **R_f** = 0.5 (25% ethyl acetate in hexane, vis. PMA).

^{1}H NMR (500 MHz, $CDCl_3$) δ 4.01 (s, 2H), 3.68 (s, 3H), 2.82 (t, *J* = 12.5 Hz, 2H), 2.44 (tt, *J* = 11.0, 3.9 Hz, 1H), 1.86 (dd, *J* = 12.9, 4.3 Hz, 2H), 1.62 (dtd, *J* = 13.4, 11.4, 4.3 Hz, 2H), 1.45 (s, 9H).

Step 3: To a flame-dried 100 mL round-bottom flask equipped with a magnetic stir bar was added 1-(*tert*-butyl) 4-methyl piperidine-1,4-dicarboxylate (1.04 g, 4.27 mmol, 1 eq) and anhydrous

dichloromethane (20 mL) at -78 ºC. DIBAL-H (1.0 M in heptane, 4.27 mL, 4.27 mmol, 1 eq) was added slowly, then the reaction mixture stirred at -78 ºC for 30 min followed by addition of 3 N HCl (20 mL) to quench the reaction. The reaction mixture was poured into a separatory funnel, extracted with dichloromethane (20 mL x 2), washed with *sat*. $NaHCO_3$ (50 mL), brine (25 mL), dried over *anhyd.* Na_2SO_4 and concentrated *in vacuo.* The residue was purified by flash column chromatography (silica gel, 15% ethyl acetate in hexanes), affording *tert*-butyl 4-formylpiperidine-1-carboxylate (456 mg, 51% yield) as a white solid.

The ^1H NMR spectrum matched with previously reported spectra. [19]

Physical state: colorless oil. $\mathbf{R_f}$ = 0.2 (25% ethyl acetate in hexane, vis. PMA).

^{1}H NMR (500 MHz, $CDCl_3$) δ 9.66 (d, *J* = 1.0 Hz, 1H), 3.98 (s, 2H), 2.92 (ddd, *J* = 13.9, 10.8, 3.0 Hz, 2H), 2.41 (ttd, *J* = 10.7, 4.0, 1.1 Hz, 1H), 1.89 (dd, *J* = 12.5, 4.9 Hz, 2H), 1.56 (tdd, *J* = 10.8, 7.5, 5.4 Hz, 2H), 1.45 (s, 9H).

Step 4: To a flame-dried 100 mL round-bottom flask equipped with a magnetic stir bar was added triphenylphosphine methyl bromide (1.66 g, 4.64 mmol, 2.2 eq) and anhydrous tetrahydrofuran (20 mL). After cooling to -78 ºC, *n*BuLi (1.06 M, 3.98 mL, 4.22 mmol, 2 eq) was added, followed by stirring at -78 ºC for 1 h to afford a yellow solution. *t*-Butyl 4-formylpiperidine-1-carboxylate (450 mg, 2.11 mmol, 1 eq) was added slowly as a solution in tetrahydrofuran (5 mL), then the reaction mixture warmed room temperature slowly and stirred overnight. Water (100 mL) was added then extracted with dichloromethane (30 mL x 3), washed with brine (50 mL), dried over *anhyd.* Na_2SO_4, concentrated *in vacuo*, and the residue purified via flash column chromatography (silica gel, 5% ethyl acetate in hexanes) yielding *tert*-butyl 4-vinylpiperidine-1-carboxylate (290 mg, 65% yield).

The ^1H NMR spectrum matched with previously reported spectra. [15]

Physical state: colorless oil. $\mathbf{R_f}$ = 0.6 (10% ethyl acetate in hexanes, vis. PMA or $KMnO_4$).

^{1}H NMR: (600 MHz, $CDCl_3$) δ 5.76 (ddd, *J* = 17.1, 10.4, 6.4 Hz, 1H), 5.14 – 4.86 (m, 2H), 4.08 (d, *J* = 12.8 Hz, 2H), 2.81 – 2.62 (m, 2H), 2.20 – 2.01 (m, 1H), 1.71 – 1.64 (m, 2H), 1.45 (d, *J* = 1.1 Hz, 9H), 1.32 – 1.21 (m, 2H).

1-tosyl-4-vinylpiperidine

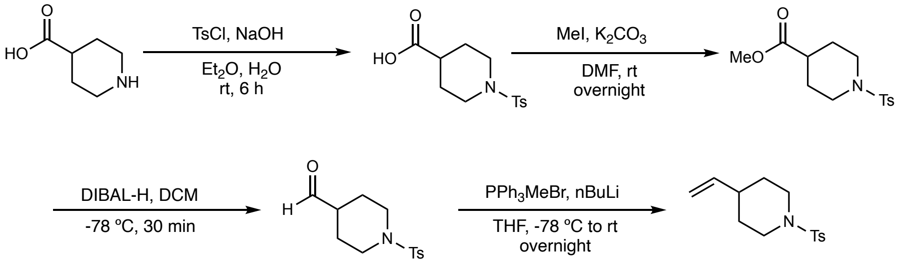

Step 1: Hu's protocol was followed with modifications. [20] To a 250 mL round-bottom flask equipped with a magnetic stir bar was added isonipecotic acid (6.45 g, 49.9 mmol, 1 eq), ether (50 mL), water (50 mL) and sodium hydroxide (4.00 g, 100 mmol, 2 eq) sequentially. Tosyl chloride (9.55 g, 49.9 mmol, 1 eq) was added portion-wise, then the mixture stirred for 6 h. More ether (50 mL) and H_2O (50 mL) added until the system became clear. The layers were separated, the aqueous layer acidified to pH 3 with 3 M HCl, the white precipitate collected by filtration and dried on vacuum overnight to afford a white solid (5.94 g, 42% yield).

The ^{1}H NMR spectrum matched with previously reported spectra. [20]

Physical state: white solid.

^{1}H NMR: (500 MHz, $CDCl_3$) δ 7.69 – 7.57 (m, 2H), 7.41 – 7.29 (m, 2H), 3.64 (dt, J = 12.1, 4.0 Hz, 2H), 2.52 – 2.40 (m, 5H), 2.29 (tt, J = 10.7, 4.0 Hz, 1H), 2.04 – 1.95 (m, 2H), 1.90 – 1.76 (m, 2H).

Step 2: To a 250 mL round-bottom flask equipped with a magnetic stir bar was added 1-tosylpiperidine-4-carboxylic acid (2.49 g, 8.79 mmol, 1 eq), dimethylformamide (50 mL) and potassium carbonate (1.33 g, 9.64 mmol, 1.1 eq). Methyl iodide (1.50 g, 657 μL, 10.6 mmol, 1.2 eq) was added and the reaction stirred at room temperature overnight. The reaction mixture was poured into water (400 mL), the precipitate was collected by filtration and dried on vacuum, affording a white solid (2.77 g, 105% yield, contains a little water).

The ^{1}H NMR spectrum matched with previously reported spectra. [21]

Physical state: White solid. **R_f** = 0.2 (17% ethyl acetate in hexanes, vis. PMA and UV).

^{1}H NMR: (500 MHz, $CDCl_3$) δ 7.70 – 7.55 (m, 2H), 7.36 – 7.28 (m, 2H), 3.69 – 3.58 (m, 5H), 2.52 – 2.39 (m, 5H), 2.25 (tt, J = 10.7, 4.0 Hz, 1H), 2.02 – 1.91 (m, 2H), 1.90 – 1.74 (m, 2H).

Step 3: To a flame-dried 100 mL round-bottom flask equipped with a magnetic stir bar was added methyl 1-tosylpiperidine-4-carboxylate (2.00 g, 6.72 mmol, 1 eq) and anhydrous dichloromethane (40 mL). The reaction mixture was cooled to -78 ºC, then DIBAL-H (1 M in heptane, 7.06 mL, 7.06 mmol, 1 eq) was added slowly. The reaction mixture was stirred at -78 ºC for 30 min then quenched with 3 N HCl (20 mL). The reaction mixture was poured into separatory funnel, extracted with dichloromethane (20 mL x 2), washed with *sat.* $NaHCO_3$ (50 mL), brine (25 mL),

dried over *anhyd.* Na_2SO_4 and concentrated *in vacuo*. The residue was purified by flash column chromatography (silica gel, 17% to 25% ethyl acetate in hexanes), affording (1.08 g, 60% yield) as a colorless oil, which solidified slowly on standing.

The ^{1}H NMR spectrum matched with previously reported spectra. [22]

Physical state: white solid. $\mathbf{R_f}$ = 0.2 (25% ethyl acetate in hexanes, vis. PMA and UV).

^{1}H NMR: (500 MHz, $CDCl_3$) δ 9.54 (s, 1H), 7.58 (d, *J* = 8.4 Hz, 2H), 7.27 (d, *J* = 8.2 Hz, 2H), 3.51 – 3.42 (m, 2H), 2.55 (ddd, *J* = 11.9, 10.1, 3.1 Hz, 2H), 2.38 (s, 3H), 2.22 – 2.13 (m, 1H), 2.00 – 1.89 (m, 2H), 1.71 (dtd, *J* = 13.9, 10.1, 4.0 Hz, 2H).

Step 4: To a flame-dried 100 mL round-bottom flask equipped with a magnetic stir bar was added triphenylphosphine methyl bromide (2.52 g, 7.05 mmol, 2.2eq) and anhydrous tetrahydrofuran (40 mL). After cooling to -78 ºC, *n*BuLi (1.06 M, 6.05 mL, 6.41 mmol, 2 eq) was added, followed by stirring at -78 ºC for 1 h to afford a yellow solution. 1-Tosylpiperidine-4-carbaldehyde (857 mg, 3.20 mmol, 1 eq) was added slowly as a solution in tetrahydrofuran (5 mL), then the reaction mixture warmed room temperature slowly and stirred overnight. Water (100 mL) was added then extracted with dichloromethane (30 mL x 3), washed with brine (50 mL), dried over *anhyd.* Na_2SO_4, concentrated *in vacuo*, and the residue purified via flash column chromatography (silica gel, 5% ethyl acetate in hexanes) yielding 1-tosyl-4-vinylpiperidine (590 mg, 67% yield) as a white solid.

The ^{1}H NMR spectrum matched with previously reported spectra. [15]

Physical state: colorless oil. $\mathbf{R_f}$ = 0.5 (17% ethyl acetate in hexanes, vis. UV, PMA or $KMnO_4$).

^{1}H NMR: (500 MHz, $CDCl_3$) δ 7.64 – 7.55 (m, 2H), 7.32 – 7.24 (m, 2H), 5.66 (ddd, *J* = 17.1, 10.5, 6.4 Hz, 1H), 5.02 – 4.73 (m, 2H), 3.84 – 3.60 (m, 2H), 2.38 (s, 3H), 2.23 (td, *J* = 11.9, 2.7 Hz, 2H), 1.90 – 1.78 (m, 1H), 1.73 – 1.65 (m, 2H), 1.43 (dtd, *J* = 13.4, 11.8, 4.1 Hz, 2H).

Methyl 3,7-dimethyloct-6-enoate

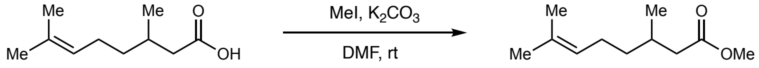

To a 50 mL round bottom flask equipped with a magnetic stir bar was added citronellic acid (1.00 g, 5.87 mmol, 1 eq), dimethylformamide (10 mL) and potassium carbonate (1.62 g, 11.8 mmol, 2 eq). The mixture was stirred for 10 min before methyl iodide (1.67 g, 731 µL, 11.8 mmol, 2 eq) was added slowly and stirred for 2 h until LC-MS indicated the disappearance of starting materials. Water (100 mL) was added and extracted with ethyl acetate (20 mL x 3), washed with 5% sodium hydroxide (25 mL) and brine (50 mL), dried over *anhyd.* Na_2SO_4 and concentrated *in vacuo*. The residue was purified via flash column chromatography (silica gel, 3% ethyl acetate in hexanes) yielding methyl 3,7-dimethyloct-6-enoate (674 mg, 62% yield) as a colorless oil.

The ^{1}H NMR spectrum matched with previously reported spectra. [23]

Physical state: colorless oil; $\mathbf{R_f}$ = 0.5 (20% ethyl acetate in hexanes, vis iodine).

^{1}HNMR: (600 MHz, $CDCl_3$) δ 5.08 (t, J = 5.7 Hz, 1H), 3.66, (s, 3H), 2.32 (dd, J = 5.7, 14.9 Hz, 1H), 2.12 (dd, J = 8.6, 14.9 Hz, 1H), 2.01-1.94 (m, 3H), 1.67 (s, 3H), 1.59 (s, 3H), 1.37-1.18 (m, 2H), 0.94 (d, J = 6.3 Hz, 3H).

4-Methoxybenzyl 3,7-dimethyloct-6-enoate

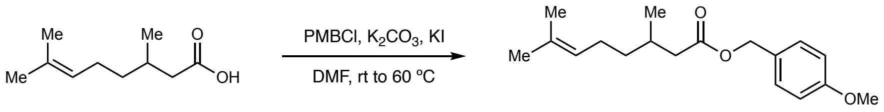

To a 50 mL round bottom flask equipped with a magnetic stir bar was added citronellic acid (1.00 g, 5.87 mmol, 1 eq), dimethylformamide (10 mL) and potassium carbonate (1.62 g, 11.8 mmol, 2 eq). The reaction mixture was stirred for 10 min, followed by addition of 4-methoxybenzyl chloride (1.84 g, 11.8 mmol, 2 eq) and potassium iodide (cat.). The reaction mixture was then stirred at 60 ºC for 2 h until TLC indicated disappearance of starting materials. Water (100 mL) was added and extracted with ethyl acetate (20 mL x 3), washed with 5% sodium hydroxide (25 mL) and brine (50 mL), dried over *anhyd.* Na_2SO_4 and concentrated *in vacuo*. The residue was purified via flash column chromatography (silica gel, 1% to 3% ethyl acetate in hexanes) yielding 4-methoxybenzyl 3,7-dimethyloct-6-enoate (1.29 g, 76% yield) as a colorless oil.

Physical state: colorless oil; $\mathbf{R_f}$ = 0.4 (10% ethyl acetate in hexanes, vis UV and CAM).

^{1}H NMR (500 MHz, $CDCl_3$) δ 7.29 (d, J = 8.7 Hz, 2H), 6.88 (d, J = 8.7 Hz, 2H), 5.13 – 4.97 (m, 3H), 3.81 (s, 3H), 2.33 (dd, J = 14.6, 6.0 Hz, 1H), 2.14 (dd, J = 14.6, 8.2 Hz, 1H), 2.03 – 1.90 (m, 3H), 1.67 (t, J = 1.3 Hz, 3H), 1.58 (d, J = 1.1 Hz, 4H), 1.33 (ddt, J = 13.4, 9.5, 6.3 Hz, 1H), 1.20 (dddd, J = 13.6, 9.3, 7.8, 6.0 Hz, 1H), 0.93 (d, J = 6.7 Hz, 3H).

^{13}C NMR (126 MHz, $CDCl_3$) δ 173.3, 159.7, 131.6, 130.2, 128.4, 124.4, 114.0, 66.0, 55.4, 42.0, 36.90, 30.2, 25.8, 25.5, 19.7, 17.8.

HRMS: Calculated for $C_{18}H_{27}O_3^+$ 291.1960 [M+H$^+$]; found 291.1958.

***tert*-Butyl (4-(but-3-en-1-yloxy)phenyl)carbamate**

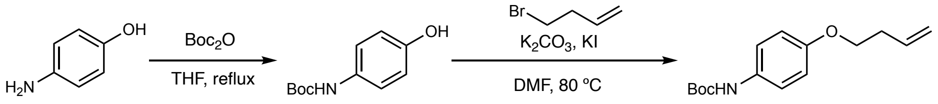

Step 1: To a 500 mL round bottom flask equipped with a magnetic stir bar was added 4-aminophenol (5.00 g, 45.8 mmol, 1 eq) and anhydrous tetrahydrofuran (100 mL). Boc_2O (11.0 g, 50.4 mmol, 1.1 eq) in anhydrous tetrahydrofuran (50 mL) was added dropwise. The reaction mixture was stirred vigorously at room temperature for 24 h and concentrated *in vacuo*, affording *tert*-butyl (4-(but-3-en-1-yloxy)phenyl)carbamate in quantitative yield and was used without further purification.

The ^1H NMR spectrum matched with previously reported spectra. [24]

^{1}H NMR: (500 MHz, DMSO) δ 9.04 (s, 1H), 8.97 (s, 1H), 7.18 (d, *J* = 8.3 Hz, 2H), 6.65 – 6.58 (m, 2H), 1.43 (s, 9H).

Step 2: To a 25 mL round-bottom flask equipped with a magnetic stir bar was added *tert*-butyl (4-hydroxyphenyl)carbamate (1.20 g, 5.73 mmol, 1 eq) and dimethylformamide (6 mL). 1-Bromobut-3-ene (1.70 g, 1.28 mL, 12.6 mmol, 2.2 eq) and potassium iodide (cat.) were added, then the reaction mixture was stirred at 80 ºC for 24 h. Water (50 mL) was added and extracted with ethyl acetate (50 mL x 3), washed with brine (50 mL), dried over *anhyd.* Na_2SO_4 and the solvent removed *in vacuo*. The residue was purified via flash column chromatography (silica gel, 5% to 10% ethyl acetate in hexanes) yielding *tert*-butyl (4-(but-3-en-1-yloxy)phenyl)carbamate (490 mg, 32% yield) as a white solid.

Physical state: white solid; **R_f** = 0.3 (10% ethyl acetate in hexanes, vis UV and CAM).

^{1}H NMR (500 MHz, $CDCl_3$) δ 7.27 (d, *J* = 8.1 Hz, 2H), 6.89 – 6.82 (m, 2H), 6.34 (s, 1H), 5.92 (ddt, *J* = 17.1, 10.4, 6.7 Hz, 1H), 5.23 – 5.05 (m, 2H), 4.00 (t, *J* = 6.7 Hz, 2H), 2.54 (qt, *J* = 6.7, 1.4 Hz, 2H), 1.53 (s, 9H).

^{13}C NMR (126 MHz, $CDCl_3$) δ 155.1, 153.3, 134.6, 131.6, 120.6, 117.1, 115.2, 80.4, 67.7, 33.8, 28.5.

HRMS: Calculated for de-*tert*-butyl compound $C_{11}H_{14}NO_3^+$ 208.0968 [M+H$^+$]; found 208.0973.

4-(But-3-en-1-yloxy)aniline

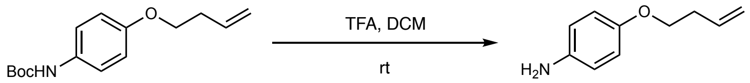

To a 25 mL round-bottom flask equipped with a magnetic stir bar was added 1-(4-*N*-Boc-aminophenoxy)-3-butene (788 mg, 2.99 mmol, 1 eq) and dichloromethane (8 mL). Trifluoroacetic acid (2 ml) was added, and the reaction mixture stirred at room temperature for 30 min. The reaction mixture was concentrated *in vacuo*, and the residue was diluted with dichloromethane, basified with *sat.* $NaHCO_3$, extracted with dichloromethane (20 mL x 3), dried over *anhyd.* $MgSO_4$ and concentrated *in vacuo*. The residue was purified via flash column chromatography (silica gel, 0 – 5% methanol in DCM) to afford 4-(but-3-en-1-yloxy)aniline (447 mg, 92% yield) as a yellow oil.

The ^{1}H NMR spectrum matched with previously reported spectra. [25]

Physical state: yellow oil; $\mathbf{R_f}$ = 0.5 (3% methanol in DCM, vis UV and CAM)

^{1}H NMR (500 MHz, $CDCl_3$) δ 6.84 – 6.71 (m, 2H), 6.68 – 6.54 (m, 2H), 5.90 (ddt, *J* = 17.1, 10.4, 6.7 Hz, 1H), 5.30 – 4.98 (m, 2H), 3.94 (t, *J* = 6.8 Hz, 2H), 3.41 (s, 2H), 2.50 (qt, *J* = 6.7, 1.5 Hz, 2H).

^{13}C NMR (126 MHz, $CDCl_3$) δ 152.2, 140.2, 134.8, 116.9, 116.6, 116.5, 116.0, 68.1, 34.0.

HRMS: Calculated for $C_{10}H_{14}NO^+$ 164.1070 [M+H$^+$]; found 164.1071.

***N*-(4-(But-3-en-1-yloxy)phenyl)acetamide**

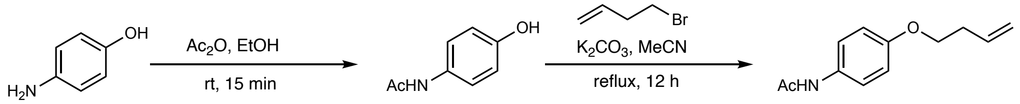

Step 1: To a 25 mL round-bottom flask equipped with a magnetic stir bar was added 4-aminophenol (500 mg, 4.58 mmol, 1 eq), absolute ethanol (5 mL) and acetic anhydride (467 mg, 433 µL, 4.58 mmol, 1 eq). The solution was stirred for 15 min at room temperature, then evaporated to dryness. The residue was purified via flash column chromatography (silica gel, 5% methanol in DCM) affording *N*-(4-hydroxyphenyl)acetamide (463 mg, 67% yield) as a white solid.

The ^{1}H NMR spectrum matched with previously reported spectra.[26]

Physical state: white solid; **R_f** = 0.1 (50% EA in hexane, vis UV and CAM)

^{1}H NMR: (600 MHz, DMSO) δ 9.67 (s, 1H), 9.15 (s, 1H), 7.36 (d, *J* = 8.9 Hz, 1H), 6.70 (d, *J* = 8.8 Hz, 1H), 2.01 (s, 3H).

Step 2: To a 25 mL round bottom flask equipped with a magnetic stir bar was added *N*-(4-hydroxyphenyl)acetamide (290 mg, 1.92 mmol, 1 eq), potassium carbonate (398 mg, 2.88 mmol, 1.5 eq) and acetonitrile (10 mL). 1-Bromobut-3-ene (518 mg, 390 µL, 3.84 mmol, 2 eq) was added, and the reaction mixture stirred under reflux for 12 h, concentrated *in vacuo*, diluted with water (10 mL), extracted with ethyl acetate (25 mL x 3), washed with brine, dried over $MgSO_4$, and concentrated *in vacuo*. The residue was purified via flash column chromatography (silica gel, 15 – 100% ethyl acetate in hexanes) affording *N*-(4-(but-3-en-1-yloxy)phenyl)-acetamide (148 mg, 38% yield).

The ^{1}H NMR spectrum matched with previously reported spectra.[27]

Physical state: off white solid. **R_f** = 0.20 (50 % ethyl acetate in hexanes, vis iodine)

^{1}H NMR: (400 MHz, $CDCl_3$) δ 7.41 – 7.34 (m, 2H), 7.15 (s, 1H), 6.90 – 6.82 (m, 2H), 5.89 (ddt, *J* = 17.1, 10.2, 6.7 Hz, 1H), 5.19-5.09 (m, 2H), 3.99 (t, *J* = 6.7 Hz, 2H), 2.53 (qt, *J* = 6.7, 1.4 Hz, 2H), 2.15 (s, 3H).

Benzyl (4-hydroxyphenyl) carbamate

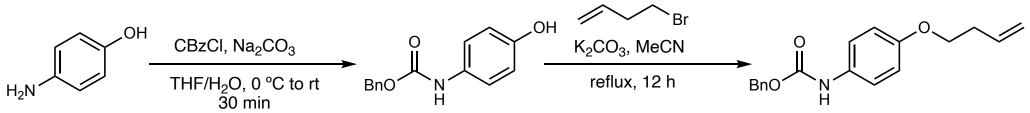

Step 1: To a 25 mL round-bottom flask equipped with a magnetic stir bar was added 4-aminophenol (250 mg, 2.29 mmol, 1 eq), water (2 mL), tetrahydrofuran (2 mL) and sodium carbonate (486 mg, 4.59 mmol, 2 eq). The reaction mixture was cooled to 0 °C, followed by the addition of benzyl chloroformate (406 mg, 340 µL, 2.38 mmol, 1.04 eq) in tetrahydrofuran (1 mL) dropwise over 15 min. The brown suspension was then stirred for 30 min at room temperature and concentrated *in vacuo*. The residue was diluted with water (10 mL) and extracted with ethyl acetate (10 mL x 3), washed with brine (10 mL x 2), dried over *anhyd.* Na_2SO_4 and concentrated *in vacuo*. The crude product was recrystallized from ethyl acetate to yield benzyl (4-hydroxyphenyl)carbamate (347 mg, 62% yield).

The ^1H NMR spectrum matched with previously reported spectra. [28]

Physical state: off white solid **R_f**= 0.3 (25% ethyl acetate in hexanes, vis. UV).

^{1}H NMR (500 MHz, $CDCl_3$) δ 7.45 – 7.30 (m, 5H), 7.23 (d, *J* = 8.1 Hz, 2H), 6.81 – 6.75 (m, 2H), 6.51 (s, 1H), 5.19 (s, 2H), 4.70 (s, 1H).

Step 2: Followed the same procedure as above from benzyl (4-hydroxyphenyl)carbamate on a 0.740 mmol scale. Purification via flash column chromatography (silica gel, 5 – 20% ethyl acetate in hexanes) afforded benzyl (4-(but-3-en-1-yloxy)phenyl)carbamate (93.1 mg, 42% yield).

Physical state: off white solid. **R_f** = 0.7 (25 % ethyl acetate in hexanes, vis. UV and CAM).

^{1}H NMR (500 MHz, $CDCl_3$) δ 7.49 – 7.12 (m, 8H), 6.95 – 6.78 (m, 2H), 6.53 (s, 1H), 5.90 (ddt, *J* = 17.1, 10.2, 6.7 Hz, 1H), 5.27 – 5.07 (m, 4H), 3.99 (t, *J* = 6.7 Hz, 2H), 2.62 – 2.33 (m, 2H).

^{13}C NMR (126 MHz, $CDCl_3$) δ 155.5, 153.8, 136.3, 134.6, 131.0, 128.8, 128.5, 120.7, 117.2, 115.2, 67.7, 67.1, 33.8.

HRMS: calculated for $C_{18}H_{20}NO_3$ 298.1438 [M+H$^+$]; found 298.1431.

[1.1.1]Propellane solution in pentane/ether

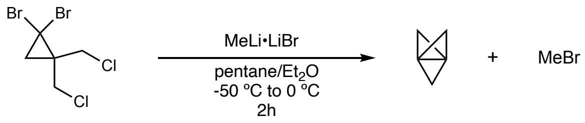

Followed previously reported protocol. [29] To a 50 mL 3 neck-round-bottom flask equipped with a magnetic stir bar and a thermometer under argon was added 1,1-dibromo-2,2-bis(chloromethyl)cyclopropane (2.96 g, 9.97 mmol, 1 eq), followed by anhydrous ether (0.36 mL) and pentane (2.4 mL). The reaction mixture was cooled to -50 ºC with an isopropanol/dry ice bath, then methyllithium-lithium bromide complex (1.5 M, 16.0 mL, 24.0 mmol, 2.4 eq) was added slowly with a syringe. (Keep the temperature inside between -40 ºC to -50 ºC.) Isopropanol/dry ice bath was removed after addition and the reaction mixture warmed to 0 ºC and stirred for 2 h with an ice bath. A short path distilling head was attached, with a 25 mL round-bottom flask as a receiving flask (cooling with an acetone/dry ice bath). A vacuum was slowly applied, collected [1.1.1]propellane as a solution in ether and pentane (~15 mL, 0.452 M), which was stored at -80 ºC before use. (The concentration of [1.1.1]propellane was determined by titration with thiophenol.)

The ^1H NMR spectrum matched with previously reported spectra. [29]

^{1}H NMR (500 MHz, $CDCl_3$) δ 2.04 (s, 6H).

Optimization Experiments:

Supplementary Table 1: Hydroamination reaction optimization with 4-phenylbut-1-ene

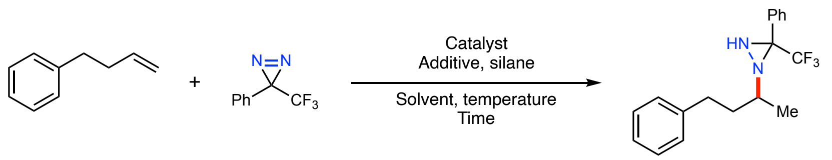

Entry	Diazirine (equiv)	Catalyst (mol%)	Additive (equiv)	Silane (equiv)	Solvent temp (°C), time (h)	Yield (%)
1	1.5	**cat-4**, (5)	none	$PhSiH_3$ (0.5)	DCM/IPA 4:1 40 °C, 16 h	35
2	1.5	**cat-4**, (5)	none	$(SiMe_2H)_2O$, (0.5)	DCM/IPA 4:1 40 °C, 16 h	24
3	1.5	**cat-4**, (5)	none	TTMS, (0.5)	DCM/IPA 4:1 40 °C, 16 h	ND
4	1.5	**cat-4**, (5)	none	PMHS, (0.1)	DCM/IPA 4:1 40 °C, 16 h	ND
5	1.5	$Mn(dpm)_3$, (5)	none	$PhSiH_3$ (0.5)	DCM/IPA 4:1 0 °C, 2 h	44/39
6	1.5	$Co(dpm)_3$, (5)	none	$PhSiH_3$ (0.5)	DCM/IPA 4:1 0 °C, 16 h	11
7	1.5	$Mn(acac)_2$, (5)	none	$PhSiH_3$ (0.5)	DCM/IPA 4:1 0 °C, 16 h	ND
8	1.5	$Co(acac)_2$, (5)	none	$PhSiH_3$ (0.5)	DCM/IPA 4:1 0 °C, 16 h	trace
9	1.5	$Fe(acac)_2$, (5)	none	$PhSiH_3$ (0.5)	DCM/IPA 4:1 0 °C, 16 h	trace
10	1.5	Co(TPP)Cl, (5)	none	$PhSiH_3$ (0.5)	DCM/IPA 4:1 0 °C, 16 h	trace
11	1.5	Mn(TPP)Cl, (5)	none	$PhSiH_3$ (0.5)	DCM/IPA 4:1 0 °C, 16 h	trace
12	1.5	$Co(OAc)_2$, (5)	none	$PhSiH_3$ (0.5)	DCM/IPA 4:1 0 °C, 48 h	trace
13	1.5	**cat-4**, (5)	none	$PhSiH_3$ (0.5)	DCE/IPA 4:1 40 °C, 16 h	54
14	2	**cat-4**, (10)	none	$PhSiH_3$ (0.5)	DCE/IPA 4:1 40 °C, 16 h	67
15	1.5	**cat-5**, (10)	none	$PhSiH_3$ (0.5)	DCE/IPA 4:1 40 °C, 16 h	trace
16	1.5	**cat-1**, (5)	none	$PhSiH_3$ (0.33)	DCE/IPA 4:1 40 °C, 16 h	47
17	1.5	**cat-1**, (5)	none	$(SiMe_2H)_2O$, (0.5)	DCE/IPA 4:1 40 °C, 16 h	35
18	1.5	**cat-6**, (5)	none	$PhSiH_3$ (0.5)	DCE/IPA 4:1 40 °C, 16 h	ND

Supplementary Table 2: Hydroamination reaction optimization with **2**

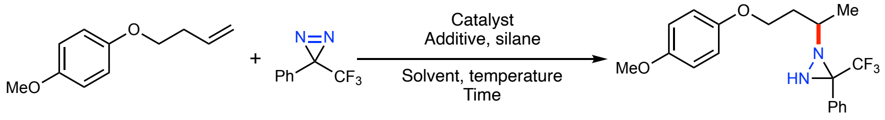

Entry	Diazirine (equiv)	Catalyst (mol%)	Additive (equiv)	Silane (equiv)	Solvent temp (ºC), time (h)	Yield (%)
1	1.5	**cat-4**, (5)	none	$PhSiH_3$ (0.3)	DCE/IPA 4:1 40 ºC, 16 h	27
2	1.5	**cat-5**, (5)	none	$PhSiH_3$ (0.3)	DCE/IPA 4:1 40 ºC, 16 h	trace
3	1.5	**cat-1**, (5)	none	$PhSiH_3$, (1)	DCE/IPA 4:1 40 ºC, 20 h	38
4	1.5	**cat-1**, (5)	t-BuOOH	$PhSiH_3$, (1)	DCE/IPA 4:1 40 ºC, 20 h	36
5	1.5	**cat-1**, (5)	t-BuOOt-Bu	$PhSiH_3$, (1)	DCE/IPA 4:1 40 ºC, 20 h	99
6	1.5	**cat-1**, (5)	t-BuOOt-Bu	$PhSiH_3$, (1)	IPA 40 ºC, 30 h	84
7	1.5	**cat-2**, (5)	t-BuOOt-Bu	$PhSiH_3$, (1)	DCE/IPA 4:1 40 ºC, 20 h	51
8	1.5	**cat-3**, (5)	t-BuOOt-Bu	$PhSiH_3$, (1)	DCE/IPA 4:1 40 ºC, 20 h	39
9	1.5	**cat-4**, (5)	t-BuOOt-Bu	$PhSiH_3$, (1)	DCE/IPA 4:1 40 ºC, 20 h	57
10	1.5	**cat-1**, (5)	t-BuOOt-Bu	$PhSiH_3$, (1)	DCE/IPA 4:1 60 ºC, 20 h	56
11	1.5	**cat-1**, (5)	t-BuOOt-Bu	$PhSiH_3$, (1)	DCE/IPA 4:1 40 ºC, 3 h	66
12	1.5	**cat-1**, (5)	t-BuOOt-Bu	$PhSiH_3$, (1)	DCE/IPA 4:1 40 ºC, 16 h	93
13	1.5	**cat-1**, (5)	t-BuOOt-Bu	TES, (1)	DCE/IPA 4:1 40 ºC, 20 h	trace
14	1.5	**cat-1**, (5)	t-BuOOt-Bu	PHMS, (1)	DCE/IPA 4:1 40 ºC, 20 h	trace
15	1.5	$Mn(dpm)_3$, (5)	none	$PhSiH_3$, (1)	DCE/IPA 4:1 0 ºC, 2 h	51/35
16	1.5	$Co(dpm)_3$, (5)	none	$PhSiH_3$, (1)	DCE/IPA 4:1 0 ºC, 16 h	trace
17	1.5	Co(TPP)Cl, (5)	none	$PhSiH_3$, (1)	DCE/IPA 4:1 0 ºC, 16 h	trace

Supplementary Table 3: List of catalysts

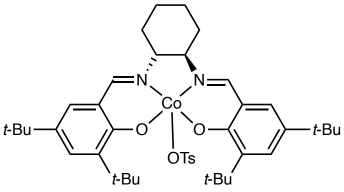

cat-1

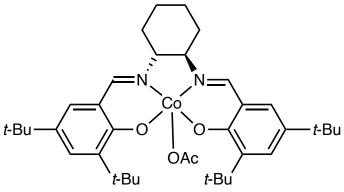

cat-2

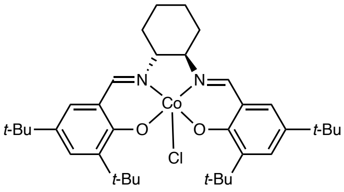

cat-3

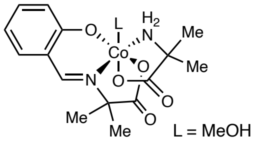

cat-4

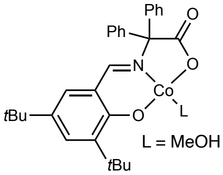

cat-5

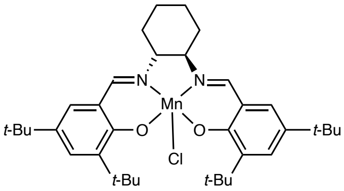

cat-6

Supplementary Table 4: Hydroamination reaction of chiral substrates with (*R,R*)-**cat-1** and (*S,S*)-**cat-1**

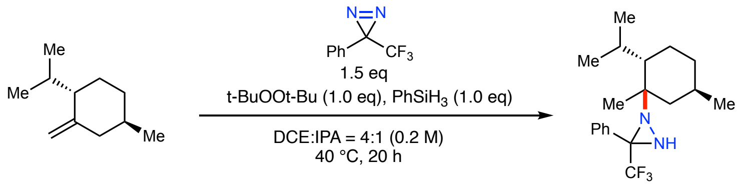

Entry	Catalyst	Yield (%)
1	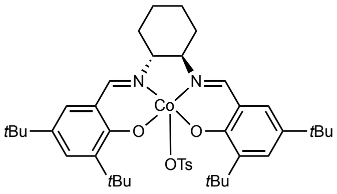 (R,R)-**cat-1**	42
2	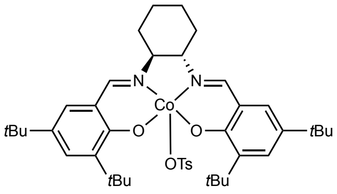 (S,S)-**cat-1**	41

General Procedure A: Cobalt Catalyzed Markovnikov-Type Hydroamination

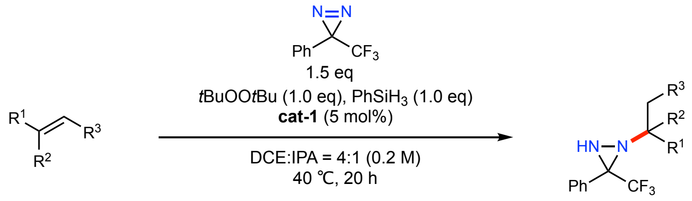

To a flame-dried reaction vial equipped with a magnetic stir-bar and rubber-lined cap under argon atmosphere was added catalyst **cat-1** (3.9 mg, 0.005 mmol, 0.05 eq) and the vial back-flushed with argon twice, followed by addition of a mixture of *anhydrous* DCE: IPA (4:1, 500 µL) via syringe, resulting in a dark green solution. To this solution, alkene (0.100 mmol, 1 eq.), diazirine **1** (28.0 mg, 0.150 mmol, 1.5 eq), *t*-BuOO*t*-Bu (14.6 mg, 18.4 µL, 0.100 mmol, 1 eq) and phenylsilane (10.8 mg, 12.3 µL, 0.100 mmol, 1 eq) were added sequentially via syringe. The vial was covered with aluminum foil and stirred at 40 °C for 20 h. The crude reaction mixture was dried *in vacuo*, adsorbed onto silica gel, and purified via flash column chromatography on silica gel.

(Note: Alternatively, the reaction mixture may also be quenched with water (10 mL), extracted with either dichloromethane or ethyl acetate (15 mL x 3), dried over anhydrous $MgSO_4$, concentrated *in vacuo,* and stored at -20 °C for later purification.)

General Procedure B: Cobalt Catalyzed Markovnikov-Type Hydroamination with IPA

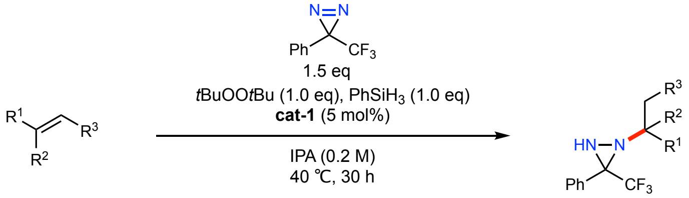

To a flame-dried reaction vial equipped with a magnetic stir-bar and rubber-lined cap under argon atmosphere was added catalyst **cat-1** (3.9 mg, 0.005 mmol, 0.05 eq) and the vial back-flushed with argon twice, followed by addition of *anhydrous* IPA (500 µL) via syringe, resulting in a dark green solution. To this solution, alkene (0.100 mmol, 1 eq.), diazirine **1** (28.0 mg, 0.150 mmol, 1.5 eq), *t*-BuOO*t*-Bu (14.6 mg, 18.4 µL, 0.100 mmol, 1 eq) and phenylsilane (10.8 mg, 12.3 µL, 0.100 mmol, 1 eq) were added sequentially via syringe. The vial was covered with aluminum foil and stirred at 40 °C for 20 h. The crude reaction mixture was dried *in vacuo*, adsorbed onto silica gel, and purified via flash column chromatography on silica gel.

(Note: Alternatively, the reaction mixture may also be quenched with water (10 mL), extracted with either dichloromethane or ethyl acetate (15 mL x 3), dried over anhydrous $MgSO_4$, concentrated *in vacuo,* and stored at -20 °C for later purification.)

Graphical Procedure for Co-catalyzed Synthesis of Substituted Diaziridines:

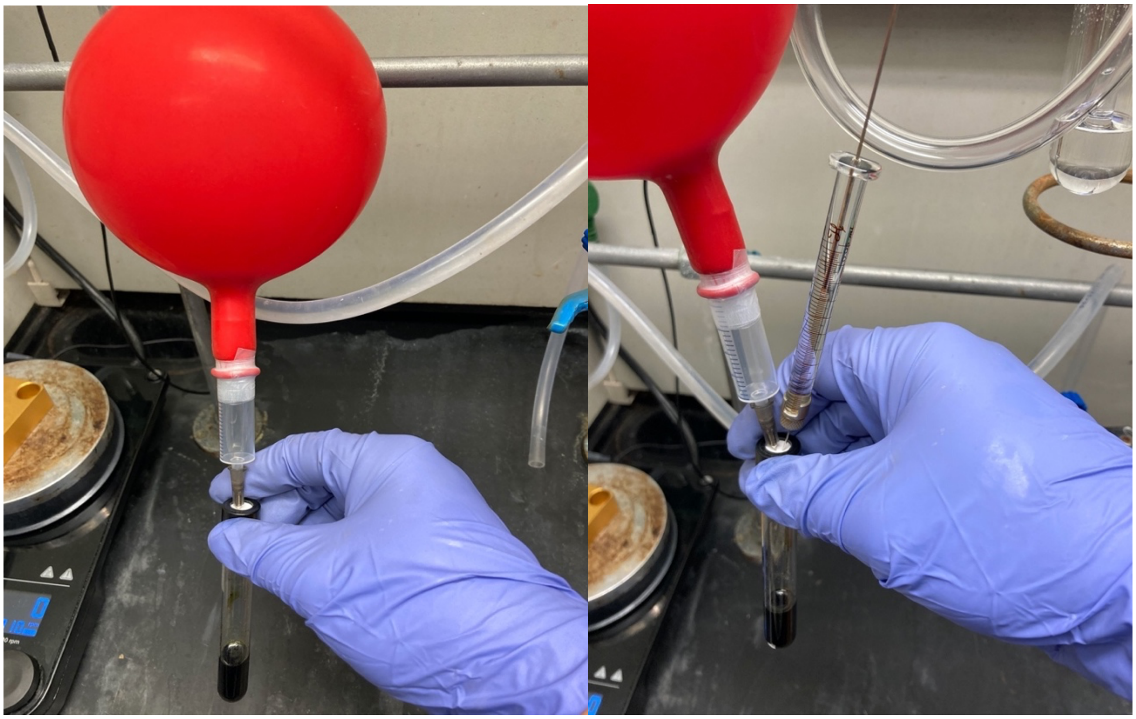

Supplementary Figure 3: Left: Addition of catalyst, alkene, and solvent to reaction vial. **Right**: Addition of diazirine **2** to reaction mixture.

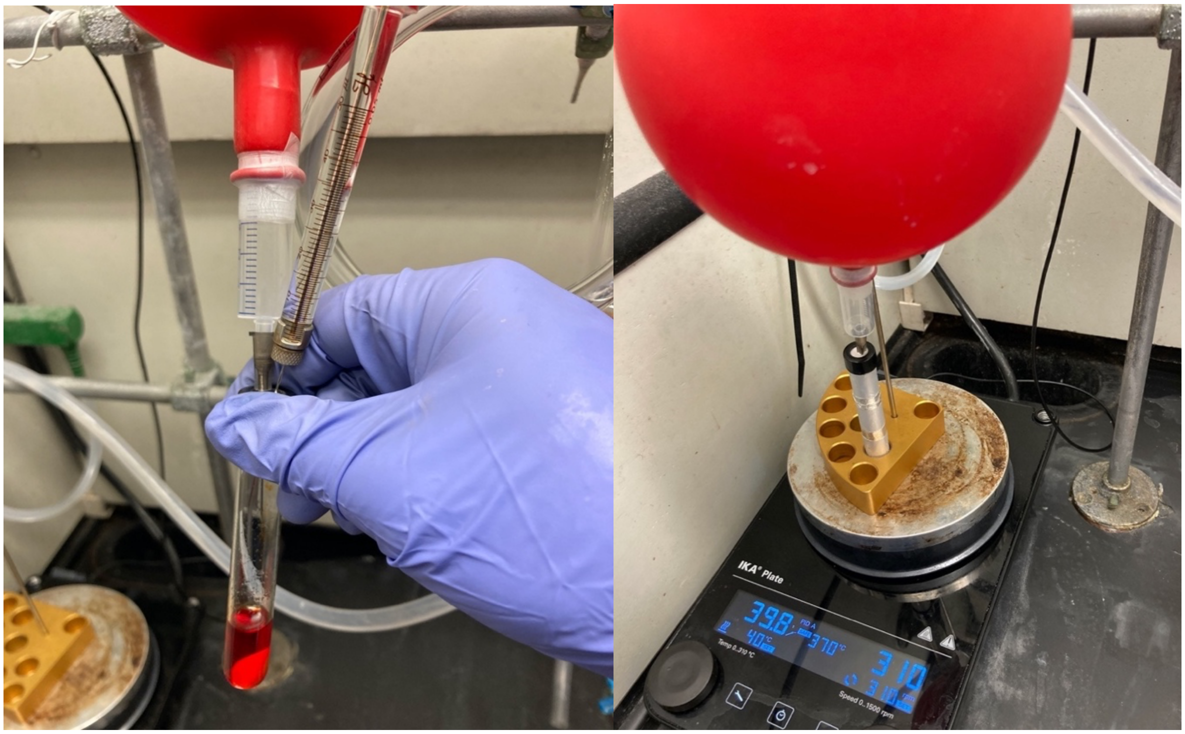

Supplementary Figure 4: Left: Addition of *t*-BuOO*t*-Bu and silane to reaction mixture; the color changed to red after silane addition. **Right:** Vial was covered with aluminum foil and heated with stirring.

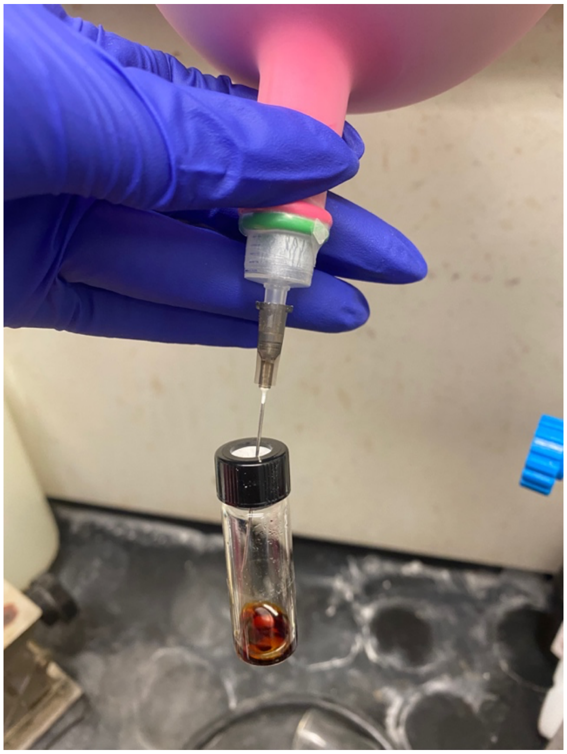

Supplementary Figure 5: Reaction mixture after stirring for 20 h at 40 ºC.

General Procedure C: Mn-Catalyzed Hydroamination

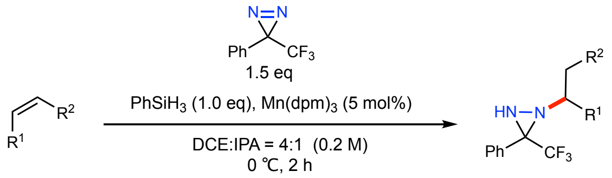

To a flame-dried reaction vial equipped with a magnetic stir-bar and rubber-lined cap under argon atmosphere was added $Mn(dpm)_3$ (3.0 mg, 0.005 mmol, 0.05 eq) and the vial back-flushed with argon, followed by addition of a mixture of anhydrous DCE: IPA (4:1, 500 µL) via syringe, resulting in a black suspension. To this mixture, alkene (0.100 mmol, 1 eq.), diazirine **1** (28.0 mg, 0.150 mmol, 1.5 eq), and phenylsilane (10.8 mg, 12.3 µL, 0.100 mmol, 1 eq) were added sequentially via syringe. The reaction vial was then cooled to 0 ºC in an ice bath and covered with aluminum foil and stirred for two hours until TLC monitoring indicated total consumption of the starting material. A color change from black to yellow or brown (substrate dependent) also indicated reaction completion. The crude reaction mixture was dried *in vacuo*, adsorbed onto silica gel, and purified via flash column chromatography on silica gel.

(Note: Alternatively, the reaction mixture may also be quenched with water (10 mL), extracted with either dichloromethane or ethyl acetate (15 mL x 3), dried over anhydrous $MgSO_4$, concentrated *in vacuo,* and stored at -20 °C for later purification.)

Graphical Procedure for Mn-Catalyzed Synthesis of Substituted Diaziridines:

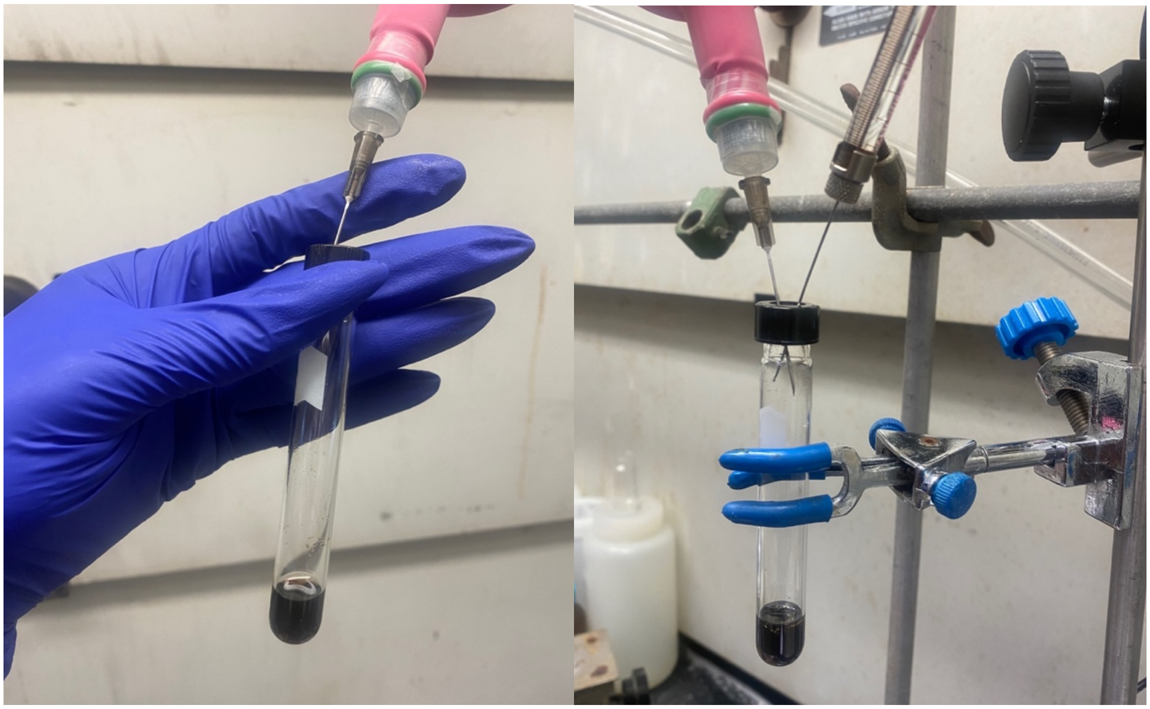

Supplementary Figure 6: Left: Addition of catalyst, alkene, and solvent to reaction vial. **Right:** Addition of diazirine **2** to reaction mixture.

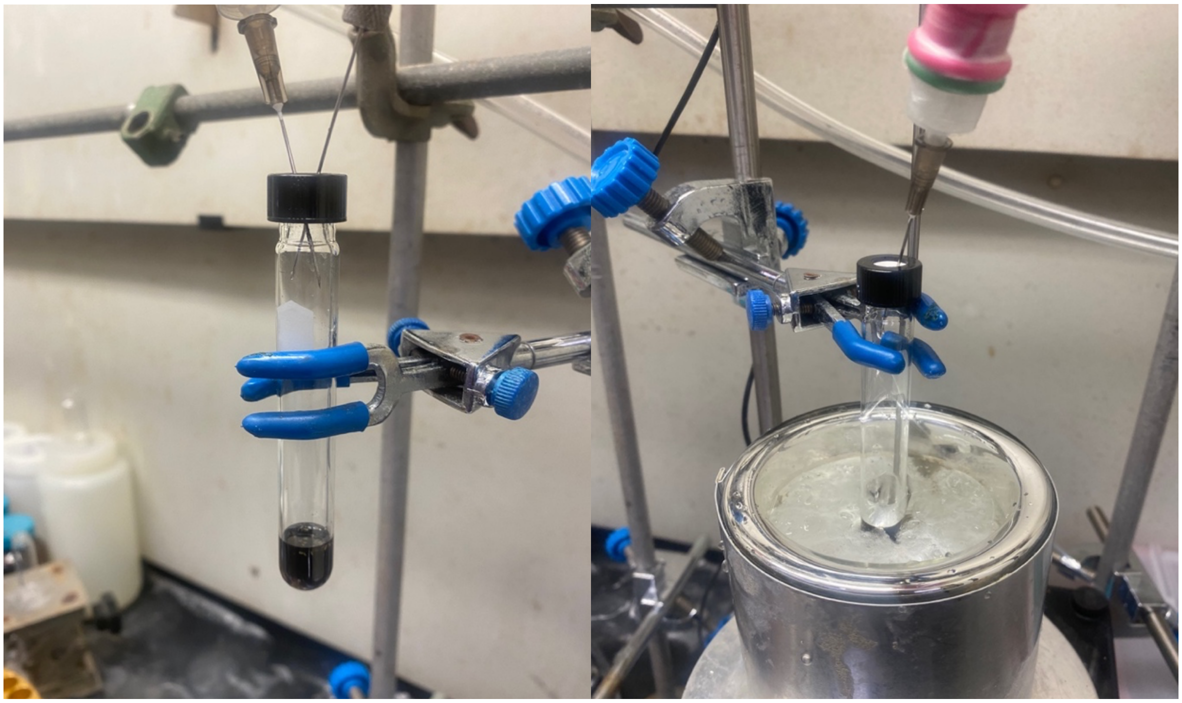

Supplementary Figure 7: Left: Addition of silane to reaction mixture. **Right:** Reaction mixture cooled to 0 ºC in an ice bath with stirring.

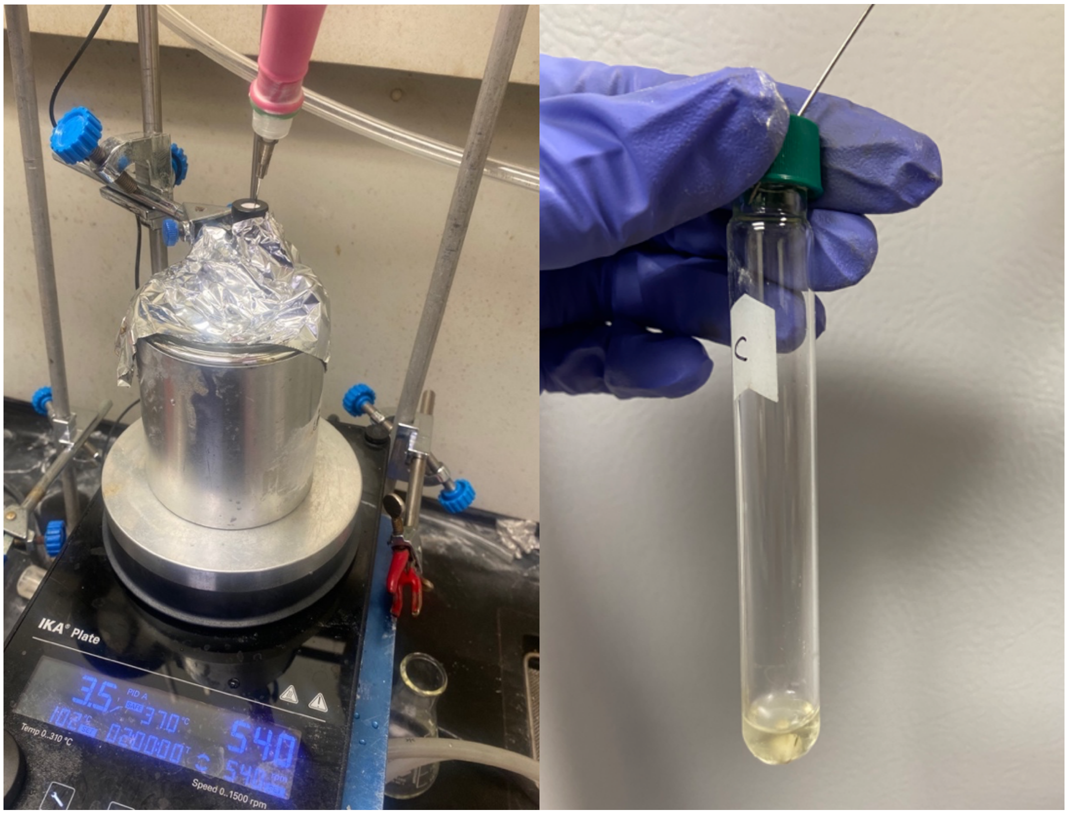

Supplementary Figure 8: Right: Vial covered with aluminum foil **Left:** Reaction mixture after stirring at 0 ºC for 2 h (light brown or yellow color change typically observed).

Supplementary Discussion:

Synthesis of Substituted Diaziridine Substrates:

1-(4-(4-methoxyphenoxy)butan-2-yl)-3-phenyl-3-(trifluoromethyl)diaziridine (3a)

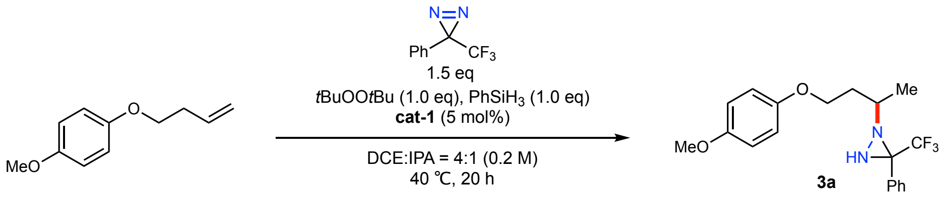

General procedure A was followed on a 0.100 mmol scale. Purification via column chromatography (silica gel, 0 – 10 % ethyl acetate in hexanes) afforded **3a** (36.2 mg, 99 % yield).

Physical state: dark yellow oil. R_f = 0.6 (10 % ethyl acetate in hexanes, vis. UV, iodine, and CAM).

^{1}H NMR (500 MHz, $CDCl_3$) δ 7.73 – 7.65 (m, 2H), 7.50 – 7.38 (m, 3H), 6.84 – 6.73 (m, 3H), 6.66 – 6.57 (m, 1H), 4.05 – 3.93 (m, 1H), 3.80 – 3.66 (m, 4H), 3.05 – 2.91 (m, 1H), 2.21 – 1.72 (m, 3H), 1.23 – 0.94 (m, 3H).

^{13}C NMR (126 MHz, $CDCl_3$) δ 153.9, 153.8, 153.2, 152.9, 130.3, 130.2, 130.1, 128.9, 128.5, 128.4, 128.3, 128.1, 128.0, 123.9 (q, J = 278.8 Hz), 123.8 (q, J = 278.8 Hz), 115.6, 115.3, 114.7, 114.7, 65.8, 64.8, 64.1 (q, J = 34.3 Hz), 63.2 (q, J = 34.3 Hz), 55.9, 54.4, 54.0, 36.0, 34.0, 19.3, 17.8.

^{19}F NMR (471 MHz, $CDCl_3$) δ -73.95.

HRMS: *m/z* calculated for $C_{19}H_{22}F_3N_3O_2^+$: 367.1628 [M+H$^+$]; found: 367.1624.

1-(4-(4-Nitrophenoxy)butan-2-yl)-3-phenyl-3-(trifluoromethyl)diaziridine (3b)

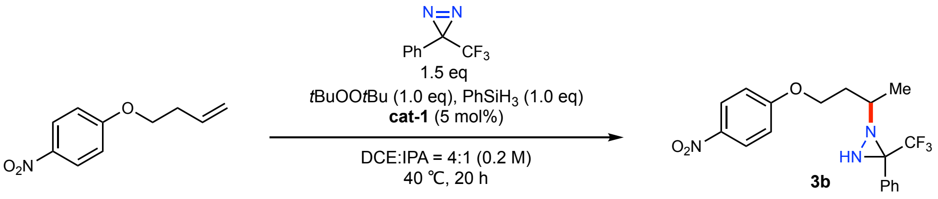

General procedure A was followed on a 0.140 mmol scale. Purification via column chromatography (silica gel, 0 – 30% EA in hexanes) afforded **3b** (42.8 mg, 83 % yield).

Physical state: yellow oil. $\mathbf{R_f}$ = 0.3 (10% ethyl acetate in hexanes, vis. UV and iodine).

^{1}H NMR (500 MHz, $CDCl_3$) δ 8.32 – 8.13 (m, 2H), 7.76 – 7.63 (m, 2H), 7.53 – 7.38 (m, 3H), 6.95 – 6.71 (m, 2H), 4.28 – 3.84 (m, 2H), 3.11 – 2.91 (m, 1H), 2.06 – 1.80 (m, 3H), 1.26 – 0.99 (m, 4H).

^{13}C NMR (126 MHz, $CDCl_3$) δ 164.0, 163.6, 141.4, 130.2, 130.1, 128.4, 128.4, 127.8, 127.0, 125.9, 125.9, 123.6 (q, J = 278.8 Hz), 114.4, 114.2, 65.9, 65.0, 63.9 (q, J = 34.5 Hz), 63.0 (d, J = 34.5 Hz), 54.0, 53.5, 35.5, 33.3, 19.1, 17.7.

^{19}F NMR (471 MHz, $CDCl_3$) δ -73.78.

HRMS: *m/z* calculated for $C_{18}H_{19}F_3N_3O_3^+$: 382.1373 [M+H$^+$]; found:382.1367.

tert-butyl (4-(3-(3-phenyl-3-(trifluoromethyl)diaziridin-1-yl)butoxy)phenyl)carbamate (3c)

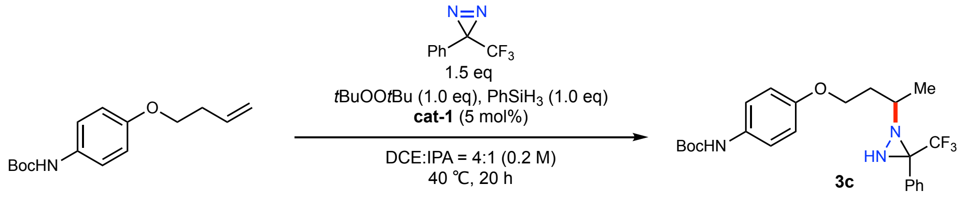

General procedure A was followed on a 0.100 mmol scale. Purification via column chromatography (silica gel, 10 % ethyl acetate in hexanes) afforded 32.5 mg (72% yield).

Physical state: pale yellow oil. $\mathbf{R_f}$ = 0.4 (25% ethyl acetate in hexanes, vis. UV, iodine, and CAM).

^{1}H NMR (500 MHz, $CDCl_3$) δ 7.68 (t, J = 8.0 Hz, 2H), 7.54 – 7.35 (m, 3H), 7.22 (dd, J = 15.5, 8.6 Hz, 2H), 6.83 – 6.57 (m, 2H), 6.47 – 6.24 (m, 1H), 3.99 (qt, J = 9.3, 6.5 Hz, 1H), 3.85 – 3.58 (m, 1H), 3.11 – 2.91 (m, 1H), 2.21 – 1.71 (m, 3H), 1.51 (s, 9H), 1.23 – 0.93 (m, 3H).

^{13}C NMR (126 MHz, $CDCl_3$) δ 155.0, 154.7, 153.2, 131.4, 131.4, 129.9, 130.0, 128.4, 128.3, 128.0, 127.9, 123.8 (d, J = 278.8 Hz), 123.7 (d, J = 278.8 Hz), 120.4, 114.9, 114.6, 80.2, 65.3, 64.0 (q, J = 34.3 Hz), 63.0 (q, J = 34.5 Hz), 54.2, 53.8, 35.8, 33.8, 28.4, 19.2, 17.6.

^{19}F NMR (471 MHz, $CDCl_3$) δ -73.91, -73.93.

HRMS: m/z calculated for $C_{23}H_{29}F_3N_3O_3^+$: 452.2156 [M+H$^+$]; found:452.2156.

***N*-(4-(3-(3-Phenyl-3-(trifluoromethyl)diaziridin-1-yl)butoxy)phenyl)acetamide (3d)**

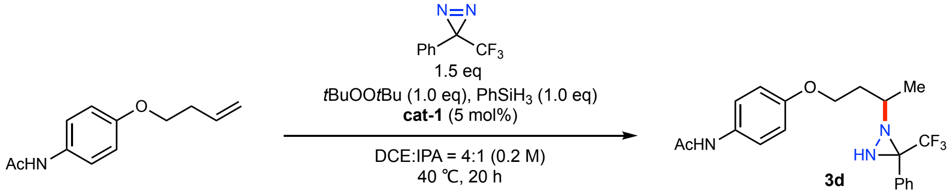

General procedure A was followed on a 0.100 mmol scale. Purification via column chromatography (silica gel, 30% ethyl acetate in hexanes) afforded **3d** (26.9 mg, 68 % yield).

Physical state: pale yellow oil. $\mathbf{R_f}$ = 0.3 (50 % ethyl acetate in hexanes, vis. UV and iodine).

^{1}H NMR (500 MHz, $CDCl_3$) δ 7.67 (t, J = 7.9 Hz, 2H), 7.51 – 7.27 (m, 6H), 6.81 – 6.53 (m, 2H), 4.11 – 3.93 (m, 1H), 3.85 – 3.64 (m, 1H), 2.99 (d, J = 16.6 Hz, 1H), 2.13 (s, 3H), 1.98 – 1.67 (m, 4H), 1.23 – 0.93 (m, 3H).

^{13}C NMR (126 MHz, $CDCl_3$) δ 168.4, 155.9, 155.6, 131.0, 131.0, 130.2, 130.1, 128.5, 128.4, 128.0, 127.9, 123.8 (d, J = 278.8 Hz), 123.8 (q, J = 278.8 Hz), 122.0, 121.9, 114.9, 114.6, 65.4, 64.5, 64.1 (d, J = 34.5 Hz), 63.2 (d, J = 34.5 Hz), 54.3, 53.9, 35.9, 33.8, 24.4, 19.3, 17.7.

^{19}F NMR (471 MHz, $CDCl_3$) δ -73.91, -73.94.

HRMS: *m/z* calculated for $C_{20}H_{23}F_3N_3O_2^+$: 394.1737 [M+H$^+$]; found: 394.1739.

Benzyl (4-(3-(3-phenyl-3-(trifluoromethyl)diaziridin-1-yl)butoxy)phenyl)carbamate (3e)

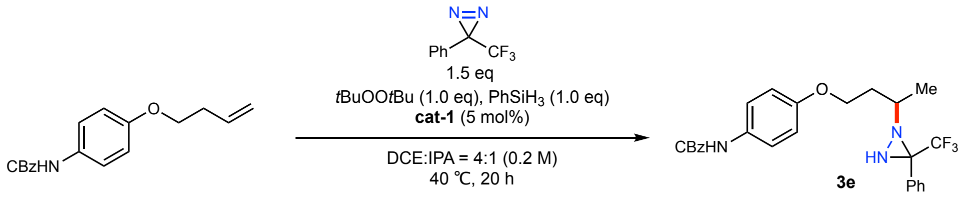

General procedure A was followed on a 0.100 mmol scale. Purification via column chromatography (silica gel, 30% ethyl acetate in hexanes) afforded **3e** (18.1 mg, 80% yield).

Physical state: pale yellow oil. $\mathbf{R_f}$ = 0.7 (30% ethyl acetate in hexanes, vis. UV, iodine and CAM).

^{1}H NMR (500 MHz, $CDCl_3$) δ 7.56 (t, J = 8.1 Hz, 2H), 7.40 – 7.18 (m, 8H), 7.12 (q, J = 9.2 Hz, 2H), 6.71 – 6.62 (m, 1H), 6.50 (dq, J = 10.2, 4.0 Hz, 2H), 5.06 (s, 2H), 3.87 (qt, J = 9.3, 6.6 Hz, 1H), 3.77 – 3.43 (m, 1H), 3.04 – 2.80 (m, 1H), 2.08 – 1.60 (m, 3H), 1.12 – 0.79 (m, 3H).

^{13}C NMR (126 MHz, $CDCl_3$) δ 155.4, 155.2, 153.8, 136.3, 136.3, 130.9, 130.8, 130.1, 130.1, 128.7, 128.6, 128.5, 128.4, 128.0, 128.0, 123.8 (d, J = 278.4 Hz), 123.8 (d, J = 278.8 Hz), 120.7, 115.1, 114.8, 67.0, 65.4, 64.5, 64.1 (q, J = 34.5 Hz), 63.1 (q, J = 34.5 Hz), 54.3, 53.9, 35.9, 33.8, 19.3, 17.7.

^{19}F NMR (471 MHz, $CDCl_3$) δ -73.87, -73.89.

HRMS: Calculated for $C_{26}H_{24}F_3N_3O_2Na^+$ 508.1818 [M+Na^+]; found 508.1826.

4-(3-(3-Phenyl-3-(trifluoromethyl)diaziridin-1-yl)butoxy)aniline (3f)

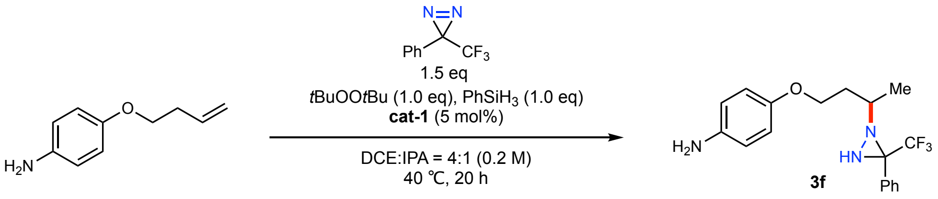

General procedure A was followed on a 0.100 mmol scale. Purification via column chromatography (silica gel, 0 – 5% methanol in DCM) afforded **3f** (18.1 mg, 54 % yield).

Physical state: dark yellow oil. $\mathbf{R_f}$ = 0.5 (3 % methanol in DCM, vis. UV and iodine).

^{1}H NMR (500 MHz, $CDCl_3$) δ 7.68 (ddd, *J* = 10.0, 8.0, 1.5 Hz, 2H), 7.55 – 7.34 (m, 3H), 6.71 – 6.66 (m, 1H), 6.64 – 6.57 (m, 2H), 6.53 (d, *J* = 8.8 Hz, 1H), 4.02 – 3.88 (m, 1H), 3.82 – 3.60 (m, 1H), 3.40 (s, 2H), 2.98 (dd, *J* = 15.2, 2.1 Hz, 1H), 2.20 – 2.07 (m, 1H), 1.98 – 1.80 (m, 2H), 1.75 (dddd, *J* = 14.0, 8.7, 6.7, 5.6 Hz, 1H), 1.21 – 0.92 (m, 3H).

^{13}C NMR (126 MHz, $CDCl_3$) δ 152.2, 151.9, 139.7, 139.6, 130.0, 129.9, 128.4, 126.4, 123.7 (d, *J* = 279.0 Hz), 116.5, 116.4, 115.8, 115.3, 65.7, 64.7, 63.0 (d, *J* = 34.6 Hz), 54.3, 54.0, 35.9, 33.9, 30.0, 29.7, 19.2, 17.6, 14.1.

^{19}F NMR (471 MHz, $CDCl_3$) δ -73.95, -73.99.

HRMS: *m/z* calculated for $C_{18}H_{21}F_3N_3O^+$: 352.1631 [M+H$^+$]; found: 352.1629.

1-(4-(4-methoxyphenoxy)butan-2-yl)-3-(perfluorooctyl)-3-phenyldiaziridine (3g)

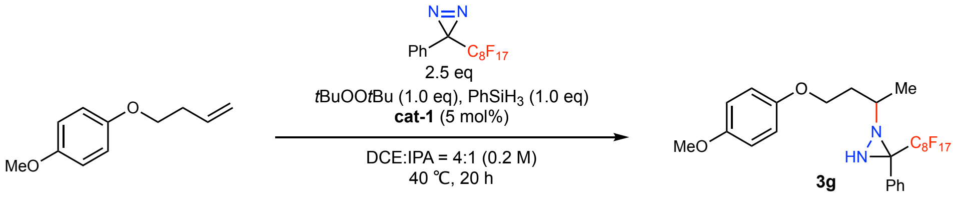

General procedure A was followed on a 0.100 mmol scale. Purification via column chromatography (silica gel, 5% ethyl acetate in hexanes, isocratic) afforded **3g** (66.5 mg, 93% yield).

Physical state: brown solid. $\mathbf{R_f}$ = 0.5 (30% ethyl acetate in hexanes, vis. UV, iodine, and CAM).

^{1}H NMR (500 MHz, $CDCl_3$, major isomer) δ 7.72 – 7.61 (m, 2H, merged with minor isomer), 7.49 – 7.34 (m, 3H, merged with minor isomer), 6.84 – 6.57 (m, 4H, merged with minor isomer), 3.82 (ddd, J = 9.5, 7.0, 5.3 Hz, 1H), 3.76 (s, 3H, merged with minor isomer), 3.71 (ddd, J = 9.3, 7.5, 6.4 Hz, 1H), 2.97 (t, J = 3.1 Hz, 1H), 2.00 – 1.74 (m, 3H, merged with minor isomer), 1.19 (d, J = 6.1 Hz, 3H).

^{1}H NMR (500 MHz, $CDCl_3$, minor isomer) δ 7.72 – 7.61 (m, 2H, merged with major isomer), 7.49 – 7.34 (m, 3H, merged with major isomer), 6.84 – 6.61 (m, 4H, merged with major isomer), 4.02 – 3.92 (m, 2H), 3.76 (s, 3H, merged with major isomer), 2.94 (d, J = 3.4 Hz, 1H), 2.16 – 2.07 (m, 1H), 2.00 – 1.74 (m, 2H, merged with major isomer), 1.03 (d, J = 6.3 Hz, 3H).

^{13}C NMR (126 MHz, $CDCl_3$, mixture of isomers) δ 153.8, 153.7, 153.0, 152.8, 130.6, 130.0, 123.0, 128.2, 128.2, 118.1 (d, J = 33.6 Hz), 115.9, 115.5, 115.1, 114.6, 114.6, 112.8, 110.5 (dd, J = 63.6, 31.8 Hz), 109.5 – 107.8 (m), 65.7, 64.8, 63.8 (t, J = 22.5 Hz), 63.1 (t, J = 22.9 Hz), 55.73, 53.9, 53.5, 35.9, 33.8, 28.4, 19.1, 17.6.

^{19}F NMR (471 MHz, $CDCl_3$, mixture of isomers) δ -80.79 (t, J = 9.5 Hz), -114.32 (q, J = 9.1 Hz), -114.81 – -115.05 (m), -118.22 (ddt, J = 36.4, 24.3, 12.6 Hz), -118.86 (ddt, J = 36.4, 19.1, 12.1 Hz), -119.75 (q, J = 11.3 Hz), -120.27 – -120.51 (m), -120.53 – -120.79 (m), -120.93 – -121.45 (m), -121.53 – -122.22 (m), -122.45 – -123.03 (m), -125.35 (ddt, J = 48.6, 40.7, 13.4 Hz), -125.80 – -126.15 (m), -126.32 (dt, J = 19.1, 10.8 Hz).

HRMS: *m/z* calculated for $C_{26}H_{22}F_{17}N_2O_2^+$: 717.1404 [M+H$^+$]; found: 717.1405.

1-(Heptan-2-yl)-3-phenyl-3-(trifluoromethyl)diaziridine (3h)

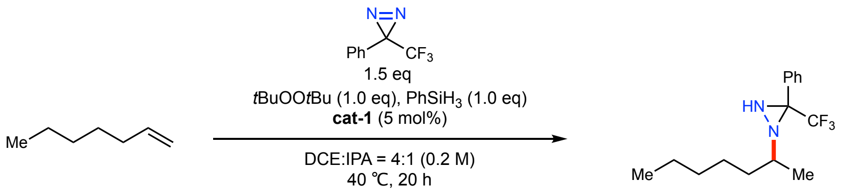

General procedure A was followed on a 10.0 mmol scale. Purification via column chromatography (silica gel, 100% hexanes) afforded **3h** (2.09 g, 73 % yield).

Physical state: pale yellow oil. $\mathbf{R_f}$ = 0.2, (100% hexanes, vis. iodine).

^{1}H NMR: (500 MHz, $CDCl_3$) δ 7.80 – 7.59 (m, 2H), 7.52 – 7.32 (m, 3H), 2.93 (s, 1H), 1.62 (tt, *J* = 8.5, 4.6 Hz, 2H), 1.42 (qt, *J* = 9.3, 4.6 Hz, 1H), 1.33 – 1.09 (m, 6H), 0.89 (d, *J* = 6.3 Hz, 3H), 0.86 (t, *J* = 7.2 Hz, 3H).

^{13}C NMR: (126 MHz, $CDCl_3$) δ 130.0, 129.9, 128.2, 128.2, 123.8 (q, *J* = 278.8 Hz), 63.1 (q, *J* = 34.5 Hz), 56.8, 36.2, 32.0, 29.7, 25.2, 22.6, 17.1, 14.1.

^{19}F NMR: (471 MHz, $CDCl_3$) δ -74.06.

HRMS: *m/z* calculated for $C_{15}H_{22}F_3N_2^+$: 287.1730 [M+H$^+$]; found 287.1736.

1-(Octan-2-yl)-3-phenyl-3-(trifluoromethyl)diaziridine (3i)

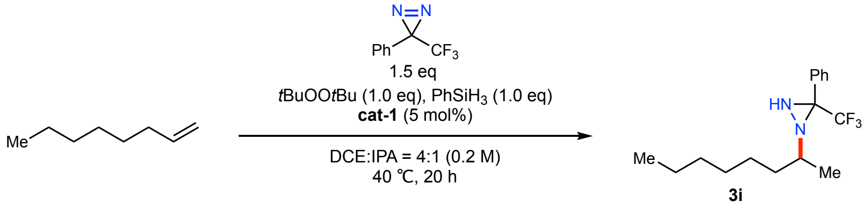

General procedure A was followed on a 0.100 mmol scale. Purification via column chromatography (silica gel, 0 – 6 % ethyl acetate in hexanes) afforded **3i** (22.2 mg, 74 % yield).

Physical state: yellow oil. $\mathbf{R_f}$ = 0.8, 0.7 (5% ethyl acetate in hexanes, vis. UV, iodine and CAM).

^{1}H NMR (500 MHz, $CDCl_3$) δ 7.79 – 7.62 (m, 2H), 7.52 – 7.36 (m, 3H), 2.94 (d, J = 6.1 Hz, 1H), 1.74 – 1.52 (m, 2H), 1.49 – 1.17 (m, 7H), 1.15 – 1.06 (m, 3H), 1.06 – 0.97 (m, 1H), 0.95 – 0.78 (m, 5H).

^{13}C NMR (126 MHz, $CDCl_3$) δ 130.1, 129.9, 128.3, 128.2, 123.9 (q, J = 278.8 Hz), 123.9 (q, J = 278.8 Hz), 120.4, 64.0 (q, J = 34.5 Hz), 63.2 (q, J = 34.3 Hz), 56.8, 56.7, 36.2, 33.9, 31.8, 31.6, 29.5, 29.0, 25.5, 25.2, 22.6, 22.5, 19.0, 17.1, 14.1, 14.0.

^{19}F NMR (471 MHz, $CDCl_3$) δ -74.02, -74.06.

HRMS: Calculated for $C_{16}H_{25}F_3N_2^+$ 301.1886 [$M+H^+$]; found 301.1880.

5-(3-Phenyl-3-(trifluoromethyl)diaziridin-1-yl)hexanenitrile (3j)

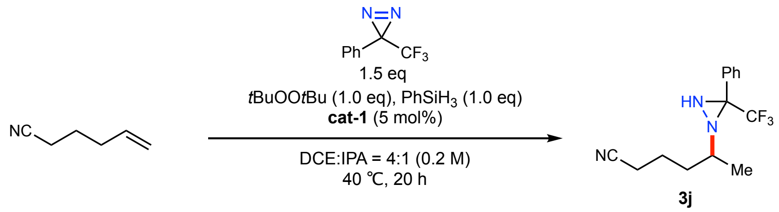

General procedure A was followed on a 0.200 mmol scale. Purification via column chromatography (silica gel, 0 – 20% ethyl acetate in hexanes) afforded **3j** (37.4 mg, 66 % yield).

Physical state: pale yellow oil. $\mathbf{R_f}$ = 0.3 (10 % ethyl acetate in hexanes, vis. iodine and CAM).

^{1}H NMR (500 MHz, $CDCl_3$) δ 7.68 (t, J = 5.9 Hz, 2H), 7.52 – 7.38 (m, 3H), 3.10 – 2.89 (m, 1H), 2.33 (t, J = 6.9 Hz, 1H), 2.11 (td, J = 7.1, 3.3 Hz, 1H), 1.83 – 1.54 (m, 4H), 1.52 – 1.40 (m, 1H), 1.37 – 1.23 (m, 1H), 1.13 (d, J = 6.4 Hz, 3H, major isomer), 0.91 (d, J = 6.3 Hz, 3H, minor isomer).

^{13}C NMR (126 MHz, $CDCl_3$) δ 130.3, 130.2, 129.8, 128.6, 128.5, 128.0, 127.9, 123.7 (d, J = 278.8 Hz), 123.6 (q, J = 279.1 Hz), 63.8 (d, J = 34.5 Hz), 63.0 (d, J = 35.0 Hz), 55.8, 55.7, 35.2, 33.0, 21.7, 21.3, 18.9, 17.4, 17.3.

^{19}F NMR (471 MHz, $CDCl_3$) δ -73.86, -74.10.

HRMS: Calculated for $C_{14}H_{18}F_3N_2^+$ 284.1369 [M+H$^+$]; found 284.1363.

3-phenyl-1-(1-phenylpropan-2-yl)-3-(trifluoromethyl)diaziridine (3k)

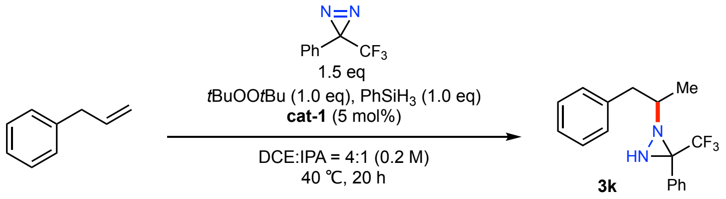

General procedure A was followed on a 0.200 mmol scale. Purification via column chromatography (silica gel, 3 – 5% ethyl acetate in hexanes) afforded **3k** (43.1 mg, 70% yield).

Physical state: pale yellow oil. $\mathbf{R_f}$ = 0.5 (10% ethyl acetate in hexanes, vis. iodine and CAM).

^{1}H NMR (major isomer): (500 MHz, $CDCl_3$) δ 7.70 (dd, *J* = 5.8, 3.6 Hz, 2H, merged with minor isomer), 7.58 – 7.37 (m, 2H, merged with minor isomer), 7.24 – 7.10 (m, 3H, merged with minor isomer), 7.07 (dd, *J* = 7.0, 1.7 Hz, 1H, merged with minor isomer), 6.66 – 6.59 (m, 1H, merged with minor isomer), 3.02 (s, 1H, merged with minor isomer), 2.75 (dd, *J* = 13.2, 3.8 Hz, 1H), 2.48 (dd, *J* = 13.2, 9.7 Hz, 1H), 1.90 (tdd, *J* = 9.5, 6.5, 3.8 Hz, 1H, merged with minor isomer), 1.05 (d, *J* = 6.2 Hz, 3H).

Minor isomer: (500 MHz, $CDCl_3$) δ 7.70 (dd, *J* = 5.8, 3.6 Hz, 2H, merged with major isomer), 7.58 – 7.37 (m, 3H, merged with major isomer), 7.24 – 7.10 (m, 3H, merged with major isomer), 7.09 – 7.03 (m, 1H, merged with major isomer), 6.66 – 6.59 (m, 1H, merged with major isomer), 3.07 (dd, *J* = 13.1, 4.1 Hz, 1H), 3.02 (s, 1H, merged with major isomer), 2.65 (dd, *J* = 13.1, 9.5 Hz, 1H), 1.91 (dtt, *J* = 13.0, 6.5, 3.2 Hz, 1H, merged with major isomer), 0.83 (d, *J* = 6.3 Hz, 3H).

^{13}C NMR: (126 MHz, $CDCl_3$, mixture of diastereomers) δ 138.8, 138.2, 133.0, 130.2, 130.1, 129.7, 129.4, 128.7, 128.5, 128.4, 128.3, 128.3, 128.2, 128.1, 128.1, 126.2, 126.2, 127.4 – 120.4 (m, merged both major and minor), 64.5 – 63.3 (m, merged both major and minor), 59.0, 58.6, 42.7, 40.6, 18.7, 16.7.

^{19}F NMR: (471 MHz, $CDCl_3$) Major isomer: δ -73.68, minor isomer: δ -74.06.

HRMS: Calculated for $C_{17}H_{18}F_3N_2^+$ 307.1417 [M+H$^+$]; found 307.1423.

3-phenyl-1-(4-(phenylthio)butan-2-yl)-3-(trifluoromethyl)diaziridine (3l)

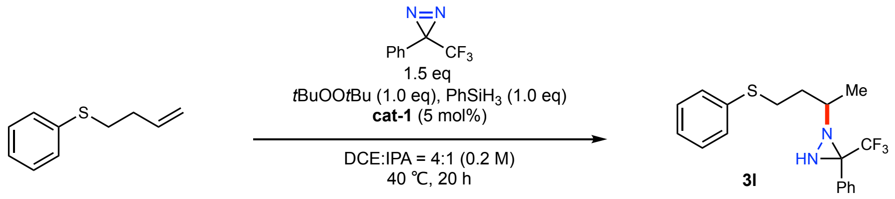

General procedure A was followed on a 0.200 mmol scale. Purification via column chromatography (silica gel, 3 – 5% ethyl acetate in hexanes) afforded **3l** (57.6 mg, 83% yield).

Physical state: pale yellow oil. $\mathbf{R_f}$ = 0.4 (10% ethyl acetate in hexanes, vis. iodine and CAM).

^{1}H NMR (500 MHz, $CDCl_3$) δ 7.74 – 7.52 (m, 2H), 7.51 – 7.39 (m, 2H), 7.38 – 7.26 (m, 4H), 7.25 – 7.14 (m, 2H), 3.05 – 2.76 (m, 2H), 2.04 – 1.62 (m, 3H), 1.19 – 0.85 (m, 3H).

^{13}C NMR (126 MHz, $CDCl_3$) δ 136.7, 136.3, 130.2, 130.1, 129.8, 129.4, 129.4, 129.0, 129.0, 128.9, 128.6, 128.5, 128.0, 126.2, 126.1, 123.8 (d, J = 266.6 Hz), 123.8 (d, J = 266.6 Hz), 55.9, 55.8, 35.9, 33.9, 30.2, 29.4, 18.9, 17.2.

^{19}F NMR (471 MHz, $CDCl_3$) δ -73.99, -74.00.

HRMS: Calculated for $C_{18}H_{20}F_3N_2S^+$ 353.1294 [M+H$^+$]; found 353.1297.

3-Phenyl-1-(4-phenylbutan-2-yl)-3-(trifluoromethyl)diaziridine (3m)

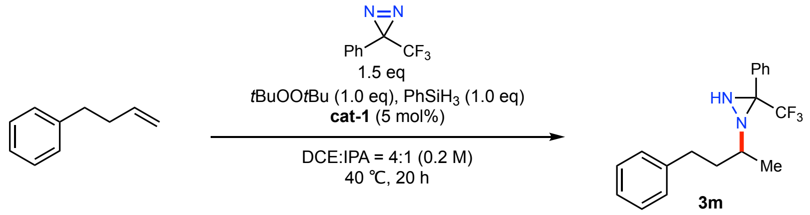

General procedure A was followed on a 0.100 mmol scale. Purification via column chromatography (silica gel, 0 – 10 % ethyl acetate in hexanes) afforded **3m** (24.0 mg, 75% yield).

Physical state: yellow oil. $\mathbf{R_f}$ = 0.6 (10 % ethyl acetate in hexanes, vis. UV, iodine, and CAM).

^{1}H NMR (500 MHz, $CDCl_3$, major isomer) δ 7.59 – 7.46 (m, 2H, merged with minor isomer), 7.34 – 7.22 (m, 3H, merged with minor isomer), 7.17 – 6.97 (m, 4H, merged with minor isomer), 6.88 – 6.74 (m, 1H, merged with minor isomer), 2.92 – 2.82 (m, 1H), 2.46 – 2.28 (m, 1H, merged with minor isomer), 2.04 (ddd, *J* = 13.7, 10.1, 6.7 Hz, 1H), 1.70 – 1.55 (m, 3H), 1.05 (d, *J* = 6.3 Hz, 3H).

^{1}H NMR (500 MHz, $CDCl_3$, minor isomer) δ 7.59 – 7.46 (m, 2H, merged with major isomer), 7.34 – 7.22 (m, 3H, merged with major isomer), 7.17 – 6.97 (m, 4H, merged with major isomer), 6.88 – 6.74 (m, 1H, merged with major isomer), 2.82 – 2.75 (m, 1H), 2.52 (ddd, *J* = 14.0, 11.1, 5.1 Hz, 1H), 2.46 – 2.28 (m, 1H, merged with major isomer), 1.93 – 1.78 (m, 1H), 1.51 (dtd, *J* = 10.4, 7.7, 4.3 Hz, 2H), 0.82 (d, *J* = 6.1 Hz, 3H).

^{13}C NMR (126 MHz, $CDCl_3$) δ 142.3, 141.7, 123.0, 129.9, 128.4, 128.4, 128.3, 128.3, 128.3, 128.3, 128.1, 128.1, 128.1, 125.8, 125.7, 123.8 (d, *J* = 278.8 Hz), 123.7 (d, *J* = 278.8 Hz), 63.8 (q, *J* = 34.5 Hz), 63.0 (q, *J* = 34.3 Hz), 56.4, 56.3, 37.8, 35.6, 31.8, 31.7, 18.9, 17.2.

^{19}F NMR (471 MHz, $CDCl_3$) δ -73.91, -73.94.

HRMS: *m/z* calculated for $C_{18}H_{20}F_3N_2^+$: 321.1573 [M+H$^+$]; found: 321.1574.

***tert*-butyl (6-methoxyquinolin-8-yl)(4-(3-phenyl-3-(trifluoromethyl)diaziridin-1-yl)pentyl)carbamate (3n)**

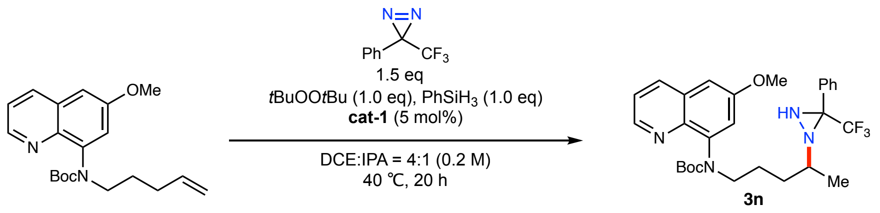

General procedure A was followed on a 0.100 mmol scale. Purification via column chromatography (silica gel, 20 – 50% ethyl acetate in hexanes) afforded **3n** (28.4 mg, 54% yield).

Physical state: pale yellow gum like. $\mathbf{R_f}$ = 0.4 (35% ethyl acetate in hexanes, UV and iodine).

^{1}H NMR (major isomer): (500 MHz, $CDCl_3$) δ 8.76 (dd, *J* = 4.2, 1.7 Hz, 1H, merged with minor), 8.03 (ddd, *J* = 8.3, 4.0, 1.7 Hz, 1H, merged with minor), 7.61 (dd, *J* = 16.4, 7.5 Hz, 2H, merged with minor), 7.47 – 7.29 (m, 4H, merged with minor), 7.23 – 7.08 (m, 1H, merged with minor), 7.00 (t, *J* = 3.3 Hz, 1H, merged with minor), 3.93 (s, 3H), 3.59 (s, br, 2H), 2.90 (s, 1H), 1.79 – 1.56 (m, 3H, merged with minor), 1.50 – 1.14 (m, 11H, merged with minor), 1.06 (d, *J* = 6.3 Hz, 3H).

Minor isomer: (500 MHz, $CDCl_3$) δ 8.76 (dd, *J* = 4.2, 1.7 Hz, 1H, merged with major), 8.03 (ddd, *J* = 8.3, 4.0, 1.7 Hz, 1H, merged with minor), 7.61 (dd, *J* = 16.4, 7.5 Hz, 2H, merged with minor), 7.47 – 7.29 (m, 4H, merged with minor), 7.23 – 7.08 (m, 1H, merged with minor), 7.00 (t, *J* = 3.3 Hz, 1H, merged with minor), 3.93 (s, 3H), 3.59 (s, br, 2H, merged with minor), 1.79 – 1.56 (m, 3H, merged with minor), 1.50 – 1.14 (m, 11H, merged with minor), 0.84 (d, *J* = 6.1 Hz, 3H).

^{13}C NMR: (126 MHz, $CDCl_3$, mixture of diastereomers) δ 157.2, 155.7, 155.6, 147.7, 141.0, 134.9, 130.0, 130.0, 128.4, 128.3, 128.3, 128.2, 123.8 (d, *J* = 266.6 Hz), 121.7, 104.5, 79.8, 63.9 (d, *J* = 34.5 Hz), 56.6, 56.5, 55.7, 50.3, 33.4, 31.6, 28.3, 24.8, 19.2, 17.2.

^{19}F NMR: Major: (471 MHz, $CDCl_3$) δ -74.07. Minor: δ -74.05.

HRMS: Calculated for $C_{28}H_{34}F_3N_4O_3^+$ 531.2578 [M+H$^+$]; found 531.2583.

tert-butyl 4-(1-(3-phenyl-3-(trifluoromethyl)diaziridin-1-yl)ethyl)piperidine-1-carboxylate (3o)

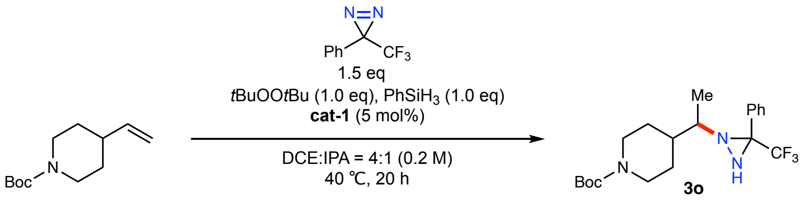

General procedure A was followed on a 0.961 mmol scale. Purification via column chromatography (silica gel, 5 – 10% ethyl acetate in hexanes) afforded **3o** (355 mg, 92% yield).

Physical state: white solid. $\mathbf{R_f}$ = 0.5 (17% ethyl acetate in hexanes, vis. iodine).

^{1}H NMR major isomer (spectrum contaminated with minor isomer): (500 MHz, $CDCl_3$) δ 7.71 – 7.63 (m, 2H), 7.47 – 7.37 (m, 3H), 4.26 – 4.00 (m, 2H), 2.92 (d, J = 1.7 Hz, 1H), 2.64 (s, 2H), 1.79 (dt, J = 12.8, 2.7 Hz, 1H), 1.76 – 1.66 (m, 1H), 1.63 – 1.54 (m, 1H), 1.44 (s, 9H), 1.33 – 1.13 (m, 2H), 0.79 (d, J = 6.6 Hz, 3H).

minor isomer: (500 MHz, $CDCl_3$) δ 7.66 (dd, J = 8.0, 1.8 Hz, 2H), 7.52 – 7.36 (m, 3H), 4.39 – 3.81 (m, 2H), 2.95 (d, J = 1.8 Hz, 1H), 2.74 – 2.40 (m, 2H), 1.83 – 1.71 (m, 1H), 1.69 – 1.60 (m, 1H), 1.43 (s, 9H), 1.24 – 1.12 (m, 1H), 1.07 – 0.92 (m, 5H).

^{13}C NMR major isomer (spectrum contaminated with minor isomer): (126 MHz, $CDCl_3$) δ 155.0, 130.2 (br), 130.1, 128.4, 128.1, 123.9 (q, J = 278.8 Hz), 79.4, 62.8 (d, J = 34.5 Hz), 60.2, 44.2, 41.2, 28.6, 26.7, 13.5.

minor isomer: (126 MHz, $CDCl_3$) δ 154.8, 130.0, 129.8, 128.3, 128.2, 123.7 (q, J = 278.8 Hz), 79.3, 63.8 (q, J = 34.1 Hz), 59.2, 44.1, 40.0, 28.8, 28.5, 26.3, 15.0.

^{19}F NMR major isomer (spectrum contaminated with minor isomer): (471 MHz, $CDCl_3$) δ -73.92.

minor isomer: (471 MHz, $CDCl_3$) δ -73.65.

HRMS: _m/z_ calculated for $C_{20}H_{28}F_3N_3NaO_2^+$: 422.2026 [M+H$^+$]; found: 422.2032. Also found minus _t_Bu form _m/z_ calculated for $C_{16}H_{21}F_3N_3O_2^+$: 344.1580 [M+H$^+$]; found: 344.1577.

4-(1-(3-phenyl-3-(trifluoromethyl)diaziridin-1-yl)ethyl)-1-tosylpiperidine (3p)

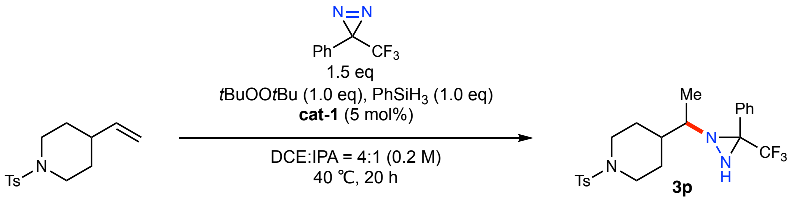

General procedure A was followed on a 1.02 mmol scale. Purification via column chromatography (silica gel, 7 – 12% ethyl acetate in hexanes) afforded **3p** (400 mg, 87% yield).

Physical state: colorless oil. $\mathbf{R_f}$ = 0.6 (17% ethyl acetate in hexanes, vis. iodine and UV).

^{1}H NMR major isomer: (500 MHz, $CDCl_3$) δ 7.68 – 7.55 (m, 4H, merged with minor isomer), 7.49 – 7.29 (m, 5H, merged in minor isomer), 3.91 – 3.66 (m, 2H, merged in minor isomer), 2.93 (s, 1H), 2.45 (s, 3H), 2.24 – 2.11 (m, 2H, merged in minor isomer), 1.66 – 1.52 (m, 2H), 1.52 – 1.34 (m, 1H, merged in minor isomer), 1.28 – 1.15 (m, 2H, merged in minor isomer), 0.99 (d, J = 6.6 Hz, 3H).

minor isomer: (500 MHz, $CDCl_3$) δ 7.70 – 7.55 (m, 4H, merged with major isomer), 7.50 – 7.29 (m, 5H, merged with major isomer), 3.96 – 3.59 (m, 2H, merged with major isomer), 2.89 – 2.73 (s, 1H), 2.43 (s, 3H), 2.28 – 1.98 (m, 2H, merged with major isomer), 1.83 (tt, J = 13.1, 2.7 Hz, 2H), 1.68 – 1.51 (m, 2H, merged with major isomer), 1.51 – 1.33 (m, 1H, merged with major isomer), 1.28 – 1.07 (m, 2H, merged with major isomer), 0.76 (d, J = 6.4 Hz, 3H).

^{13}C NMR mixture of both isomers: (126 MHz, $CDCl_3$) δ 143.6, 143.5, 133.4, 133.3, 130.2, 130.1, 129.8, 129.7, 128.5, 128.4, 128.2, 127.9, 127.8, 123.8 (q, J = 279.1 Hz, major isomer), 123.8 (d, J = 278.8 Hz), 64.0 (q, J = 34.3 Hz, major isomer), 62.8 (q, J = 34.5 Hz), 59.7, 58.8, 46.8, 46.7, 46.7, 46.7, 40.3, 39.3, 28.4, 28.3, 26.0, 25.9, 21.7, 21.6, 15.1, 13.4.

^{19}F NMR major isomer: (471 MHz, $CDCl_3$) δ -73.67.

minor isomer: (471 MHz, $CDCl_3$) δ -73.99.

HRMS: m/z calculated for $C_{22}H_{27}F_3N_3O_2S^+$: 454.1771 [M+H$^+$]; found: 454.1763.

3-methyl-3-(3-phenyl-3-(trifluoromethyl)diaziridin-1-yl)butan-1-ol (3q)

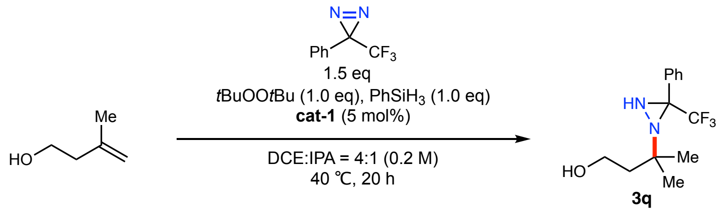

General procedure A was followed on a 0.100 mmol scale. Purification via column chromatography (silica gel, 10% ethyl acetate in hexanes) afforded **3q** (21.1 mg, 77% yield).

Physical state: light yellow oil. $\mathbf{R_f}$ = 0.1 (10% ethyl acetate in hexanes, vis. iodine).

^{1}**H NMR** (500 MHz, $CDCl_3$) δ 7.69 (dt, *J* = 6.6, 1.5 Hz, 2H), 7.52 – 7.35 (m, 3H), 3.95 (ddd, *J* = 11.5, 9.1, 4.0 Hz, 1H), 3.77 (dt, *J* = 11.5, 5.1 Hz, 1H), 2.90 (s, 1H), 1.87 (ddd, *J* = 14.1, 9.1, 4.8 Hz, 1H), 1.47 (ddd, *J* = 14.6, 5.4, 4.0 Hz, 1H), 1.01 (s, 3H), 0.57 (s, 3H).

^{13}**C NMR** (126 MHz, $CDCl_3$) δ 131.3, 130.1, 129.5, 129.2, 128.9, 128.2, 123.6 (d, *J* = 279.9 Hz), 62.6 (d, *J* = 34.5 Hz), 61.0, 59.9, 44.6, 24.5, 23.3.

^{19}**F NMR** (471 MHz, $CDCl_3$) δ -76.23.

HRMS Calculated for $C_{13}H_{18}F_3N_2O^+$ 275.1366 [M+H$^+$]; found 275.1367.

3-methyl-3-(3-phenyl-3-(trifluoromethyl)diaziridin-1-yl)butyl acetate (3r)

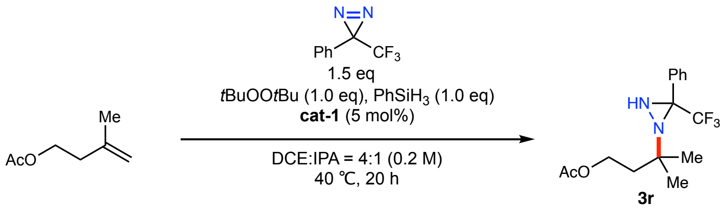

General procedure A was followed on a 0.130 mmol scale. Purification via column chromatography (silica gel, 3% ethyl acetate in hexanes) afforded **3r** (31.3 mg, 76% yield).

Physical state: yellow oil. $\mathbf{R_f}$ = 0.3 (10% ethyl acetate in hexanes, iodine)

^{1}**H NMR** (500 MHz, $CDCl_3$) δ 7.68 (d, J = 6.5 Hz, 2H), 7.41 (ddq, J = 14.7, 7.6, 4.3 Hz, 3H), 4.26 (ddd, J = 8.2, 7.0, 1.7 Hz, 2H), 2.78 (s, 1H), 2.03 (s, 3H), 1.76 (td, J = 7.2, 1.4 Hz, 2H), 0.86 (s, 3H), 0.65 (s, 3H).

^{13}**C NMR** (126 MHz, $CDCl_3$) δ 171.3, 131.3, 129.9, 129.4, 128.8, 128.2, 123.8 (q, J = 279.9 Hz), 62.1 (q, J = 34.2 Hz), 61.4, 58.9, 41.4, 24.3, 21.2.

^{19}**F NMR** (471 MHz, $CDCl_3$) δ -75.79.

HRMS: Calculated for $C_{15}H_{20}F_3N_2O_2^+$ 317.1471 [M+H$^+$]; found 317.1472.

1-(4-(methoxymethoxy)-2-methylbutan-2-yl)-3-phenyl-3-(trifluoromethyl)diaziridine (3s)

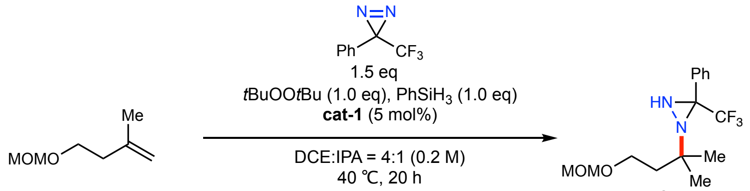

General procedure A was followed on a 0.130 mmol scale. Purification via column chromatography (silica gel, 2% ethyl acetate in hexanes) afforded **3s** (30.5 mg, 74% yield).

Physical state: yellow oil. $\mathbf{R_f}$ = 0.4 (10% ethyl acetate in hexanes, vis. iodine).

^{1}H NMR (500 MHz, $CDCl_3$) δ 7.75 – 7.64 (m, 2H), 7.43 – 7.33 (m, 3H), 4.61 (s, 2H), 3.78 – 3.60 (m, 2H), 3.36 (s, 3H), 2.76 (s, 1H), 1.83 – 1.68 (m, 2H), 0.82 (s, 3H), 0.69 (s, 3H).

^{13}C NMR (126 MHz, $CDCl_3$) δ 131.3, 129.9, 129.8, 129.5, 128.8, 128.1, 123.9 (q, J = 279.7 Hz), 96.6, 64.3, 62.1 (q, J = 34.2 Hz), 59.0, 55.3, 42.3, 24.9, 24.3.

^{19}F NMR (471 MHz, $CDCl_3$) δ -75.75.

HRMS: Calculated for $C_{15}H_{22}F_3N_2O_2^+$ 319.1628 [M+H$^+$]; found 319.1631.

1-(2-methyl-4-((tetrahydro-2*H*-pyran-2-yl)oxy)butan-2-yl)-3-phenyl-3-(trifluoromethyl)diaziridine (3t)

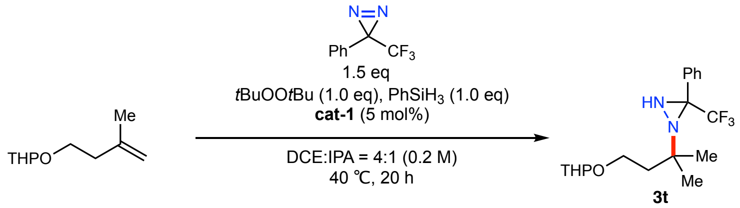

General procedure A was followed on a 0.150 mmol scale. Purification via column chromatography (silica gel, 2% ethyl acetate in hexanes) afforded **3t** (39.0 mg, 71% yield).

Physical state: yellow oil. $\mathbf{R_f}$ = 0.5 (10% ethyl acetate in hexanes, vis. iodine).

^{1}H NMR (500 MHz, $CDCl_3$) δ 7.74 – 7.60 (m, 2H), 7.44 – 7.33 (m, 3H), 4.58 (dd, *J* = 4.7, 2.6 Hz, 1H), 3.99 – 3.77 (m, 2H), 3.65 – 3.42 (m, 2H), 2.74 (s, 1H), 1.86 – 1.66 (m, 4H), 1.59 – 1.46 (m, 4H), 0.82 (d, *J* = 7.8 Hz, 3H), 0.68 (d, *J* = 5.9 Hz, 3H).

^{13}C NMR (126 MHz, $CDCl_3$) δ 131.3, 130.0, 130.0, 129.5, 128.7, 128.1, 123.9 (q, *J* = 279.8 Hz), 99.2, 64.1, 62.6, 62.5, 62.1 (q, *J* = 34.1 Hz), 59.0, 42.4, 31.0, 25.6, 24.8, 24.3, 19.8.

^{19}F NMR (471 MHz, $CDCl_3$) δ -75.72, -75.74.

HRMS: Calculated for $C_{18}H_{26}F_3N_2O_2^+$ 359.1941 [M+H$^+$]; found 359.1943.

2-(3-methyl-3-(3-phenyl-3-(trifluoromethyl)diaziridin-1-yl)butoxy)-1-phenyl-2□^{2}-ethan-1-one (3u)

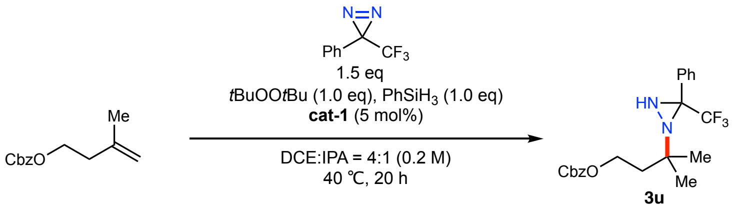

General procedure A was followed on a 0.143 mmol scale. Purification via column chromatography (silica gel, 2 – 5% ethyl acetate in hexanes) afforded **3u** (41.4 mg, 71% yield).

Physical state: pale yellow foam. $\mathbf{R_f}$ = 0.4 (10% ethyl acetate in hexanes, vis. iodine and CAM).

^{1}H NMR: (500 MHz, $CDCl_3$) δ 7.78 – 7.63 (m, 2H), 7.44 – 7.33 (m, 5H), 5.34 – 5.11 (m, 1H), 4.43 – 4.28 (m, 2H), 2.88 – 2.68 (m, 1H), 1.80 (dd, J = 8.2, 6.7 Hz, 2H), 0.89 (d, J = 2.0 Hz, 3H), 0.64 (d, J = 6.0 Hz, 3H).

^{13}C NMR: (126 MHz, $CDCl_3$) δ 155.3, 155.2, 135.4, 131.2, 129.8, 129.6, 129.6, 129.3, 128.8, 128.7, 128.6, 128.5, 128.3, 128.0, 123.7 (d, J = 280.2 Hz), 69.5, 65.0, 64.7, 62.0 (q, J = 34.1 Hz), 58.7, 41.4, 41.3, 24.3, 24.3, 24.2, 24.2.

^{19}F NMR: (471 MHz, $CDCl_3$) δ -75.77.

HRMS: Calculated for $C_{21}H_{22}F_3N_2O_2^+$ 391.1628. [M+H$^+$]; found 391.1635.

1-(4-(benzyloxy)-2-methylbutan-2-yl)-3-phenyl-3-(trifluoromethyl)diaziridine (3v)

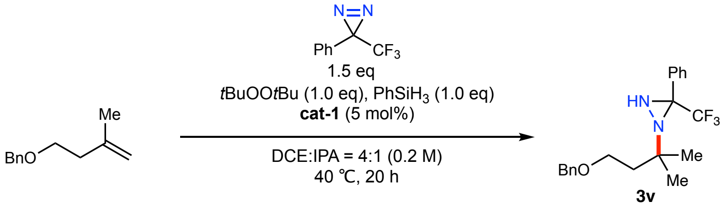

General procedure A was followed on a 0.155 mmol scale. Purification via column chromatography (silica gel, 2% ethyl acetate in hexanes) afforded **3v** (47.2 mg, 84% yield).

Physical state: yellow oil. $\mathbf{R_f}$ = 0.6 (10% ethyl acetate in hexanes, iodine).

^{1}H NMR: (500 MHz, $CDCl_3$) δ 7.68 (t, J = 7.7 Hz, 2H), 7.50 – 7.27 (m, 8H), 4.51 (s, 2H), 3.79 – 3.54 (m, 2H), 2.74 (s, 1H), 1.95 – 1.71 (m, 2H), 0.82 (s, 3H), 0.70 (s, 3H).

^{13}C NMR: (126 MHz, $CDCl_3$) δ 138.6, 131.2, 129.8, 129.7, 129.4, 128.6, 128.4, 128.0, 127.6, 127.5, 123.8 (q, J = 279.9 Hz), 73.0, 66.8, 62.0 (q, J = 34.2 Hz), 59.0, 42.2, 24.9, 24.2.

^{19}F NMR: (471 MHz, $CDCl_3$) δ -75.80.

HRMS: Calculated for $C_{20}H_{24}F_3N_2O^+$ 365.1835 [M+H$^+$]; found 365.1838.

4-(2-(3-phenyl-3-(trifluoromethyl)diaziridin-1-yl)propan-2-yl)-1-tosylpiperidine (3w)

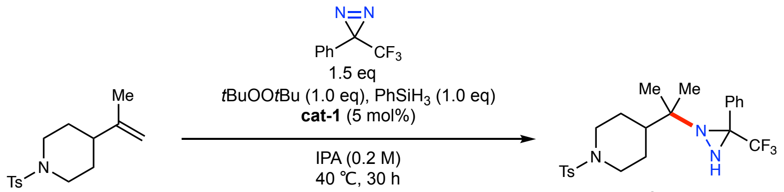

General procedure B was followed on a 0.200 mmol scale. Purification via column chromatography (silica gel, 10 – 20% ethyl acetate in hexanes) afforded **3w** (62.3 mg, 67% yield).

Physical state: pale yellow foam. **R_f** = 0.40 (20% ethyl acetate in hexanes, vis. UV and iodine).

^{1}H NMR: (500 MHz, $CDCl_3$) δ 7.68 – 7.58 (m, 4H), 7.40 – 7.30 (m, 5H), 3.87 (ddq, J = 13.7, 11.4, 2.1 Hz, 2H), 2.68 (d, J = 1.5 Hz, 1H), 2.43 (s, 3H), 2.25 – 2.09 (m, 2H), 1.89 (dt, J = 13.3, 2.7 Hz, 1H), 1.76 (dt, J = 13.1, 2.8 Hz, 1H), 1.58 (qd, J = 11.8, 3.4 Hz, 1H), 1.48 – 1.39 (m, 1H), 1.24 – 1.16 (m, 1H), 0.77 (d, J = 1.7 Hz, 3H), 0.45 (d, J = 1.7 Hz, 3H).

^{13}C NMR: (126 MHz, $CDCl_3$) δ 143.4, 133.2, 131.3, 129.7, 129.7, 129.6, 129.6, 129.4, 128.6, 128.1, 128.1, 127.9, 127.8, 127.8, 123.7 (d, J = 279.7 Hz), 61.0 (q, J = 34.1 Hz), 60.9, 47.4, 47.0, 46.9, 26.3, 25.9, 22.1, 21.5, 20.6.

^{19}F NMR: (471 MHz, $CDCl_3$) δ -75.57.

HRMS: Calculated for $C_{23}H_{29}F_3N_3O_2S^+$ 468.1927 [M+H$^+$]; found 468.1931.

4-(2-(3-(perfluorooctyl)-3-phenyldiaziridin-1-yl)propan-2-yl)-1-tosylpiperidine (3x)

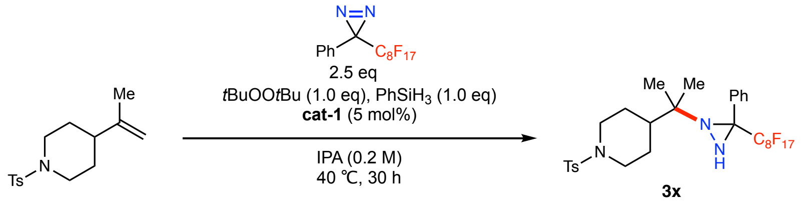

General procedure B was followed on a 0.100 mmol scale. Purification via column chromatography (silica gel, 0 – 30% ethyl acetate in hexanes) afforded **3x** (61.1 mg, 75% yield).

Physical state: brown oil. **R_f** = 0.5 (15% ethyl acetate in hexanes, vis. UV, iodine and CAM).

^{1}H NMR (500 MHz, $CDCl_3$) δ 7.68 – 7.58 (m, 4H), 7.41 – 7.31 (m, 5H), 3.98 – 3.82 (m, 2H), 2.65 (t, *J* = 3.0 Hz, 1H), 2.44 (s, 3H), 2.26 – 2.11 (m, 2H), 1.95 – 1.87 (m, 1H), 1.77 (dt, *J* = 13.3, 2.9 Hz, 1H), 1.66 – 1.58 (m, 1H), 1.45 (td, *J* = 12.7, 4.1 Hz, 1H), 1.20 (tt, *J* = 12.2, 3.3 Hz, 1H), 0.79 (s, 3H), 0.40 (s, 3H).

^{13}C NMR (126 MHz, $CDCl_3$) δ 143.4, 133.3, 131.5, 130.4, 129.8, 129.6, 129.5, 128.6, 128.1, 127.8, 127.8, 127.6, 60.9, 47.6, 47.0, 46.9, 26.3, 26.0, 22.0, 21.5, 20.7.

^{19}F NMR (471 MHz, $CDCl_3$) δ -80.75 (t, *J* = 10.0 Hz), -111.36 – -111.55 (m), -114.35 (q, *J* = 13.4 Hz), -114.81 – -115.07 (m), -117.29 – -117.50 (m), -118.03 (dt, *J* = 21.7, 13.9 Hz), -120.18 (dd, *J* = 17.3, 10.4 Hz), -120.71 – -120.94 (m), -121.18 – -121.48 (m), -121.52 – -122.35 (m), -122.39 – -123.07 (m), -124.91 (dt, *J* = 26.9, 13.9 Hz), -125.39 – -125.61 (m), -125.77 – -126.17 (m), -126.31 (dd, *J* = 17.3, 10.4 Hz).

HRMS: *m/z* calculated for $C_{30}H_{29}F_{17}N_2O_2S^+$: 818.1704 [M+H$^+$]; found: 818.1709.

1-(2-Methyl-4-phenylbutan-2-yl)-3-phenyl-3-(trifluoromethyl)diaziridine (3y)

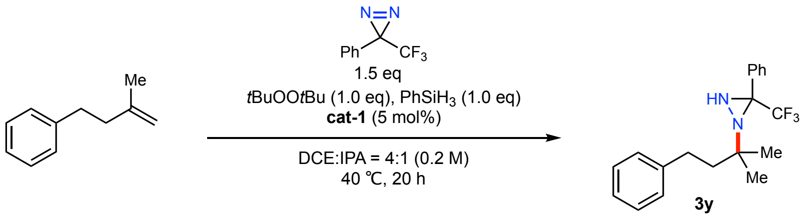

General procedure A was followed on a 0.100 mmol scale. Purification via column chromatography (silica gel, 0 – 5% ethyl acetate in hexanes) afforded **3y** (28.4 mg, 85% yield).

Physical state: pale yellow oil. $\mathbf{R_f}$ = 0.6 (10 % ethyl acetate in hexanes, vis. UV, iodine and CAM).

^{1}H NMR (500 MHz, $CDCl_3$) δ 7.75 – 7.64 (m, 2H), 7.46 – 7.36 (m, 3H), 7.32 – 7.27 (m, 2H), 7.19 (ddt, J = 7.3, 3.5, 1.5 Hz, 3H), 2.85 – 2.64 (m, 3H), 1.84 – 1.66 (m, 2H), 0.82 (s, 3H), 0.74 (s, 3H).

^{13}C NMR (126 MHz, $CDCl_3$) δ 143.1, 131.4, 130.1, 129.8, 129.5, 128.8, 128.5, 128.5, 128.1, 125.8, 124.0 (q, J = 279.7 Hz), 62.0 (q, J = 34.1 Hz), 59.7, 45.1, 30.6, 24.6, 23.9.

^{19}F NMR (471 MHz, $CDCl_3$) δ -75.62.

HRMS: Calculated for $C_{19}H_{22}F_3N_2^+$ 335.1730 [M+H$^+$]; found 335.1727.

1-(2-methyladamantan-2-yl)-3-phenyl-3-(trifluoromethyl)diaziridine (3z)

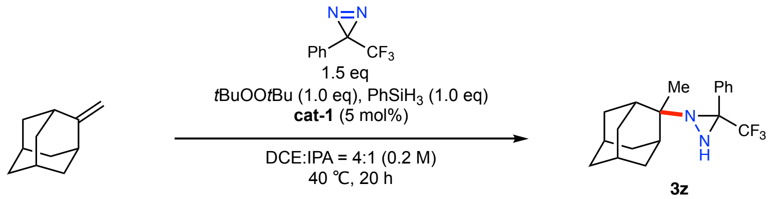

3z

General procedure A was followed on a 0.100 mmol scale. Purification via column chromatography (silica gel, 0 – 1% ethyl acetate in hexanes) afforded **3z** (20.1 mg, 60% yield).

Physical state: pale yellow oil. $\mathbf{R_f}$ = 0.4 (100% hexanes, vis. iodine).

^{1}H NMR: (500 MHz, $CDCl_3$) δ 7.81 – 7.49 (m, 2H), 7.49 – 7.28 (m, 3H), 2.77 – 2.71 (m, 1H), 2.69 (d, J = 14.0 Hz, 2H), 1.91 – 1.83 (m, 2H), 1.78 – 1.69 (m, 4H), 1.66 – 1.54 (m, 5H), 1.51 (dd, J = 12.1, 2.6 Hz, 1H), 0.38 (s, 3H).

^{13}C NMR: (126 MHz, $CDCl_3$) δ 131.2, 130.4, 129.4, 128.5, 124.1 (d, J = 280.3 Hz), 61.6 (q, J = 34.0 Hz), 39.2, 38.1, 36.4, 34.1, 33.8, 32.8, 32.6, 29.7, 27.5, 27.5, 17.5.

^{19}F NMR: (471 MHz, $CDCl_3$) δ -75.50.

HRMS: Calculated for $C_{19}H_{24}F_3N_2^+$ 337.1886 [M+H$^+$]; found 337.1895.

***tert*-Butyl 4-methyl-4-(3-phenyl-3-(trifluoromethyl)diaziridin-1-yl)piperidine-1-carboxylate (3aa)**

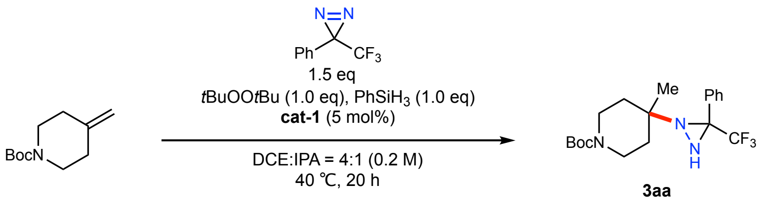

General procedure A was followed on a 0.100 mmol scale. Purification via column chromatography (silica gel, 0 – 15% ethyl acetate in hexanes) afforded **3aa** (32.7 mg, 85% yield).

Physical state: yellow oil. $\mathbf{R_f}$ = 0.4 (10% ethyl acetate in hexanes, vis. iodine and CAM).

^{1}H NMR (500 MHz, $CDCl_3$) δ 7.76 – 7.57 (m, 2H), 7.46 – 7.33 (m, 3H), 3.64 (ddd, J = 12.7, 8.2, 3.9 Hz, 1H), 3.56 (ddd, J = 12.5, 8.1, 4.0 Hz, 1H), 3.42 – 3.29 (m, 1H), 3.22 (ddd, J = 13.4, 7.5, 4.0 Hz, 1H), 2.76 (d, J = 1.7 Hz, 1H), 1.79 – 1.64 (m, 1H), 1.60 (ddd, J = 12.2, 7.6, 4.0 Hz, 1H), 1.44 (s, 10H), 0.94 – 0.82 (m, 1H), 0.56 (s, 3H).

^{13}C NMR (126 MHz, $CDCl_3$) δ 154.8, 131.1, 129.8, 129.8, 129.2, 128.8, 128.1, 123.7 (q, J = 280.0 Hz), 79.3, 62.1 (q, J = 34.5 Hz), 57.3, 40.3, 39.4, 36.3, 28.5, 21.2.

^{19}F NMR (471 MHz, $CDCl_3$) δ -75.73.

HRMS: *m/z* calculated for $C_{19}H_{26}F_3N_3NaO_2^+$: 408.1869 [M+Na$^+$]; found 408.1865.

1-((2*S*,5*R*)-2-isopropyl-1,5-dimethylcyclohexyl)-3-phenyl-3-(trifluoromethyl)diaziridine (3ab)

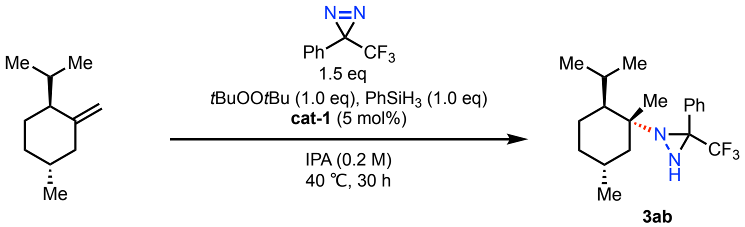

General procedure B was followed on a 0.200 mmol scale. Purification via column chromatography (silica gel, 0 – 5% ethyl acetate in hexanes) afforded **3ab** (47.8 mg, 71% yield, over 20:1 dr).

Physical state: pale yellow oil. $\mathbf{R_f}$ = 0.3 (5% ethyl acetate in hexanes, vis. iodine).

^{1}H NMR: (500 MHz, $CDCl_3$) δ 7.79 – 7.60 (m, 2H), 7.43 – 7.30 (m, 3H), 2.84 – 2.44 (m, 2H), 1.53 (ddq, J = 16.5, 10.1, 3.4 Hz, 2H), 1.33 – 1.21 (m, 2H), 1.16 – 1.05 (m, 2H), 1.03 (s, 3H), 0.94 (d, J = 7.0 Hz, 3H), 0.79 (d, J = 7.0 Hz, 3H), 0.70 (qd, J = 13.0, 4.0 Hz, 1H), 0.50 (d, J = 6.4 Hz, 3H), 0.09 (ddd, J = 12.8, 3.6, 1.9 Hz, 1H).

^{13}C NMR: (126 MHz, $CDCl_3$) δ 131.0, 130.7, 129.5, 129.4, 129.3, 128.4, 127.8, 127.3, 123.9 (q, J = 280.2 Hz), 120.6, 63.3, 62.7, 62.3 (q, J = 34.1 Hz), 61.9, 50.9, 43.2, 34.7, 29.2, 24.9, 24.6, 22.7, 22.6, 18.7, 18.3.

^{19}F NMR: (471 MHz, $CDCl_3$) δ -75.82, -75.87.

HRMS: *m/z* calculated for $C_{19}H_{28}F_3N_2^+$: 341.2199 [M+H$^+$]; found: 341.2193.

1-(1,3,3,5,5-pentamethylcyclohexyl)-3-phenyl-3-(trifluoromethyl)diaziridine (3ac)

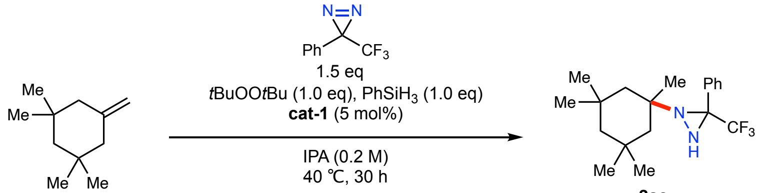

3ac

General procedure B was followed on a 0.130 mmol scale. Purification via column chromatography (silica gel, 0 – 3% ethyl acetate in hexanes) afforded **3ac** (26.4 mg, 59% yield).

Physical state: pale yellow oil. $\mathbf{R_f}$ = 0.3 (100% hexanes, vis. iodine).

^{1}H NMR: (500 MHz, $CDCl_3$) δ 7.67 (t, *J* = 7.5 Hz, 2H), 7.48 – 7.32 (m, 3H), 2.58 (s, 1H), 1.68 (ddd, *J* = 14.7, 9.6, 2.2 Hz, 2H), 1.45 – 1.36 (m, 1H), 1.31 (d, *J* = 2.5 Hz, 3H), 1.23 (d, *J* = 2.5 Hz, 3H), 1.07 (ddd, *J* = 14.7, 7.7, 2.4 Hz, 2H), 0.92 (d, *J* = 2.4 Hz, 3H), 0.87 (d, *J* = 2.5 Hz, 3H), 0.80 (dd, *J* = 14.2, 2.5 Hz, 1H), 0.18 (d, *J* = 2.5 Hz, 3H).

^{13}C NMR: (126 MHz, $CDCl_3$) δ 131.2, 130.5, 129.5, 128.6, 127.9, 123.9 (d, *J* = 280.3 Hz), 63.5 (d, *J* = 34.1 Hz), 60.9, 51.8, 51.6, 48.5, 36.3, 36.1, 31.5, 31.4, 29.8, 28.8, 25.1.

^{19}F NMR: (471 MHz, $CDCl_3$) δ -75.81.

HRMS: Calculated for $C_{19}H_{28}F_3N_2^+$ 341.2199 [M+H$^+$]; found 341.2206.

3-methyl-3-(3-phenyl-3-(trifluoromethyl)diaziridin-1-yl)butan-1-ol (4a)

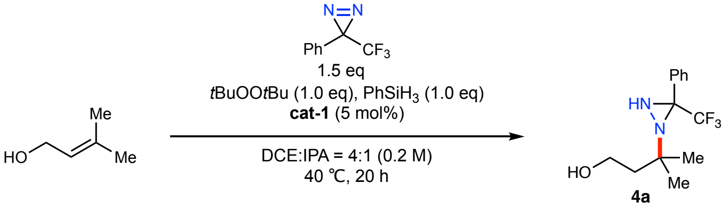

General procedure A was followed on a 0.100 mmol scale. Purification via column chromatography (silica gel, 10% ethyl acetate in hexanes) afforded **4a** (17.5 mg, 64% yield).

Physical state: light yellow oil. $\mathbf{R_f}$ = 0.1 (10% ethyl acetate in hexanes, vis. iodine).

^{1}**H NMR** (500 MHz, $CDCl_3$) δ 7.75 – 7.65 (m, 2H), 7.49 – 7.34 (m, 3H), 3.95 (ddd, J = 11.4, 8.9, 4.2 Hz, 1H), 3.78 (dt, J = 11.1, 5.2 Hz, 1H), 3.53 – 3.31 (m, 1H), 2.89 (s, 1H), 1.87 (ddd, J = 14.0, 8.9, 4.9 Hz, 1H), 1.48 (ddd, J = 14.5, 5.5, 4.1 Hz, 1H), 1.00 (s, 3H), 0.58 (s, 3H).

^{13}**C NMR** (126 MHz, $CDCl_3$) δ 131.3, 130.1, 129.5, 129.2, 128.9, 128.2, 123.6 (q, J = 280.0 Hz), 62.6 (q, J = 34.1 Hz), 61.0, 59.9, 44.6, 24.5, 23.4.

^{19}**F NMR** (471 MHz, $CDCl_3$) δ -76.22.

HRMS Calculated for $C_{13}H_{18}F_3N_2O^+$ 275.1366 [M+H$^+$]; found 275.1361.

6-methyl-6-(3-phenyl-3-(trifluoromethyl)diaziridin-1-yl)heptan-2-one (4b)

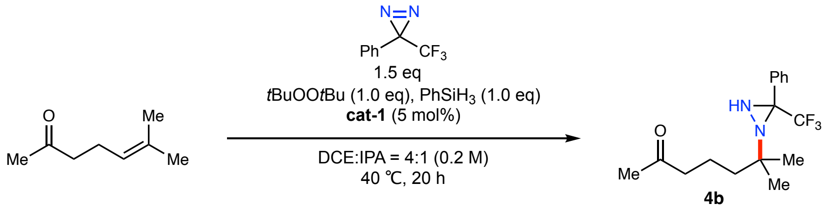

General procedure B was followed on a 0.100 mmol scale. Purification via column chromatography (silica gel, 5 – 10% ethyl acetate in hexanes) afforded **4b** (23.0 mg, 73% yield).

Physical state: pale yellow oil. $\mathbf{R_f}$ = 0.4 (20% ethyl acetate in hexanes, iodine).

^{1}H NMR: (500 MHz, $CDCl_3$) δ 7.67 (t, *J* = 7.2 Hz, 2H), 7.52 – 7.31 (m, 3H), 2.74 (s, 1H), 2.41 (t, *J* = 7.3 Hz, 2H), 2.13 (s, 3H), 1.73 – 1.60 (m, 2H), 1.45 – 1.29 (m, 2H), 0.79 (s, 3H), 0.63 (s, 3H).

^{13}C NMR: (126 MHz, $CDCl_3$) δ 209.1, 131.4, 130.1, 129.8, 129.5, 128.7, 128.1, 123.9 (d, *J* = 279.9 Hz), 61.9 (d, *J* = 34.1 Hz), 59.7, 44.3, 42.5, 30.0, 24.2, 23.9, 18.6.

^{19}F NMR: (471 MHz, $CDCl_3$) δ -75.71.

HRMS: Calculated for $C_{16}H_{22}F_3N_2O^+$ 315.1679 [M+H$^+$]; found 315.1687.

1-(1-methylcyclohexyl)-3-phenyl-3-(trifluoromethyl)diaziridine (4c)

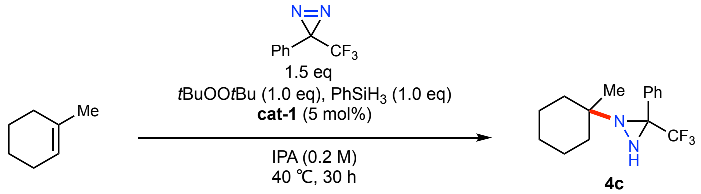

General procedure B was followed on a 0.100 mmol scale. Purification via column chromatography (silica gel, 2% ethyl acetate in hexanes) afforded **4c** (19.9 mg, 71% yield).

Physical state: yellow oil. $\mathbf{R_f}$ = 0.6 (10% ethyl acetate in hexanes, vis. iodine).

^{1}H NMR (500 MHz, CDCl3) δ 7.70 – 7.63 (m, 2H), 7.41 – 7.34 (m, 3H), 2.69 (s, 1H), 1.71 (ddt, *J* = 12.7, 9.7, 5.2 Hz, 1H), 1.65 – 1.57 (m, 2H), 1.52 – 1.28 (m, 6H), 0.92 – 0.86 (m, 1H), 0.54 (s, 3H).

^{13}C NMR (126 MHz, $CDCl_3$) δ 131.2, 130.5, 129.6, 129.5, 128.7, 128.0, 124.0 (d, *J* = 280.0 Hz), 62.2 (d, *J* = 34.0 Hz), 59.3, 37.2, 37.1, 29.8, 25.9, 22.4, 21.2.

^{19}F NMR (471 MHz, $CDCl_3$) δ -75.76.

HRMS: Calculated for $C_{15}H_{20}F_3N_2^+$ 285.1573 [M+H$^+$]; found 285.1582.

1-(1-methylcyclohexyl)-3-(perfluorooctyl)-3-phenyldiaziridine (4d)

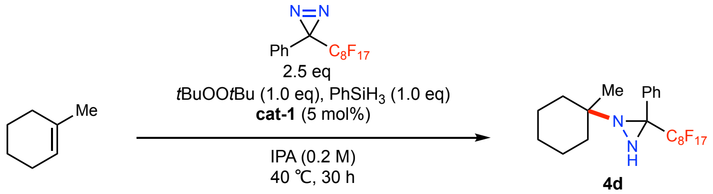

General procedure B was followed on a 0.100 mmol scale. Purification via column chromatography (silica gel, 0 – 3% ethyl acetate in hexanes) afforded **4d** (15.1 mg, 42% yield).

Physical state: yellow amorphous solid. $\mathbf{R_f}$ = 0.7 (15% ethyl acetate in hexanes, vis. UV, iodine, and CAM).

^{1}H NMR: (600 MHz, $CDCl_3$) δ 7.60 (t, J = 9.6 Hz, 2H), 7.35 – 7.26 (m, 3H), 2.60 (s, 1H), 1.65 (q, J = 8.2 Hz, 1H), 1.55 (tt, J = 8.6, 4.2 Hz, 2H), 1.46 – 1.34 (m, 2H), 1.33 – 1.25 (m, 3H), 1.27 – 1.22 (m, 2H), 0.93 – 0.78 (m, 1H), 0.42 (s, 3H).

^{13}C NMR: (151 MHz, $CDCl_3$) δ 131.3, 130.4, 130.2, 129.6, 128.5, 127.5, 62.1, 59.3, 37.4, 36.8, 36.5, 25.8, 22.2, 22.2, 21.0.

^{19}F NMR: (471 MHz, $CDCl_3$) δ -80.59 – -80.97 (m), -114.36 (t, J = 14.5 Hz), -114.94 (d, J = 14.7 Hz), -117.25 – -117.54 (m), -117.86 – -118.20 (m), -120.18 (dd, J = 17.8, 10.0 Hz), -120.82 (p, J = 13.1, 10.4 Hz), -121.14 – -122.27 (m), -122.36 – -123.12 (m), -124.90 (d, J = 19.6 Hz), -125.20 – -125.64 (m), -125.74 – -126.48 (m), -126.89 – -127.03 (m).

HRMS: m/z calculated for $C_{22}H_{19}F_{17}N_2^+$: 635.1350 [M+H$^+$]; found: 635.1358.

1-(4-((*tert*-butyldimethylsilyl)oxy)-2-methylbutan-2-yl)-3-phenyl-3-(trifluoromethyl)diaziridine (4e)

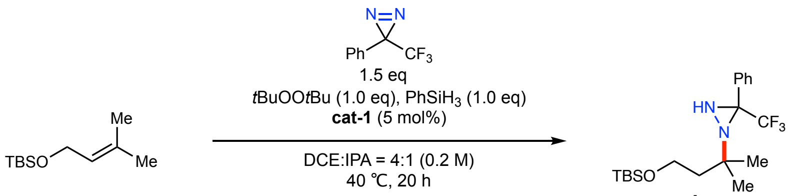

General procedure A was followed on a 0.110 mmol scale. Purification via column chromatography (silica gel, 1% ethyl acetate in hexanes) afforded **4e** (38.0 mg, 89% yield).

Physical state: yellow oil. $\mathbf{R_f}$ = 0.1 (100% hexanes, vis. iodine).

^{1}**H NMR** (500 MHz, $CDCl_3$) δ 7.68 (d, J = 7.4 Hz, 2H), 7.49 – 7.33 (m, 3H), 3.86 – 3.70 (m, 2H), 2.72 (d, J = 2.2 Hz, 1H), 1.68 (t, J = 7.5 Hz, 2H), 0.89 (s, 9H), 0.78 (s, 3H), 0.70 (s, 3H), 0.05 (s, 6H).

^{13}**C NMR** (126 MHz, $CDCl_3$) δ 131.3, 130.0, 129.8, 129.5, 128.8, 128.1, 123.9 (q, J = 279.7 Hz), 62.2 (q, J = 34.1 Hz), 59.7, 59.2, 45.3, 26.1, 25.1, 24.2, 18.4, -5.1, -5.2.

^{19}**F NMR** (471 MHz, $CDCl_3$) δ -75.78.

HRMS Calculated for $C_{19}H_{32}F_3N_2OSi^+$ 389.2231 [M+H$^+$]; found 389.2241.

4-Methoxybenzyl 3,7-dimethyl-7-(3-phenyl-3-(trifluoromethyl)diaziridin-1-yl)octanoate (4f)

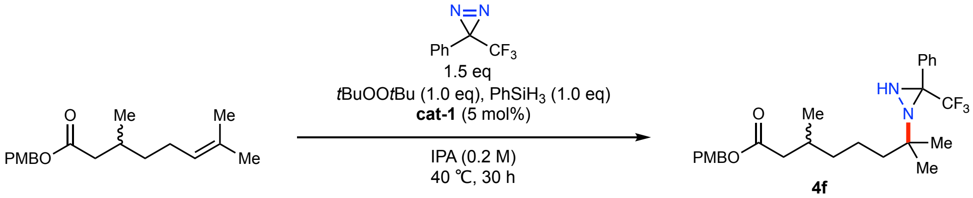

General procedure B was followed on a 0.200 mmol scale. Purification via column chromatography (silica gel, 0 – 20% ethyl acetate in hexanes) afforded **4f** (61.9 mg, 65% yield).

Physical state: pale yellow oil. $\mathbf{R_f}$ = 0.4 (20 % ethyl acetate in hexanes, vis. UV, iodine and CAM).

^{1}**H NMR** (500 MHz, $CDCl_3$) δ 7.73 – 7.61 (m, 2H), 7.44 – 7.34 (m, 3H), 7.33 – 7.27 (m, 2H), 6.94 – 6.81 (m, 2H), 5.06 (s, 2H), 3.80 (s, 3H), 2.81 – 2.63 (m, 1H), 2.33 (ddd, J = 14.6, 6.1, 2.2 Hz, 1H), 2.14 (ddd, J = 14.6, 8.1, 5.3 Hz, 1H), 2.03 – 1.90 (m, 1H), 1.44 – 1.20 (m, 5H), 1.21 – 1.08 (m, 1H), 0.92 (d, J = 6.7 Hz, 3H), 0.74 (d, J = 6.7 Hz, 3H), 0.63 (d, J = 5.8 Hz, 3H).

^{13}**C NMR** (126 MHz, $CDCl_3$) δ 173.3, 173.3, 159.7, 131.4, 130.2, 130.2, 130.2, 130.1, 129.7, 129.5, 128.7, 128.4, 128.0, 124.0 (q, J = 280.0 Hz), 114.0, 66.0, 66.0, 61.9 (q, J = 34.3 Hz), 59.7, 55.4, 43.1, 43.1, 42.1, 42.0, 37.4, 37.4, 30.4, 30.4, 24.5, 24.4, 23.9, 21.3, 21.3, 19.9, 19.8.

^{19}**F NMR** (471 MHz, $CDCl_3$) δ -75.65.

HRMS: Calculated for $C_{26}H_{34}F_3N_2O_3^+$ 479.2522. [M+H$^+$]; found 479.2525.

Methyl 3,7-dimethyl-7-(3-phenyl-3-(trifluoromethyl)diaziridin-1-yl)octanoate (4g)

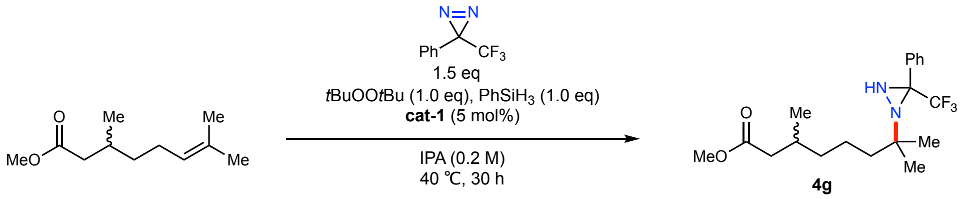

General procedure B was followed on a 0.100 mmol scale. Purification via column chromatography (silica gel, 5% ethyl acetate in hexanes) afforded **4g** (21.2 mg, 57% yield).

Physical state: colorless oil. $\mathbf{R_f}$ = 0.5 (10 % ethyl acetate in hexanes, vis. iodine and CAM).

^{1}H NMR (500 MHz, $CDCl_3$) δ 7.67 (dd, J = 14.8, 7.6 Hz, 2H), 7.39 (tq, J = 7.8, 3.6 Hz, 3H), 3.67 (s, 3H), 2.82 – 2.65 (m, 1H), 2.31 (ddd, J = 14.6, 6.0, 1.6 Hz, 1H), 2.12 (ddd, J = 14.6, 8.2, 4.1 Hz, 1H), 2.04 – 1.88 (m, 1H), 1.46 – 1.22 (m, 5H), 1.16 (ddd, J = 15.1, 10.8, 7.1 Hz, 1H), 0.93 (d, J = 6.7 Hz, 3H), 0.74 (d, J = 6.3 Hz, 3H), 0.64 (d, J = 4.7 Hz, 3H).

^{13}C NMR (126 MHz, $CDCl_3$) δ 173.9, 173.9, 131.4, 130.2, 130.1, 129.7, 129.5, 128.7, 128.0, 124.0 (q, J = 279.7 Hz), 61.9 (q, J = 34.1 Hz), 59.8, 51.5, 43.1, 43.0, 41.8, 41.7, 37.5, 30.4, 30.4, 24.5, 24.4, 23.9, 21.3, 19.9, 19.8.

^{19}F NMR (471 MHz, $CDCl_3$) δ -75.69.

HRMS: Calculated for $C_{19}H_{28}F_3N_2O_2^+$ 373.2097 $[M^+H^+]$; found 373.2096.

tert-butyl 4-(3-phenyl-3-(trifluoromethyl)diaziridin-1-yl)azepane-1-carboxylate (5a)

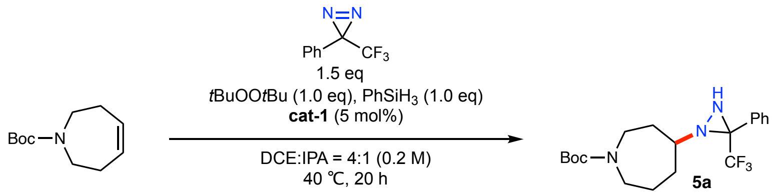

General procedure A was followed on a 0.450 mmol scale. Purification via column chromatography (silica gel, 5% to 10% ethyl acetate in hexanes) afforded **5a** (75.7 mg, 44% yield).

Physical state: pale yellow foam. **R_f** = 0.3 (10% ethyl acetate in hexanes, vis. iodine).

^{1}H NMR (500 MHz, $CDCl_3$) δ 7.65 (d, J = 7.3 Hz, 2H), 7.53 – 7.32 (m, 3H), 3.55 – 2.87 (m, 5H), 2.06 – 1.55 (m, 6H), 1.53 – 1.44 (m, 1H, major isomer), 1.41 – 1.30 (m, 9H), 1.00 (dddt, J = 12.8, 9.2, 6.0, 3.1 Hz, 1H, minor isomer).

^{13}C NMR (126 MHz, $CDCl_3$, mixture of diastereomers) δ 155.2, 155.2, 130.1, 129.9, 128.4, 128.0, 127.8, 123.6 (q, J = 278.4 Hz), 79.1, 63.9 (d, J = 38.1 Hz), 61.0, 60.9, 60.8, 60.6, 46.8, 46.6, 46.2, 45.7, 44.2, 43.6, 43.0, 42.5, 34.2, 33.8, 32.5, 32.4 31.8, 31.7, 30.6, 30.2, 28.4, 28.4, 28.4, 24.9, 24.7, 24.1, 23.7.

^{19}F NMR (471 MHz, $CDCl_3$) δ -74.03, -74.09, -74.19.

HRMS: Calculated for $C_{19}H_{27}F_3N_3O_2^+$ 386.2050. [M+H$^+$]; found 386.2058.

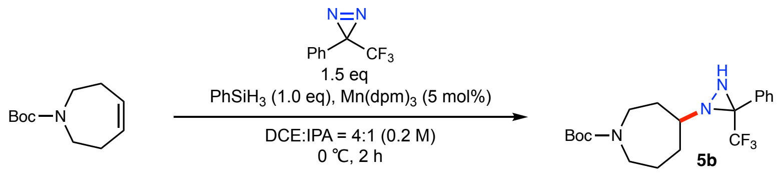

General procedure C was followed on a 0.270 mmol scale. Purification via column chromatography (silica gel, 5% to 10% ethyl acetate in hexanes) afforded **5b** (89.3 mg, 85% yield).

Characterization data matched with **5a**.

1-(Bicyclo[2.2.1]heptan-2-yl)-3-phenyl-3-(trifluoromethyl)diaziridine (5c)

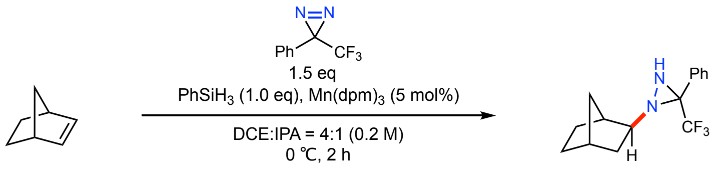

5c

General procedure A was followed on a 0.200 mmol scale. Purification via column chromatography (silica gel, 5 – 10 % ethyl acetate in hexanes) afforded **5c** (41.3 mg, 73 % yield).

Physical state: pale yellow oil. $\mathbf{R_f}$ = 0.5 (10 % ethyl acetate in hexanes, vis. iodine).

^{1}**H NMR** (500 MHz, $CDCl_3$) δ 7.75 – 7.51 (m, 2H), 7.53 – 7.33 (m, 3H), 3.09 – 2.79 (m, 1H), 2.31 – 2.03 (m, 2H), 1.89 – 1.74 (m, 1H), 1.60 (ddt, J = 12.6, 10.9, 3.4 Hz, 2H), 1.49 – 1.24 (m, 4H), 1.14 (dddd, J = 9.5, 4.0, 2.6, 1.4 Hz, 1H), 1.03 (ddd, J = 12.6, 7.5, 2.5 Hz, 1H, major isomer), 0.93 – 0.80 (m, 2H), 0.58 – 0.45 (m, 1H, minor isomer).

^{13}**C NMR** (126 MHz, $CDCl_3$) δ 130.3, 129.9, 129.9, 129.0, 128.9, 128.5, 128.3, 123.8 (q, J = 278.6 Hz), 123.8 (q, J = 278.6 Hz), 66.4, 65.0, 64.6 (d, J = 34.4 Hz), 63.4 (q, J = 34.5 Hz), 42.2, 41.2, 37.9, 36.6, 36.3, 36.0, 35.6, 35.6, 28.7, 28.6, 26.3.

^{19}**F NMR** (471 MHz, $CDCl_3$) δ -74.90, -75.03.

HRMS: *m/z* calculated for $C_{15}H_{19}F_3N_2^+$: 283.1417 [M+H$^+$]; found 282.1416.

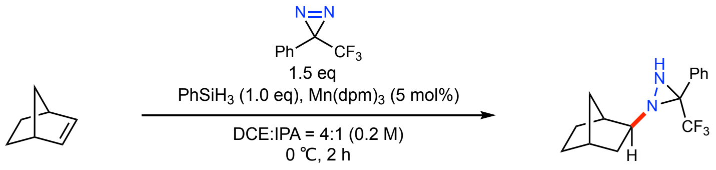

5d

General procedure C was followed on a 0.200 mmol scale. Purification via column chromatography (silica gel, 5% to 10% ethyl acetate in hexanes) afforded **5d** (47.3 mg, 84% yield).

Characterization data matched with **5c**.

1-(bicyclo[1.1.1]pentan-1-yl)-3-phenyl-3-(trifluoromethyl)diaziridine (5e)

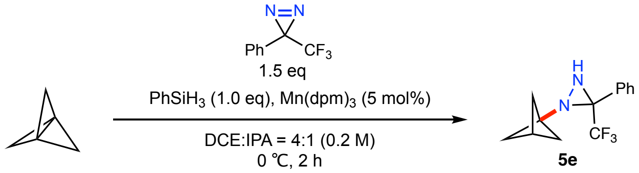

General procedure C was followed on a 0.200 mmol scale. [1.1.1]Propellane was added as a solution in ether/pentane. (See preparation of [1.1.1]propellane on page S34.) Purification via column chromatography (silica gel, 2% ethyl acetate in hexanes) afforded **5e** (35.8 mg, 72% yield).

Physical state: colorless oil. $\mathbf{R_f}$ = 0.7 (10% ethyl acetate in hexanes, vis. iodine).

^{1}H NMR (500 MHz, $CDCl_3$) δ 7.63 (d, *J* = 7.3 Hz, 2H), 7.49 – 7.36 (m, 3H), 3.11 – 2.85 (m, 1H), 2.16 (s, 1H), 1.60 (dd, *J* = 9.3, 1.8 Hz, 3H), 1.29 (dd, *J* = 9.3, 1.8 Hz, 3H).

^{13}C NMR (126 MHz, $CDCl_3$) δ 130.9, 130.1, 128.7, 127.9, 123.5 (d, *J* = 278.8 Hz), 63.4 (q, *J* = 34.5 Hz), 57.2, 51.2, 24.2.

^{19}F NMR (471 MHz, $CDCl_3$) δ -75.70.

HRMS: *m/z* calculated for $C_{13}H_{14}F_3N_2^+$: 255.1104 [M+H$^+$]; found: 255.1106.

1-cyclooctyl-3-phenyl-3-(trifluoromethyl)diaziridine (5f)

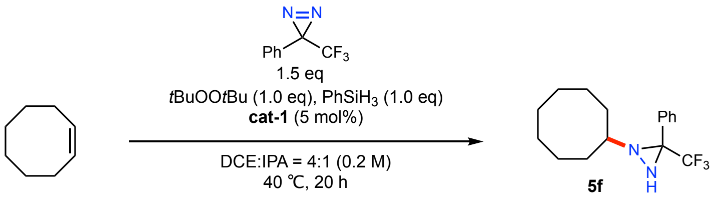

General procedure A was followed on a 0.100 mmol scale. Purification via column chromatography (silica gel, 0 – 5% ethyl acetate in hexanes) afforded **5f** (24.5 mg, 82 % yield).

Physical state: pale yellow oil. $\mathbf{R_f}$ = 0.6 (5 % ethyl acetate in hexanes, vis. iodine).

^{1}H NMR (500 MHz, $CDCl_3$) δ 7.68 (dd, J = 7.9, 1.8 Hz, 2H), 7.54 – 7.37 (m, 3H), 2.94 (s, 1H), 1.89 – 1.73 (m, 1H), 1.68 (qd, J = 7.9, 3.2 Hz, 3H), 1.62 – 1.46 (m, 4H), 1.46 – 1.36 (m, 3H), 1.36 – 1.30 (m, 1H), 1.26 (ddt, J = 11.7, 8.5, 3.2 Hz, 1H), 1.17 (tdd, J = 14.2, 7.6, 3.9 Hz, 1H), 1.05 (dddd, J = 15.6, 13.3, 6.6, 2.9 Hz, 1H), 0.91 – 0.77 (m, 1H).

^{13}C NMR (126 MHz, $CDCl_3$) δ 130.2, 130.0, 128.5, 128.3, 123.9 (q, J = 278.6 Hz), 63.9 (q, J = 34.1 Hz), 61.3, 33.1, 30.3, 27.0, 26.2, 26.1, 24.8, 23.7.

^{19}F NMR (471 MHz, $CDCl_3$) δ -73.91.

HRMS: m/z calculated for $C_{16}H_{23}F_3N_2^+$: 299.1730 [M+H$^+$]; found: 299.1726.

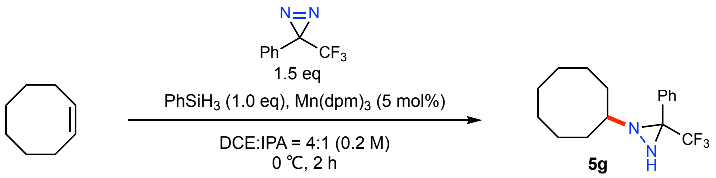

General procedure C was followed on a 0.100 mmol scale. Purification via column chromatography (silica gel, 0 to 5% ethyl acetate in hexanes) afforded **5g** (27.6 mg, 93% yield).

Characterization data matched with **5f**.

2-methoxy-4-(2-(3-phenyl-3-(trifluoromethyl)diaziridin-1-yl)propyl)phenol (6a)

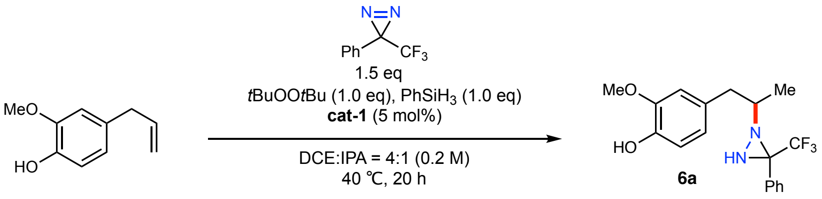

General procedure A was followed on a 0.100 mmol scale. Purification via column chromatography (silica gel, 5 – 10% ethyl acetate in hexanes) afforded **6a** (21.9 mg, 62% yield).

Physical state: pale yellow oil. **R_f** = 0.3 (35% ethyl acetate in hexanes, vis. UV and iodine).

^{1}H NMR: Major isomer: (500 MHz, $CDCl_3$) δ 7.71 (ddd, J = 7.4, 5.1, 1.7 Hz, 2H), 7.55 – 7.35 (m, 3H), 6.69 (d, J = 8.0 Hz, 1H), 6.26 (dd, J = 8.0, 1.9 Hz, 1H), 5.96 (d, J = 1.9 Hz, 1H), 5.40 (s, 1H), 3.66 (s, 3H), 3.01 (d, J = 2.4 Hz, 1H), 2.66 (dd, J = 13.3, 3.7 Hz, 1H), 2.40 (dd, J = 13.3, 9.5 Hz, 1H), 1.85 (dtt, J = 13.4, 6.6, 3.2 Hz, 1H), 1.05 (d, J = 6.2 Hz, 3H).

Minor isomer: (500 MHz, $CDCl_3$) δ 7.71 (ddd, J = 7.4, 5.1, 1.8 Hz, 2H), 7.55 – 7.35 (m, 3H), 6.76 (d, J = 8.5 Hz, 1H), 6.59 – 6.49 (m, 2H), 5.43 (s, 1H), 3.82 (s, 3H), 3.01 (d, J = 2.4 Hz, 1H), 2.98 (dd, J = 13.3, 4.1 Hz, 1H), 2.56 (dd, J = 13.3, 9.4 Hz, 1H), 1.85 (tdd, J = 9.8, 6.5, 3.7 Hz, 1H), 0.83 (d, J = 6.3 Hz, 3H).

^{13}C NMR: (126 MHz, $CDCl_3$, mixture of diasteromers) δ 146.1, 143.9, 130.6, 130.0, 129.9, 129.8, 128.3, 128.2, 128.0, 123.8 (d, J = 279.3 Hz), 122.2, 122.0, 114.0, 113.9, 112.0, 111.4, 63.8 (dd, J = 34.5, 19.4 Hz), 59.0, 58.8, 55.8, 55.7, 42.2, 40.2, 18.6, 16.6.

^{19}F NMR: (471 MHz, $CDCl_3$) major isomer: δ -73.57, minor isomer: δ -74.10.

HRMS: Calculated for $C_{18}H_{20}F_3N_2O_2^+$ 353.1471 [M+H$^+$]; found 353.1475.

(4*R*,4a*S*,6*R*)-4,4a-dimethyl-6-(2-(3-phenyl-3-(trifluoromethyl)diaziridin-1-yl)propan-2-yl)-4,4a,5,6,7,8-hexahydronaphthalen-2(3*H*)-one (6b)

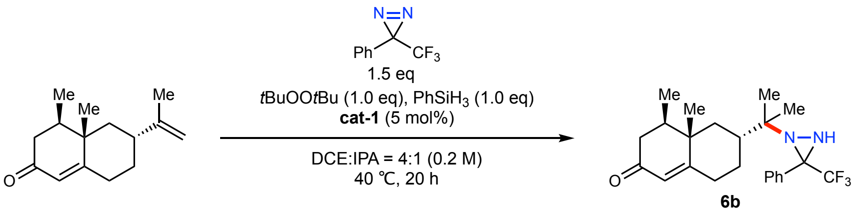

General procedure A was followed on a 0.115 mmol scale. Purification via column chromatography (silica gel, 10% – 17% ethyl acetate in hexanes) afforded **6b** (19.4 mg, 42% yield).

Physical state: pale yellow oil. $\mathbf{R_f}$ = 0.3 (17% ethyl acetate in hexanes, vis. iodine).

^{1}H NMR (500 MHz, $CDCl_3$) δ 7.85 – 7.62 (m, 2H), 7.48 – 7.36 (m, 3H), 2.86 – 2.70 (m, 1H), 2.60 – 2.11 (m, 5H), 2.11 – 1.71 (m, 3H), 1.40 – 1.28 (m, 1H), 1.24 – 1.17 (m, 1H), 1.17 – 1.09 (m, 2H), 1.05 (s, 2H), 1.03 – 0.81 (m, 5H), 0.80 – 0.56 (m, 6H).

^{13}C NMR (126 MHz, $CDCl_3$) δ 199.8, 199.7, 170.9, 131.4, 129.8, 129.8, 129.7, 129.4, 129.2, 128.7, 128.7, 128.0, 127.9, 127.8, 124.4, 124.4, 123.8 (d, *J* = 279.3 Hz), 113.9, 61.6, 61.5 (d, *J* = 34.1 Hz), 61.4, 61.1 (d, *J* = 34.1 Hz), 44.1, 43.4, 42.1, 40.6, 40.5, 39.8, 39.3, 39.2, 39.2, 33.1, 28.0, 27.4, 22.1, 21.9, 21.9, 21.2, 16.9, 16.9, 15.0.

^{19}F NMR (471 MHz, $CDCl_3$) δ -75.27, -75.44.

HRMS: Calculated for $C_{23}H_{30}F_3N_2O^+$ 407.2305. [M+H$^+$]; found 407.2309.

(1'*R*,2'*R*)-5'-methyl-4-pentyl-2'-(2-((1*R*)-3-phenyl-3-(trifluoromethyl)-1□^{4}-diaziridin-1-yl)propan-2-yl)-1',2',3',4'-tetrahydro-[1,1'-biphenyl]-2,6-diol (6c)

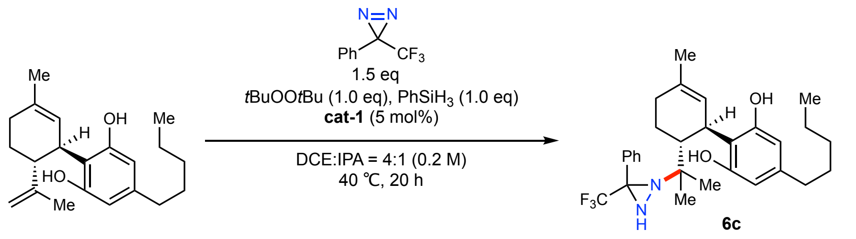

General procedure A was followed on a 0.100 mmol scale. Purification via column chromatography (silica gel, 2 – 5% ethyl acetate in hexanes) afforded **6c** (28.6 mg, 57% yield, dr = 6:4).

Physical state: pale yellow foam. $\mathbf{R_f}$ = 0.5 (10% ethyl acetate in hexanes, vis. UV and iodine).

^{1}H NMR major isomer (500 MHz, $CDCl_3$) δ 7.71 (t, *J* = 9.1 Hz, 2H), 7.50 – 7.35 (m, 4H), 6.85 (s, 1H), 6.33 (s, 1H), 6.22 (s, 1H), 5.84 (d, *J* = 4.7 Hz, 1H), 4.06 – 3.82 (m, 1H), 3.04 (s, 1H), 2.46 (dd, *J* = 8.8, 6.6 Hz, 2H), 2.12 – 2.02 (m, 1H), 1.98 (dd, *J* = 9.8, 3.4 Hz, 2H), 1.86 (d, *J* = 4.7 Hz, 1H), 1.81 (d, *J* = 2.1 Hz, 3H), 1.59 (d, *J* = 7.6 Hz, 2H), 1.31 (ddt, *J* = 10.1, 6.7, 5.0 Hz, 5H), 1.08 (s, 3H), 0.91 – 0.87 (m, 3H), 0.42 (s, 3H).

Minor isomer (500 MHz, $CDCl_3$) δ 7.70 – 7.62 (m, 2H), 7.50 – 7.31 (m, 4H), 6.42 (s, 1H), 6.29 (s, 1H), 5.80 (s, 1H), 5.52 – 5.37 (m, 1H), 4.10 (dq, *J* = 9.3, 2.7 Hz, 1H), 3.05 (s, 1H), 2.48 (td, *J* = 7.6, 4.0 Hz, 2H), 2.08 (qd, *J* = 4.7, 2.3 Hz, 4H), 1.76 (t, *J* = 1.8 Hz, 5H), 1.31 (dt, *J* = 7.6, 3.1 Hz, 4H), 0.95 – 0.83 (m, 8H), 0.35 (s, 3H).

^{13}C NMR (126 MHz, $CDCl_3$) δ 156.4, 154.7, 143.8, 143.7, 140.8, 139.6, 131.5, 131.2, 130.3, 130.2, 130.2, 129.7, 129.6, 129.4, 129.3, 129.1, 129.0, 128.9, 128.9, 128.8, 128.2, 128.2, 128.1, 125.3, 124.0, 123.6 (d, *J* = 280.7 Hz), 116.2, 113.2, 110.0, 109.3, 108.8, 64.1, 63.3 (d, *J* = 34.1 Hz), 62.6, 60.7 (d, *J* = 35.0 Hz), 50.8, 48.4, 35.7, 35.6, 34.8, 33.8, 31.7, 31.0, 30.8, 28.0, 26.2, 24.9, 23.8, 23.7, 23.3, 22.7, 22.4, 22.0, 20.7, 14.2, 14.2.

^{19}F NMR (471 MHz, $CDCl_3$) Major isomer: δ -74.77. Minor isomer: δ -75.31.

HRMS: Calculated for $C_{29}H_{38}F_3N_2O_2^+$ 503.2880. [M+H$^+$]; found 503.2888.

((4*S*)-4-(2-(3-phenyl-3-(trifluoromethyl)diaziridin-1-yl)propan-2-yl)cyclohex-1-en-1-yl)methanol (6d)

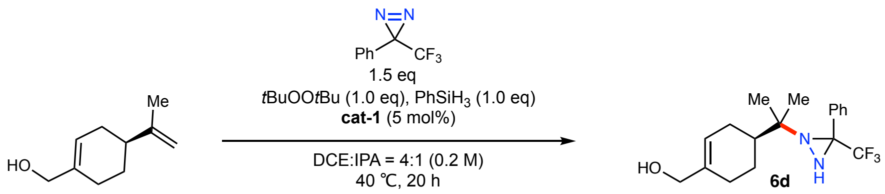

General procedure A was followed on a 0.100 mmol scale. Purification via column chromatography (silica gel, 10% ethyl acetate in hexanes) afforded **6d** (23.5 mg, 69% yield).

Physical state: pale yellow oil. $\mathbf{R_f}$ = 0.3 (20% ethyl acetate in hexanes, vis. UV and iodine).

^{1}H NMR: (500 MHz, $CDCl_3$, mixture of diastereomers) δ 7.82 – 7.60 (m, 2H), 7.44 – 7.36 (m, 3H), 5.82 – 5.59 (m, 1H), 4.08 – 3.92 (m, 2H), 2.83 – 2.64 (m, 1H), 2.30 – 1.83 (m, 5H), 1.67 – 1.58 (m, 1H), 1.30 – 1.22 (m, 2H), 0.85 – 0.52 (m, 6H).

^{13}C NMR: (126 MHz, $CDCl_3$, mixture of diastereomers) δ 137.54 137.2, 131.4, 130.0, 129.61, 129.6, 128.6, 127.9, 123.1, 123.9 (d, J = 273.4 Hz), 122.6, 67.3, 67.2, 61.4 (d, J = 16.2 Hz), 45.7, 45.0, 26.8, 26.8, 26.6, 26.2, 23.91, 23.4, 22.3, 21.8, 21.3, 20.8.

^{19}F NMR (471 MHz, $CDCl_3$) major isomer: δ -75.54, minor isomer: δ -75.52.

HRMS: Calculated for $C_{18}H_{24}F_3N_2O^+$ 341.1835 [M+H$^+$]; found 341.1843.

1-(2-(6-methyl-7-oxabicyclo[4.1.0]heptan-3-yl)propan-2-yl)-3-phenyl-3-(trifluoromethyl)diaziridine (6e)

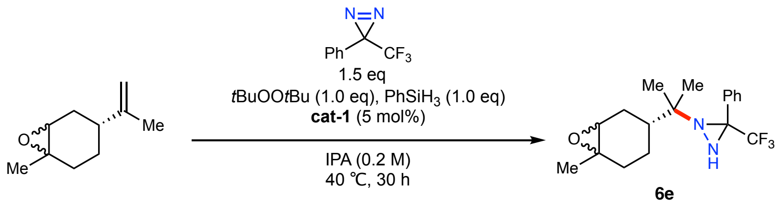

General procedure B was followed on a 0.100 mmol scale. Purification via column chromatography (silica gel, 0 – 10% ethyl acetate in hexanes) afforded **6e** (17.6 mg, 52% yield).

Physical state: pale yellow oil. $\mathbf{R_f}$ = 0.3 (20% ethyl acetate in hexanes, iodine).

^{1}H NMR: (500 MHz, $CDCl_3$, mixture of diastereomers) δ 7.67 (dd, J = 13.7, 6.9 Hz, 2H), 7.49 – 7.31 (m, 3H), 3.06 – 2.93 (m, 1H), 2.80 – 2.63 (m, 1H), 2.15 – 1.83 (m, 2H), 1.66 (dtd, J = 17.2, 13.8, 11.2 Hz, 2H), 1.49 – 1.33 (m, 1H), 1.32 (t, J = 2.8 Hz, 3H), 1.30 – 1.09 (m, 2H), 0.85 – 0.38 (m, 6H).

^{13}C NMR: (126 MHz, $CDCl_3$) δ 131.4, 130.0, 129.6, 129.6, 128.6, 127.9, 123.8 (d, J = 279.5 Hz), 61.4, 61.3, 61.2, 59.6, 59.5, 57.7, 45.0, 44.2, 31.2, 31.2, 25.9, 25.4, 23.0, 23.0, 22.2, 21.6, 20.9, 20.3, 20.2, 19.9.

^{19}F NMR: (471 MHz, $CDCl_3$) δ -75.48.

HRMS: Calculated for $C_{18}H_{24}F_3N_2O^+$ 341.1835 [M+H$^+$]; found 341.1834.

(1*R*,2*S*,5*R*)-5-Methyl-2-(2-(3-phenyl-3-(trifluoromethyl)diaziridin-1-yl)propan-2-yl)cyclohexan-1-ol (6f)

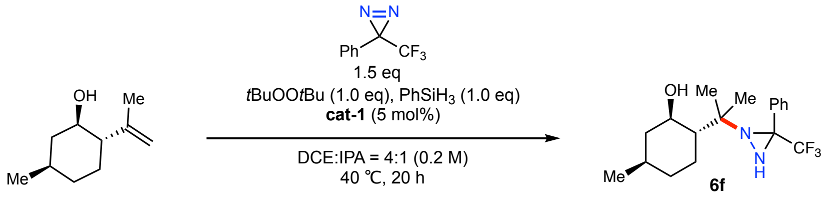

General procedure A was followed on a 0.100 mmol scale. Purification via column chromatography (silica gel, 5 – 40% ethyl acetate in hexanes) afforded **6f** (24.0 mg, 70% yield).

Physical state: yellow oil. $\mathbf{R_f}$=0.5 (30% ethyl acetate in hexanes, vis. iodine and CAM.

^{1}H NMR (600 MHz, $CDCl_3$, mixture of diastereomers) δ 7.61 – 7.48 (m, 2H), 7.33 – 7.24 (m, 3H), 5.41 (s, 1H), 3.59 (tdd, J = 10.3, 6.3, 4.2 Hz, 1H), 2.78 (s, 1H), 1.95 – 1.88 (m, 1H), 1.84 (dq, J = 12.6, 3.5 Hz, 1H), 1.55 – 1.21 (m, 5H), 1.14 – 1.00 (m, 4H), 1.00 – 0.84 (m, 2H), 0.84 – 0.65 (m, 6H), 0.23 – -0.02 (m, 3H).

^{13}C NMR (151 MHz, $CDCl_3$, mixture of diastereomers) δ 131.1, 131.0, 130.1, 130.0, 129.5, 129.4, 129.0, 128.9, 128.8, 128.2, 128.1, 123.3 (qd, $J_{C–F}$ = 280.7, 57.9 Hz), 62.6 (qd, $J_{C–F}$ = 34.5, 18.8z Hz), 77.2, 77.0, 76.8, 72.2, 72.0, 64.9, 64.8, 52.5, 51.8, 44.3, 44.0, 34.8, 34.7, 30.9, 25.9, 25.4, 23.7, 22.0, 21.1, 20.5, 19.2.

^{19}F NMR: (564 MHz, $CDCl_3$, mixture of diastereomers) δ -76.35, -76.39.

HRMS: *m/z* calculated for $C_{18}H_{26}F_3N_2O^+$ 343.1992 [M+H$^+$]; found 343.1988.

1-(2-((2*R*,8*R*,8a*S*)-8,8a-dimethyl-1,2,3,4,6,7,8,8a-octahydronaphthalen-2-yl)propan-2-yl)-3-phenyl-3-(trifluoromethyl)diaziridine (6g)

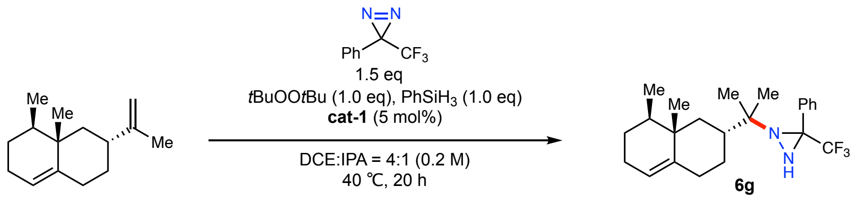

General procedure A was followed on a 0.100 mmol scale. Purification via column chromatography (silica gel, 0 – 2% ethyl acetate in hexanes) afforded **6g** (20.4 mg, 52% yield).

Physical state: pale yellow oil. $\mathbf{R_f}$ = 0.7 (10% ethyl acetate in hexanes, vis. iodine).

^{1}H NMR: (500 MHz, $CDCl_3$, mixture of diastereomers) δ 7.70 (td, *J* = 9.4, 4.1 Hz, 2H), 7.49 – 7.31 (m, 3H), 5.40 – 5.19 (m, 1H), 2.72 (d, *J* = 10.7 Hz, 1H), 2.35 – 1.79 (m, 5H), 1.78 – 1.63 (m, 2H), 1.49 – 1.24 (m, 4H), 1.21 (d, *J* = 6.0 Hz, 1H), 1.20 – 0.90 (m, 4H), 0.88 (s, 2H), 0.83 (dd, *J* = 11.5, 6.5 Hz, 2H), 0.79 – 0.49 (m, 6H).

^{13}C NMR: (126 MHz, $CDCl_3$, mixture of diastereomers) δ 144.9, 144.3, 143.6, 143.5, 134.9, 131.4, 130.1, 129.9, 129.6, 129.5, 129.5, 129.4, 128.5, 127.9, 127.8, 127.6, 123.9 (d, *J* = 279.7 Hz), 119.7, 119.2, 118.7, 65.4, 61.9, 61.8, 61.6, 61.4, 61.2 (d, *J* = 34.1 Hz), 44.2, 44.2, 44.1, 44.0, 43.5, 41.8, 41.5, 41.1, 41.0, 41.0, 40.7, 40.3, 39.9, 39.2, 38.7, 38.7, 38.5, 37.8, 37.7, 37.7, 32.8, 32.8, 32.5, 32.5, 31.2, 29.7, 29.3, 29.2, 28.7, 27.8, 27.6, 27.2, 27.2, 27.1, 25.9, 25.9, 25.5, 24.8, 22.2, 22.1, 22.1, 22.0, 21.8, 21.8, 21.5, 21.4, 21.2, 18.5, 18.0, 17.8, 15.8, 15.8, 15.8, 15.7.

^{19}F NMR: (471 MHz, $CDCl_3$) δ -75.28, -75.39, -75.40, -75.49.

HRMS: Calculated for $C_{23}H_{32}F_3N_2^+$ 393.2512. [M+H$^+$]; found 393.2516.

6-methyl-2-((*S*)-4-methylcyclohex-3-en-1-yl)-6-(3-phenyl-3-(trifluoromethyl)diaziridin-1-yl)heptan-2-ol (6h)

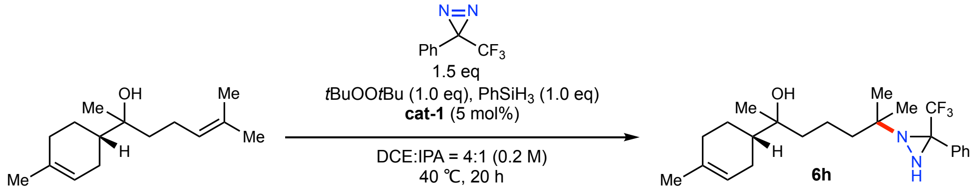

General procedure A was followed on a 0.100 mmol scale. Purification via column chromatography (silica gel, 5 – 10% ethyl acetate in hexanes) afforded **6h** (26.6 mg, 65% yield).

Physical state: pale yellow foam. $\mathbf{R_f}$ = 0.3 (20% ethyl acetate in hexanes, vis. iodine).

^{1}H NMR: (500 MHz, $CDCl_3$) δ 7.67 (dd, *J* = 14.3, 8.2 Hz, 2H), 7.47 – 7.35 (m, 3H), 5.49 – 5.27 (m, 1H), 2.74 (s, 1H), 2.06 – 1.93 (m, 3H), 1.90 (ddt, *J* = 12.4, 5.2, 2.3 Hz, 1H), 1.83 – 1.75 (m, 1H), 1.56 (tdt, *J* = 11.4, 4.7, 2.2 Hz, 2H), 1.50 – 1.34 (m, 6H), 1.34 – 1.20 (m, 2H), 1.17 – 1.12 (m, 1H), 1.11 (d, *J* = 2.9 Hz, 3H), 0.75 (d, *J* = 6.6 Hz, 3H), 0.67 (d, *J* = 6.4 Hz, 3H).

^{13}C NMR: (126 MHz, $CDCl_3$, mixture of diastereomers) δ 134.2, 134.2, 133.9, 133.9, 131.3, 130.0, 130.0, 129.6, 129.3, 128.6, 127.9, 123.8 (d, *J* = 279.7 Hz), 120.6, 74.3, 74.3, 74.3, 61.8 (d, *J* = 34.1 Hz), 59.7, 43.6, 43.5, 43.5, 43.4, 43.1, 42.9, 42.8, 41.0, 41.0, 40.3, 40.1, 31.0, 31.0, 26.9, 26.9, 26.1, 26.1, 24.5, 24.4, 24.2, 24.2, 24.0, 23.9, 23.8, 23.8, 23.8, 23.5, 23.4, 23.4, 23.4, 23.3, 17.9, 17.9, 17.7, 17.6.

^{19}F NMR: (471 MHz, $CDCl_3$) δ -75.64, -75.65.

HRMS: Calculated for $C_{23}H_{34}F_3N_2O^+$ 411.2618. [M+H$^+$]; found 411.2618.

(1R,2R,8R)-1-((3R)-3-hydroxy-3-methyl-4-(3-phenyl-3-(trifluoromethyl)diaziridin-1-yl)pentyl)-2,5,5,8-tetramethyldecahydronaphthalen-2-ol (6i)

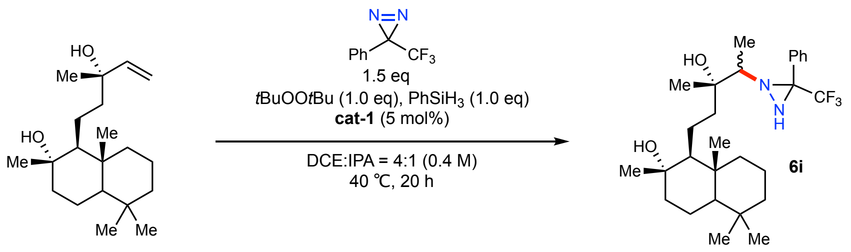

General procedure A was followed on a 0.105 mmol scale on 0.4 M concentration. Purification via column chromatography (silica gel, 10 – 20% ethyl acetate in hexanes) afforded **6i** (37.5 mg, 72% yield).

Physical state: yellow oil. $\mathbf{R_f}$ = 0.4 (30% ethyl acetate in hexanes, vis. iodine and CAM).

^{1}H NMR: (500 MHz, $CDCl_3$, mixture of diastereomers) δ 7.66 (ddd, J = 16.2, 7.6, 3.2 Hz, 2H), 7.55 – 7.34 (m, 3H), 3.21 – 2.64 (m, 2H), 1.96 – 1.71 (m, 3H), 1.71 – 1.29 (m, 10H), 1.29 – 1.18 (m, 4H), 1.18 – 1.00 (m, 7H), 1.00 – 0.91 (m, 2H), 0.91 – 0.80 (m, 7H), 0.80 – 0.71 (m, 6H).

^{13}C NMR: (126 MHz, $CDCl_3$, mixture of diastereomers) δ 130.4, 130.4, 130.3, 130.1, 128.7, 128.6, 128.6, 128.6, 128.5, 128.5, 128.4, 127.4, 127.3, 123.8 (d, J = 278.8 Hz, three different sets), 76.4, 75.8, 75.0, 74.7, 74.6, 74.4, 64.0 (d, J = 34.1 Hz), 63.1, 62.4, 62.4, 62.3, 62.1, 61.7, 56.3, 56.2, 56.2, 44.2, 44.0, 43.3, 42.4, 42.2, 42.1, 41.0, 40.4, 39.8, 39.8, 39.7, 39.2, 39.2, 33.5, 33.5, 33.4, 33.4, 24.6, 24.4, 24.3, 23.5, 21.6, 21.5, 20.6, 20.5, 20.5, 18.6, 18.6, 18.4, 18.3, 17.9, 15.6, 15.5, 15.5, 14.2, 14.2, 13.6.

^{19}F NMR: (471 MHz, $CDCl_3$) δ -73.43, -73.93, -74.02.

HRMS: Calculated for $C_{28}H_{44}F_3N_2O_2^+$ 497.3349. [M+H$^+$]; found 497.3355.

3-phenyl-3-(trifluoromethyl)-1-((1*S*,2*R*,4*R*)-2,3,3-trimethylbicyclo[2.2.1]heptan-2-yl)diaziridine (6j)

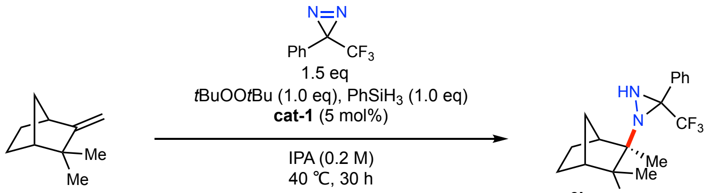

General procedure B was followed on a 0.100 mmol scale. Purification via column chromatography (silica gel, 2% ethyl acetate in hexanes) afforded **6j** (24.1 mg, 75% yield).

Physical state: Colorless oil. $\mathbf{R_f}$ = 0.7 (10% ethyl acetate in hexanes, vis. iodine).

^{1}H NMR (major isomer): (500 MHz, $CDCl_3$) δ 7.71 (dd, *J* = 7.6, 1.8 Hz, 2H), 7.44 – 7.33 (m, 3H, merged with minor isomer), 2.69 (s, 1H, merged with minor isomer), 2.43 (dtd, *J* = 9.8, 2.5, 1.3 Hz, 1H), 1.80 (dd, *J* = 4.4, 1.8 Hz, 1H), 1.72 – 1.67 (m, 1H), 1.28 – 1.10 (m, 7H, merged with minor isomer), 1.00 (dt, *J* = 9.7, 1.5 Hz, 1H), 0.80 (s, 3H), 0.49 (s, 3H).

Minor isomer (500 MHz, $CDCl_3$) δ 7.66 (dd, *J* = 7.9, 1.8 Hz, 2H), 7.43 – 7.33 (m, 3H, merged with major isomer), 2.67 (s, 1H, merged with major isomer), 2.59 – 2.53 (m, 1H), 2.16 (dd, *J* = 4.4, 1.7 Hz, 1H), 1.76 (dt, *J* = 2.8, 1.6 Hz, 1H), 1.30 – 1.09 (m, 8H, merged with major isomer), 0.82 (s, 3H), 0.20 (s, 3H).

^{13}C NMR (126 MHz, $CDCl_3$) δ 130.8, 130.6, 129.6, 129.5, 128.4, 125.2, 123.0, 69.5, 69.1, 62.8, 60.8, 50.4, 50.4, 49.0, 48.8, 46.0, 45.1, 35.0, 34.7, 29.9, 26.8, 24.2, 23.9, 23.8, 23.5, 23.3, 23.2, 16.1, 13.8.

^{19}F NMR (471 MHz, $CDCl_3$) **major isomer:** δ -75.75. **Minor isomer:** δ -75.71

HRMS Calculated for $C_{18}H_{24}F_3N_2^+$ 325.1886 [M+H$^+$]; found 325.1892.

1-(2-((*R*)-4-methylcyclohex-3-en-1-yl)propan-2-yl)-3-phenyl-3-(trifluoromethyl)diaziridine (6k)

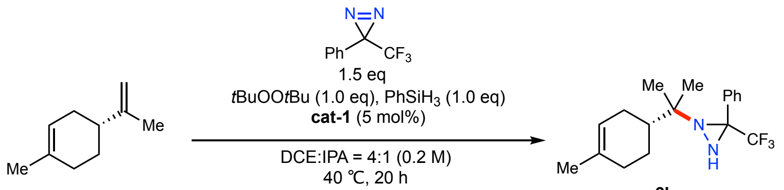

General procedure A was followed on a 0.100 mmol scale. Purification via column chromatography (silica gel, 0 – 2% ethyl acetate in hexanes) afforded **6k** (27.0 mg, 83% yield).

Physical state: pale yellow oil. **R$_f$** = 0.2 (100% hexanes, vis. iodine).

^{1}H NMR: (500 MHz, $CDCl_3$) δ 7.75 – 7.50 (m, 2H), 7.37 – 7.16 (m, 3H), 5.38 – 5.21 (m, 1H), 2.69 – 2.56 (m, 1H), 2.04 (ddt, *J* = 12.5, 5.3, 2.3 Hz, 1H), 1.99 – 1.64 (m, 4H), 1.55 (s, 3H), 1.52 – 1.42 (m, 1H), 1.09-1.32 (m, 1H, mixture of diastereomers), 0.77 – 0.37 (m, 6H, mixture of diastereomers).

^{13}C NMR: (126 MHz, $CDCl_3$, mixture of diastereomers) δ 134.3, 133.8, 131.5, 130.3, 129.7, 129.6, 128.6, 128.1, 128.0, 124.0 (d, *J* = 279.8 Hz), 121.3, 120.9, 61.7, 61.6, 61.4 (d, *J* = 20.3 Hz), 61.1 (d, *J* = 20.0 Hz), 45.6, 44.9, 31.4, 31.3, 27.1, 26.6, 25.6, 24.4, 24.0, 23.5, 23.5, 22.3, 21.9, 21.5, 20.9.

^{19}F NMR: (471 MHz, $CDCl_3$) δ -75.51, -75.54.

HRMS: Calculated for $C_{18}H_{24}F_3N_2^+$ 325.1886 [M+H$^+$]; found 325.1892.

3,7-Dimethyl-7-(3-phenyl-3-(trifluoromethyl)diaziridin-1-yl)octanoic acid (6l)

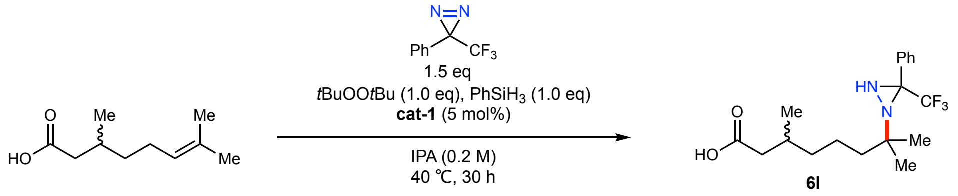

General procedure B was followed on a 0.200 mmol scale. Purification via column chromatography (silica gel, 0 – 5% MeOH in DCM) afforded **6l** (43.5 mg, 61% yield).

Physical state: yellow oil. $\mathbf{R_f}$ = 0.3 (10% ethyl acetate in hexanes, vis. iodine and CAM).

^{1}H NMR (500 MHz, $CDCl_3$) δ 7.80 – 7.56 (m, 2H), 7.40 (ddt, *J* = 11.4, 7.9, 3.5 Hz, 3H), 2.75 (s, 1H), 2.36 (ddd, *J* = 15.1, 6.0, 1.4 Hz, 1H), 2.16 (ddd, *J* = 15.1, 8.2, 4.8 Hz, 1H), 1.97 (hept, *J* = 6.1 Hz, 1H), 1.52 – 1.27 (m, 6H), 1.20 (td, *J* = 8.2, 5.4 Hz, 1H), 0.98 (d, *J* = 6.6 Hz, 3H), 0.75 (d, *J* = 5.8 Hz, 3H), 0.65 (d, *J* = 4.7 Hz, 3H).

^{13}C NMR (126 MHz, $CDCl_3$) δ 178.4, 178.4, 131.2, 130.0, 130.0, 129.6, 129.4, 128.6, 127.9, 123.8 (d, *J* = 280.2 Hz), 61.8 (d, *J* = 36.3 Hz), 59.6, 42.9, 42.8, 41.4, 41.3, 37.2, 30.1, 30.0, 24.4, 24.3, 23.8, 21.2, 19.7, 19.7.

^{19}F NMR (471 MHz, $CDCl_3$) δ -75.66, -75.67.

HRMS: *m/z* calculated for $C_{18}H_{26}F_3N_2O_2^+$: 359.1941 [M+H$^+$]; found: 359.1944.

3,7-Dimethyl-7-(3-phenyl-3-(trifluoromethyl)diaziridin-1-yl)octanal (6m)

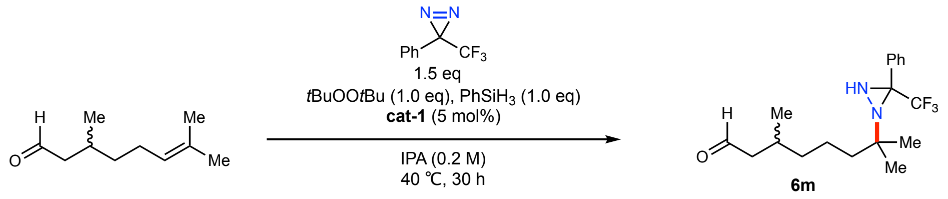

General procedure B was followed on a 0.100 mmol scale. Purification via column chromatography (silica gel, 3 – 10% ethyl acetate in hexanes) afforded **6m** (24.3 mg, 71% yield).

Physical state: pale yellow oil. $\mathbf{R_f}$ = 0.4 (10% ethyl acetate in hexanes, vis. iodine and CAM).

^{1}H NMR (500 MHz, $CDCl_3$) δ 9.76 (t, *J* = 2.7 Hz, 1H), 7.80 – 7.59 (m, 2H), 7.40 (ddt, *J* = 11.6, 7.9, 3.5 Hz, 3H), 2.81 – 2.66 (m, 1H), 2.40 (ddd, *J* = 16.0, 5.7, 2.1 Hz, 1H), 2.29 – 2.19 (m, 1H), 2.12 – 2.00 (m, 1H), 1.47 – 1.17 (m, 6H), 0.96 (d, *J* = 6.7 Hz, 3H), 0.75 (d, *J* = 3.8 Hz, 3H), 0.65 (d, *J* = 2.7 Hz, 3H).

^{13}C NMR (126 MHz, $CDCl_3$) δ 203.2, 131.4, 130.1, 130.1, 129.8, 129.4, 128.7, 128.0, 124.0 (q, *J* = 279.7 Hz), 61.9 (q, *J* = 34.1 Hz), 59.7, 51.2, 51.2, 43.0, 37.6, 28.2, 24.5, 24.5, 23.9, 21.4, 20.1, 20.1.

^{19}F NMR (471 MHz, $CDCl_3$) δ -75.68.

HRMS: *m/z* calculated for $C_{18}H_{26}F_3N_2O^+$: 343.1992 [M+H$^+$]; found: 343.1990.

3,7-Dimethyl-7-(3-phenyl-3-(trifluoromethyl)diaziridin-1-yl)octan-1-ol (6n)

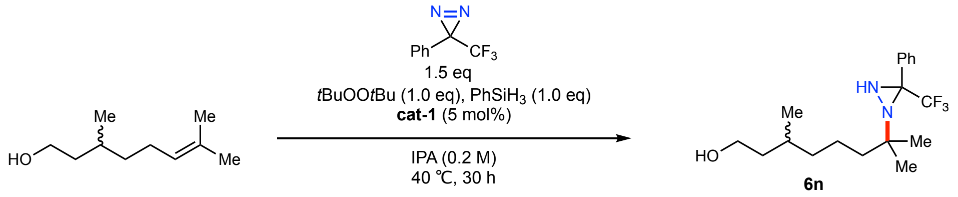

General procedure B was followed on a 0.100 mmol scale. Purification via column chromatography (silica gel, 0 – 10% ethyl acetate in hexanes) afforded **6n** (24.6 mg, 72% yield).

Physical state: pale yellow oil. $\mathbf{R_f}$ = 0.3 (20% ethyl acetate in hexanes, vis. iodine and CAM).

^{1}H NMR (500 MHz, $CDCl_3$) δ 7.80 – 7.58 (m, 2H), 7.45 – 7.32 (m, 3H), 3.79 – 3.56 (m, 2H), 2.85 – 2.58 (m, 1H), 1.73 – 1.06 (m, 12H), 0.95 – 0.84 (m, 3H), 0.74 (d, *J* = 4.6 Hz, 3H), 0.66 (d, *J* = 2.9 Hz, 3H).

^{13}C NMR (126 MHz, $CDCl_3$) δ 131.4, 130.2, 130.2, 129.7, 129.5, 128.7, 128.0, 124.0 (q, *J* = 279.7 Hz), 61.9 (q, *J* = 33.8 Hz), 61.4, 59.8, 43.2, 43.2, 40.1, 40.1, 37.9, 29.5, 29.5, 24.6, 24.5, 23.9, 21.4, 19.8, 19.7.

^{19}F NMR (471 MHz, $CDCl_3$) δ -75.67, -75.68.

HRMS: Calculated for $C_{18}H_{28}F_3N_2O^+C_{18}H_{27}F_3N_2NaO$ 367.1968 [M+Na$^+$]; found 367.1972.

Proposed Mechanism:

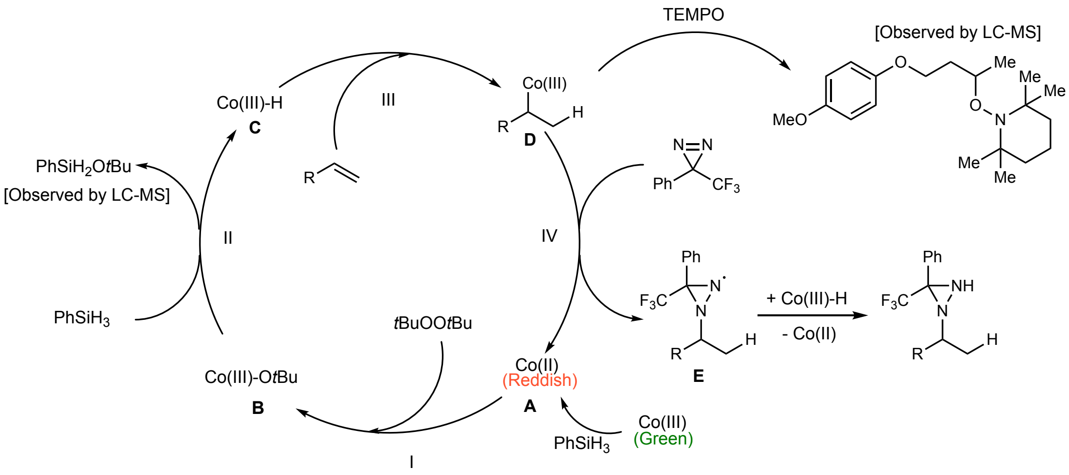

Supplementary Figure 9: Proposed mechanism of hydroamination reaction with diazirine **1.**

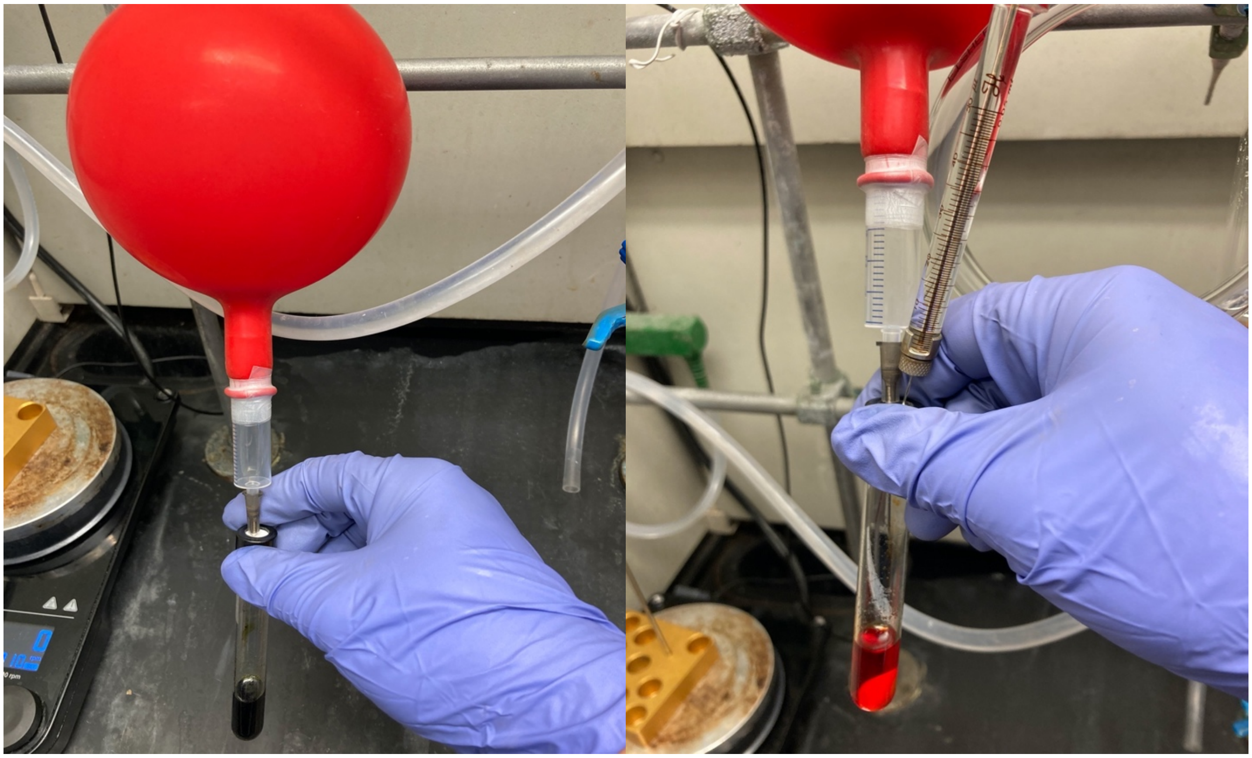

Supplementary Figure 10: Left: Before addition of $PhSiH_3$, the Co(III) complex showed a dark green color.
Right: After addition of $PhSiH_3$, the system became a reddish Co(II) color .

A plausible mechanism was proposed based on our observations and literature reports. [30-33]The original Co(III)-Salen-OTs was reduced to Co(II) after the addition of $PhSiH_3$. This can be supported by the reddish mixture after addition and a similar result can be obtained with the Co(II) complex. The catalytic cycle is proposed as follow:

I: The Co(II) complex **A** was oxidized by peroxide to form complex **B**. It was reported by Nojima[34] that the cobalt oxygen bond can facilitate formation of the cobalt hydride complex, which agreed with our observation that lower yields were obtained without *t*-BuOO*t*-Bu.

II: The oxidized cobalt species then exchanged ligands with $PhSiH_3$ to afford cobalt hydride complex **C**.[34] $PhSiH_2O$*t*Bu was detected by LC-MS (calculated 181.1 [M+H$^+$], found 181.0) within the crude reaction mixtures, which supports the proposed ligand exchange.

III: Cobalt hydride complex **C** underwent migratory insertion into the double bond of the substrate, with cobalt bonded to the more substituted side due to the stability of the radical and produced **D**.

IV: The carbon-cobalt complex **D** cleaved into complex **A** and the corresponding carbon radical, which can be captured by TEMPO (observed by LC-MS, calculated 336.3 [M+H$^+$], found 336.2). The carbon radical is presumably quenched by the diazirine and forms diaziridinyl radical **E**, which was then quenched by a hydrogen radical source to deliver the product. Running the reaction in *i*PrOD did not show any deuterium incorporation into the product (see NMR below), suggesting that both protons originate from the silane as hydrogen radicals.

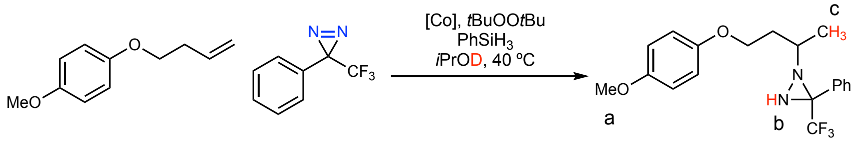

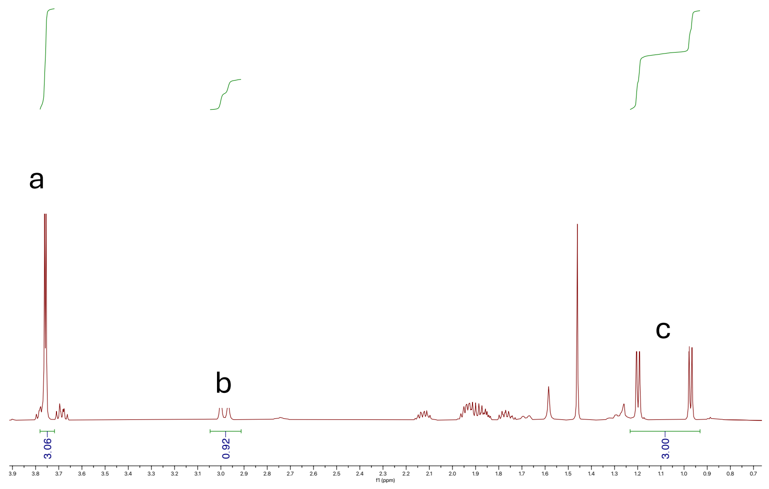

Supplementary Figure 11: Hydroamination reaction with *i*PrOD as solvent.

Applications:

Quinocide hydrochloride (8)

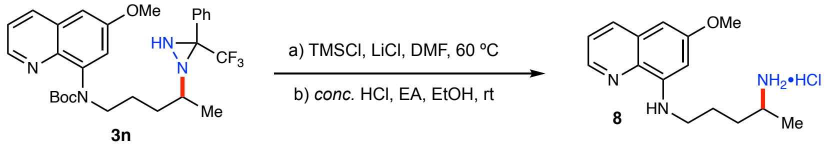

Followed our previously reported procedure. [1] To a septum capped vial equipped with a magnetic stir bar was added **3n** (24.6 mg, 0.046 mmol, 1 eq) and dimethylformamide (0.2 mL). Lithium chloride (5.9 mg, 0.138 mmol, 3 eq) and trimethylsilyl chloride (60.0 mg, 70.1 µL, 0.552 mmol, 12 eq) were added, then the vial was sealed and stirred at 60 ºC for 24 h. To the reaction mixture, 10% NaOH (20 mL) was added, and the mixture was extracted with dichloromethane (20 mL x 3), washed with water (50 mL), and brine (20 mL), dried with *anhyd.* Na_2SO_4, concentrated *in vacuo* and purified by flash column chromatography (basic alumina, 5% MeOH in DCM to 20% MeOH in DCM) to afford the crude product. It was was dissolved in ethyl acetate (1 mL) and ethanol (1 mL). Conc. HCl (200 µL) was added, the reaction mixture stirred for 1 h and concentrated. The residue was washed with several drops of dichloromethane, which gave 11.0 mg (80%) of **8** as a brown solid.

The ^{1}H NMR spectrum matched with previously reported ^{1}H NMR. [35]

Physical state: brown solid. $\mathbf{R_f}$ = 0.3 (20% MeOH in DCM, vis. UV and PMA).

^{1}H NMR (500 MHz, DMSO) δ 8.66 – 8.56 (m, 1H), 8.35 – 8.17 (m, 1H), 7.87 (s, 3H), 7.62 – 7.44 (m, 1H), 6.59 (d, *J* = 9.0 Hz, 1H), 6.34 (d, *J* = 4.7 Hz, 1H), 3.84 (s, 3H), 3.26 (t, *J* = 6.5 Hz, 2H), 1.79 – 1.52 (m, 4H), 1.19 (d, *J* = 6.6 Hz, 3H).

^{13}C NMR (126 MHz, DMSO) δ 159.7, 143.6, 142.4, 138.5, 130.4, 122.2, 98.3, 92.9, 55.3, 46.7, 42.5, 31.9, 24.0, 18.2.

HRMS: Calculated for $C_{15}H_{22}N_3O^+$: 260.1757 [M+H$^+$]; found: 260.1752.

(1*S*,2*R*,4*R*)-2,3,3-trimethylbicyclo[2.2.1]heptan-2-amine

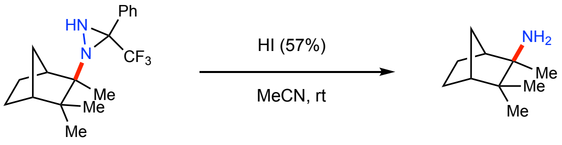

Followed our previously reported procedure. [1] To a 25 mL round bottom flask equipped with a magnetic stir bar was added **6j** (109 mg, 0.336 mmol, 1 eq) and acetonitrile (3 mL). The mixture was cooled with an ice bath, followed by slow addition of addition of hydroiodic acid (57%, 310 μL, 7 eq). Then the reaction mixture was stirred at room temperature for 1 h until TLC indicating disappearance of starting material. Sat. Na_2SO_3 (10 mL) and 1 M NaOH (5 mL) was added and stirred until the color of iodine disappeared. The mixture was then extracted with dichloromethane (20 mL x 3), washed with brine (20 mL), dried with *anhyd.* Na_2SO_4, concentrated *in vacuo* and purified by flash column chromatography (basic alumina, 10% MeOH in DCM) to afford a white solid (42.2 mg, 82% yield).

The ^{1}H NMR spectrum matched with previously reported ^{1}H NMR. [36]

Physical state: white solid. $\mathbf{R_f}$ = 0.3 (20% MeOH in DCM, vis. PMA).

^{1}H NMR (500 MHz, $CDCl_3$) δ 1.92 (dt, J = 10.4, 2.4 Hz, 1H), 1.83 – 1.78 (m, 1H), 1.71 (dd, J = 4.0, 1.8 Hz, 1H), 1.58 (ddt, J = 12.1, 9.0, 2.7 Hz, 1H), 1.51 (dddd, J = 11.1, 8.7, 4.4, 2.4 Hz, 1H), 1.40 – 1.31 (m, 1H), 1.31 – 1.16 (m, 3H), 1.06 (s, 3H), 0.97 (s, 3H), 0.90 (s, 3H).

^{13}C NMR (126 MHz, $CDCl_3$) δ 59.8, 52.5, 50.3, 42.9, 34.4, 26.3, 24.0, 23.6, 23.5, 23.2.

HRMS: Calculated for $C_{10}H_{20}N^+$: 154.1590 [M+H$^+$]; found 154.1588.

Mecamylamine HCl (10)

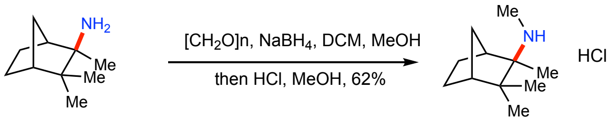

10

To a 10 mL round bottom flask equipped with a magnetic stir bar under argon was added (1*S*,2*R*,4*R*)-2,3,3-trimethylbicyclo[2.2.1]heptan-2-amine (31.0 mg, 0.202 mmol, 1 eq) and anhydrous dichloromethane (2.5 mL). Paraformaldehyde (60.5 mg, 2.02 mmol, 10 eq) and activated 4 Å MS (70 mg) was added sequentially, followed by back-flush with argon and the reaction mixture stirred under reflux overnight until crude NMR indicated the disappearance of starting materials. The reaction mixture was then cooled to 0 ºC and anhydrous methanol (1 mL) was added, followed by sodium borohydride (38.0 mg, 0.950 mmol, 5 eq) portion-wise. The reaction was then stirred at room temperature overnight before acetone (~2 mL) was added to quench the reaction. The reaction mixture was concentrated *in vacuo* and the residue purified by flash column chromatography (silica gel, ethyl acetate to 50% MeOH in ethyl acetate). To the obtained white solid was added methanol (5 mL), followed by *conc.* HCl (1 mL). The mixture was shaken for 5 min and concentrated *in vacuo*, offering mecamylamine HCl as a white solid (25.7 mg, 62% yield).

The ^{1}H NMR spectrum matched with previously reported ^{1}H NMR. [36]

Physical state: white solid. **R_f** = 0.2 (50% MeOH in ethyl acetate, vis. PMA).

^{1}H NMR (500 MHz, $CDCl_3$) δ 8.82 (s, 1H), 8.14 (s, 1H), 2.65 (s, 3H), 2.39 (s, 2H), 1.91 – 1.82 (m, 1H), 1.60 (d, J = 4.3 Hz, 1H), 1.44 (d, J = 17.1 Hz, 5H), 1.36 – 1.20 (m, 5H), 1.02 (s, 3H).

^{13}C NMR (126 MHz, $CDCl_3$) δ 70.2, 50.7, 45.2, 44.8, 35.3, 27.2, 23.7, 23.5, 23.1, 16.8.

HRMS: Calculated for $C_{11}H_{22}N^+$: 168.1747 [M+H$^+$]; found 168.1751.

Neramexane hydrochloride (12)

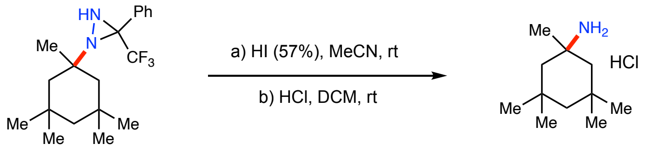

Followed our previously reported procedure. [1] To a septum capped vial equipped with a magnetic stir bar was added **3ac** (11.0 mg, 0.032 mmol, 1 eq) and acetonitrile (0.35 mL). The mixture cooled with an ice bath, followed by addition of hydroiodic acid (57%, 30 μL, 7 eq). Then the reaction mixture was stirred at room temperature for 1 h until TLC indicated disappearance of starting material. Sat. Na_2SO_3 (~10 mL) and 10% NaOH (~5 mL) were added, and the reaction stirred until the color of iodine disappeared. The mixture was then extracted with dichloromethane (20 mL x 3), washed with brine (20 mL), dried with *anhyd.* Na_2SO_4, concentrated *in vacuo* and purified by flash column chromatography (silica gel, 10% MeOH in DCM to 20% MeOH in DCM) to afford the crude product. It was redissolved in dichloromethane (~1 mL), and HCl gas was bubbled for several minutes. The mixture was concentrated *in vacuo*, washed with several drops of dichloromethane, affording neramexane hydrochloride (3.8 mg, 57% yield) as a white solid.

The ^{1}H NMR spectrum matched with previously reported ^{1}H NMR. [37]

Physical state: white solid. $\mathbf{R_f}$ = 0.4 (20% MeOH in DCM, vis. PMA).

^{1}H NMR (500 MHz, DMSO) δ 8.00 (s, 3H), 1.62 – 1.53 (m, 2H), 1.46 – 1.37 (m, 5H), 1.34 – 1.27 (m, 1H), 1.01-1.07 (m, 7H), 0.93 (s, 6H).

^{13}C NMR (126 MHz, DMSO) δ 54.5, 49.9, 46.8, 35.5, 31.1, 28.9, 26.5.

HRMS: Calculated for $C_{11}H_{24}N^+$: 170.1903 [M+H$^+$]; found 170.1905.

4-(1-(1*H*-pyrazol-1-yl)ethyl)-1-tosylpiperidine (15)

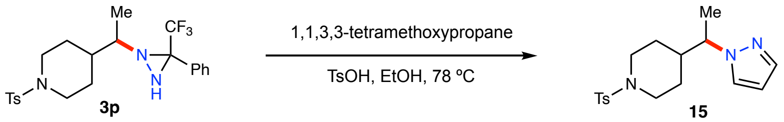

Followed our previously reported procedure. [1] To a 10 mL round bottom flask equipped with a magnetic stir bar was added **3p** (199 mg, 0.439 mmol, 1 eq) and ethanol (4 mL). 1,1,3,3-Tetramethoxypropane (210 µL, 0.878 mmol, 2 eq) was added followed by *p*-toluenesulfonic acid monohydrate (417 mg, 2.20 mmol, 5 eq). The reaction mixture was stirred for 48 h at 78 ºC. To the reaction mixture, 10% NaOH (10 mL) was added, and the mixture extracted with ethyl acetate (10 mL x 3), washed with brine (20 mL), dried over *anhyd.* Na_2SO_4, concentrated *in vacuo*. The residue was purified by flash column chromatography (silica gel, 3% ethyl acetate in DCM to 5% ethyl acetate in DCM) affording the product (94.2 mg, 64% yield) as a white solid.

Physical state: white solid. $\mathbf{R_f}$ = 0.6 (10% ethyl acetate in DCM, vis. UV and I_2).

^{1}H NMR: (500 MHz, $CDCl_3$) δ 7.65 – 7.54 (m, 2H), 7.47 (d, *J* = 2.0 Hz, 1H), 7.34 – 7.27 (m, 3H), 6.19 (t, *J* = 2.1 Hz, 1H), 4.00 (dq, *J* = 8.4, 6.9 Hz, 1H), 3.83 (ddt, *J* = 11.6, 4.7, 2.5 Hz, 1H), 3.71 (ddt, *J* = 11.6, 4.6, 2.7 Hz, 1H), 2.42 (s, 3H), 2.19 (td, *J* = 12.1, 2.7 Hz, 1H), 2.11 (td, *J* = 11.9, 3.0 Hz, 1H), 1.84 – 1.65 (m, 3H), 1.46 (d, *J* = 6.9 Hz, 3H), 1.38 (qd, *J* = 12.3, 4.3 Hz, 1H), 1.32 – 1.21 (m, 1H), 1.21 – 1.12 (m, 1H).

^{13}C NMR: (126 MHz, $CDCl_3$) δ 143.6, 139.3, 133.0, 129.7, 128.3, 127.8, 104.9, 62.0, 46.3, 41.7, 28.4, 28.2, 21.6, 18.1.

HRMS: Calculated for $C_{17}H_{24}N_3O_2S^+$: 334.1584 [M+H$^+$]; found: 334.1578.

1-(1-tosylpiperidin-4-yl)ethan-1-amine (16)

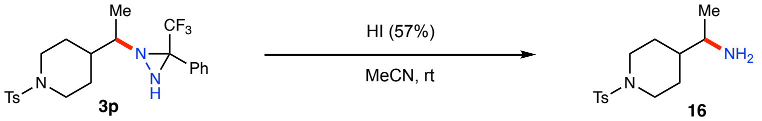

Followed our previously reported procedure. [1] To a septum capped vial equipped with a magnetic stir bar was added **3p** (31.0 mg, 0.068 mmol, 1 eq) and acetonitrile (0.7 mL). The mixture was cooled with an ice bath, followed by the addition of hydroiodic acid (57%, 65 μL, 7 eq). The reaction mixture was stirred at room temperature for 1 h until TLC indicated the disappearance of starting material. Sat. Na_2SO_3 (~10 mL) and 10% NaOH (~5 mL) were added and stirred until the color of iodine disappeared. The mixture was then extracted with dichloromethane (20 mL x 3), washed with brine (20 mL), dried with *anhyd.* Na_2SO_4, concentrated *in vacuo* and purified by flash column chromatography (basic alumina, 3% MeOH in DCM to 20% MeOH in DCM) to afford the product (14.8 mg, 77% yield) as a white solid.

Physical state: white solid. $\mathbf{R_f}$ = 0.6 (20% MeOH in DCM, vis. UV).

^{1}H NMR (500 MHz, $CDCl_3$) δ 7.71 – 7.58 (m, 2H), 7.31 (d, *J* = 8.0 Hz, 2H), 3.91 – 3.74 (m, 2H), 2.69 (t, *J* = 6.5 Hz, 1H), 2.43 (s, 3H), 2.28 – 2.12 (m, 2H), 1.87 – 1.66 (m, 2H), 1.42 – 1.14 (m, 5H), 1.01 (d, *J* = 6.4 Hz, 3H).

^{13}C NMR (126 MHz, $CDCl_3$) δ 143.5, 133.3, 129.7, 127.9, 50.7, 46.6, 46.6, 43.0, 29.8, 27.9, 27.8, 21.6, 20.9.

HRMS: Calculated for $C_{14}H_{23}N_2O_2S^+$: 283.1475 [M+H$^+$]; found: 283.1471.

4-(2-(1*H*-pyrazol-1-yl)propan-2-yl)-1-tosylpiperidine (17)

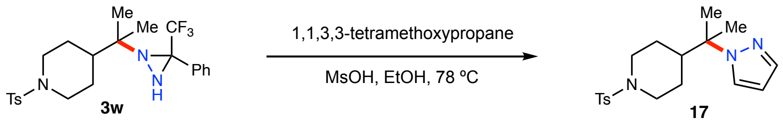

Followed our previously reported procedure. [1] To a septum capped vial equipped with a magnetic stir bar was added **3w** (39.7 mg, 0.085 mmol, 1 eq) and ethanol (1 mL). 1,1,3,3-Tetramethoxypropane (180 mg, 180 μL, 1.10 mmol, 12.9 eq) was added, followed by methanesulfonic acid (80 μL). The reaction mixture was stirred for 48 h at 78 ºC. The reaction mixture was concentrated *in vacuo* and the residue purified by flash column chromatography (silica, 20% ethyl acetate in hexane) affording the product (16.1 mg, 55% yield) as a white foam.

Physical state: white foam. $\mathbf{R_f}$ = 0.3 (30% ethyl acetate in hexanes, vis. UV).

^{1}H NMR (500 MHz, $CDCl_3$) δ 7.55 (d, *J* = 8.2 Hz, 2H), 7.44 (d, *J* = 2.0 Hz, 1H), 7.40 (d, *J* = 2.4 Hz, 1H), 7.25 (d, *J* = 8.1 Hz, 2H), 6.14 (d, *J* = 2.0 Hz, 1H), 3.79 – 3.68 (m, 2H), 2.38 (s, 3H), 2.07 (td, *J* = 11.9, 2.7 Hz, 2H), 1.85 (tt, *J* = 12.2, 3.4 Hz, 1H), 1.46 (s, 6H), 1.33 (qd, *J* = 12.5, 4.1 Hz, 2H), 1.26 – 1.16 (m, 3H).

^{13}C NMR (126 MHz, $CDCl_3$) δ 143.5, 139.0, 132.7, 129.6, 127.7, 126.6, 104.4, 62.7, 46.7, 46.0, 26.1, 24.4, 21.5.

HRMS: Calculated for $C_{18}H_{26}N_3O_2S^+$: 348.1740 [M+H$^+$]; found 348.1744.

2-(1-tosylpiperidin-4-yl)propan-2-amine (18)

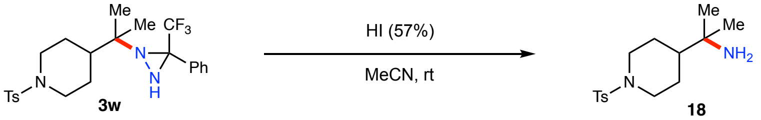

Followed our previously reported procedure. [1] To a septum capped vial equipped with a magnetic stir bar was added **3w** (39.6 mg, 0.085 mmol, 1 eq) and acetonitrile (1 mL). The mixture was cooled with an ice bath, followed by addition of hydroiodic acid (57%, 133 μL, 7 eq). Then the reaction mixture was stirred at room temperature for 1 h until TLC indicated disappearance of starting material. Sat. Na_2SO_3 (10 mL) and 10% NaOH (5 mL) were added and stirred until the color of iodine disappeared. The mixture was then extracted with dichloromethane (20 mL x 3), washed with brine (20 mL), dried with *anhyd.* Na_2SO_4, concentrated *in vacuo* and purified by flash column chromatography (silica gel, 10% MeOH in DCM, with 1% NH_4OH) to afford the product (17.9 mg, 71% yield) as a white foam.

Physical state: white foam. $\mathbf{R_f}$ = 0.2 (10% MeOH in DCM plus 1% NH_4OH, vis. UV).

^{1}H NMR (500 MHz, $CDCl_3$) δ 7.63 – 7.52 (m, 2H), 7.27 (d, *J* = 8.1 Hz, 2H), 3.81 (dq, *J* = 11.6, 2.4 Hz, 2H), 2.37 (s, 3H), 2.11 (td, *J* = 12.0, 2.4 Hz, 2H), 1.77 – 1.67 (m, 2H), 1.37 (qd, *J* = 12.5, 4.3 Hz, 2H), 1.23 – 1.08 (m, 2H), 1.04 (s, 6H).

^{13}C NMR (126 MHz, $CDCl_3$) δ 143.6, 133.0, 129.7, 127.8, 52.9, 46.6, 45.9, 26.5, 26.1, 21.5.

HRMS: Calculated for $C_{15}H_{24}N_2O_2S$ 297.1637. [$M+H^+$]; found 297.1634.

6-chloro-2,8-dimethylimidazo[1,2-*b*]pyridazine

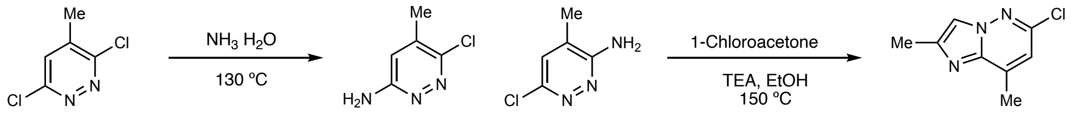

Followed a reported procedure.[38] To a 150 mL sealed tube equipped with a magnetic stir bar was added 3,6-dichloro-4-methylpyridazine (4.00 g, 36.8 mmol, 1 eq) and 25% aqueous ammonia (40 mL). The tube was sealed and stirred at 130 ºC for 24 h. The sealed tube was cooled to room temperature where a cloudy mixture formed. The precipitate was collected by filtration, washed thoroughly with water, and dried on vacuum to give a mixture of 6-chloro-5-methylpyridazin-3-amine and 6-chloro-4-methylpyridazin-3-amine (2.13 g, 61% yield in total, 1:0.85 by NMR) as white solid that was used without further purification.

The ^{1}H NMR spectrum matched with previously reported ^{1}H NMR.[38]

Physical state: white solid. **R_f** = 0.6 (ethyl acetate, vis. UV).

^{1}H NMR: major isomer (500 MHz, $CDCl_3$) δ 7.10 (d, *J* = 0.9 Hz, 1H), 4.76 (d, *J* = 6.3 Hz, 2H), 2.17 (d, *J* = 1.2 Hz, 3H).

minor isomer (500 MHz, $CDCl_3$) δ 6.63 (q, *J* = 1.1 Hz, 1H), 4.68 (s, 2H), 2.30 (d, *J* = 1.2 Hz, 3H).

Followed a reported procedure.[39] To a 150 mL sealed tube equipped with a magnetic stir bar was added mixture of 6-chloro-5-methylpyridazin-3-amine and 6-chloro-4-methylpyridazin-3-amine (2.13 g, 13.1 mmol, 1 eq), 1-chloroacetone (2.42 g, 2.10 mL, 2 eq), triethylamine (3.97 g, 5.51 mL, 3 eq) and ethanol (24 mL). The reaction mixture was stirred in a sealed tube at 150 ºC overnight. The sealed tube was cooled to room temperature, the reaction concentrated *in vacuo* and the residue purified by flash column chromatography (silica, 50% ethyl acetate in hexanes to 80% ethyl acetate in hexanes) to give 6-chloro-2,8-dimethylimidazo[1,2-*b*]pyridazine (607 mg, 49% yield based on 6-chloro-4-methylpyridazin-3-amine) as a white solid.

The ^{1}H NMR spectrum matched with previously reported ^{1}H NMR.[39]

Physical state: white solid. **R_f** = 0.7 (ethyl acetate, vis. UV).

^{1}H NMR: (500 MHz, $CDCl_3$) δ 7.66 (d, *J* = 0.9 Hz, 1H), 6.84 (q, *J* = 1.1 Hz, 1H), 2.63 (d, *J* = 1.1 Hz, 3H), 2.49 (d, *J* = 0.9 Hz, 3H).

5-bromo-3-hydroxyisobenzofuran-1(3*H*)-one

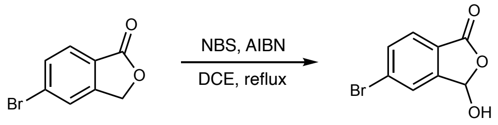

Followed a reported procedure.[40] To a 100 mL round bottom flask equipped with a magnetic stir bar under argon was added 5-bromophthalide (2.00 g, 9.4 mmol, 1 eq) and dichloroethane (45 mL). *N*-Bromosuccinimide (1.84 g, 10.3 mmol, 1.1 eq) was added, followed by AIBN (77.2 mg, 0.47 mmol, 0.05 eq). Argon was back-flushed and the reaction mixture stirred under reflux for 2 h. The reaction mixture was cooled to -20 ºC overnight, the white precipitate removed by filtration, and the filtrate was concentrated *in vacuo*. Water (20 mL) was added, and the suspension stirred under reflux for 2 h, followed by cooling to 4 ºC overnight. The white precipitate was collected by filtration, washed with cold water (5 mL x 2) and dried *in vacuo* overnight affording a white solid (1.86 g, 83% yield).

The ^{1}H NMR spectrum matched with previously reported ^{1}H NMR.[40]

Physical state: white solid.

^{1}H NMR: lactone form (500 MHz, DMSO) δ 8.29 (d, *J* = 8.7 Hz, 1H), 7.93 (d, *J* = 1.8 Hz, 1H), 7.86 (dd, *J* = 8.1, 1.7 Hz, 1H), 7.76 (d, *J* = 8.1 Hz, 1H), 6.65 (d, *J* = 7.3 Hz, 1H).

aldehyde form (500 MHz, DMSO) δ 10.43 (s, 1H), 7.96 (d, *J* = 1.1 Hz, 1H), 7.88 (s, 1H), 7.78 (d, *J* = 1.2 Hz, 1H), 5.40 (d, *J* = 0.9 Hz, 1H).

5-bromo-3-methoxyisobenzofuran-1(3*H*)-one

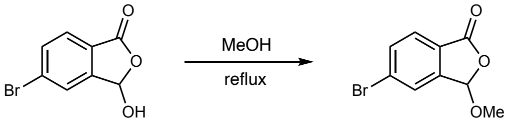

To a 10 mL round bottom flask equipped with a magnetic stir bar was added 5-bromo-3-hydroxyisobenzofuran-1(3*H*)-one (700 mg, 3.06 mmol, 1 eq) and methanol (5 mL). The reaction mixture was stirred under reflux overnight, then concentrated *in vacuo*. To the residue was added hexane (10 mL). The mixture was stirred overnight and the solid collected by filtration to afford a white solid (613 mg, 83% yield) that was used without further purification.

Physical state: white solid. $\mathbf{R_f}$ = 0.6 (25% ethyl acetate in hexane, vis. UV).

^{1}H NMR (500 MHz, $CDCl_3$) δ 7.75 (s, 3H), 6.26 (s, 1H), 3.65 (s, 3H).

^{13}C NMR (126 MHz, $CDCl_3$) δ 167.7, 146.5, 134.6, 129.8, 127.2, 126.9, 126.3, 102.5, 57.3.

HRMS: Calculated for $C_9H_7BrNaO_3^+$: 264.9471 [M+Na$^+$]; found: 264.9473.

3-methoxy-5-(4,4,5,5-tetramethyl-1,3,2-dioxaborolan-2-yl)isobenzofuran-1(3*H*)-one

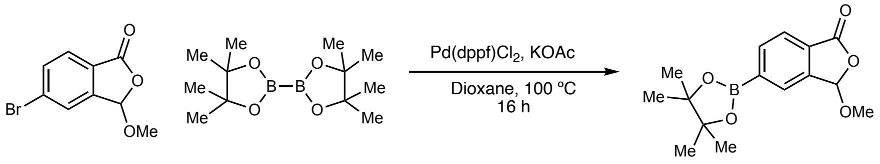

To a 50 mL round bottom flask equipped with a magnetic stir bar under argon was added 5-bromo-3-methoxyisobenzofuran-1(3*H*)-one (360 mg, 1.48 mmol, 1 eq) and dioxane (15 mL). Bis(pinacolato)diboron (451 mg, 1.78 mmol, 1.2 eq) and potassium acetate (450 mg, 4.58 mmol, 3 eq) were added and the mixture degassed with argon for 10 minutes, followed by addition of Pd(dppf)Cl_2 (100 mg, 0.148 mmol, 10 mol%). The reaction mixture was back-flushed with argon and stirred for 16 h at 100 ºC until TLC indicated total conversion of starting materials. The reaction mixture was concentrated *in vacuo*, and the residue purified by flash column chromatography (silica, 10% ethyl acetate in hexanes) to afford a white solid (246 mg, 57% yield).

Physical state: white solid. $\mathbf{R_f}$ = 0.5 (25% ethyl acetate in hexane, vis. UV).

^{1}H NMR (500 MHz, $CDCl_3$) δ 8.04 – 7.98 (m, 2H), 7.85 (d, J = 7.8 Hz, 1H), 6.29 (s, 1H), 3.61 (s, 3H), 1.35 (s, 12H).

^{13}C NMR (126 MHz, $CDCl_3$) δ 168.8, 143.9, 137.1, 129.7, 129.4, 124.6, 103.5, 84.7, 83.6, 56.9, 25.0, 25.0.

HRMS: Calculated for $C_{15}H_{20}BO_5^+$: 291.1398 [M+H$^+$]; found: 291.1389.

Methyl 2-(dimethoxymethyl)-4-(2,8-dimethylimidazo[1,2-*b*]pyridazin-6-yl)benzoate (21)

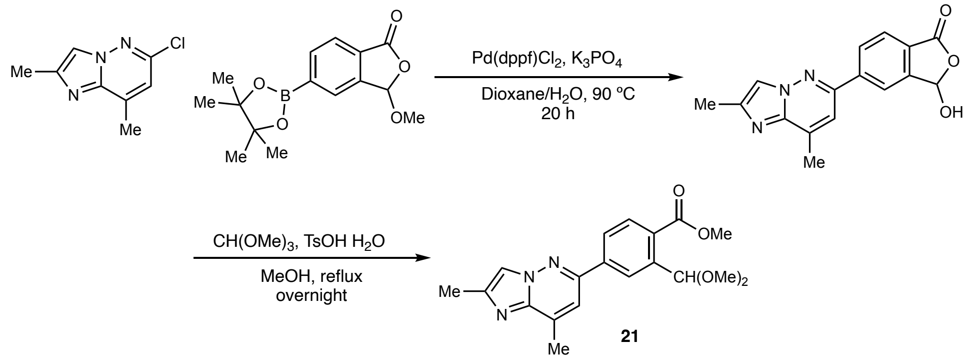

To a 25 mL round bottom flask equipped with a magnetic stir bar under argon was added 6-chloro-2,8-dimethylimidazo[1,2-*b*]pyridazine (200 mg, 1.10 mmol, 1 eq), dioxane (8 mL) and water (1.6 mL). 3-Methoxy-5-(4,4,5,5-tetramethyl-1,3,2-dioxaborolan-2-yl)isobenzofuran-1(3*H*)-one (479 mg, 1.65 mmol, 1.5 eq) was added, followed by potassium phosphate tribasic (704 mg, 3.32 mmol, 3 eq). The reaction mixture was degassed with argon for 10 minutes, followed by addition of Pd(dppf)Cl_2 (81.0 mg, 0.111 mmol, 10 mol%). The reaction mixture was back-flushed with argon and stirred overnight at 90 ºC until TLC indicated total conversion of starting materials. The reaction mixture was passed through celite, washed with methanol and concentrated *in vacuo.* To the residue, methanol (10 mL) was added, followed by trimethyl orthoformate (1.16 g, 1.21 mL, 11.0 mmol, 10 eq) and *p*-toluenesulfonic acid monohydrate (1.05 g, 5.50 mmol, 5 eq) and stirred under reflux overnight. The reaction mixture was poured into a separatory funnel, 10% NaOH (50 mL) was added and extracted with ethyl acetate (30 mL x 3), washed with brine (50 mL), dried over *anhyd.* Na_2SO_4 and concentrated *in vacuo*. The residue was purified by flash column chromatography (silica gel, 80% ethyl acetate in hexanes) to afford a white solid (243 mg, 62% yield over two steps).

Physical state: white solid. $\mathbf{R_f}$ = 0.4 (ethyl acetate, vis. UV).

^{1}H NMR (500 MHz, $CDCl_3$) δ 8.28 (d, *J* = 2.0 Hz, 1H), 8.00 (dd, *J* = 8.2, 1.9 Hz, 1H), 7.93 (d, *J* = 8.1 Hz, 1H), 7.78 (d, *J* = 0.9 Hz, 1H), 7.31 (d, *J* = 1.2 Hz, 1H), 6.15 (s, 1H), 3.95 (s, 3H), 3.43 (s, 6H), 2.72 (d, *J* = 1.1 Hz, 3H), 2.54 (d, *J* = 0.9 Hz, 3H).

^{13}C NMR (126 MHz, $CDCl_3$) δ 167.8, 149.9, 143.5, 139.6, 139.2, 139.0, 136.0, 130.8, 130.6, 126.6, 125.5, 115.0, 114.7, 100.6, 54.2, 52.4, 16.9, 14.8.

HRMS: Calculated for $C_{19}H_{22}N_3O_4^+$: 356.1605 [M+H$^+$]; found: 356.1607.

2-(azepan-4-yl)-6-(2,8-dimethylimidazo[1,2-*b*]pyridazin-6-yl)phthalazin-1(2*H*)-one (25)

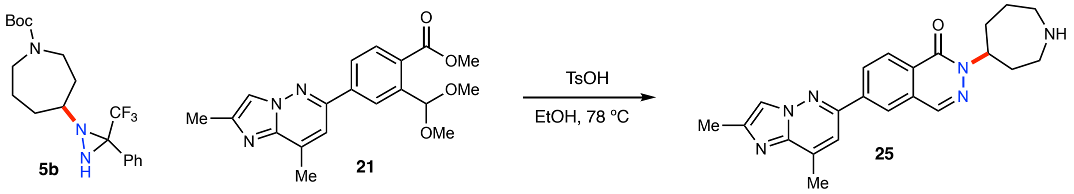

To a 10 mL round bottom flask equipped with a magnetic stir bar under argon was added *tert*-butyl 4-(3-phenyl-3-(trifluoromethyl)diaziridin-1-yl)azepane-1-carboxylate (86.0 mg, 0.223 mmol, 1 eq) and ethanol (3 mL). Methyl 2-(dimethoxymethyl)-4-(2,8-dimethylimidazo[1,2-*b*]pyridazin-6-yl)benzoate (87.2 mg, 0.245 mmol, 1.1 eq) and *p*-toluenesulfonic acid monohydrate (255 mg, 1.32 mmol, 6 eq) were added and the reaction mixture stirred at 78 ºC for 24 h, until LC-MS indicated the disappearance of starting materials. The reaction mixture was poured into separatory funnel, 10% NaOH (50 mL) was added, extracted with ethyl acetate (30 mL x 3), washed with brine (50 mL), dried over *anhyd.* Na_2SO_4 and concentrated *in vacuo*. The residue was purified by flash column chromatography (basic alumina, 5% MeOH in DCM to 10% MeOH in DCM) to afford **25** as a pale yellow solid (55.8 mg, 64% yield).

The ^{1}H NMR spectrum matched with previously reported ^{1}H NMR.[41]

Physical state: pale yellow solid. $\mathbf{R_f}$ = 0.4 (20% MeOH in DCM, vis. UV).

^{1}H NMR (500 MHz, DMSO) δ 8.53 (s, 2H), 8.46 – 8.27 (m, 2H), 8.06 (s, 1H), 7.72 (s, 1H), 5.32 – 4.97 (m, 1H), 2.90 (dt, *J* = 10.7, 5.7 Hz, 2H), 2.86 – 2.66 (m, 2H), 2.60 (s, 3H), 2.40 (s, 3H), 2.14 – 1.90 (m, 3H), 1.91 – 1.71 (m, 2H), 1.60 (dt, *J* = 10.4, 5.4 Hz, 1H).

^{13}C NMR (126 MHz, DMSO) δ 157.2, 148.4, 143.4, 139.6, 138.5, 137.8, 136.1, 129.5, 129.3, 127.3, 126.8, 124.8, 114.6, 114.6, 56.4, 48.7, 45.3, 36.0, 32.5, 27.5, 16.3, 14.6.

HRMS: Calculated for $C_{22}H_{25}N_6O^+$: 389.2084 [M+H$^+$]; found: 389.2090.

2-(azepan-4-yl)-6-bromophthalazin-1(2*H*)-one 4-methylbenzenesulfonate (23)

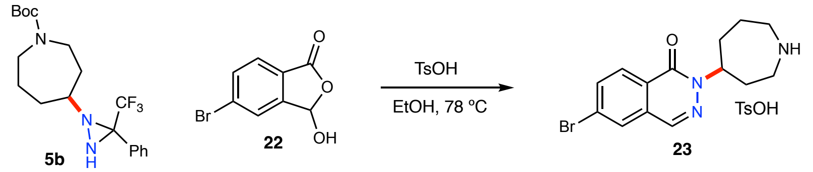

To a 5 mL round bottom flask equipped with a magnetic stir bar was added *tert*-butyl 4-(3-phenyl-3-(trifluoromethyl)diaziridin-1-yl)azepane-1-carboxylate (34.0 mg, 0.088 mmol, 1 eq) and ethanol (0.75 mL). 5-Bromo-3-hydroxyisobenzofuran-1(3*H*)-one (22.2 mg, 0.097 mmol, 1.1 eq) and *p*-toluenesulfonic acid monohydrate (33.6 mg, 0.176 mmol, 2 eq) were added and the reaction mixture stirred at 78 ºC for 24 h, until LC-MS indicated the disappearance of starting materials. Around half of the ethanol was concentrated *in vacuo* and the mixture kept at -20 ºC overnight. The solid that formed was collected by filtration and dried *in vacuo*, affording **23** (38.7 mg, 89% yield) as a white solid.

Physical state: white solid.

^{1}H NMR (500 MHz, DMSO) δ 8.58 (s, 2H), 8.46 (s, 1H), 8.25 (d, *J* = 2.0 Hz, 1H), 8.17 (d, *J* = 8.5 Hz, 1H), 8.03 (dd, *J* = 8.5, 2.0 Hz, 1H), 7.52 – 7.41 (m, 2H), 7.16 – 7.04 (m, 2H), 5.19 (tt, *J* = 9.6, 5.0 Hz, 1H), 3.43 – 3.34 (m, 1H), 3.29 – 3.18 (m, 2H), 3.14 (s, 1H), 2.28 (s, 3H), 2.27 – 2.19 (m, 1H), 2.17 – 2.08 (m, 1H), 2.08 – 1.93 (m, 3H), 1.92 – 1.76 (m, 1H).

^{13}C NMR (126 MHz, DMSO) δ 157.7, 146.3, 138.0, 137.4, 135.5, 130.9, 129.7, 128.9, 128.5, 127.8, 126.4, 126.0, 55.4, 46.2, 42.3, 31.8, 30.1, 21.7, 21.2.

HRMS: Calculated for $C_{14}H_{17}BrN_3O^+$: 322.0550 [M+H$^+$]; found: 322.0557.

(2,8-dimethylimidazo[1,2-*b*]pyridazin-6-yl)boronic acid (24)

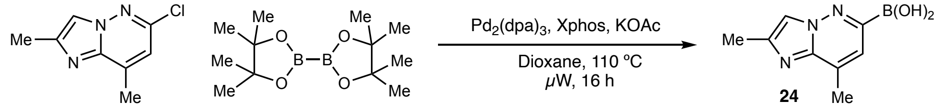

To a septum capped microwave reaction vial equipped with a magnetic stir bar under argon was added 6-chloro-2,8-dimethylimidazo[1,2-*b*]pyridazine (80.0 mg, 0.440 mmol, 1 eq), bis(pinacolato)diboron (168 mg, 0.661 mmol, 1.5 eq), dioxane (3 mL), potassium acetate (130 mg, 1.32 mmol, 3 eq) and Xphos (68.2 mg, 0.143 mmol, 0.3 eq) sequentially. The mixture was degassed with argon for 10 minutes, then $Pd_2(dpa)_3$ (68.4 mg, 0.065 mmol, 0.15 eq) was added, the reaction vial filled with argon, and stirred in a microwave reactor for 16 h at 110 ºC. The reaction vial cooled to room temperature, passed through celite, washed with ethyl acetate and concentrated *in vacuo*. The residue was used directly without further purification.

2-(azepan-4-yl)-6-(2,8-dimethylimidazo[1,2-*b*]pyridazin-6-yl)phthalazin-1(2*H*)-one (25)

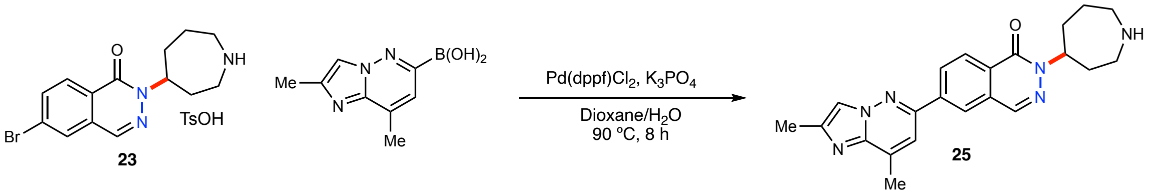

To a 25 mL round bottom flask equipped with a magnetic stir bar under argon was added 2-(azepan-4-yl)-6-bromophthalazin-1(2*H*)-one 4-methylbenzenesulfonate (**23**) (18.6 mg, 0.038 mmol, 1 eq), dioxane (1 mL) and water (0.2 mL). (2,8-Dimethylimidazo[1,2-*b*]pyridazin-6-yl)boronic acid (10.7 mg, 0.056 mmol, 1.5 eq) was added, followed by potassium phosphate tribasic (32.3 mg, 0.152 mmol, 4 eq). The reaction mixture was degassed with argon for 10 minutes, followed by addition of Pd(dppf)Cl_2 (2.8 mg, 0.004 mmol, 10 mol%). The reaction mixture was back-flushed with argon and stirred for 8 h at 90 ºC, until TLC indicated total conversion of starting materials. The reaction mixture was poured into a separatory funnel, 10% NaOH (10 mL) was added. The mixture was extracted with ethyl acetate (10 mL x 3), washed with brine (20 mL), dried over *anhyd.* Na_2SO_4 and concentrated *in vacuo*. The residue was purified by flash column chromatography (basic alumina, 5% MeOH in DCM to 10% MeOH in DCM) to afford the product (11.3 mg, 77% yield) as a pale yellow solid.

Physical state: pale yellow solid. **R_f** = 0.4 (20% MeOH in DCM, vis. UV).

Characterization data matched with **25**.

Synthesis of ^{15}N Diazirine:

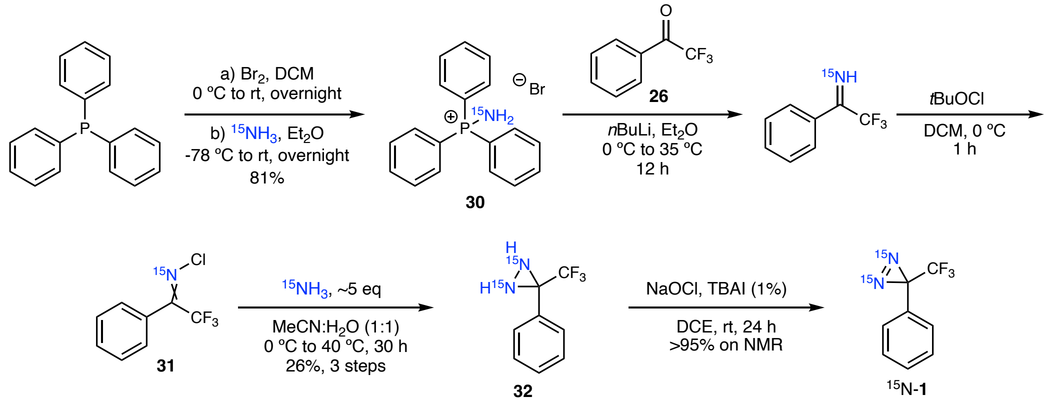

Supplementary Figure 12: Synthetic route towards ^{15}N Diazirine (^{15}N-**1**).

(Amino-^{15}N)triphenylphosphonium bromide (30)

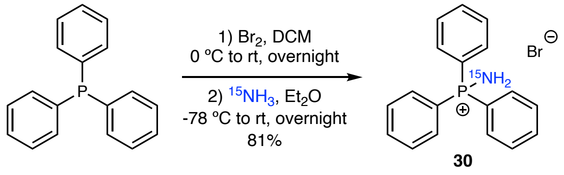

To a flame-dried 50 mL sealed tube fitted with a rubber stopper was added triphenylphosphine (2.00 g, 7.62 mmol, 1 eq) and anhydrous dichloromethane (10 mL). The tube was back-flushed with argon, cooled to 0 ºC, and bromine (1.24 g, 0.40 mL, 7.77 mmol, 1.02 eq) was added via syringe with stirring. The formation of a precipitate was observed during the addition. The mixture was warmed to room temperature after addition and stirred overnight.

Dichloromethane was removed under vacuum. Anhydrous ether (15 mL) was added via syringe and the reaction mixture was sonicated until it became a suspension. The mixture was cooled to -78 ºC and ^{15}N-ammonia (~1 g) was bubbled into the mixture with a long needle placed at the bottom of the sealed tube (no stirring) (Supplementary Figure 13). After bubbling, the tube was sealed with a cap (wrapped with parafilm and further taped on the outside) and stirred at room temperature overnight.

(Note: the volume of ammonia gas was estimated with an oil bubbler, which was calibrated with unlabeled ammonia with a similar tank. By bubbling the unlabeled ammonia gas through the bubbler, and counting the bubbles per minute (adjusted to be 1 bubble per second); the rate was kept constant for an hour and the tank was weighed before and after to estimate consumption. This experiment was repeated several times to establish precision and used to adjust the rate when bubbling the labeled ammonia in order to control the volume of gas in the system with a timer.)

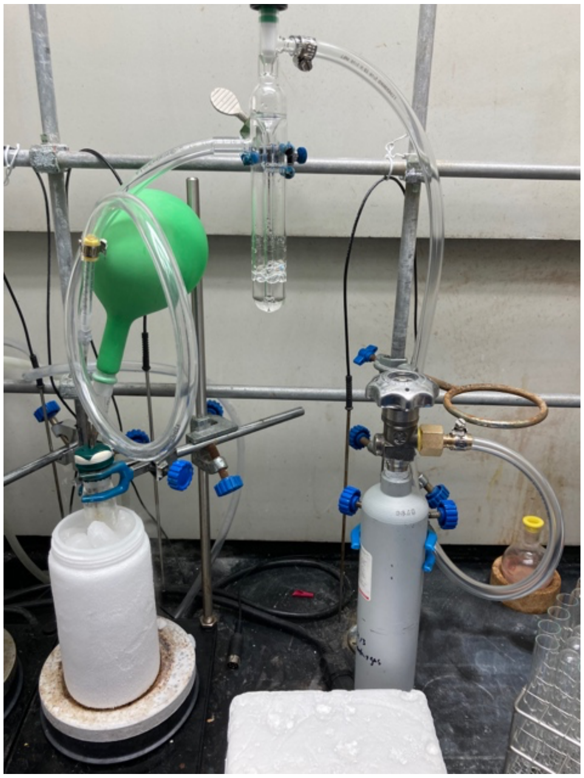

Supplementary Figure 13: Bubbling $^{15}NH_3$ gas into the PPh_3Br_2 suspension with oil bubbler as a flowmeter.

(Note: color change from orange to white observed after reaction completion.)

The reaction mixture was cooled to -78 ºC, opened, and warmed up to room temperature slowly. Ether was then removed under vacuum and chloroform (10 mL) added. The resulting mixture was stirred for an hour, filtered, and the cake washed with chloroform (5 mL). To the filtrate, ether (100 ml) was added with stirring and the formed solid was collected with vacuum filtration, washed with ether (10 mL), affording **30** (2.29 g, 81% yield) as a white solid.

Physical state: White solid. **Melting point:** 241.2 ºC – 241.6 ºC.

^{1}H NMR: (600 MHz, $CDCl_3$) δ 7.95 – 7.78 (m, 6H), 7.68 (dddd, *J* = 8.8, 7.1, 2.9, 1.3 Hz, 3H), 7.61 – 7.52 (m, 6H), 6.87 (d, *J* = 3.5 Hz, 1H), 6.73 (d, *J* = 3.5 Hz, 1H).

^{13}C NMR: (151 MHz, $CDCl_3$) δ 134.4 (d, *J* = 2.7 Hz), 133.5 (d, *J* = 11.4 Hz), 129.7 (d, *J* = 13.6 Hz), 123.5 (d, *J* = 104.1 Hz).

^{31}P NMR: (243 MHz, $CDCl_3$) δ 36.00.

HRMS: Calculated for $C_{18}H_{17}{}^{15}NP^{+}$ 279.1063; found 279.1070.

2,2,2-trifluoro-1-phenylethan-1-imine-^{15}N

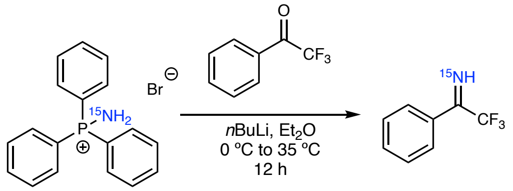

To a flame-dried 25 mL round bottom flask equipped with a magnetic stir bar under argon was added **30** (2.23 g, 6.21 mmol, 1.1 eq) and anhydrous ether (15 mL). Freshly titrated *n*BuLi (1.7 M, 3.8 mL, 6.49 mmol, 1.15 eq) was added at 0 ºC dropwise. An orange color developed after addition. The reaction mixture was stirred at room temperature for 15 min, followed by addition of 2,2,2-trifluoro-1-phenylethan-1-one (983 mg, 5.64 mmol, 1 eq) in one portion and stirred at 35 ºC overnight. Ether was removed under vacuum with a needle and the residue fitted with a micro distillation system and distilled with an oil pump. The colorless oil was collected and quickly sealed under an argon atmosphere and used without further purification.

(Note: the product is not very stable to air; slow decomposition is observed when exposed; storage in sealed vial is recommended)

N-chloro-2,2,2-trifluoro-1-phenylethan-1-imine-^{15}N (31)

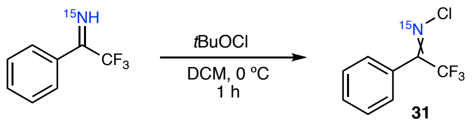

To a 50 mL flame-dried flask equipped with magnetic stir bar under argon was added imine from last step (983 mg, theoretical yield from last step) and dichloromethane (10 mL) and the flask back-flushed with argon. To the resulting mixture, freshly prepared *t*BuOCl (674 mg, 703 µL, 6.21 mmol, 1.1 eq) in dichloromethane (10 mL) was added dropwise at 0 ºC and the reaction mixture stirred at 0 ºC for 1 h, until crude ^{19}F NMR indicated total conversion of the starting material. The reaction mixture was concentrated *in vacuo* and passed through a short column (silica gel, 3% ethyl acetate in hexanes) yielding **31** (1.20 g, crude weight, the compound is volatile and unstable), which was used directly in the next step without further purification.

Physical state: colorless oil. $\mathbf{R_f}$ = 0.6 (10% ethyl acetate in hexanes, vis. UV).

^{1}H NMR (500 MHz, $CDCl_3$, crude) δ 7.70 – 7.52 (m, 3H), 7.53 – 7.37 (m, 2H).

^{13}C NMR: (126 MHz, $CDCl_3$, crude) δ 131.4, 128.9, 128.6, 127.3.

^{19}F NMR: (471 MHz, $CDCl_3$, crude) δ -63.55 (d, J = 3.5 Hz), -68.01 (d, J = 5.2 Hz).

3-phenyl-3-(trifluoromethyl)diaziridine-1,2-$^{15}N_2$ (32)

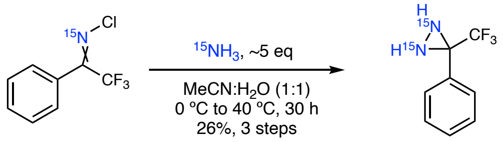

To a 10 mL flame-dried sealed tube equipped with a stir bar and rubber stopper was added **31** (1.20 g, crude), acetonitrile (1 mL) and water (1 mL). ^{15}N-ammonia gas (~0.5 g) was bubbled into the reaction mixture via a long needle at 0 ºC. The tube was then quickly sealed and stirred at 40 ºC for 30 h.

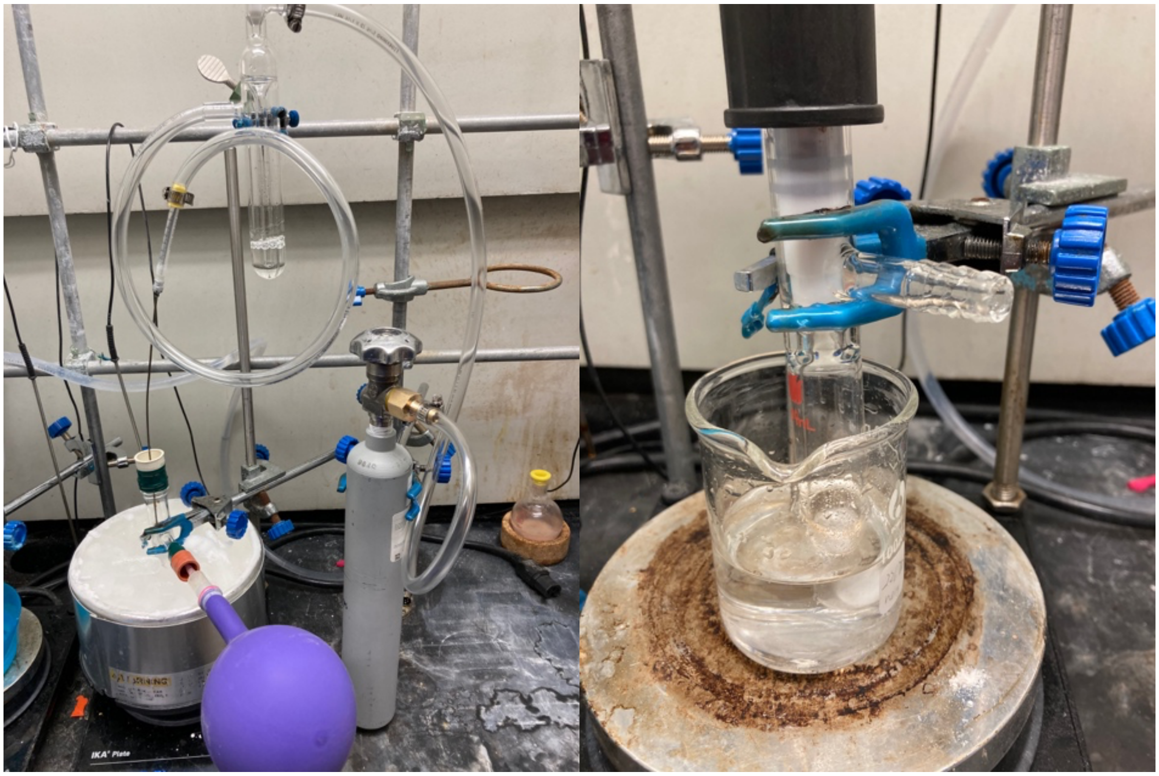

Supplementary Figure 14: Left: Bubbling $^{15}NH_3$ gas into a solution of **31** in ether with an oil bubbler as a flowmeter; **Right:** The lid was fully sealed after required amount of $^{15}NH_3$ was bubbled into solution and heated with an oil bath.

The reaction mixture was poured into water (10 mL) and extracted with ether (15 mL x 3), washed with brine, dried over *anhydrous* Na_2SO_4, and concentrated *in vacuo*. The residue was purified via flash column chromatography (silica gel, 3 –10 % ethyl acetate in hexanes) yielding **32** (252 mg, 26% yield over 3 steps).

Physical state: white solid. $\mathbf{R_f}$ = 0.2 (10% ethyl acetate in hexanes, UV).

^{1}**H NMR:** (500 MHz, $CDCl_3$) δ 7.65 (dd, *J* = 7.8, 2.0 Hz, 2H), 7.52 – 7.40 (m, 3H), 3.01 – 2.67 (m, 1H), 2.41 – 2.13 (m, 1H).

^{13}**C NMR:** (126 MHz, $CDCl_3$) δ 131.7, 131.7, 130.2, 128.8, 128.1, 123.5 (d, *J* = 272.9 Hz), 58.0 (d, *J* = 35.9 Hz).

^{19}**F NMR:** (471 MHz, $CDCl_3$) δ -75.58.

HRMS: Calculated for $C_8H_8F_3{}^{15}N_2^+$, 191.0575 [M+H$^+$]; found 191.0577.

tert-butyl (4_S_)-4-(3-phenyl-3-(trifluoromethyl)diaziridin-1-yl-1,2-$^{15}N_2$)azepane-1-carboxylate (33)

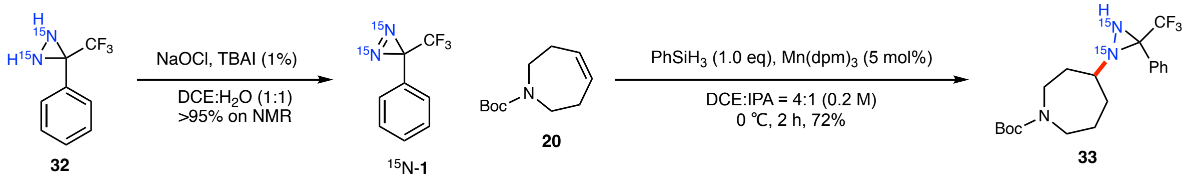

To a septum capped vial equipped with a magnetic stir bar was added 3-phenyl-3-(trifluoromethyl)diaziridine-1,2-$^{15}N_2$ (**32**) (30.0 mg, 0.157 mmol, 1.6 eq) and dichloroethane (0.5 mL). Tetrabutylammonium iodide (0.4 mg, 1 mol%) was added, followed by bleach (200 µL) at 0 ºC. The reaction mixture was stirred at room temperature overnight. On the second day, another portion of bleach (100 µL) was added and the reaction mixture stirred for another 12 h at room temperature, until ^{19}F NMR indicated total conversion of starting materials. The organic layer was washed with water (100 µL), then *sat.* Na_2SO_3 (100 µL) and brine (100 µL), and dried over a short pipette of *anhyd.* Na_2SO_4 into a flame-dried reaction vial. To the septum capped vial containing the reaction mixture above was added freshly dried isopropyl alcohol (100 µL), *tert*-butyl-2,3,6,7-tetrahydro-1*H*-azepine-1-carboxylate (**20**) (19.7 mg, 0.100 mmol, 1 eq), and Mn(dpm)$_3$ (3.0 mg, 5 mol%). The vial was back-flushed with argon, cooled to 0 ºC and $PhSiH_3$ (12.3 µL, 1 eq) was added with syringe and stirred at that temperature for 2 h. The reaction mixture was concentrated *in vacuo* and the residue purified by flash column chromatography (silica gel, 10% ethyl acetate in hexanes) to afford product **33** (28.0 mg, 72% yield) as a pale-yellow oil.

Physical state: pale yellow foam. **R_f** = 0.3 (10% ethyl acetate in hexanes, vis. iodine).

^{1}H NMR (500 MHz, $CDCl_3$, mixture of isomers) δ 7.76 – 7.57 (m, 2H), 7.53 – 7.30 (m, 3H), 3.58 – 2.87 (m, 5H), 2.02 – 1.48 (m, 6H, merged with water peak), 1.42 – 1.31 (m, 9H), 1.08 – 0.92 (m, 1H).

^{13}C NMR (126 MHz, $CDCl_3$, mixture of isomers) δ 155.4, 155.3, 155.3, 130.3, 130.2, 130.1, 128.5, 128.5, 128.5, 128.2, 128.1, 128.1, 123.8 (d, J = 274.3 Hz), 79.3, 79.2, 79.2, 79.2, 64.0 (d, J = 36.3 Hz), 61.2, 61.1, 61.0, 60.9, 60.7, 47.0, 46.7, 46.4, 45.8, 44.3, 44.3, 43.7, 43.7, 43.2, 43.2, 42.6, 34.4, 34.3, 34.0, 32.6, 32.6, 32.5, 31.9, 31.9, 31.8, 31.8, 30.7, 30.3, 30.3, 29.8, 28.6, 28.6, 28.5, 25.1, 25.1, 24.9, 24.3, 24.3, 23.9, 23.8.

^{19}F NMR (471 MHz, $CDCl_3$, mixture of isomers) δ -74.04, -74.09, -74.18, -74.19.

HRMS: Calculated for $C_{19}H_{27}F_3N^{15}N_2O_2^+$ 388.1991. [M+H$^+$]; found 388.1994.

2-(azepan-4-yl)-6-(2,8-dimethylimidazo[1,2-*b*]pyridazin-6-yl)phthalazin-1(2*H*)-one-$^{15}N_2$ (^{15}*N*-25)

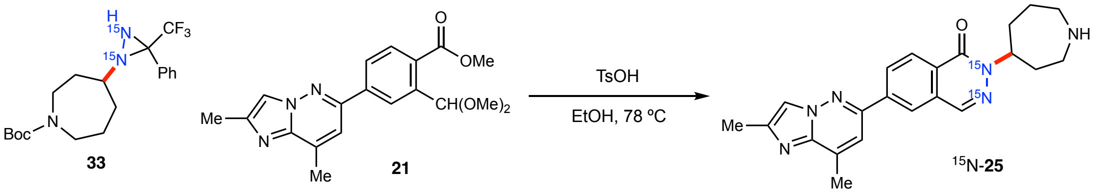

To a septum capped vial equipped with a magnetic stir bar was added *tert*-butyl 4-(3-phenyl-3-(trifluoromethyl)diaziridin-1-yl-1,2-$^{15}N_2$)azepane-1-carboxylate (**33**) (28.0 mg, 0.072 mmol, 1 eq) and ethanol (1 mL). Methyl 2-(dimethoxymethyl)-4-(2,8-dimethylimidazo[1,2-*b*]pyridazin-6-yl)benzoate (**21**) (30.9 mg, 0.087 mmol, 1.2 eq) and *p*-toluenesulfonic acid monohydrate (82.2 mg, 0.432 mmol, 6 eq) were added and the reaction mixture stirred at 78 ºC for 24 h, until LC-MS indicated the disappearance of starting materials. The reaction mixture was poured into a separatory funnel, 10% NaOH (20 mL) was added and the micture extracted with ethyl acetate (30 mL x 3), washed with brine (50 mL), dried over *anhyd.* Na_2SO_4 and concentrated *in vacuo*. The residue was purified by flash column chromatography (basic alumina, 5% MeOH in DCM to 10% MeOH in DCM) to afford product **^{15}*N*-25** as a pale yellow solid (23.1 mg, 75% yield).

Physical state: pale yellow solid. **R_f** = 0.4 (20% MeOH in DCM, vis. UV).

^{1}H NMR (500 MHz, DMSO) δ 8.66 – 8.52 (m, 2H), 8.48 (dd, *J* = 8.5, 1.9 Hz, 1H), 8.38 (d, *J* = 8.4 Hz, 1H), 8.11 (s, 1H), 7.78 (s, 1H), 5.19 (dq, *J* = 9.9, 5.5 Hz, 1H), 3.08 – 2.71 (m, 4H), 2.63 (s, 3H), 2.42 (s, 3H), 2.12 – 1.95 (m, 3H), 1.91 – 1.80 (m, 2H), 1.67 (tq, *J* = 10.4, 2.9 Hz, 1H).

^{13}C NMR (126 MHz, DMSO) δ 157.3, 148.6, 143.4, 139.8, 138.6, 137.9, 136.2, 129.7, 129.4, 128.3, 127.4, 126.9, 124.9, 114.7, 56.2, 48.1, 44.6, 34.7, 32.3, 31.3, 16.3, 14.6.

HRMS: Calculated for $C_{22}H_{25}N_4{}^{15}N_2O^+$: 391.2025 [M+H$^+$]; found: 391.2015.

NMR spectra for previously unreported compounds:

^{1}H NMR of 4-(methoxymethoxy)-2-methylbut-1-ene ($CDCl_3$, 500 MHz)

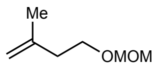

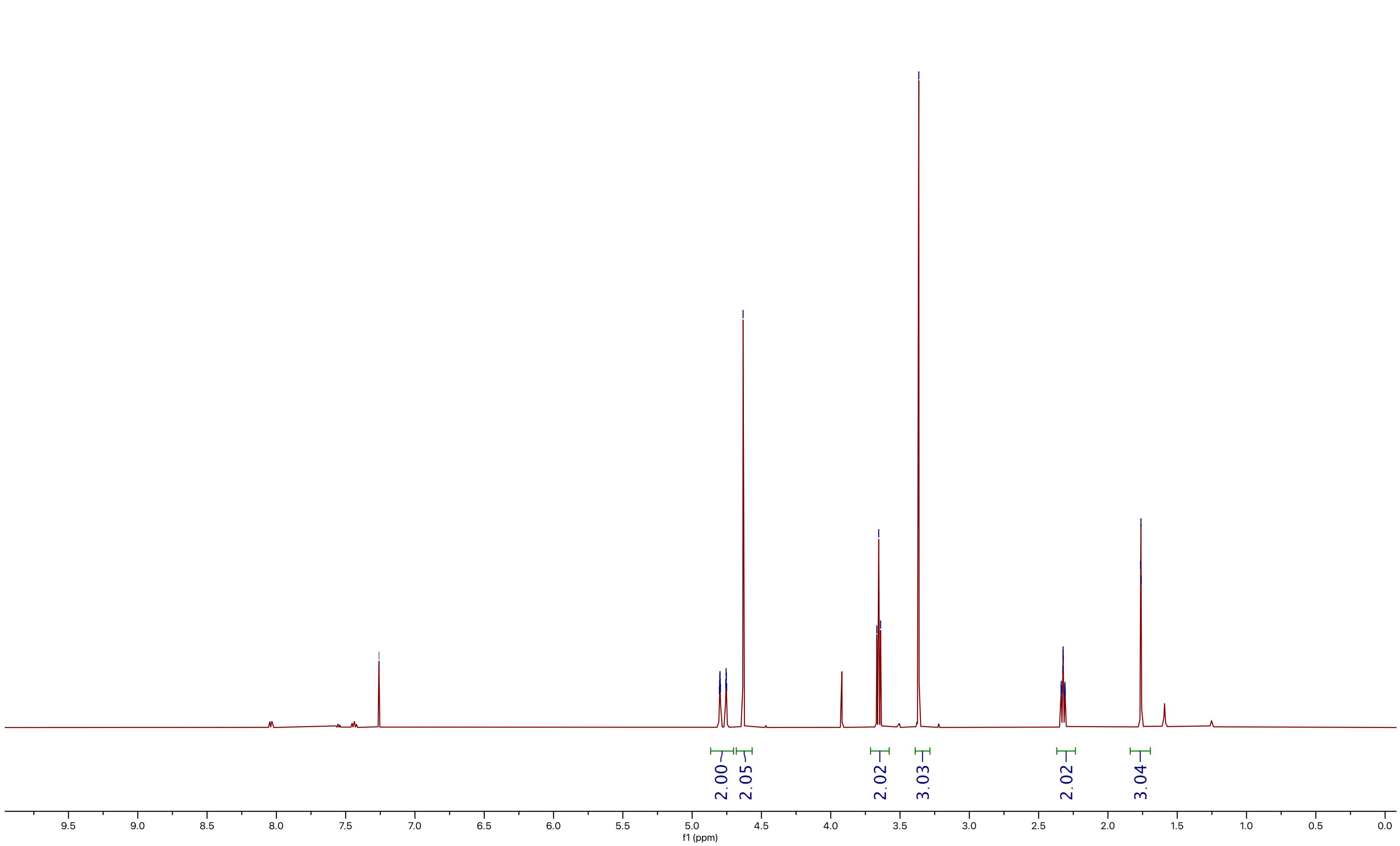

^{13}C NMR of 4-(methoxymethoxy)-2-methylbut-1-ene ($CDCl_3$, 126 MHz)

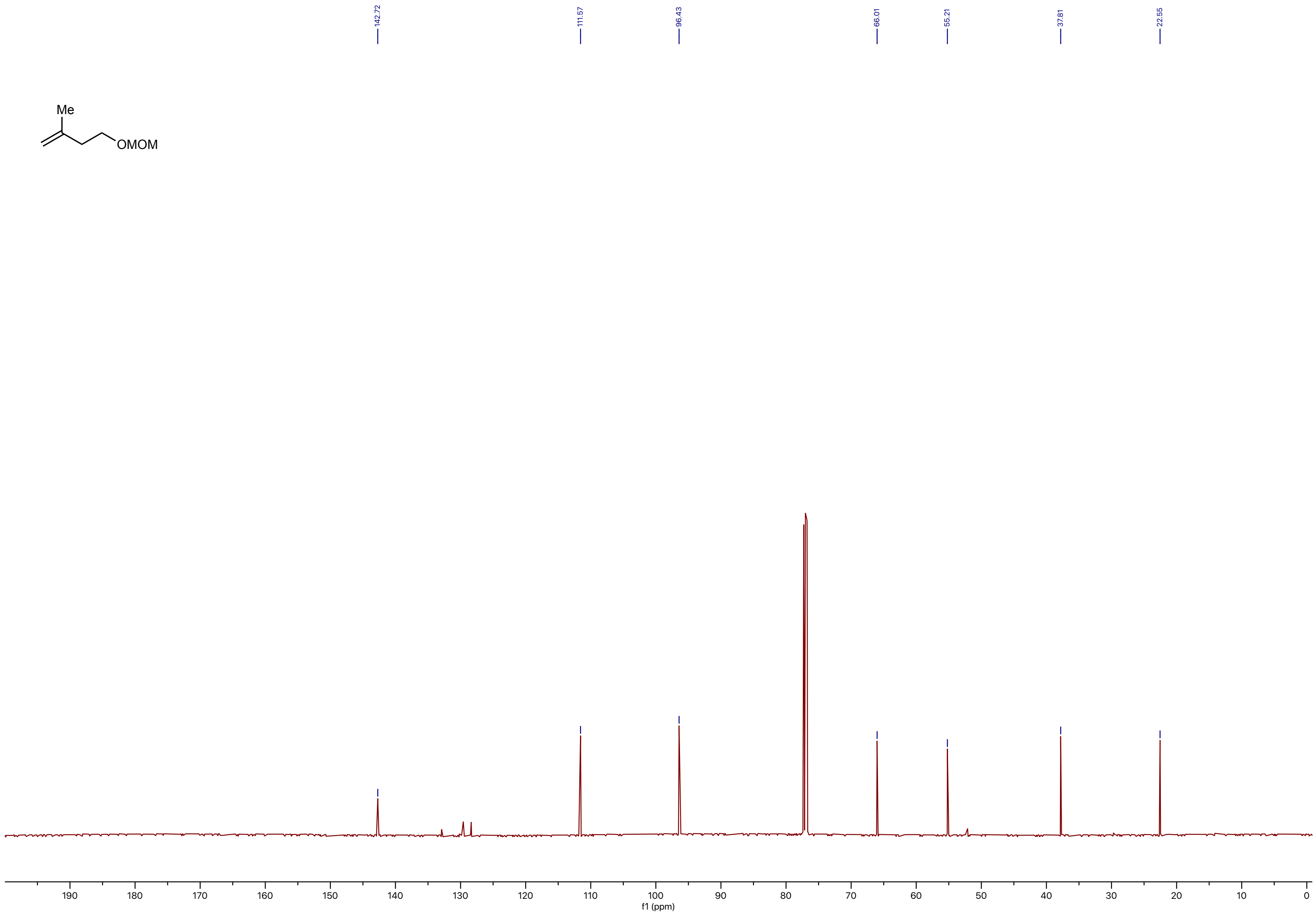

^{1}H NMR of *tert*-butyl (6-methoxyquinolin-8-yl)(pent-4-en-1-yl)carbamate ($CDCl_3$, 500 MHz)

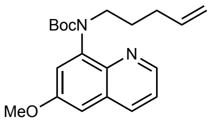

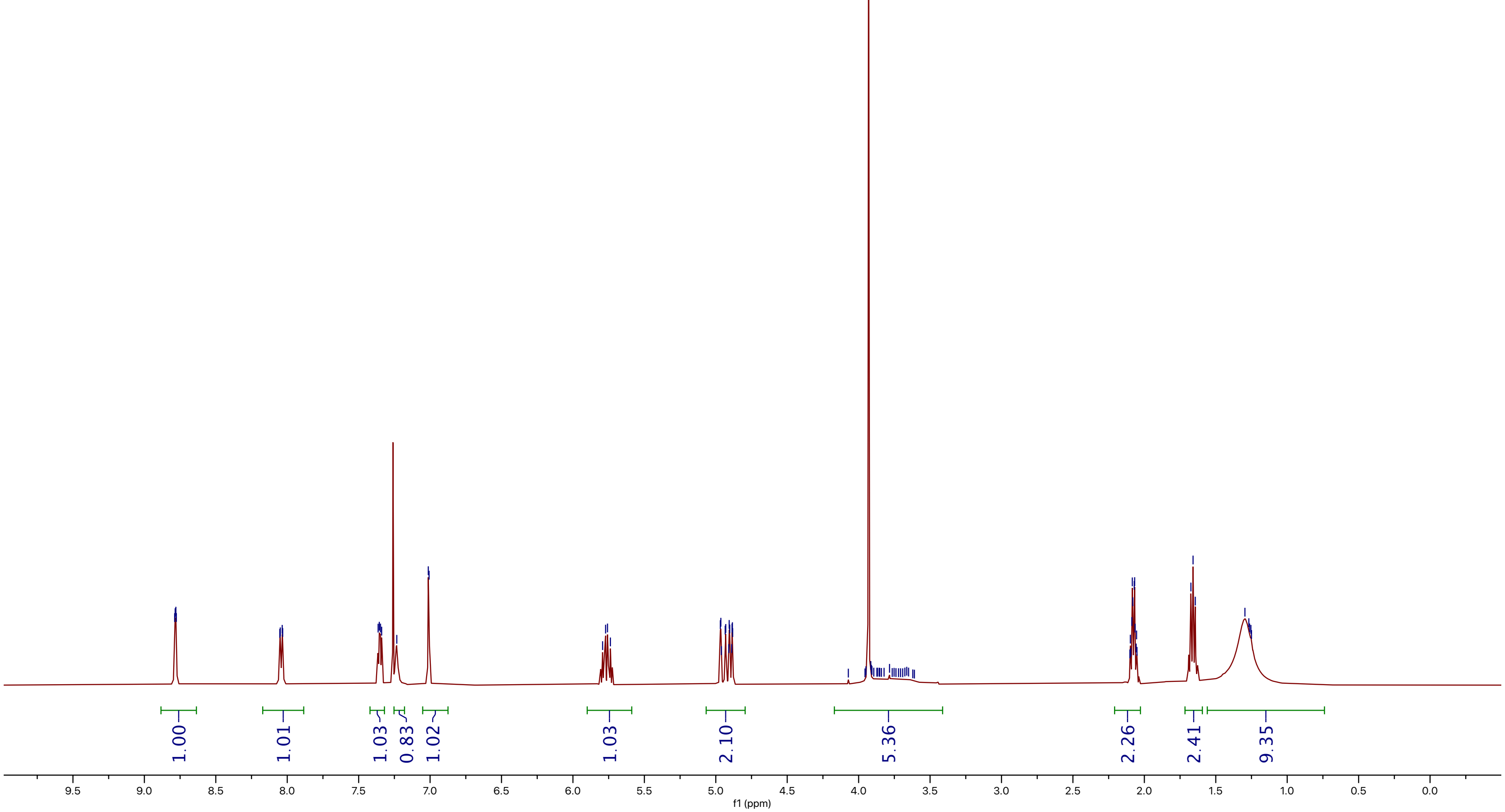

¹³C NMR of *tert*-butyl (6-methoxyquinolin-8-yl)(pent-4-en-1-yl)carbamate ($CDCl_3$, 126 MHz)

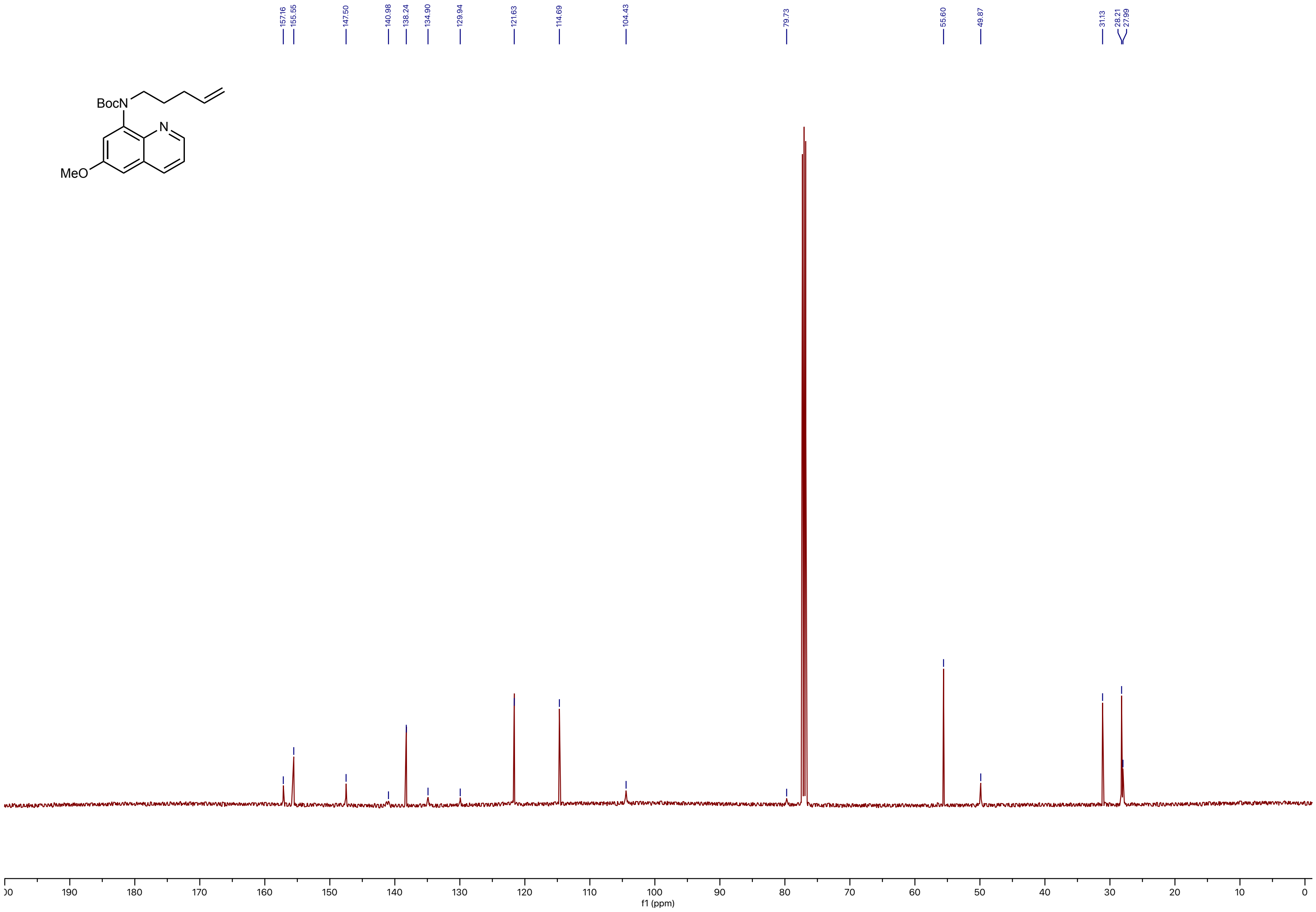

^{1}H NMR of 4-methoxybenzyl 3,7-dimethyloct-6-enoate ($CDCl_3$, 500 MHz)

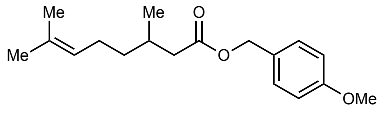

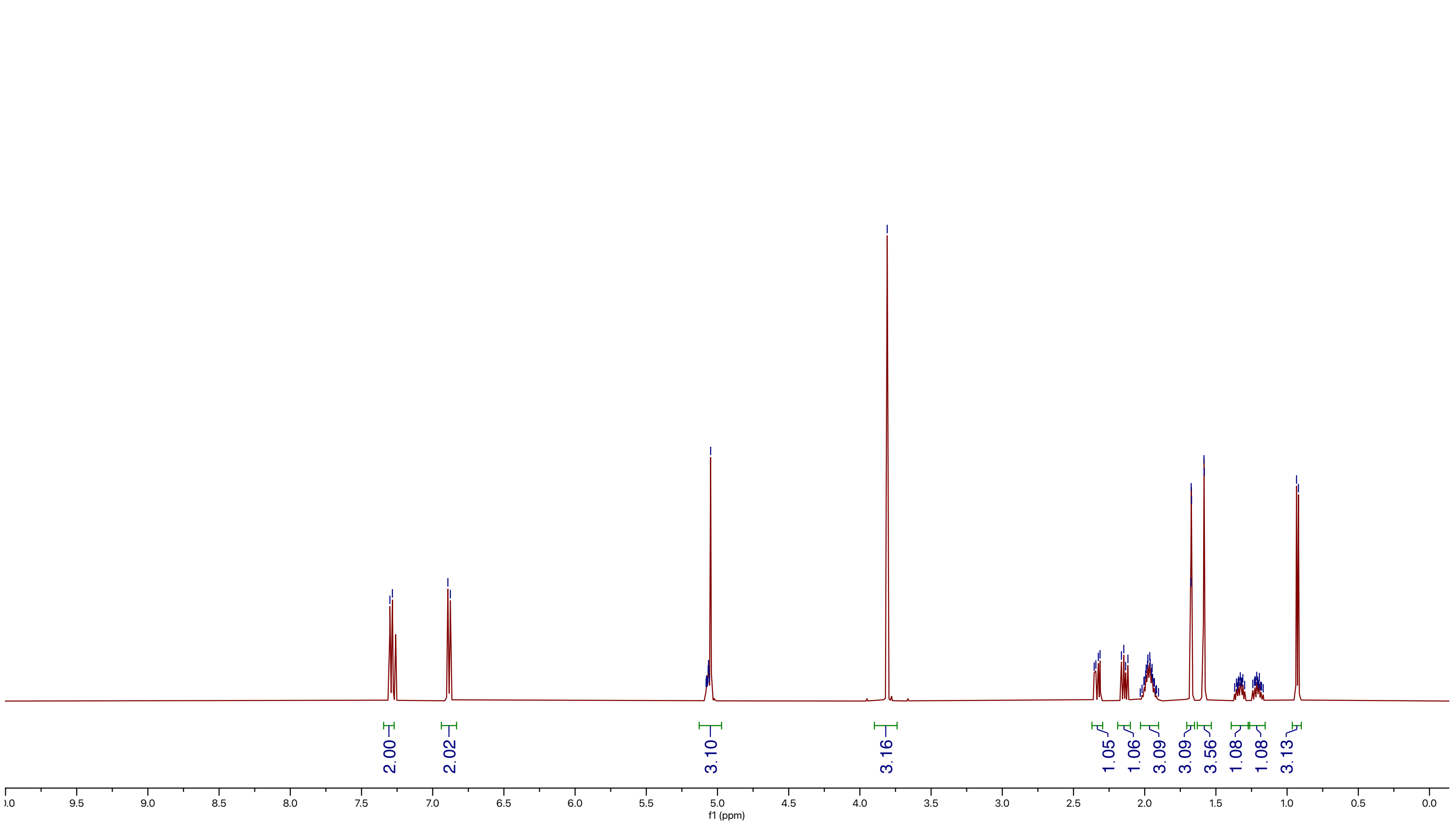

^{13}C NMR of 4-methoxybenzyl 3,7-dimethyloct-6-enoate ($CDCl_3$, 126 MHz)

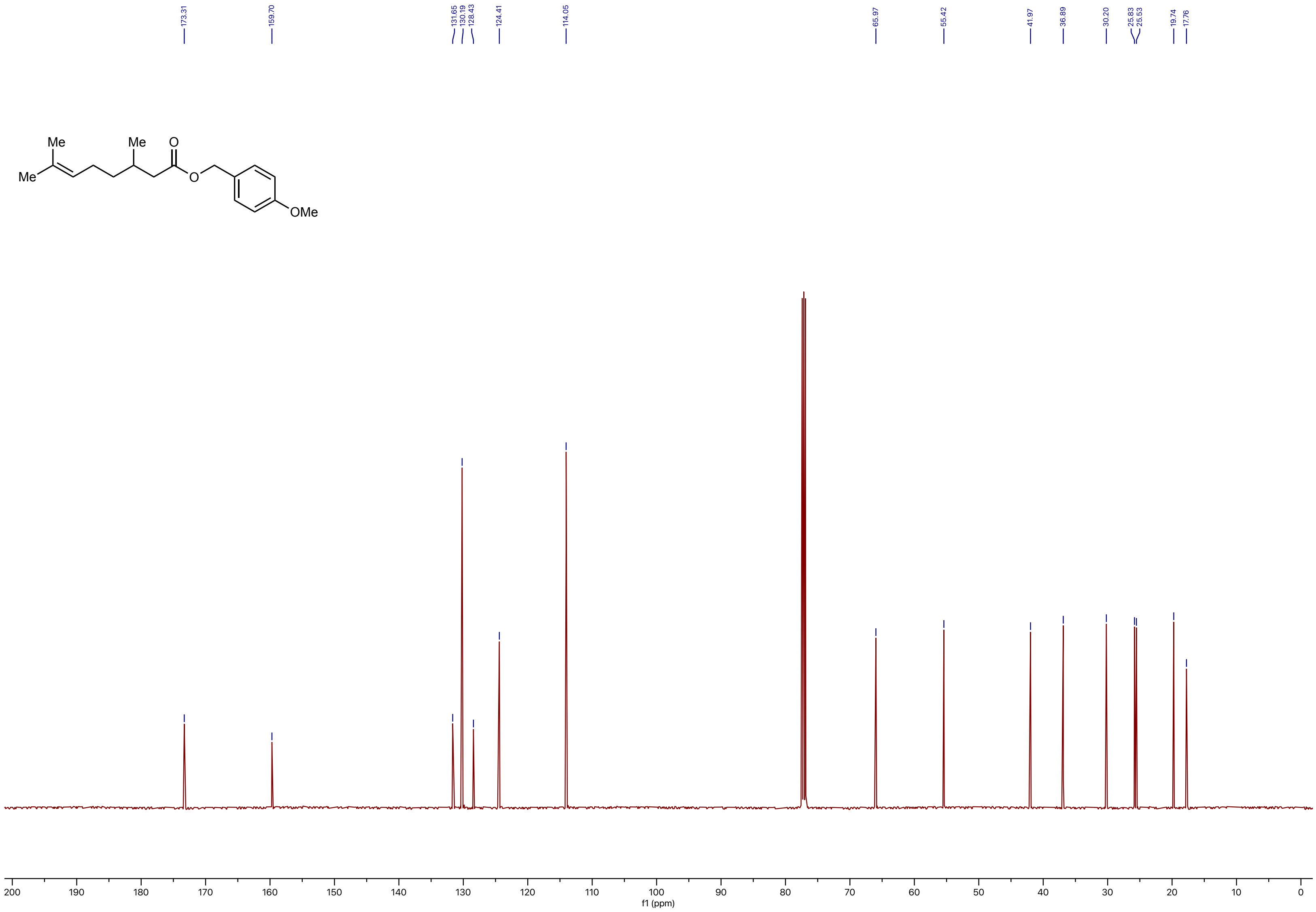

^{1}H NMR of *tert*-butyl (4-(but-3-en-1-yloxy)phenyl)carbamate ($CDCl_3$, 500 MHz)

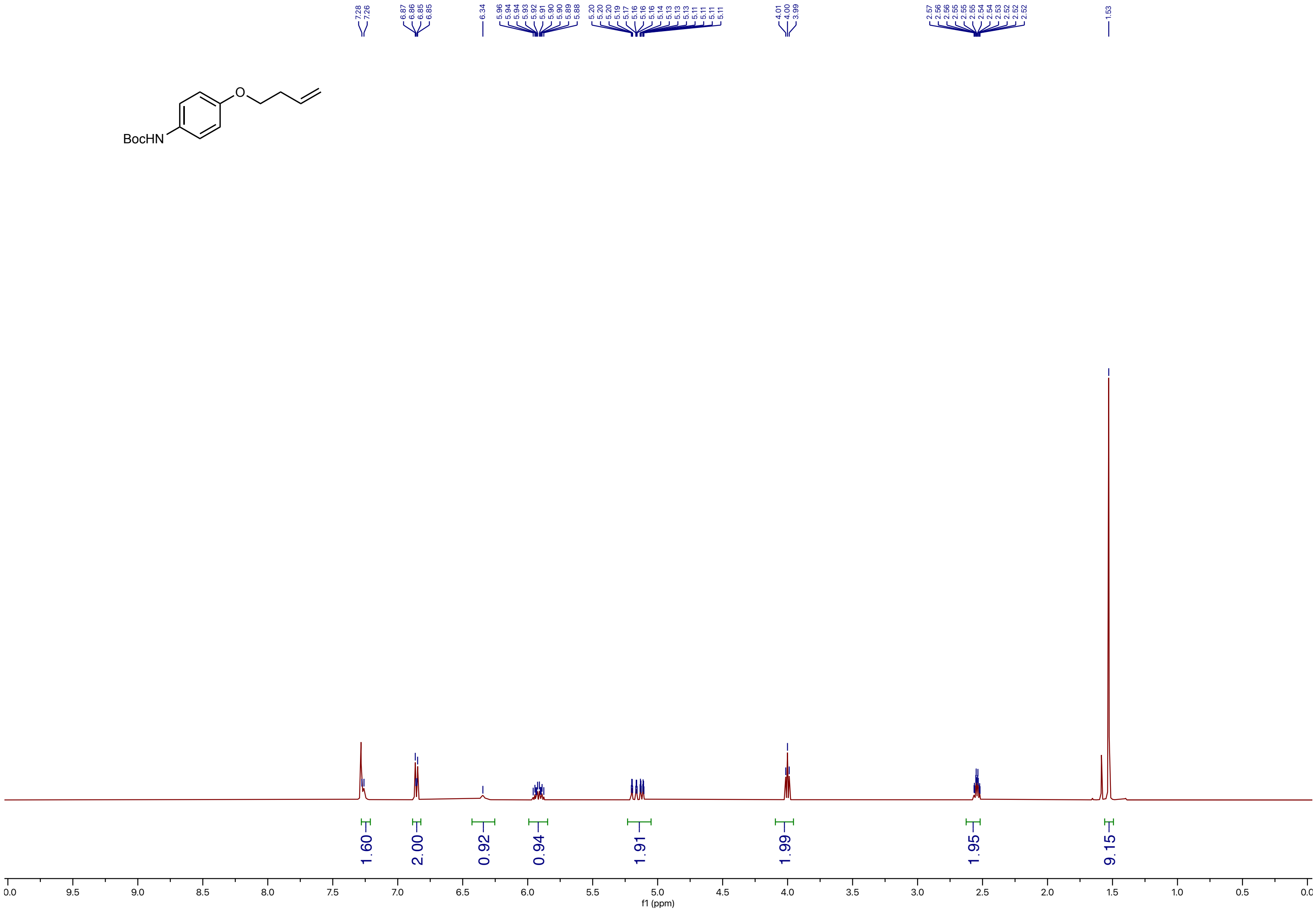

^{13}C NMR of *tert*-butyl (4-(but-3-en-1-yloxy)phenyl)carbamate ($CDCl_3$, 126 MHz)

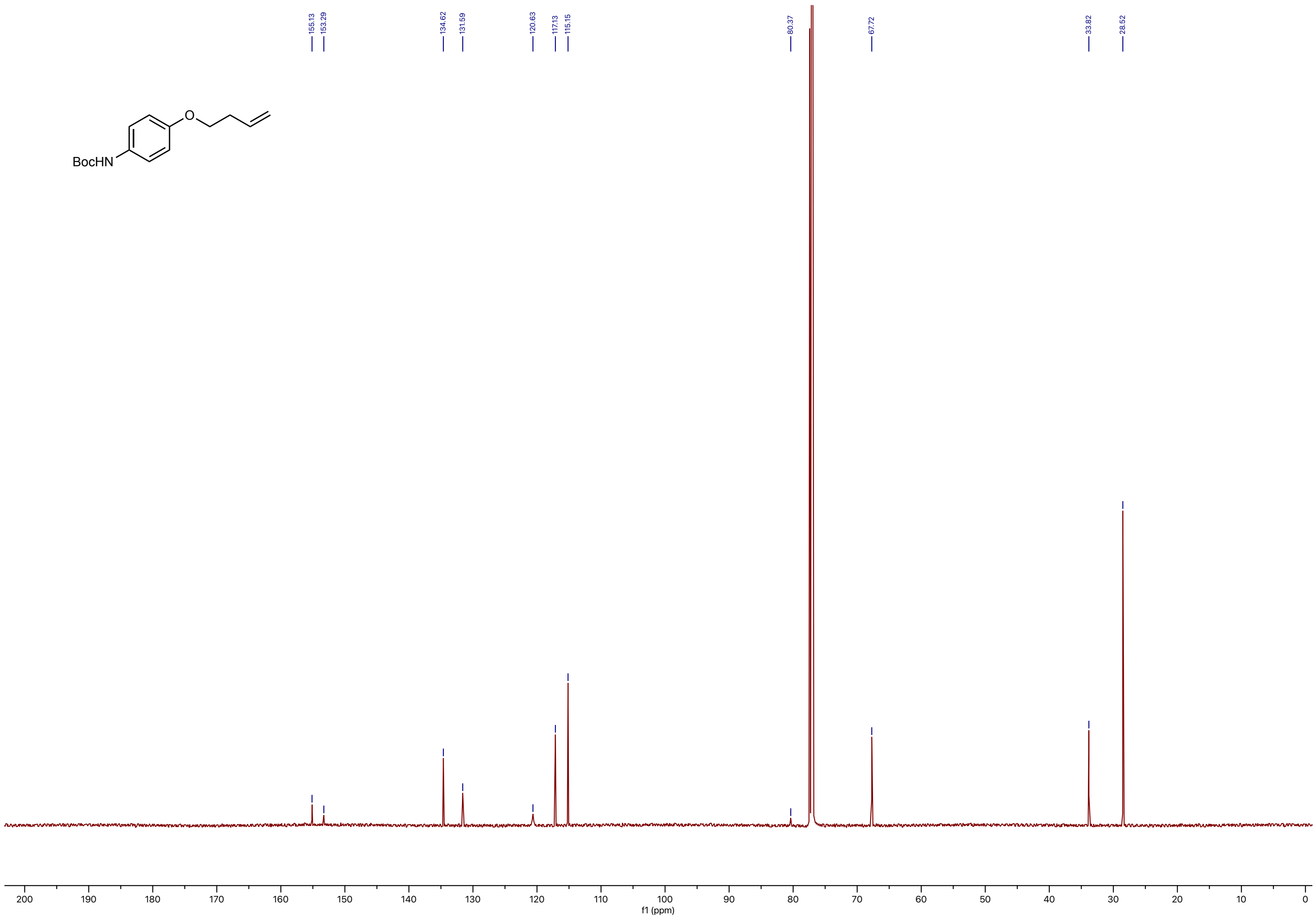

^{1}H NMR of 4-(But-3-en-1-yloxy)aniline ($CDCl_3$, 500 MHz)

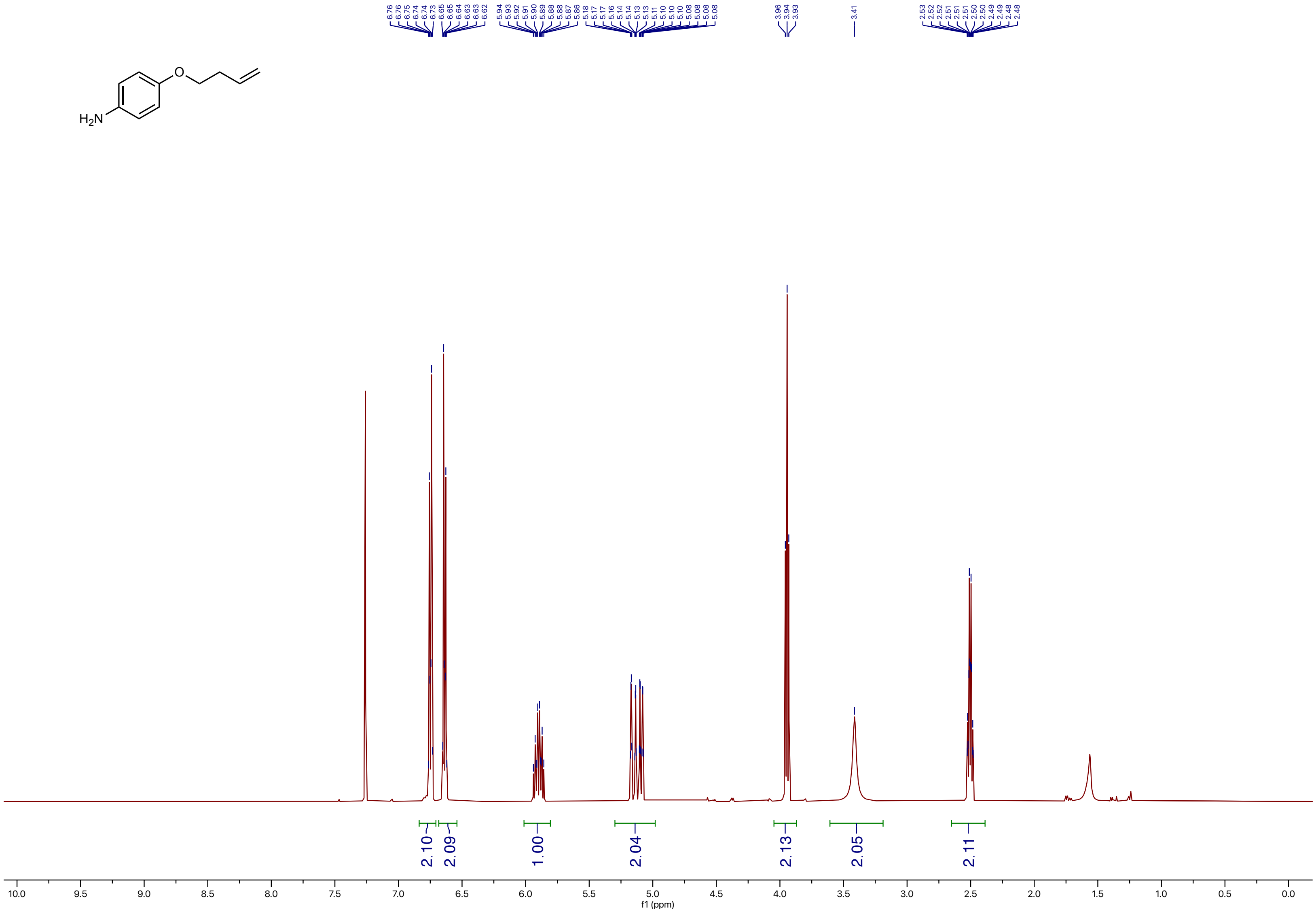

^{13}C NMR of 4-(But-3-en-1-yloxy)aniline ($CDCl_3$, 126 MHz)

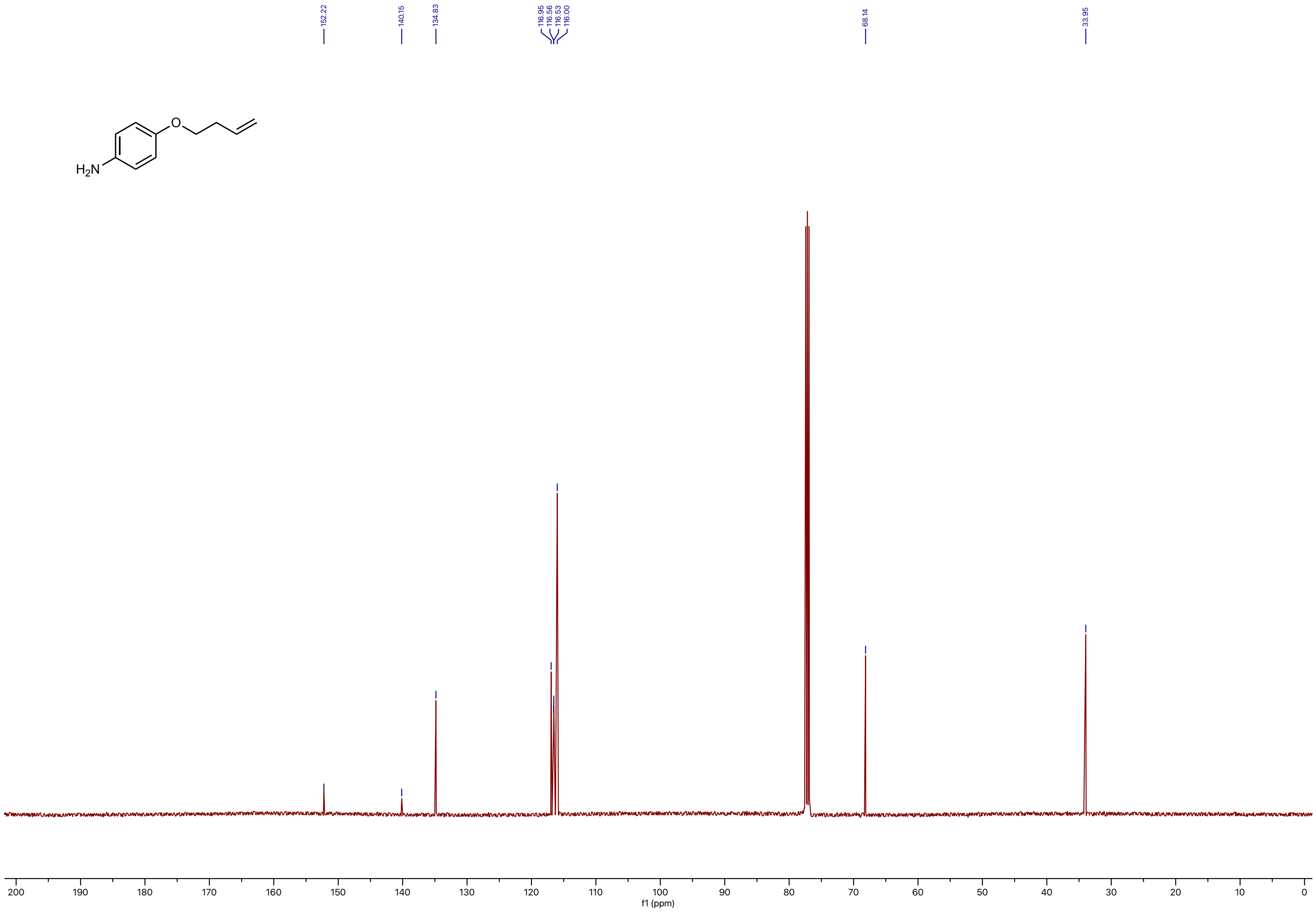

^{1}H NMR of Benzyl (4-(but-3-en-1-yloxy)phenyl)carbamate ($CDCl_3$, 500 MHz)

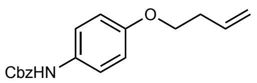

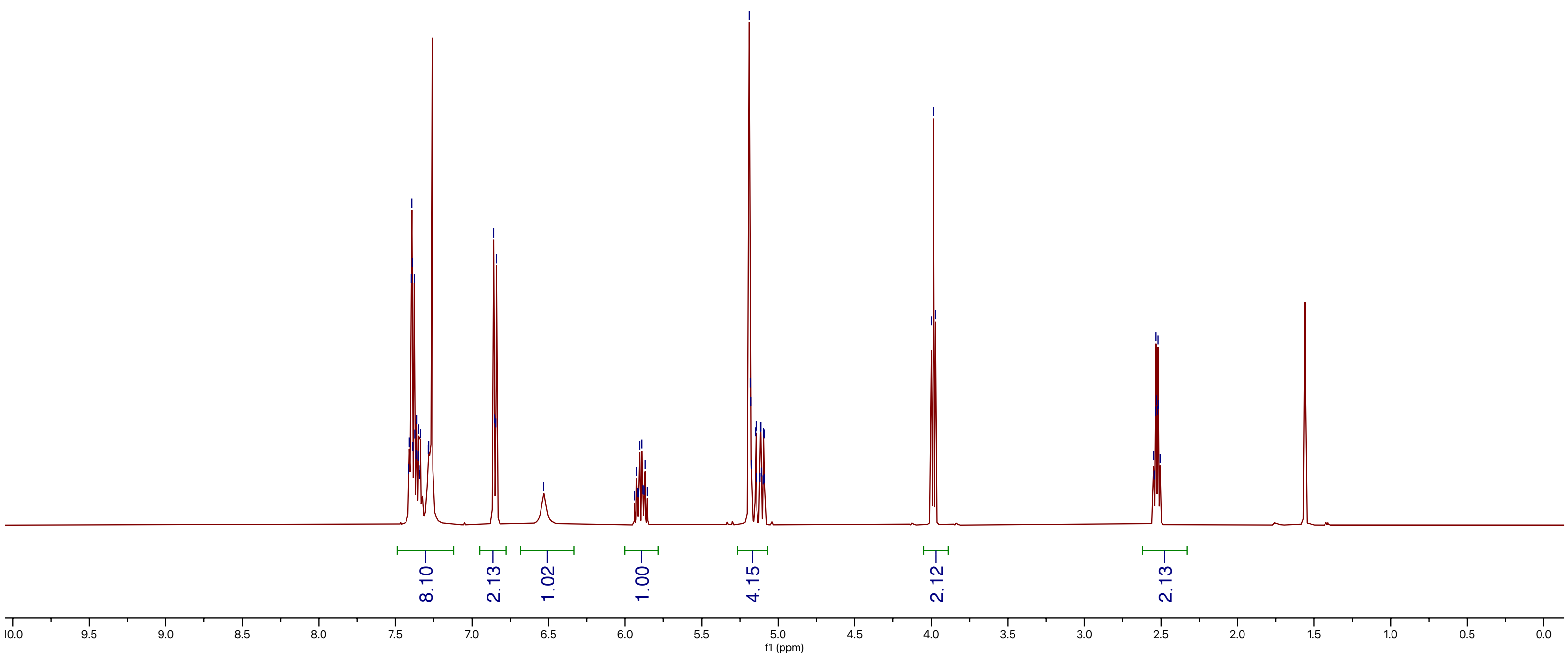

^{13}C NMR of Benzyl (4-(but-3-en-1-yloxy)phenyl)carbamate ($CDCl_3$, 126 MHz)

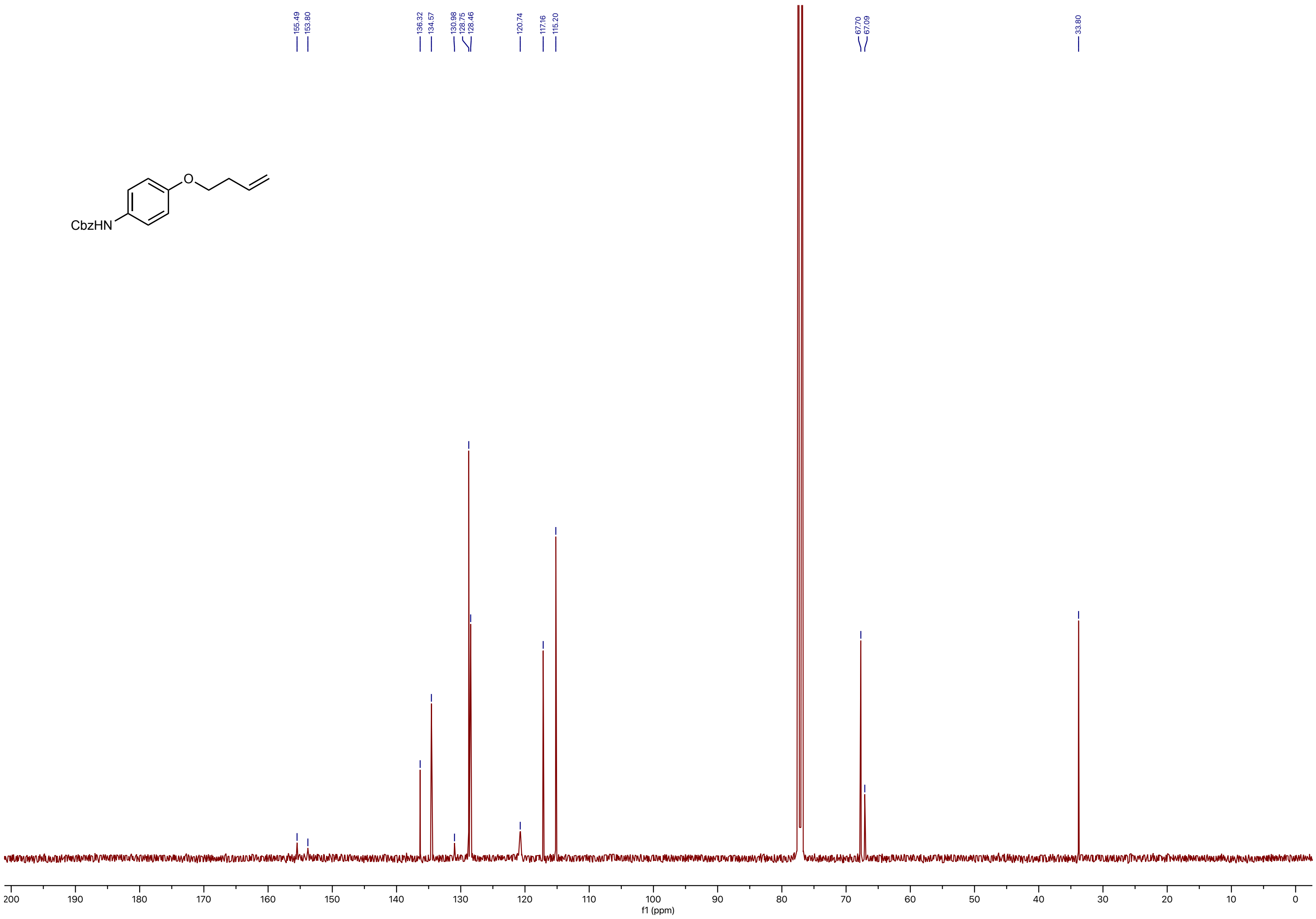

^{1}H NMR of 3a ($CDCl_3$, 500 MHz)

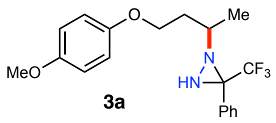

3a

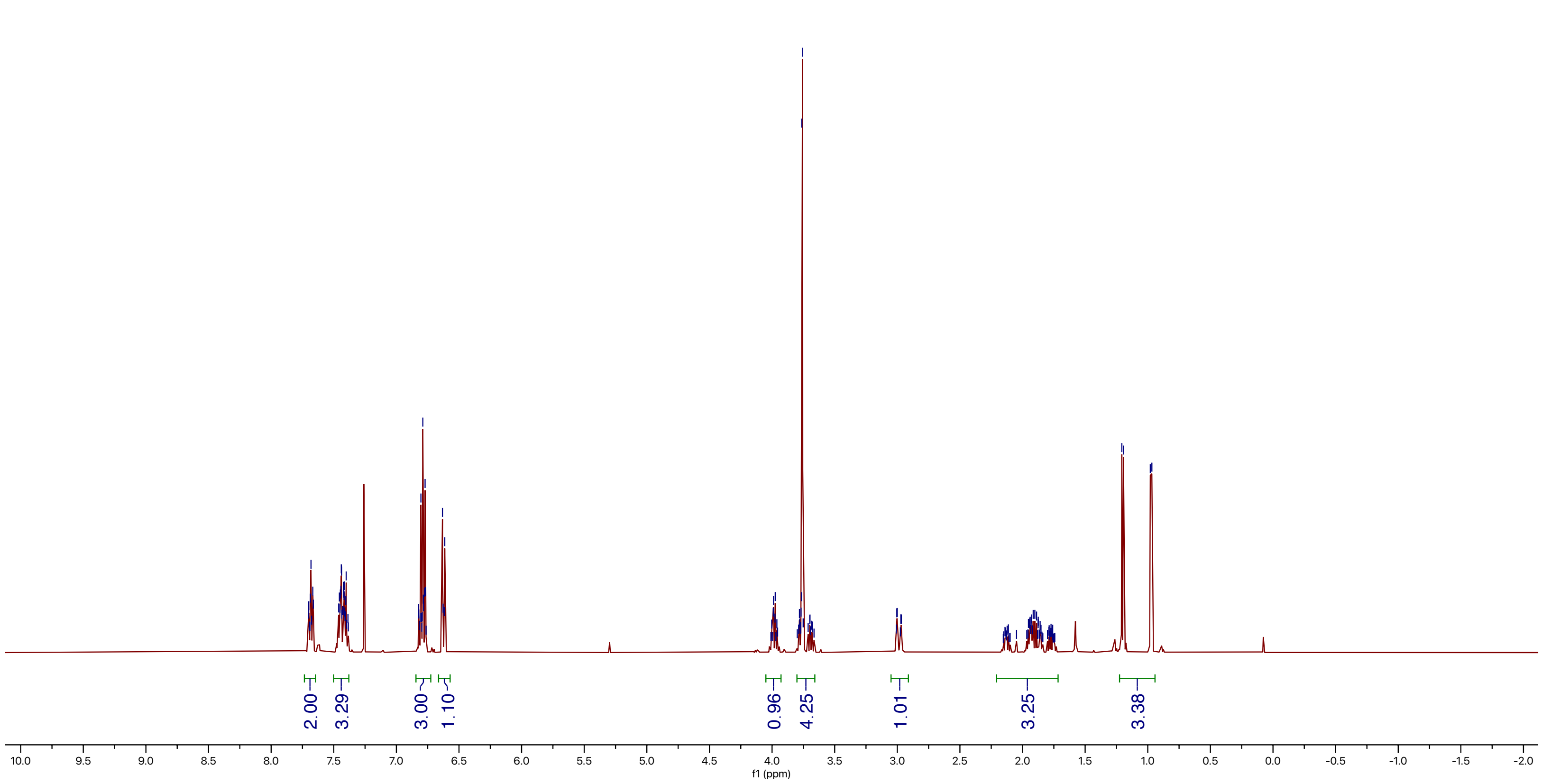

^{13}C NMR of 3a ($CDCl_3$, 126 MHz)

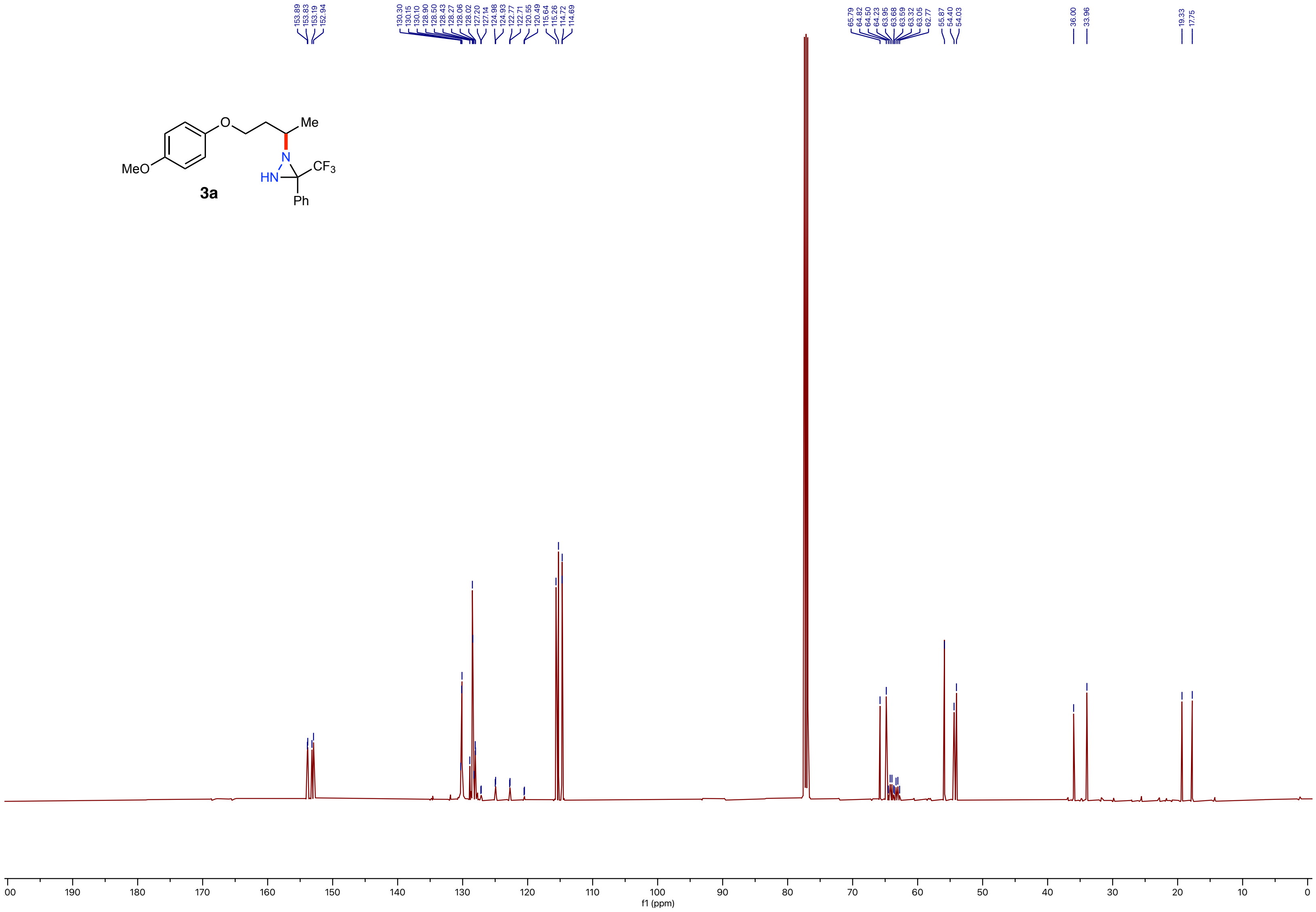

^{19}F NMR of 3a ($CDCl_3$, 471 MHz)

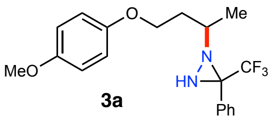

3a

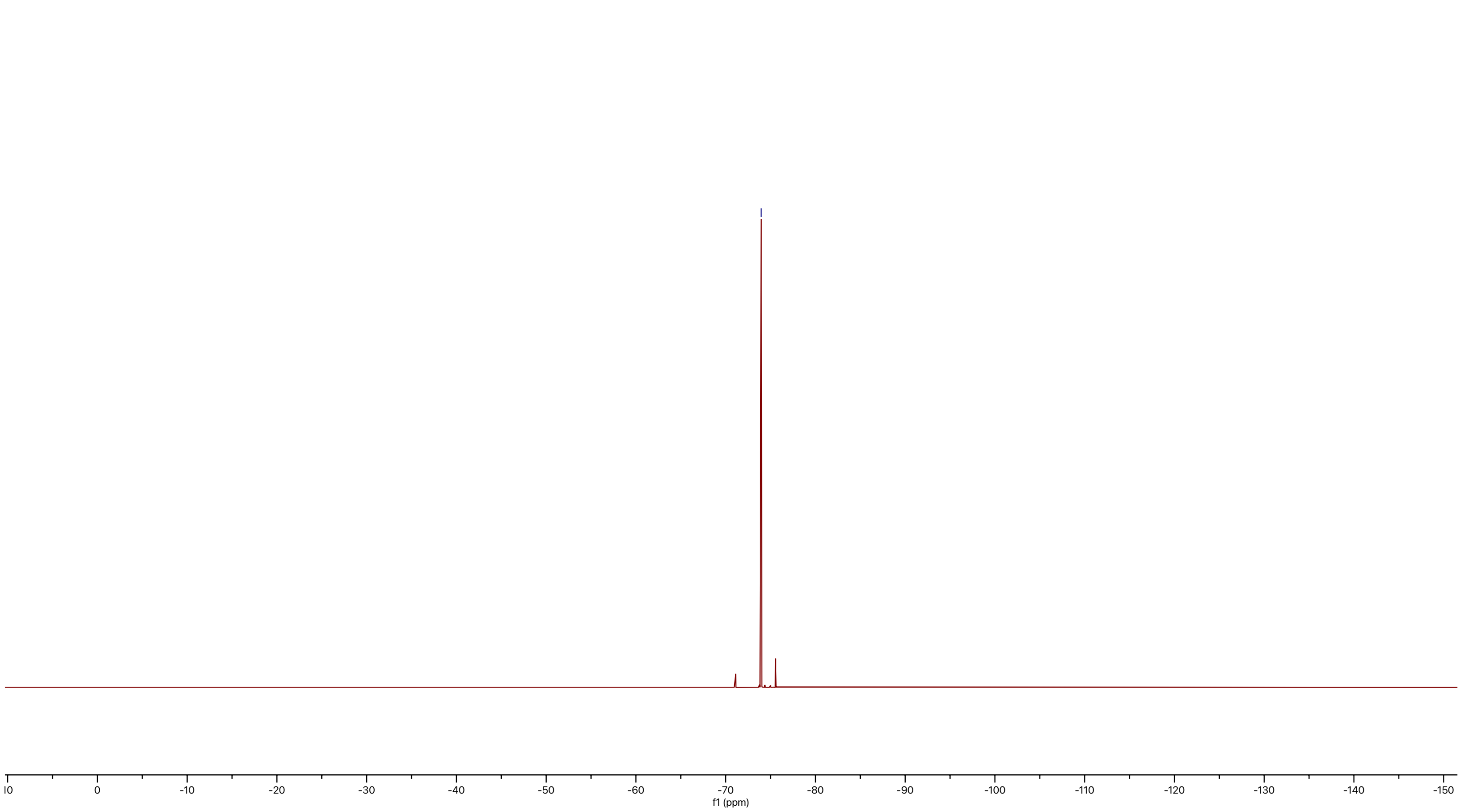

^{1}H NMR of 3b ($CDCl_3$, 500 MHz)

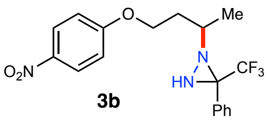

3b

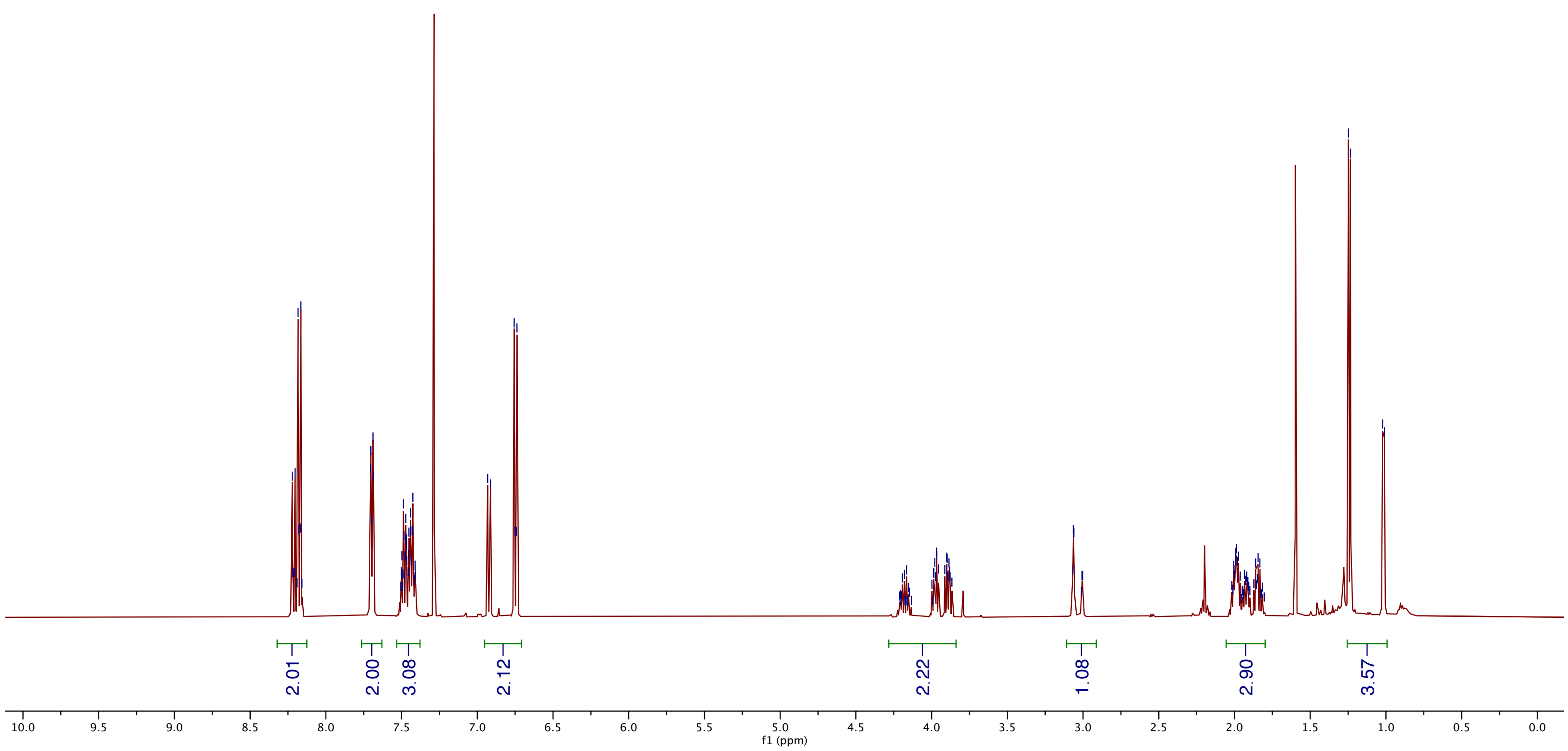

^{13}C NMR of 3b (CDCl$_3$, 126 MHz)

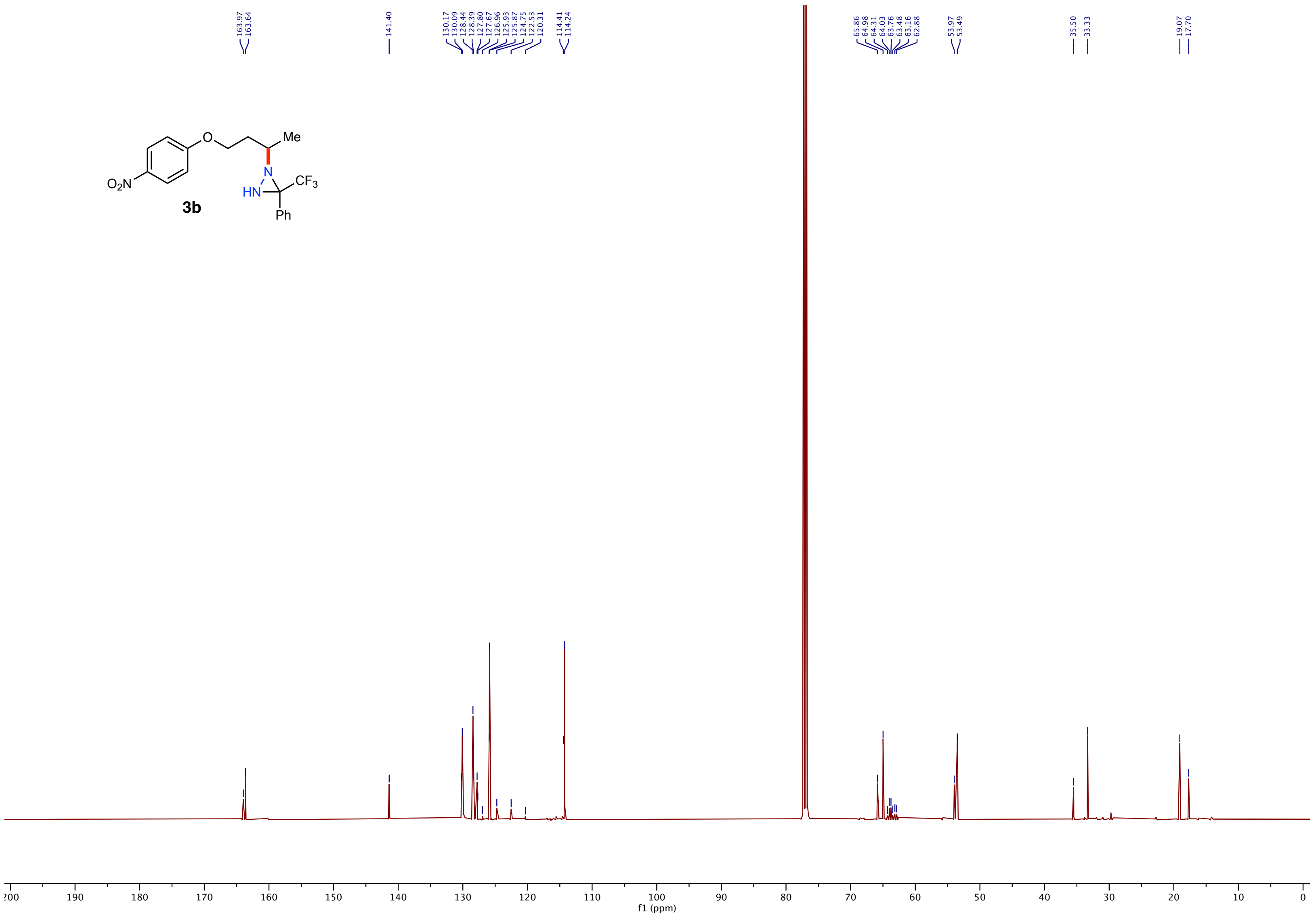

^{19}F NMR of 3b ($CDCl_3$, 471 MHz)

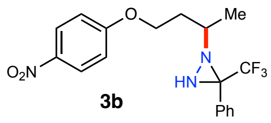

3b

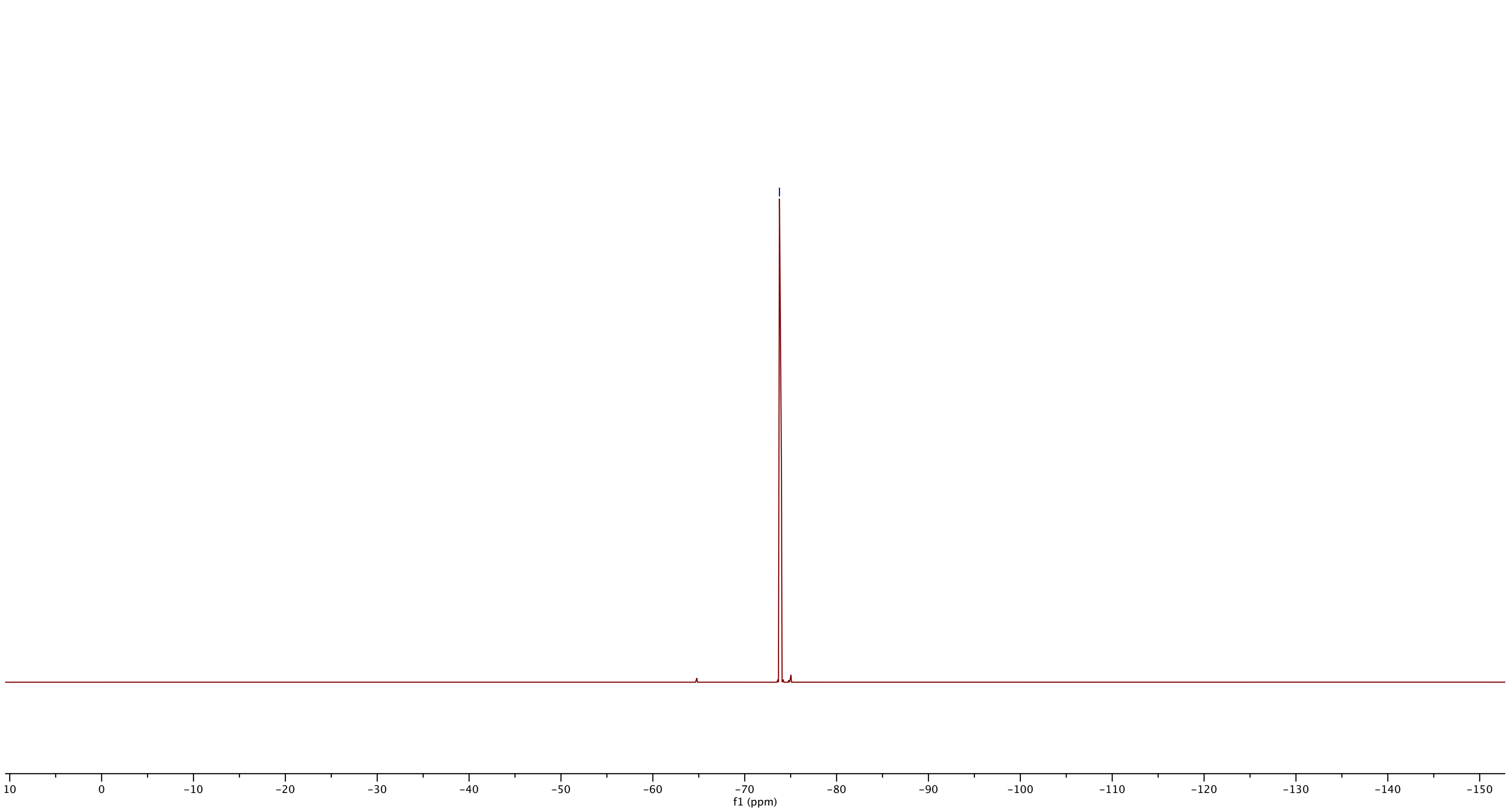

1H NMR of 3c (CDCl3, 500 MHz)

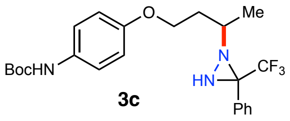

3c

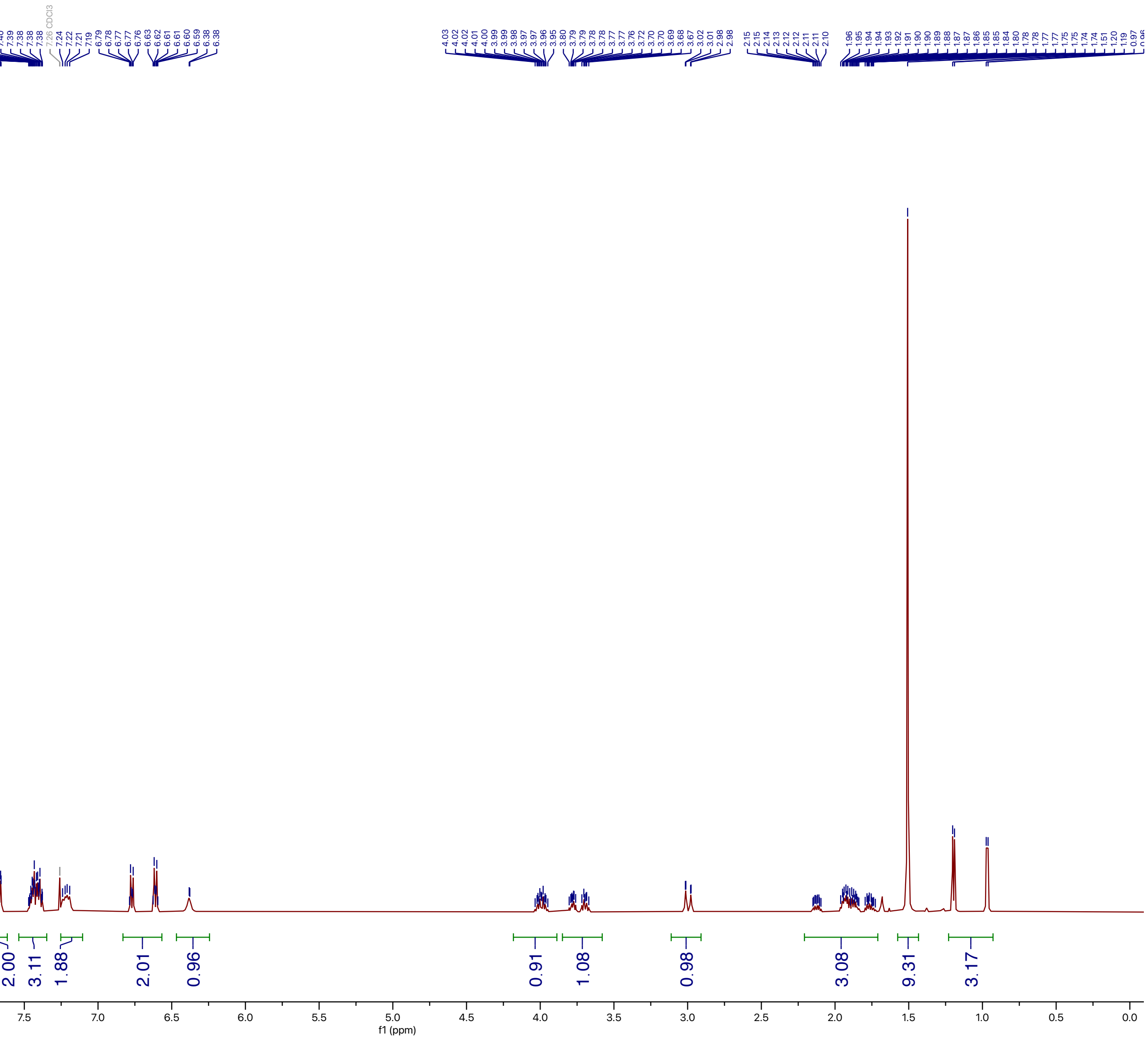

^{13}C NMR of 3c ($CDCl_3$, 126 MHz)

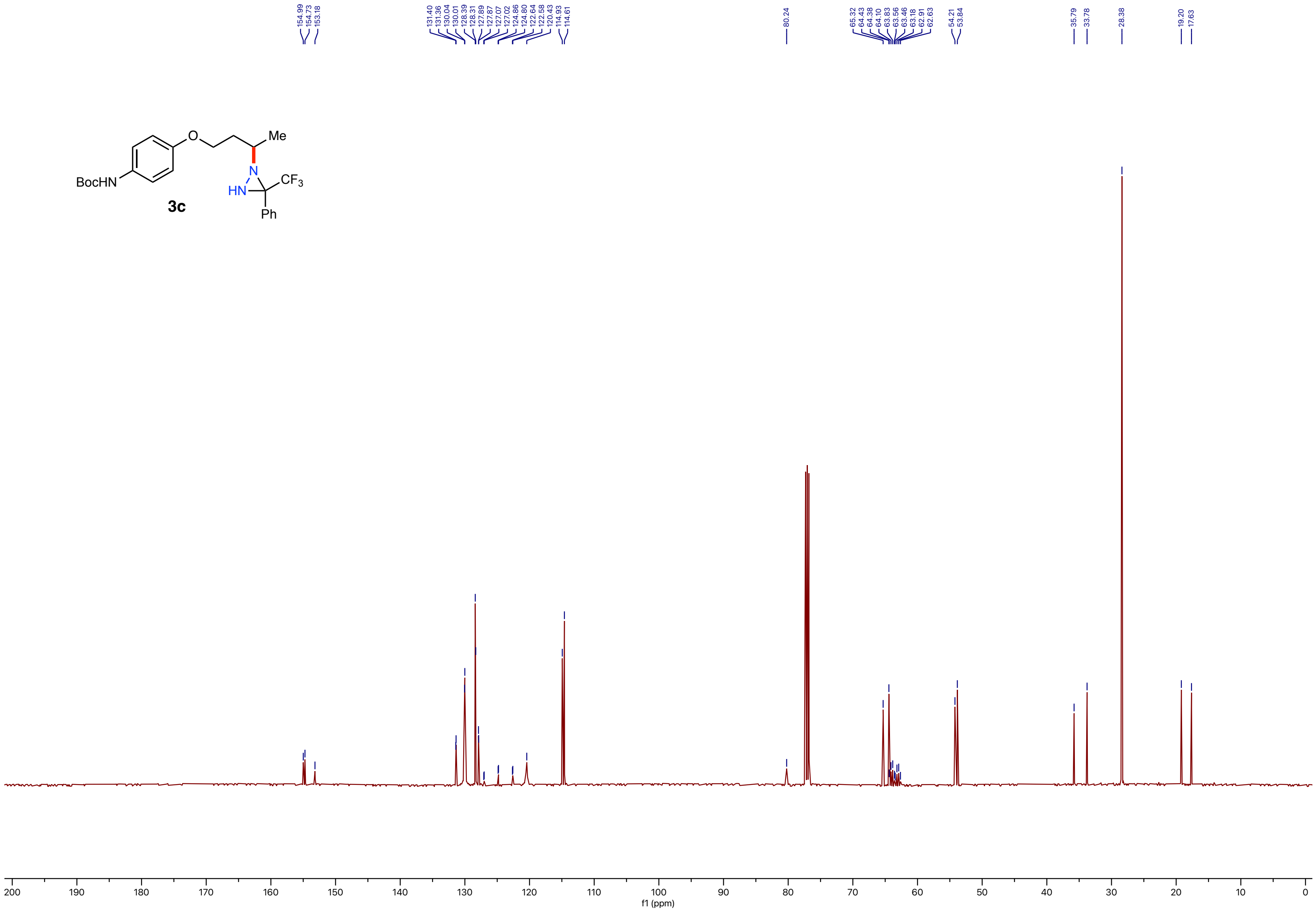

^{19}F NMR of 3c ($CDCl_3$, 471 MHz)

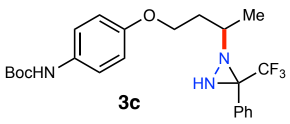

3c

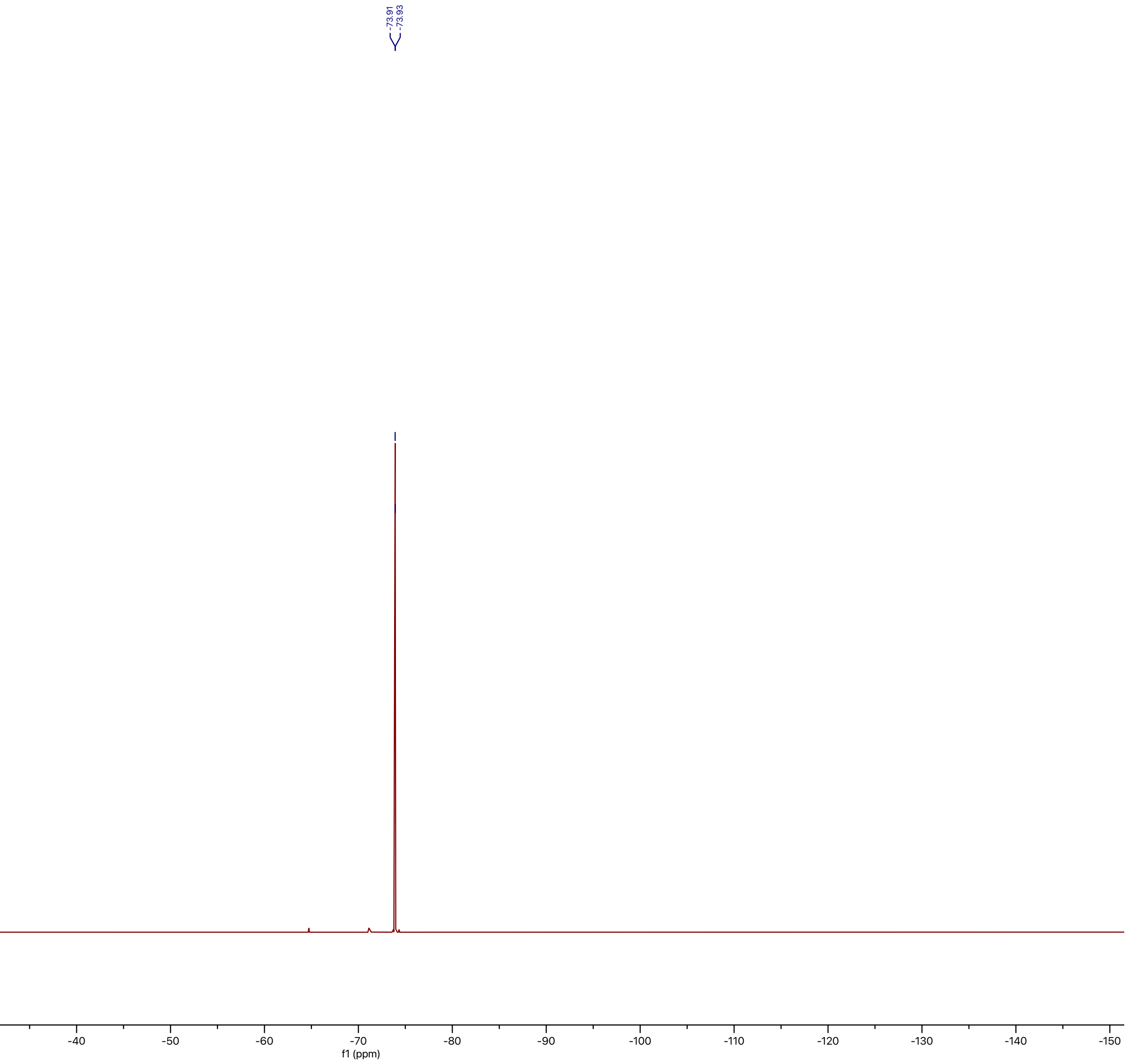

^{1}H NMR of 3d ($CDCl_3$, 500 MHz)

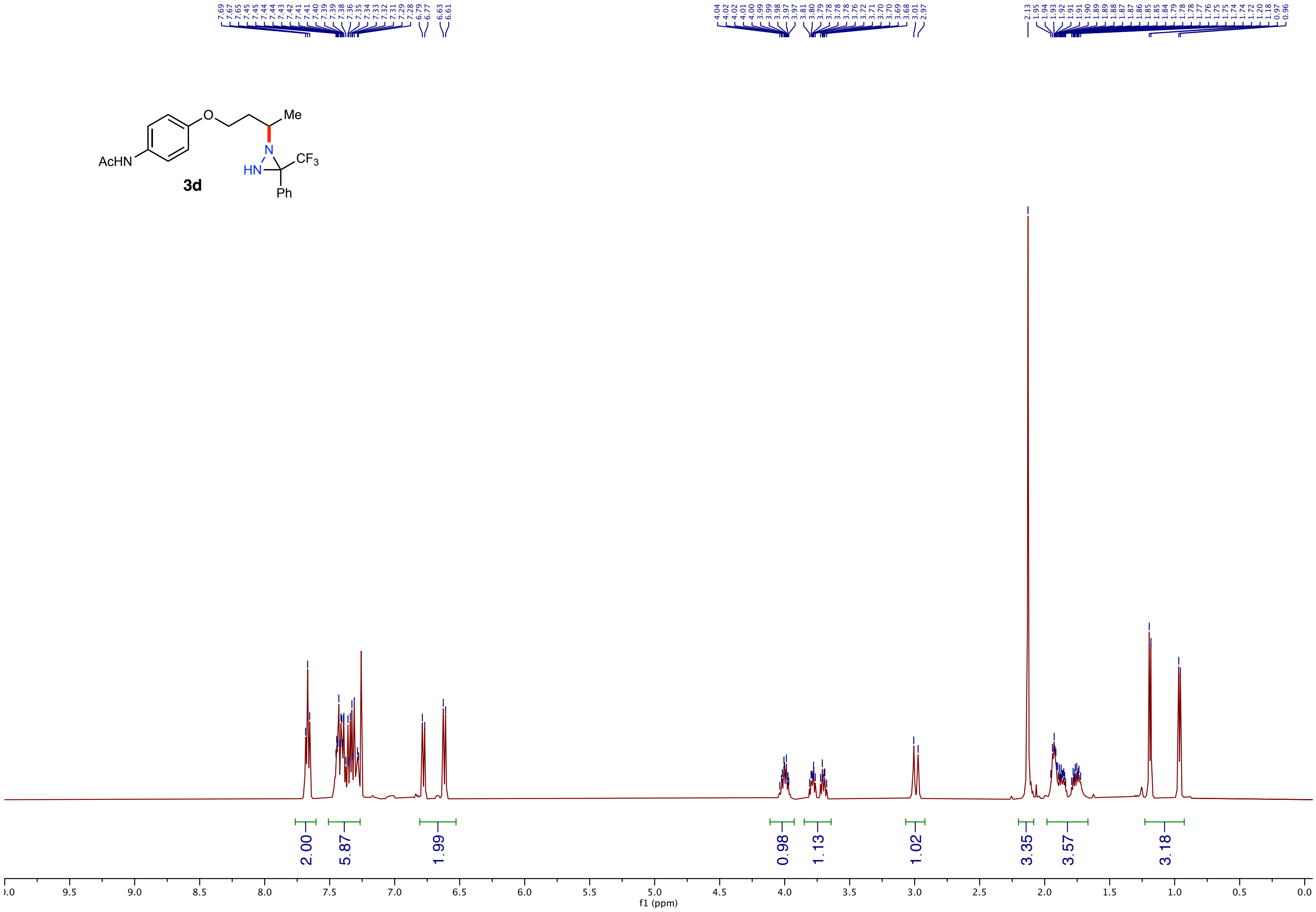

^{13}C NMR of 3d ($CDCl_3$, 126 MHz)

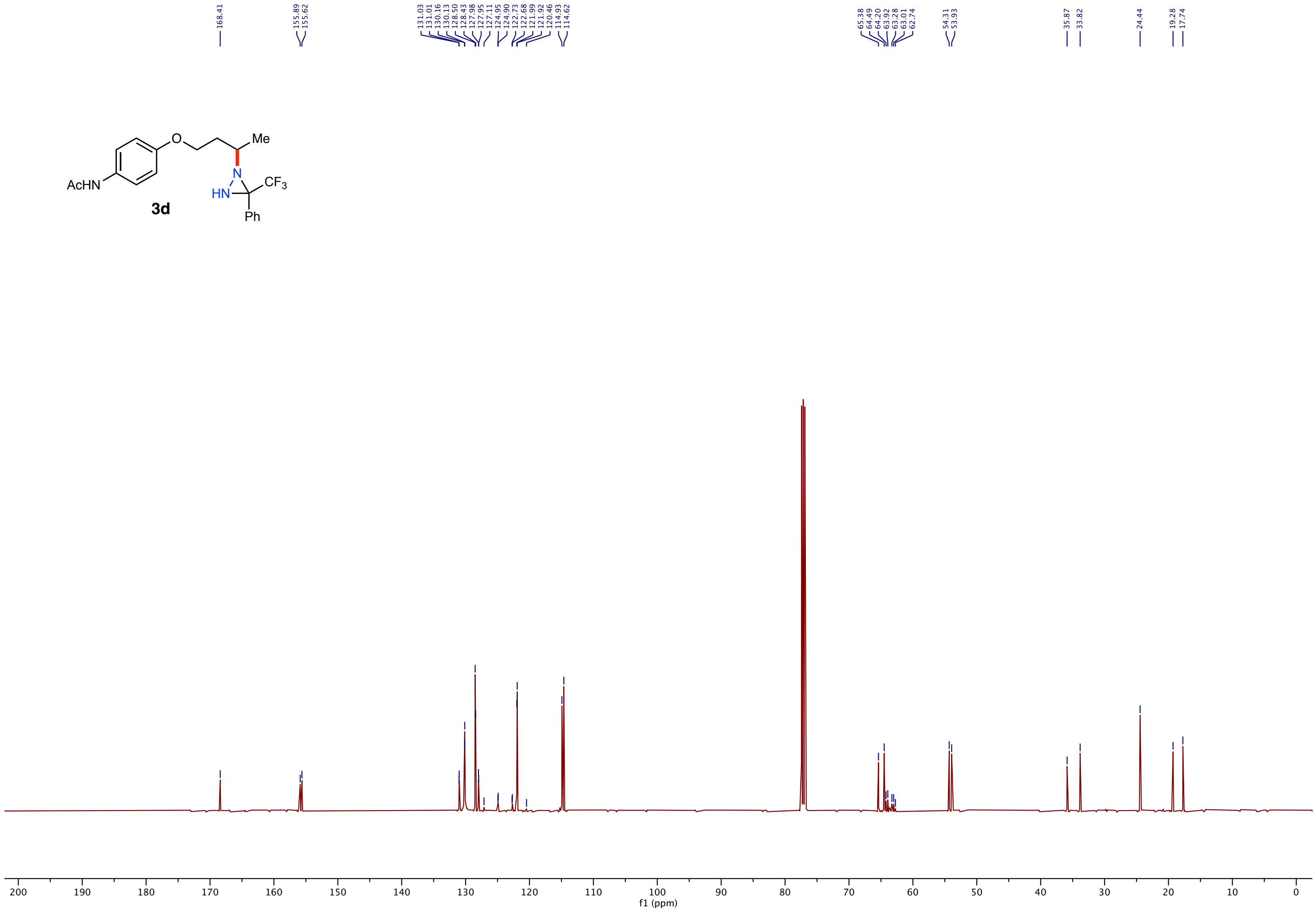

^{19}F NMR of 3d ($CDCl_3$, 471 MHz)

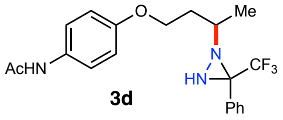

3d

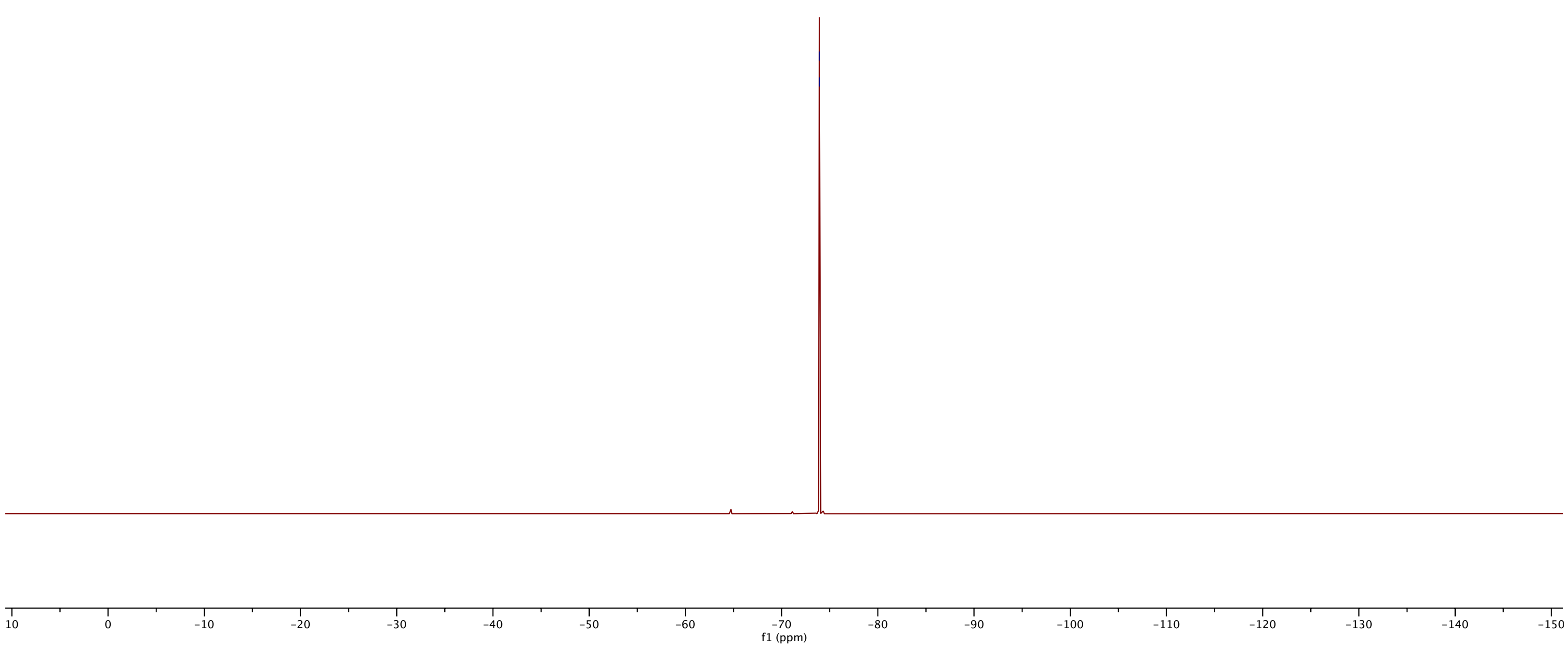

^{1}H NMR of 3e ($CDCl_3$, 500 MHz)

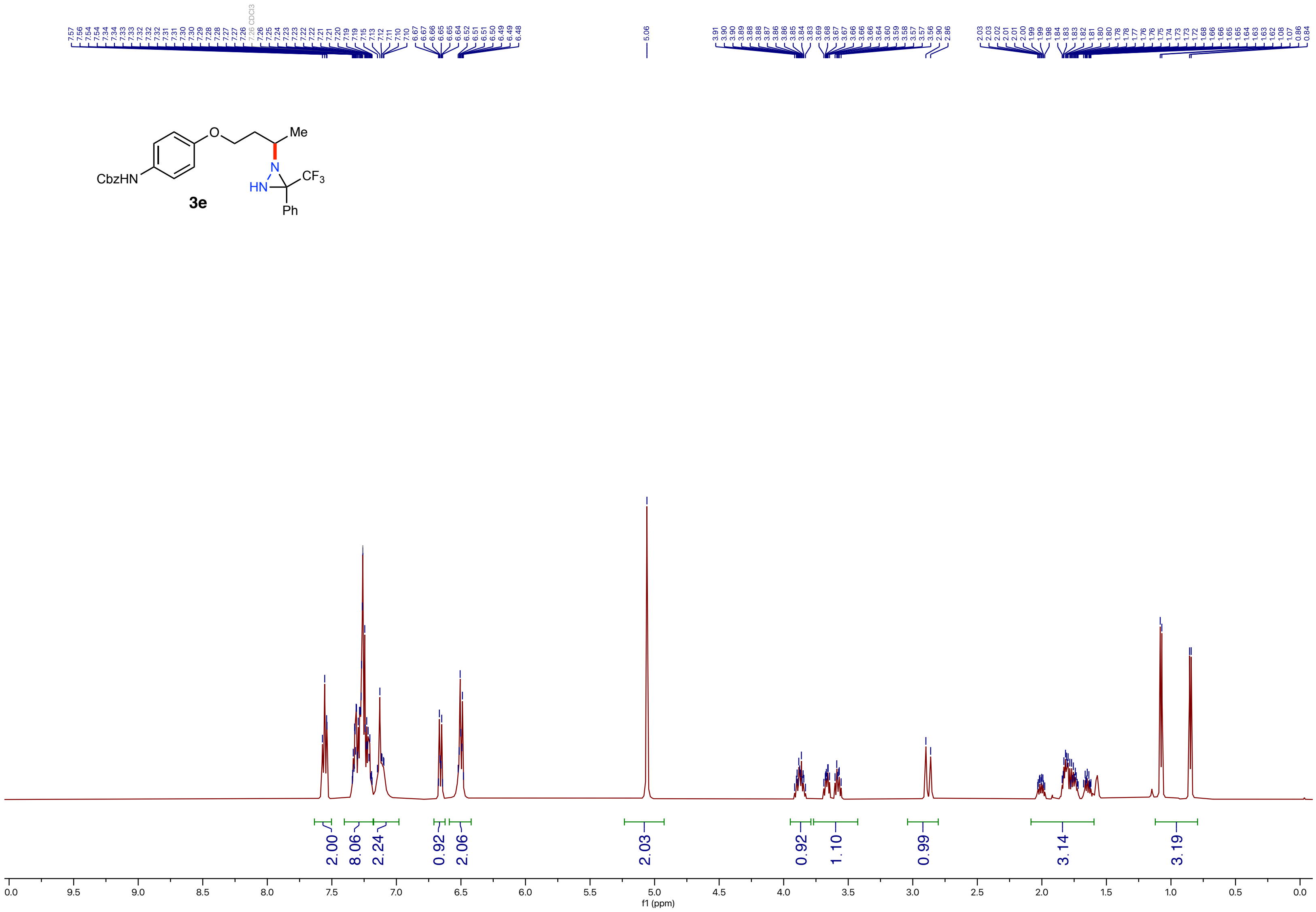

^{13}C NMR of 3e (CDCl$_3$, 126 MHz)

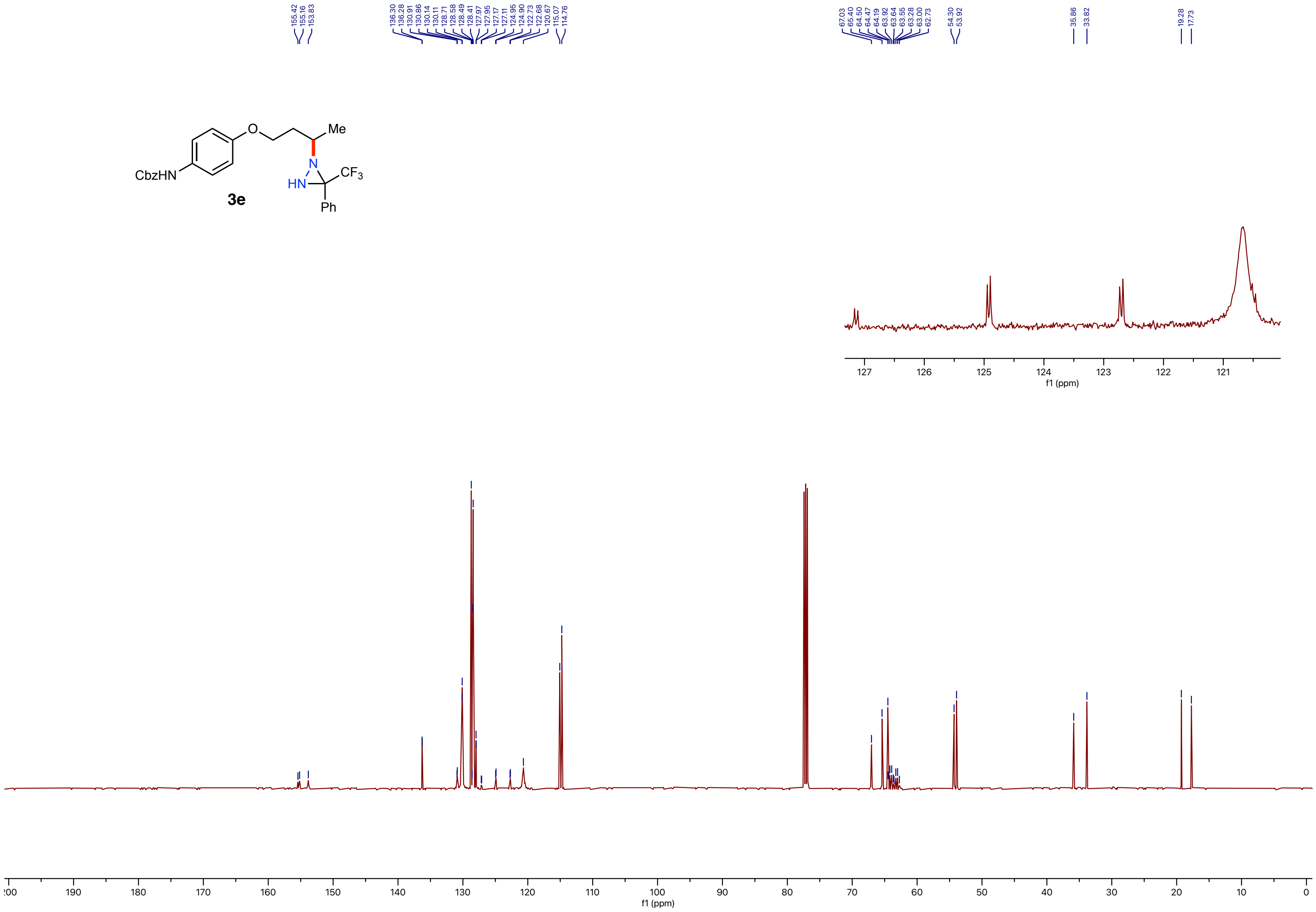

^{19}F NMR of 3e ($CDCl_3$, 471 MHz)

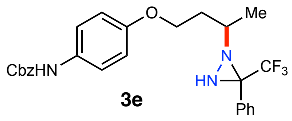

3e

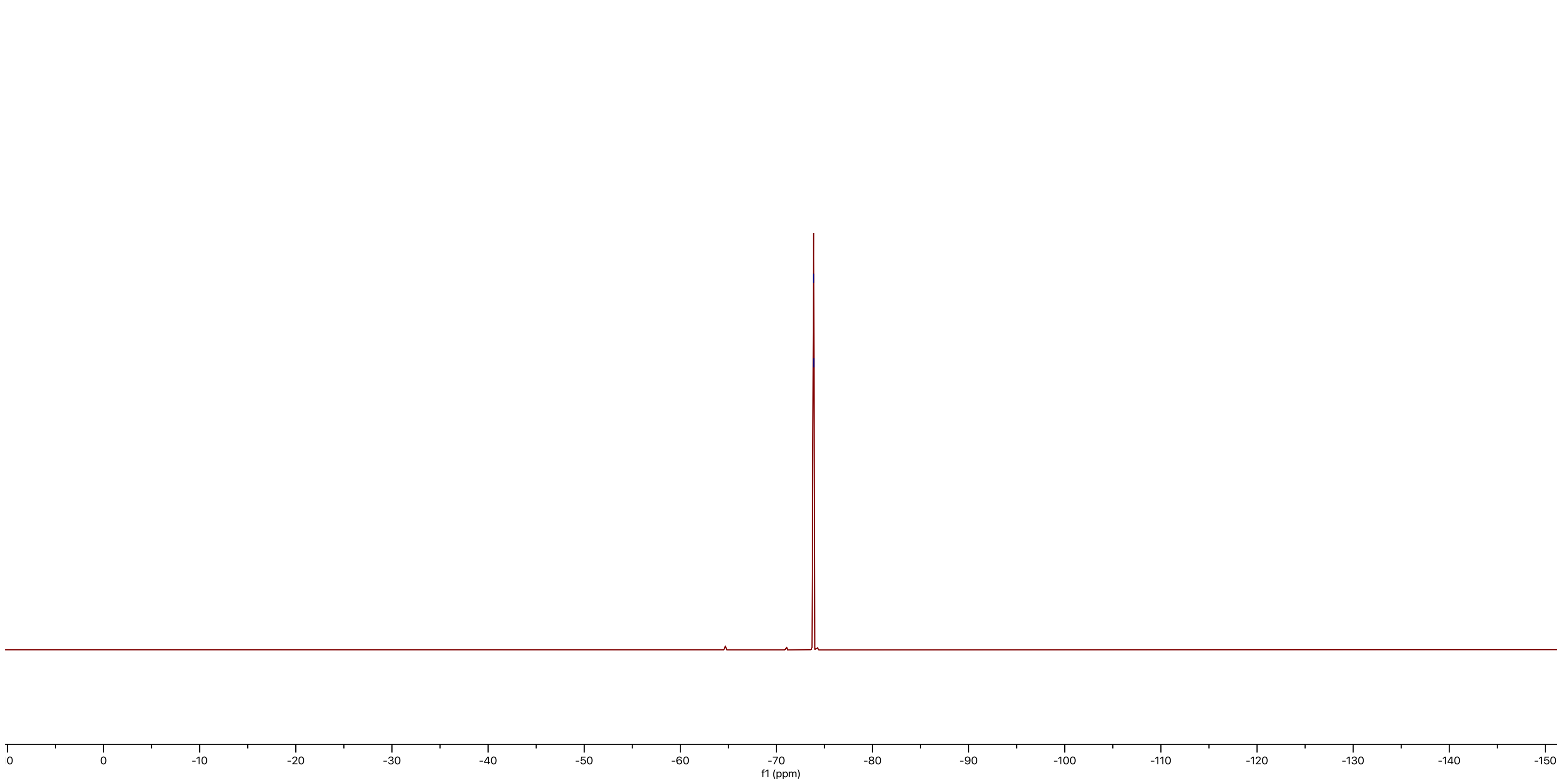

^{1}H NMR of 3f (CDCl$_3$, 500 MHz)

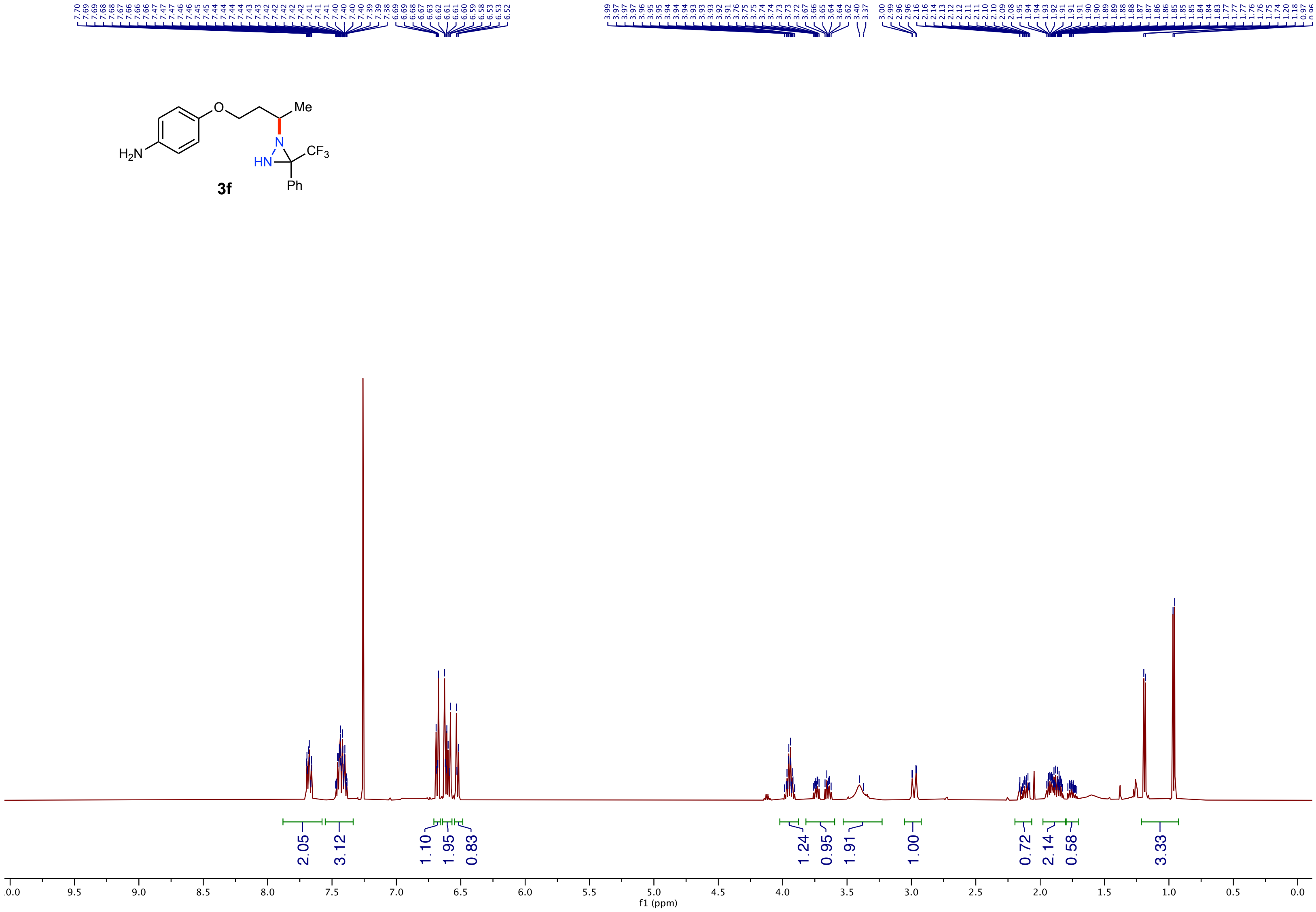

^{13}C NMR of 3f ($CDCl_3$, 126 MHz)

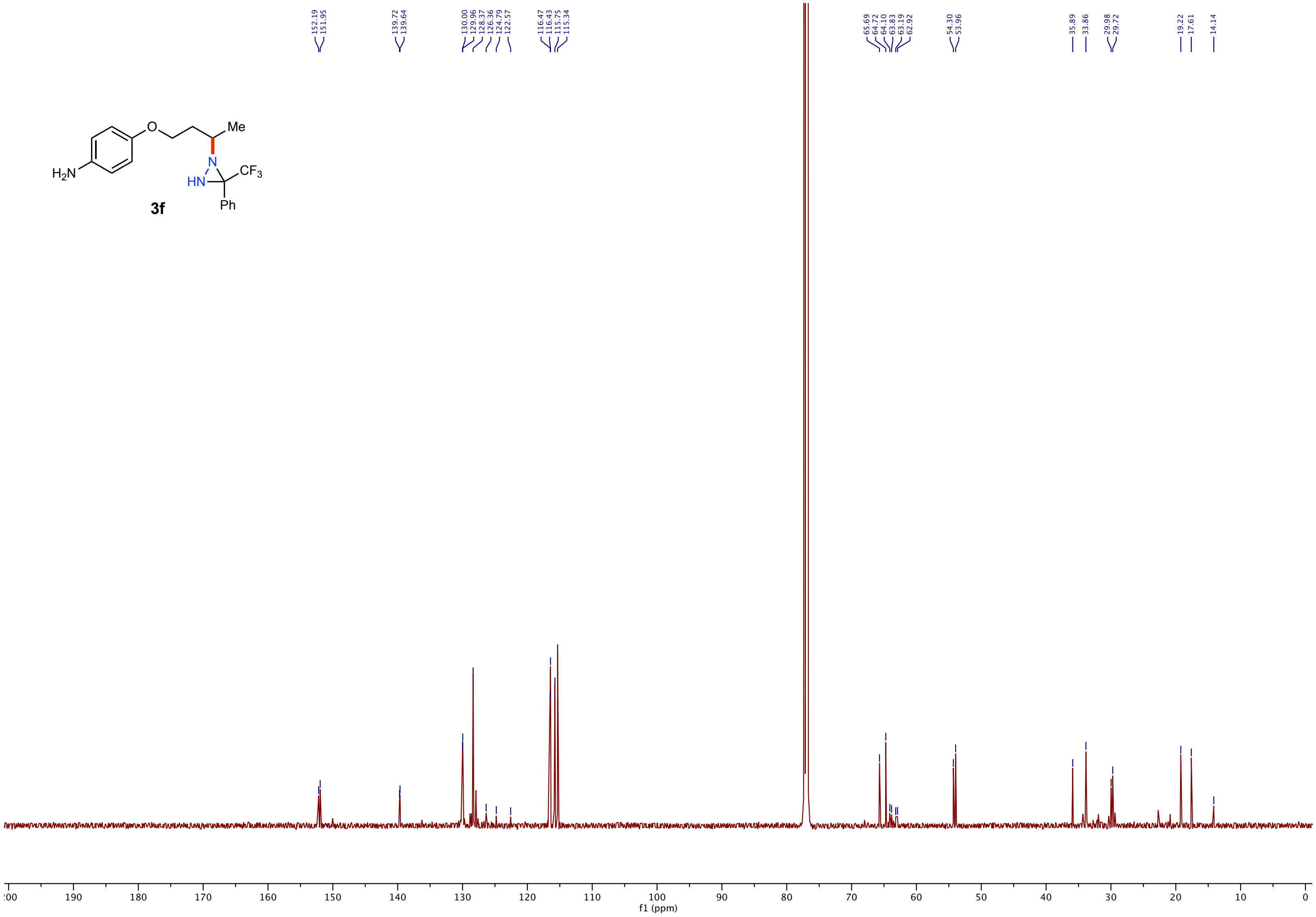

^{19}F NMR of 3f ($CDCl_3$, 471 MHz)

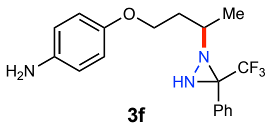

3f

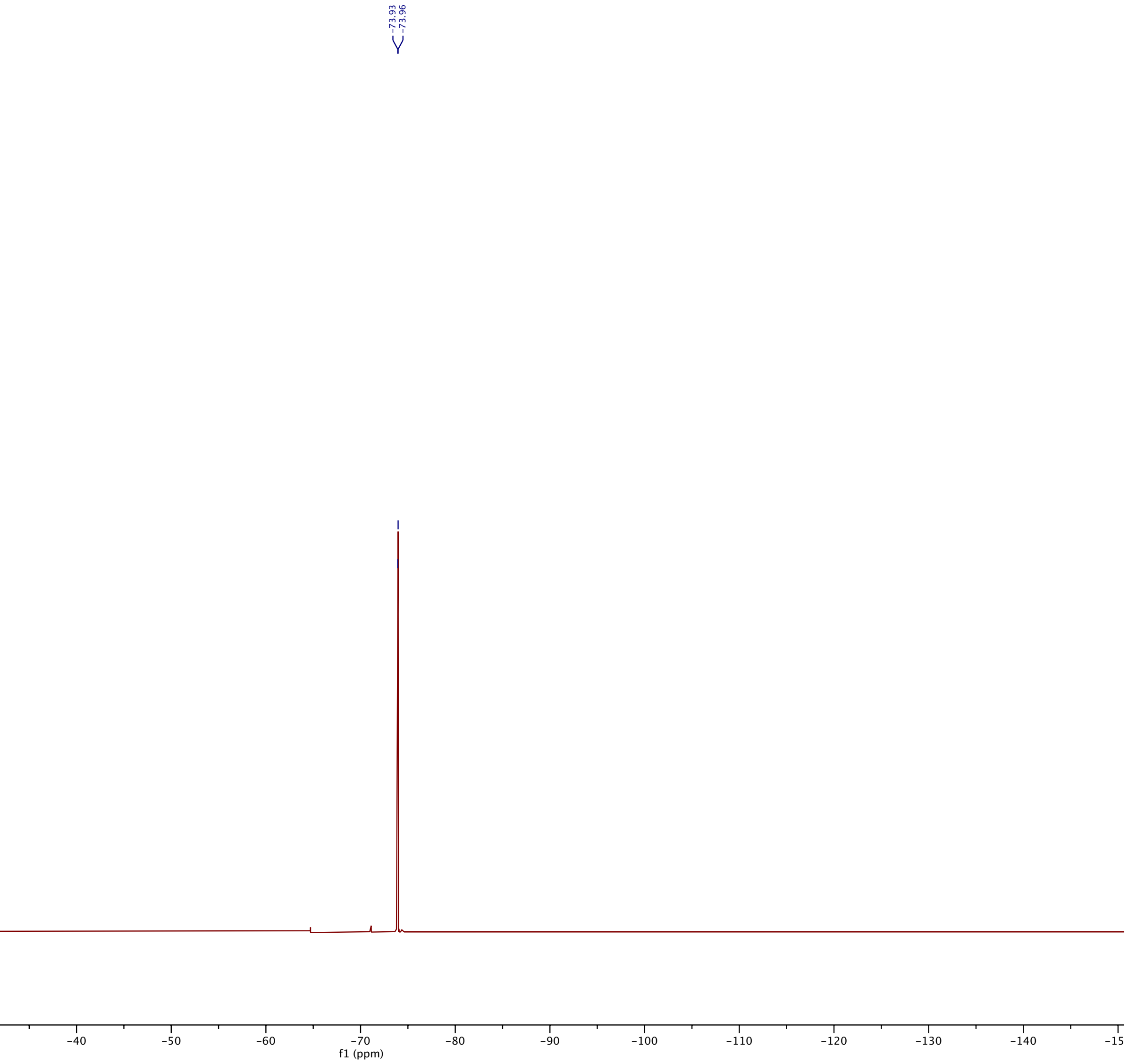

^1H NMR of 3g ($CDCl_3$, 500 MHz)

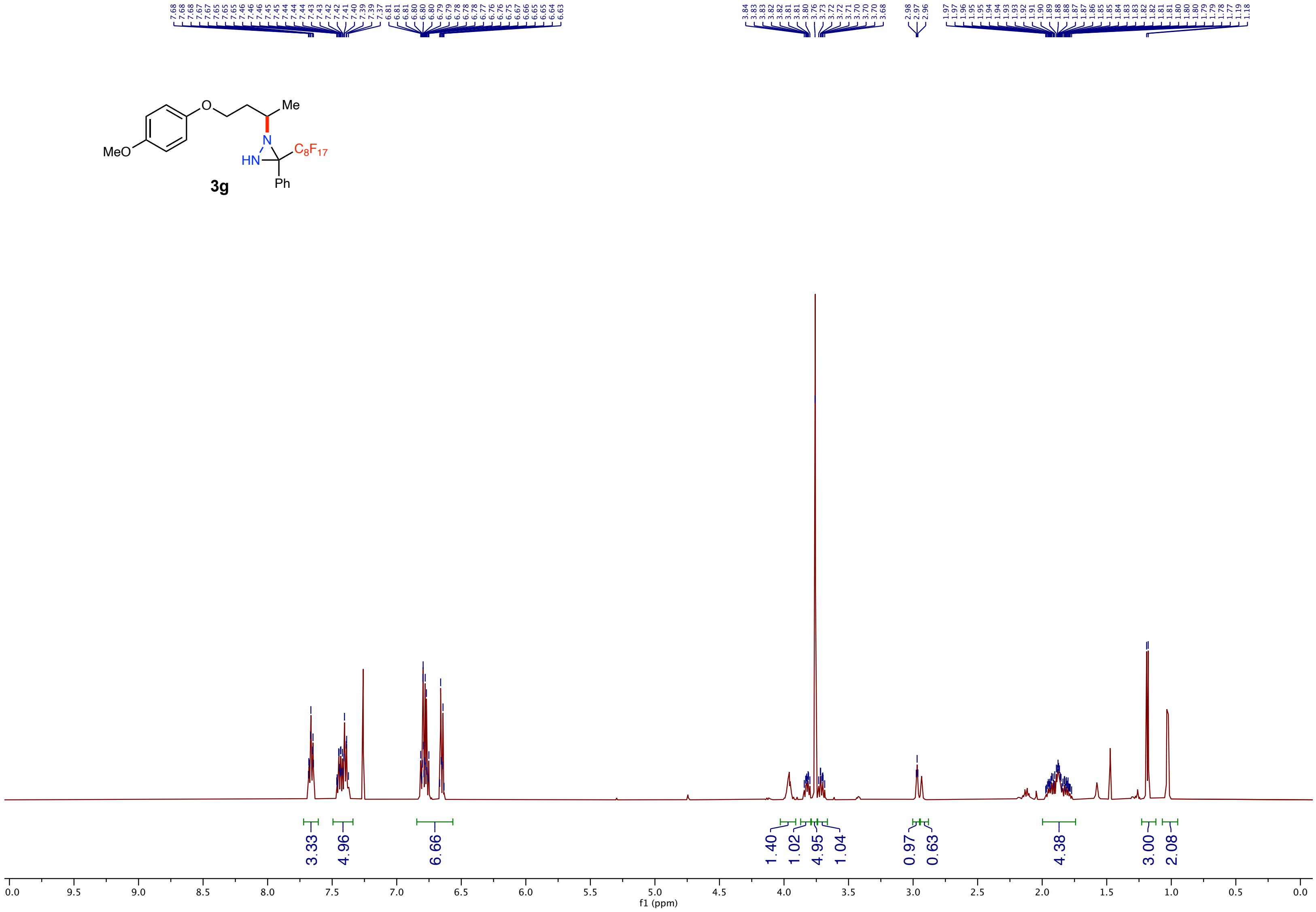

^{13}C NMR of 3g ($CDCl_3$, 126 MHz)

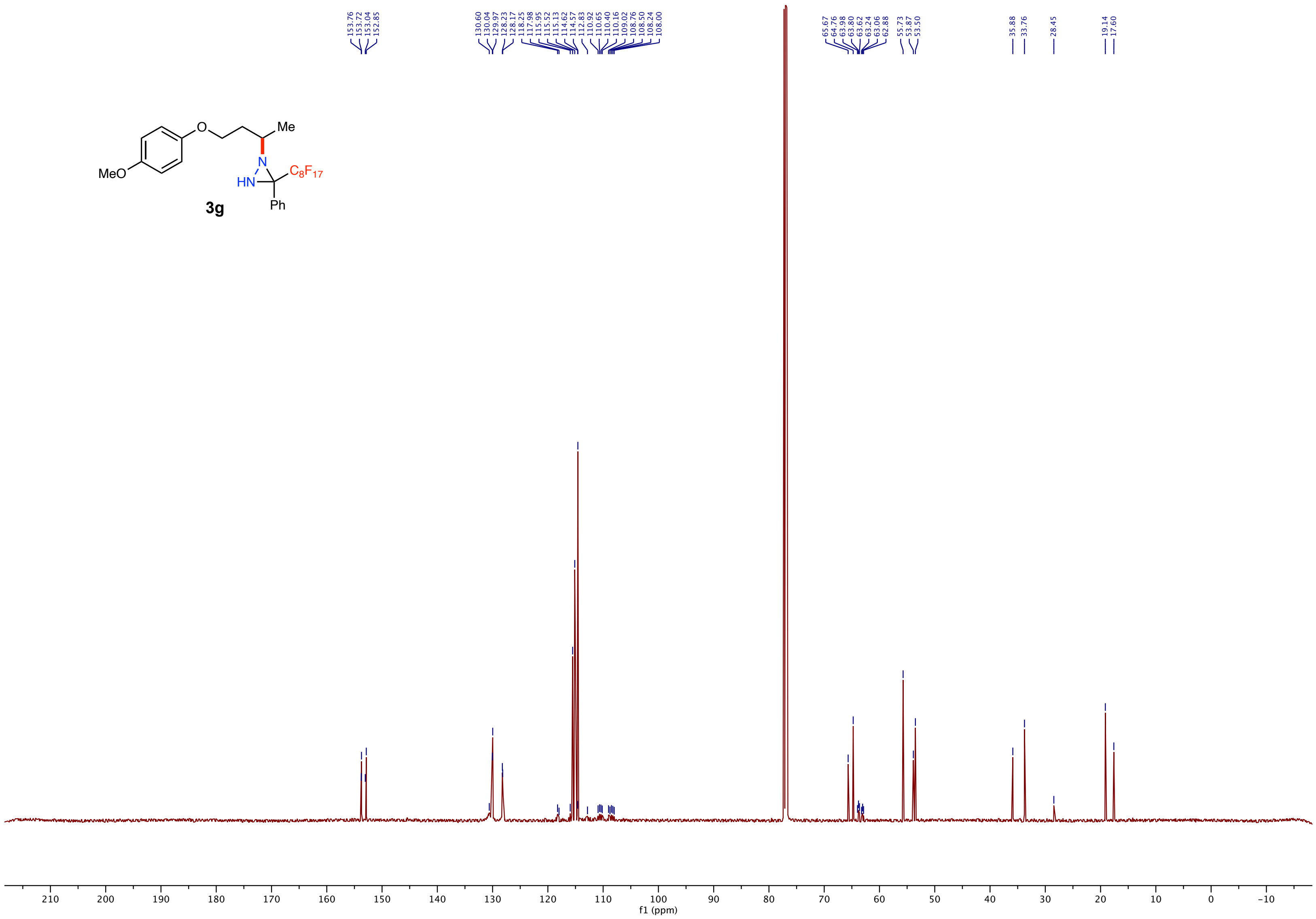

^{19}F NMR of 3g ($CDCl_3$, 471 MHz)

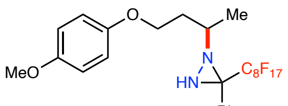

3g

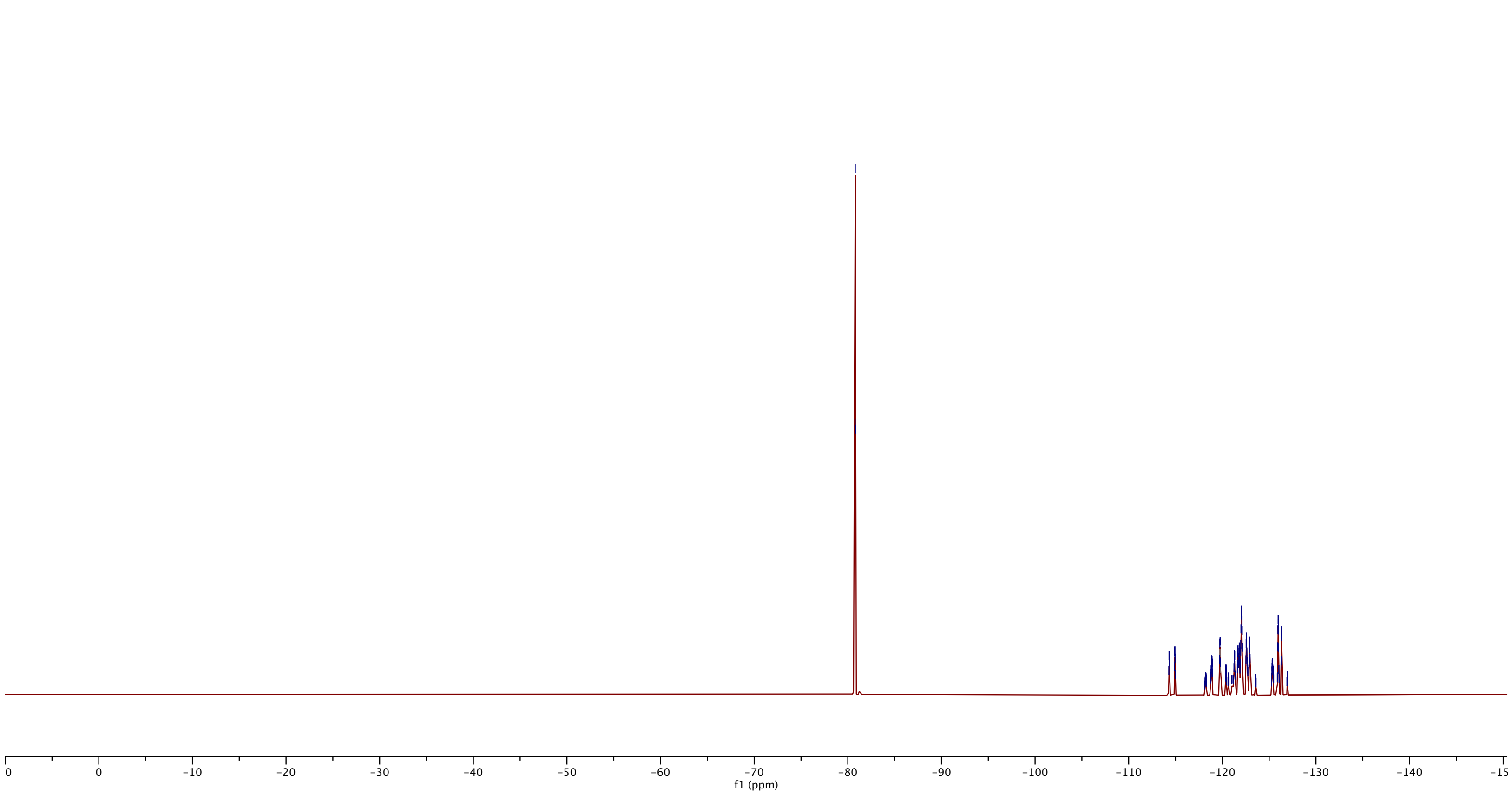

^{1}H NMR of 3h ($CDCl_3$, 500 MHz)

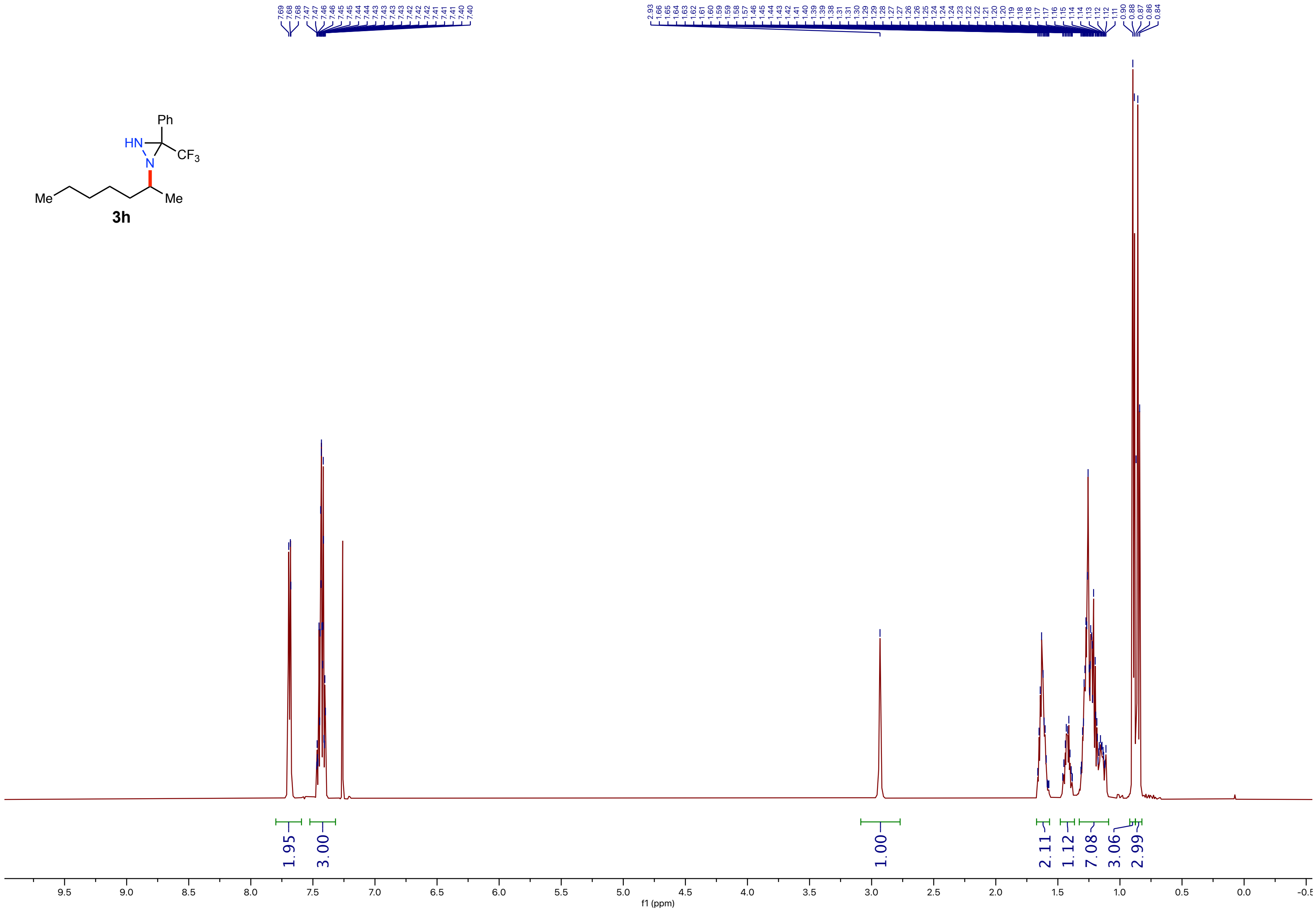

^{13}C NMR of 3h ($CDCl_3$, 126 MHz)

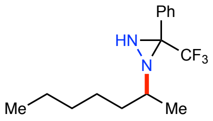

3h

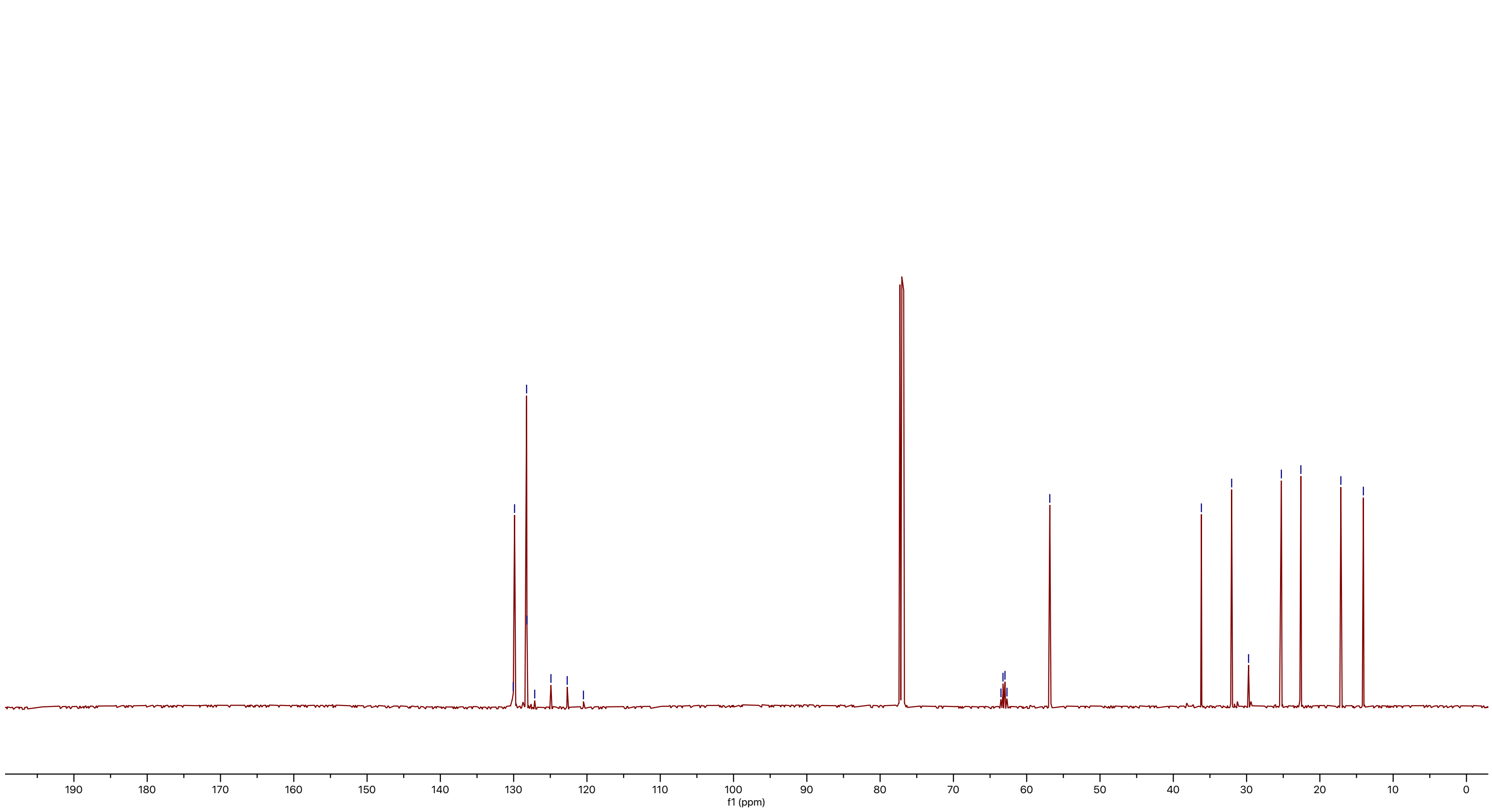

^{19}F NMR of 3h ($CDCl_3$, 471 MHz)

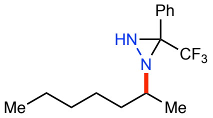

3h

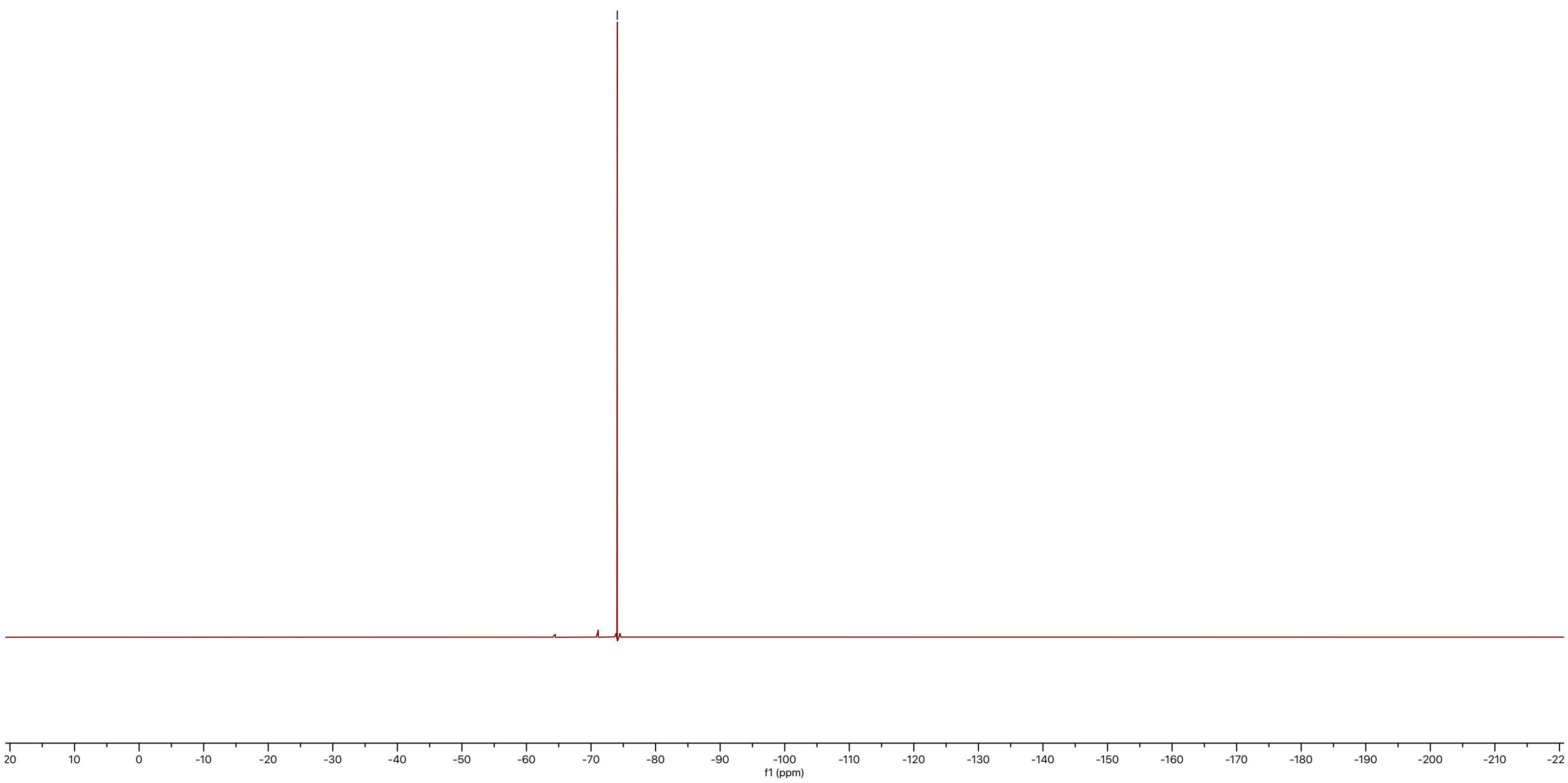

^{1}H NMR of 3i ($CDCl_3$, 500 MHz)

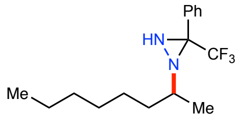

3i

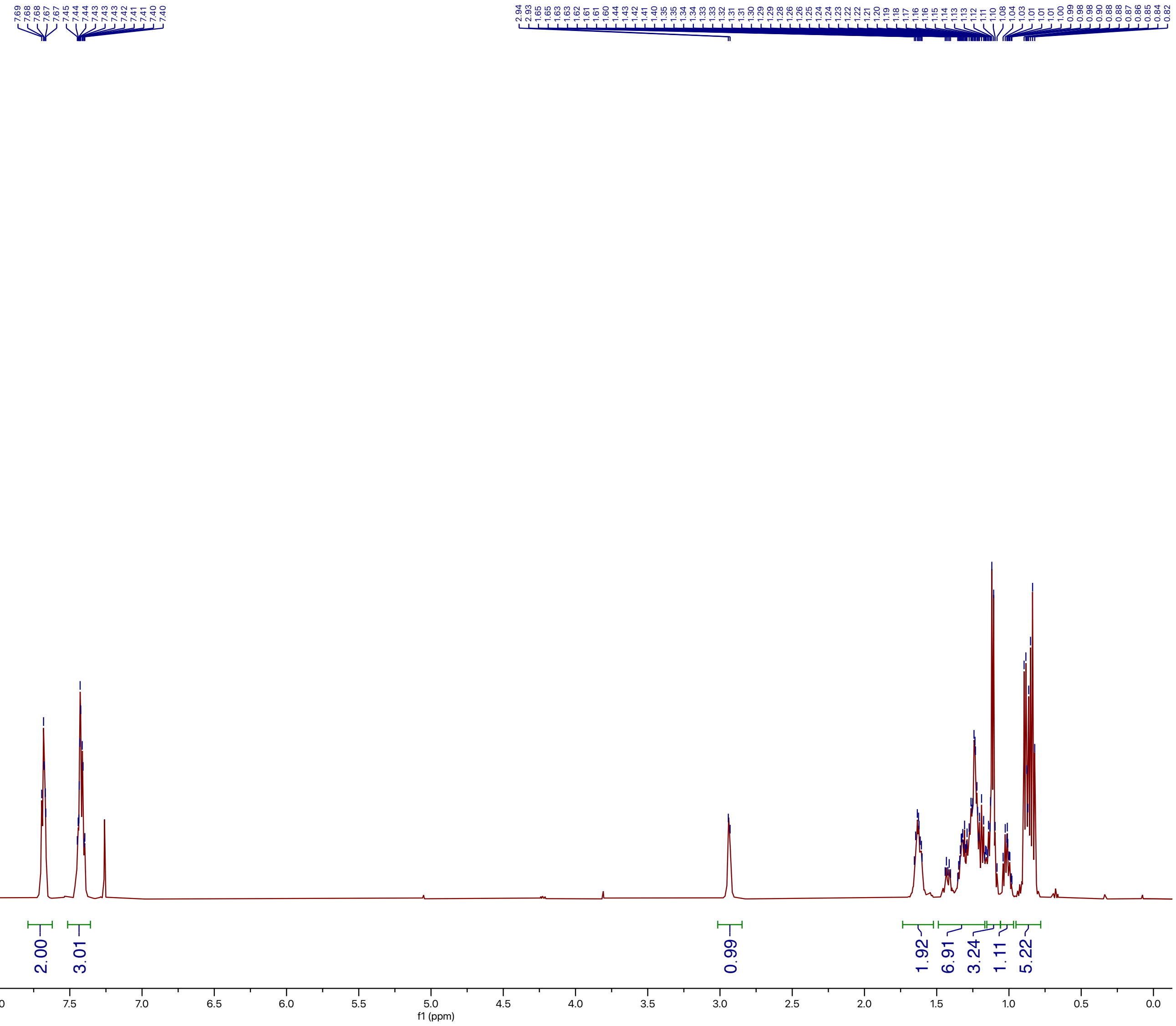

^{13}C NMR of 3i ($CDCl_3$, 126 MHz)

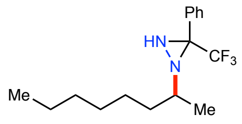

3i

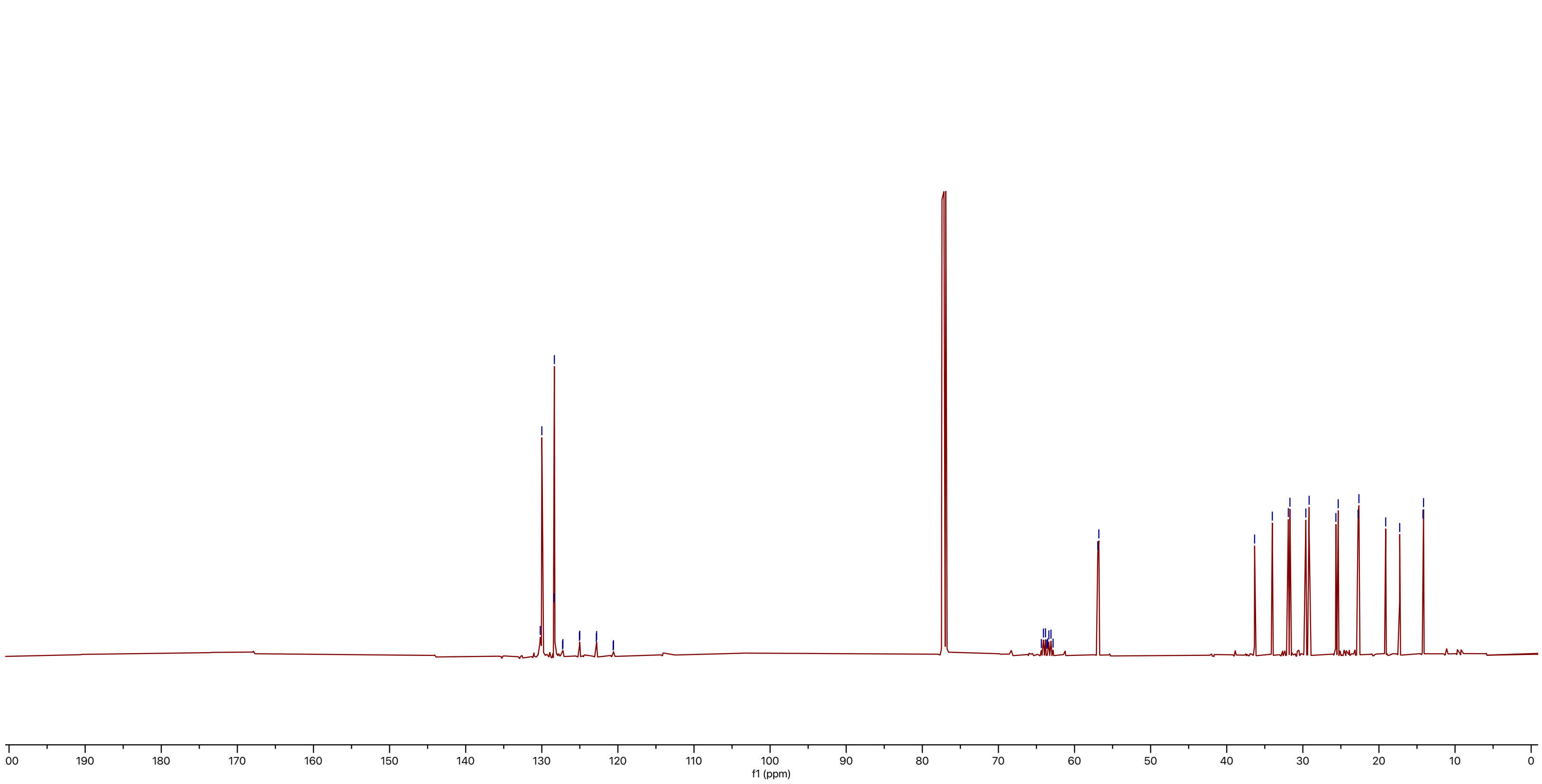

^{19}F NMR of 3i ($CDCl_3$, 471 MHz)

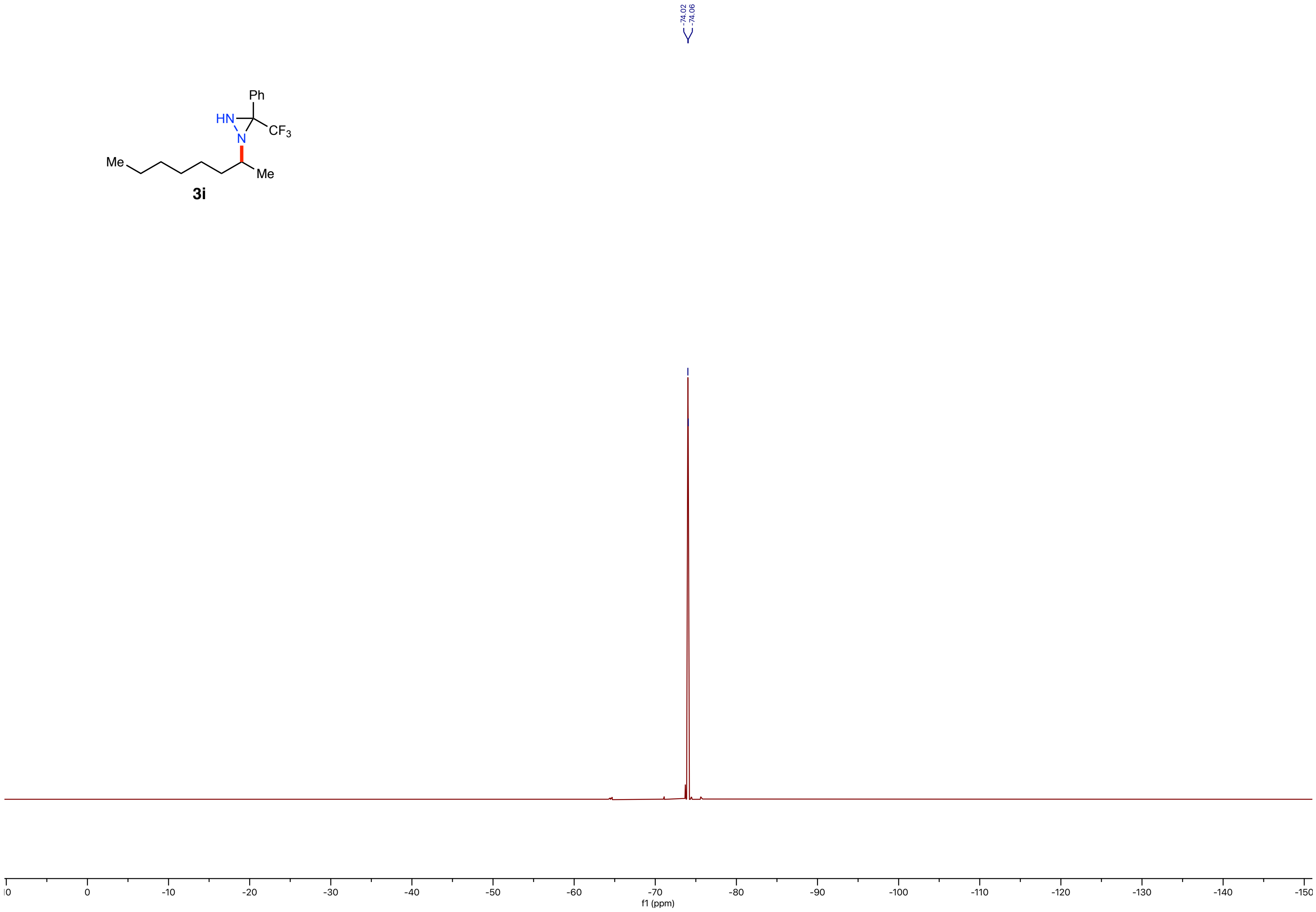

^{1}H NMR of 3j ($CDCl_3$, 500 MHz)

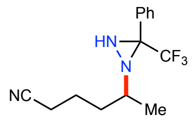
3j

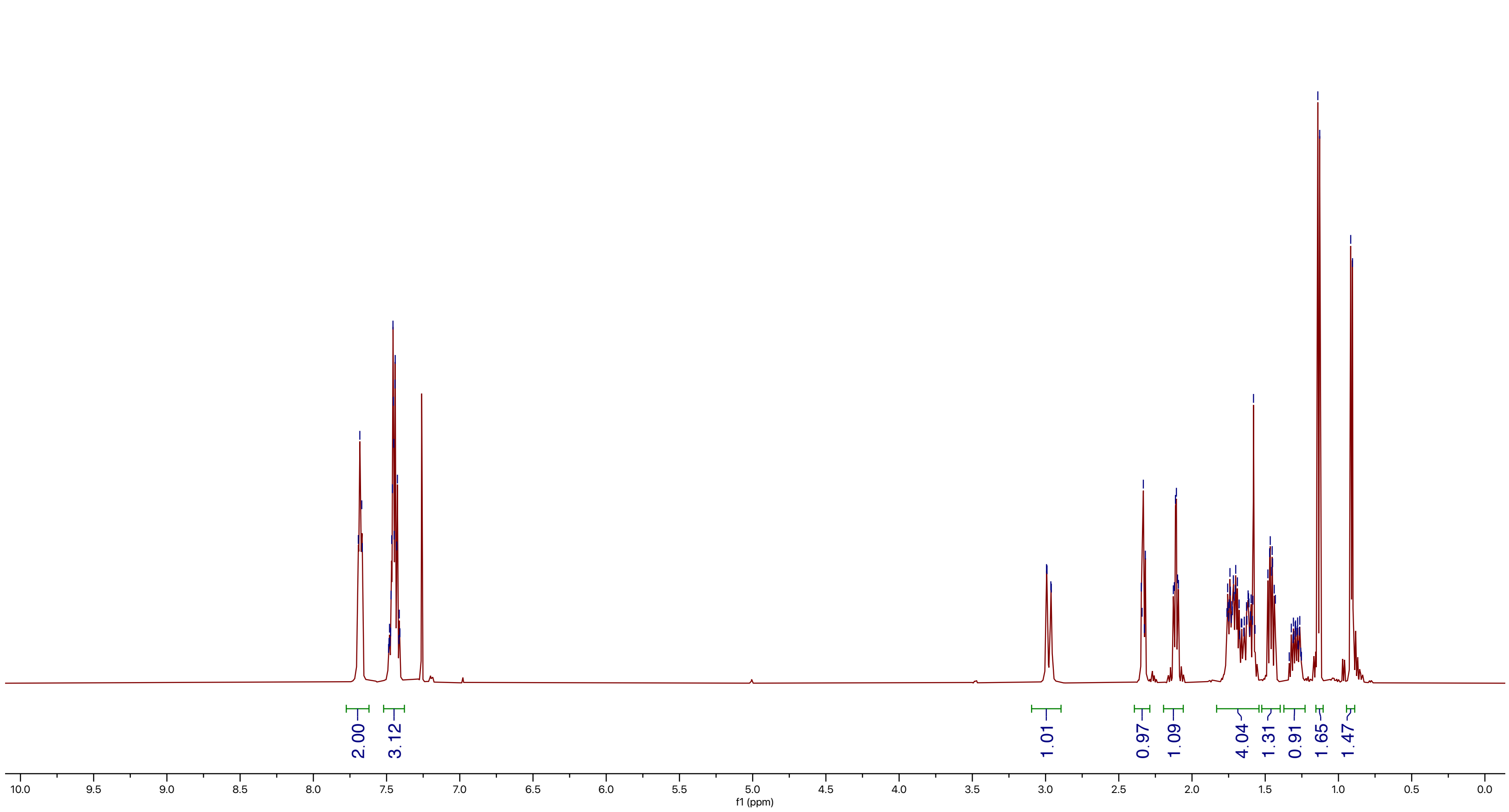

^{13}C NMR of 3j ($CDCl_3$, 126 MHz)

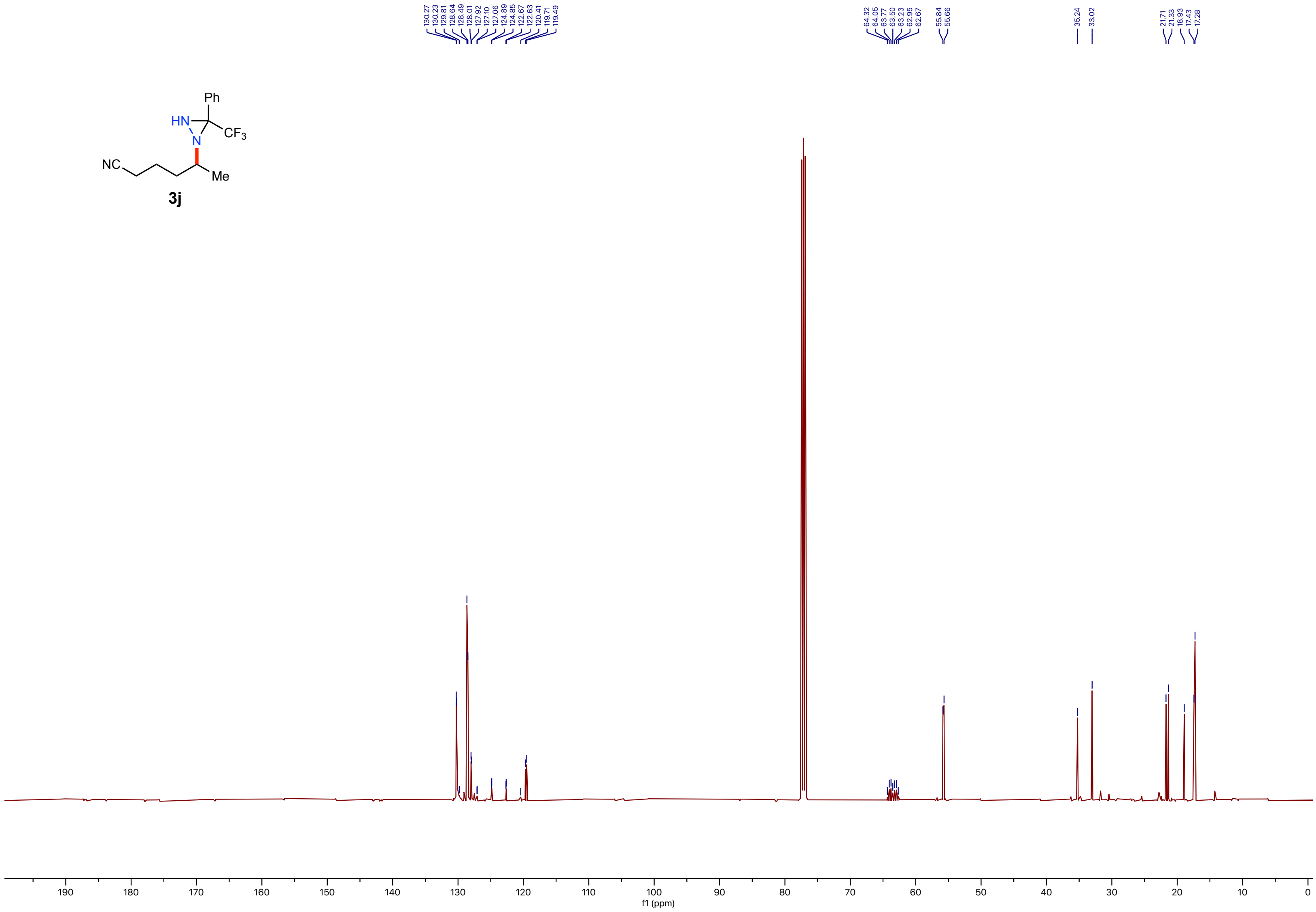

^{19}F NMR of 3j ($CDCl_3$, 471 MHz)

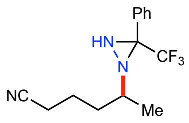

3j

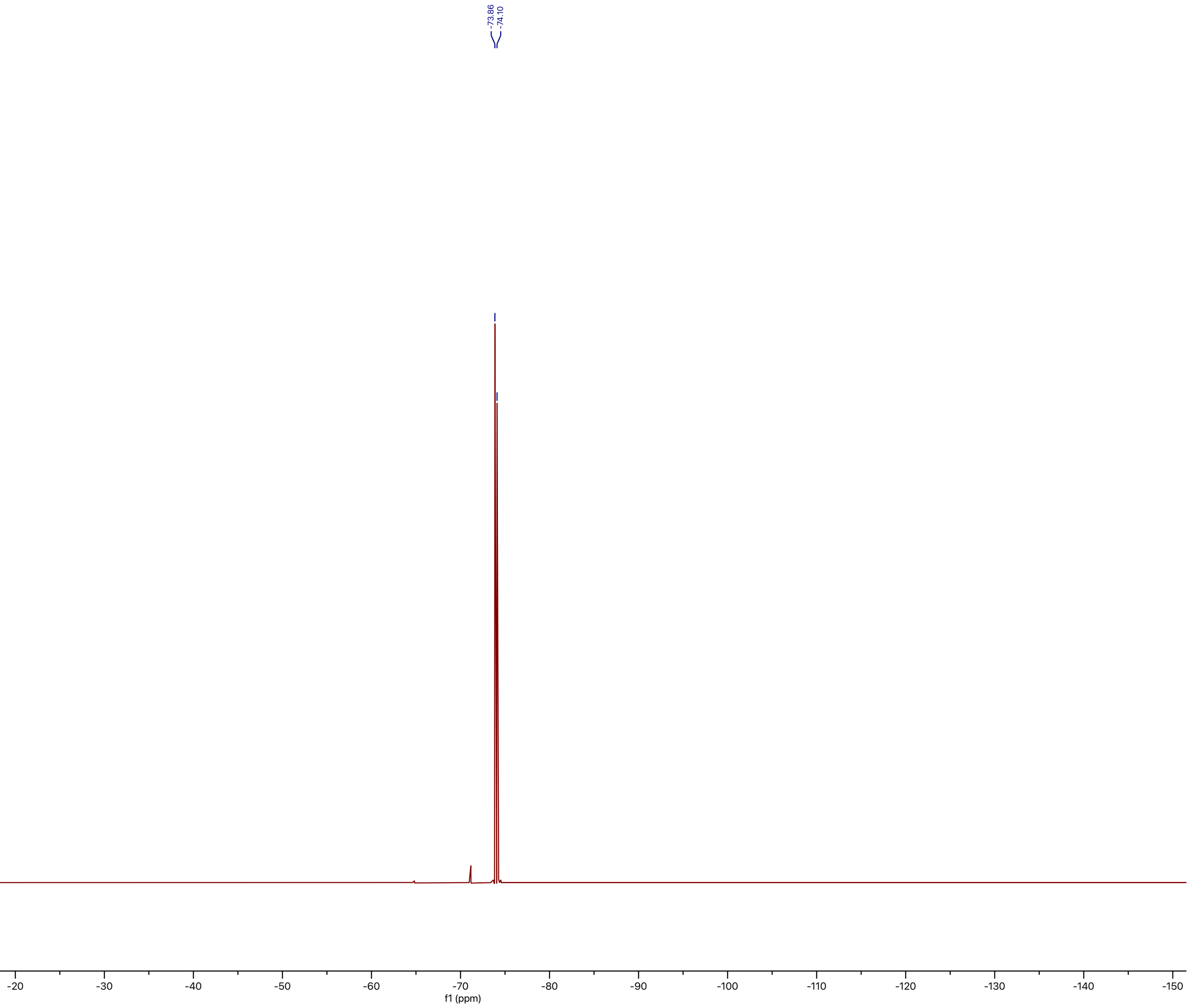

^{1}H NMR of 3k ($CDCl_3$, 500 MHz)

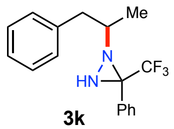

3k

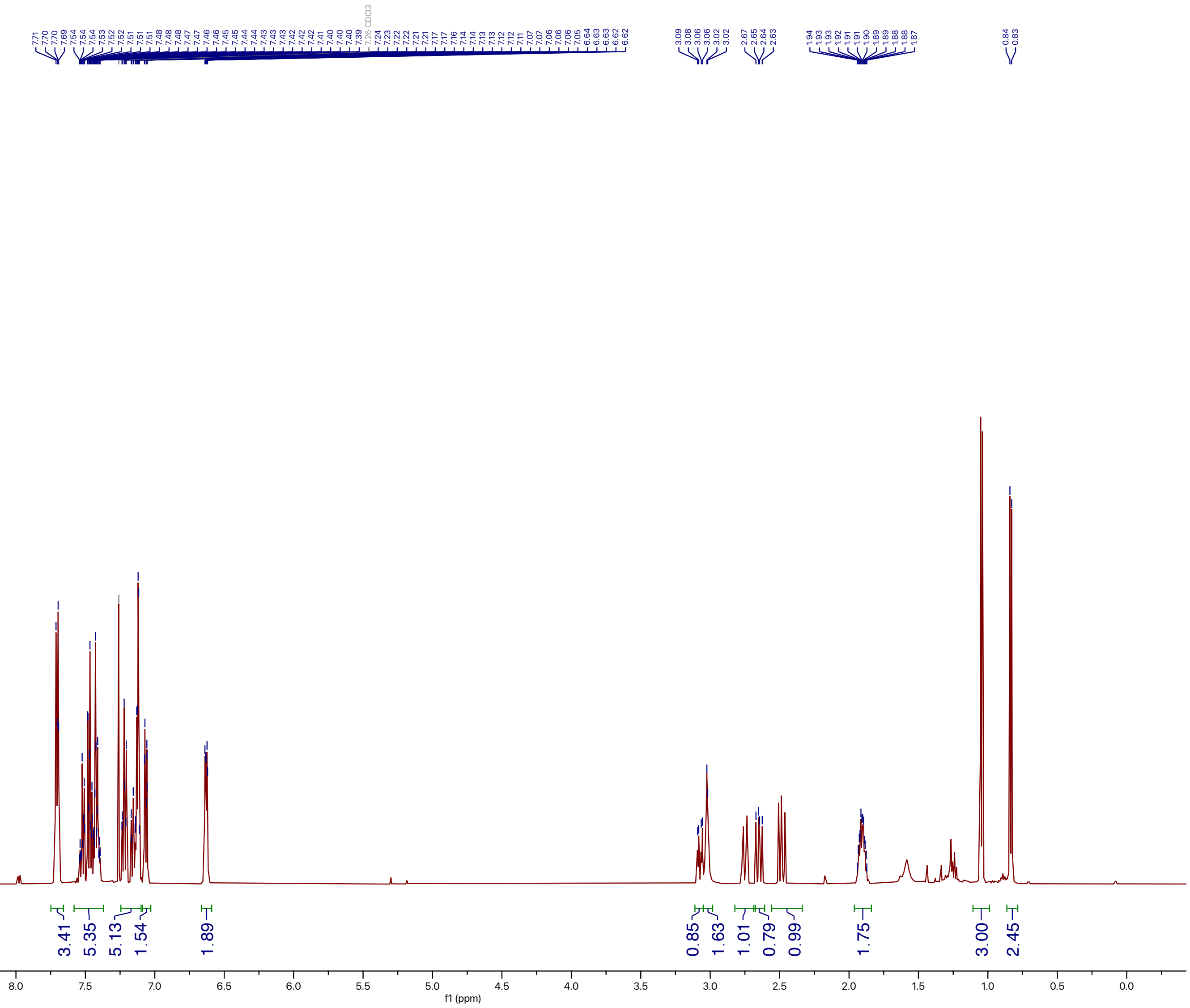

^{13}C NMR of 3k ($CDCl_3$, 126 MHz)

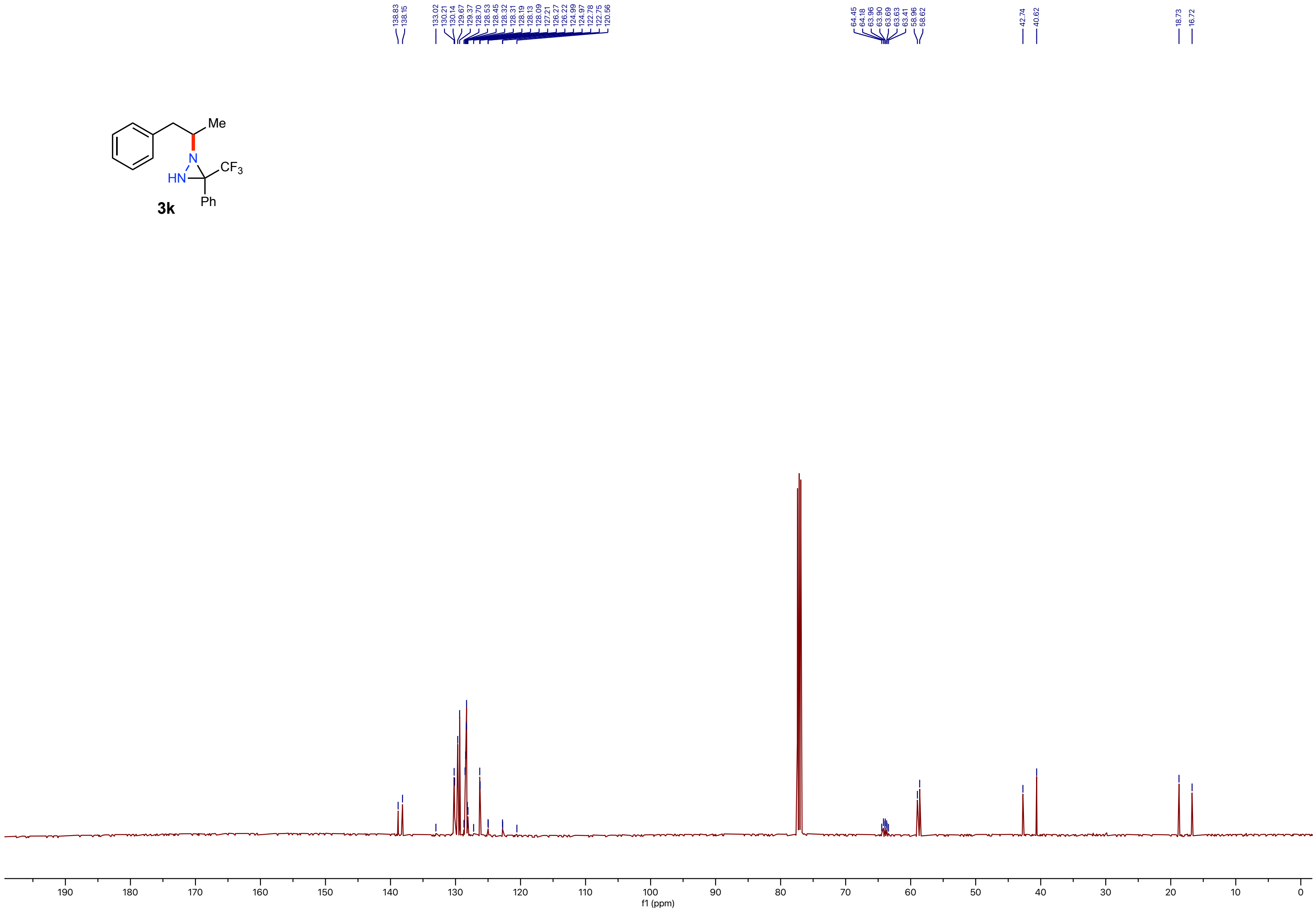

^{19}F NMR of 3k ($CDCl_3$, 471 MHz)

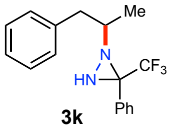

3k

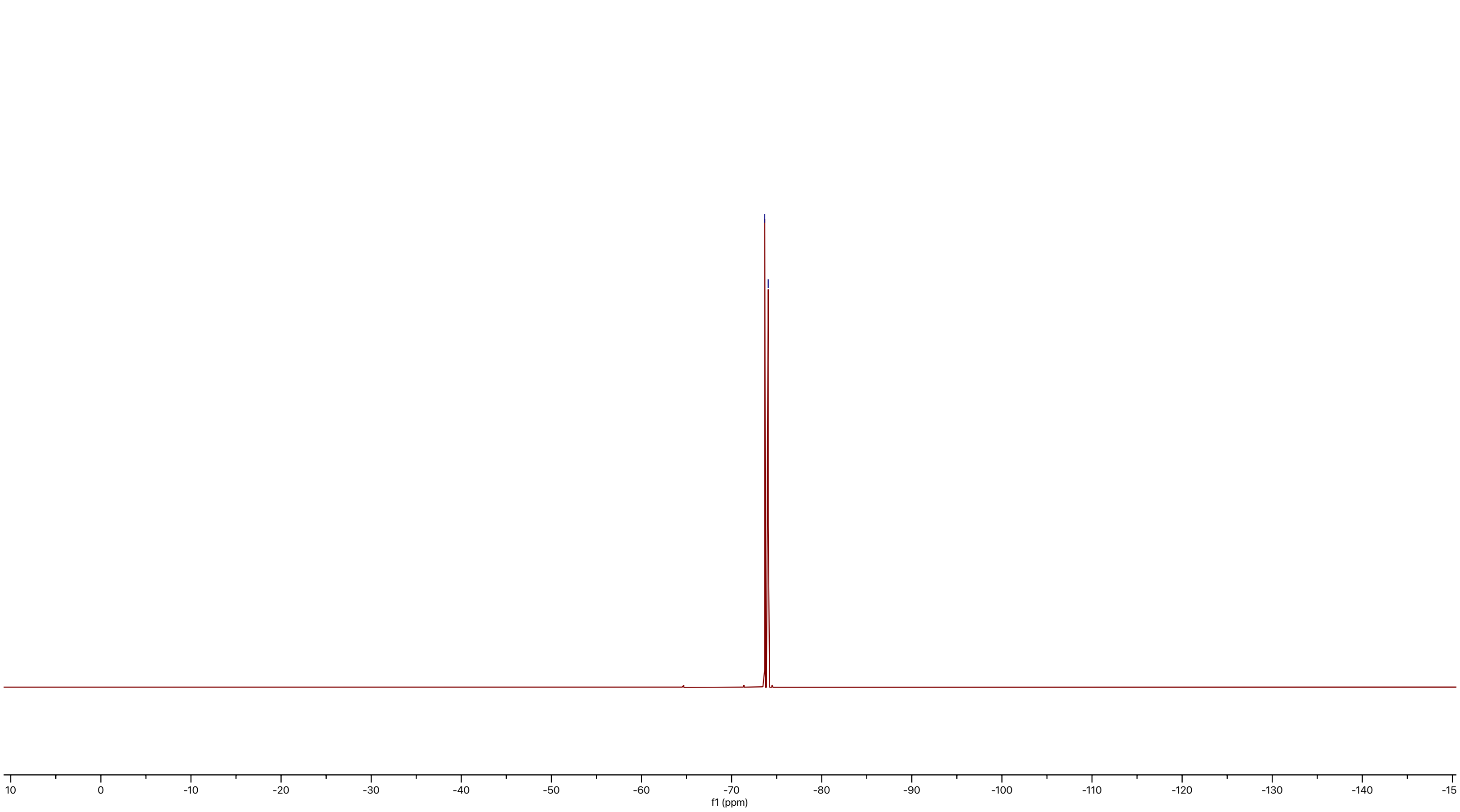

^{1}H NMR of 3l ($CDCl_3$, 500 MHz)

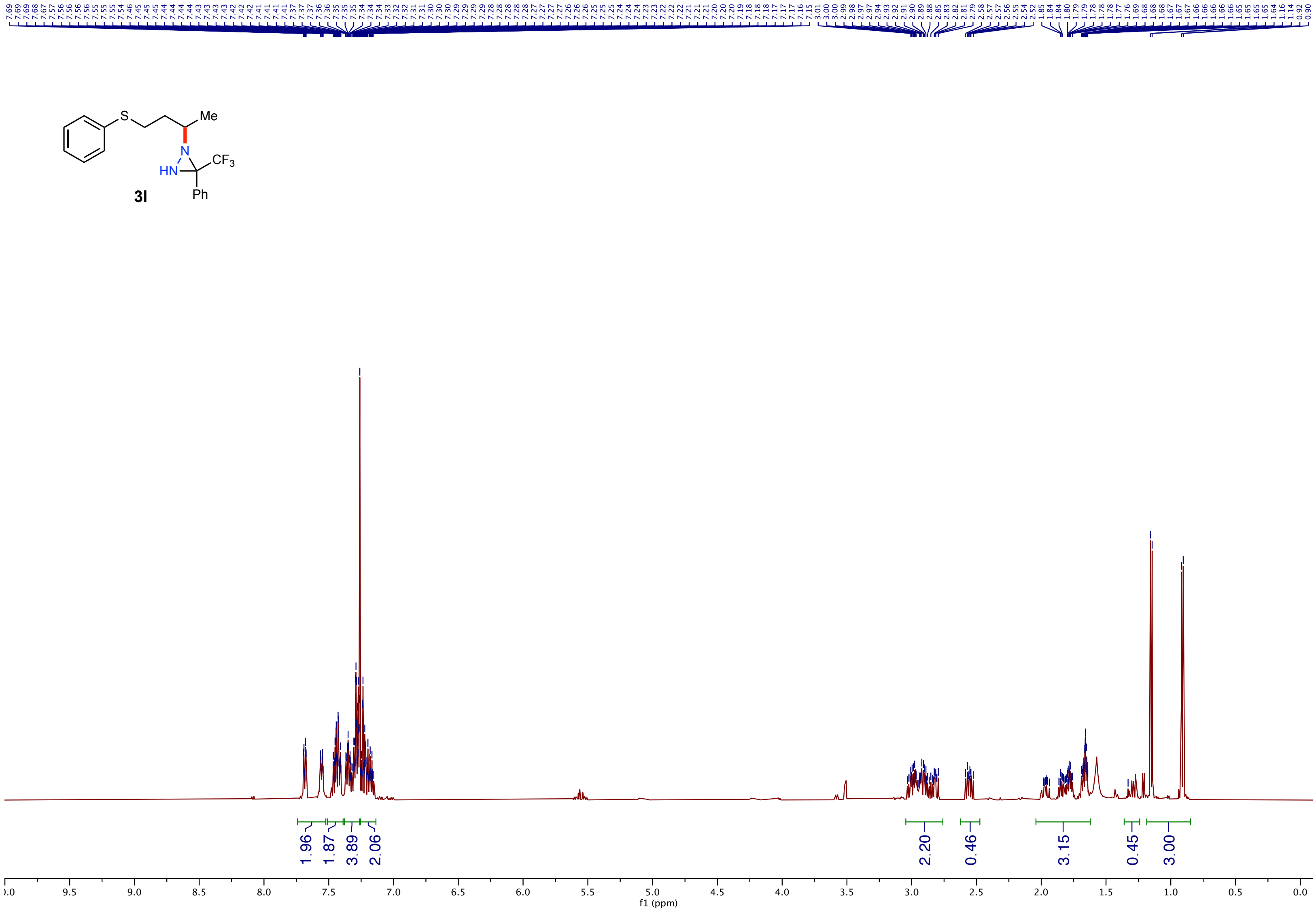

^{13}C NMR of 3l ($CDCl_3$, 126 MHz)

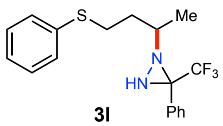

3l

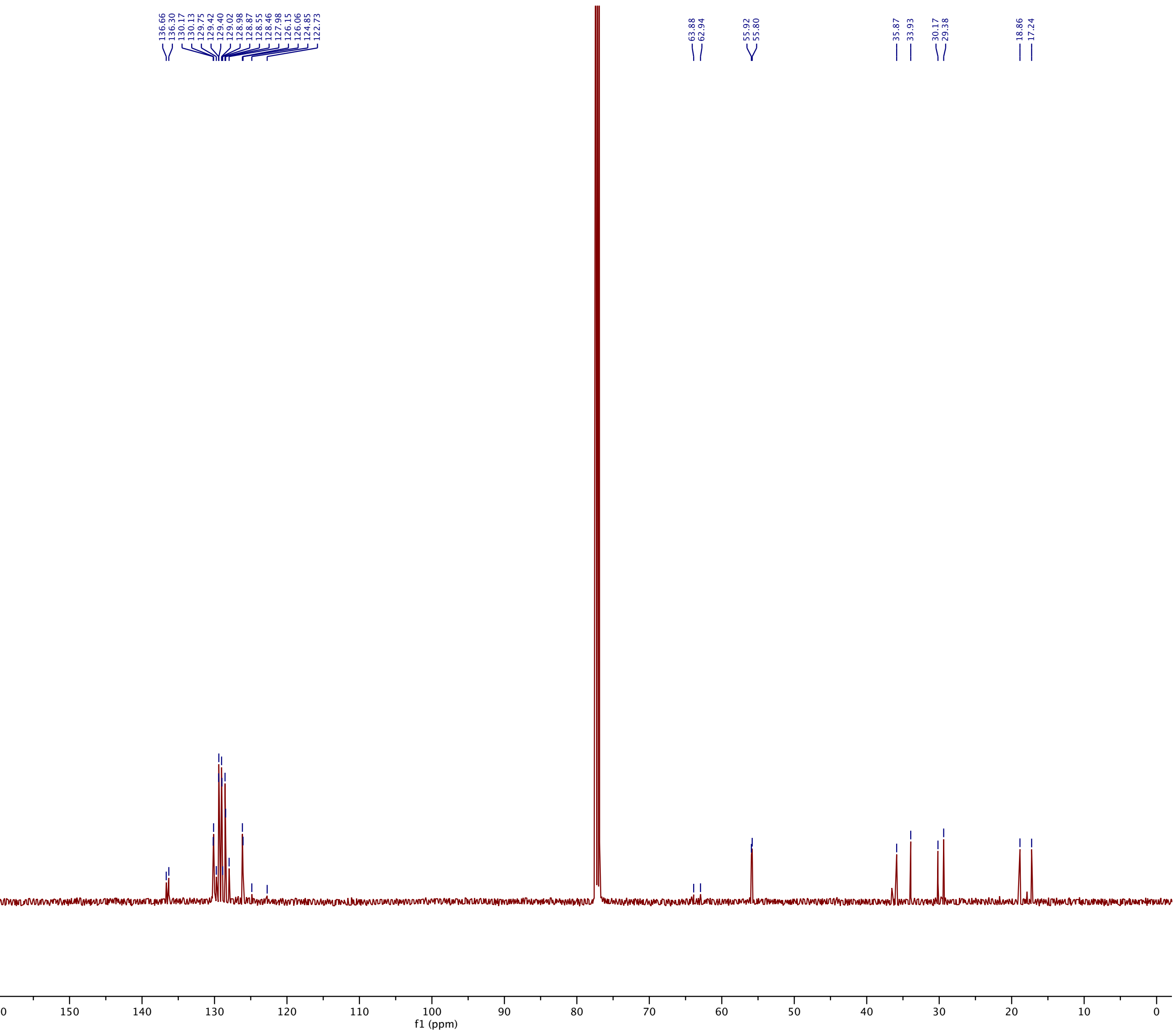

^{19}F NMR of 3l ($CDCl_3$, 471 MHz)

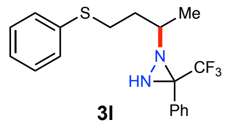

3l

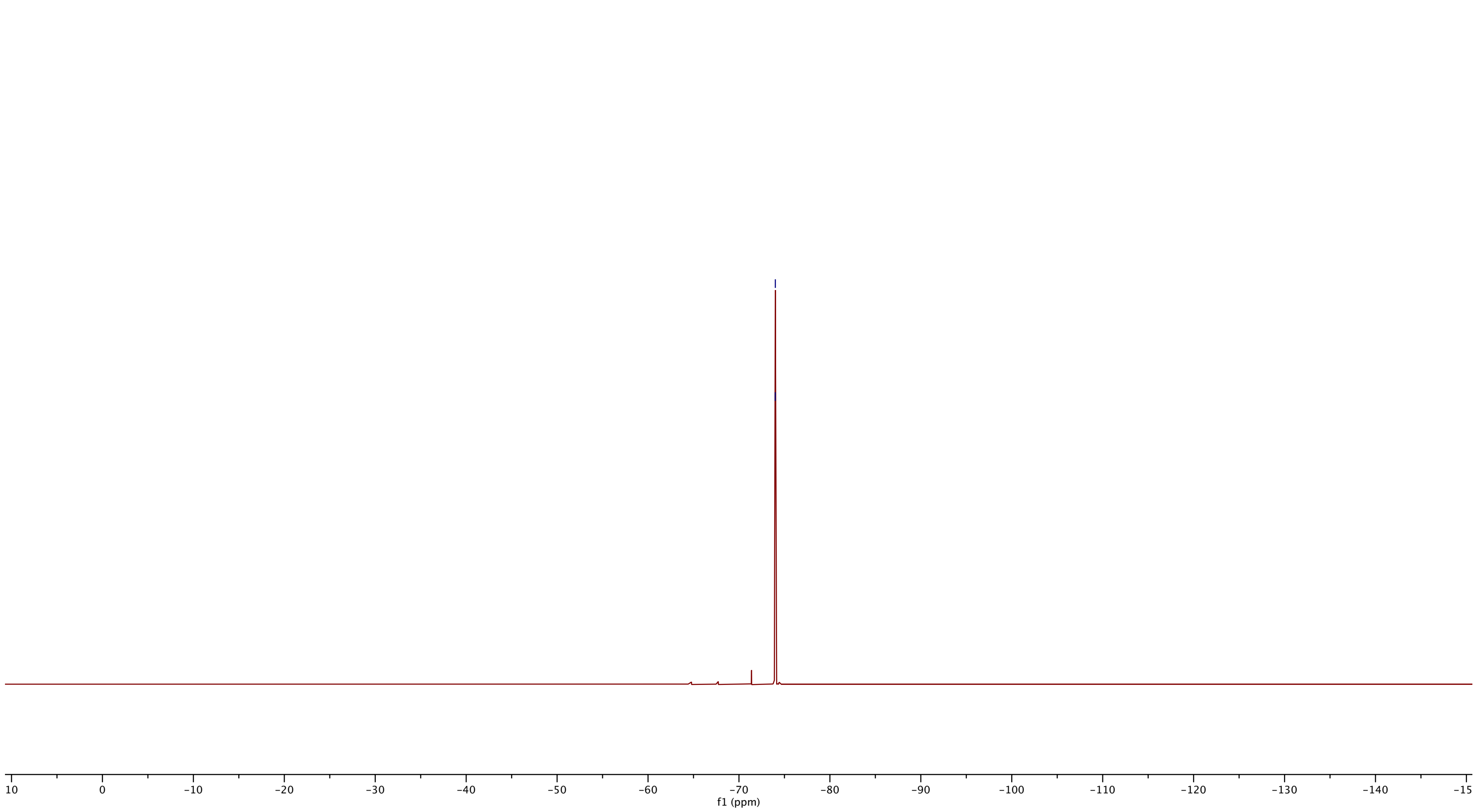

^{1}H NMR of 3m ($CDCl_3$, 500 MHz)

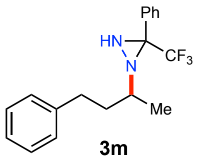

3m

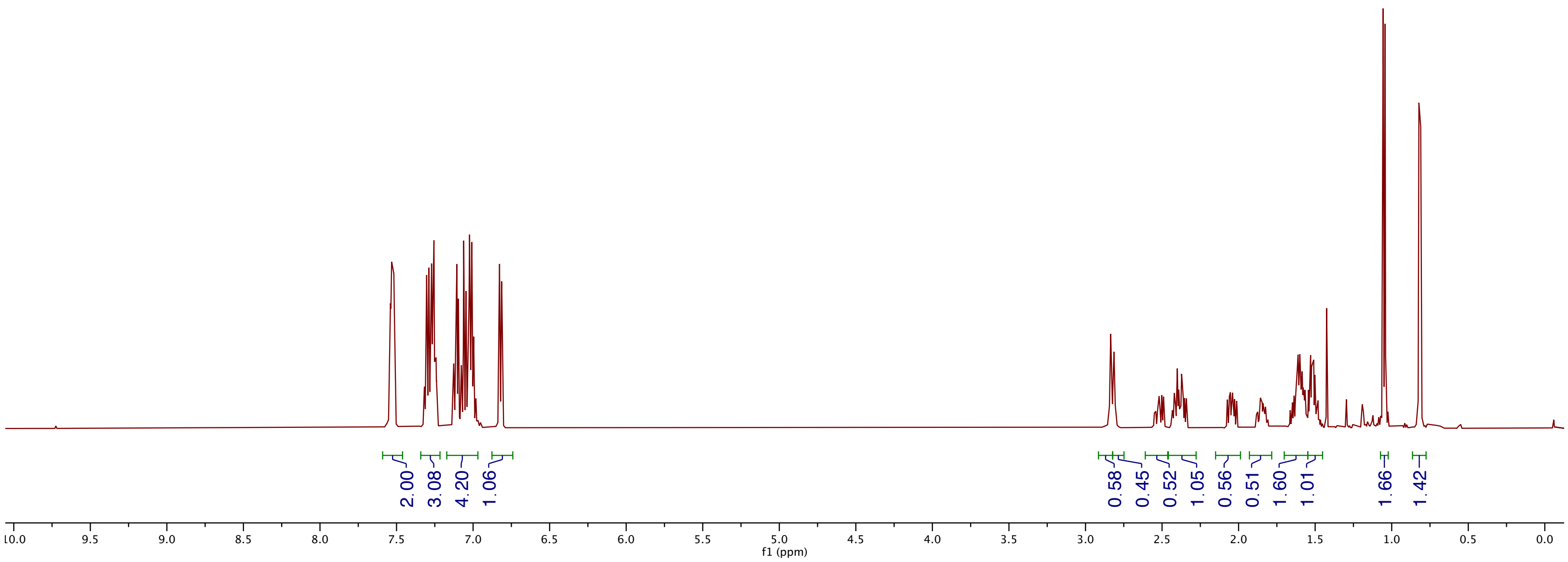

^{13}C NMR of 3m ($CDCl_3$, 126 MHz)

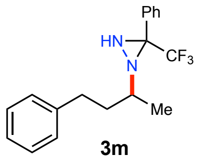

3m

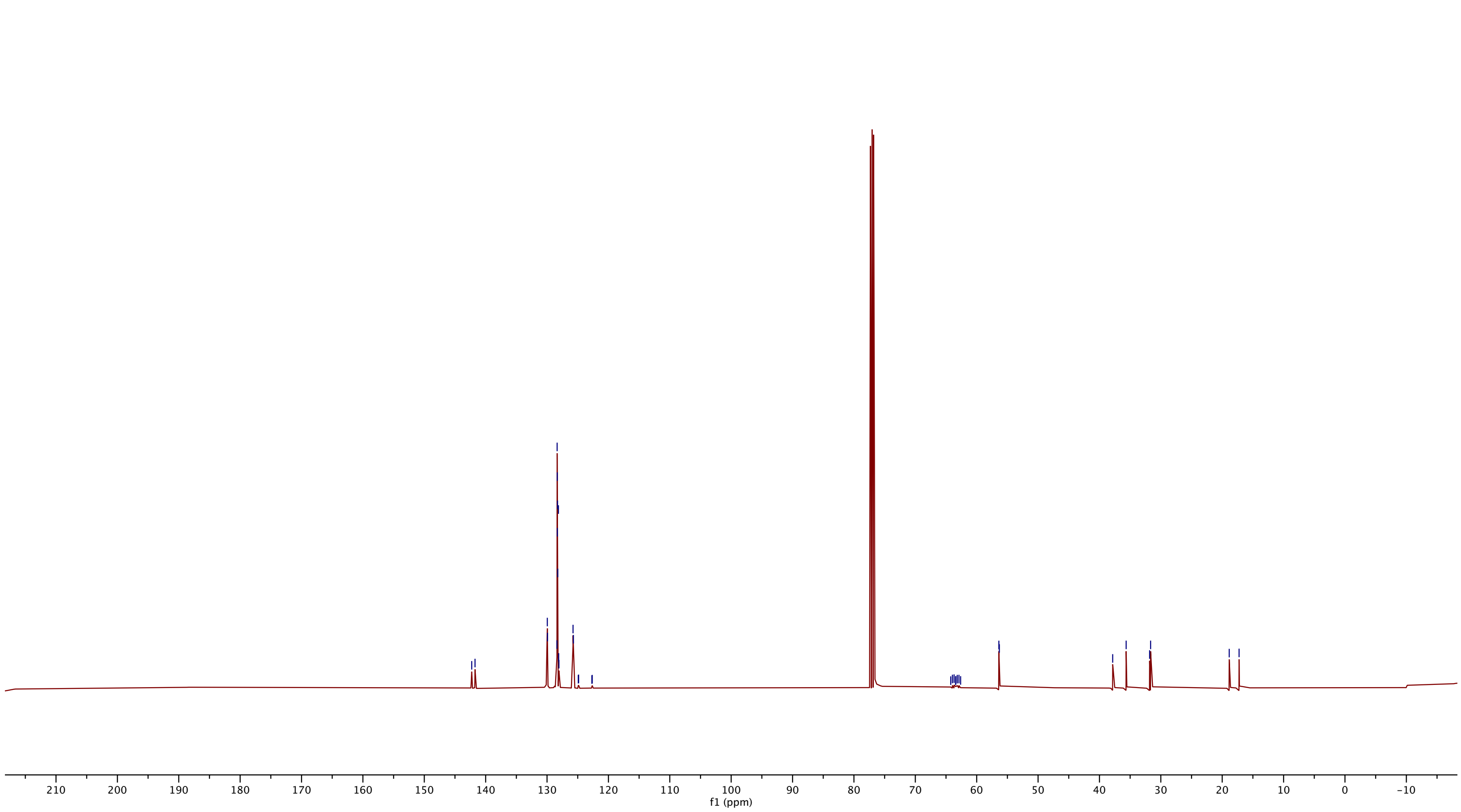

^{19}F NMR of 3m ($CDCl_3$, 471 MHz)

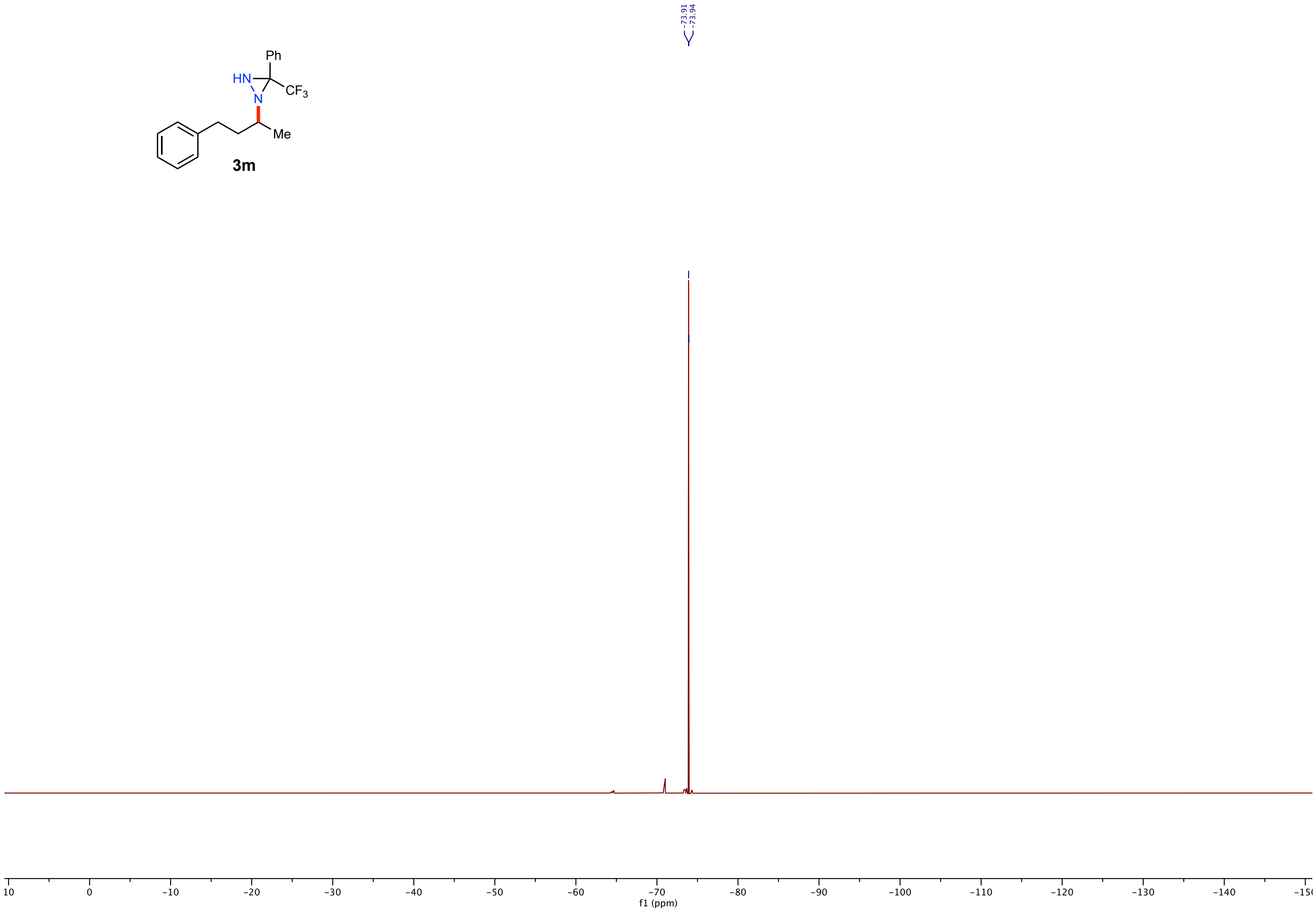

^{1}H NMR of 3n ($CDCl_3$, 500 MHz)

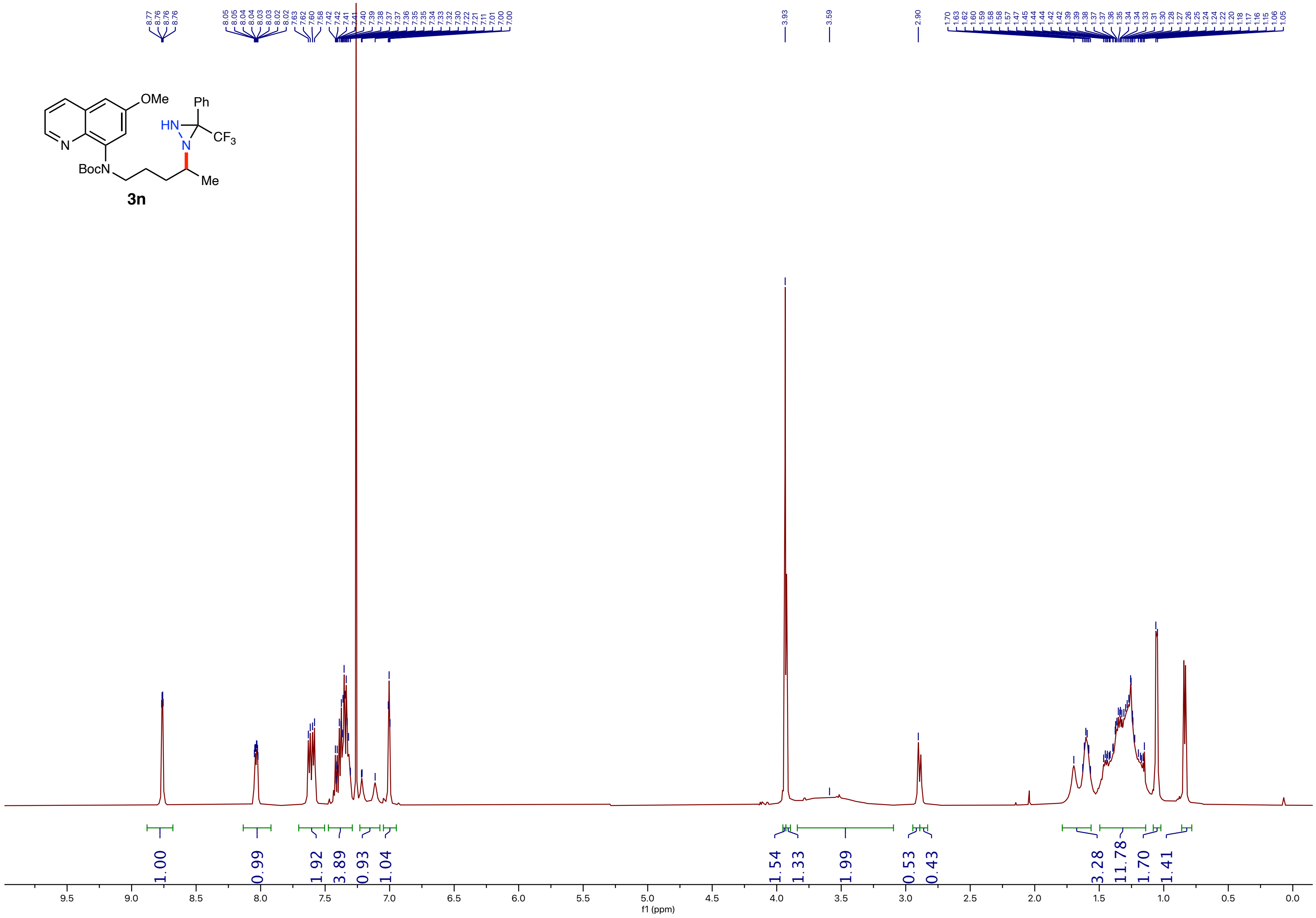

^{13}C NMR of 3n ($CDCl_3$, 126 MHz)

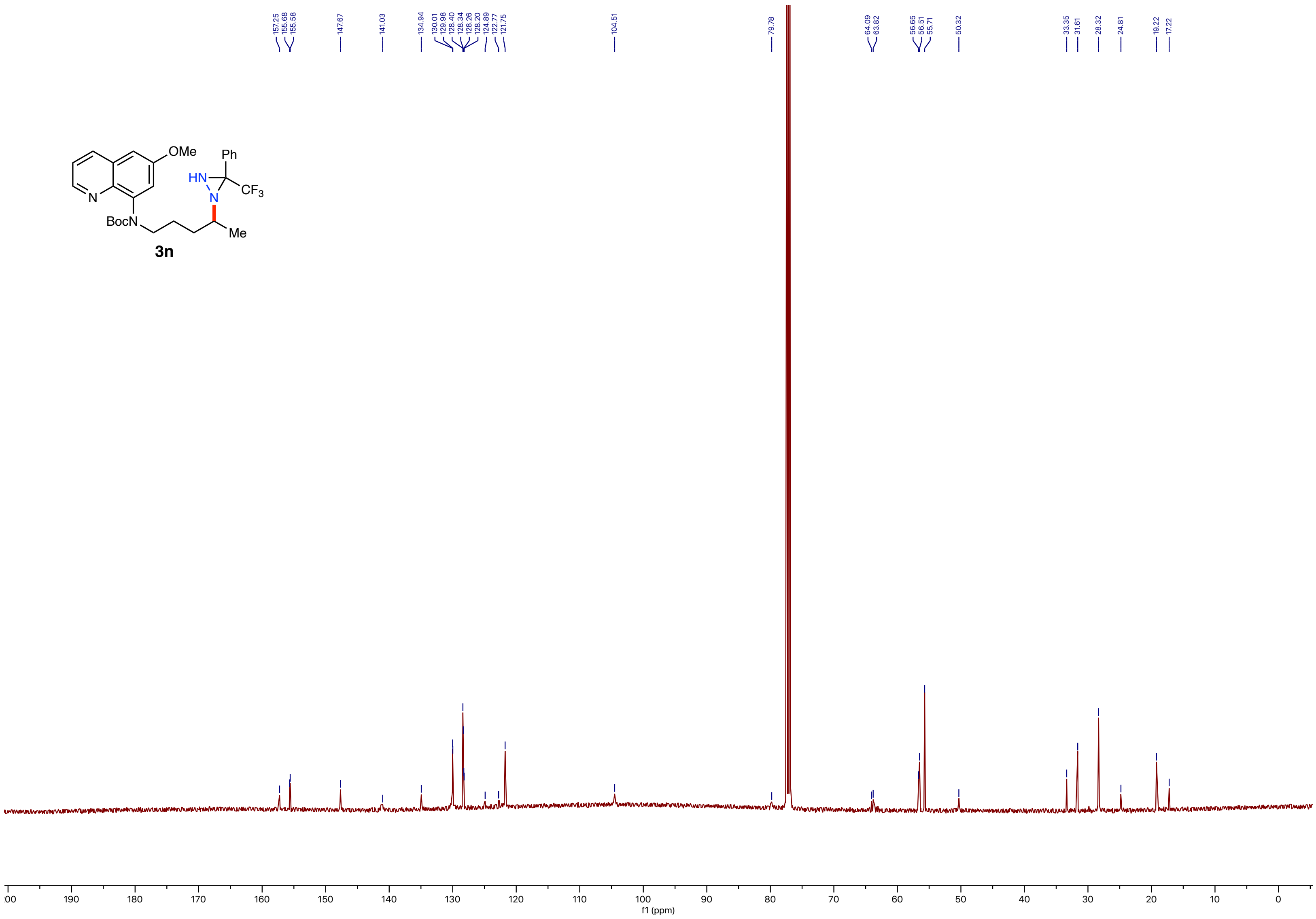

^{19}F NMR of 3n ($CDCl_3$, 471 MHz)

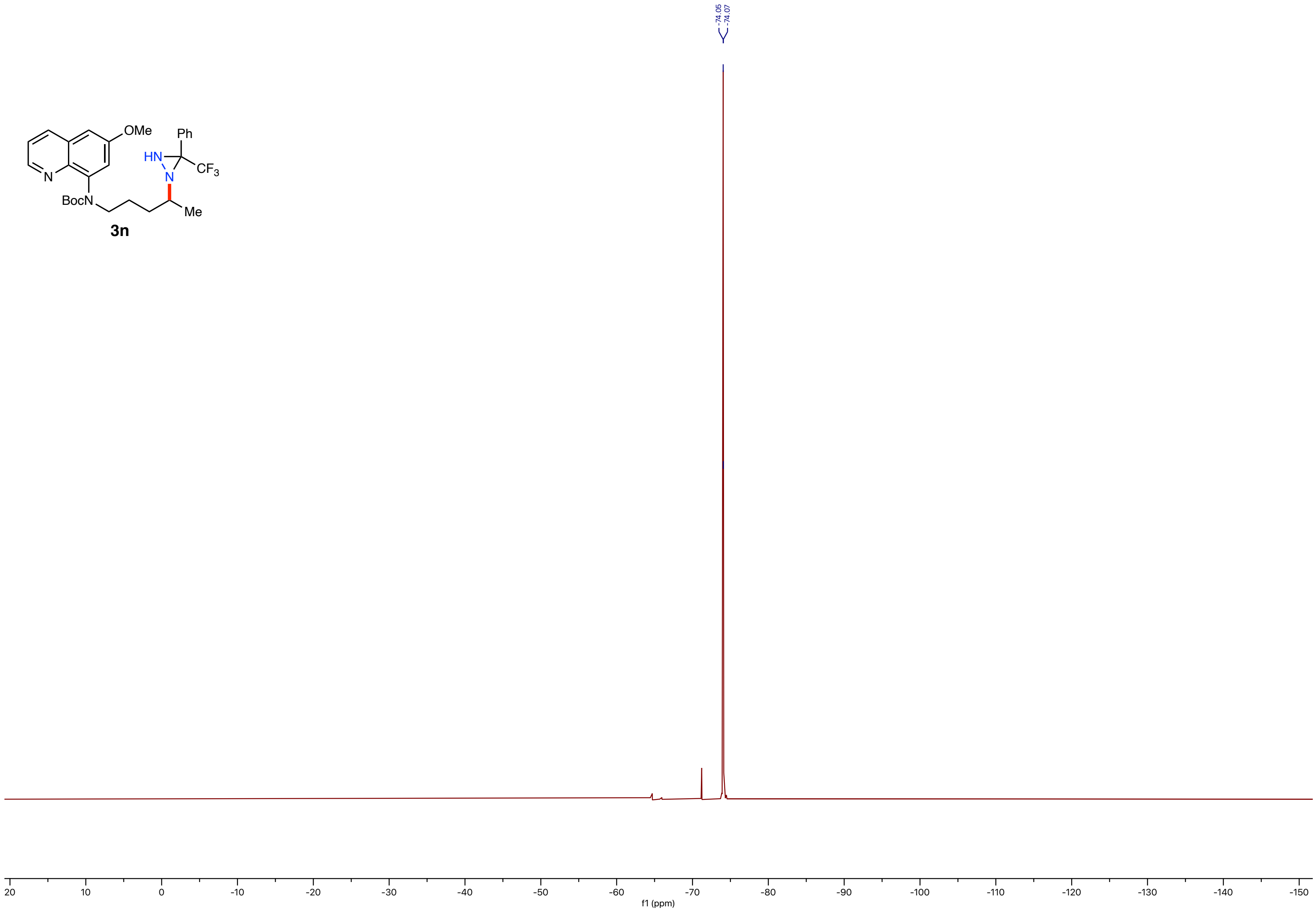

^{1}H NMR of 3o (Major, $CDCl_3$, 500 MHz)

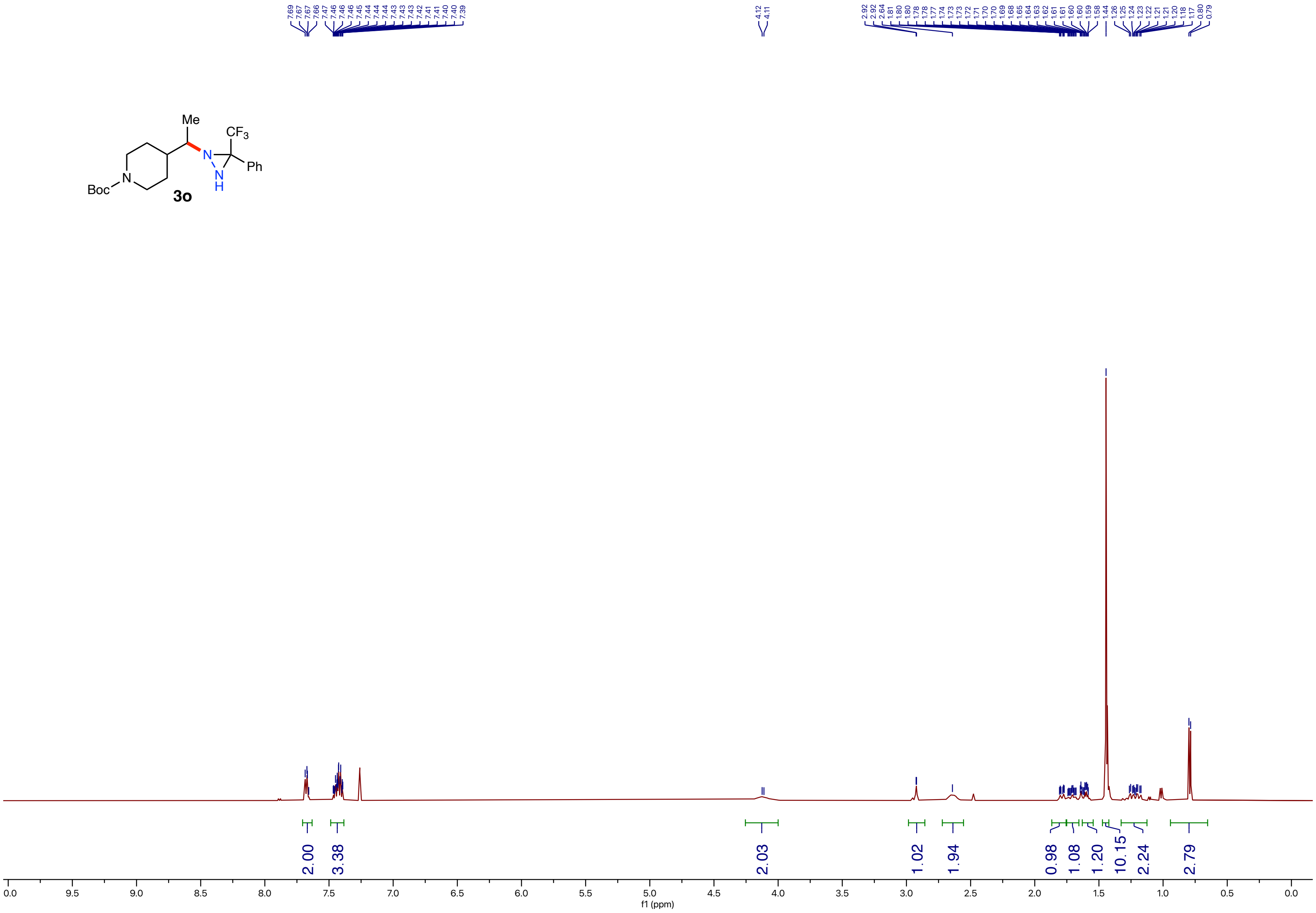

^{13}C NMR of 3o (Major, $CDCl_3$, 126 MHz)

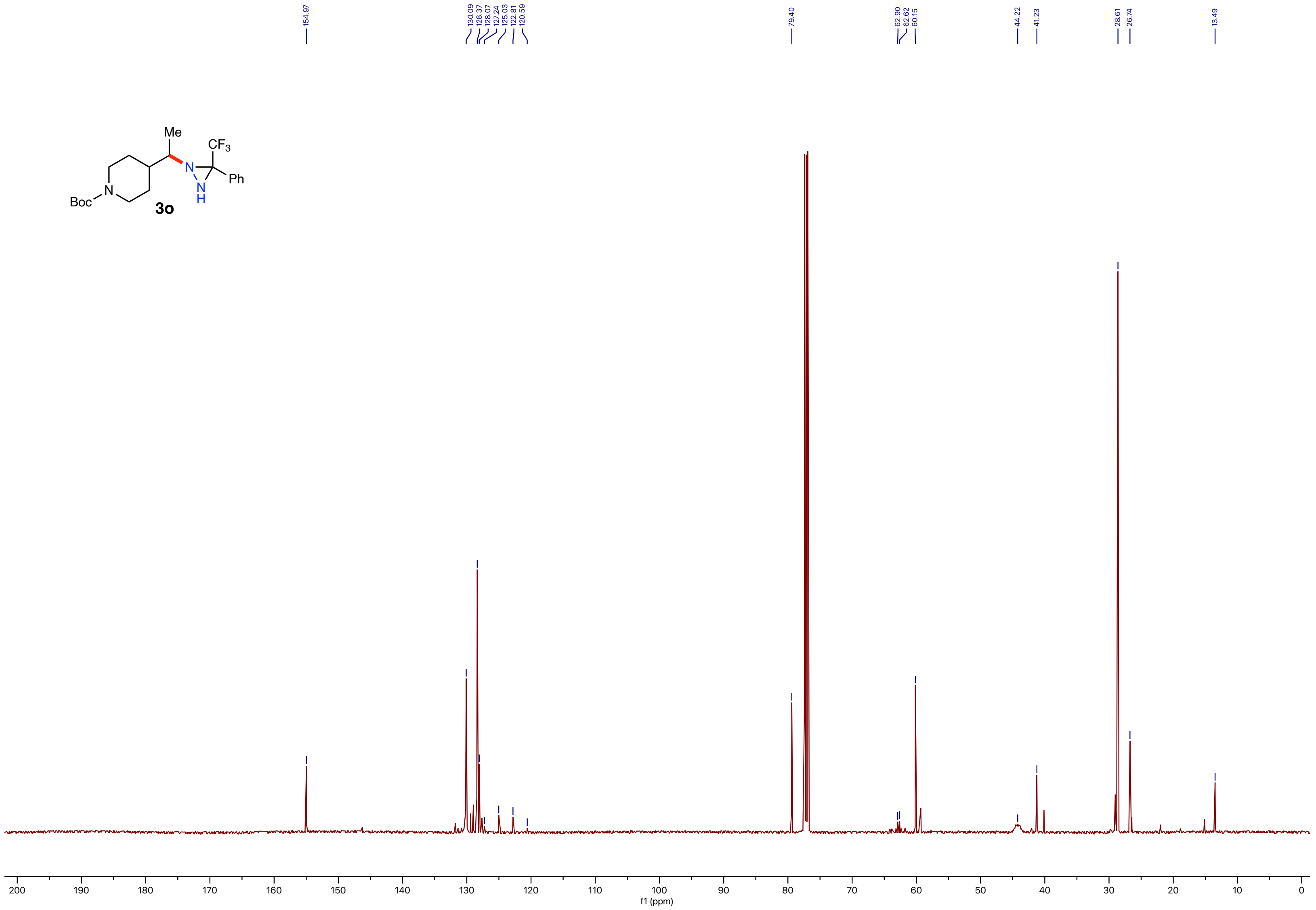

^{19}F NMR of 3o (Major, $CDCl_3$, 471 MHz)

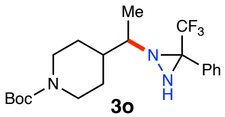

3o

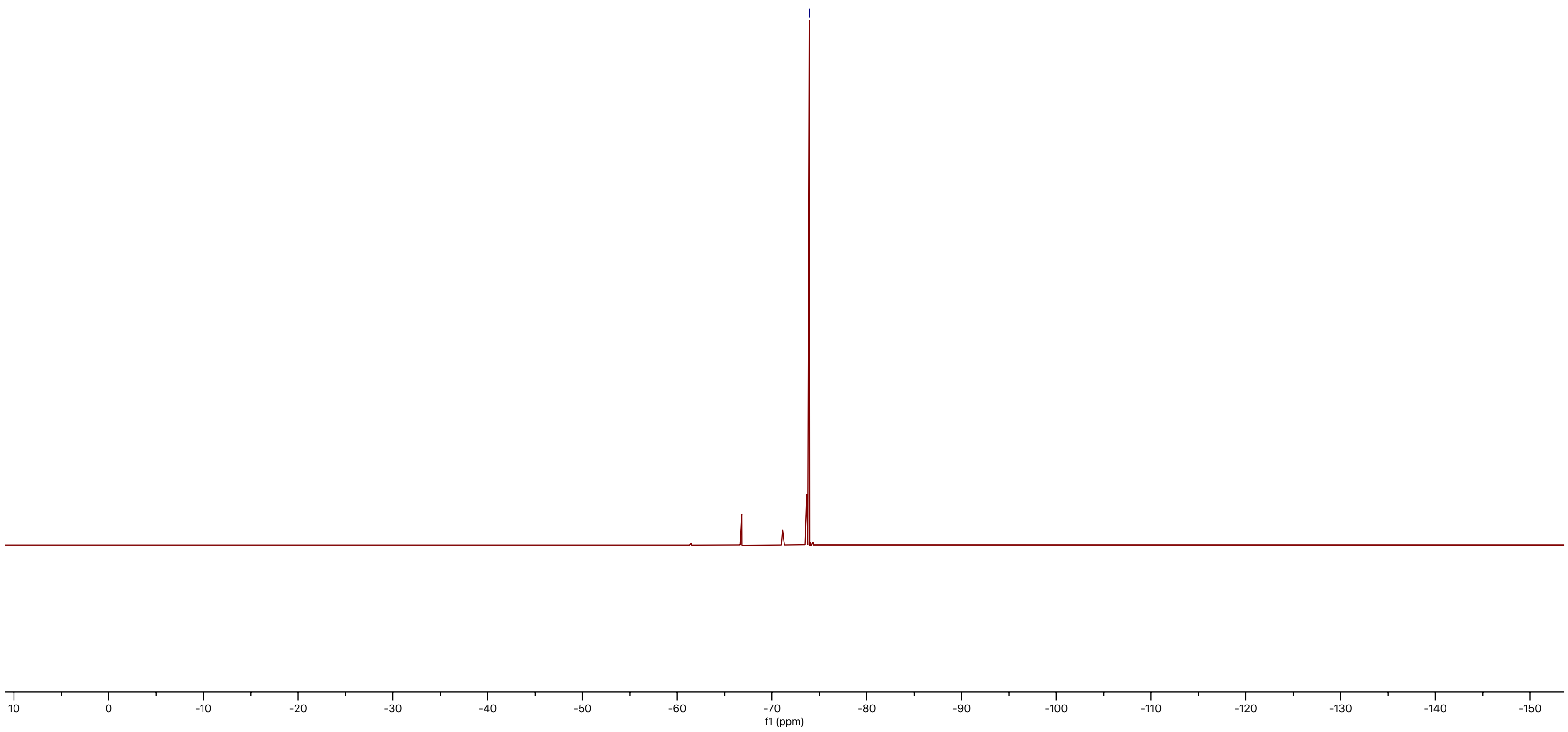

^{1}H NMR of 3o (Minor, $CDCl_3$, 500 MHz)

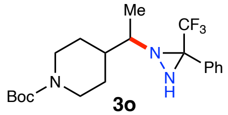

3o

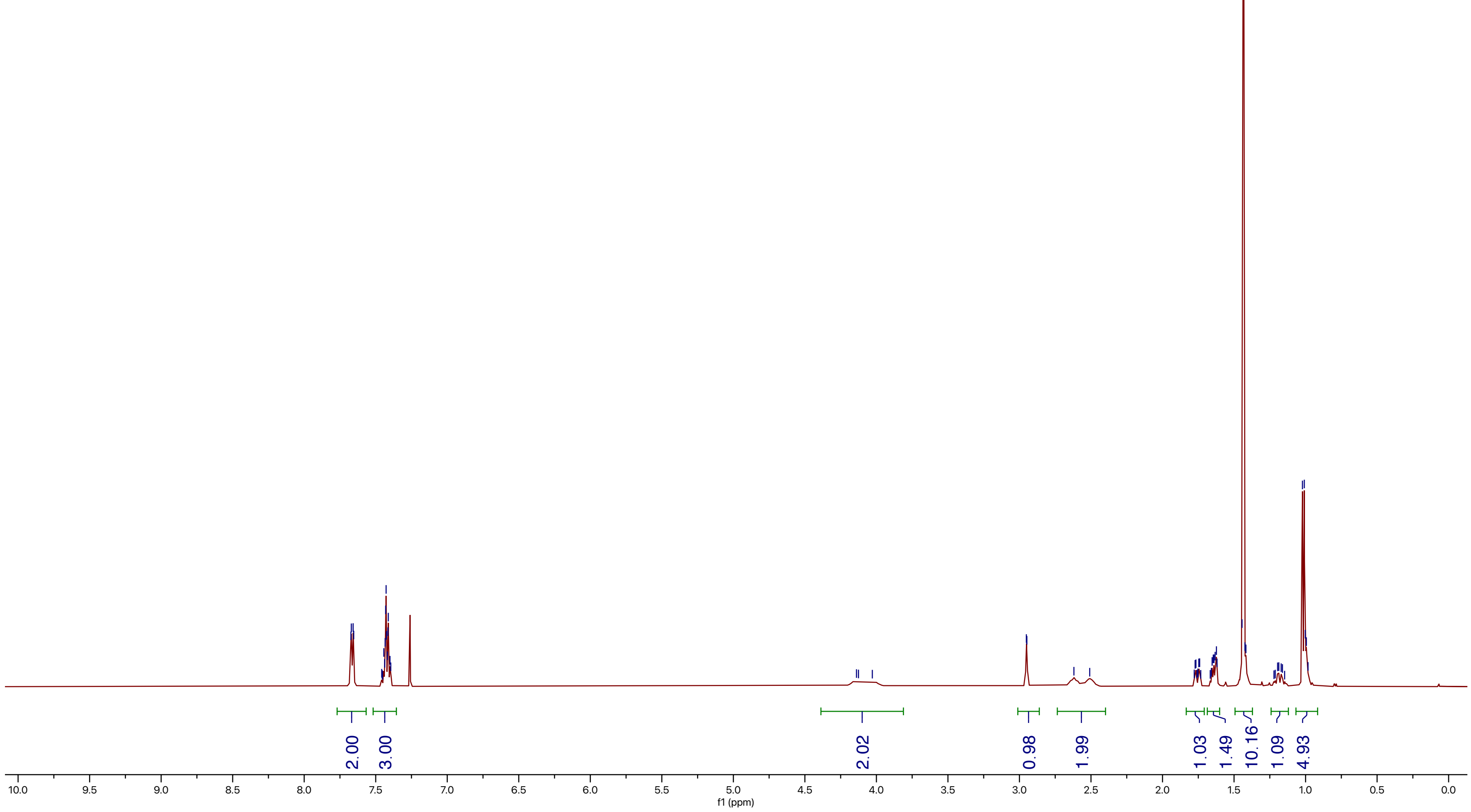

^{13}C NMR of 3o (Minor, $CDCl_3$, 126 MHz)

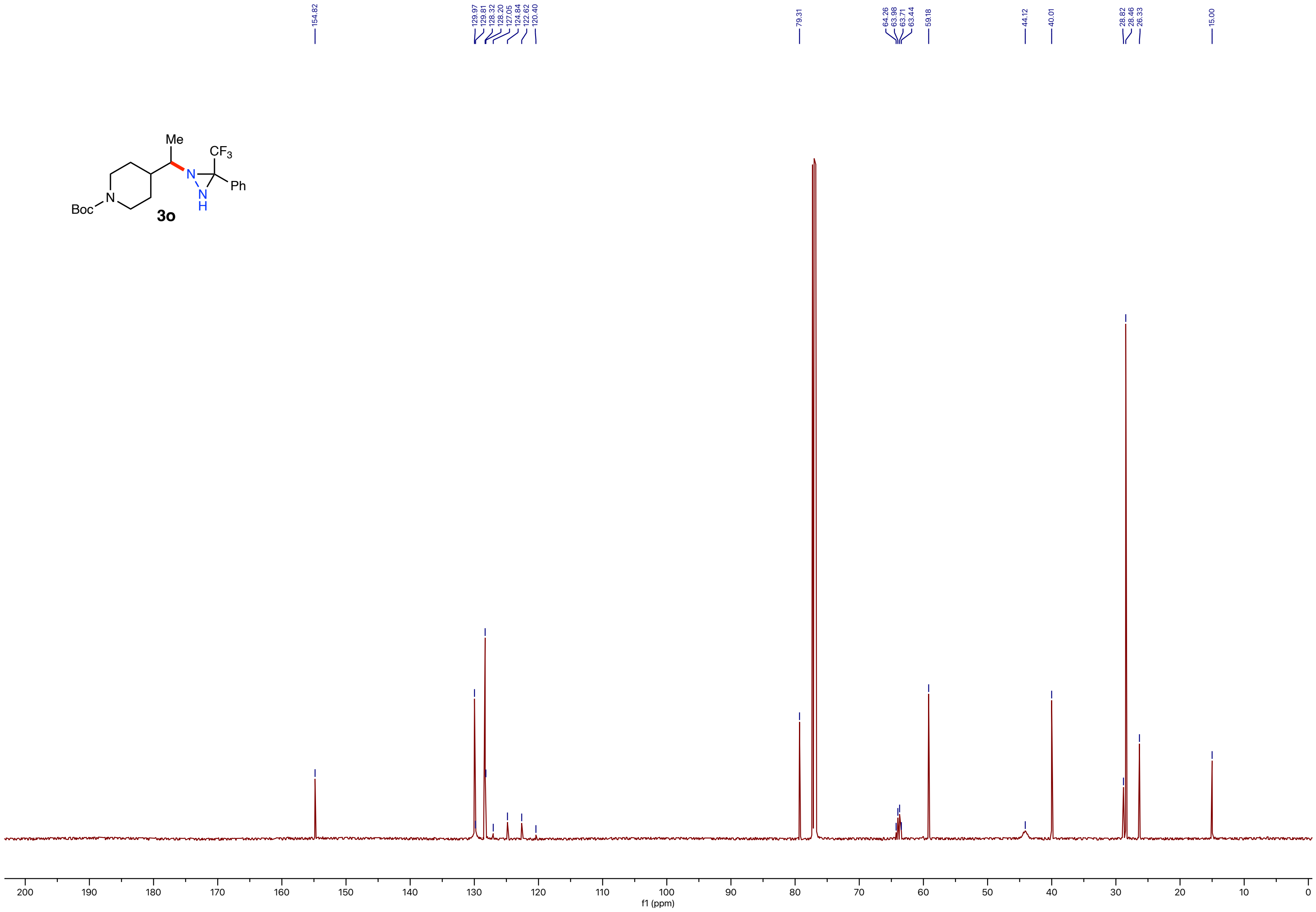

^{19}F NMR of 3o (Minor, $CDCl_3$, 471 MHz)

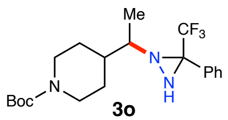

3o

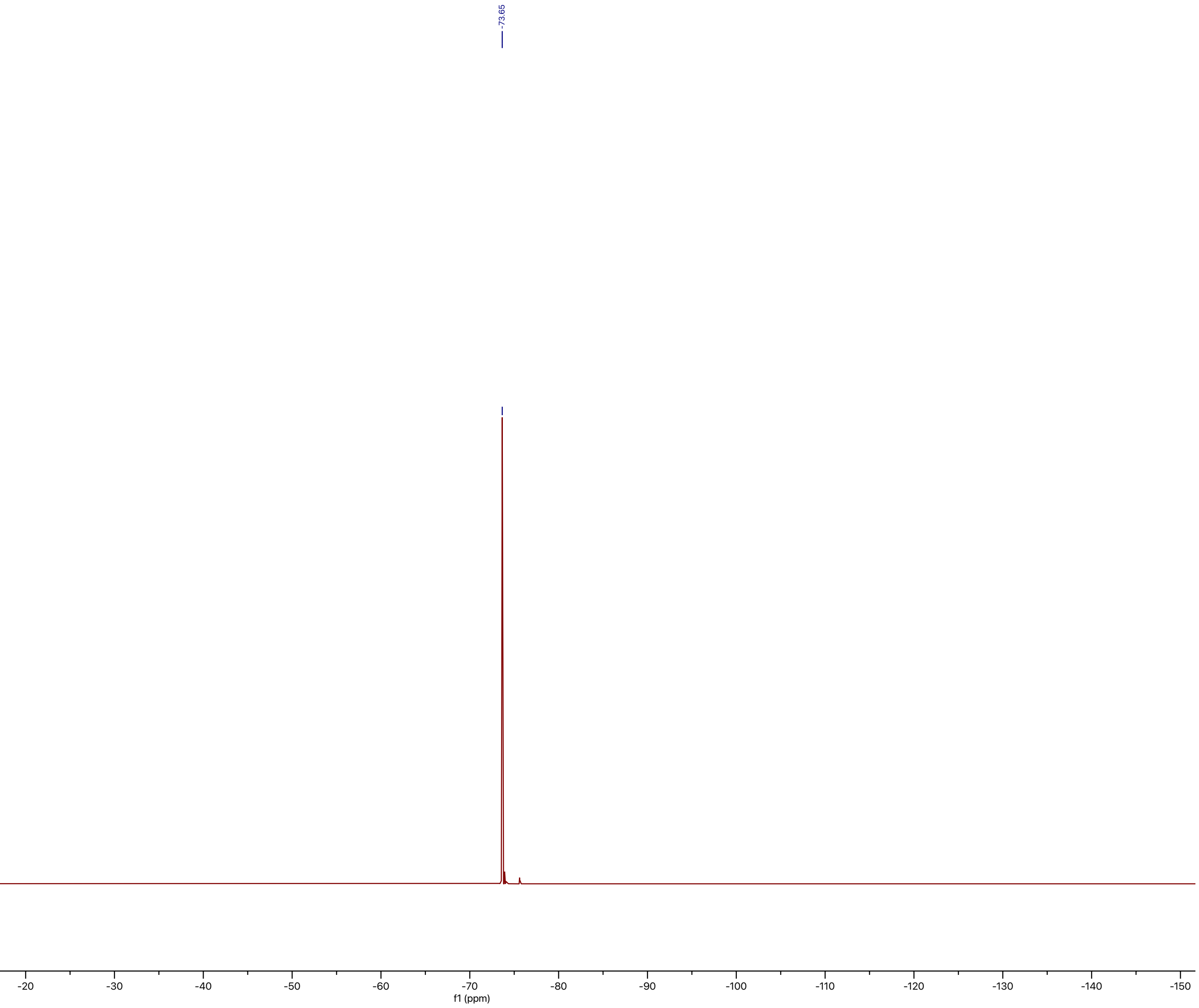

^{1}H NMR of 3p ($CDCl_3$, 500 MHz)

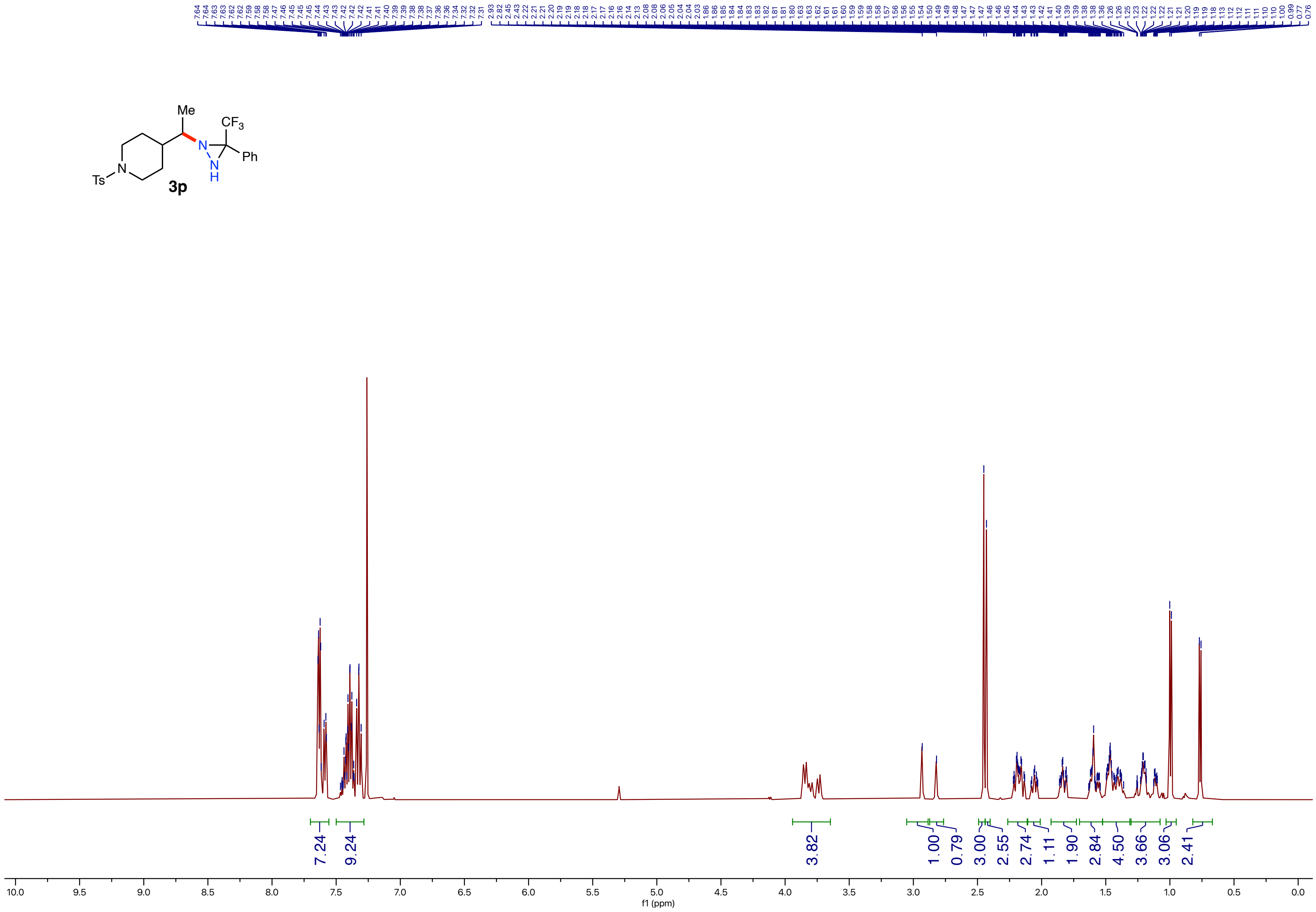

^{13}C NMR of 3p (CDCl$_3$, 126 MHz)

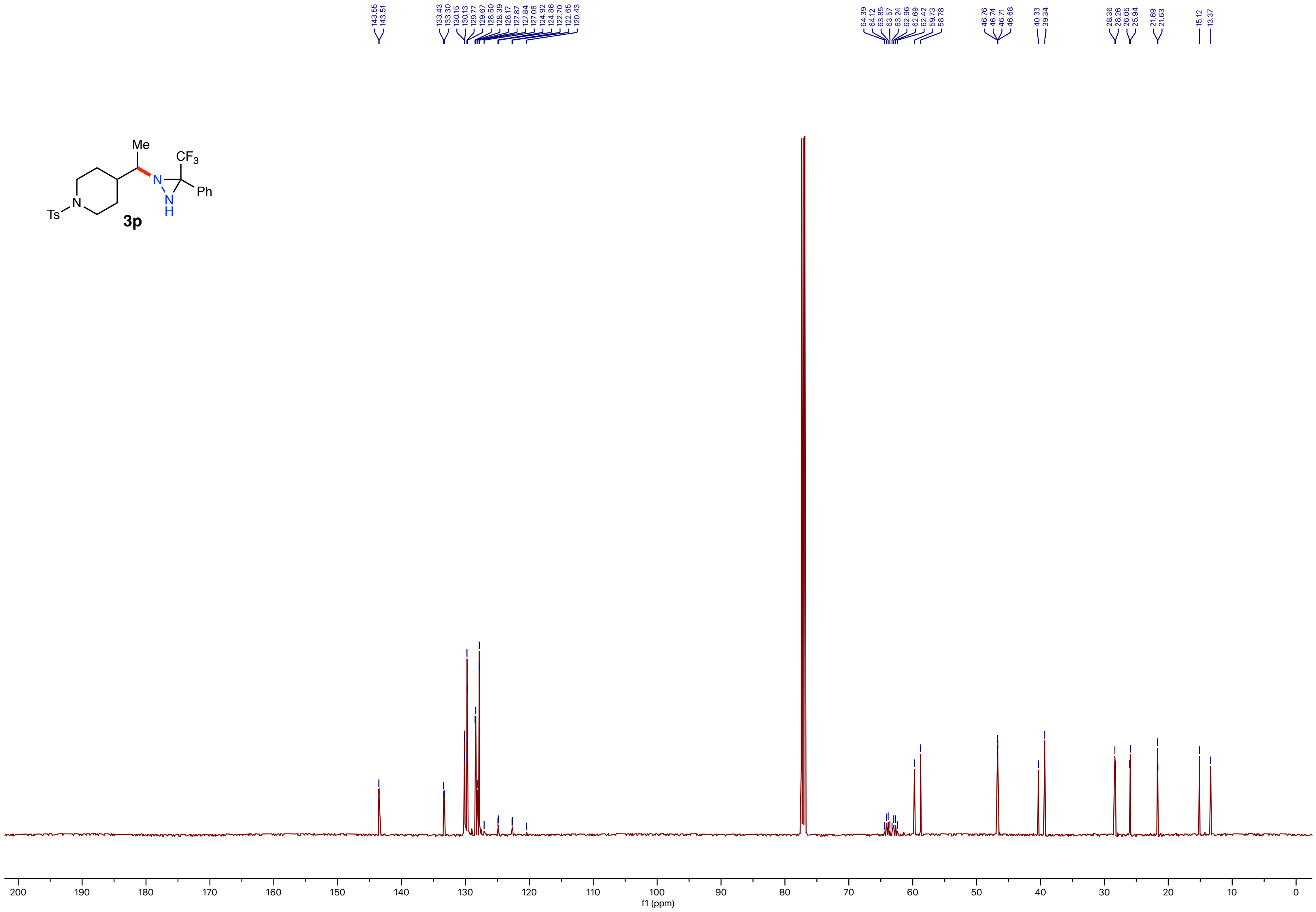

^{19}F NMR of 3p ($CDCl_3$, 471 MHz)

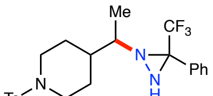

3p

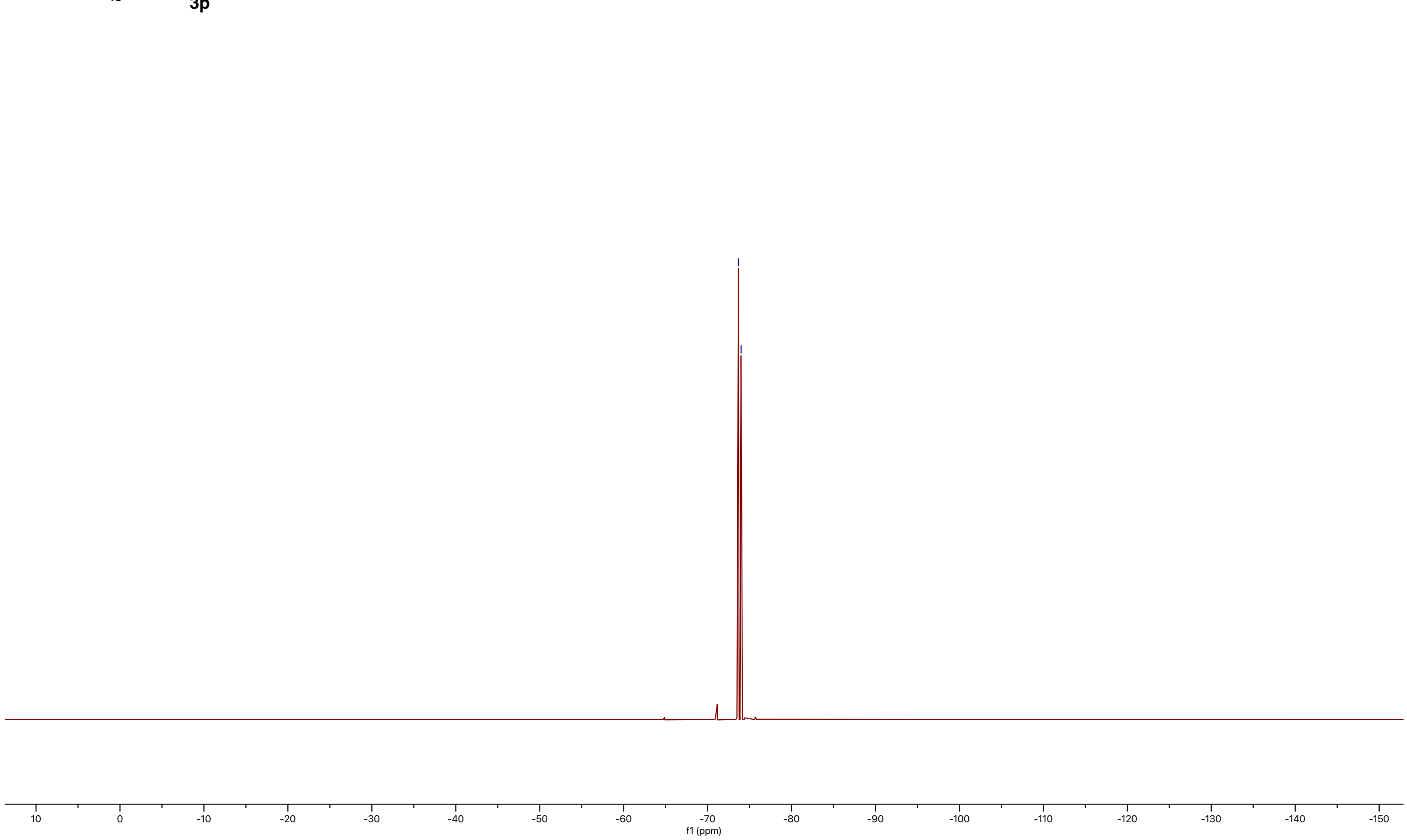

^{1}H NMR of 3q ($CDCl_3$, 500 MHz)

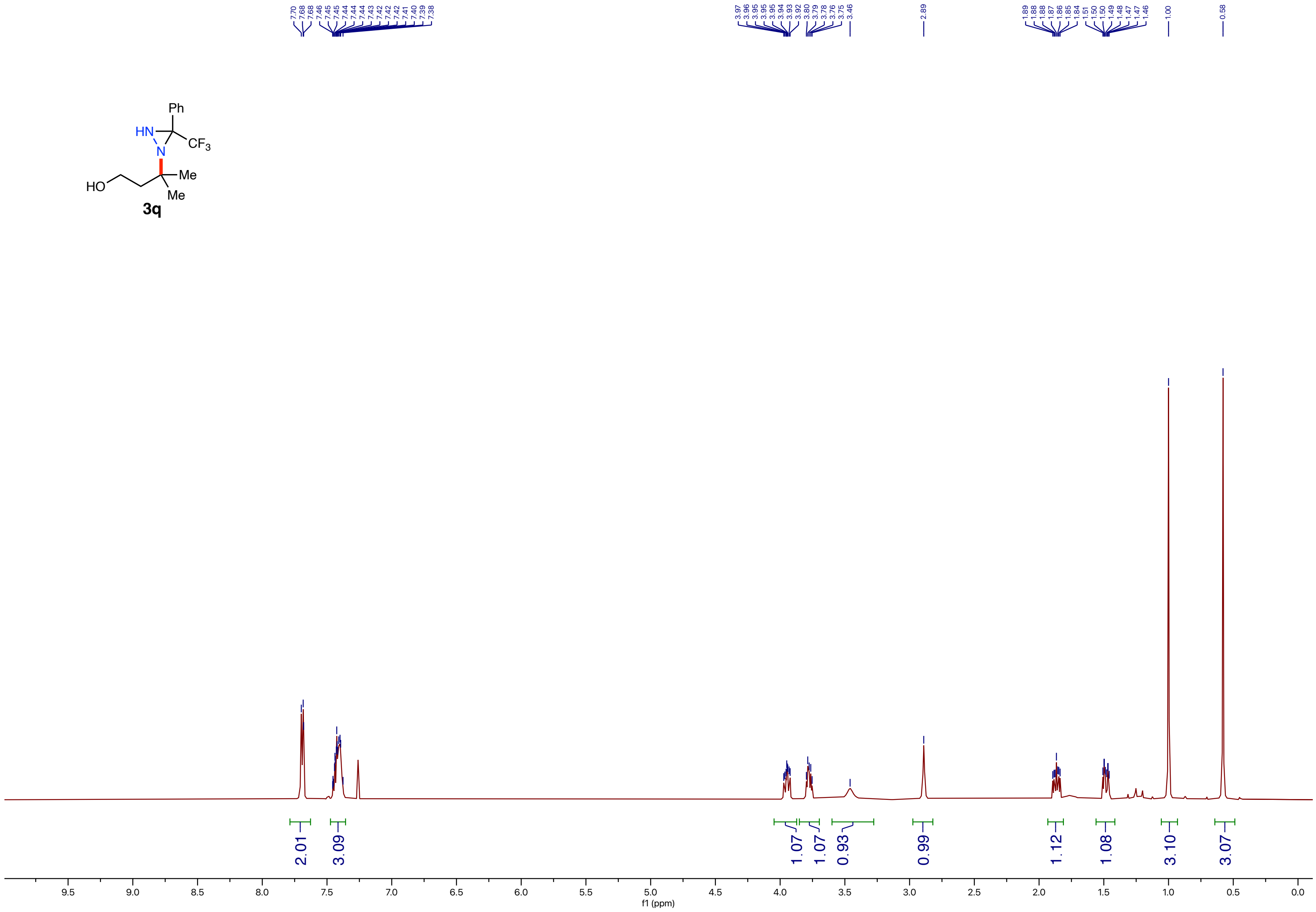

^{13}C NMR of 3q ($CDCl_3$, 126 MHz)

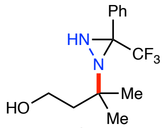

3q

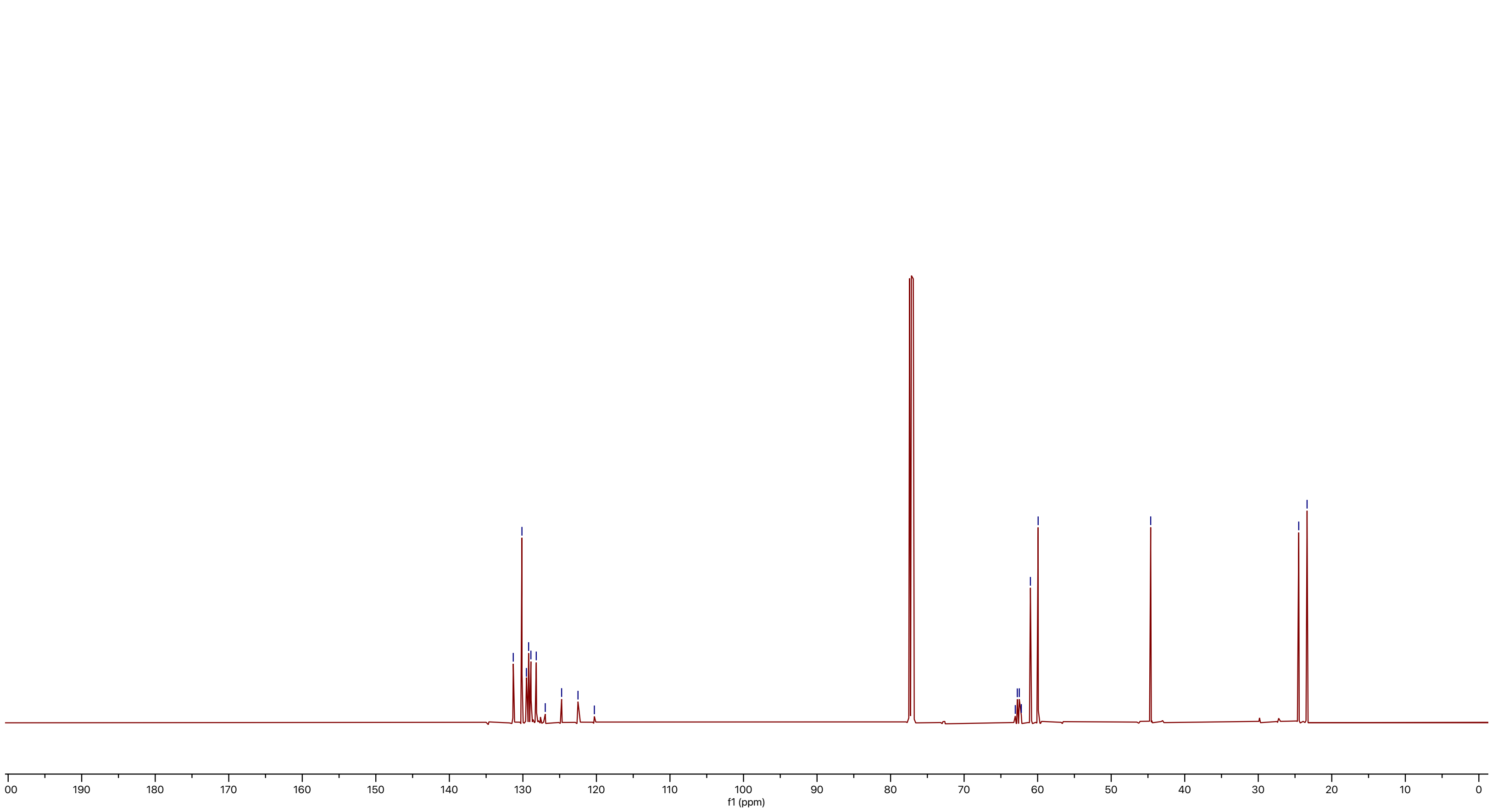

^{19}F NMR of 3q ($CDCl_3$, 471 MHz)

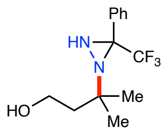

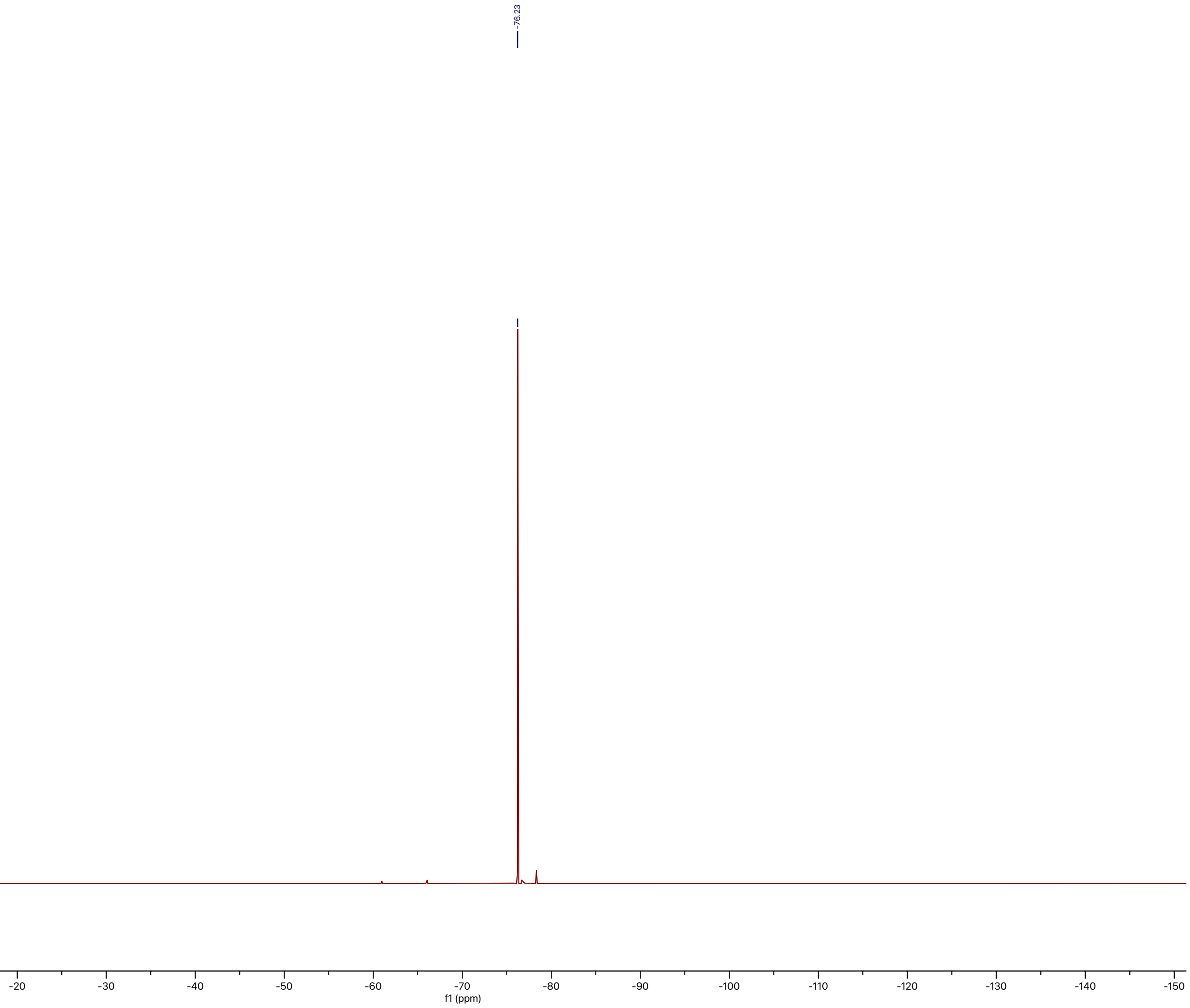

^{1}H NMR of 3r (CDCl$_3$, 500 MHz)

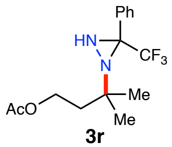

3r

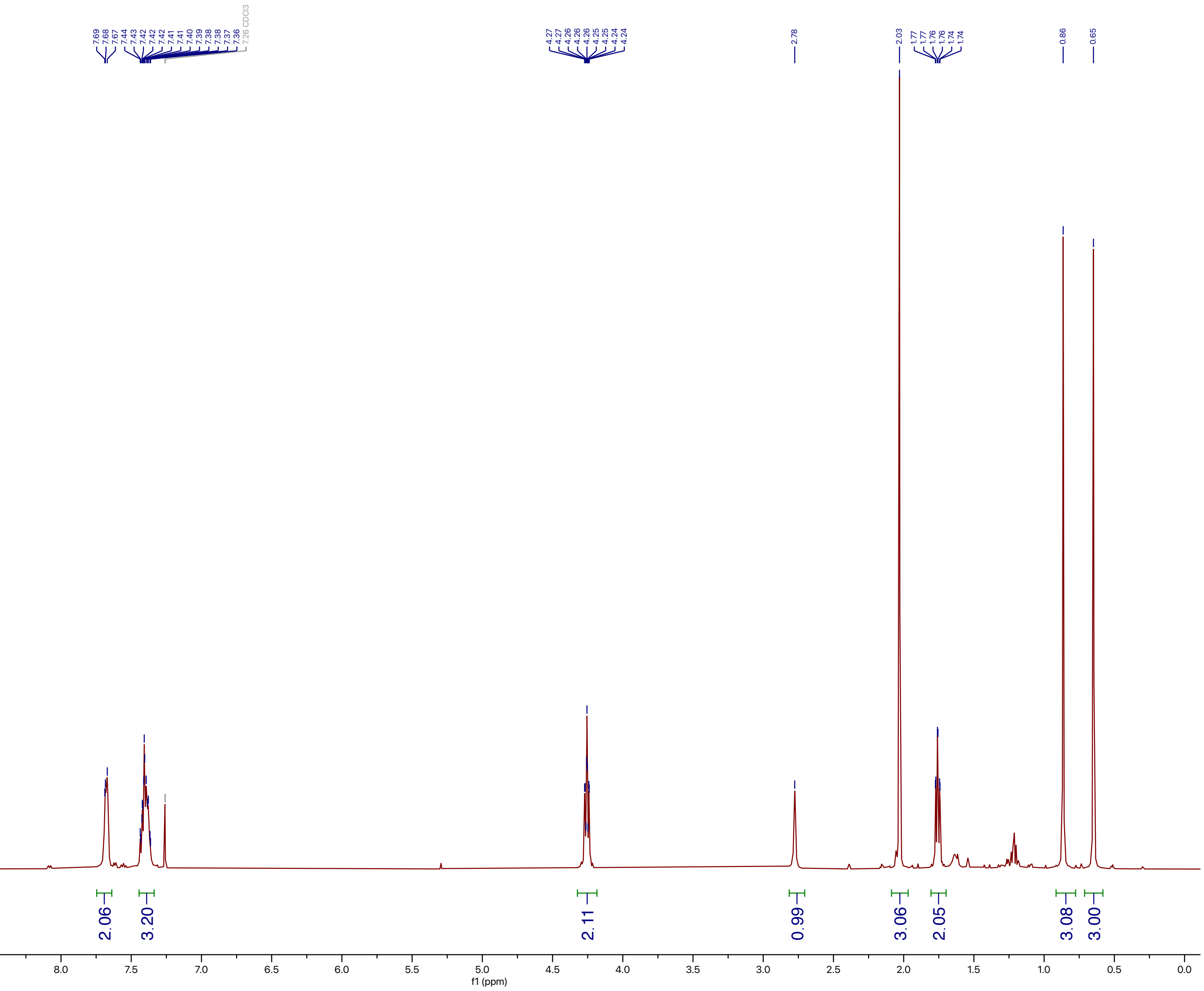

^{13}C NMR of 3r ($CDCl_3$, 126 MHz)

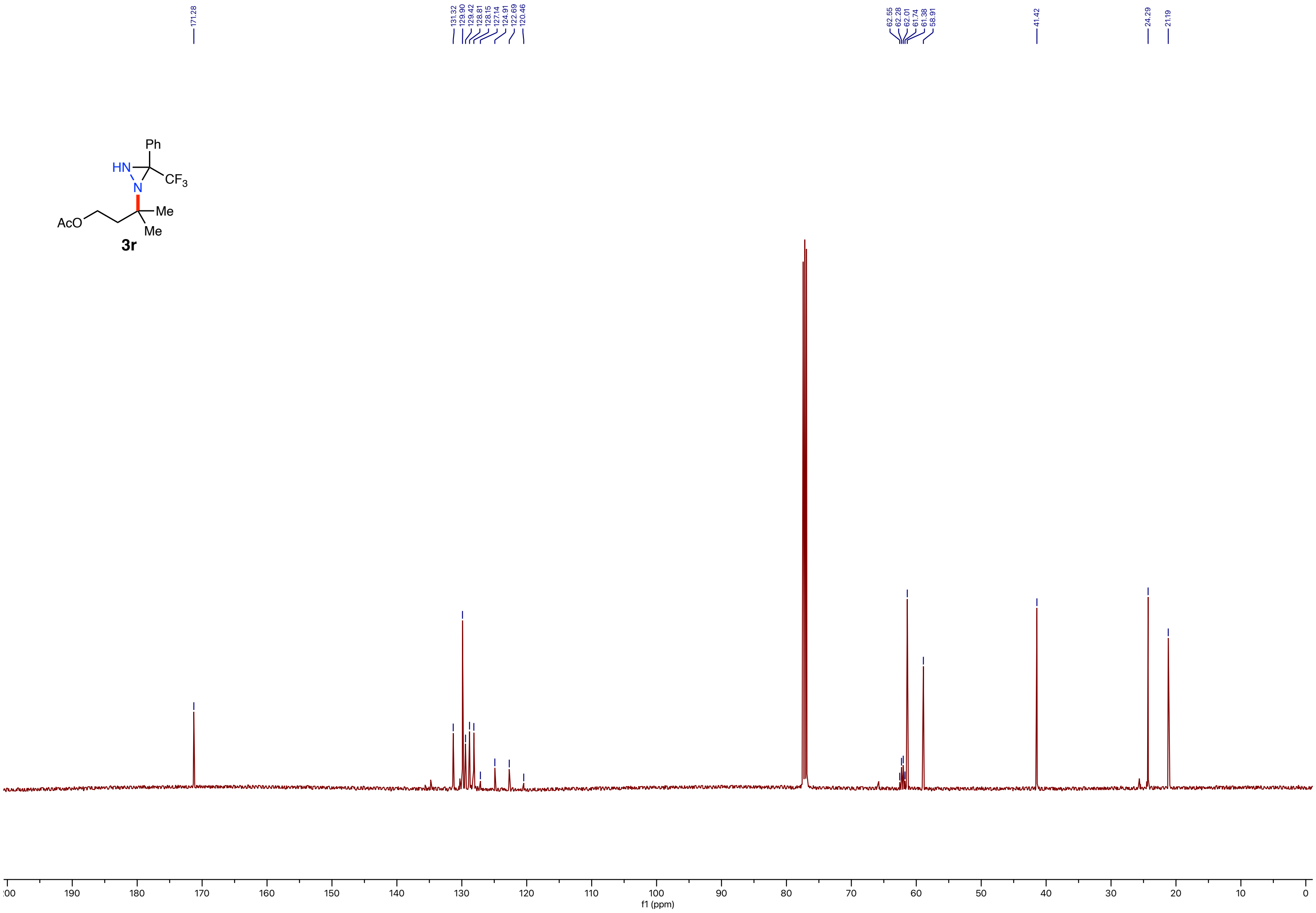

^{19}F NMR of 3r ($CDCl_3$, 471 MHz)

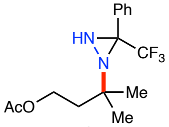

3r

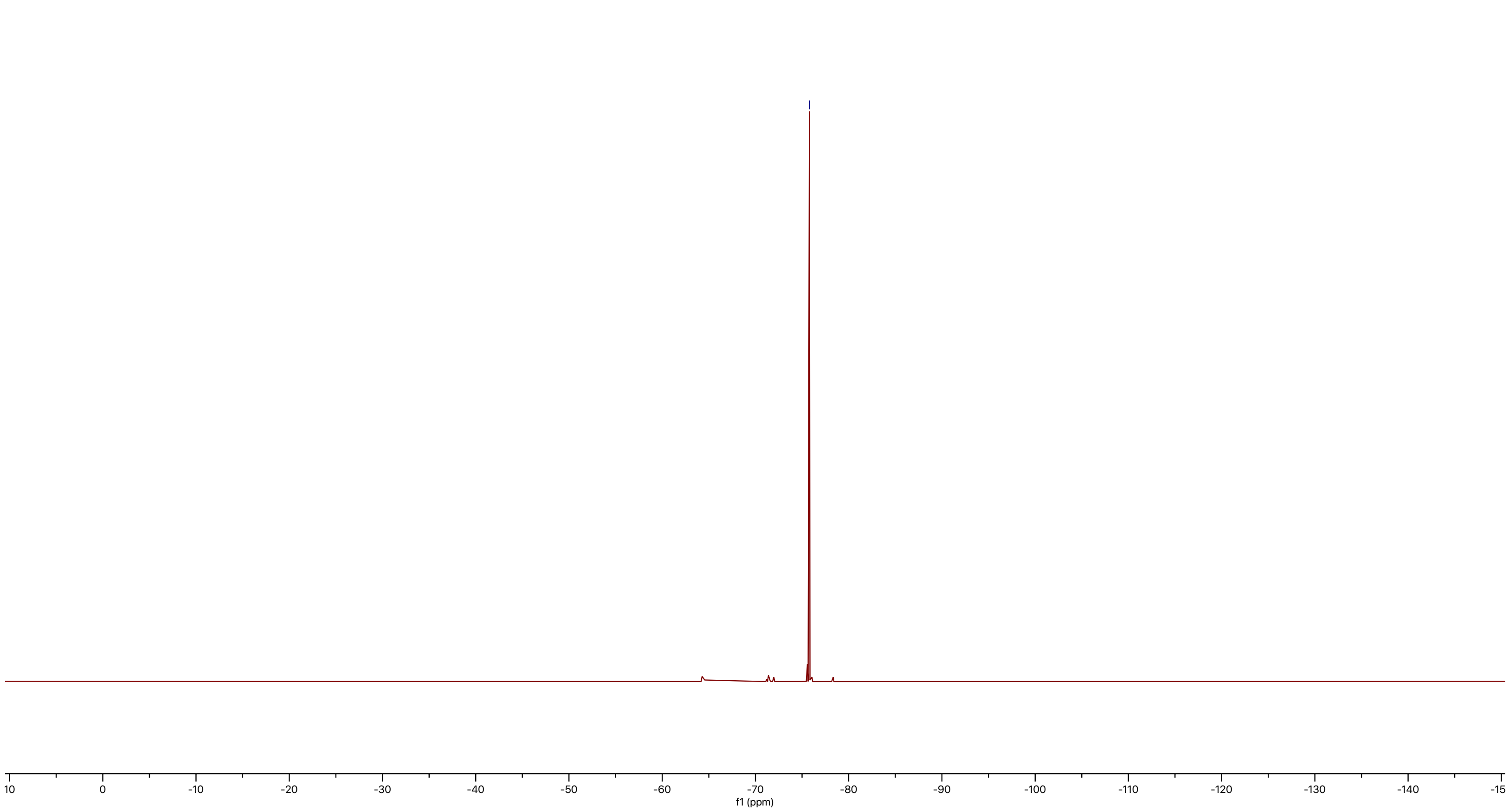

^{1}H NMR of 3s ($CDCl_3$, 500 MHz)

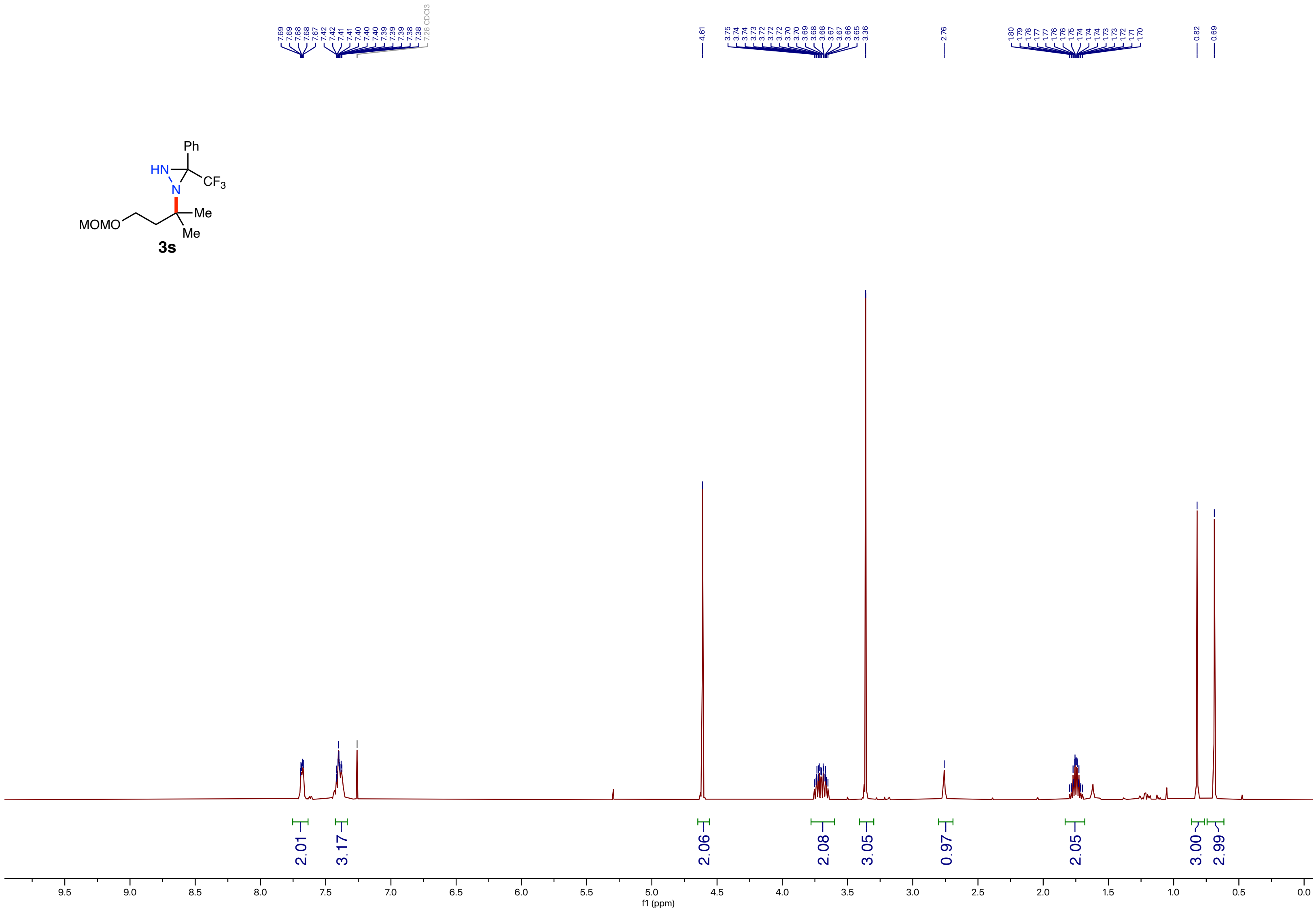

^{13}C NMR of 3s ($CDCl_3$, 126 MHz)

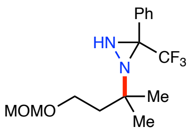

3s

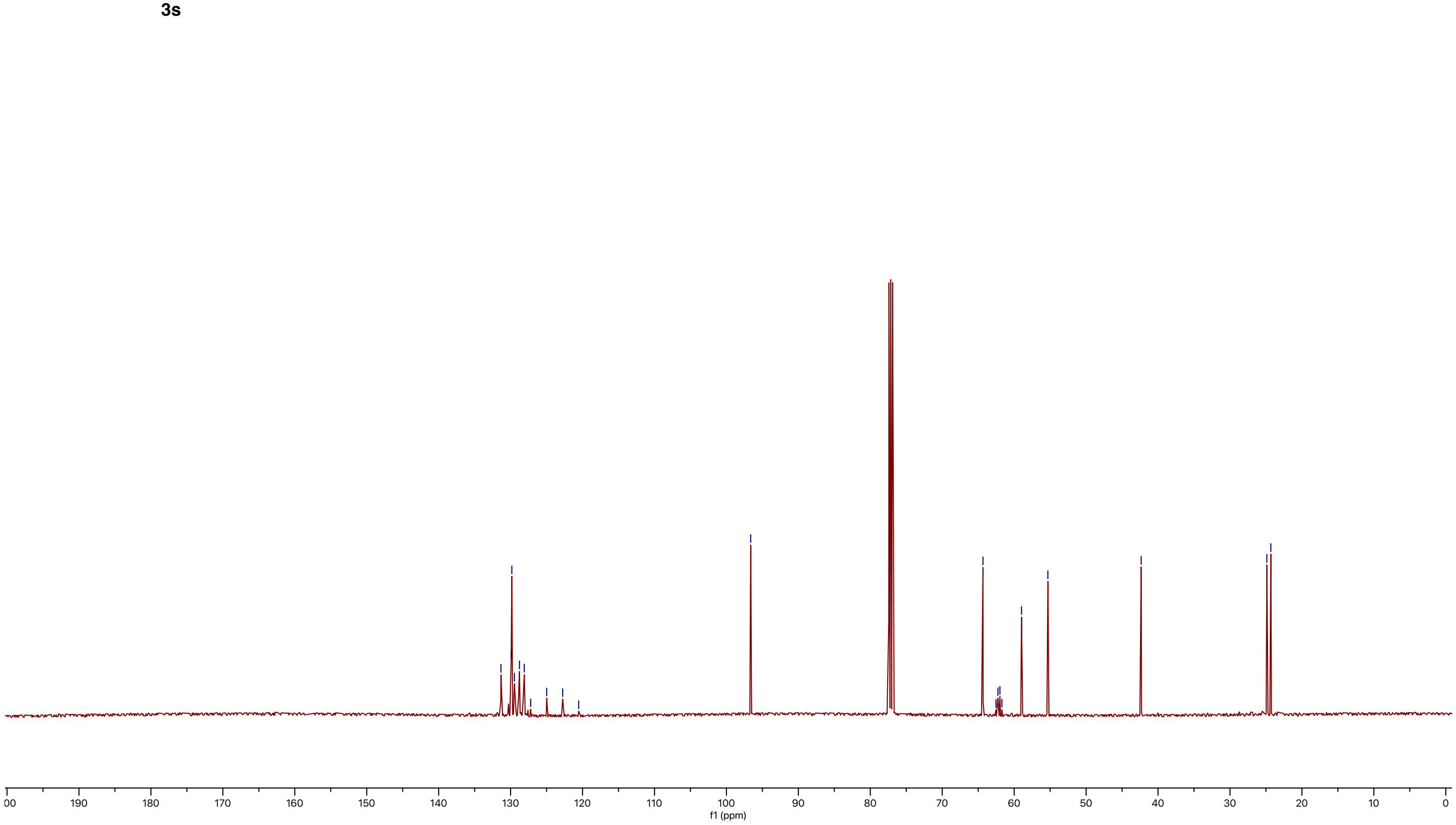

^{19}F NMR of 3s ($CDCl_3$, 471 MHz)

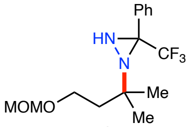

3s

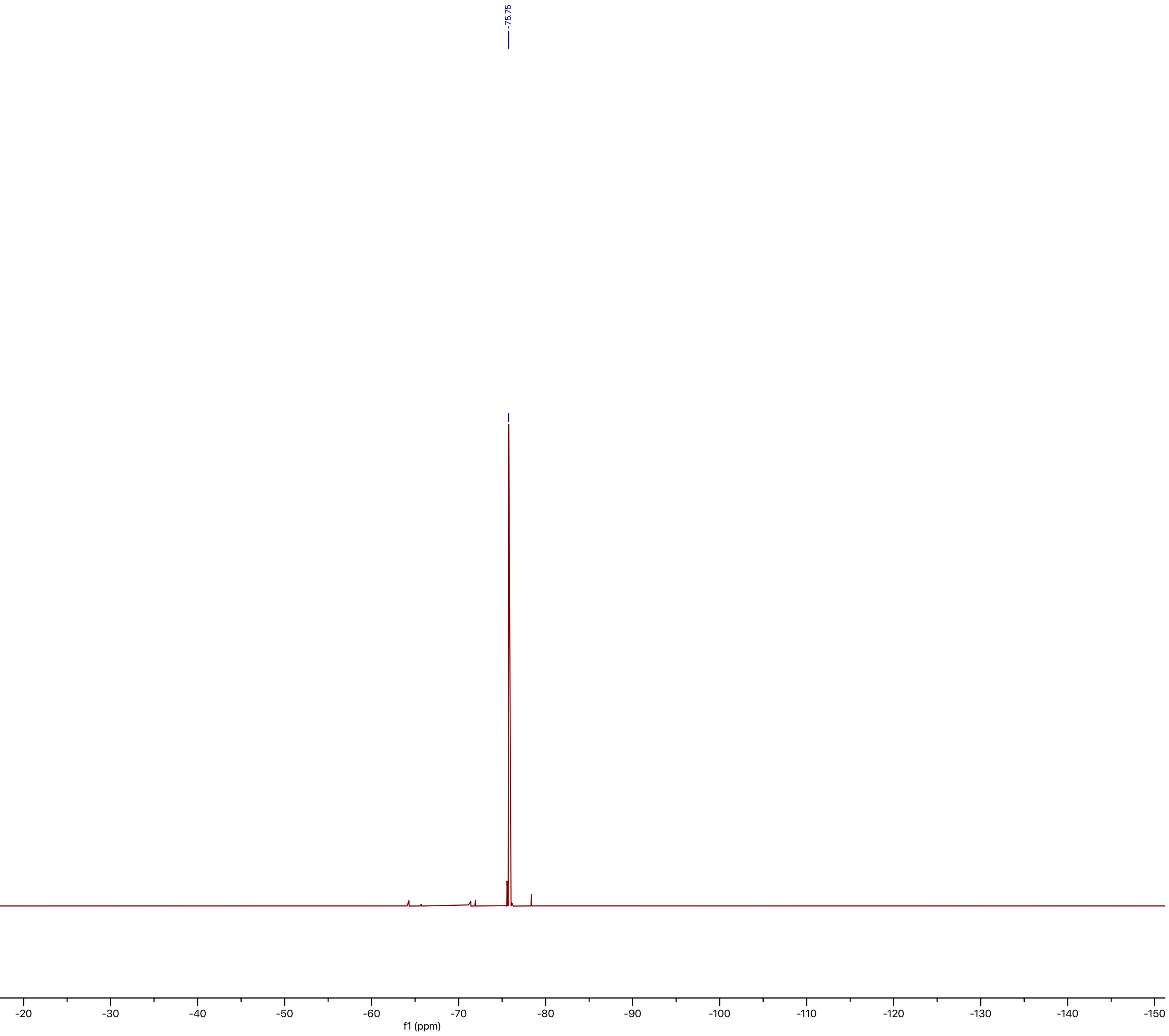

^{1}H NMR of 3t ($CDCl_3$, 500 MHz)

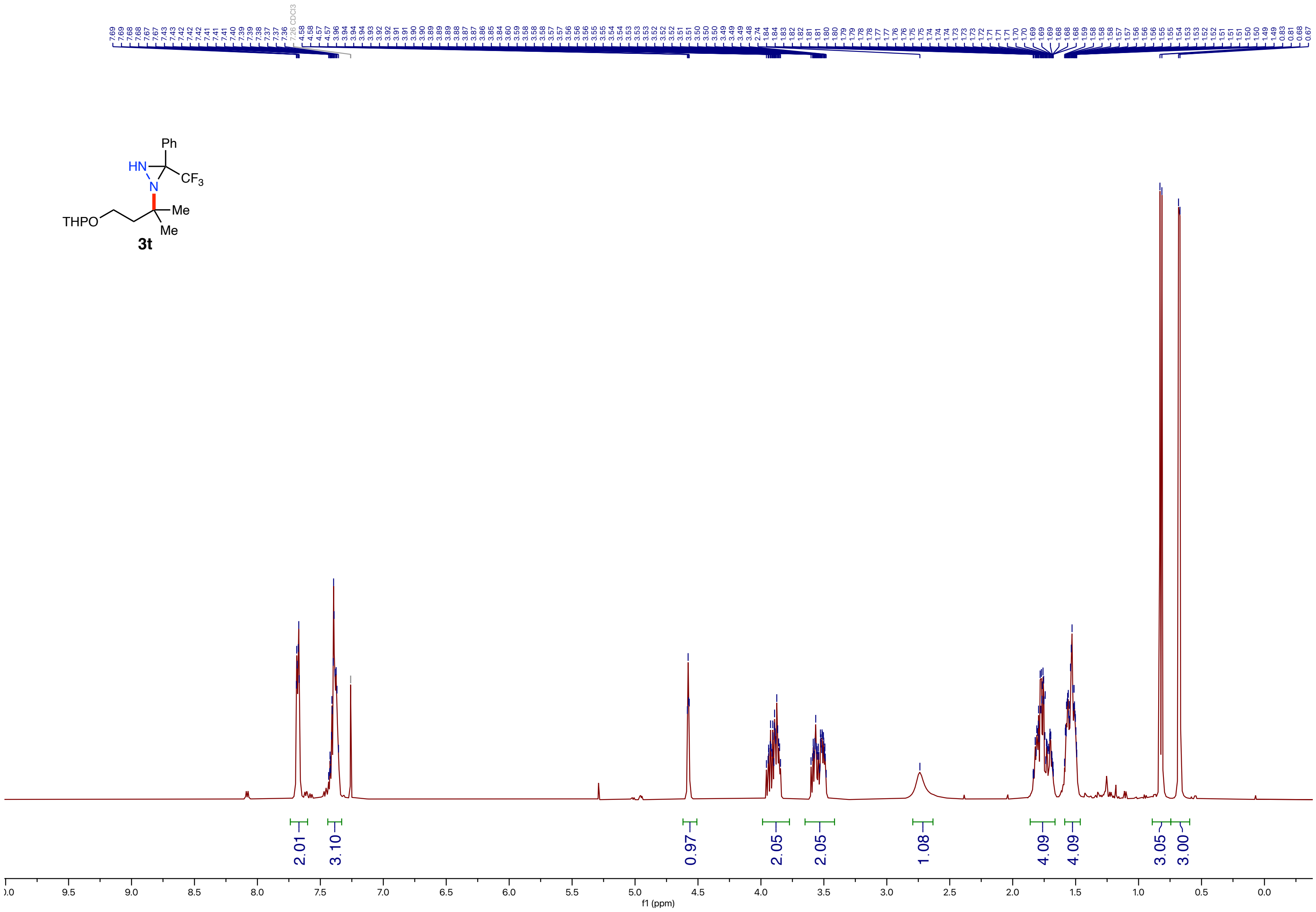

^{13}C NMR of 3t ($CDCl_3$, 126 MHz)

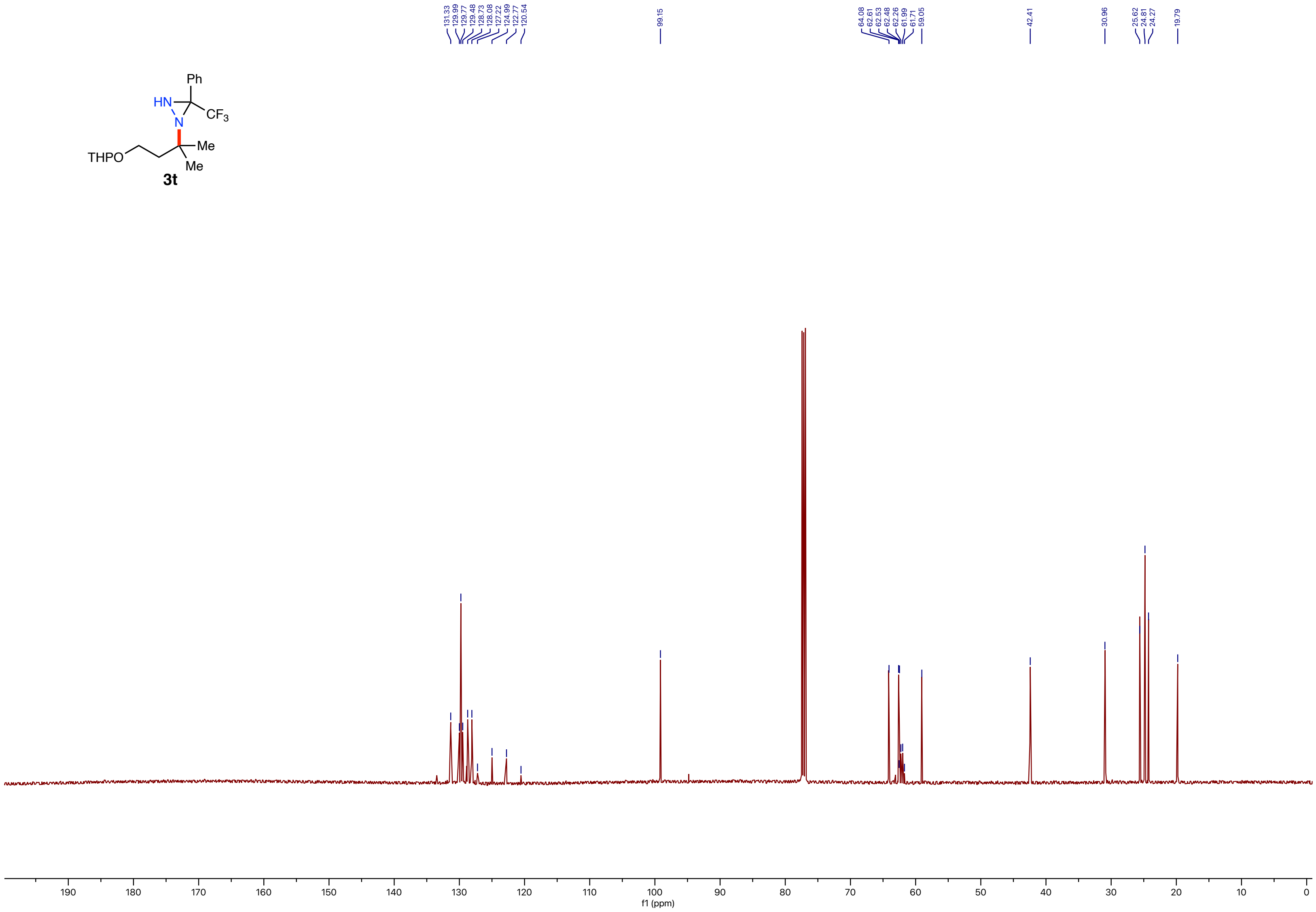

^{19}F NMR of 3t ($CDCl_3$, 471 MHz)

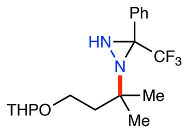

3t

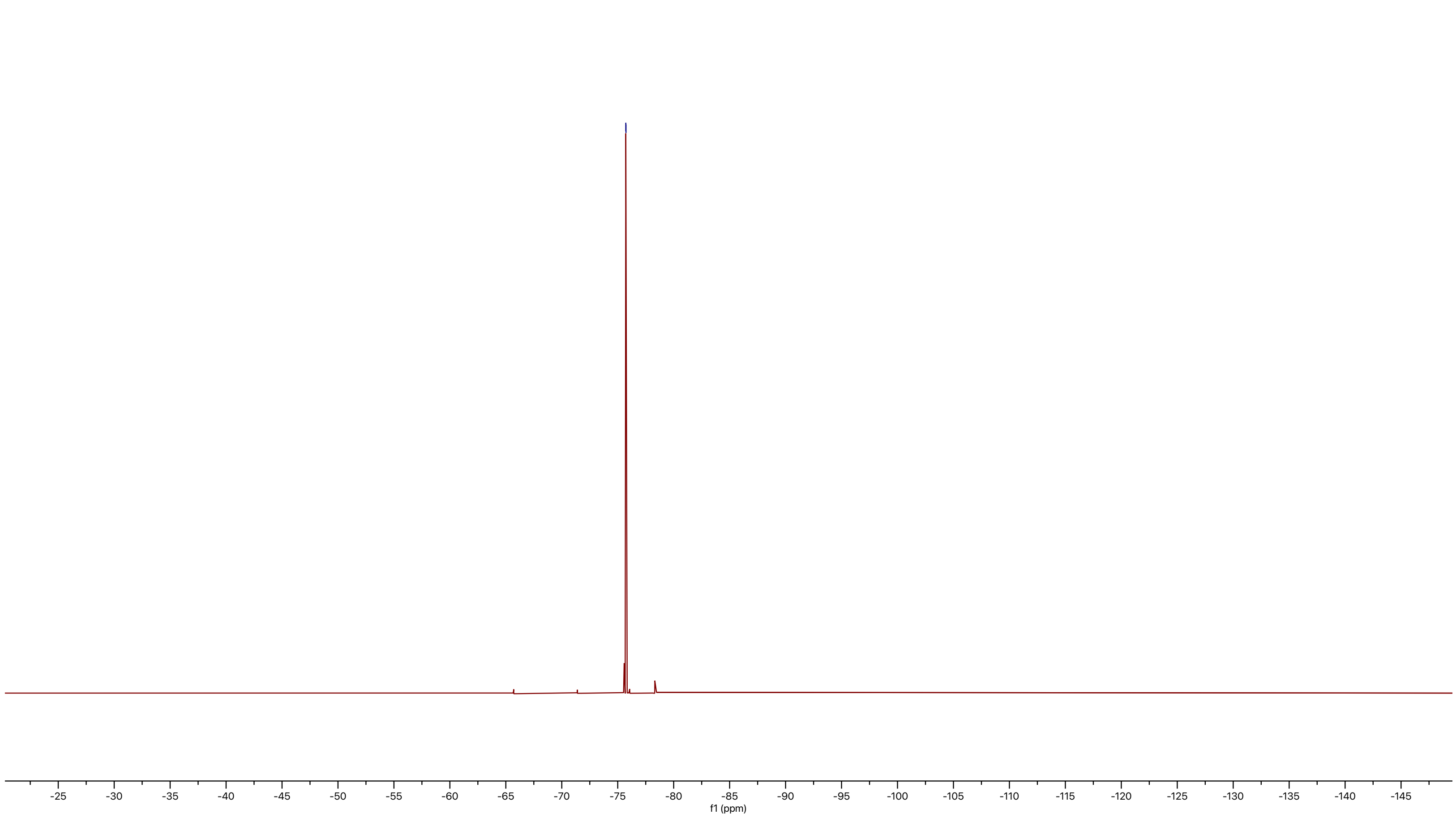

^{1}H NMR of 3u ($CDCl_3$, 500 MHz)

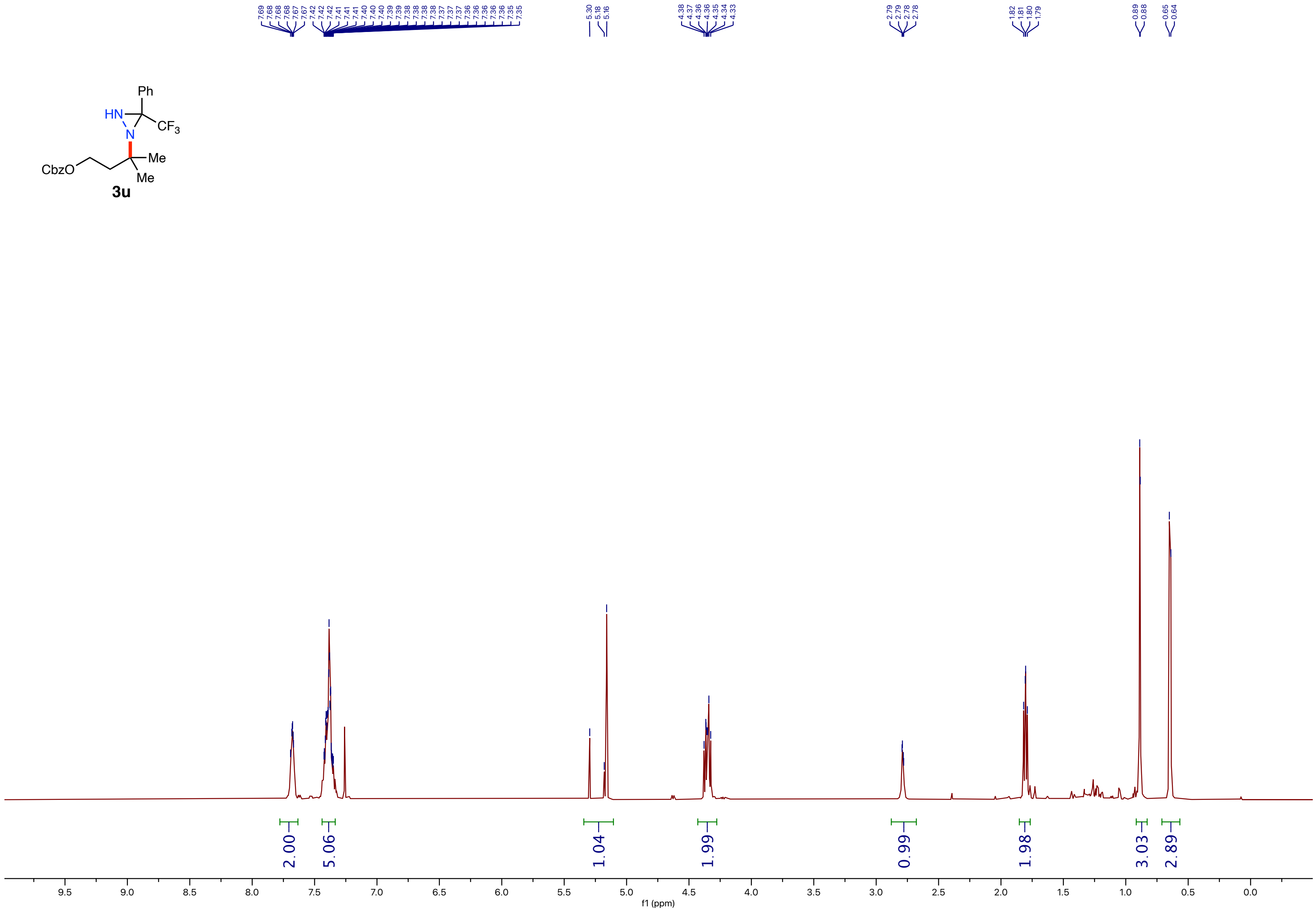

^{13}C NMR of 3u ($CDCl_3$, 126 MHz)

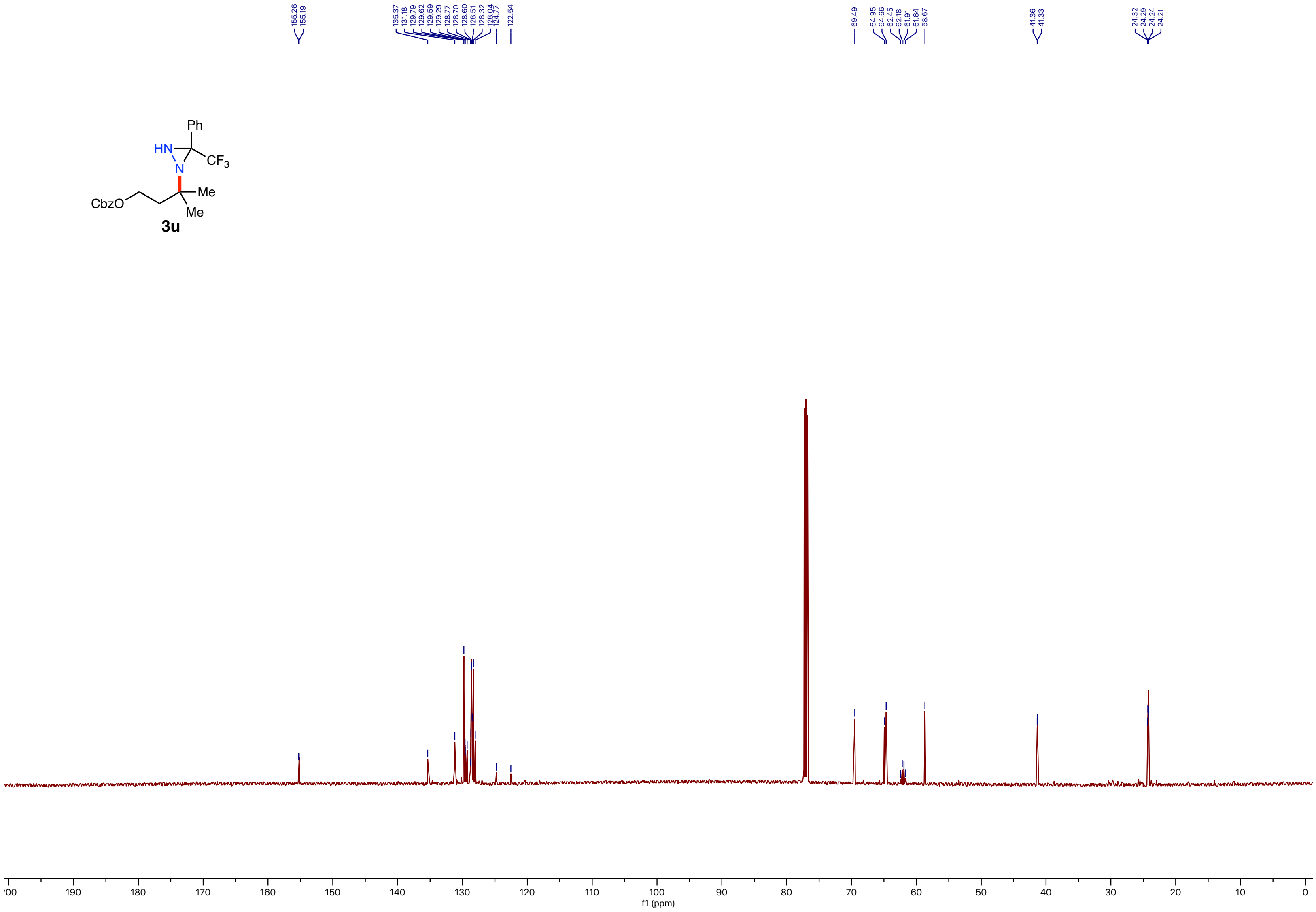

^{19}F NMR of 3u ($CDCl_3$, 471 MHz)

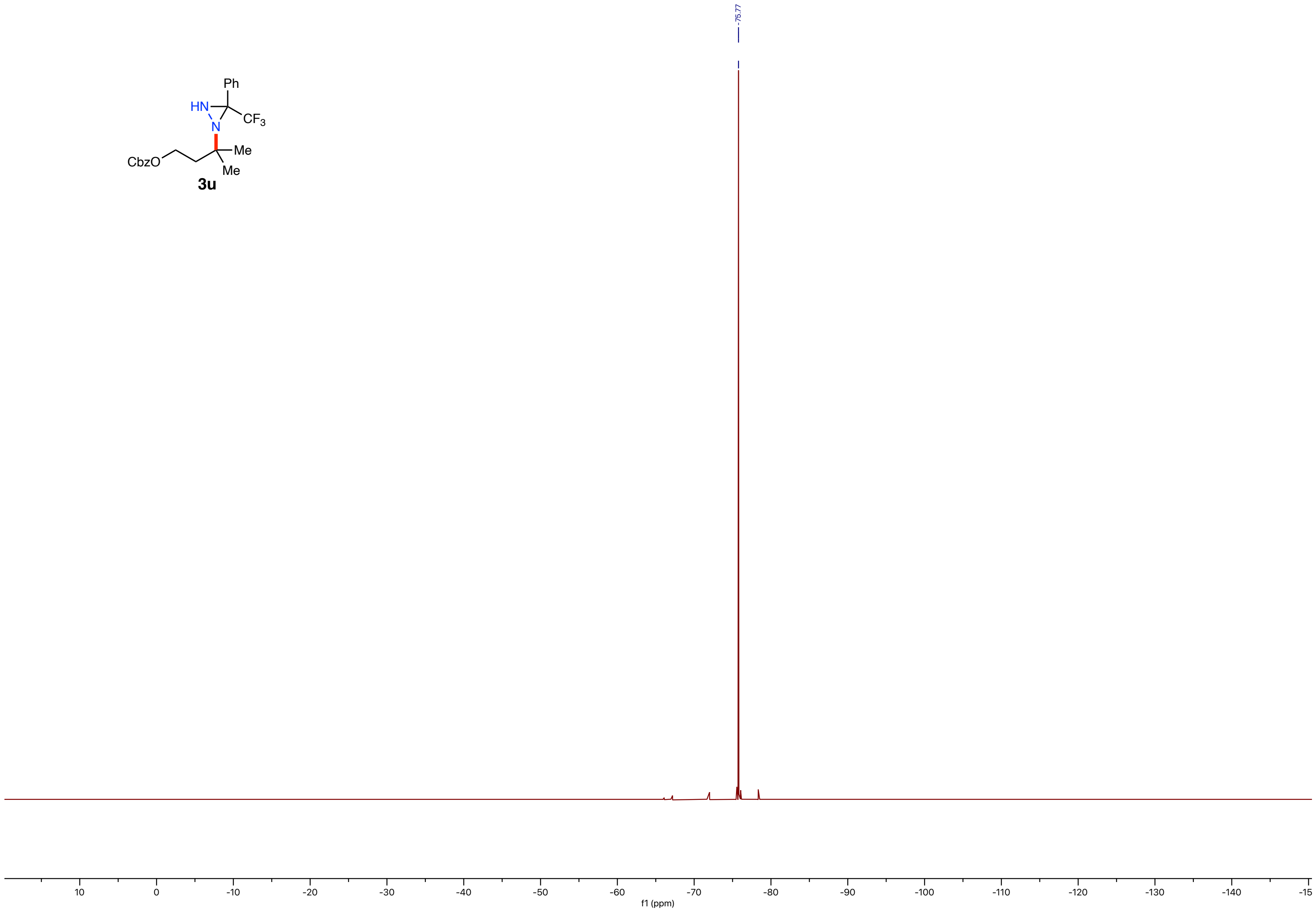

^{1}H NMR of 3v ($CDCl_3$, 500 MHz)

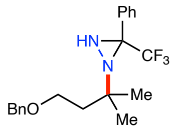

3v

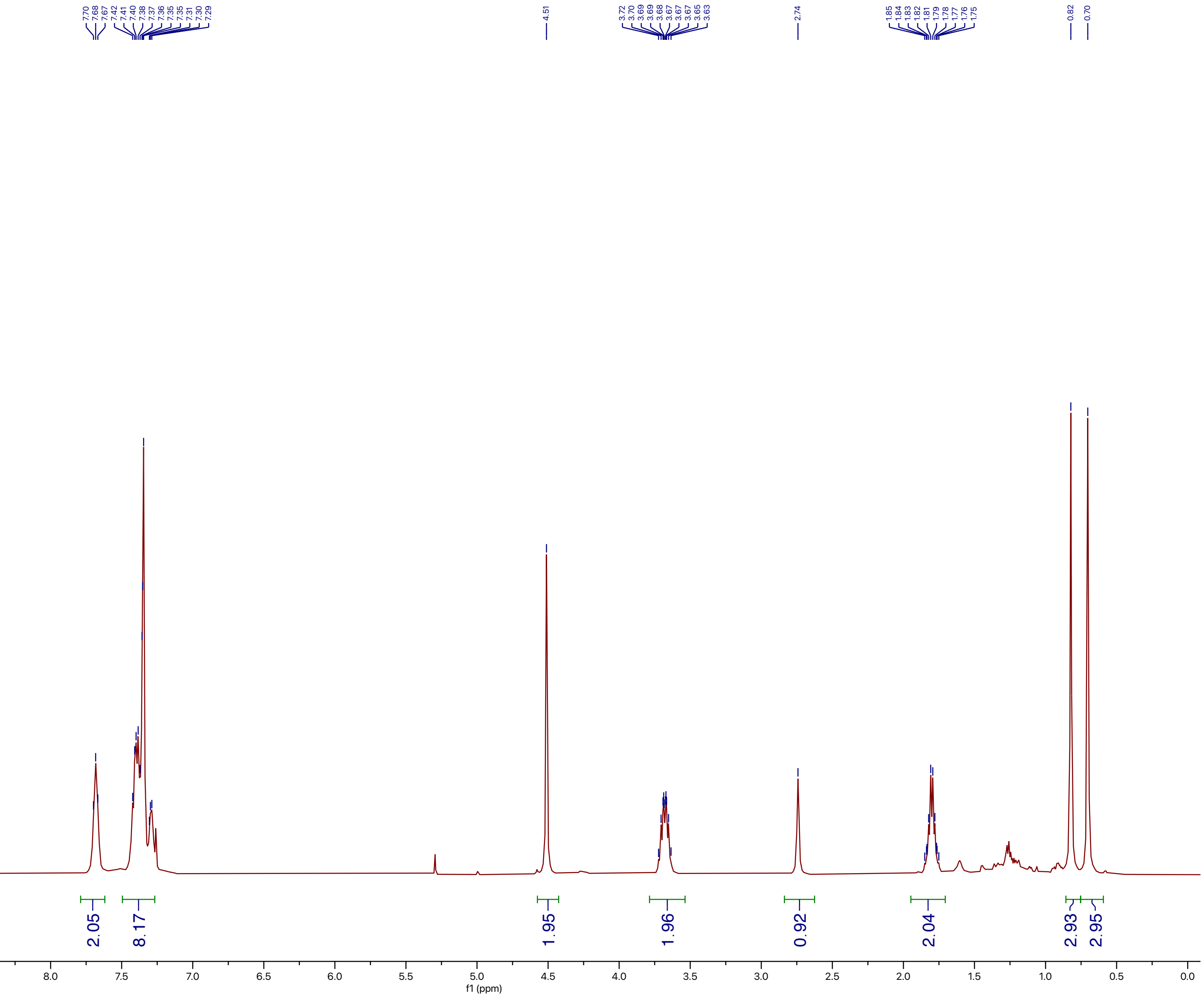

^{13}C NMR of 3v ($CDCl_3$, 126 MHz)

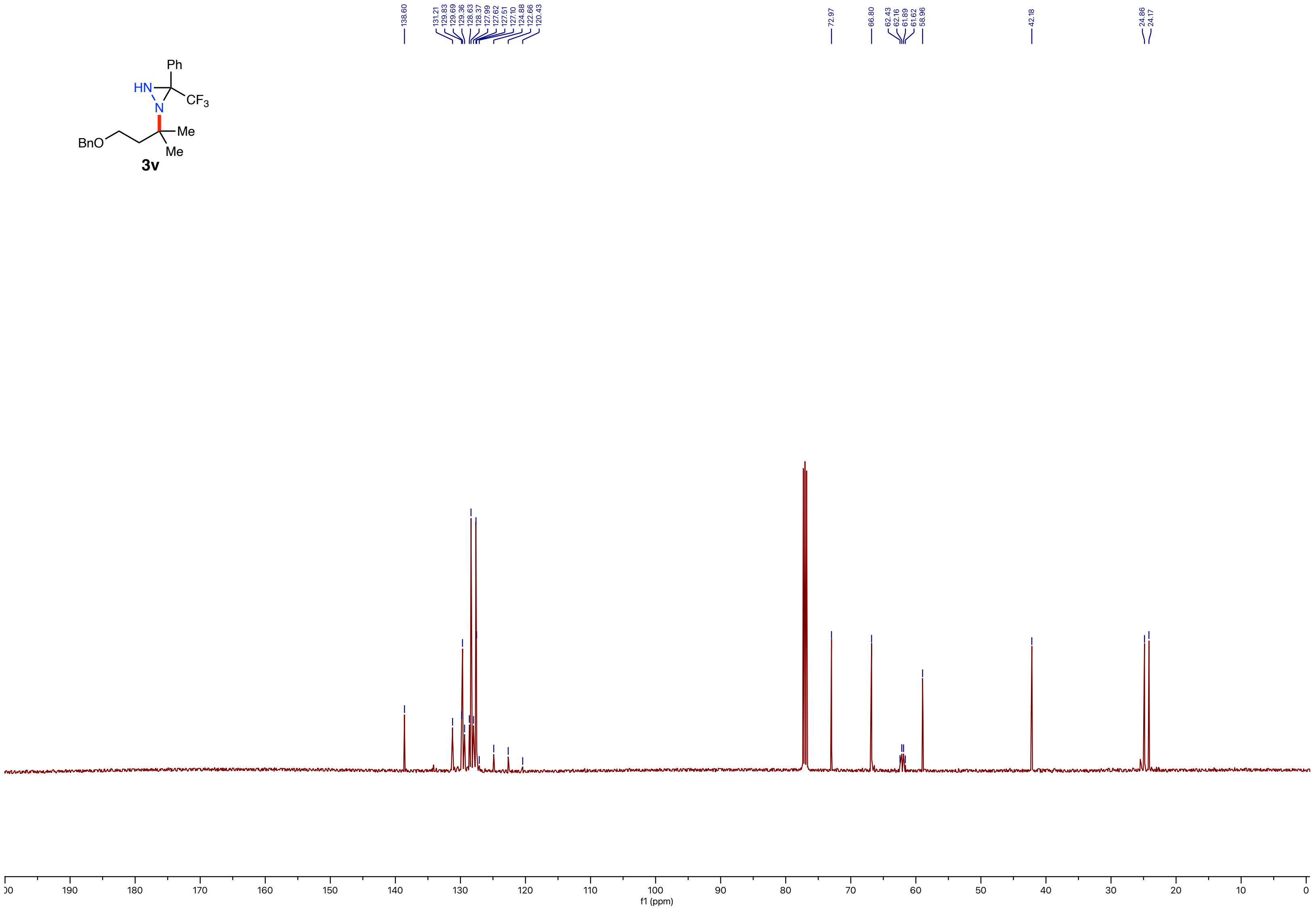

^{19}F NMR of 3v ($CDCl_3$, 471 MHz)

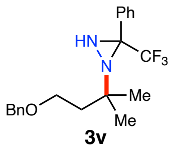

3v

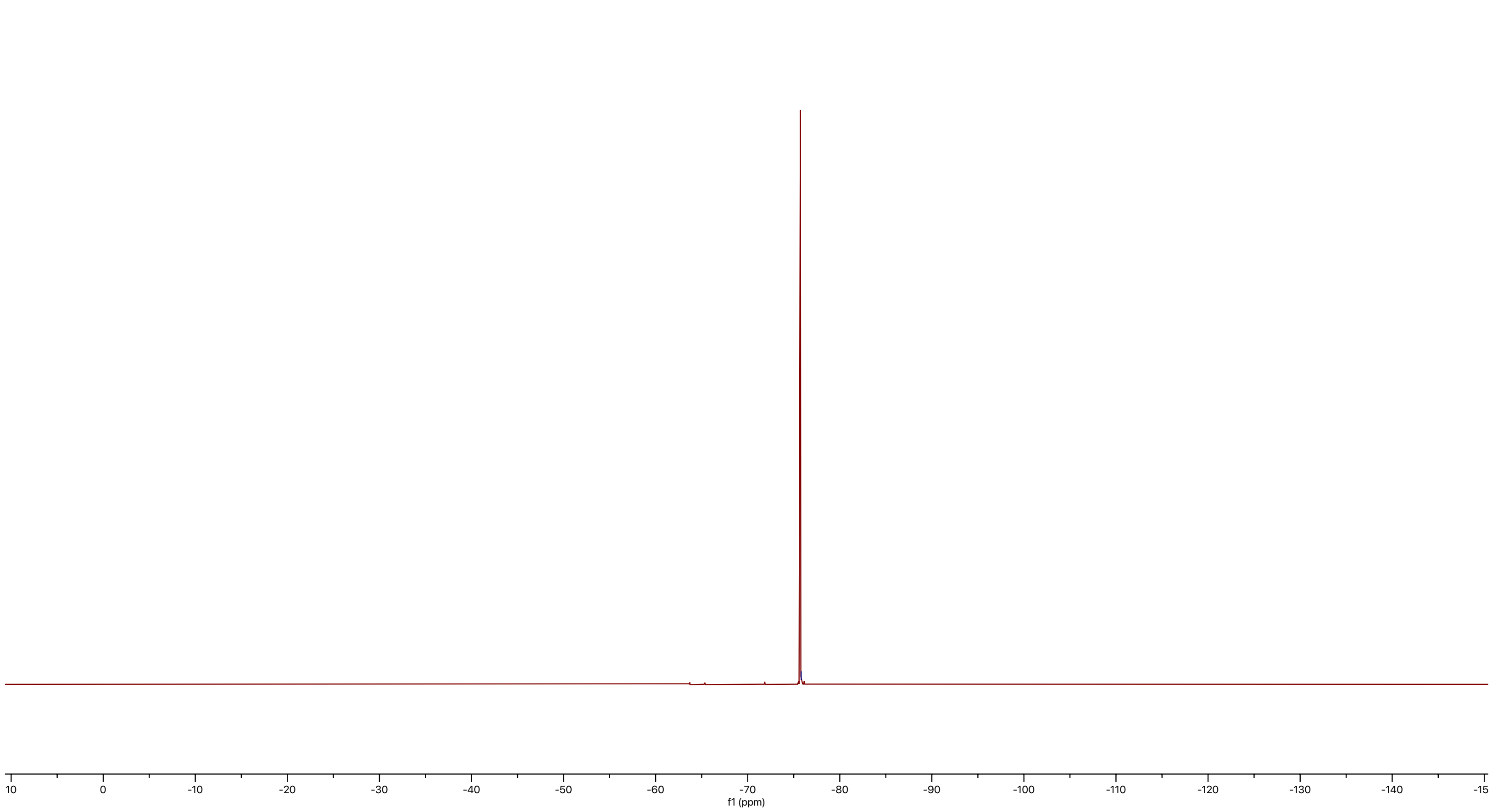

^{1}H NMR of 3w ($CDCl_3$, 500 MHz)

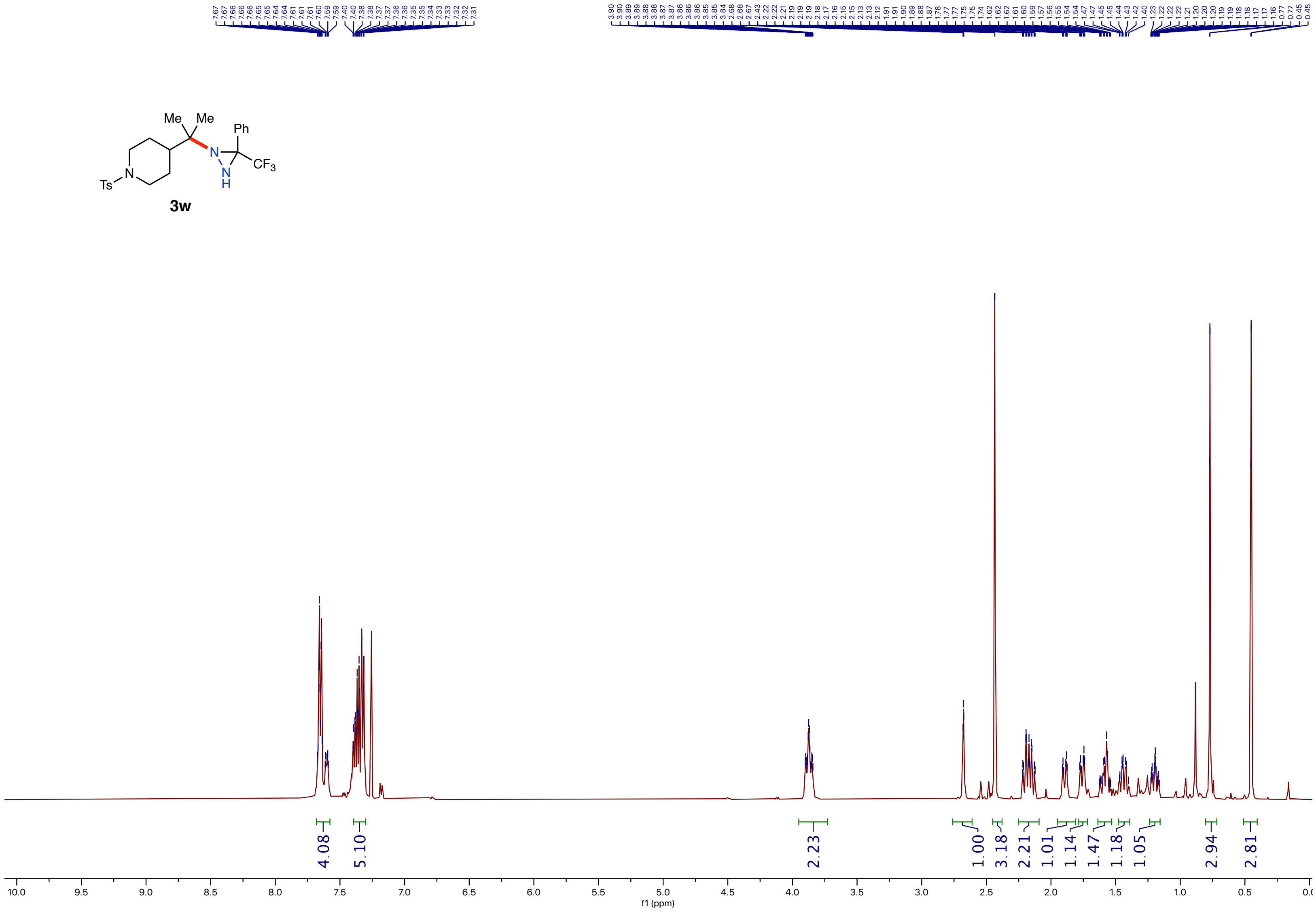

^{13}C NMR of 3w ($CDCl_3$, 126 MHz)

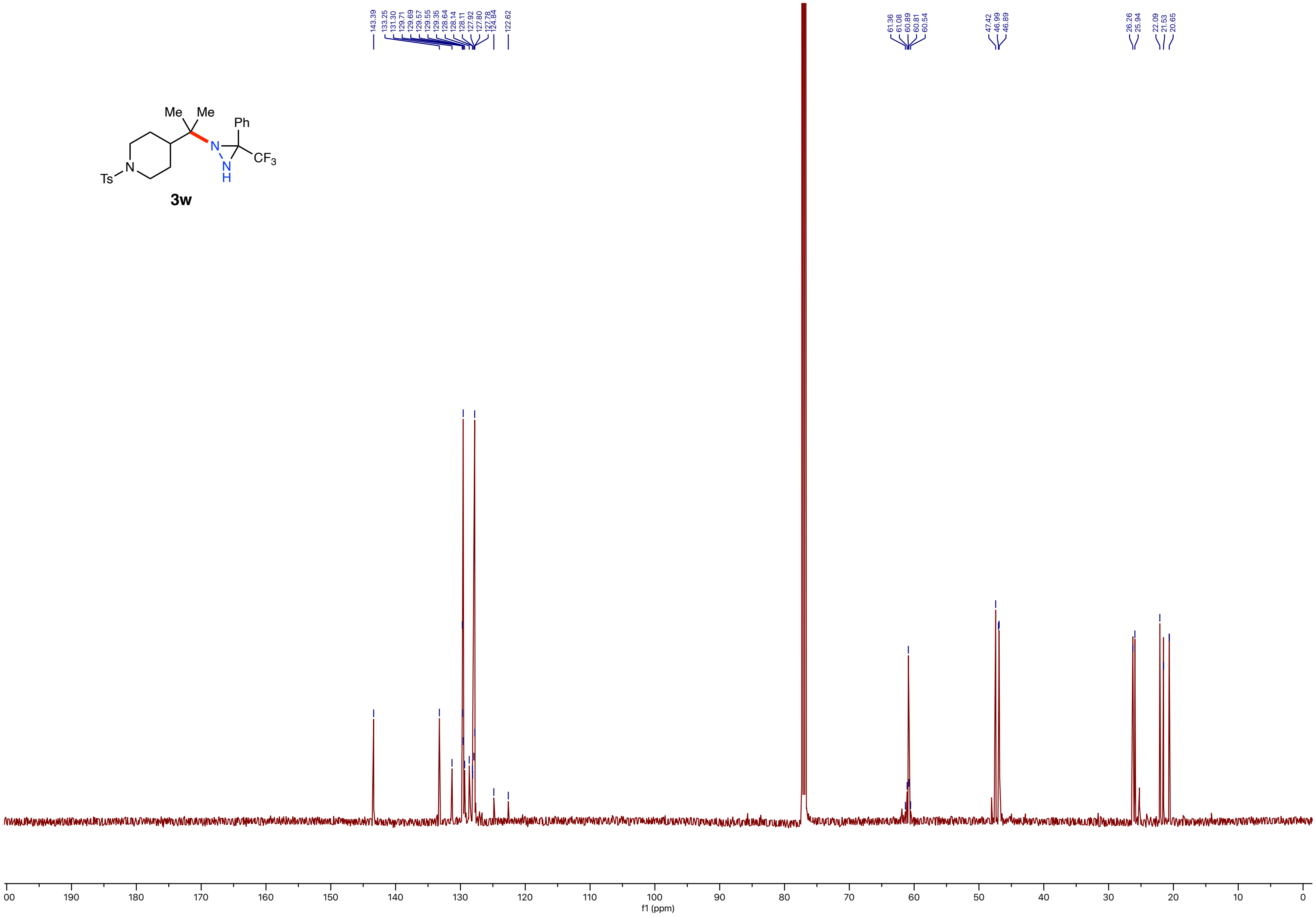

^{19}F NMR of 3w ($CDCl_3$, 471 MHz)

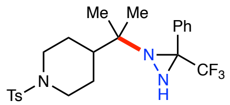

3w

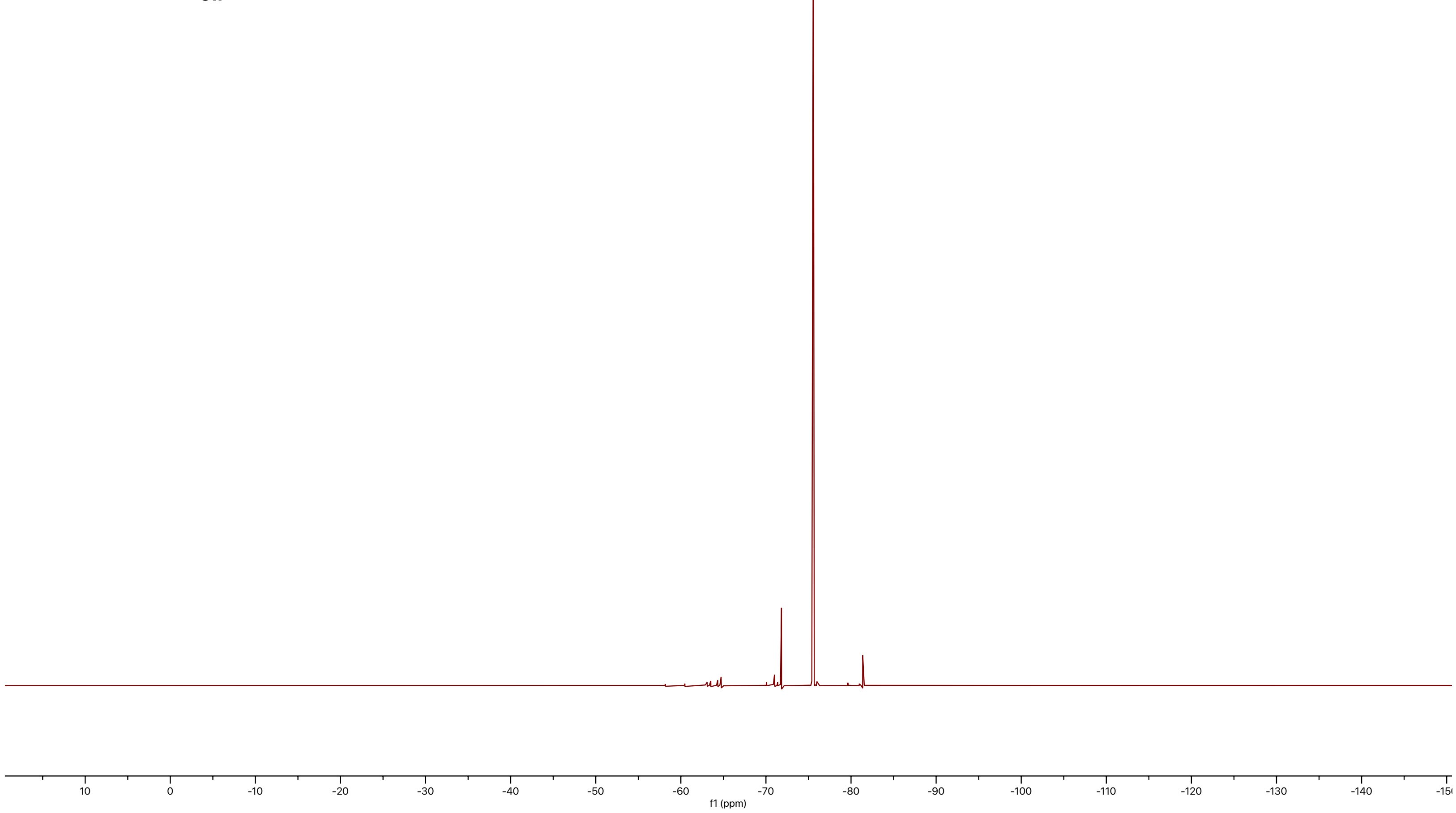

^{1}H NMR of 3x ($CDCl_3$, 500 MHz)

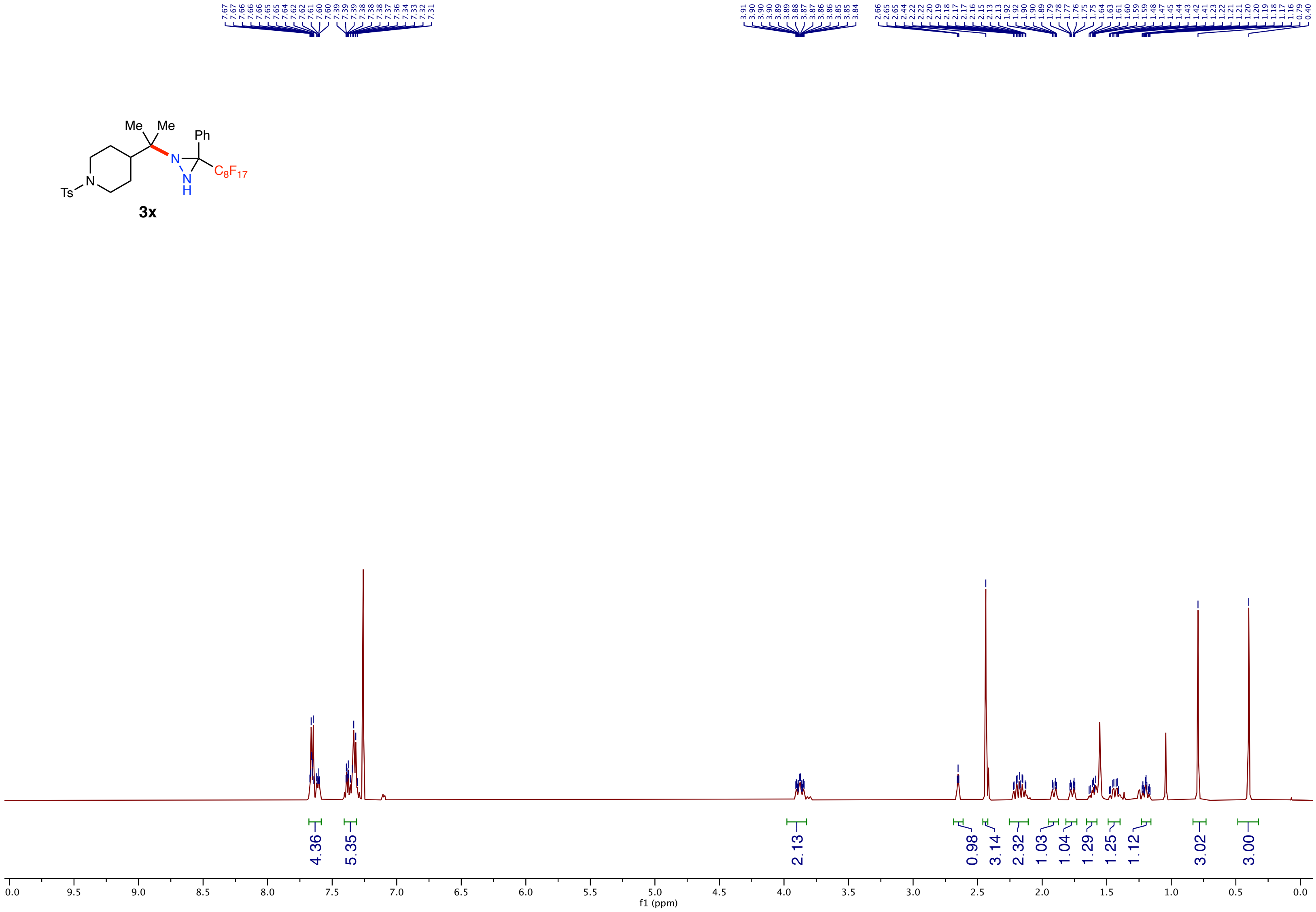

^{13}C NMR of 3x ($CDCl_3$, 126 MHz)

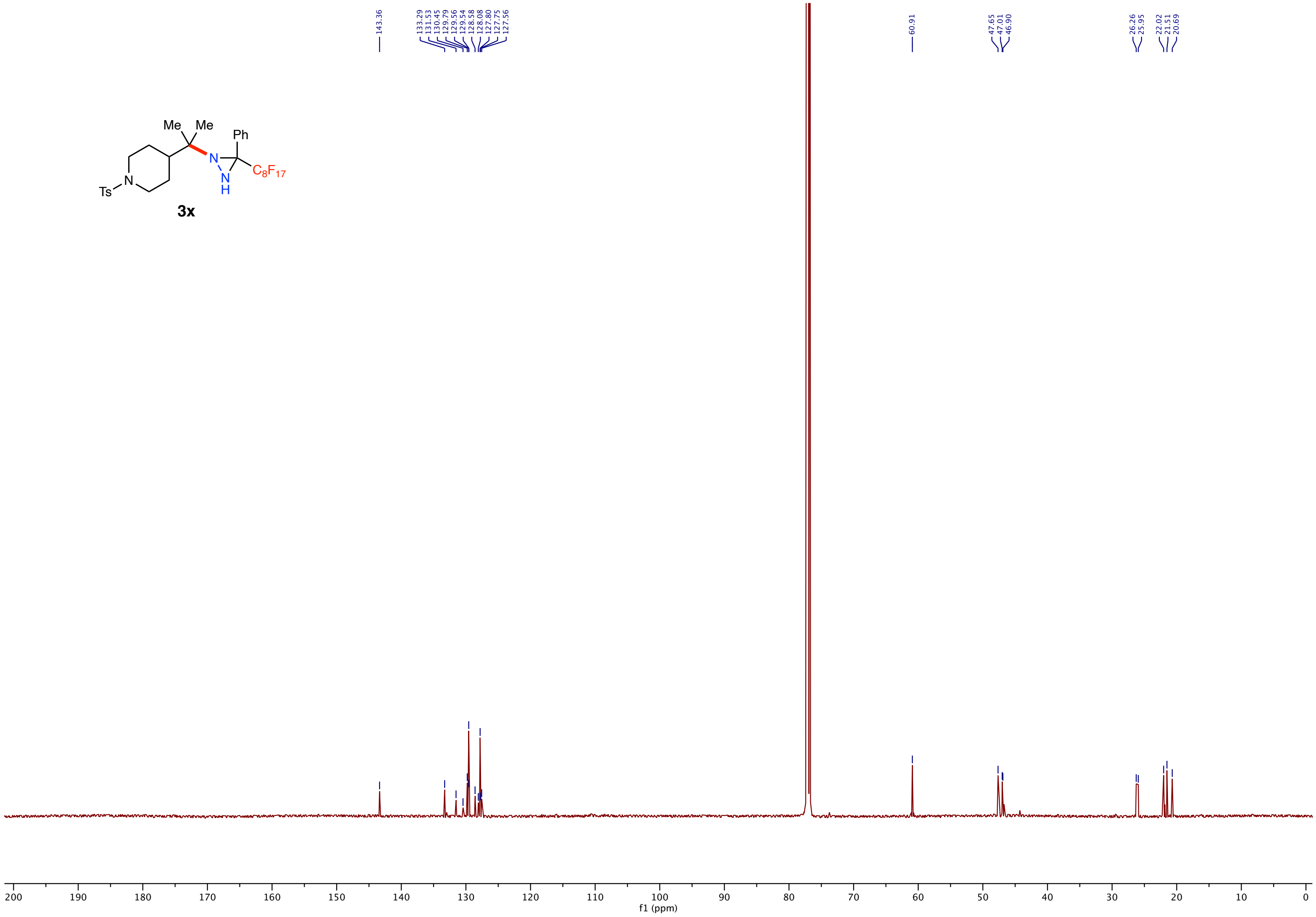

^{19}F NMR of 3x ($CDCl_3$, 471 MHz)

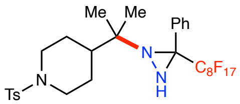

3x

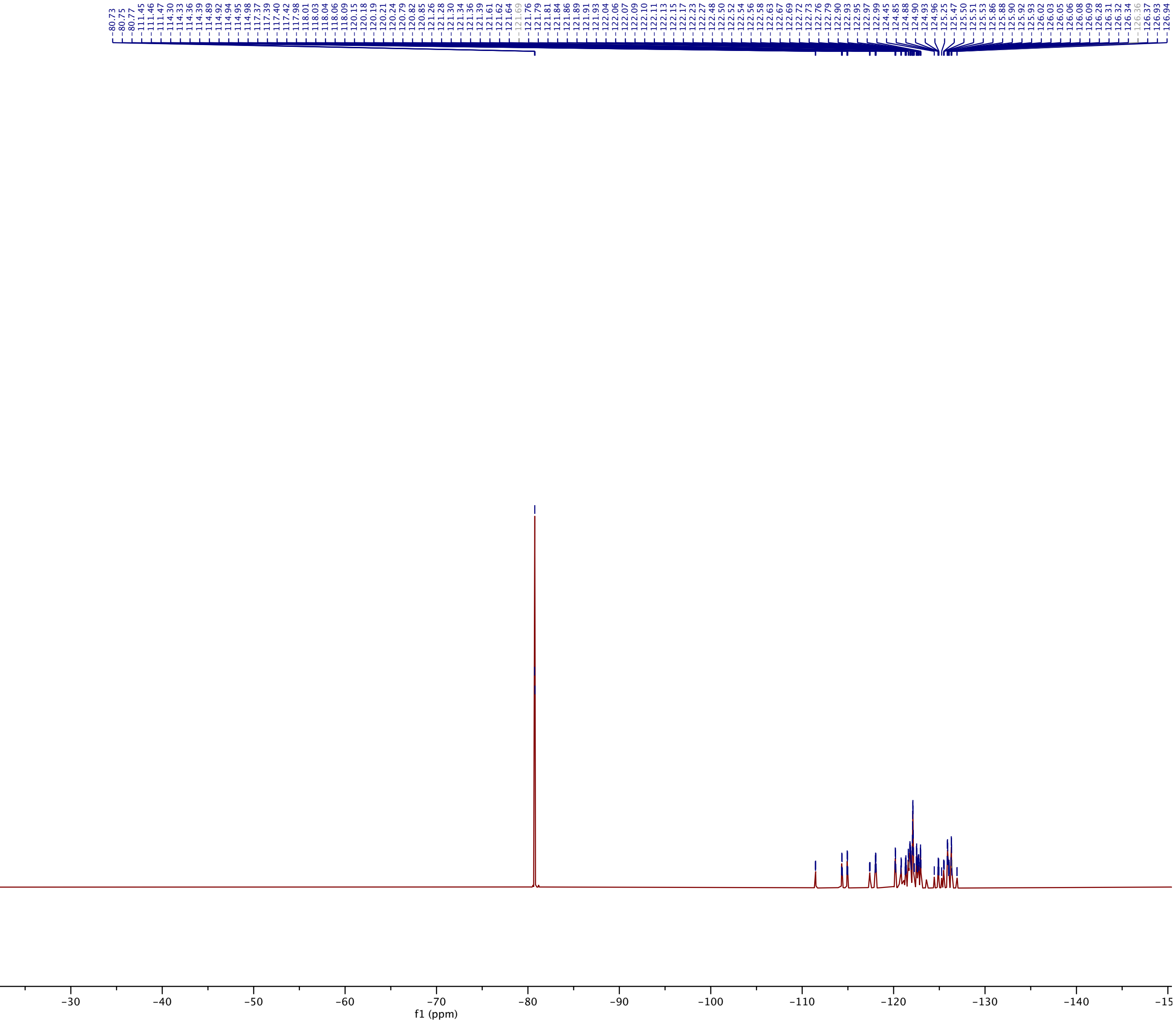

^{1}H NMR of 3y ($CDCl_3$, 500 MHz)

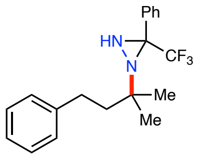

3y

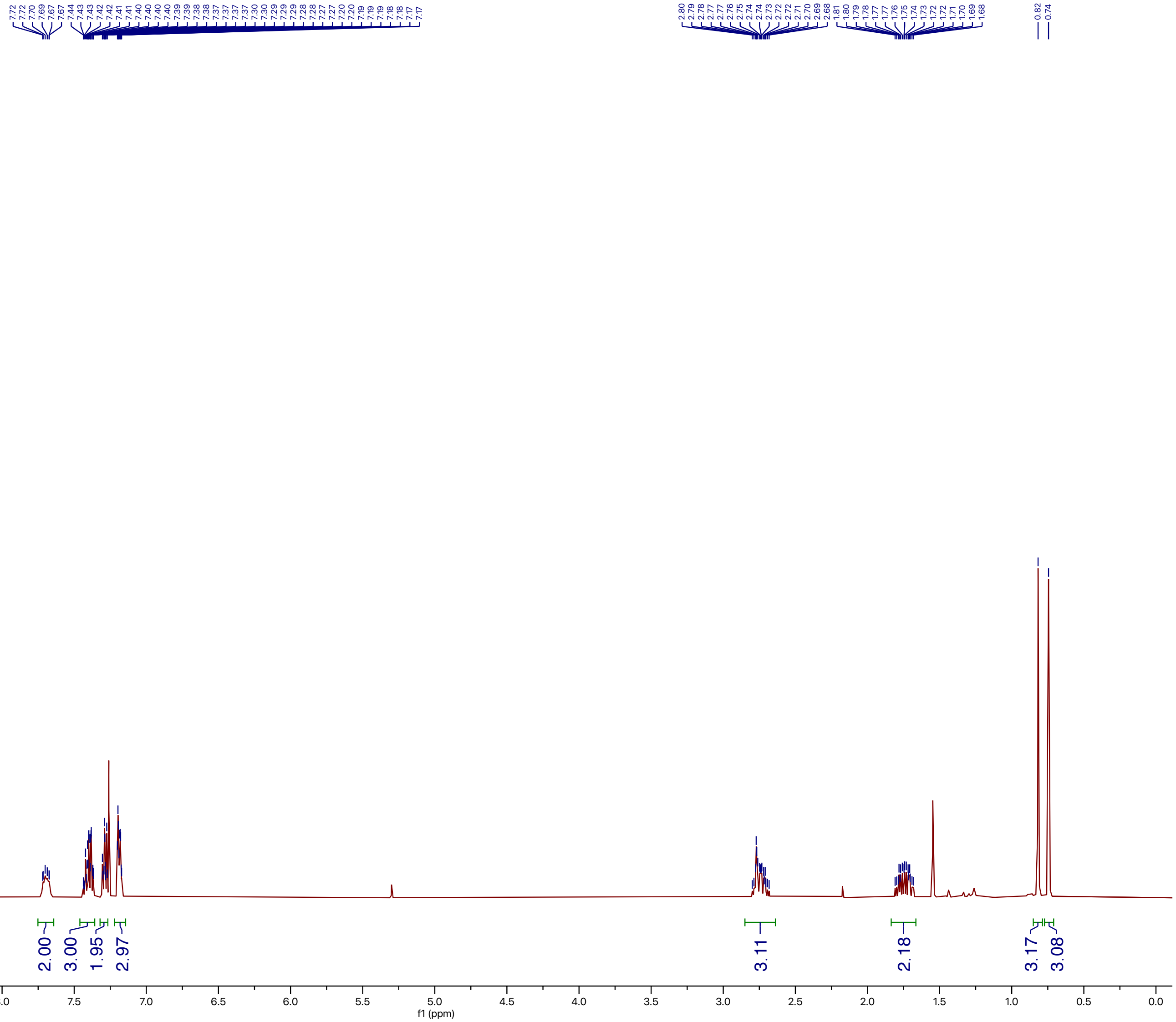

^{13}C NMR of 3y ($CDCl_3$, 126 MHz)

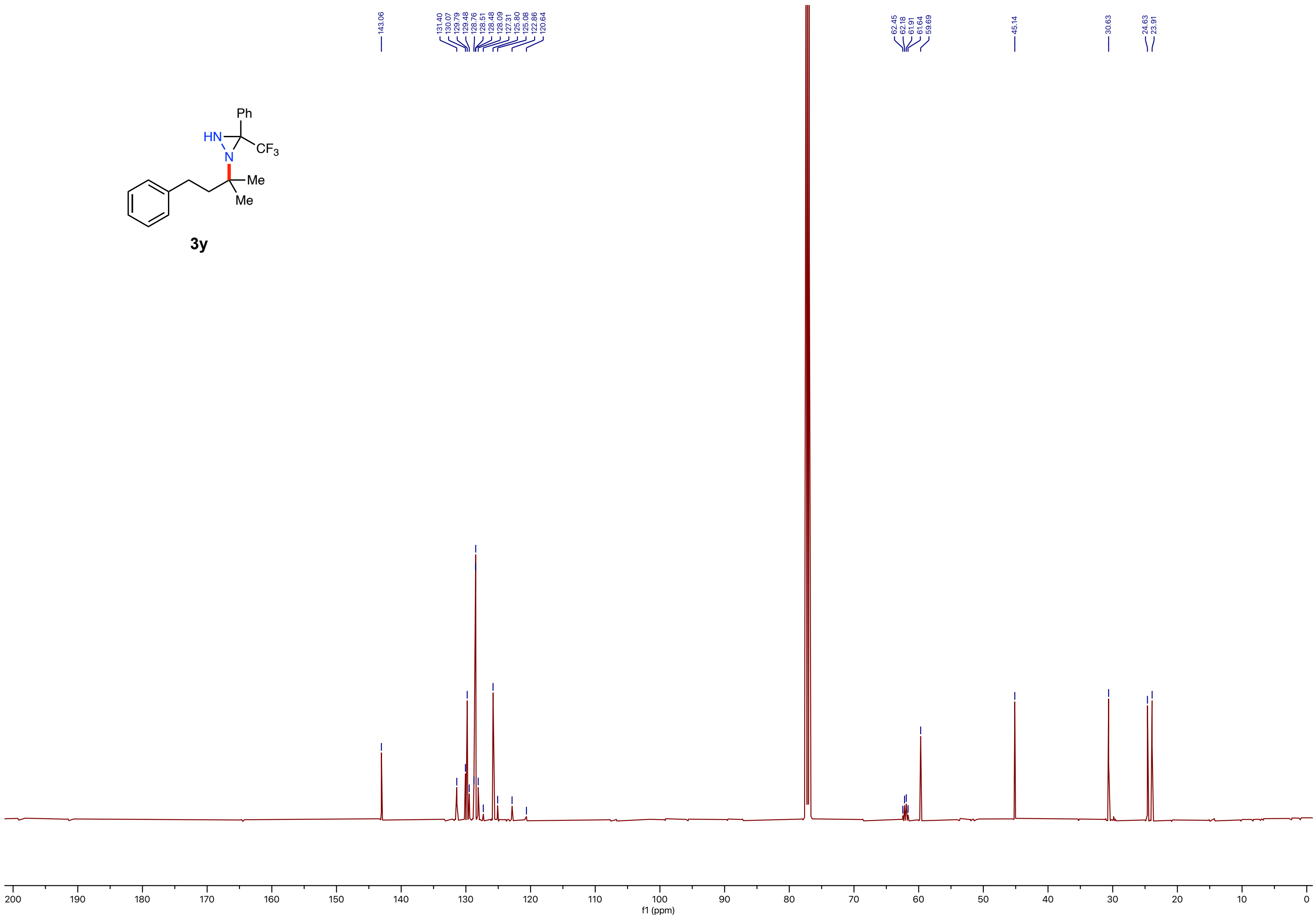

^{19}F NMR of 3y ($CDCl_3$, 471 MHz)

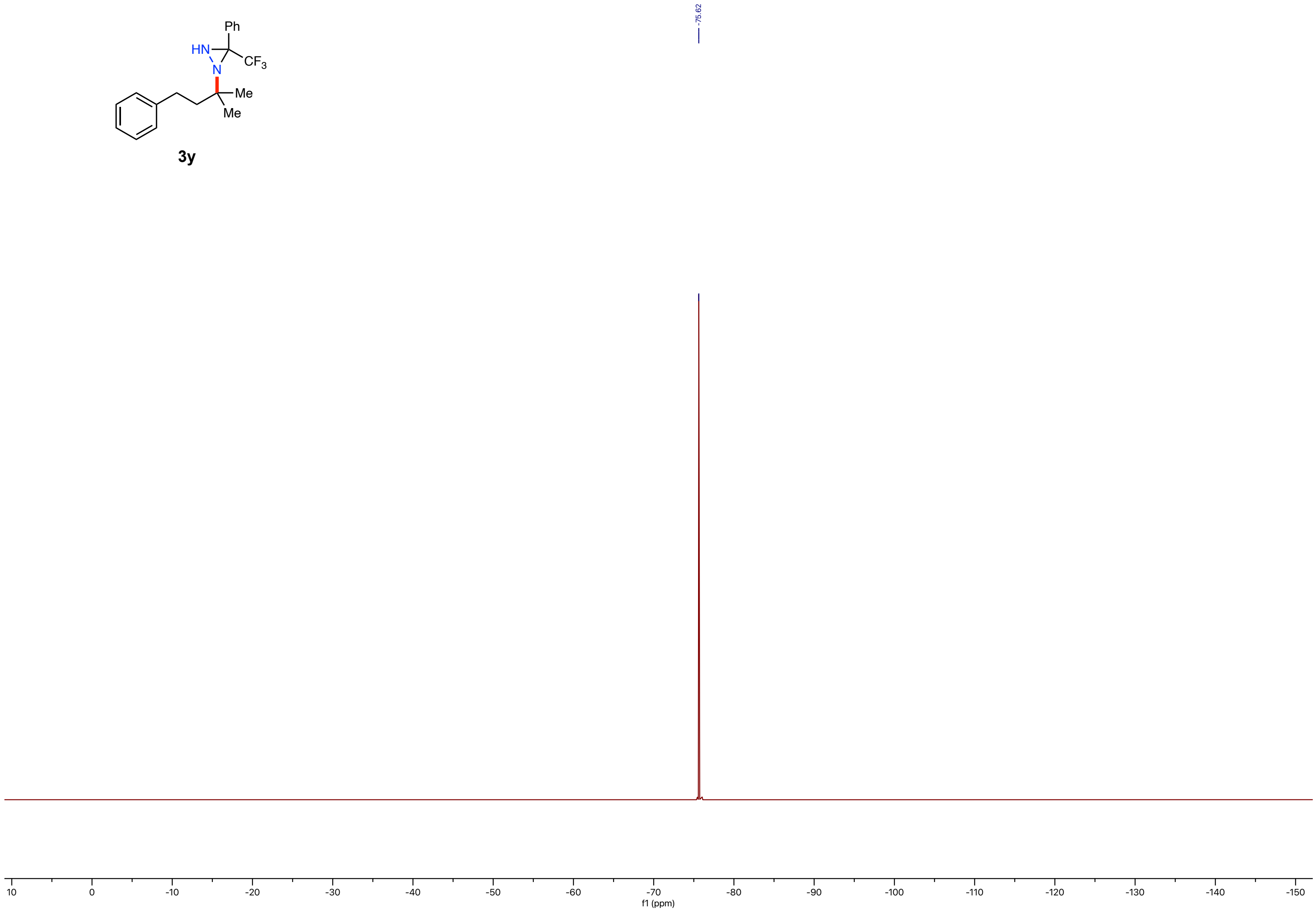

^{1}H NMR of 3z ($CDCl_3$, 500 MHz)

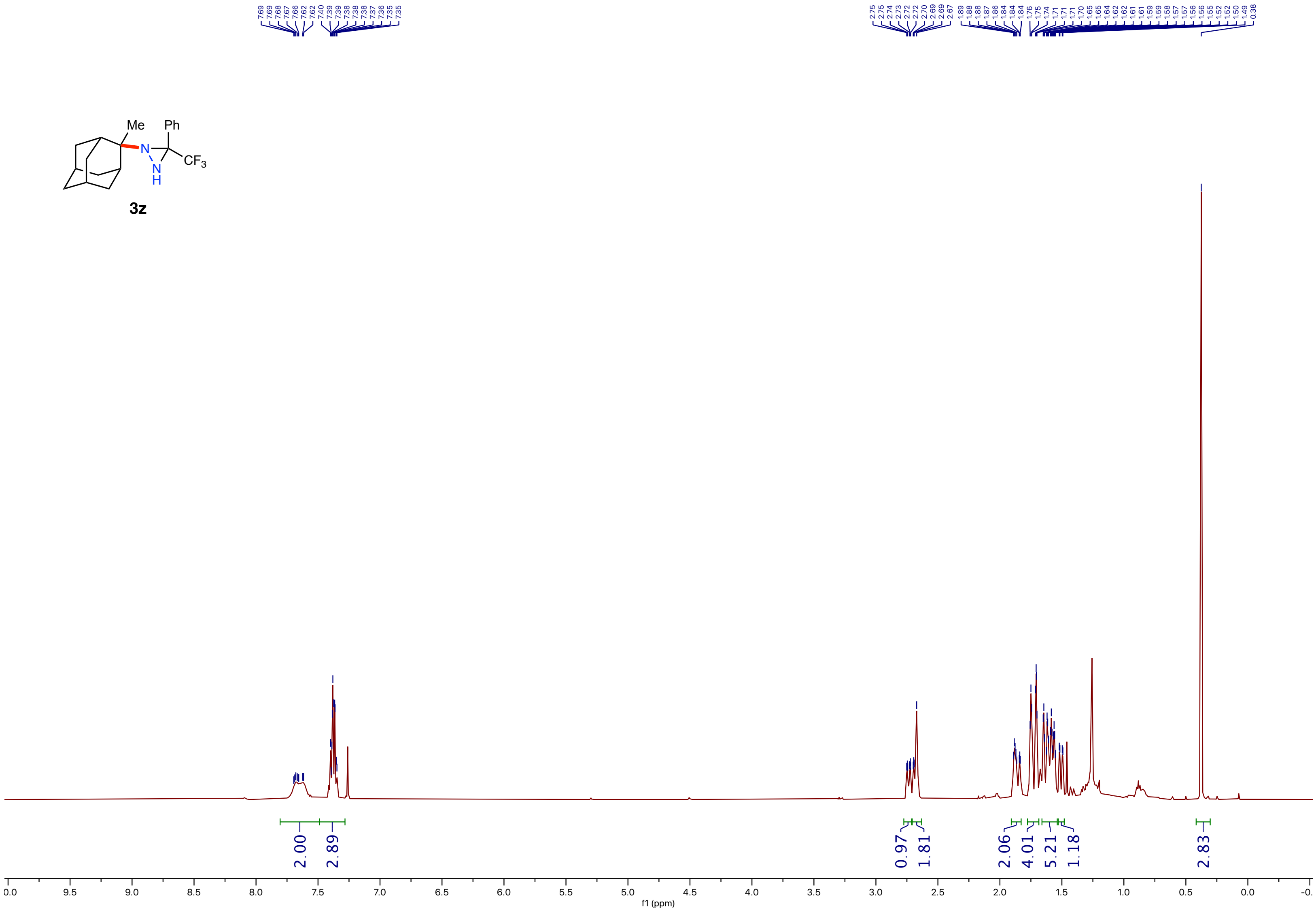

^{13}C NMR of 3z ($CDCl_3$, 126 MHz)

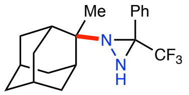

3z

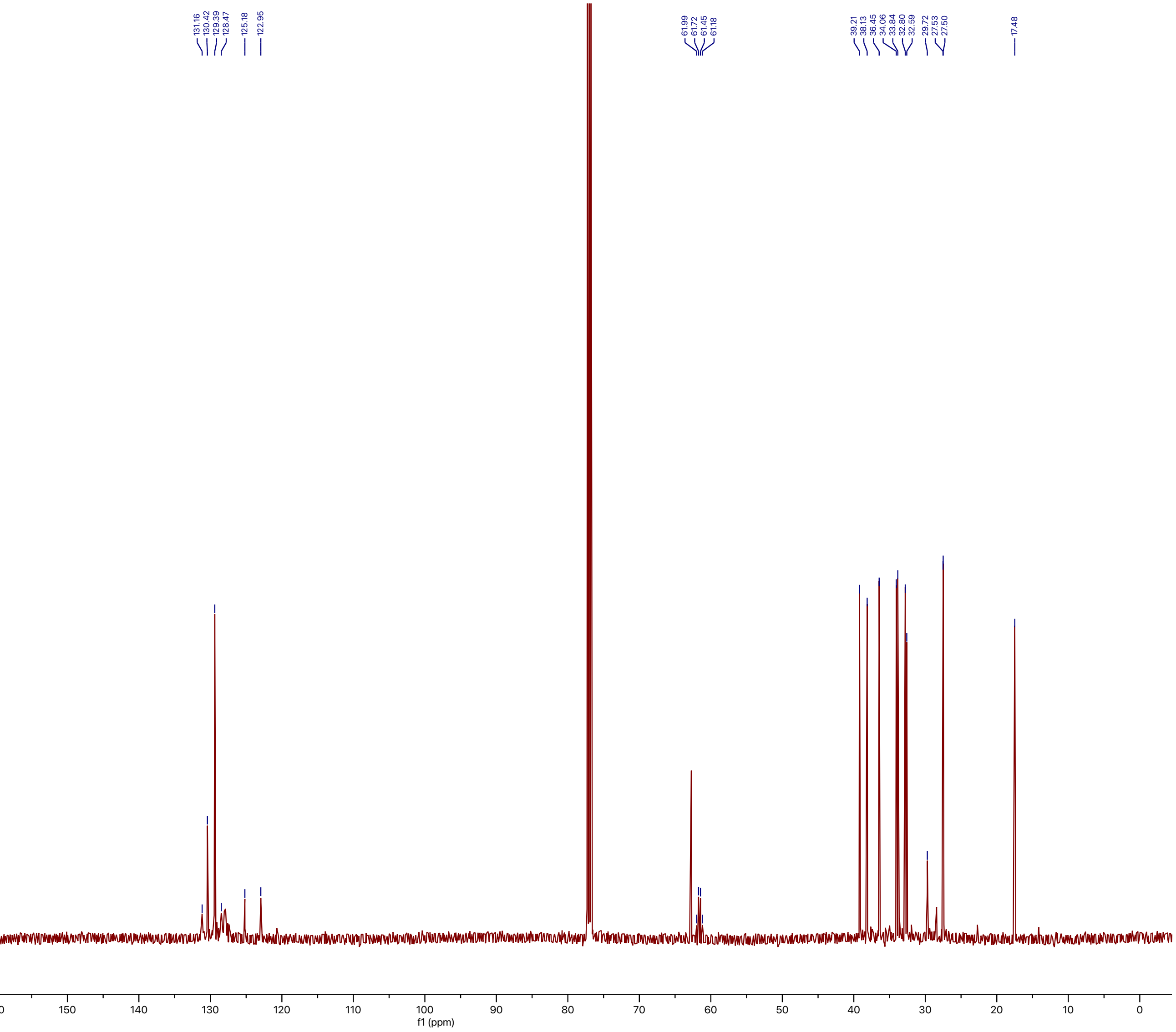

^{19}F NMR of 3z ($CDCl_3$, 471 MHz)

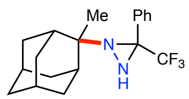

3z

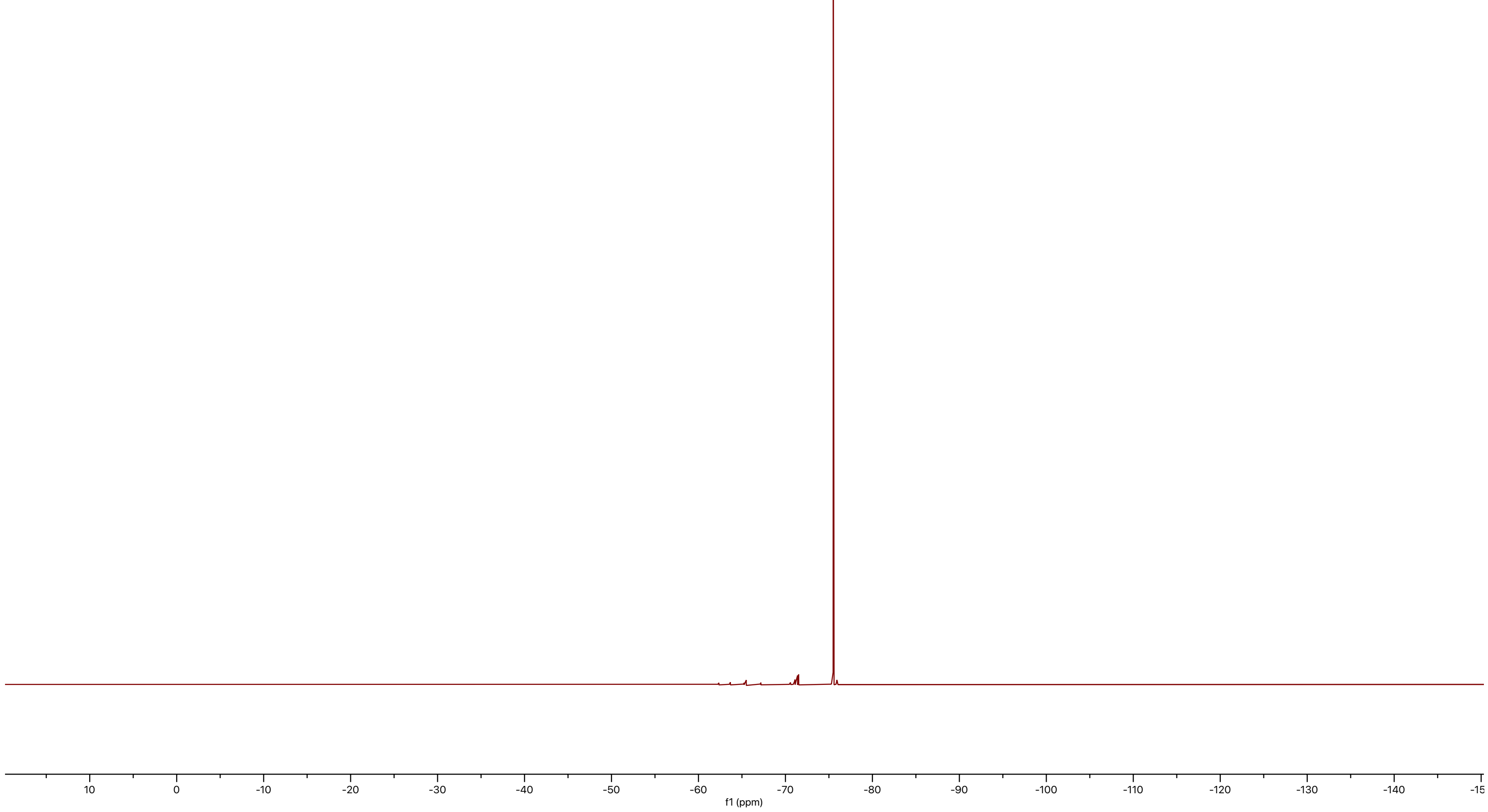

^{1}H NMR of 3aa ($CDCl_3$, 500 MHz)

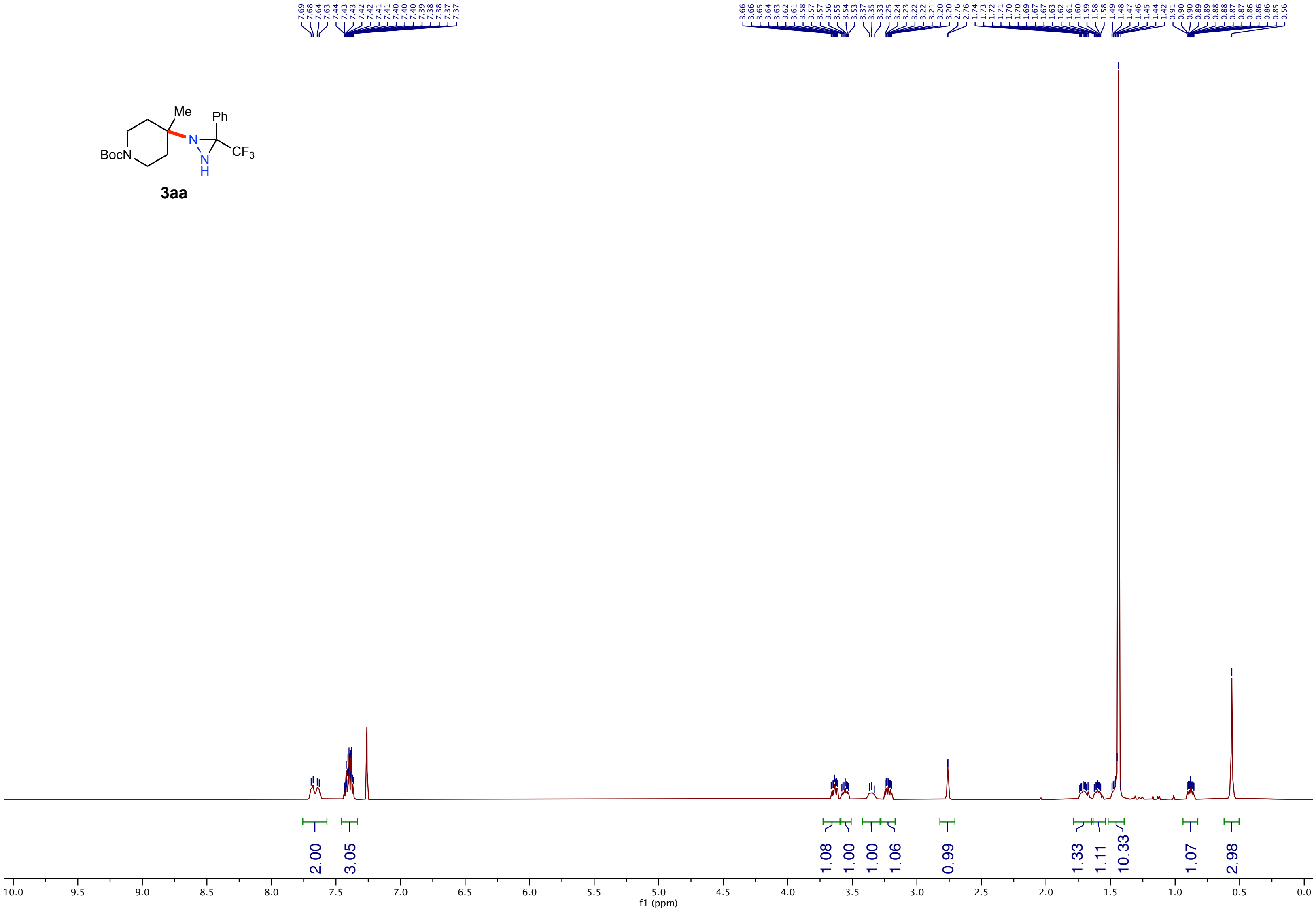

^{13}C NMR of 3aa ($CDCl_3$, 126 MHz)

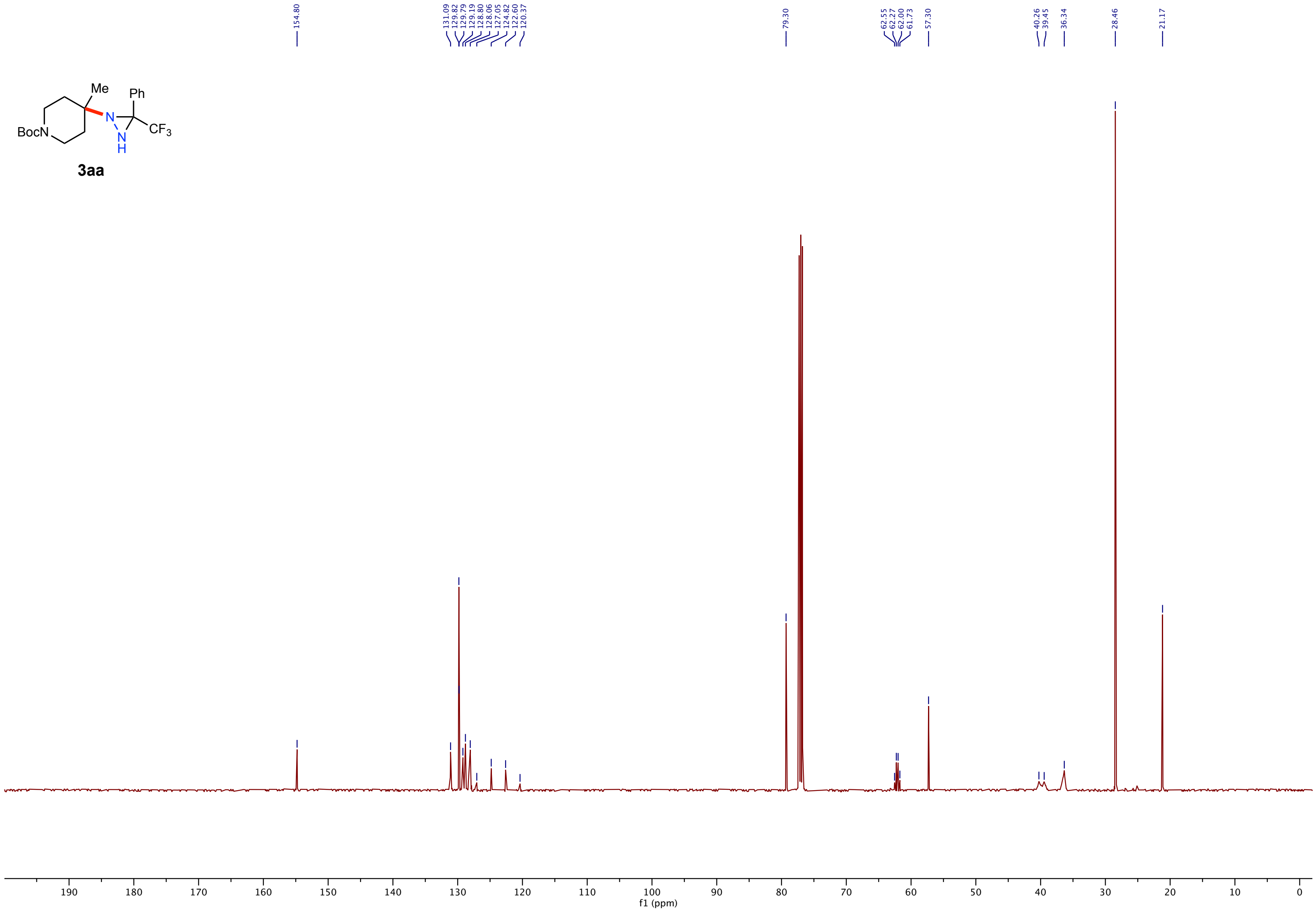

^{19}F NMR of 3aa ($CDCl_3$, 471 MHz)

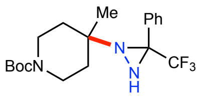

3aa

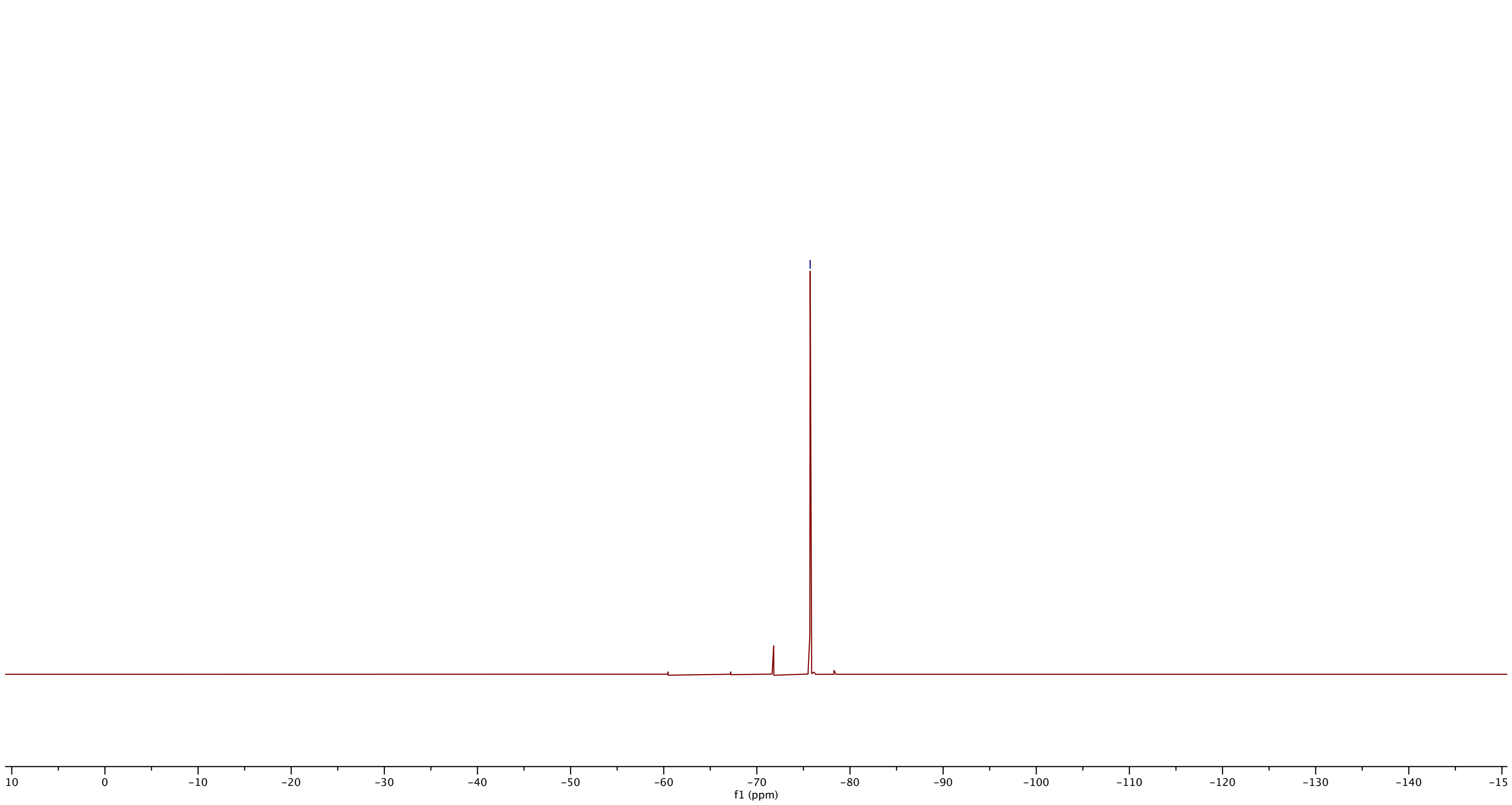

^{1}H NMR of 3ab ($CDCl_3$, 500 MHz)

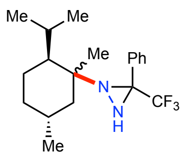

3ab

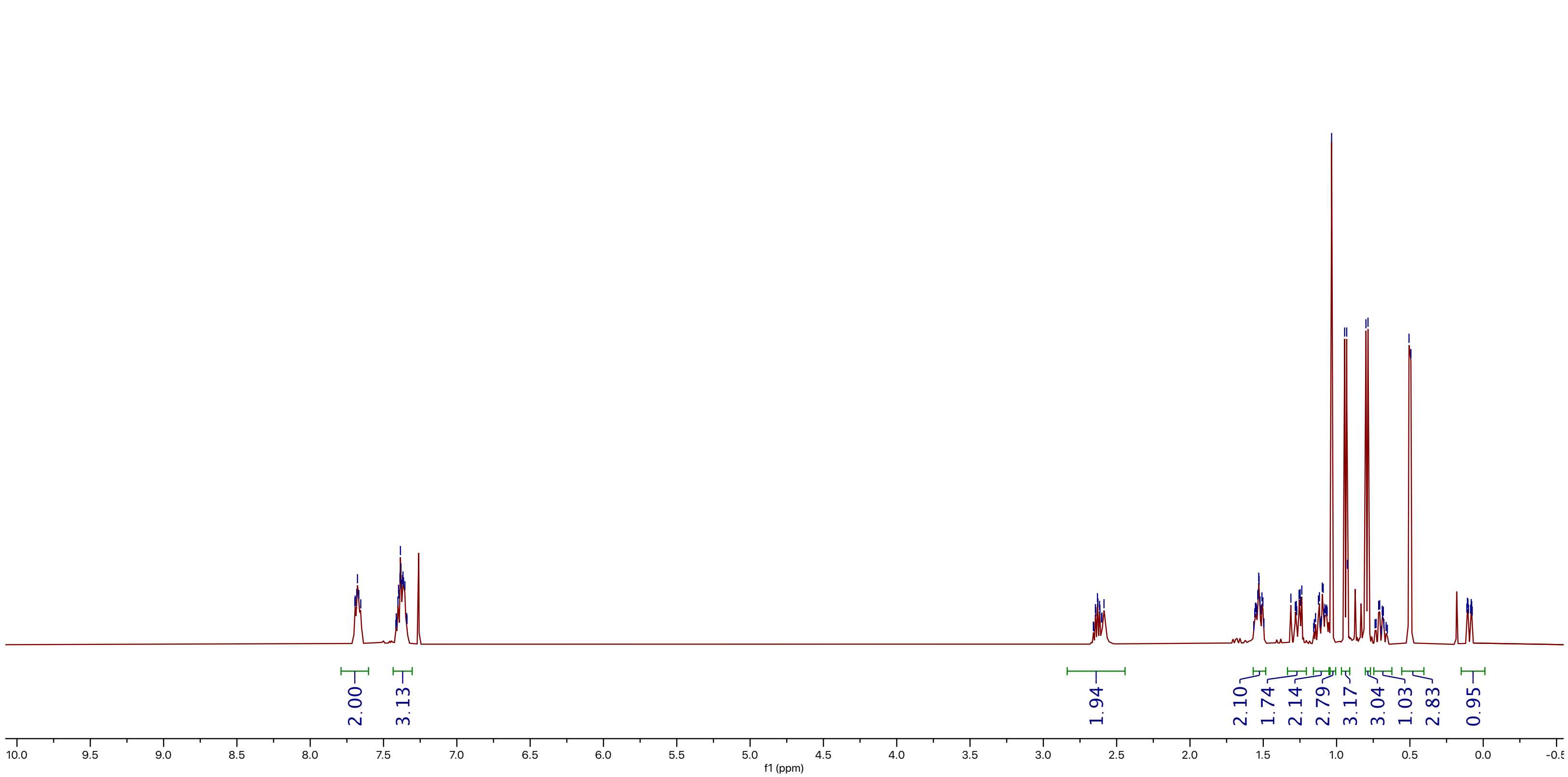

^{13}C NMR of 3ab ($CDCl_3$, 126 MHz)

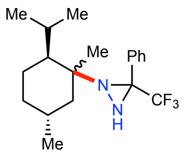

3ab

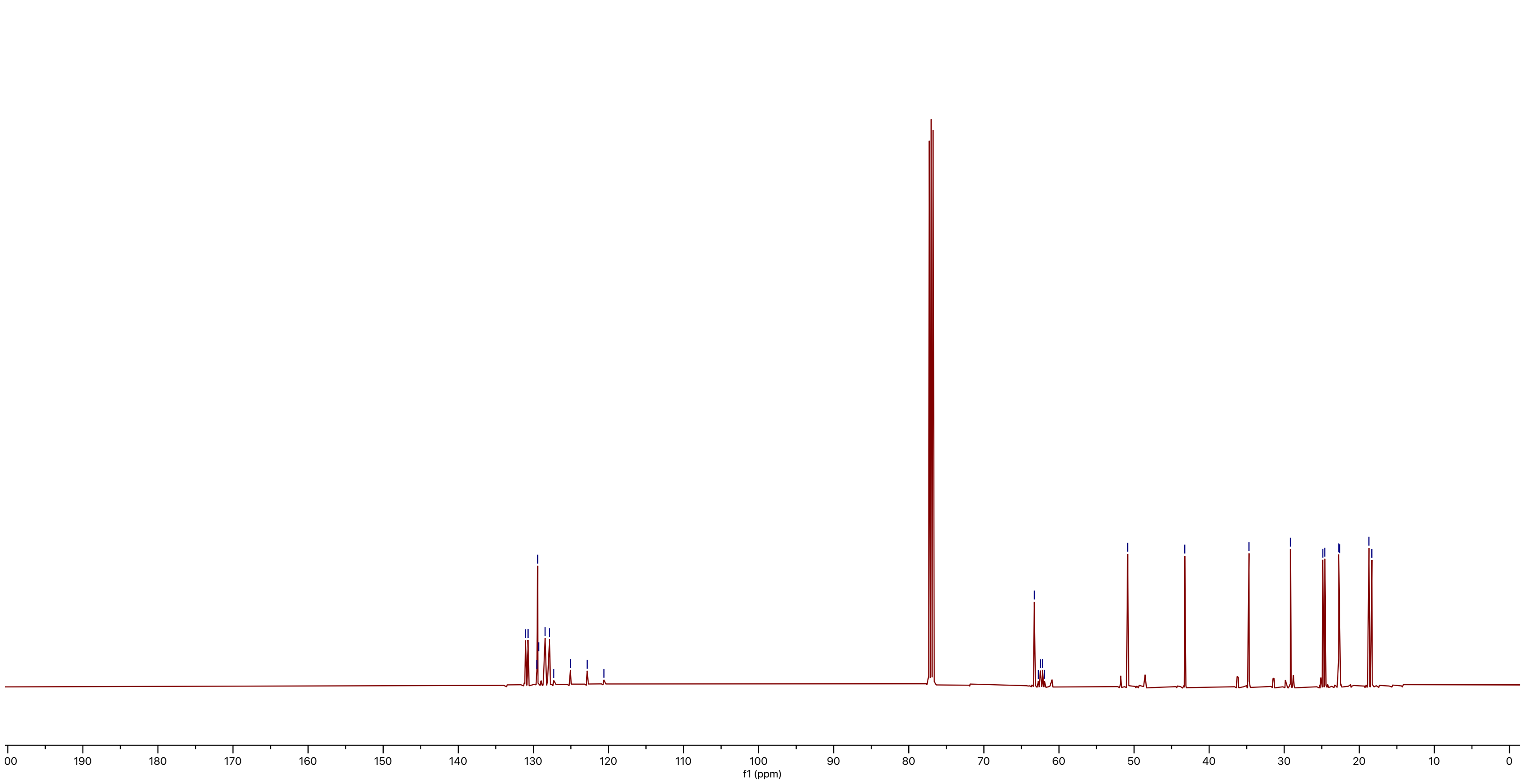

^{19}F NMR of 3ab ($CDCl_3$, 471 MHz)

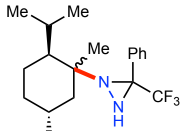

3ab

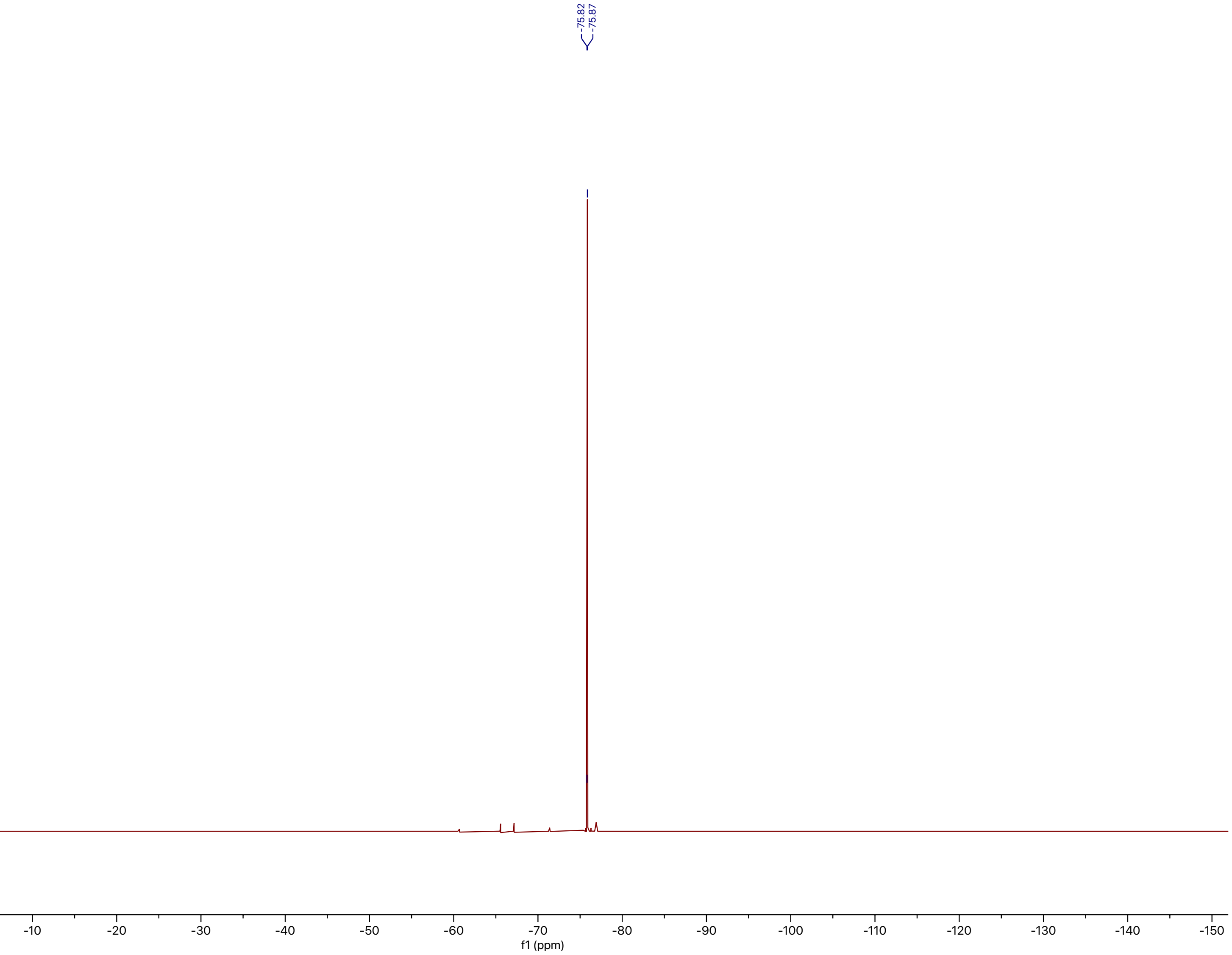

^{1}H NMR of 3ac ($CDCl_3$, 500 MHz)

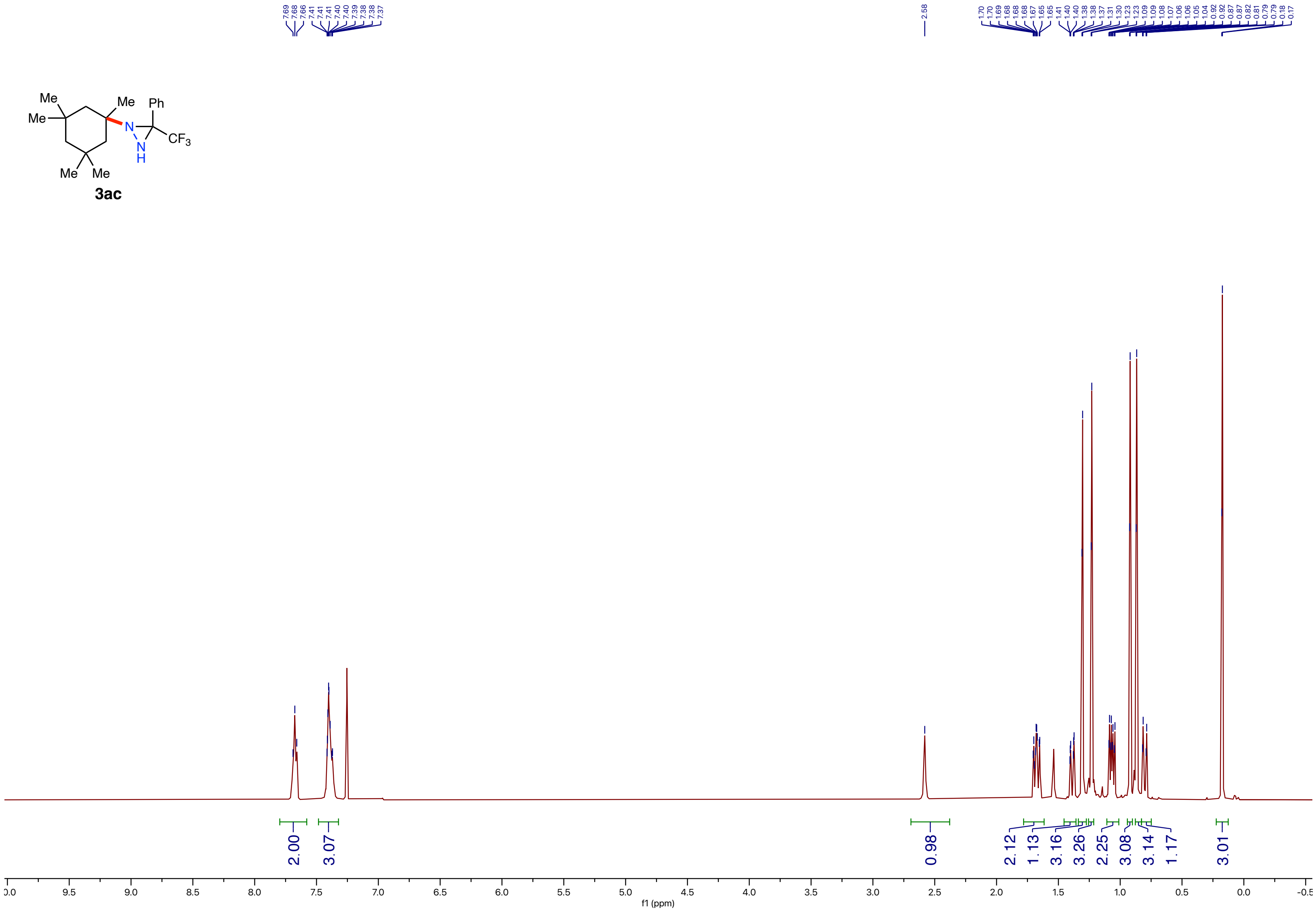

^{13}C NMR of 3ac ($CDCl_3$, 126 MHz)

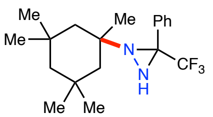

3ac

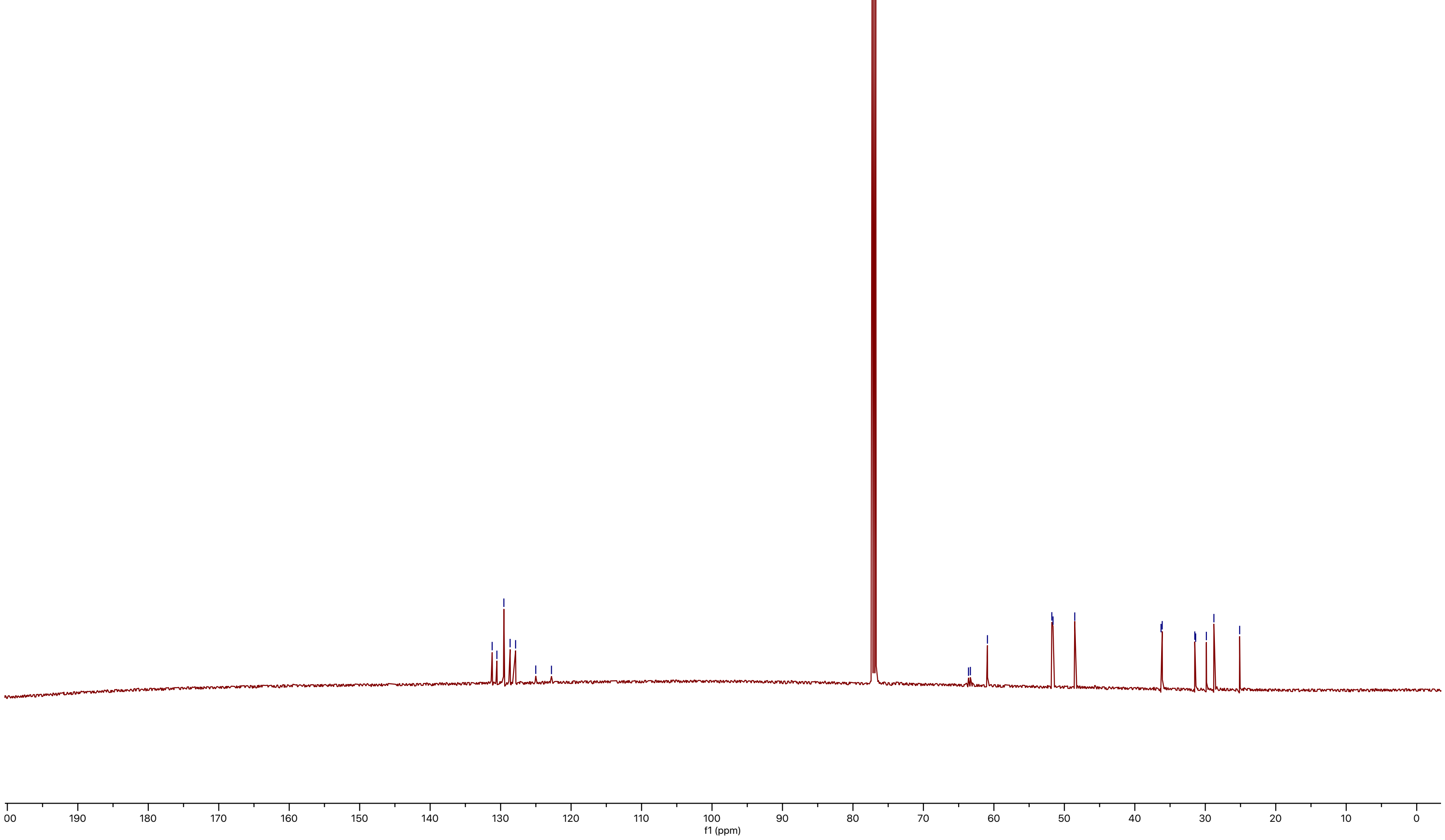

^{19}F NMR of 3ac ($CDCl_3$, 471 MHz)

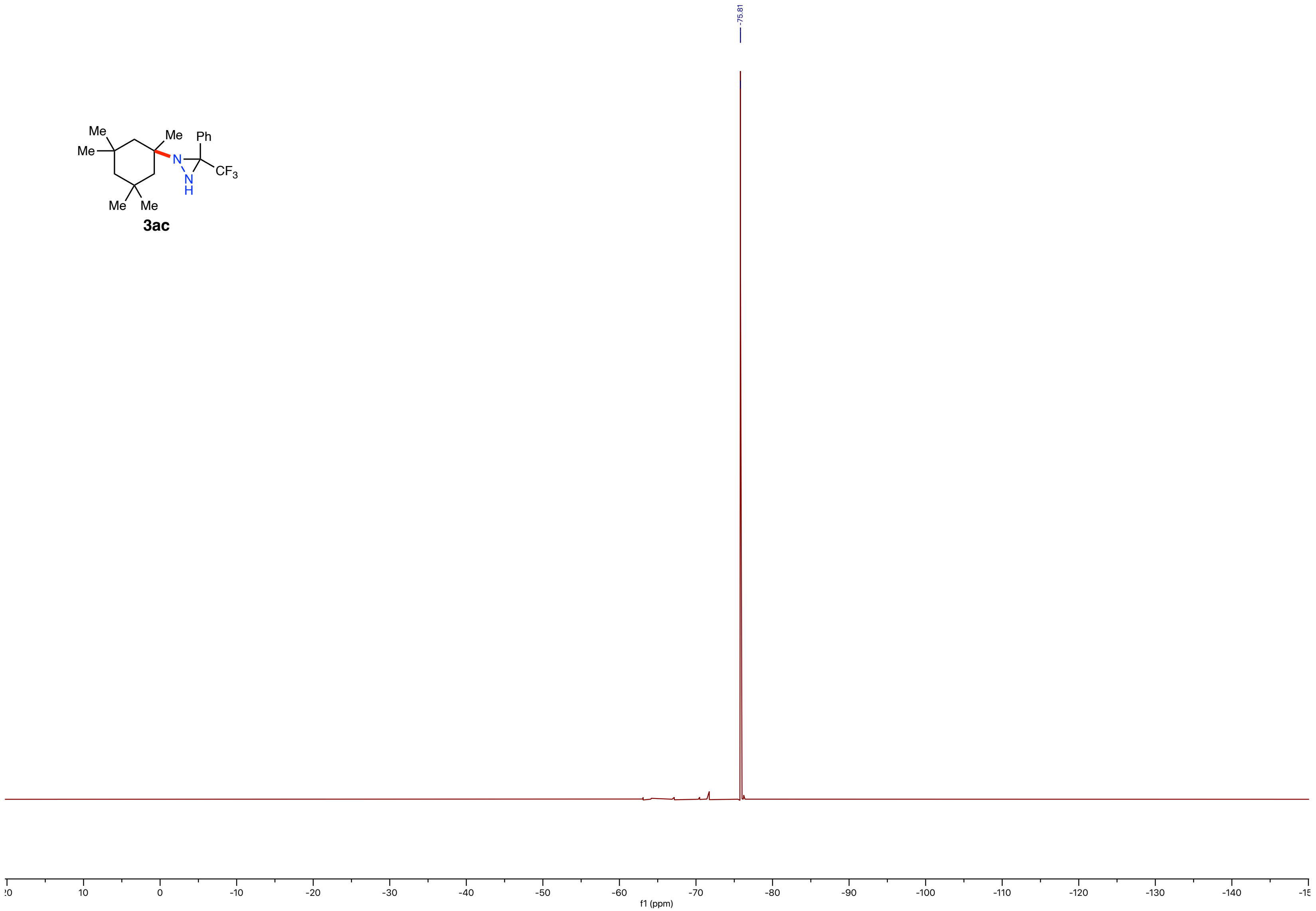

^{1}H NMR of 4a ($CDCl_3$, 500 MHz)

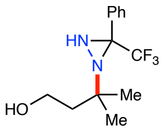

4a

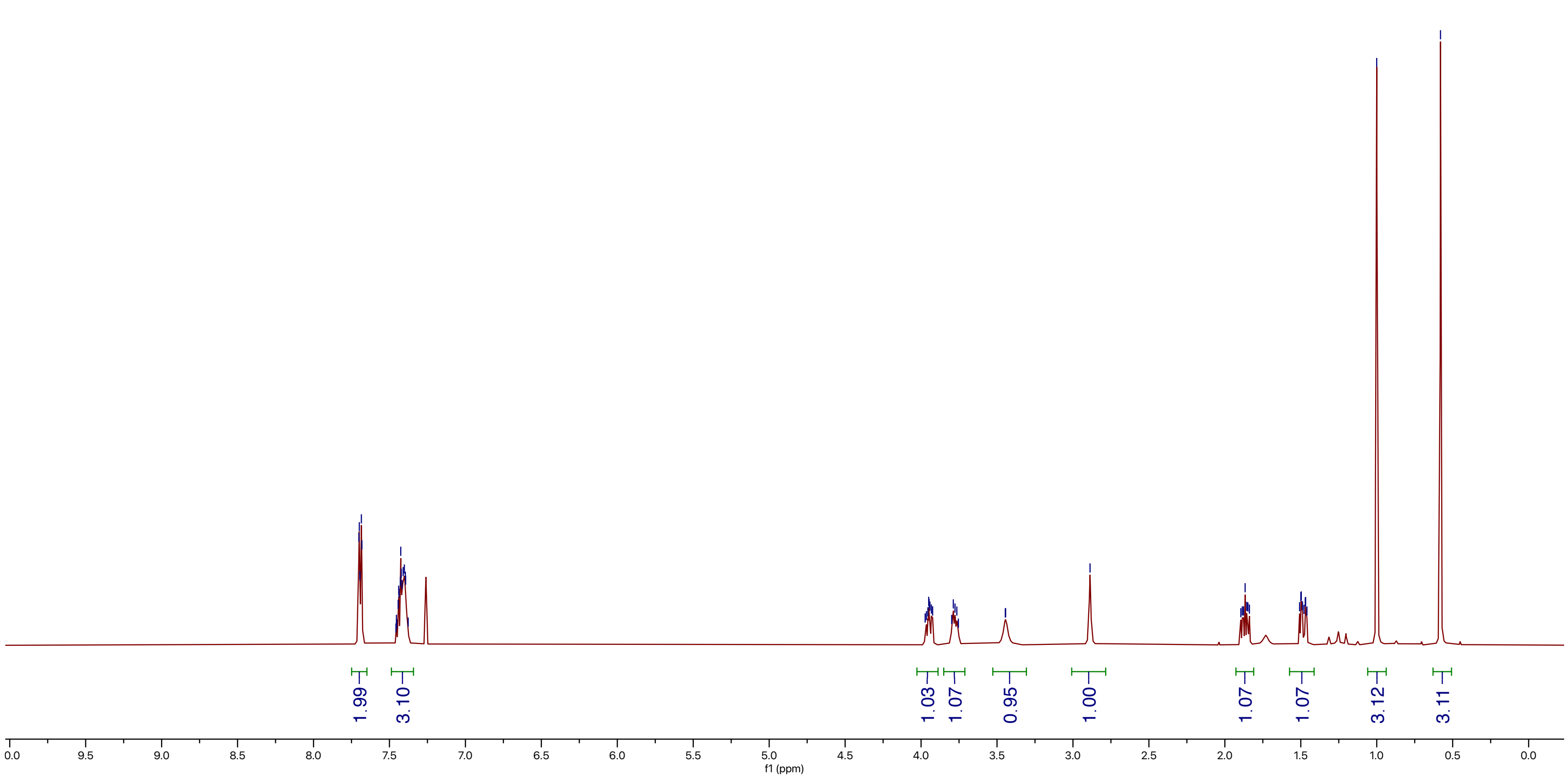

^{13}C NMR of 4a ($CDCl_3$, 126 MHz)

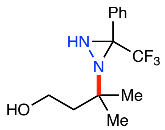

4a

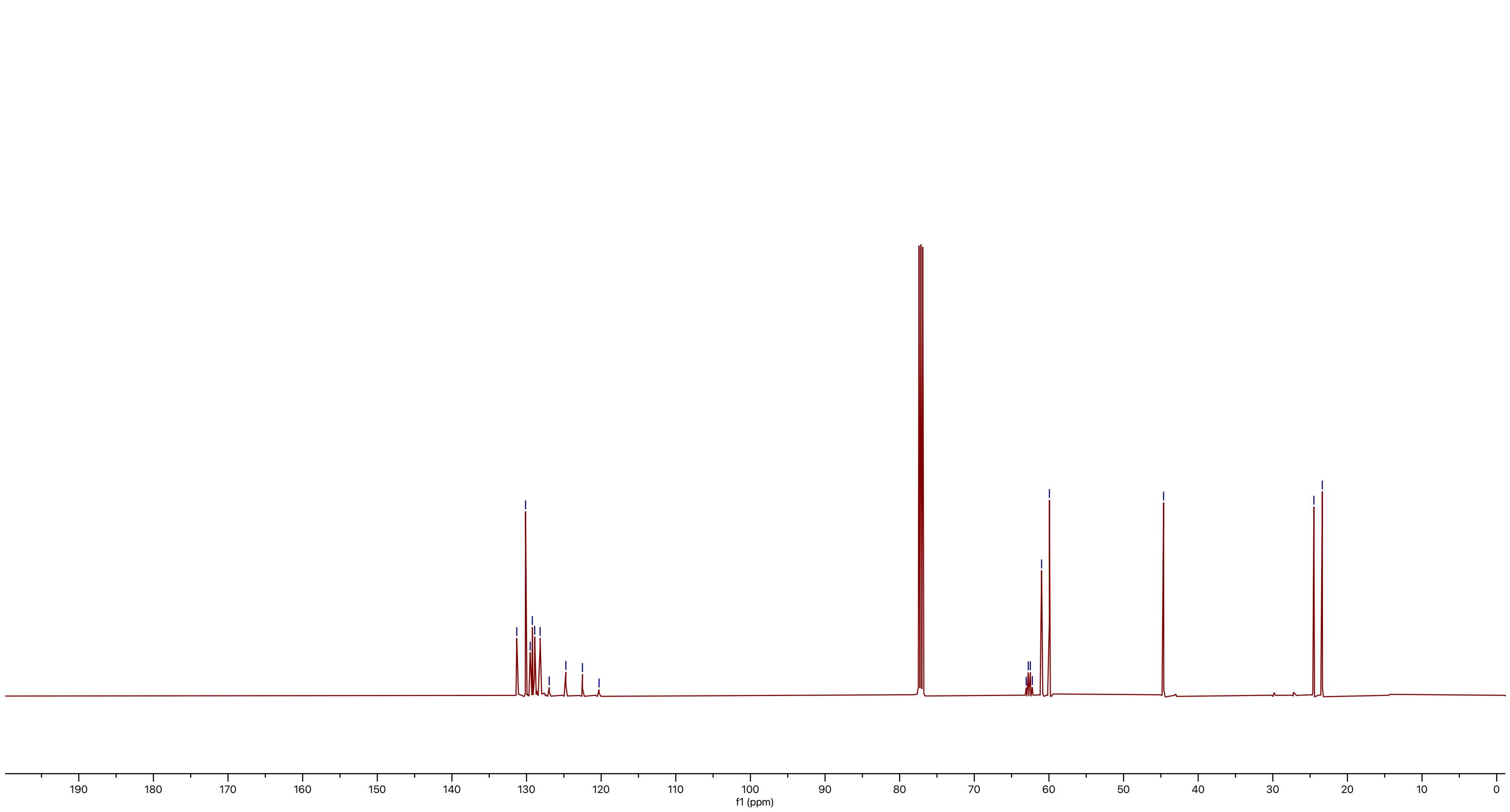

^{19}F NMR of 4a ($CDCl_3$, 471 MHz)

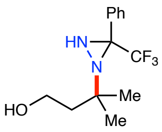

4a

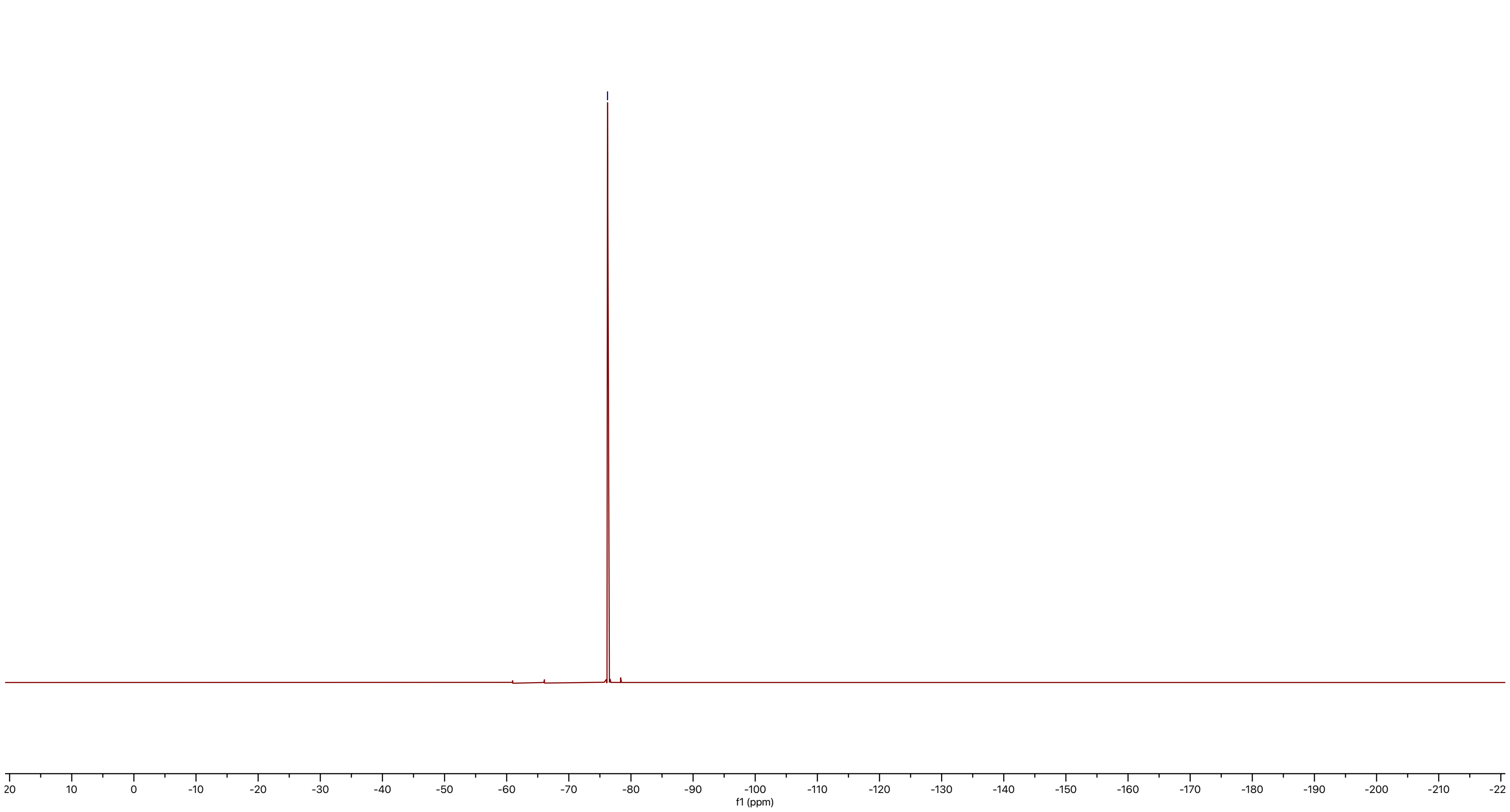

^{1}H NMR of 4b ($CDCl_3$, 500 MHz)

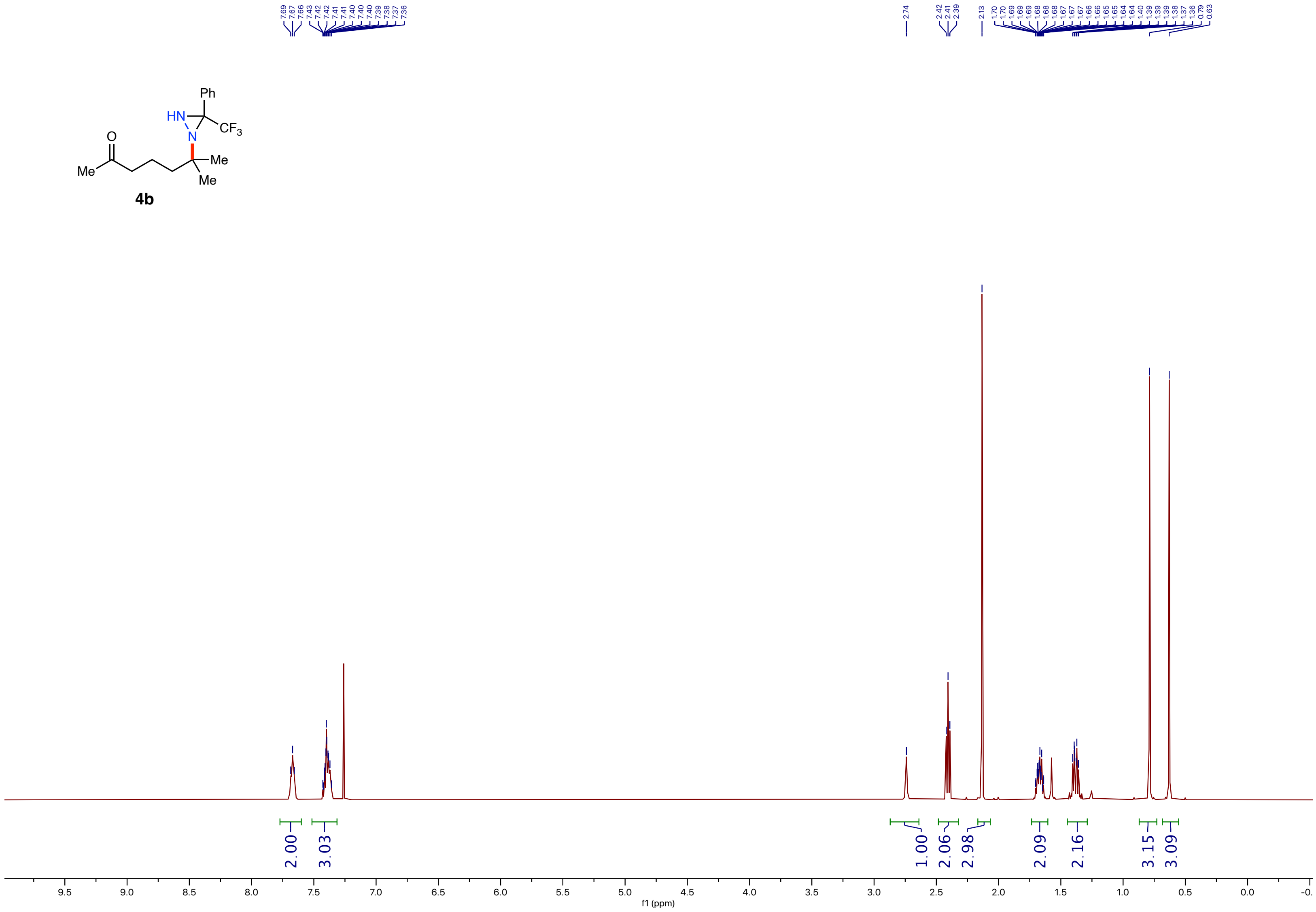

^{13}C NMR of 4b ($CDCl_3$, 126 MHz)

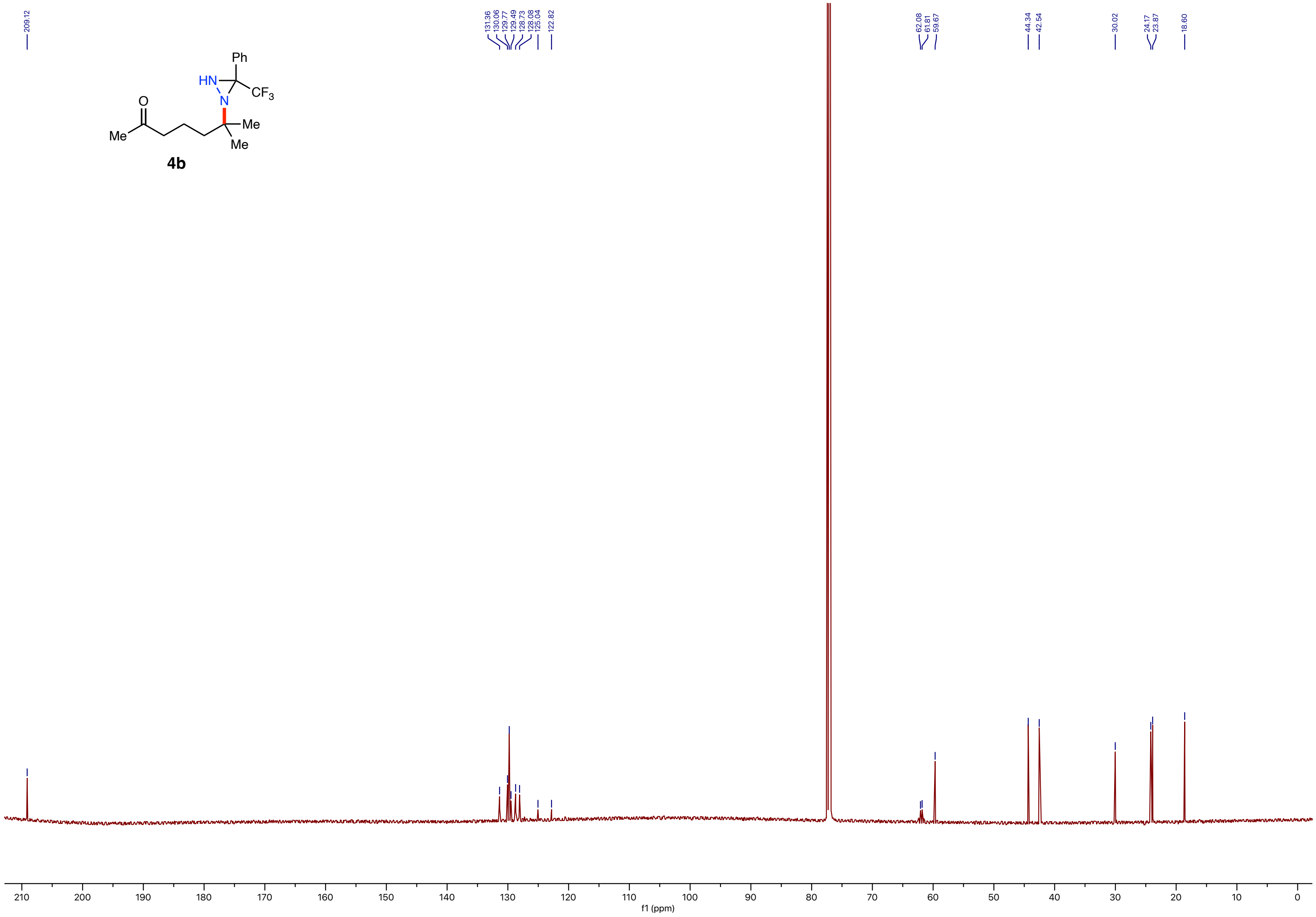

^{19}F NMR of 4b ($CDCl_3$, 471 MHz)

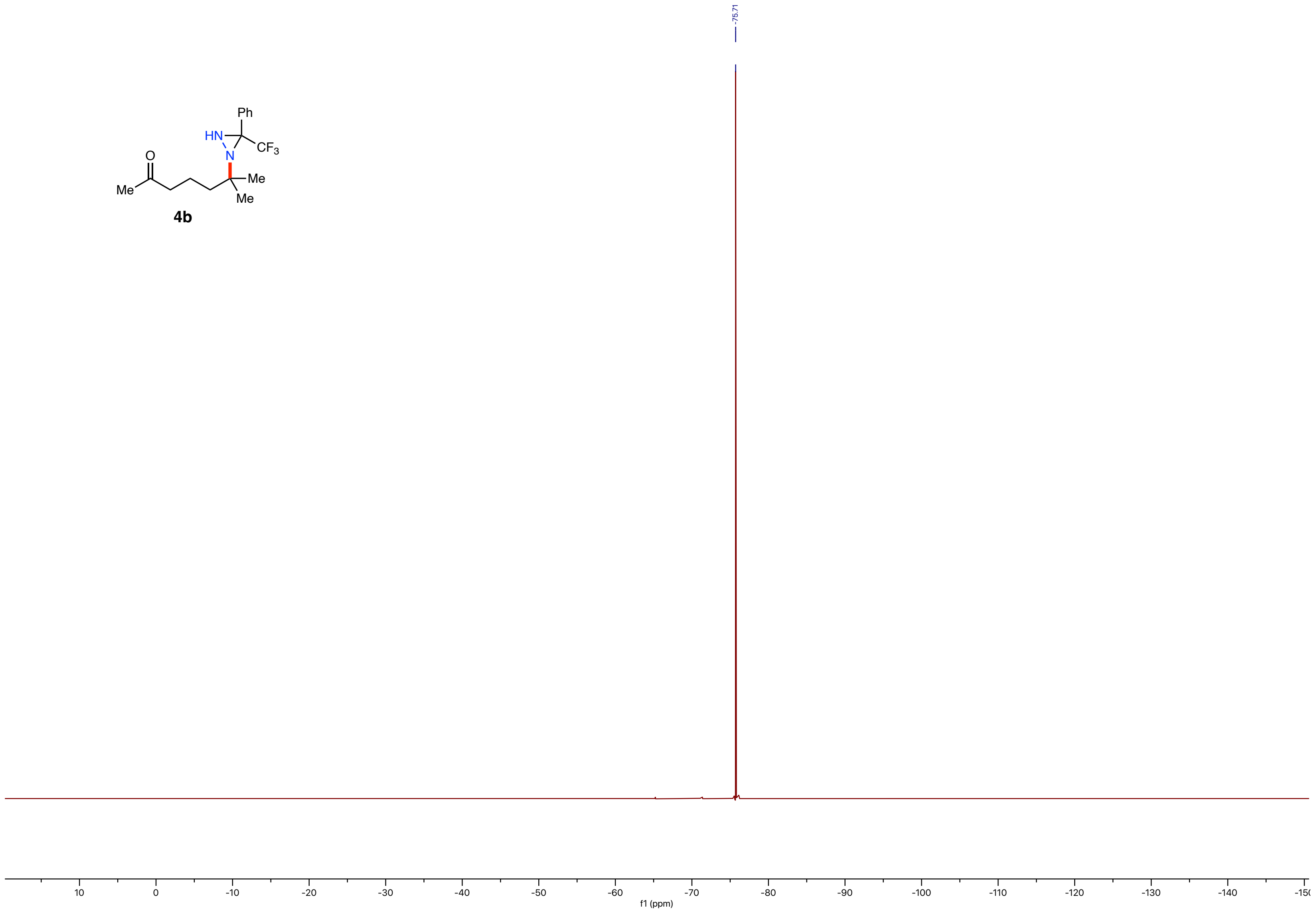

^1H NMR of 4c ($CDCl_3$, 500 MHz)

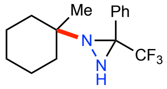

4c

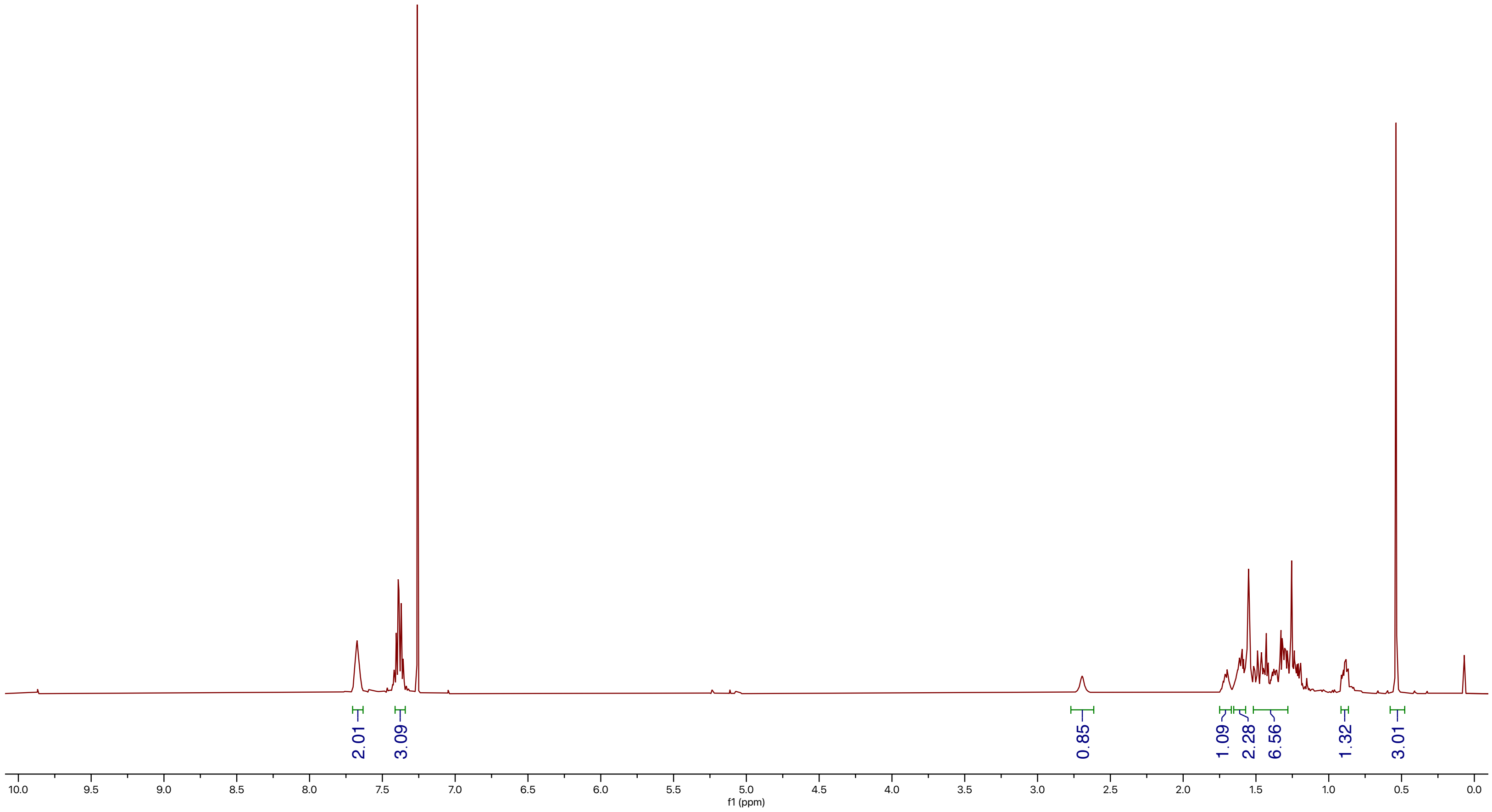

^{13}C NMR of 4c (CDCl$_3$, 126 MHz)

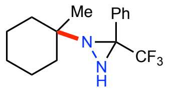

4c

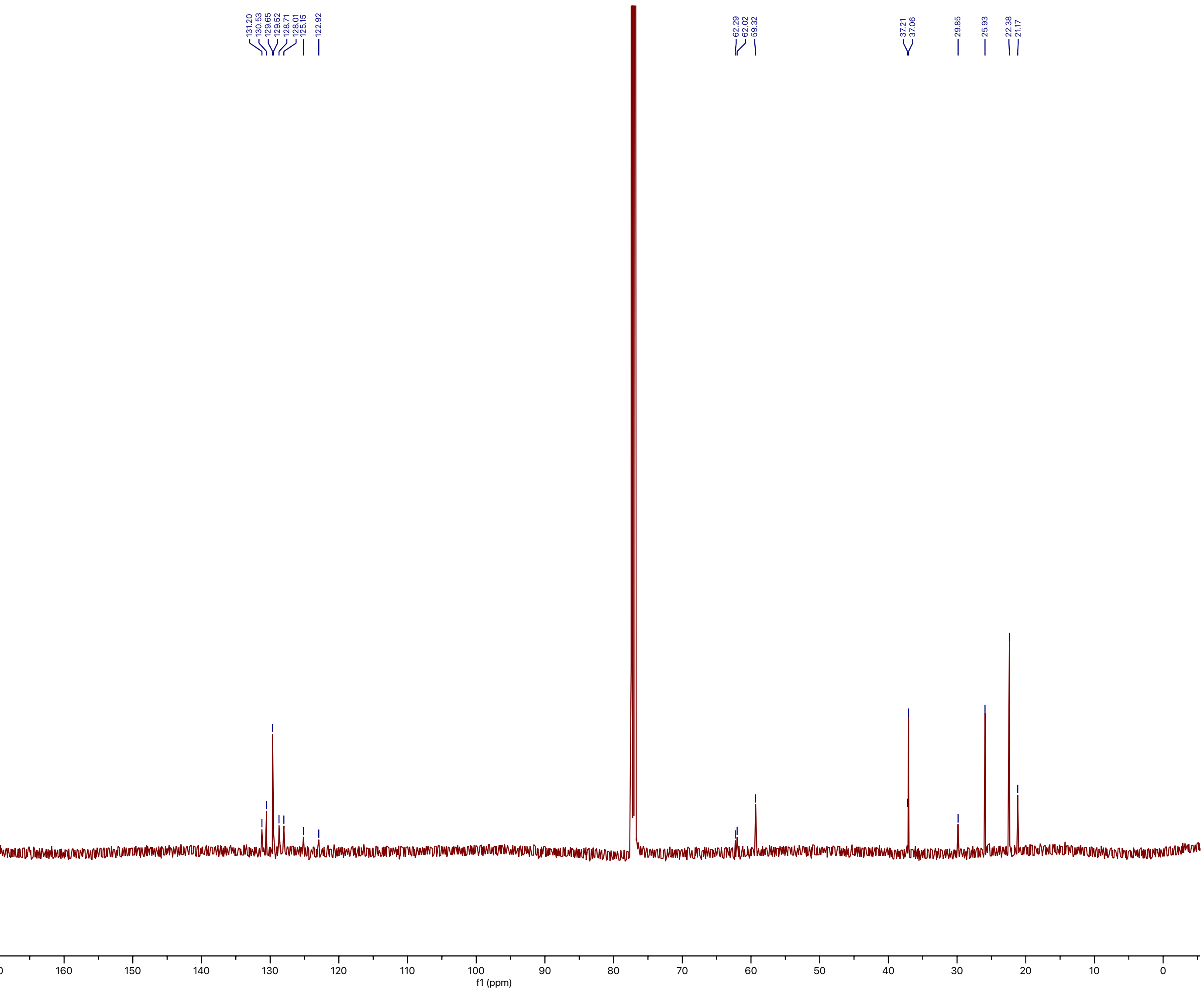

^{19}F NMR of 4c ($CDCl_3$, 471 MHz)

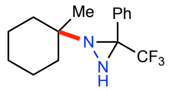

4c

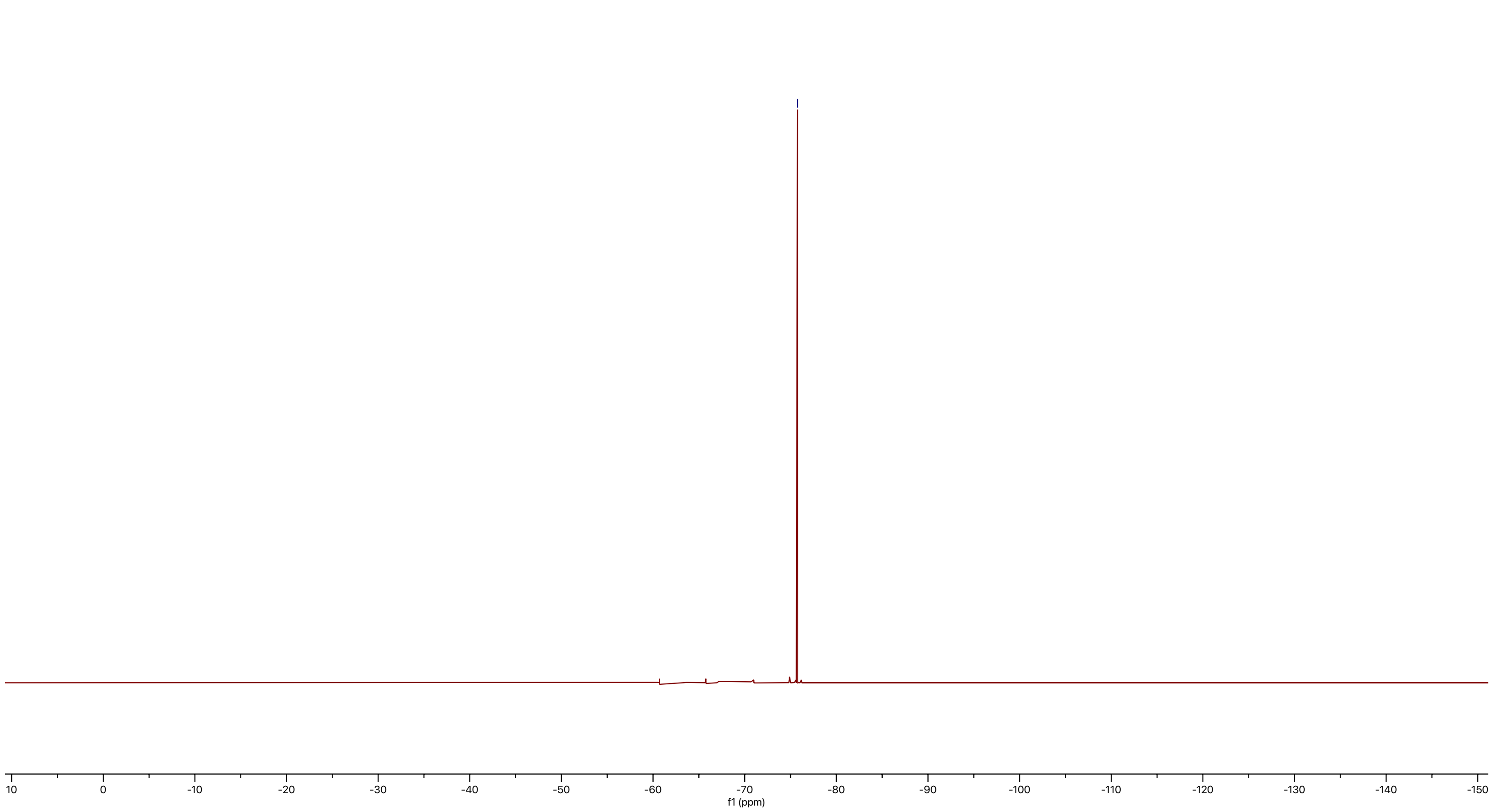

^{1}H NMR of 4d ($CDCl_3$, 500 MHz)

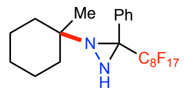

4d

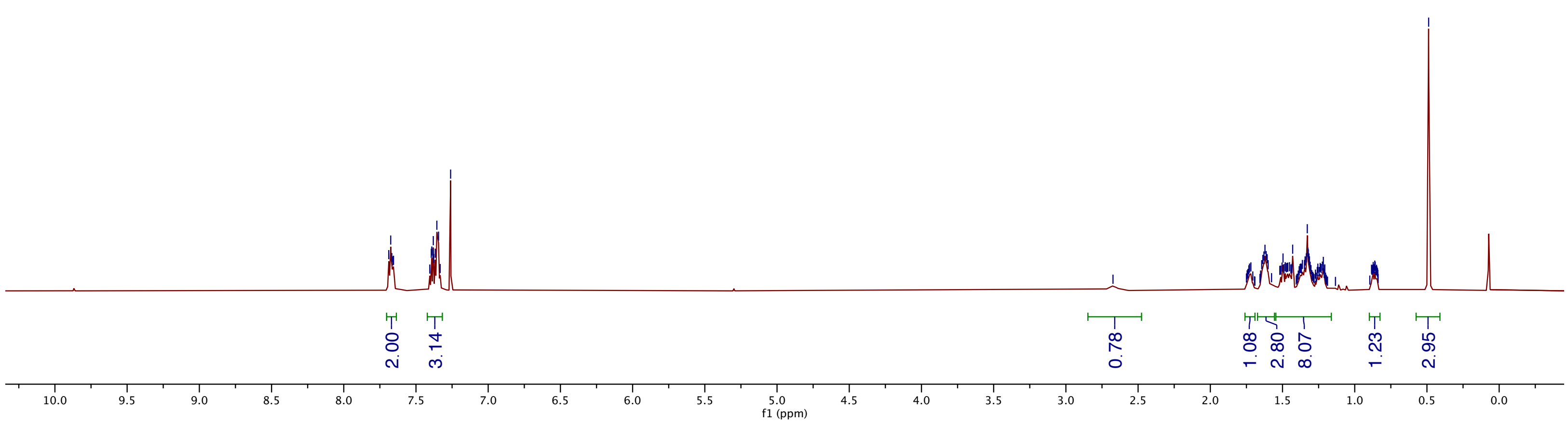

^{13}C NMR of 4d ($CDCl_3$, 126 MHz)

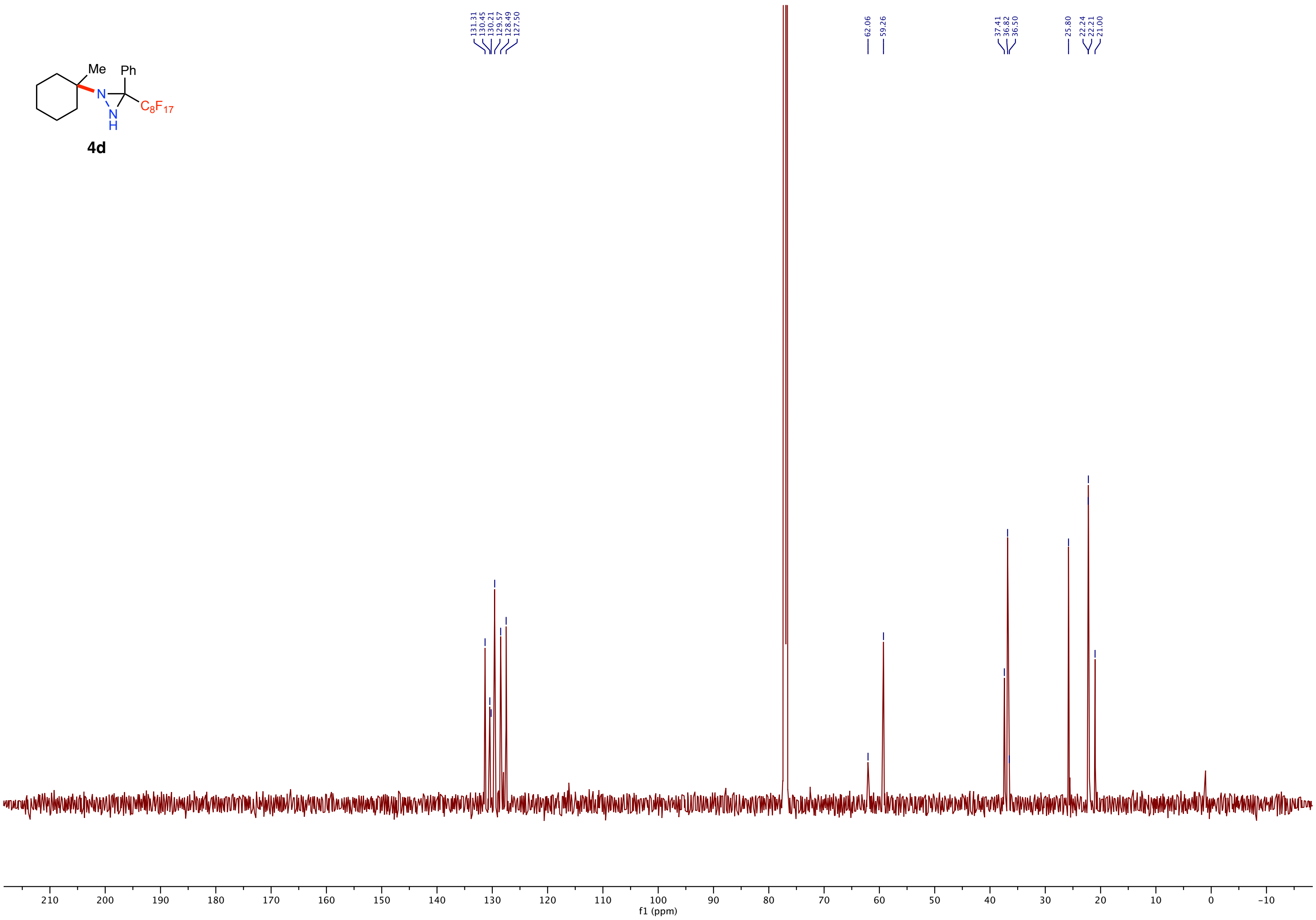

^{19}F NMR of 4d ($CDCl_3$, 471 MHz)

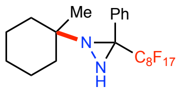

4d

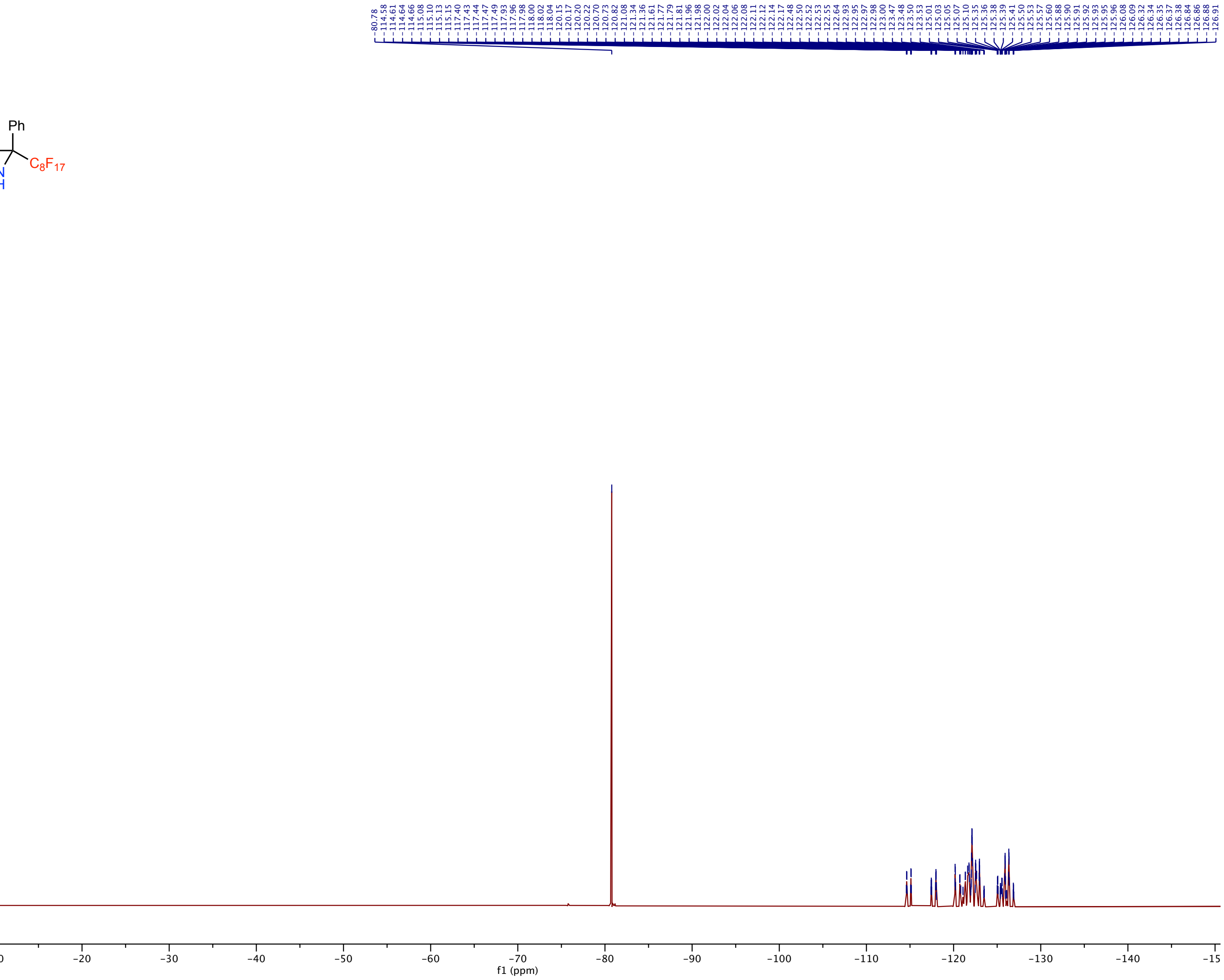

^{1}H NMR of 4e ($CDCl_3$, 500 MHz)

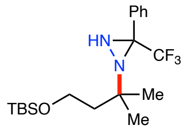

4e

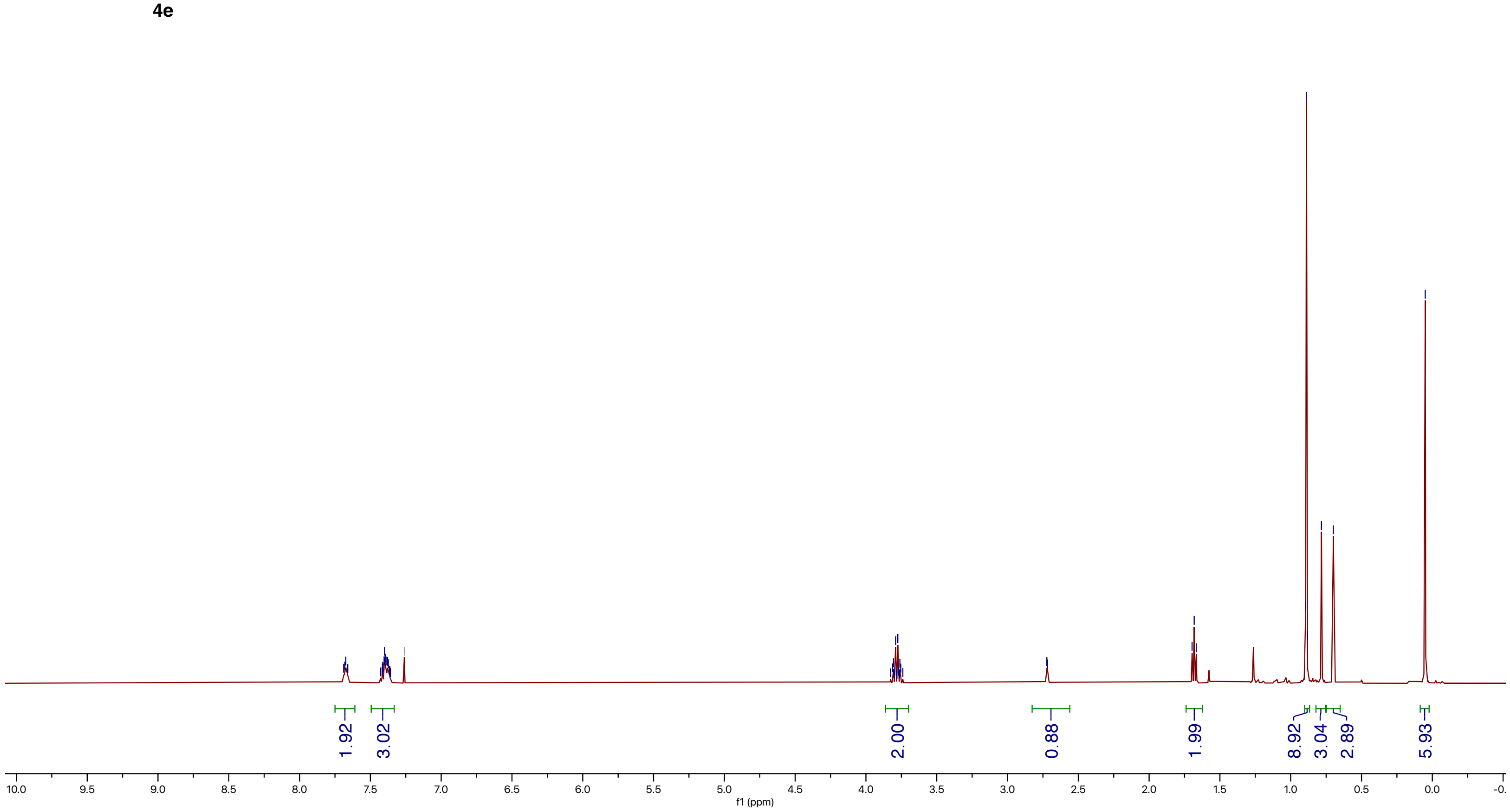

^{13}C NMR of 4e ($CDCl_3$, 471 MHz)

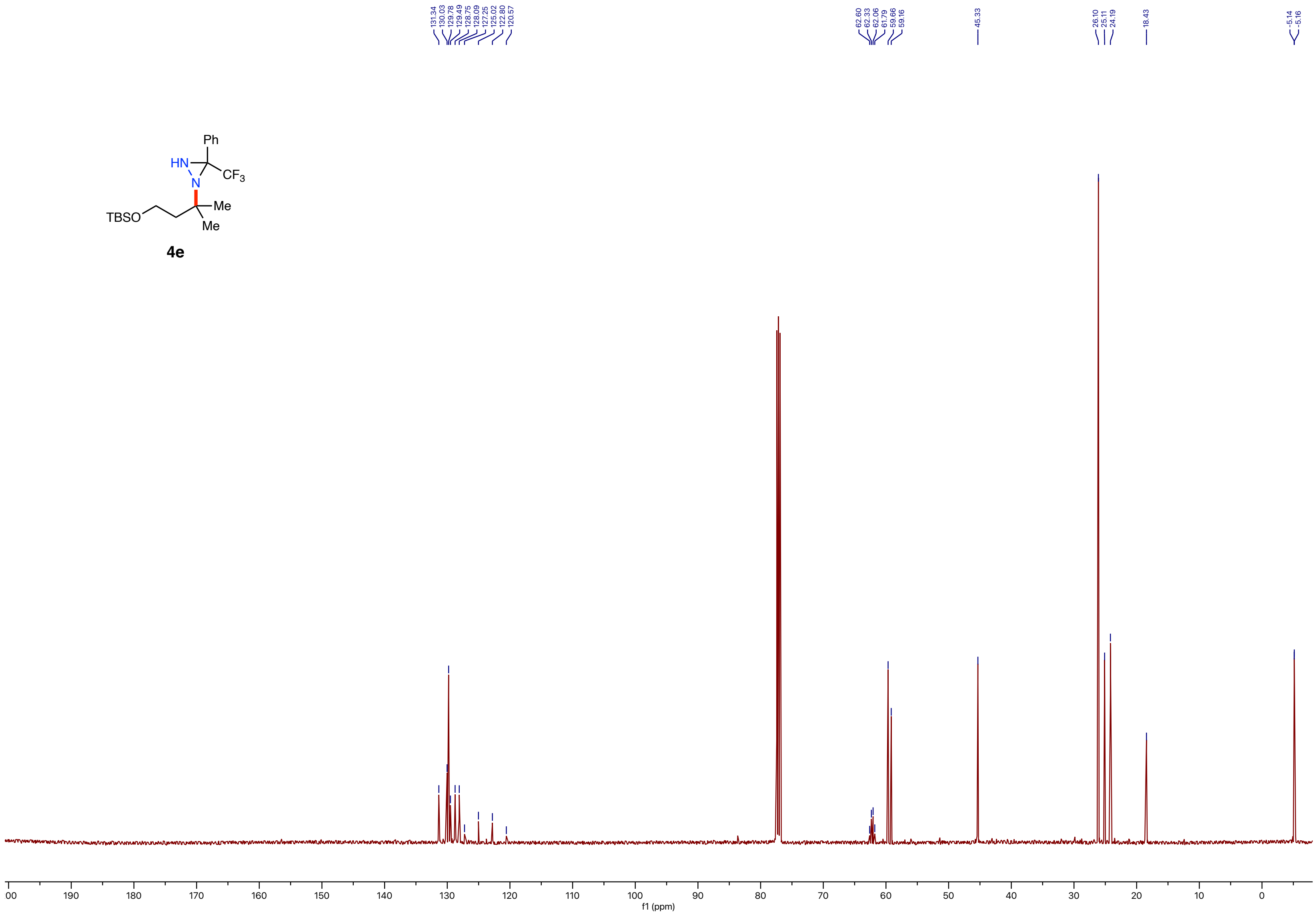

^{19}F NMR of 4e ($CDCl_3$, 126 MHz)

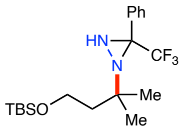

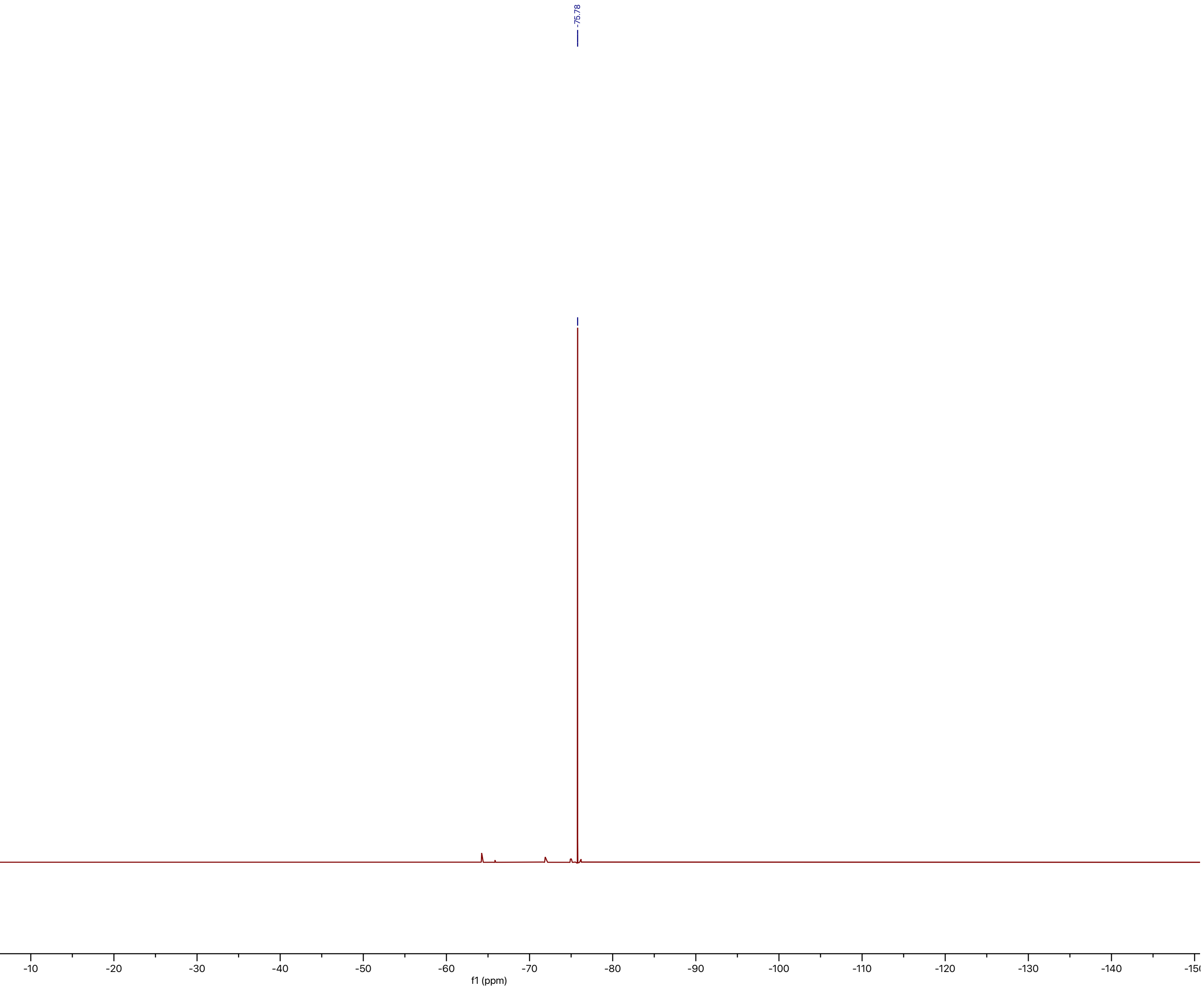

^{1}H NMR of 4f ($CDCl_3$, 500 MHz)

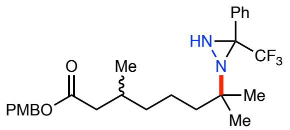

4f

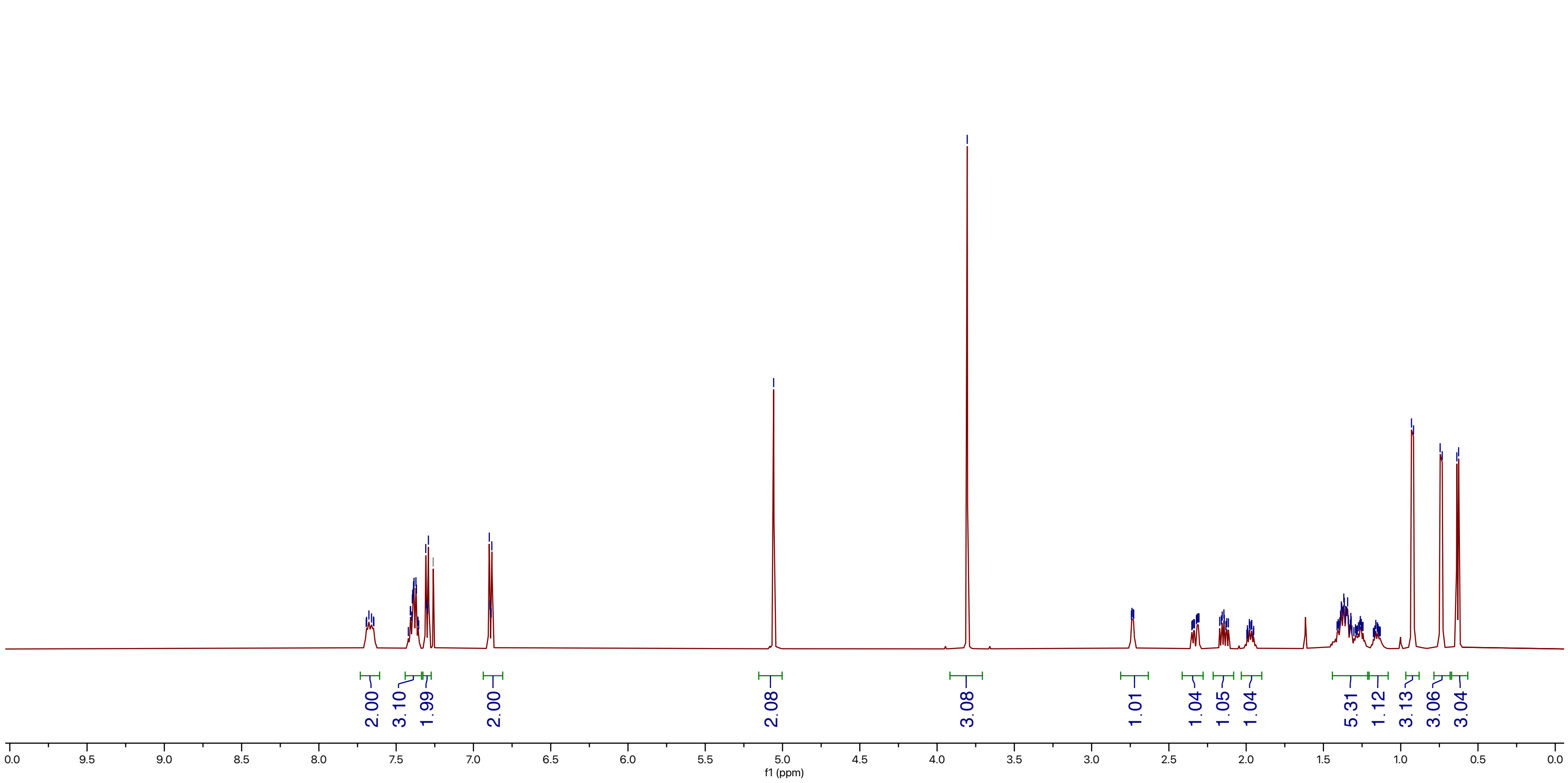

^{13}C NMR of 4f ($CDCl_3$, 126 MHz)

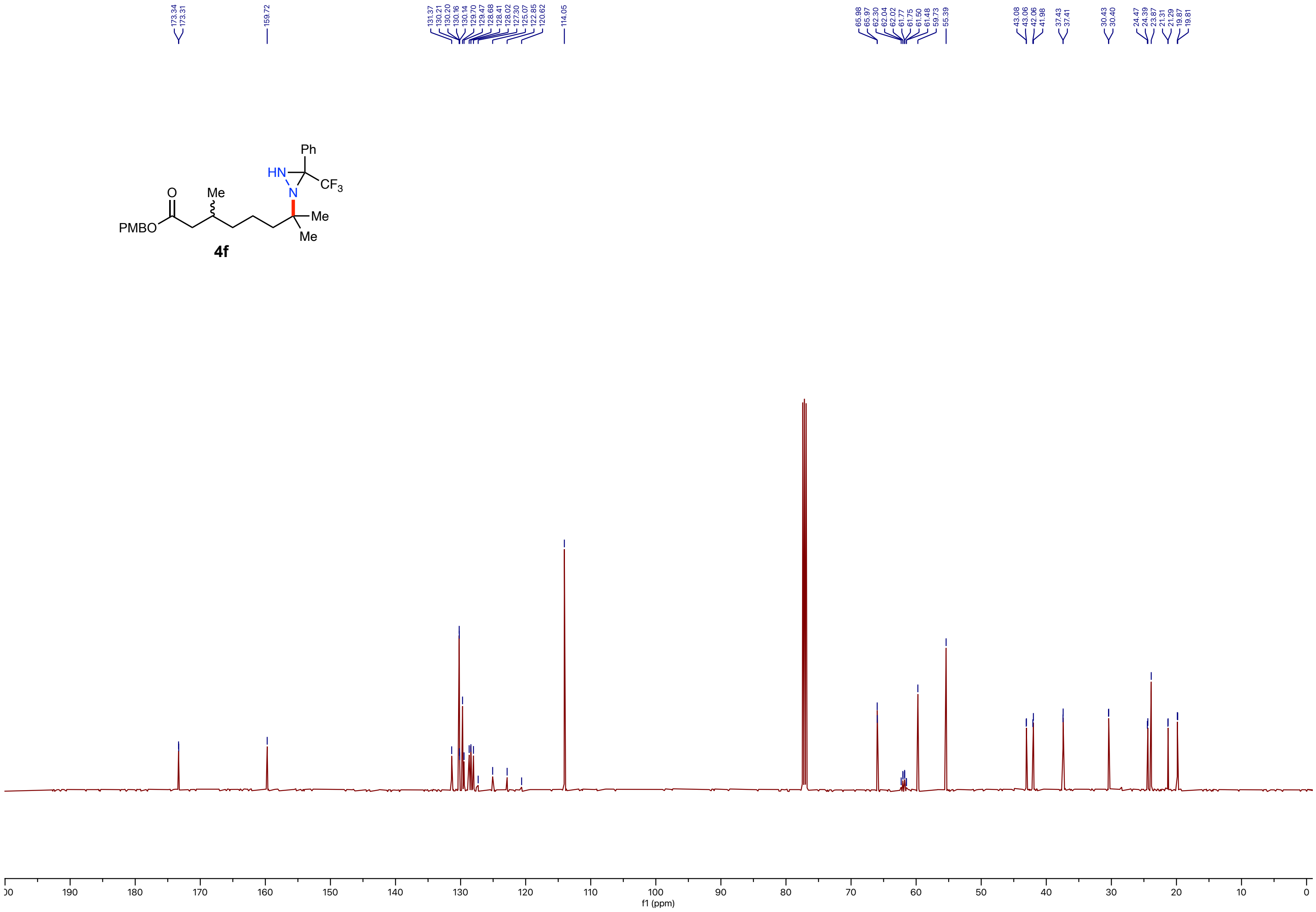

^{19}F NMR of 4f ($CDCl_3$, 471 MHz)

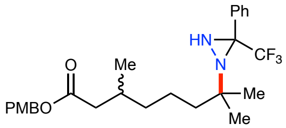

4f

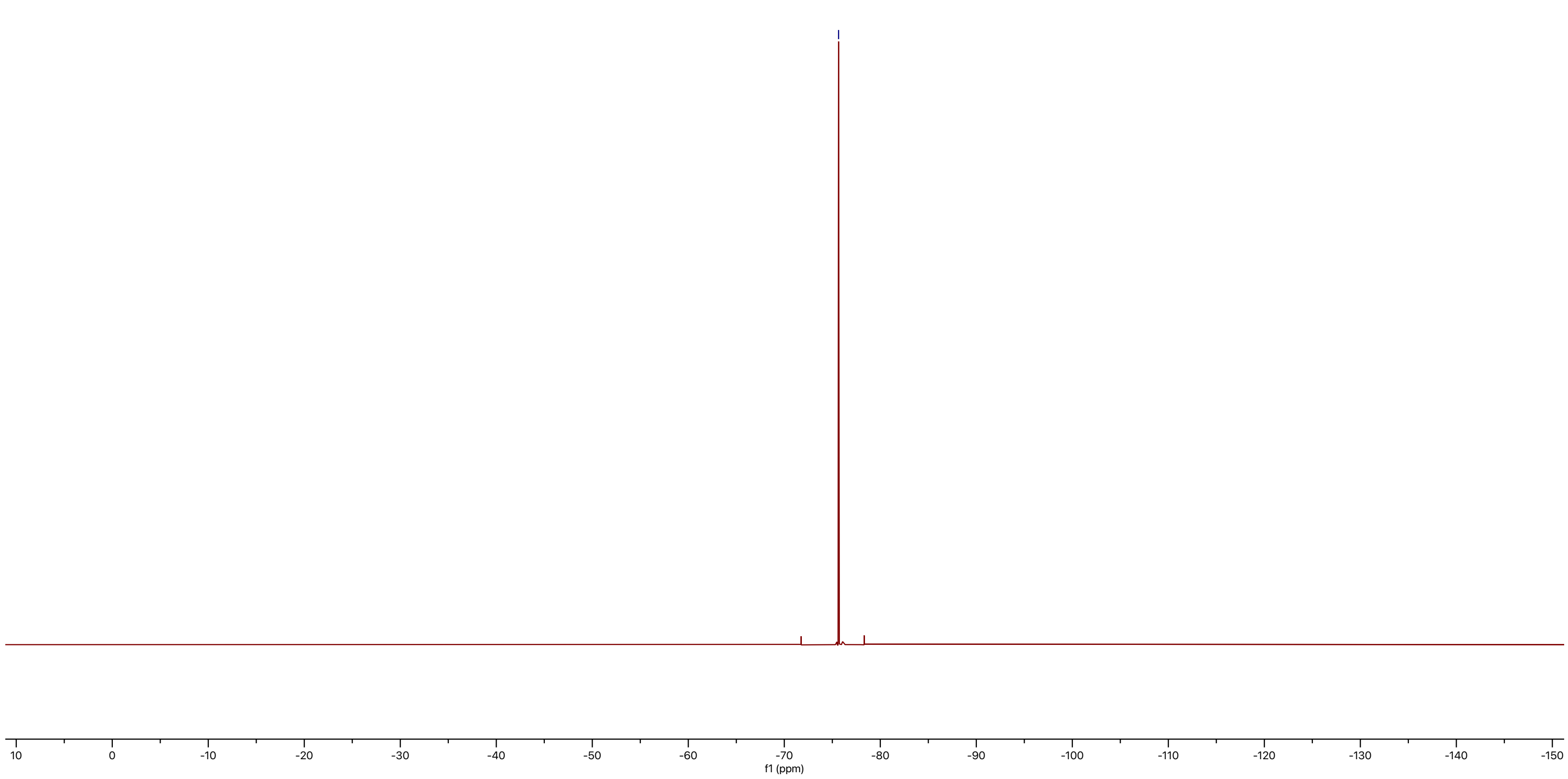

^{1}H NMR of 4g ($CDCl_3$, 500 MHz)

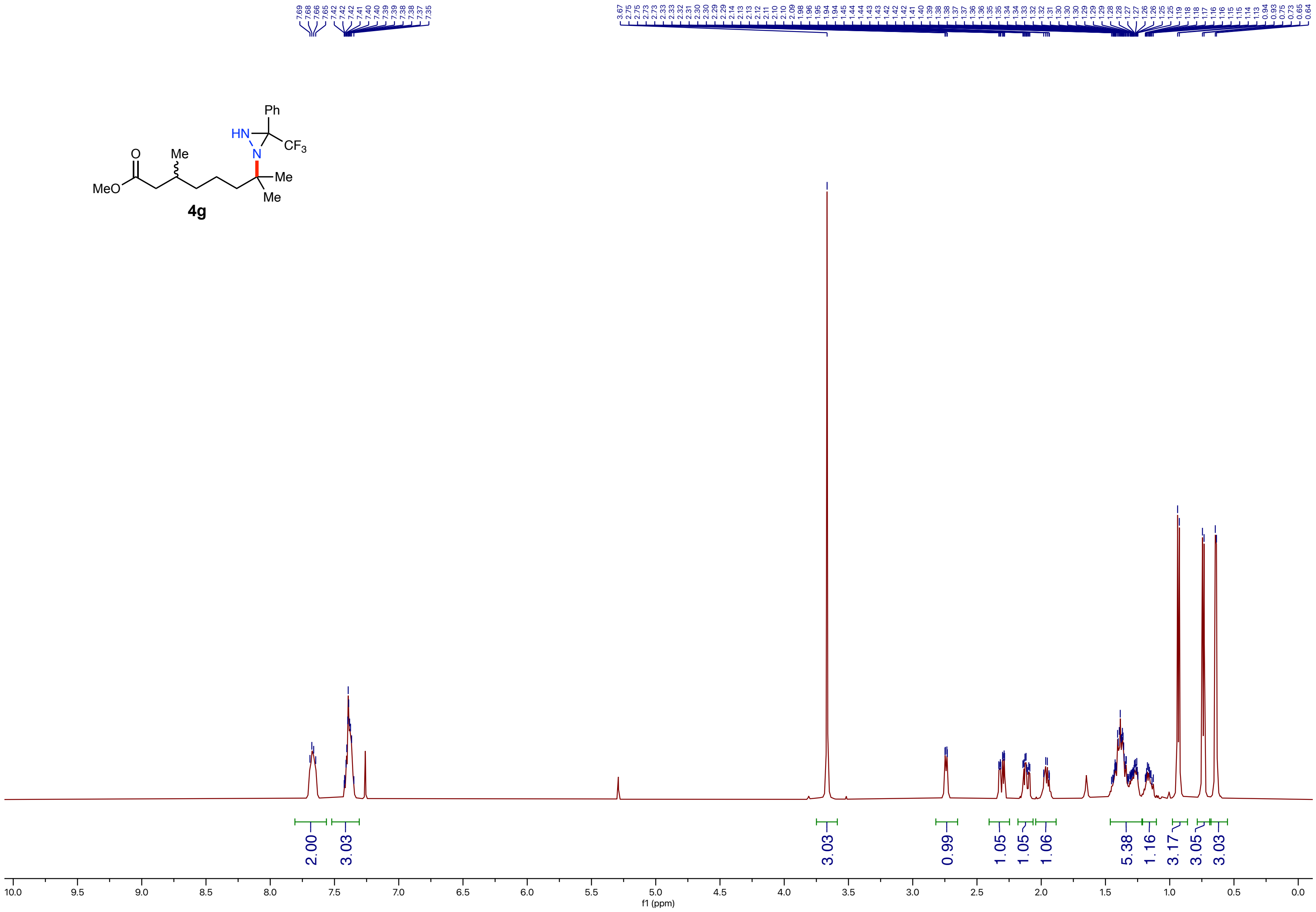

^{13}C NMR of 4g (CDCl$_3$, 126 MHz)

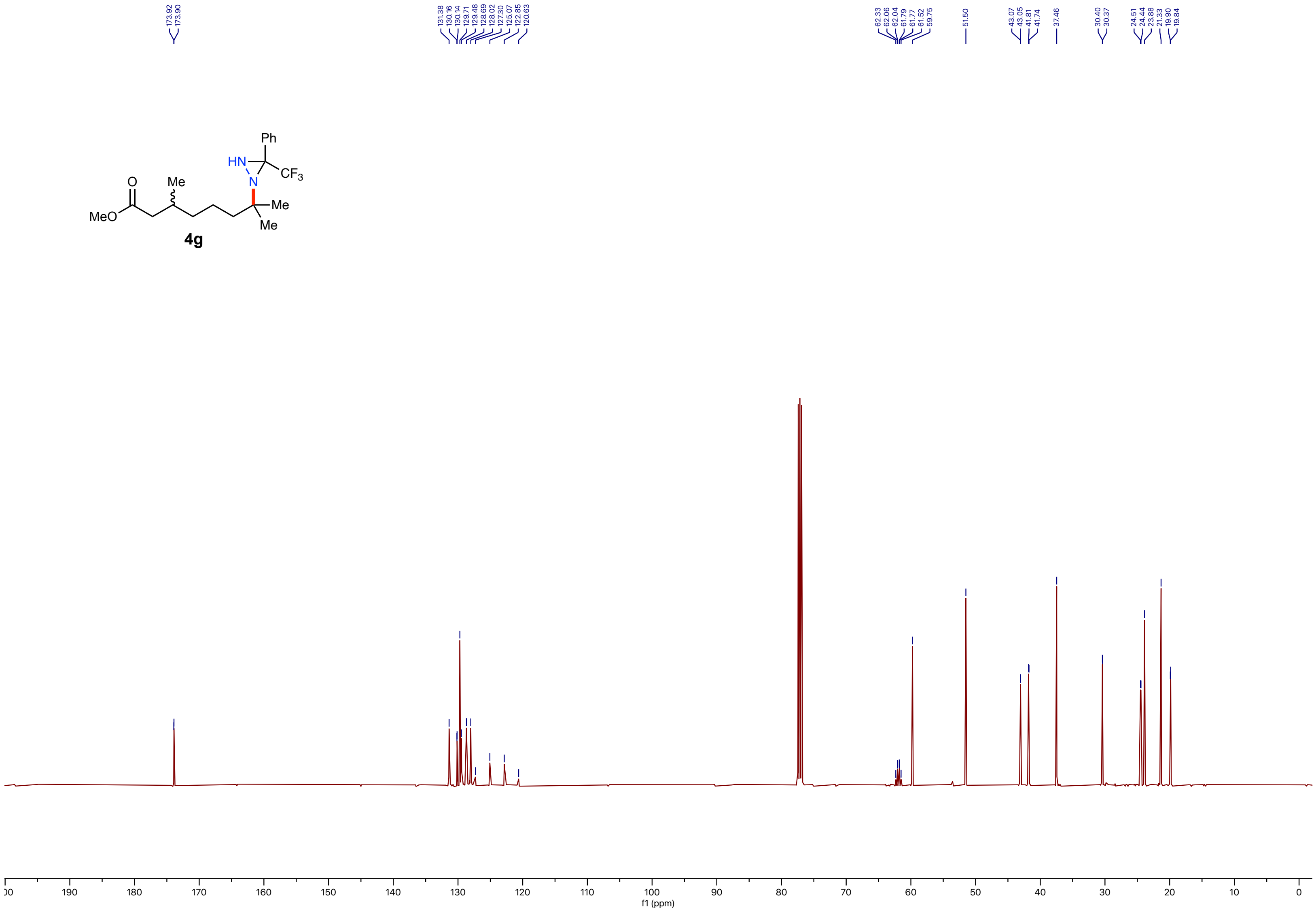

^{19}F NMR of 4g ($CDCl_3$, 471 MHz)

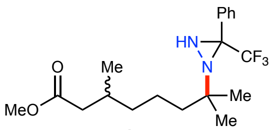

4g

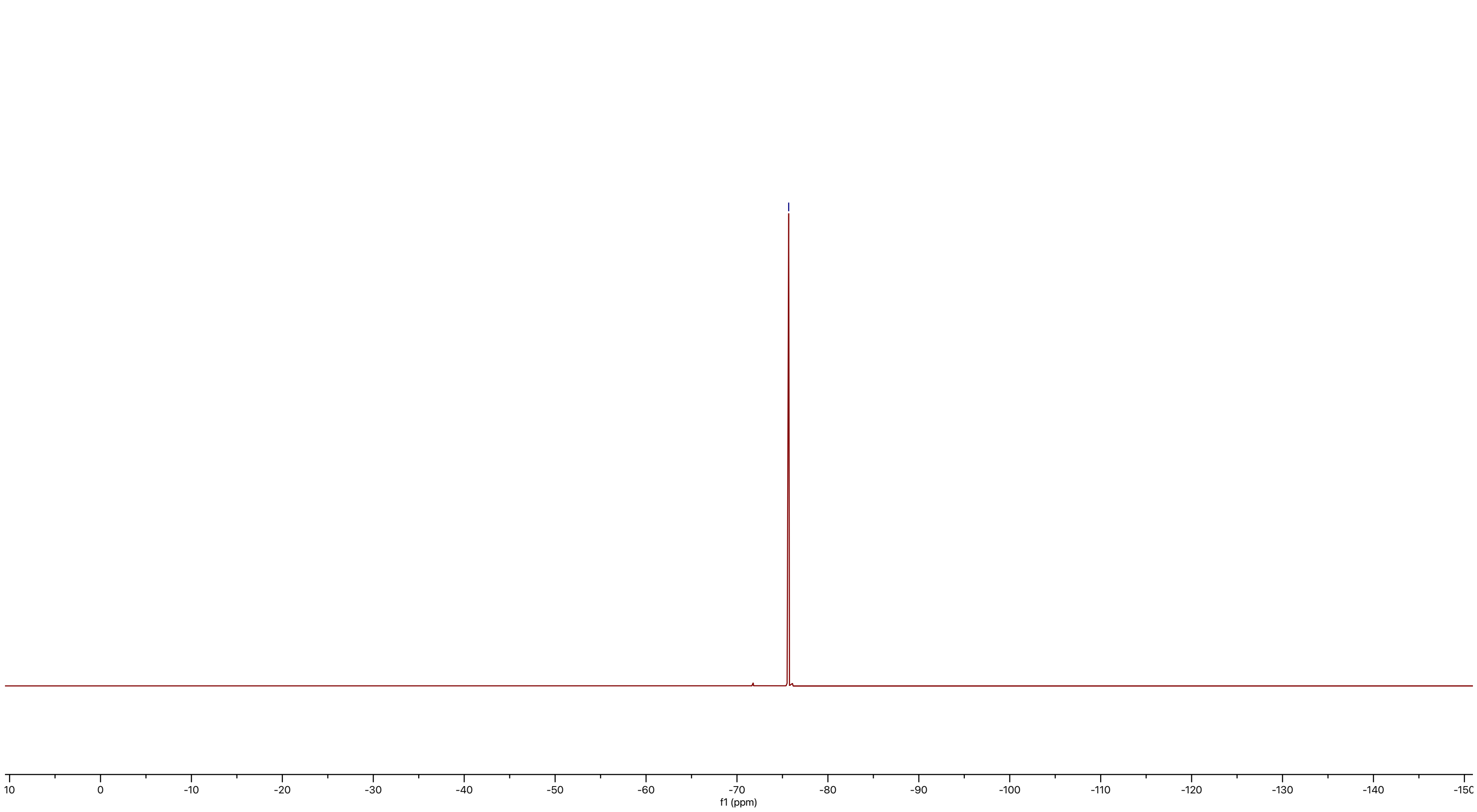

^{1}H NMR of 5a ($CDCl_3$, 500 MHz)

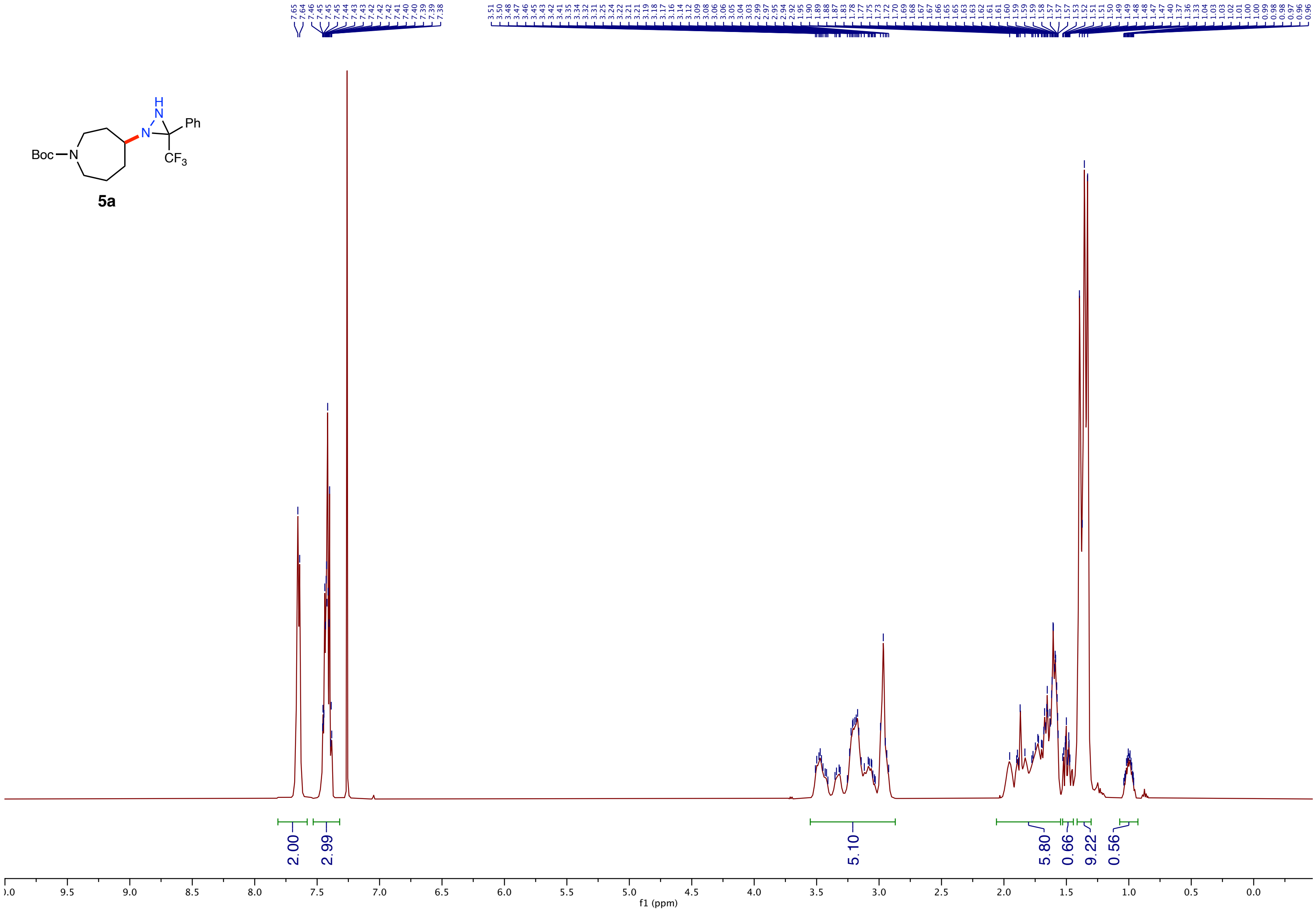

^{13}C NMR of 5a ($CDCl_3$, 126 MHz)

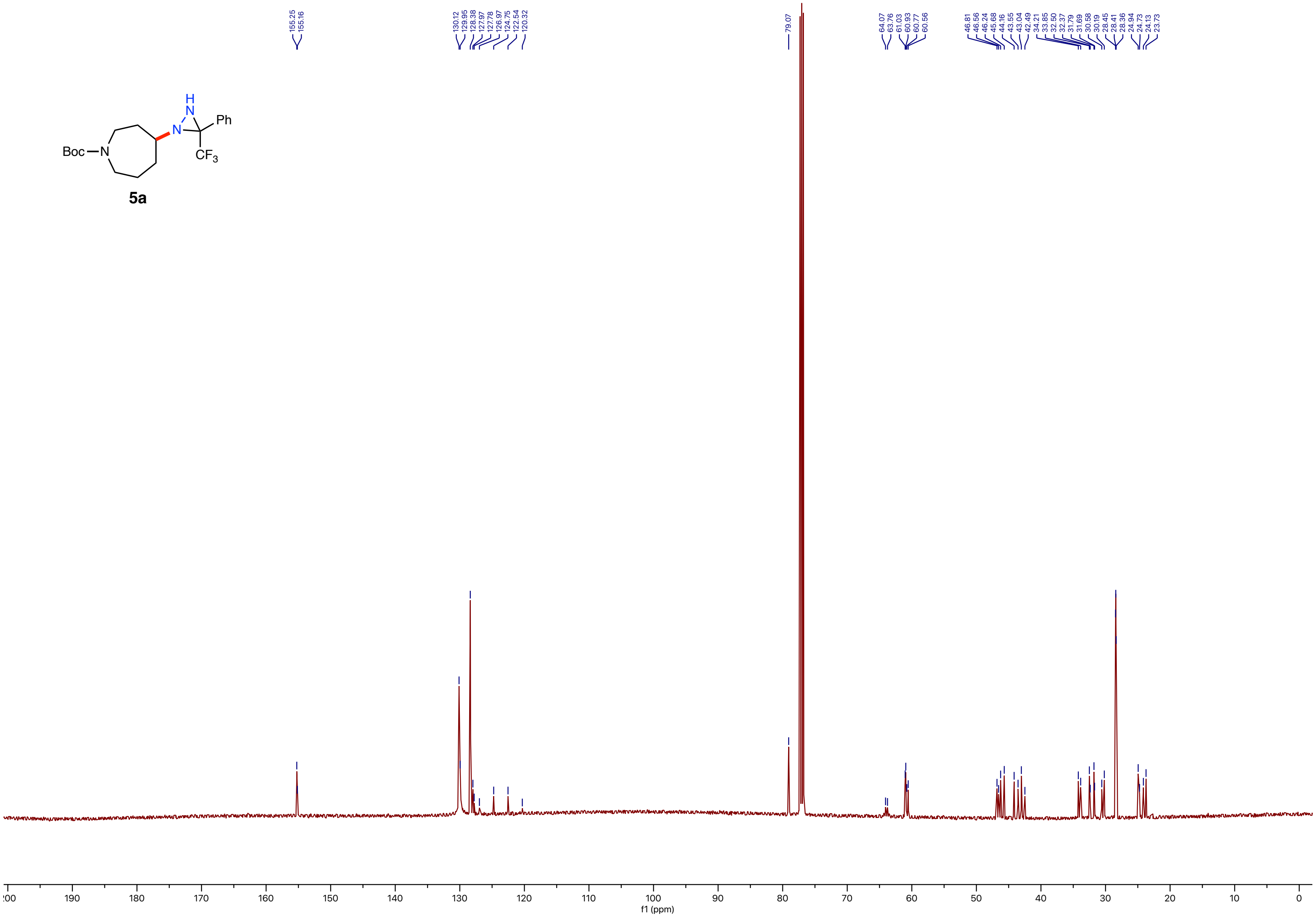

^{19}F NMR of 5a ($CDCl_3$, 471 MHz)

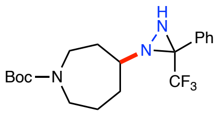

5a

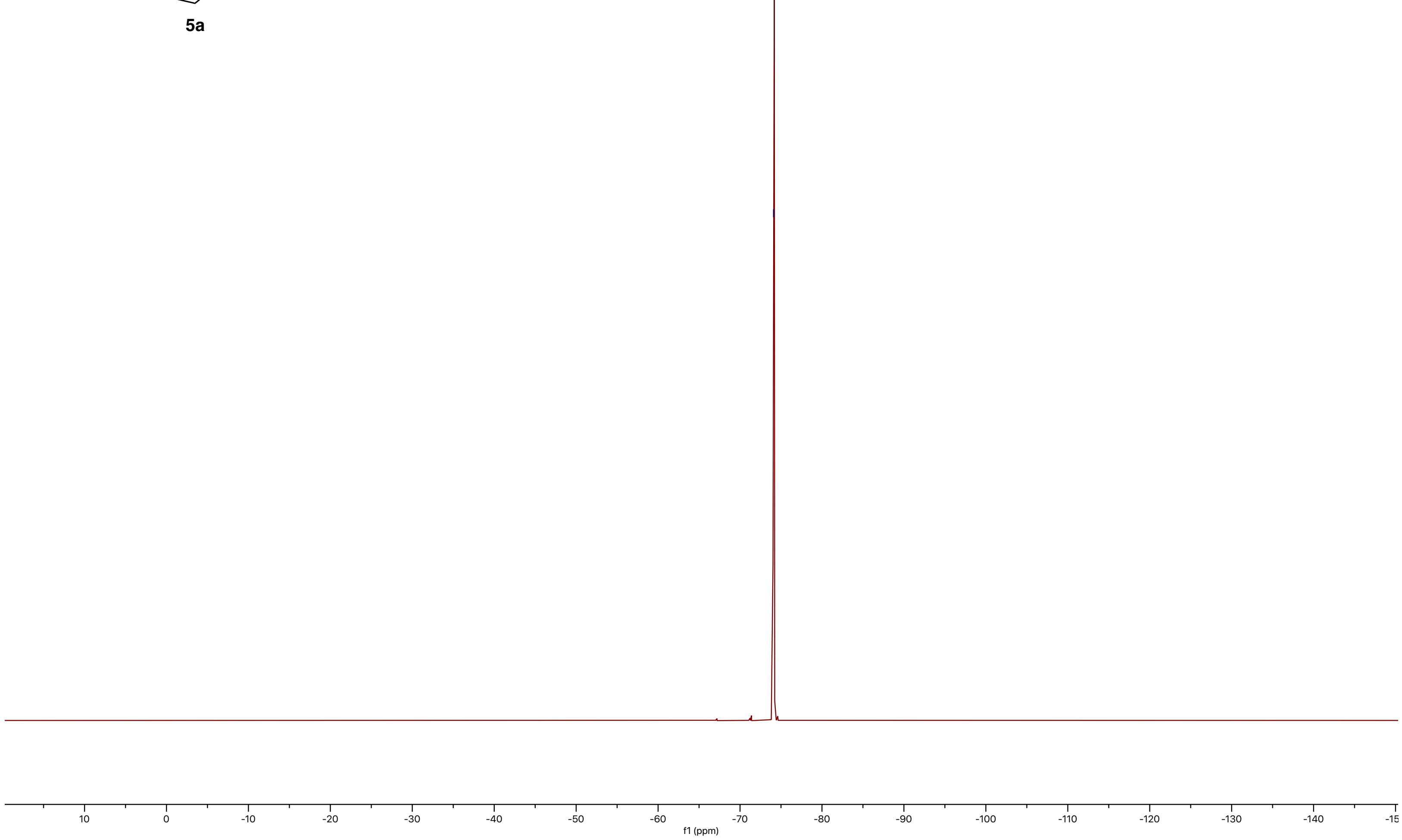

^{1}H NMR of 5c ($CDCl_3$, 500 MHz)

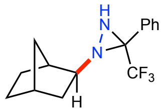

5c

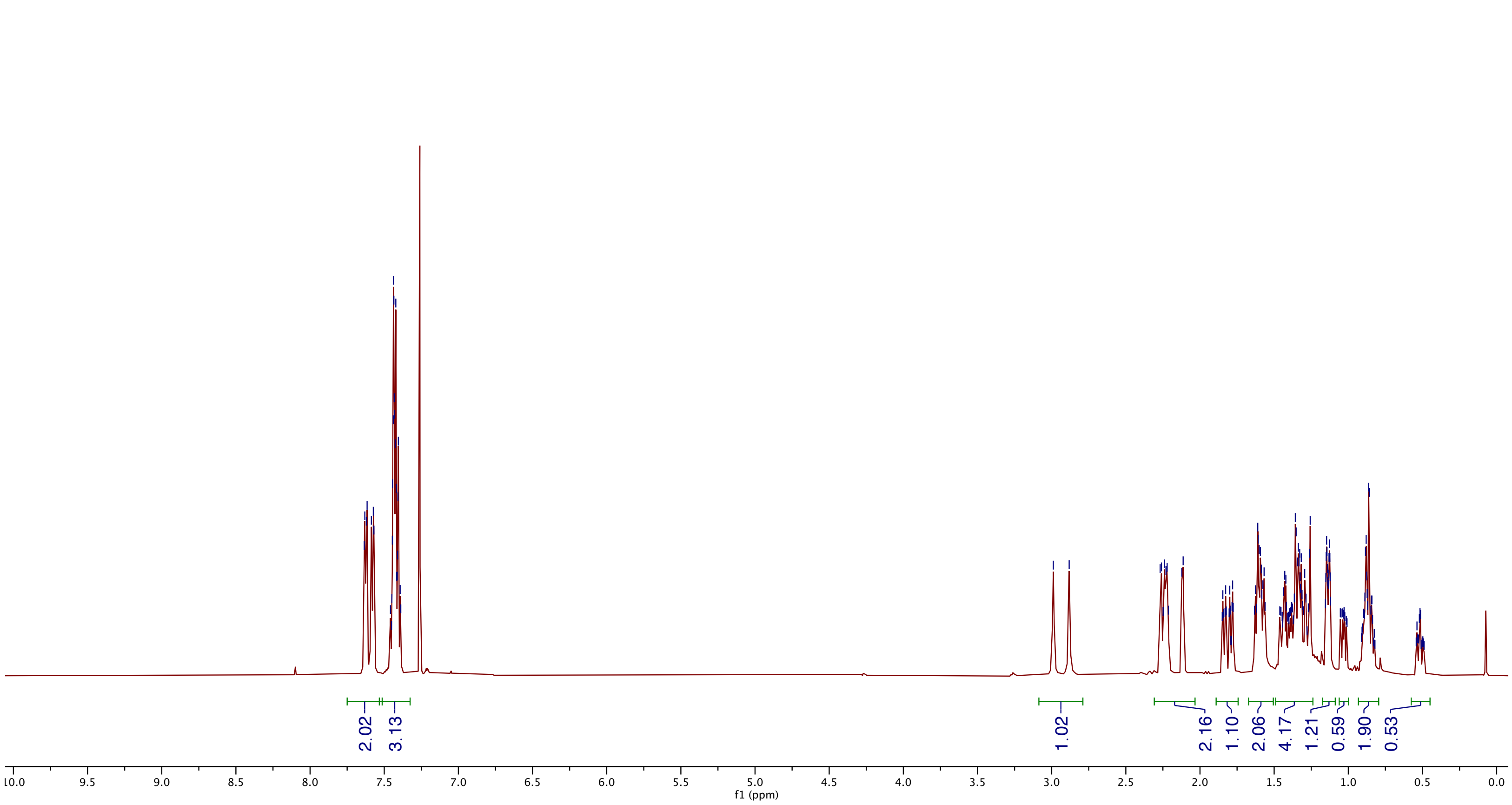

^{13}C NMR of 5c ($CDCl_3$, 126 MHz)

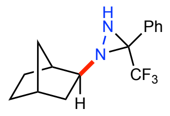

5c

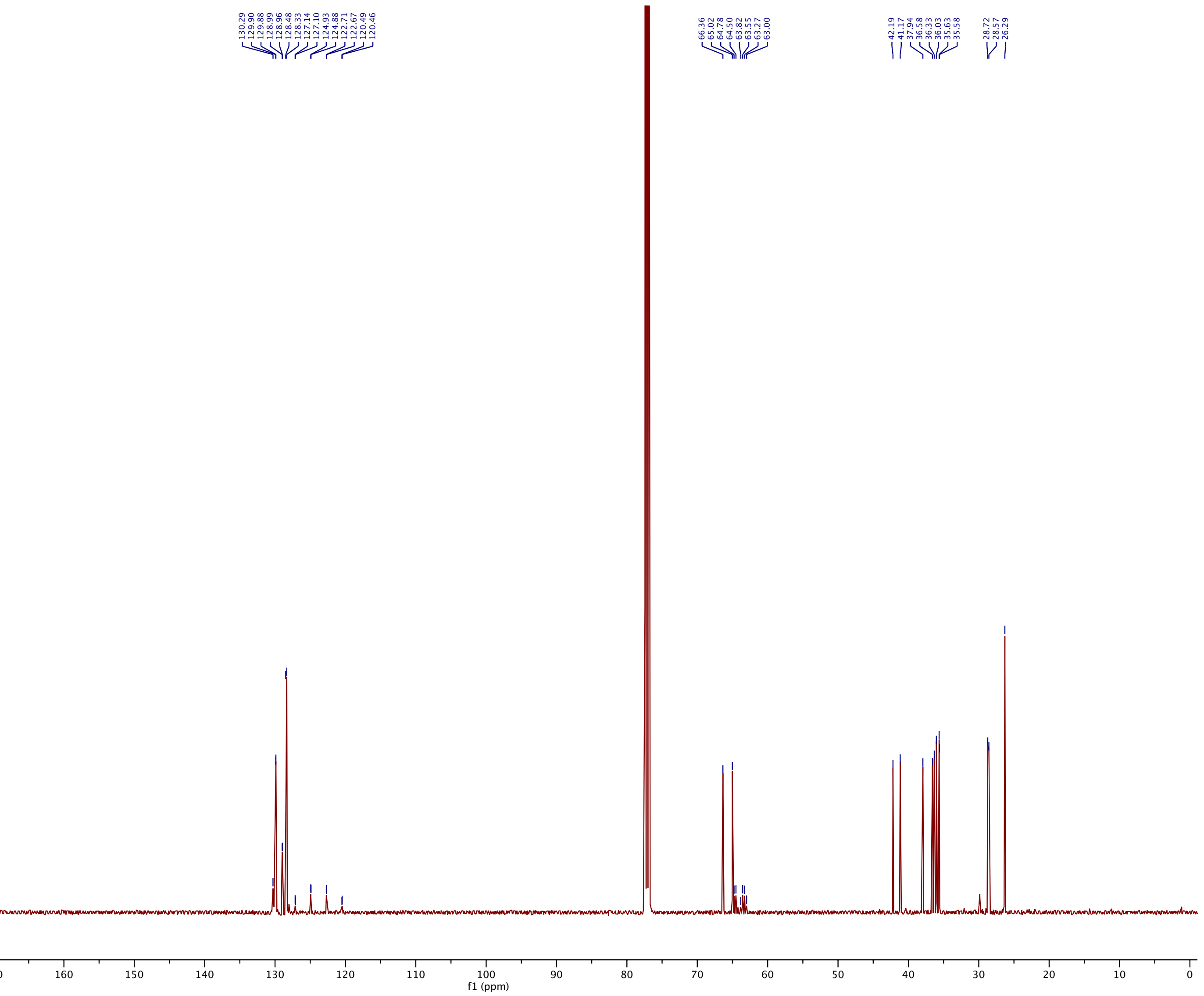

^{19}F NMR of 5c ($CDCl_3$, 471 MHz)

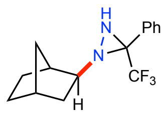

5c

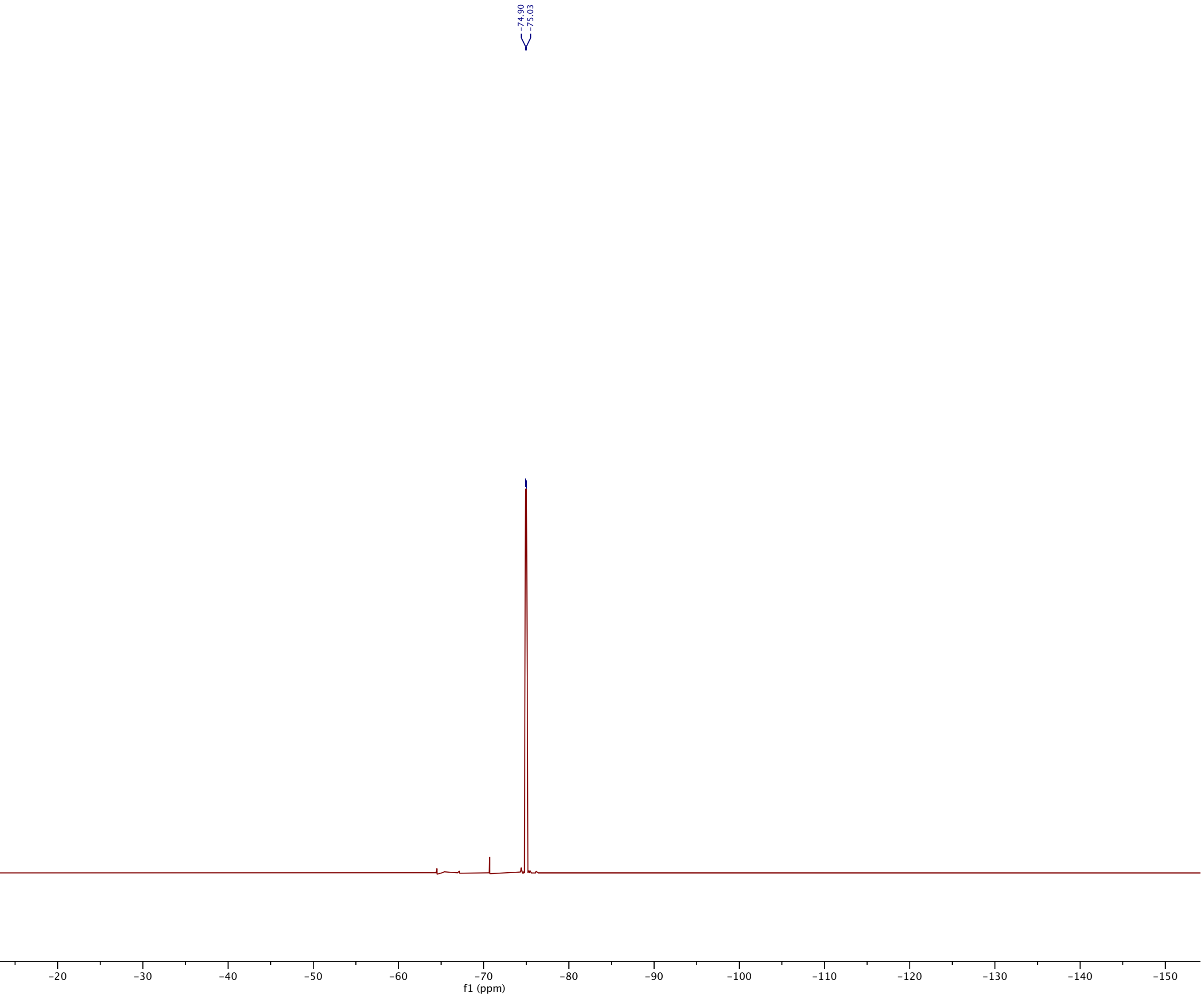

^{1}H NMR of 5e ($CDCl_3$, 500 MHz)

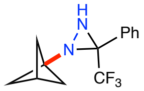

5e

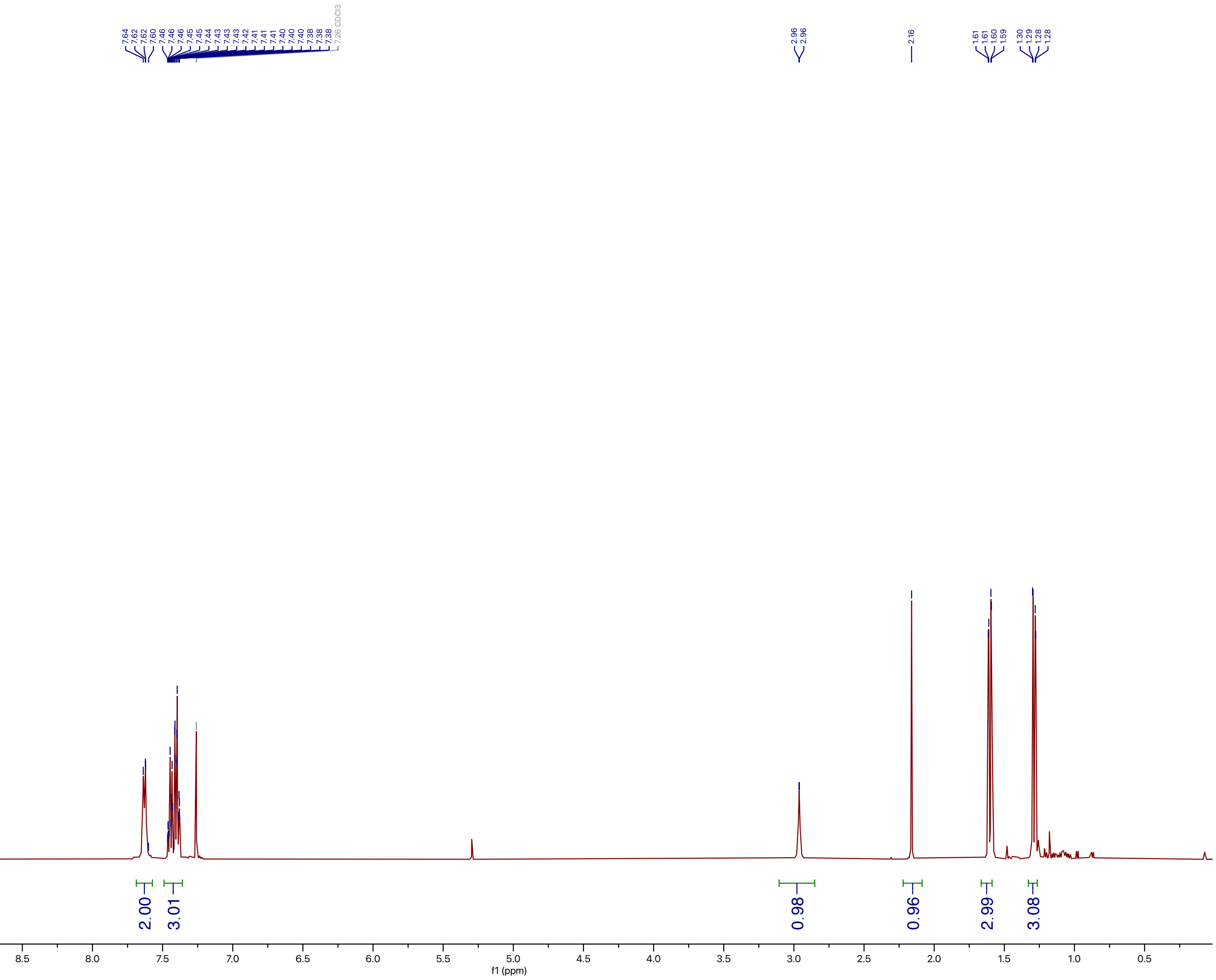

^{13}C NMR of 5e (CDCl$_3$, 126 MHz)

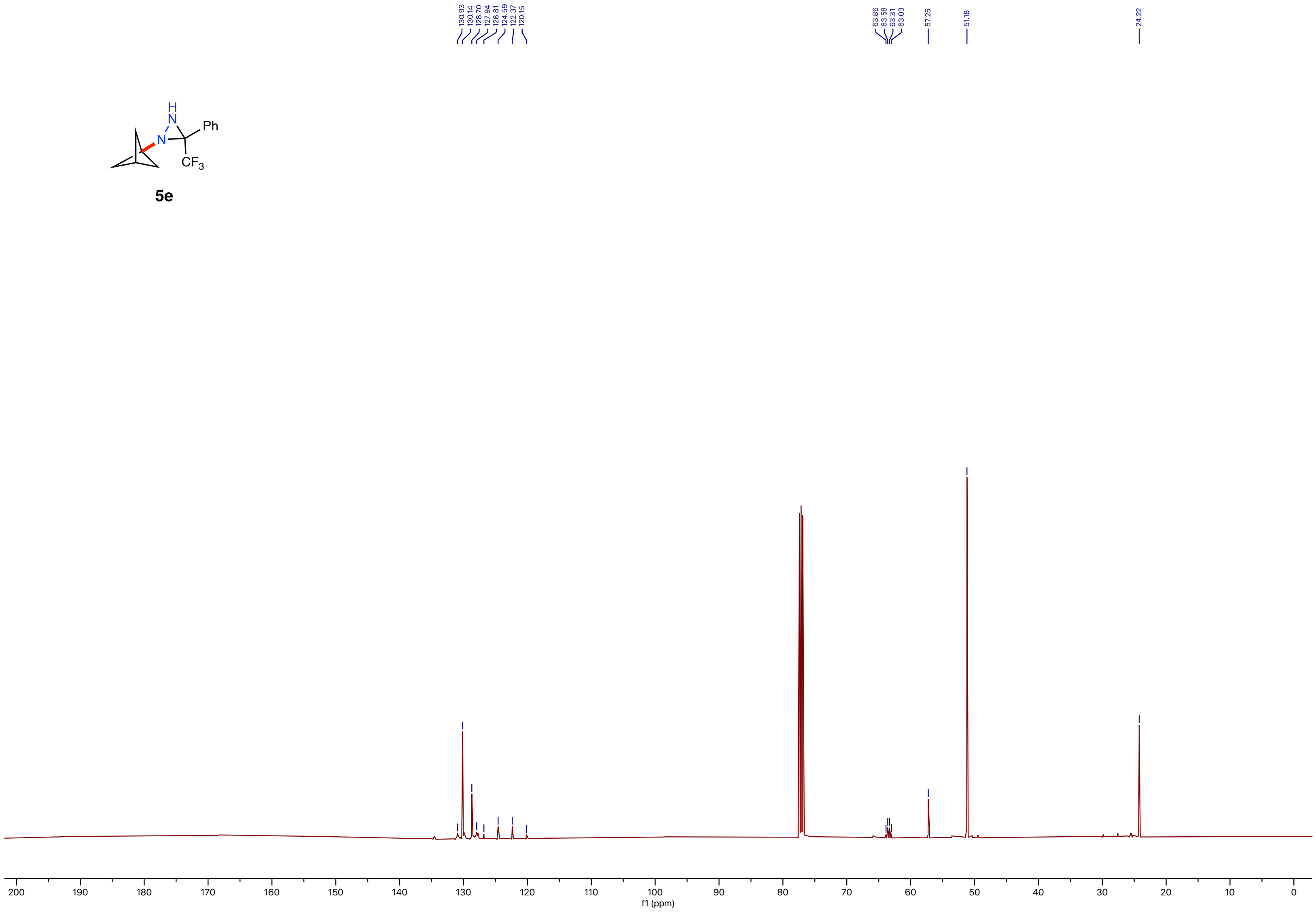

^{19}F NMR of 5e ($CDCl_3$, 471 MHz)

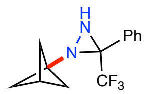

5e

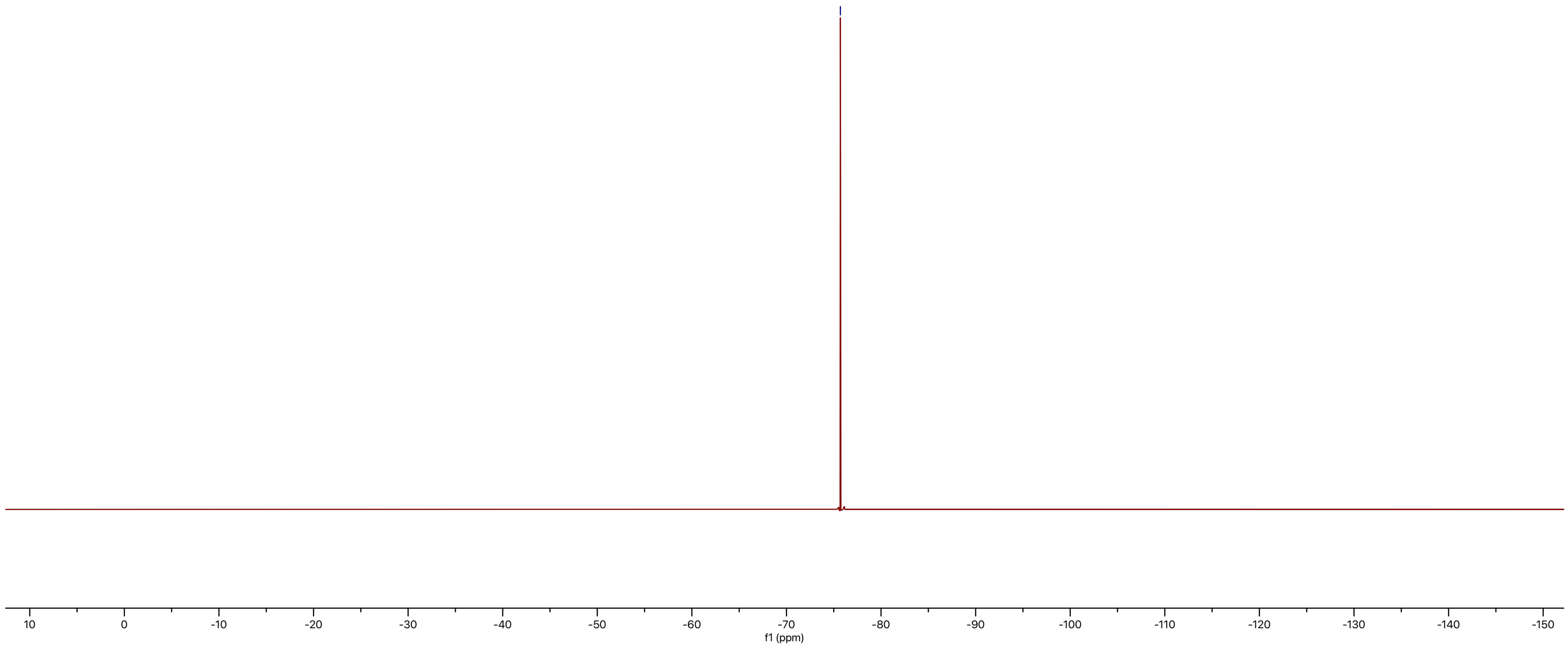

^{1}H NMR of 5f ($CDCl_3$, 500 MHz)

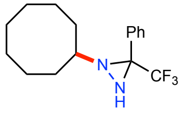

5f

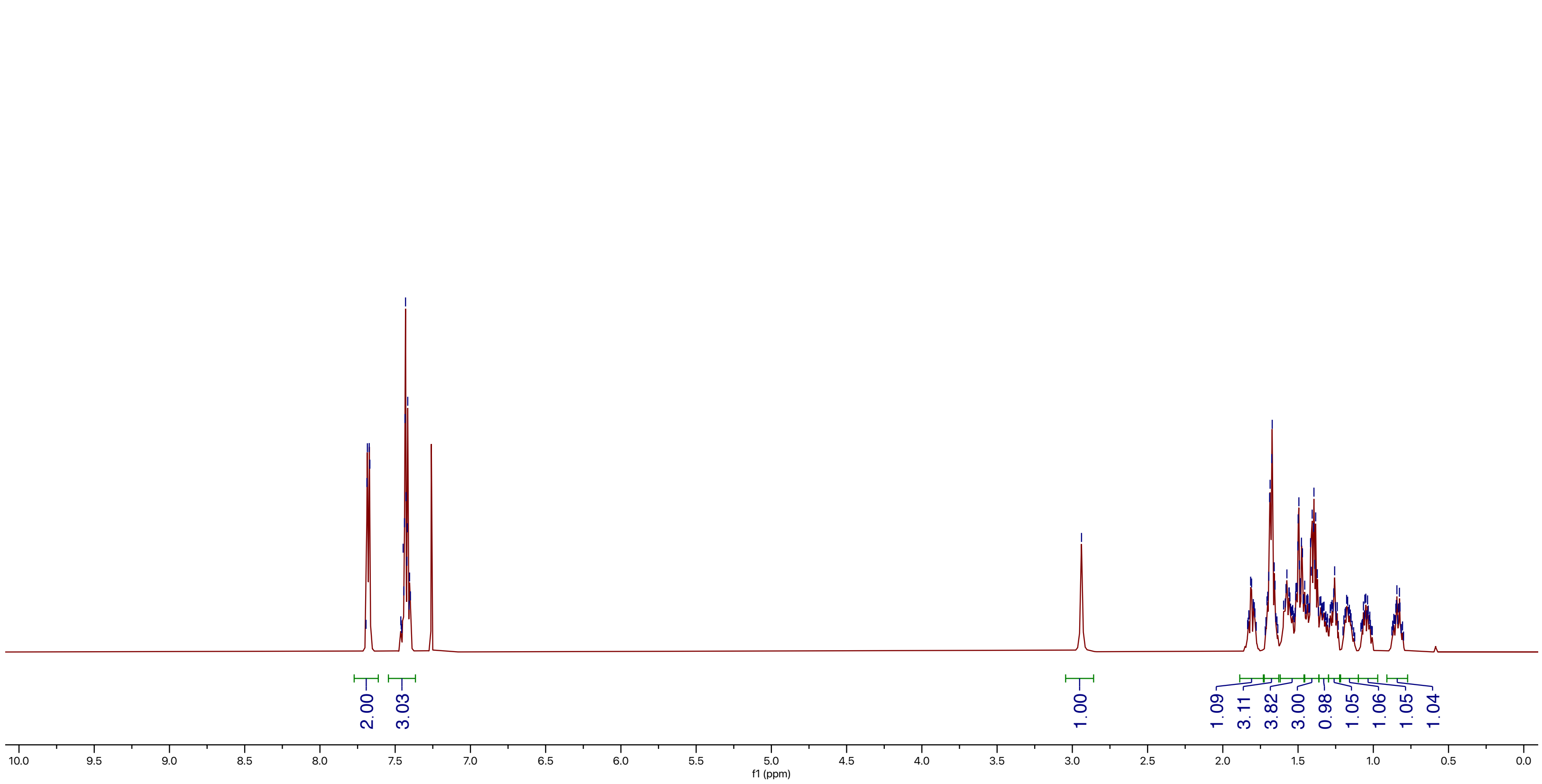

^{13}C NMR of 5f ($CDCl_3$, 126 MHz)

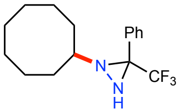

5f

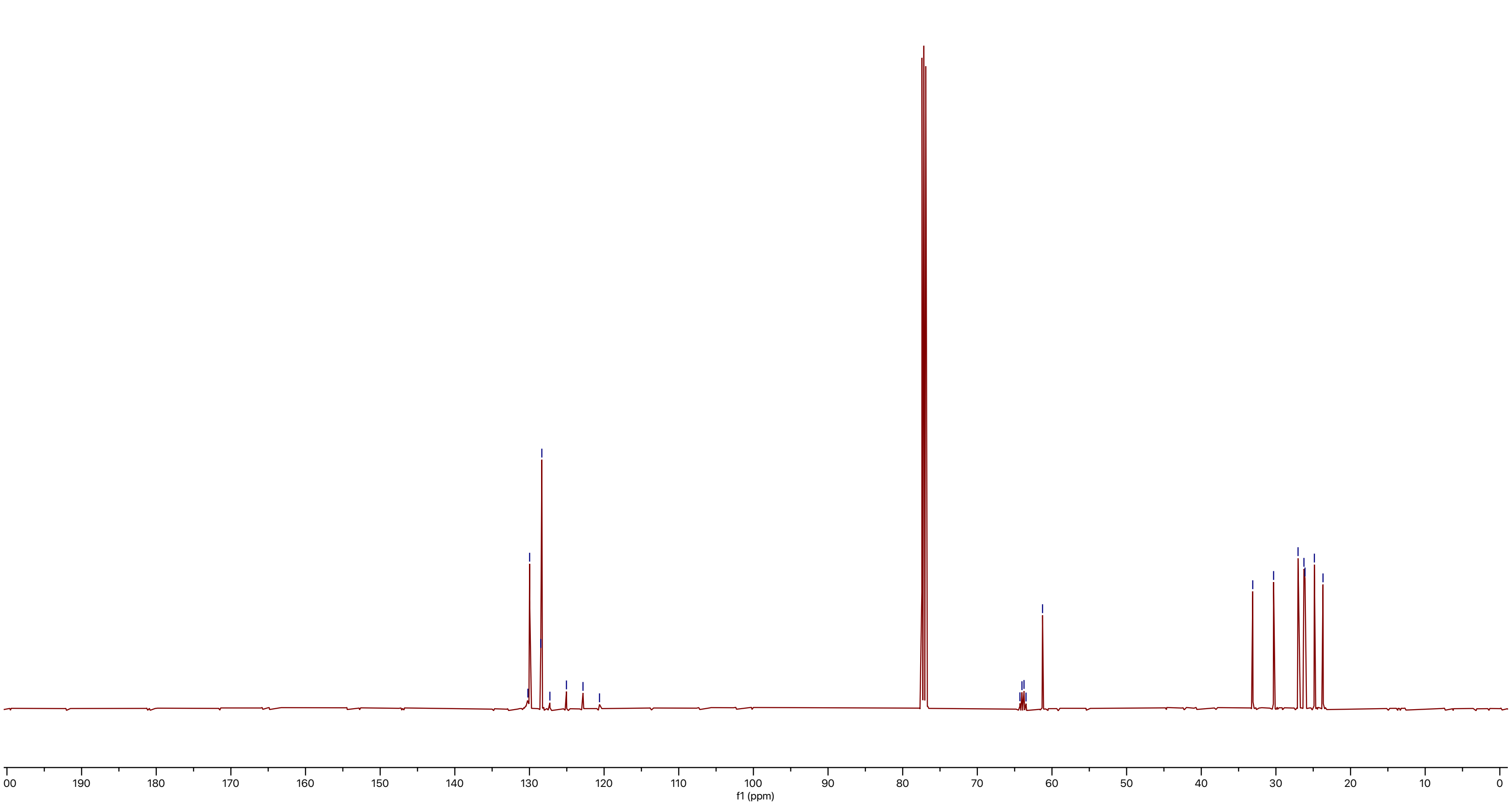

^{19}F NMR of 5f ($CDCl_3$, 471 MHz)

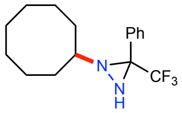

5f

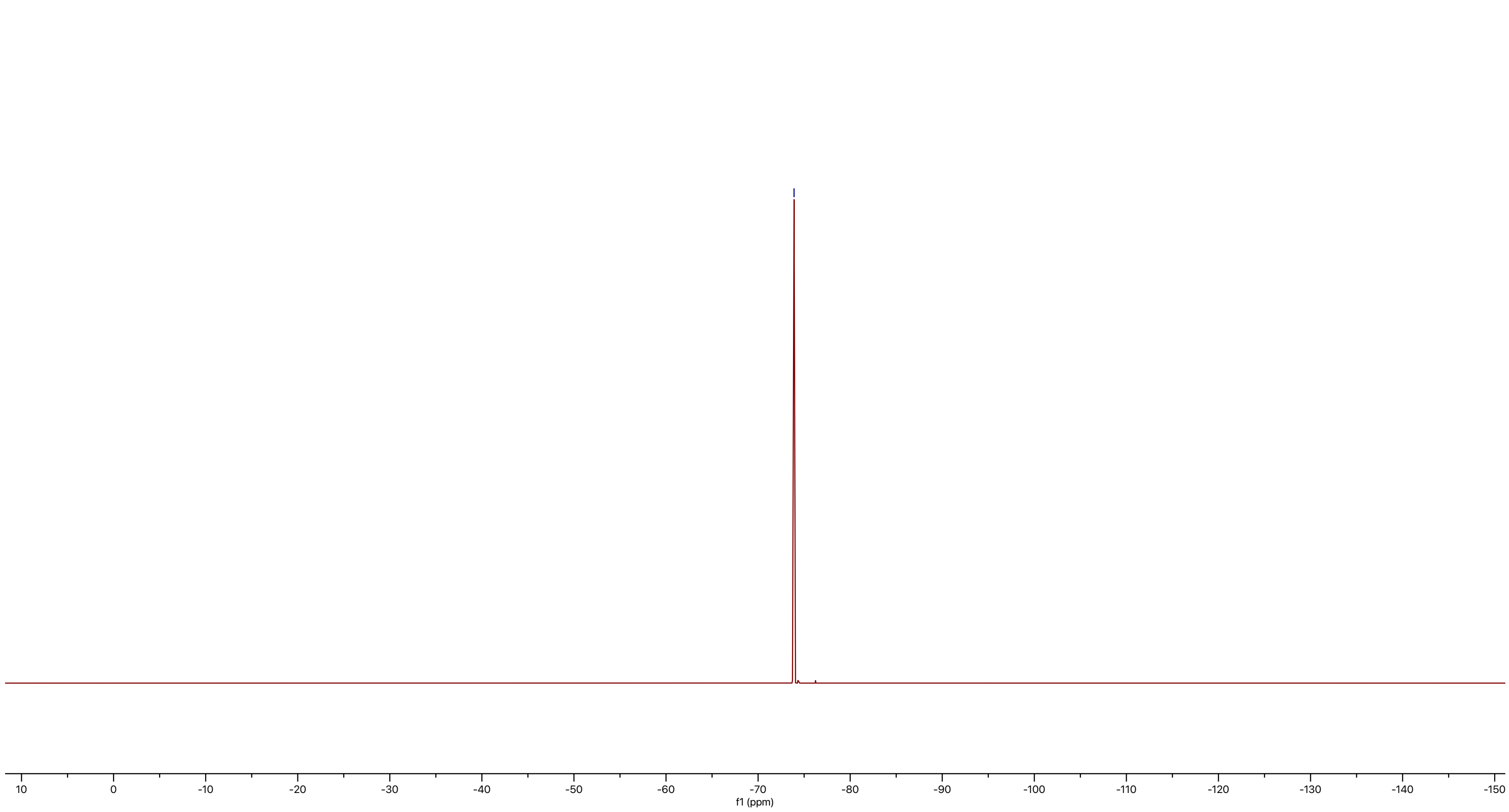

^{1}H NMR of 6a ($CDCl_3$, 500 MHz)

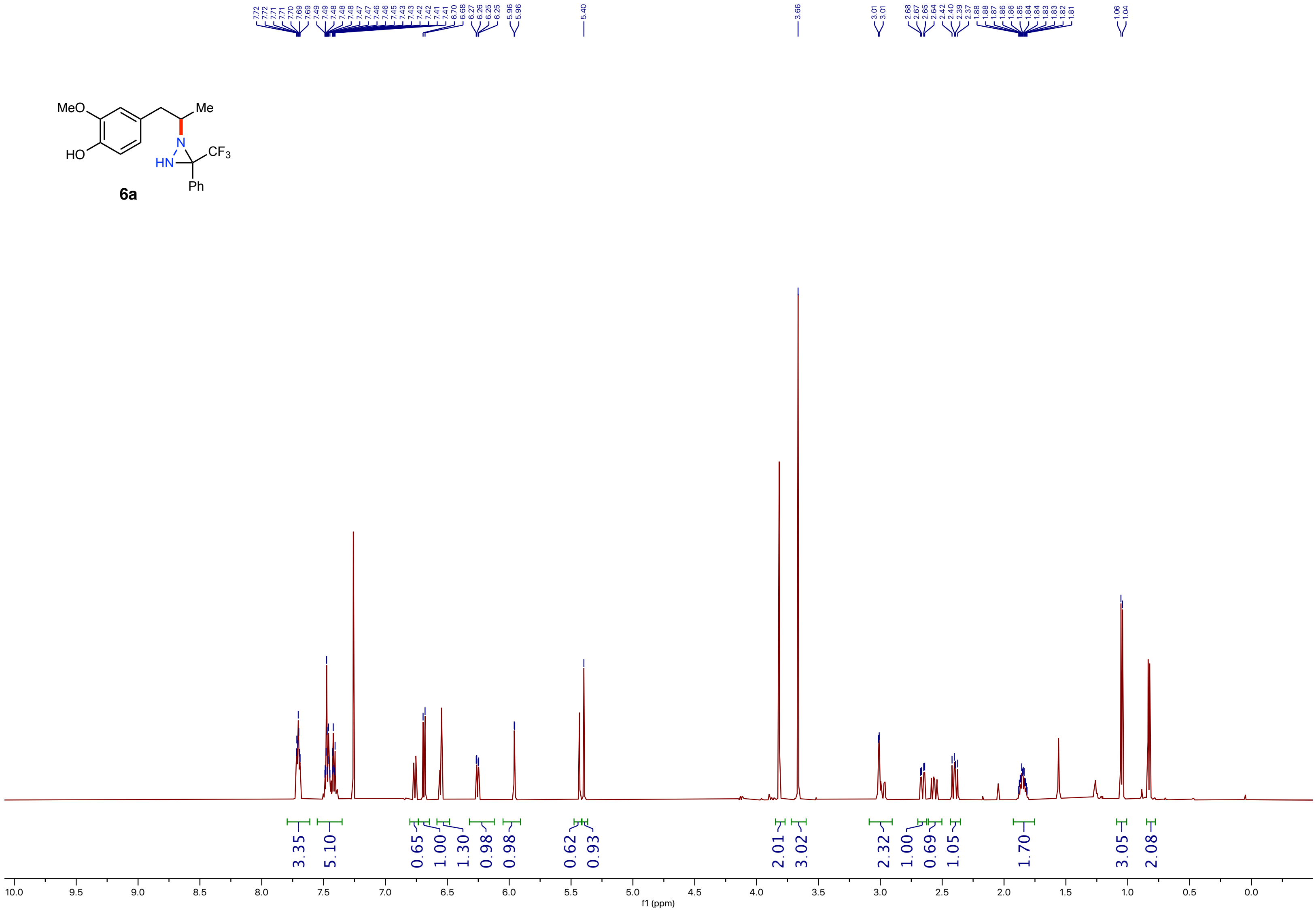

^{13}C NMR of 6a ($CDCl_3$, 126 MHz)

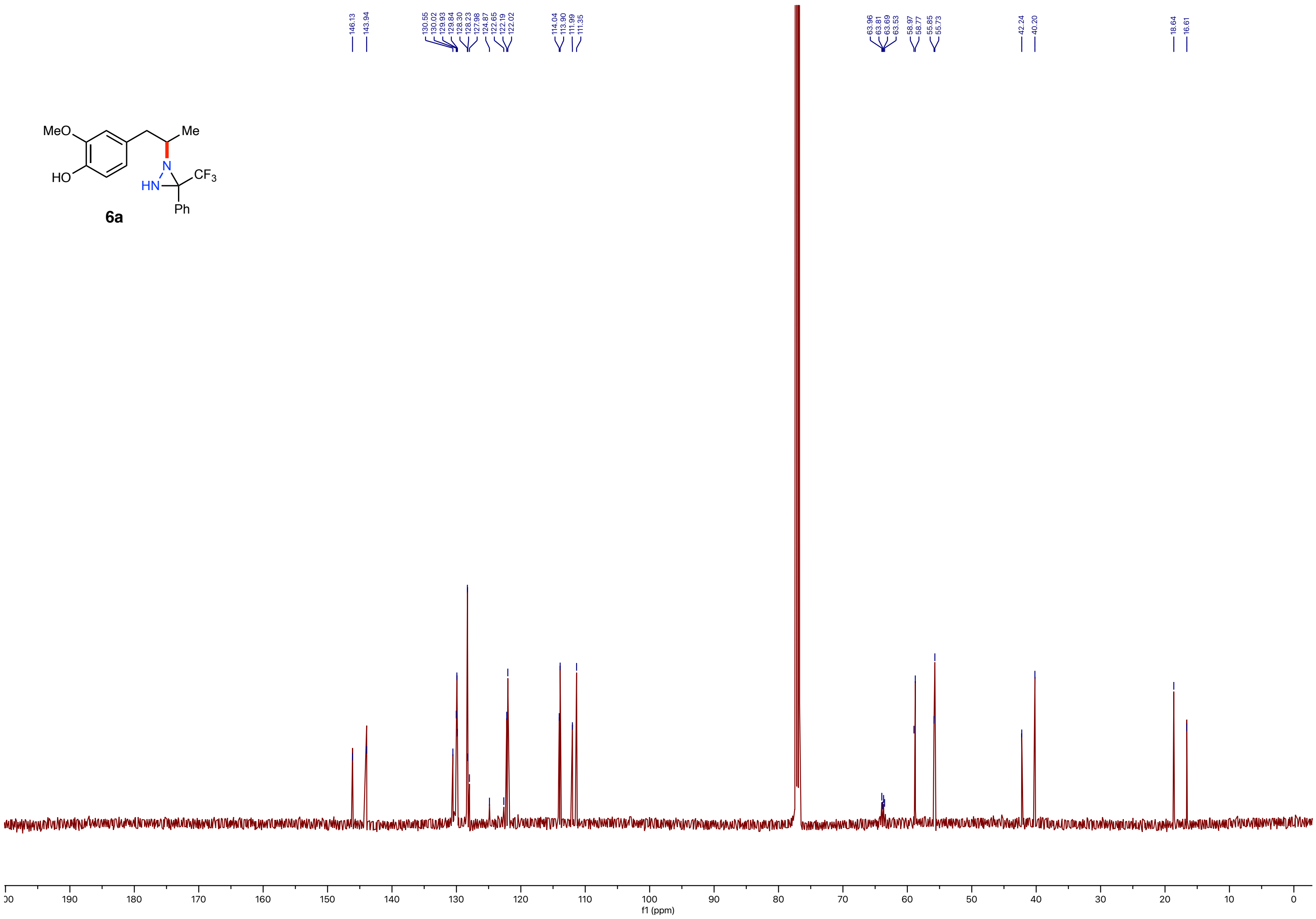

^{19}F NMR of 6a ($CDCl_3$, 471 MHz)

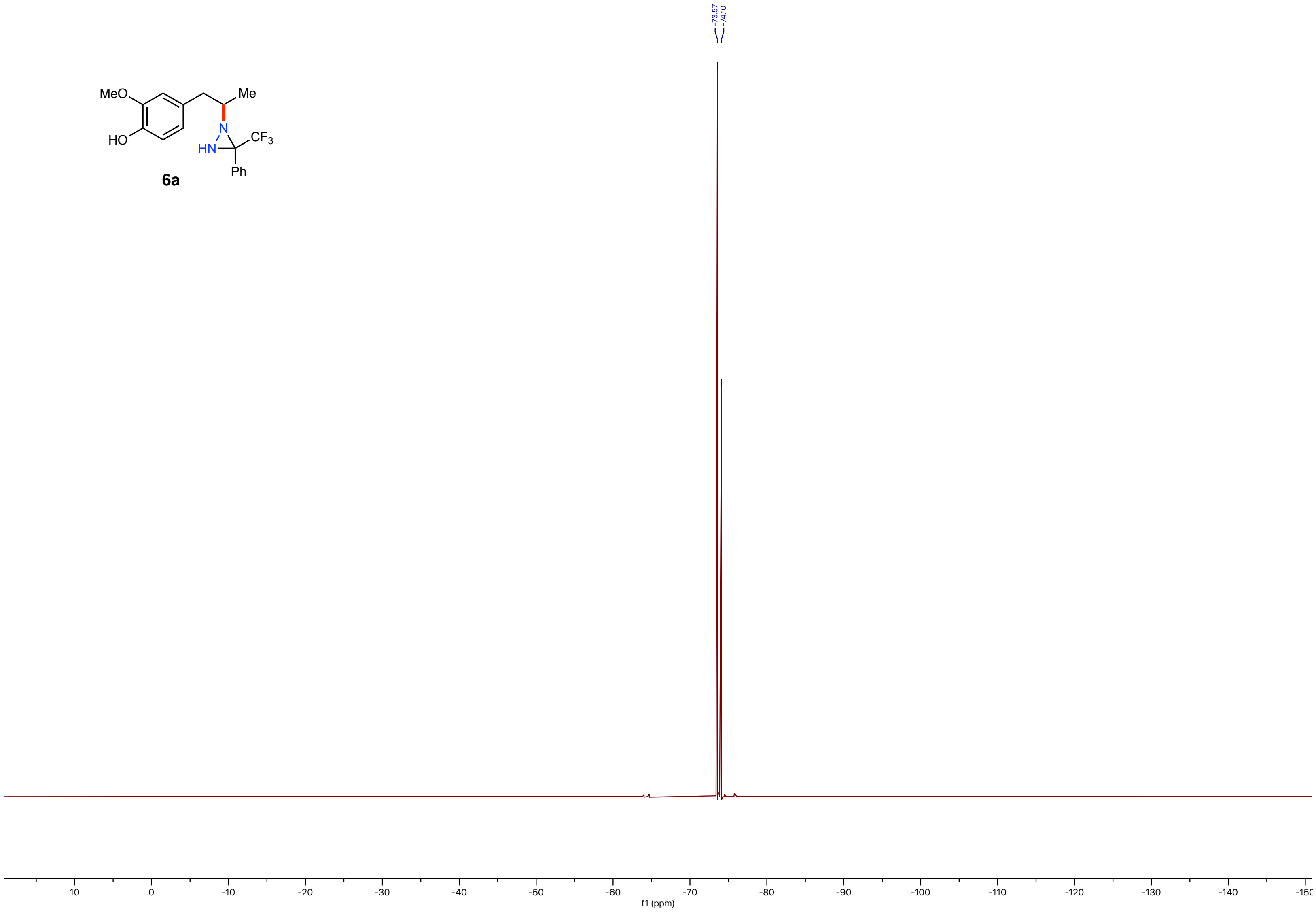

^{1}H NMR of 6b ($CDCl_3$, 500 MHz)

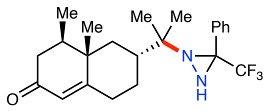

6b

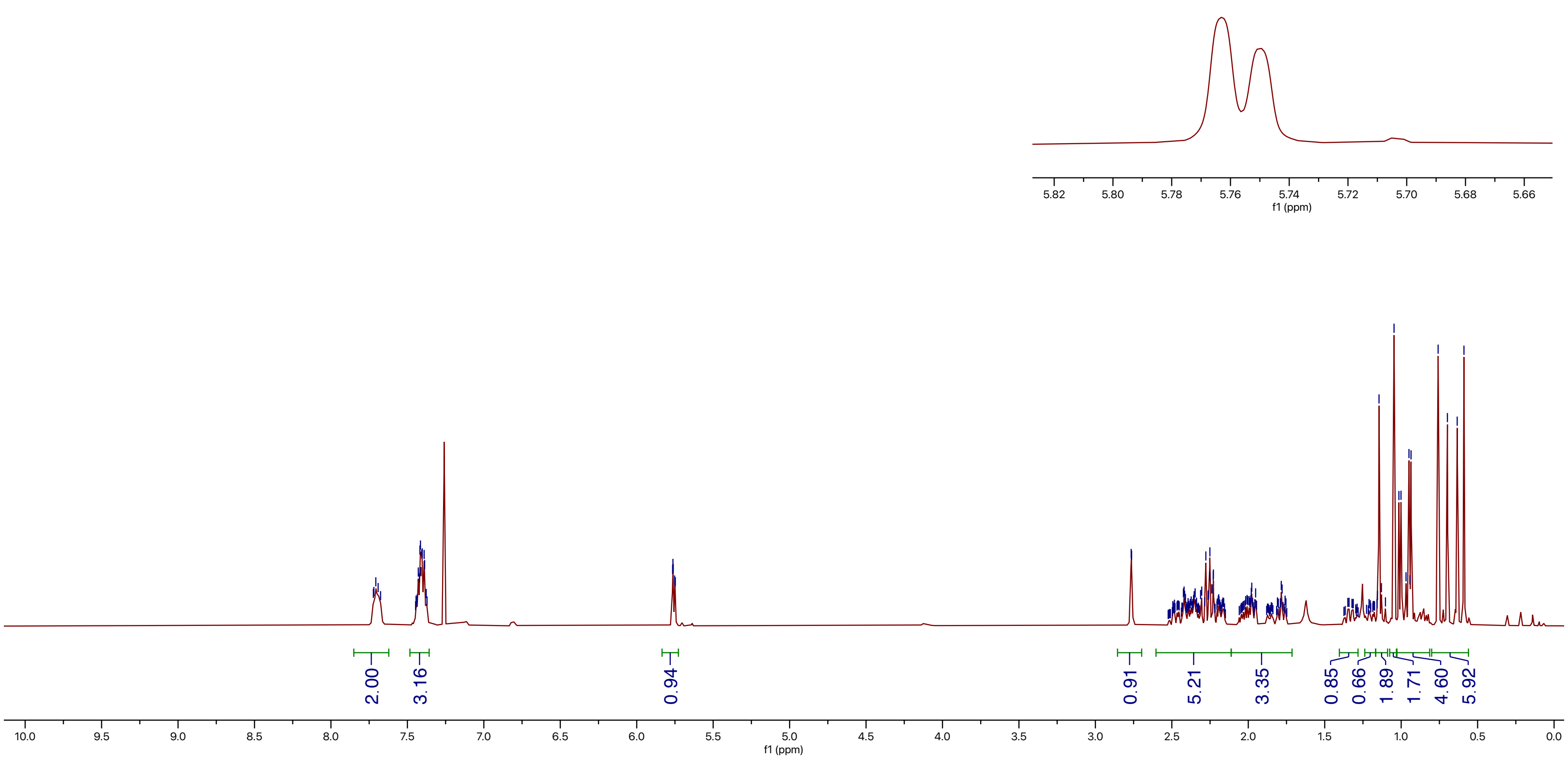

^{13}C NMR of 6b ($CDCl_3$, 126 MHz)

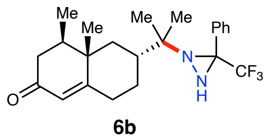

6b

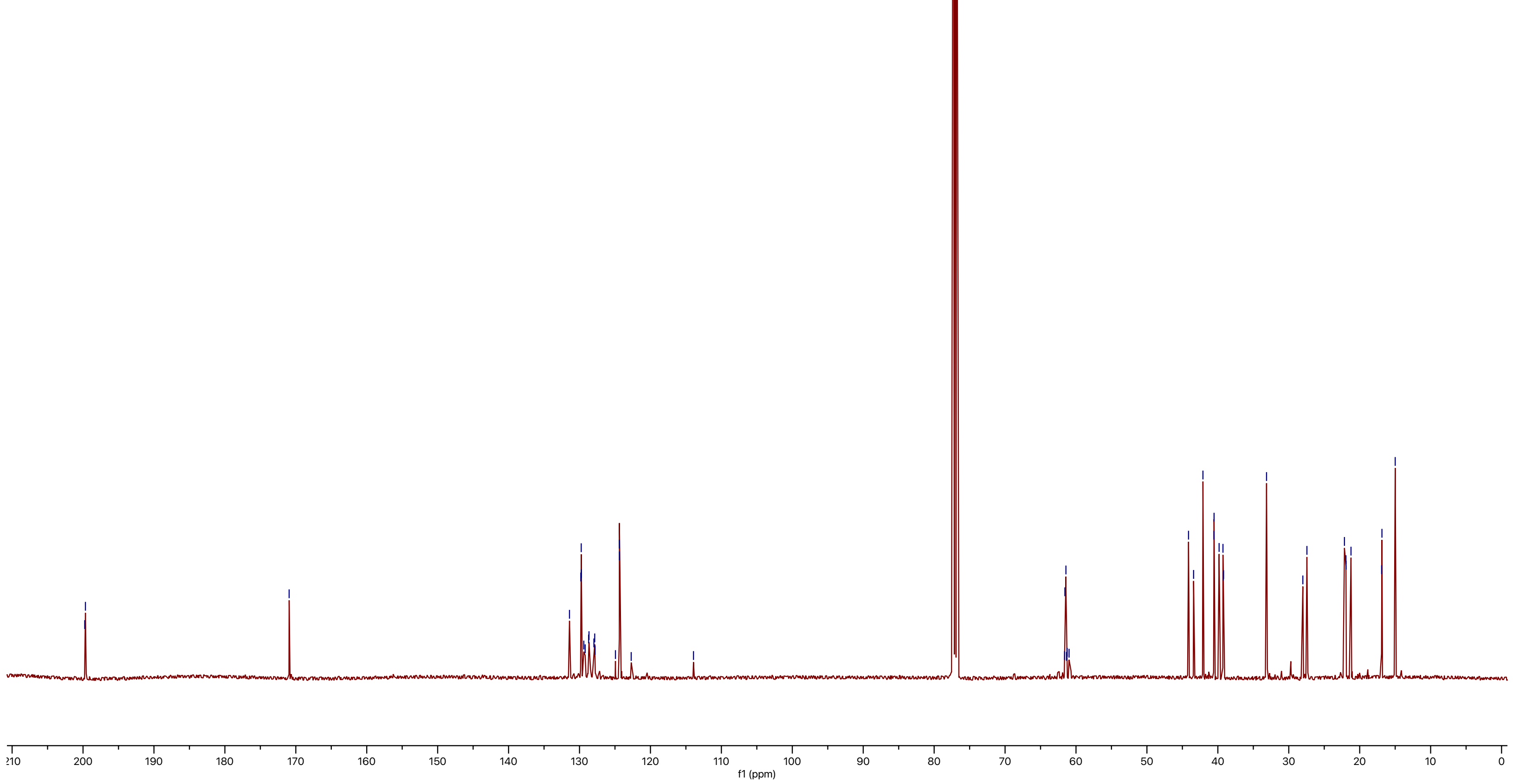

^{19}F NMR of 6b ($CDCl_3$, 471 MHz)

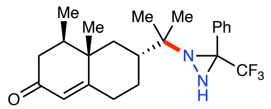

6b

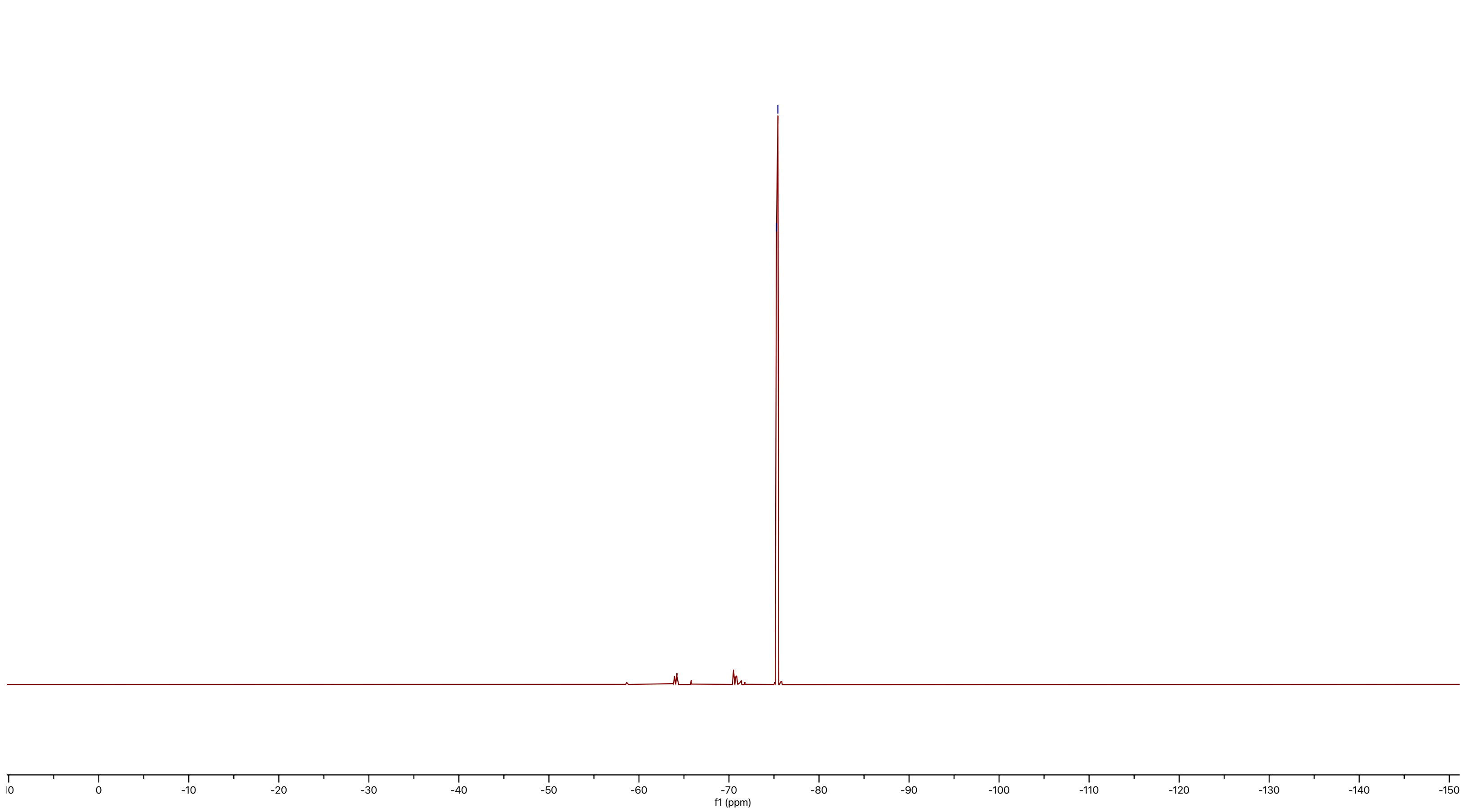

^{1}H NMR of 6c (mixture of diastereomers, $CDCl_3$, 500 MHz)

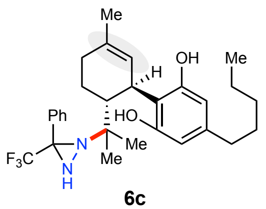

6c

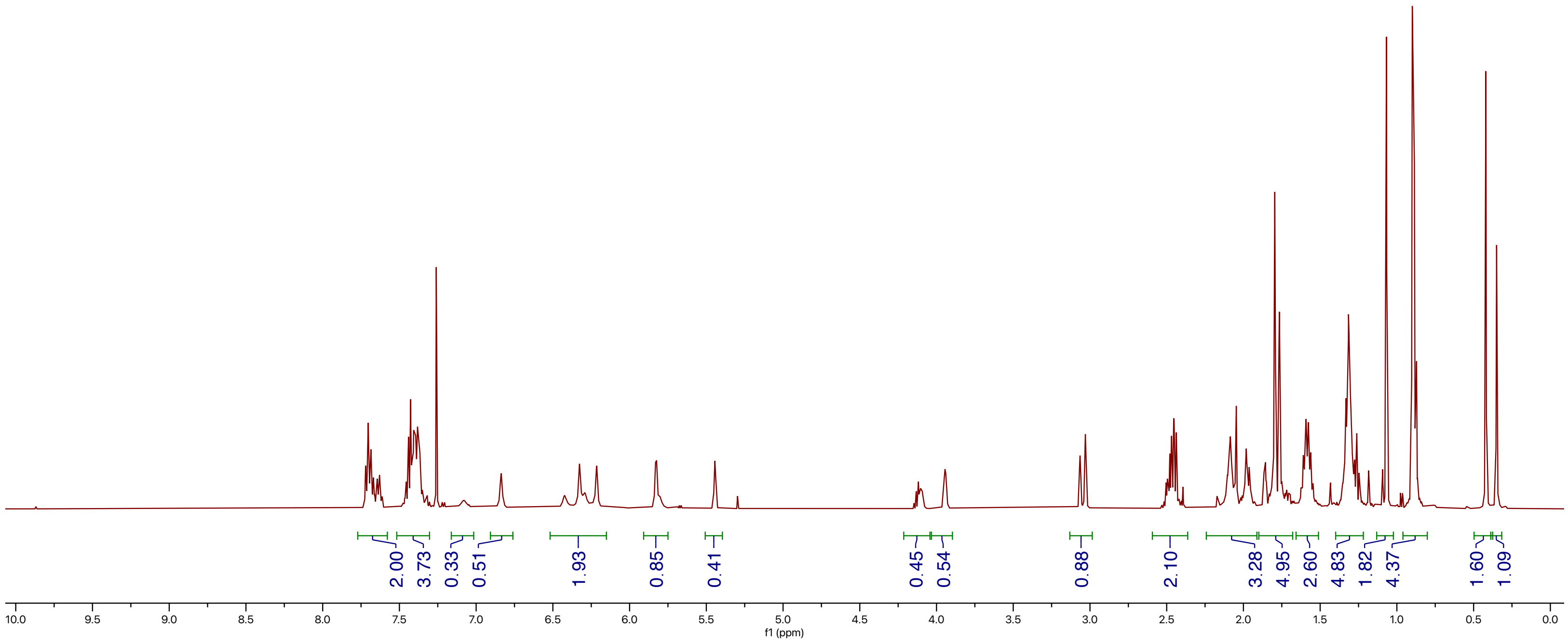

^{13}C NMR of 6c (mixture of diastereomers, $CDCl_3$, 126 MHz)

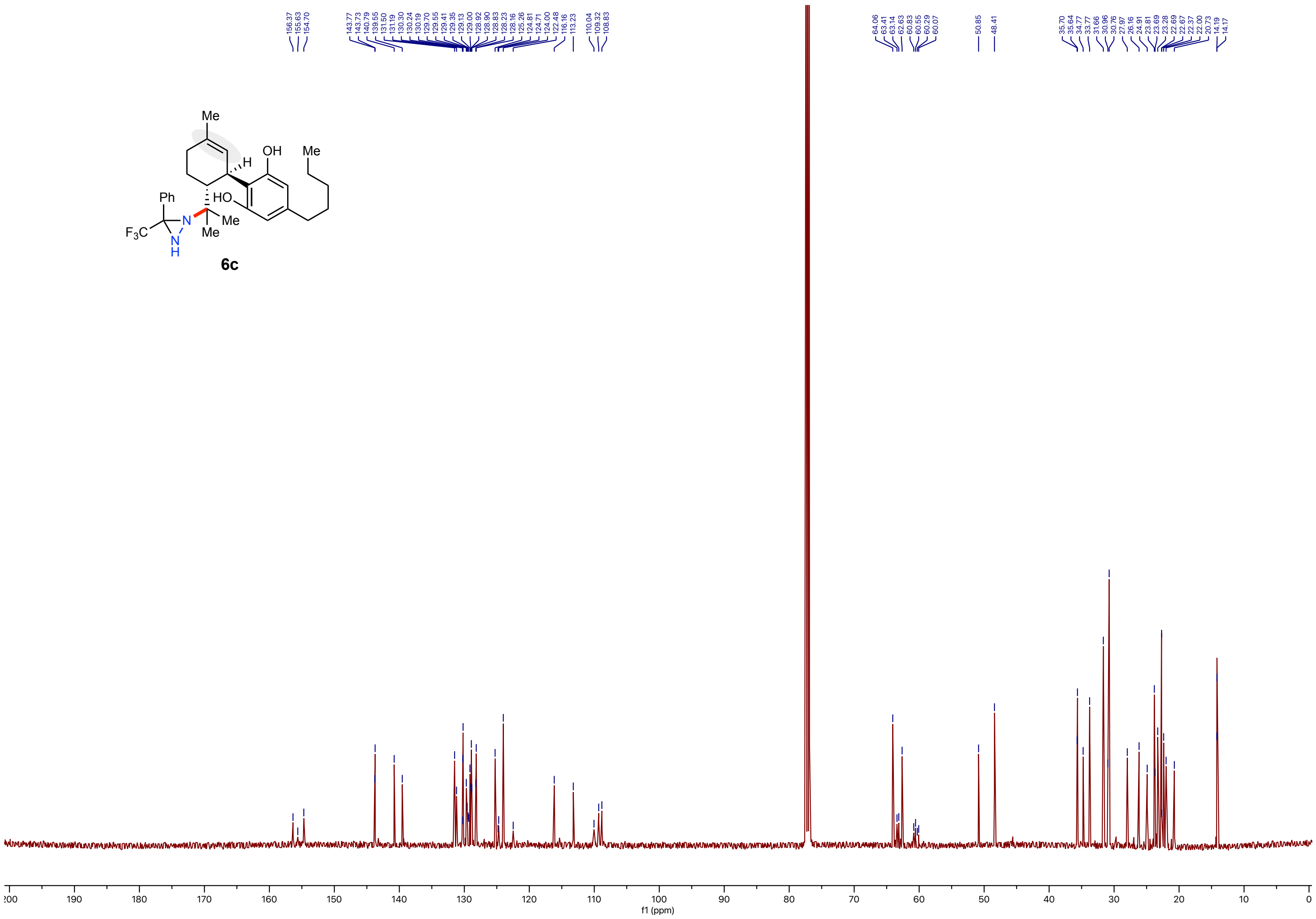

^{19}F NMR of 6c (mixture of diastereomers, $CDCl_3$, 471 MHz)

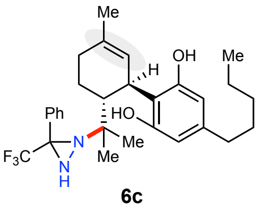

6c

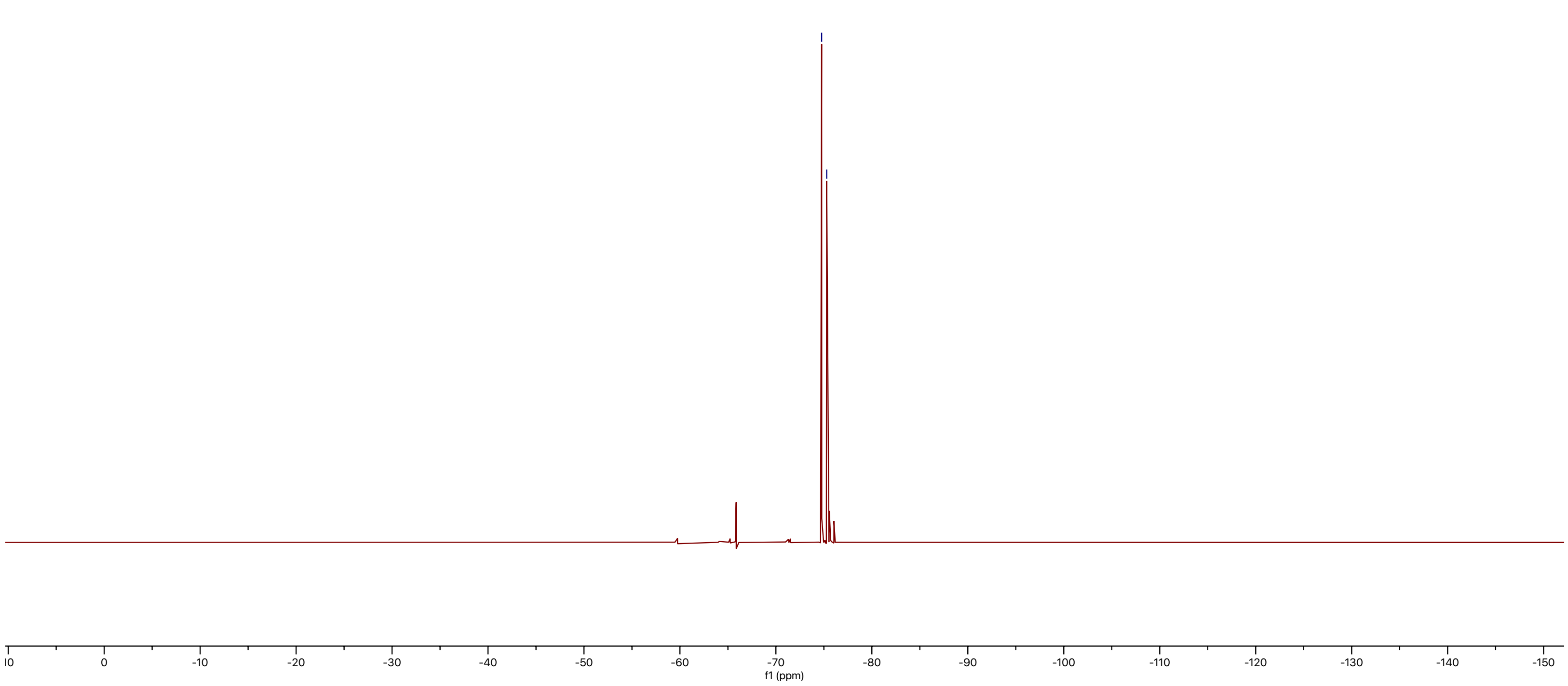

^{1}H NMR of 6c (Major isomer, $CDCl_3$, 500 MHz)

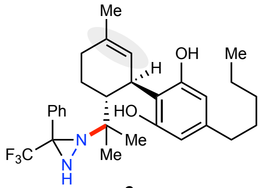

6c

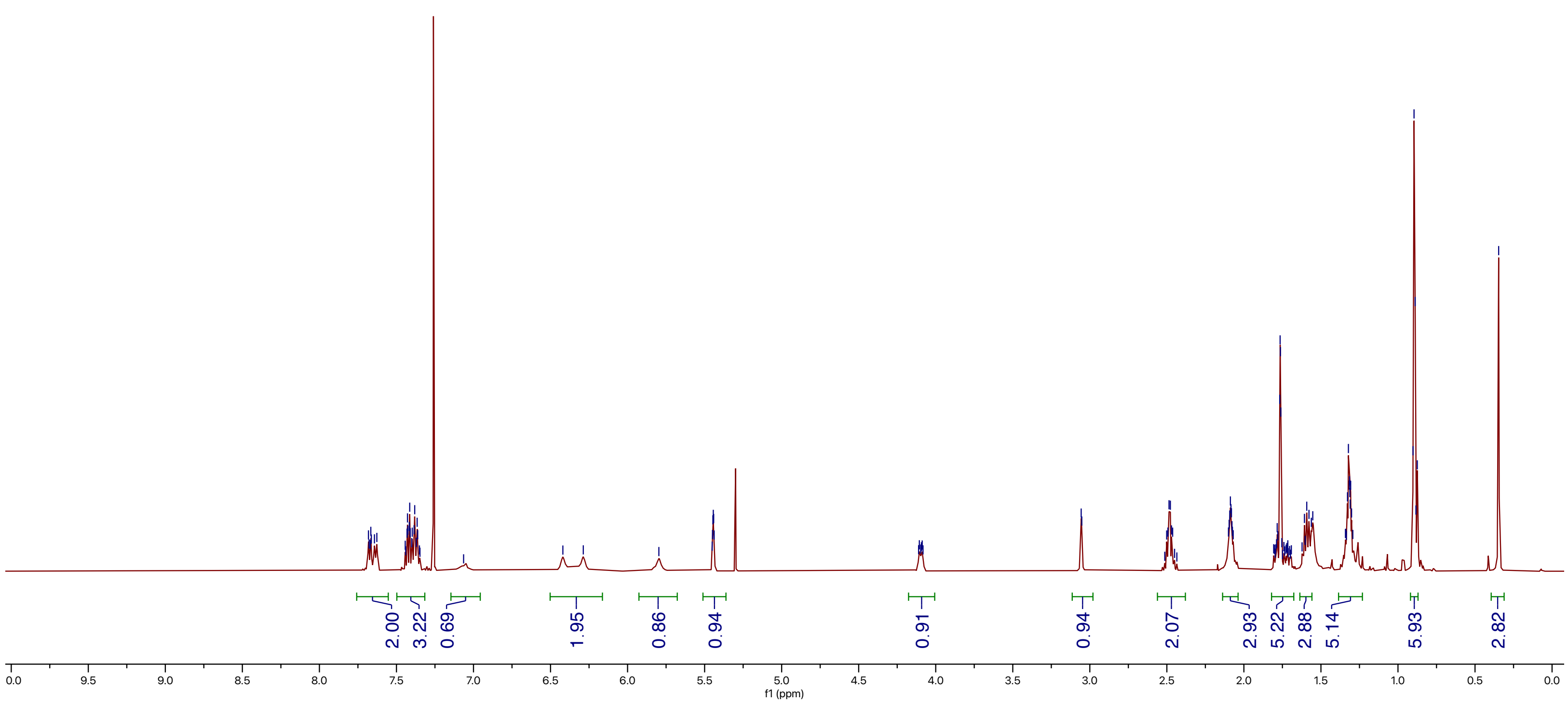

^{19}F NMR of 6c (Major isomer, $CDCl_3$, 471 MHz)

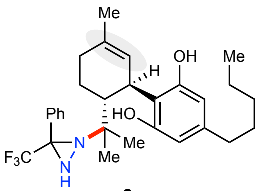

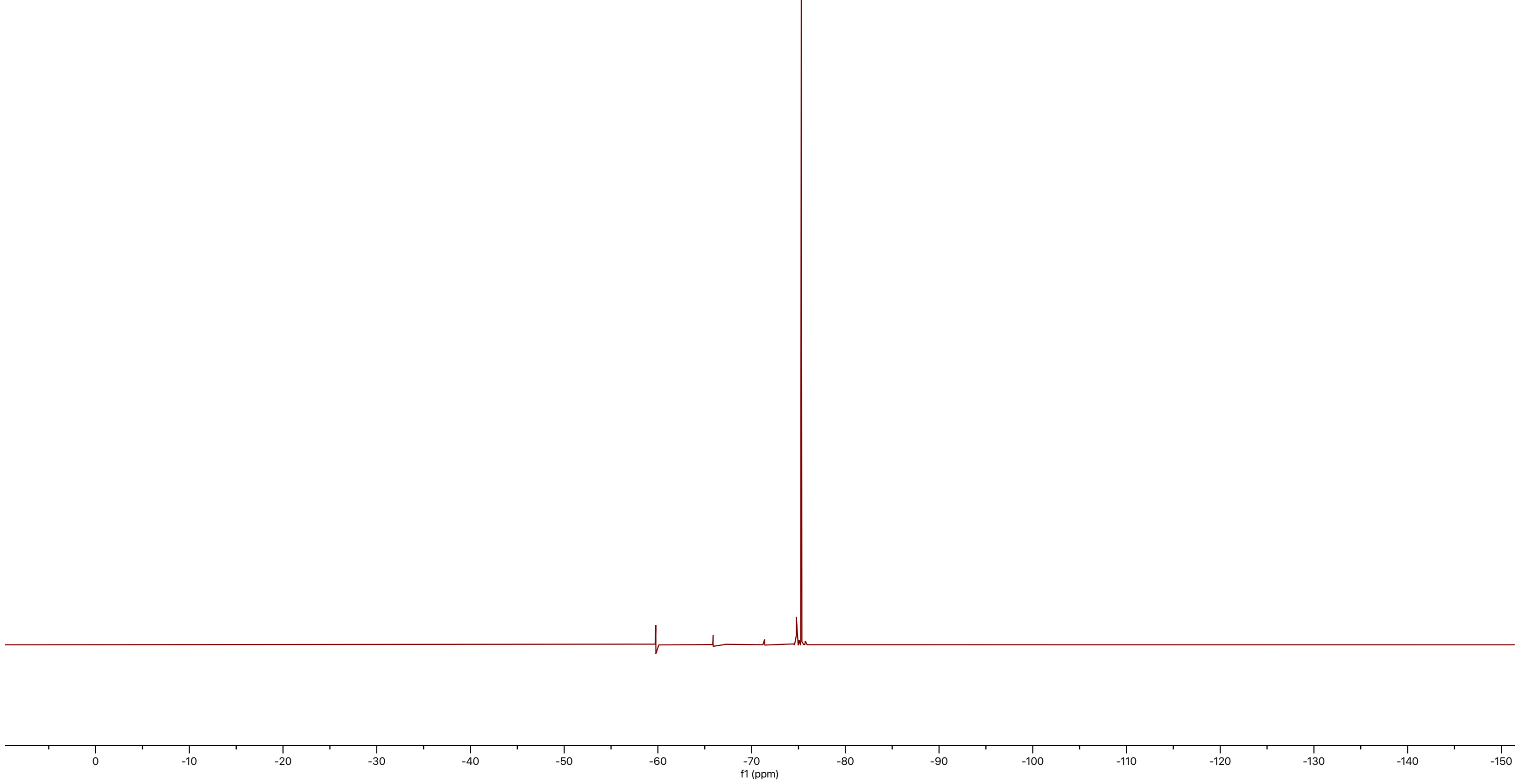

^1H NMR of 6c (Minor isomer, $CDCl_3$, 500 MHz)

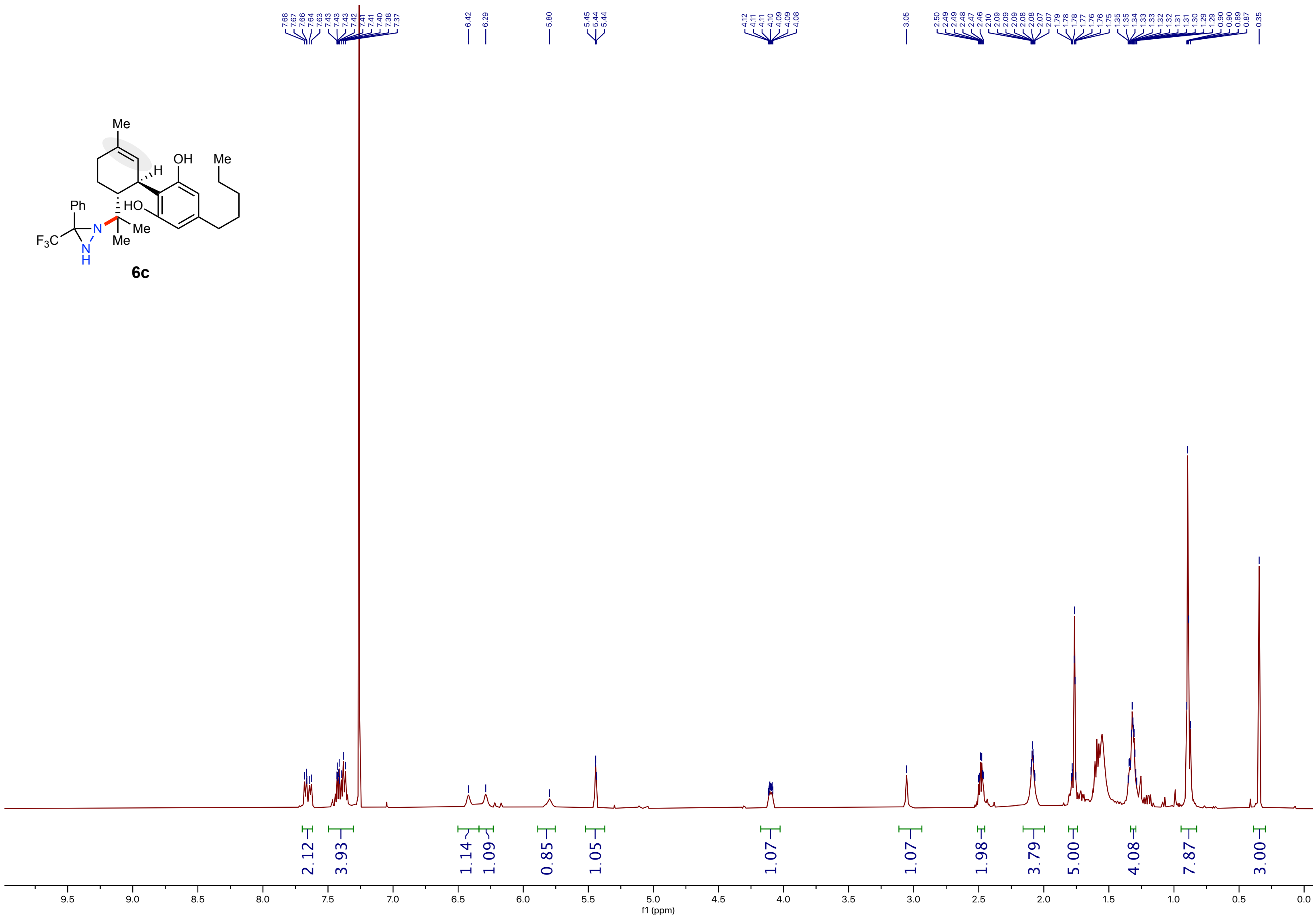

^{19}F NMR of 6c (Minor isomer, $CDCl_3$, 471 MHz)

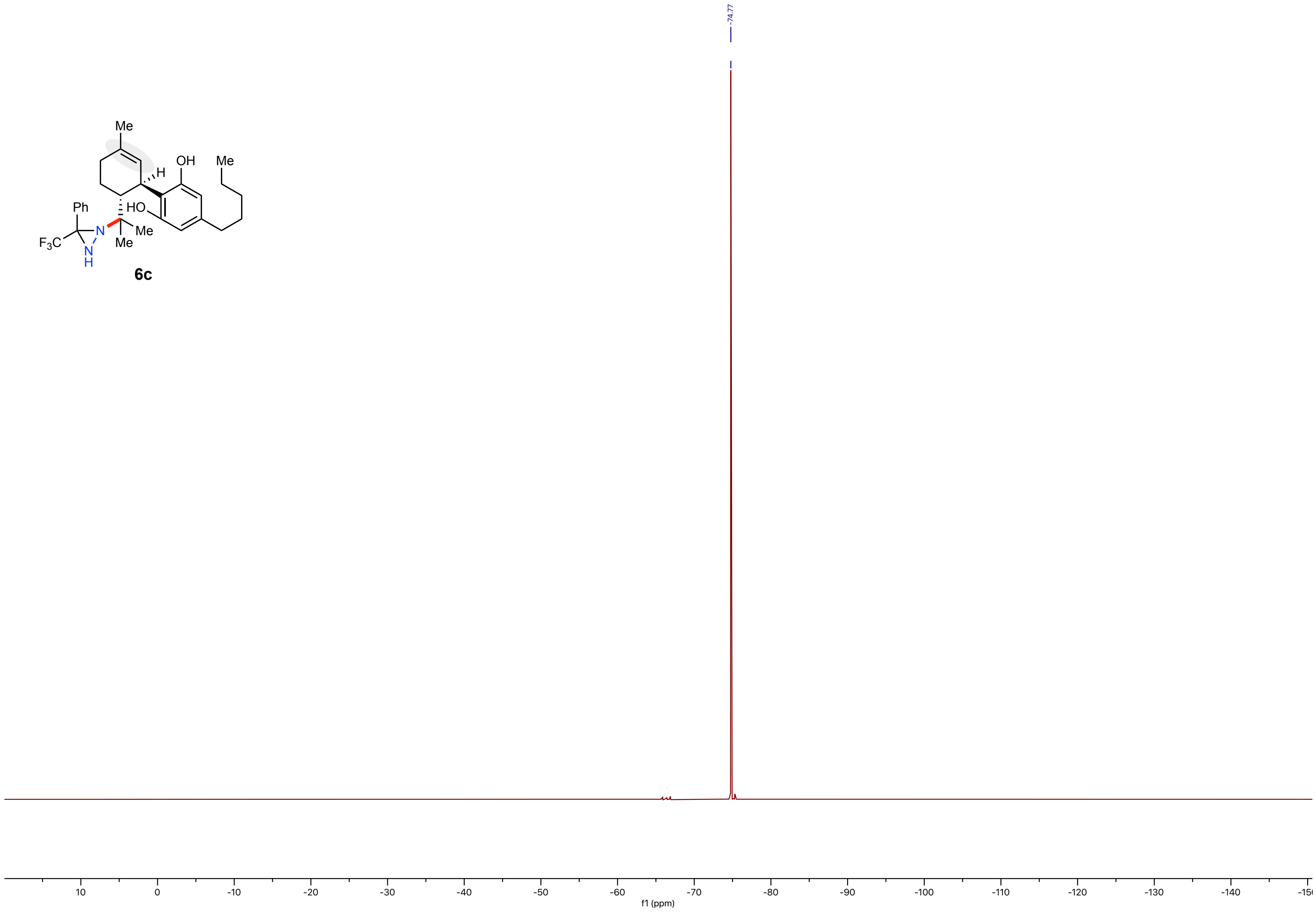

^{1}H NMR of 6d ($CDCl_3$, 500 MHz)

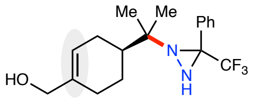

6d

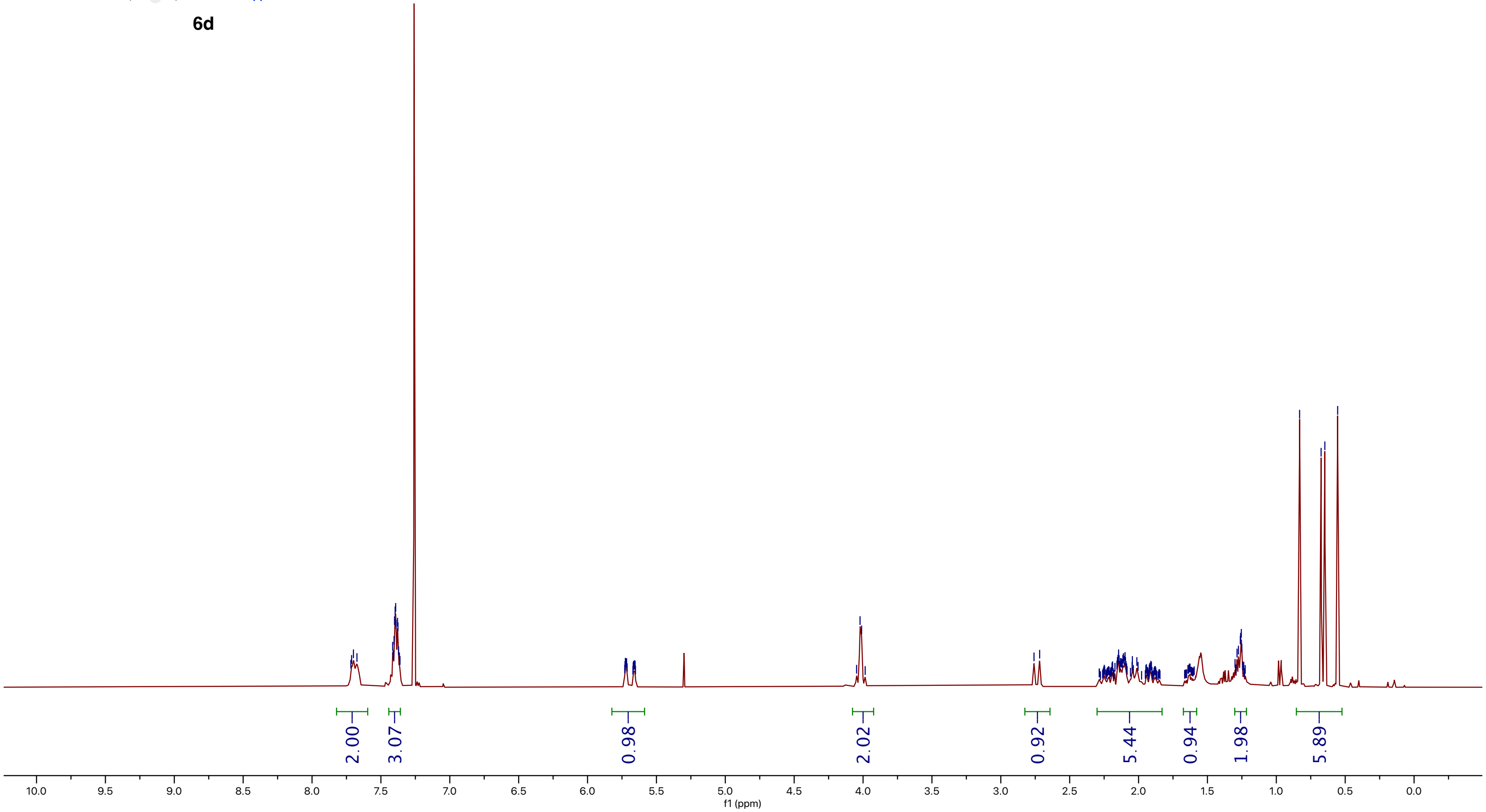

^{13}C NMR of 6d (CDCl$_3$, 126 MHz)

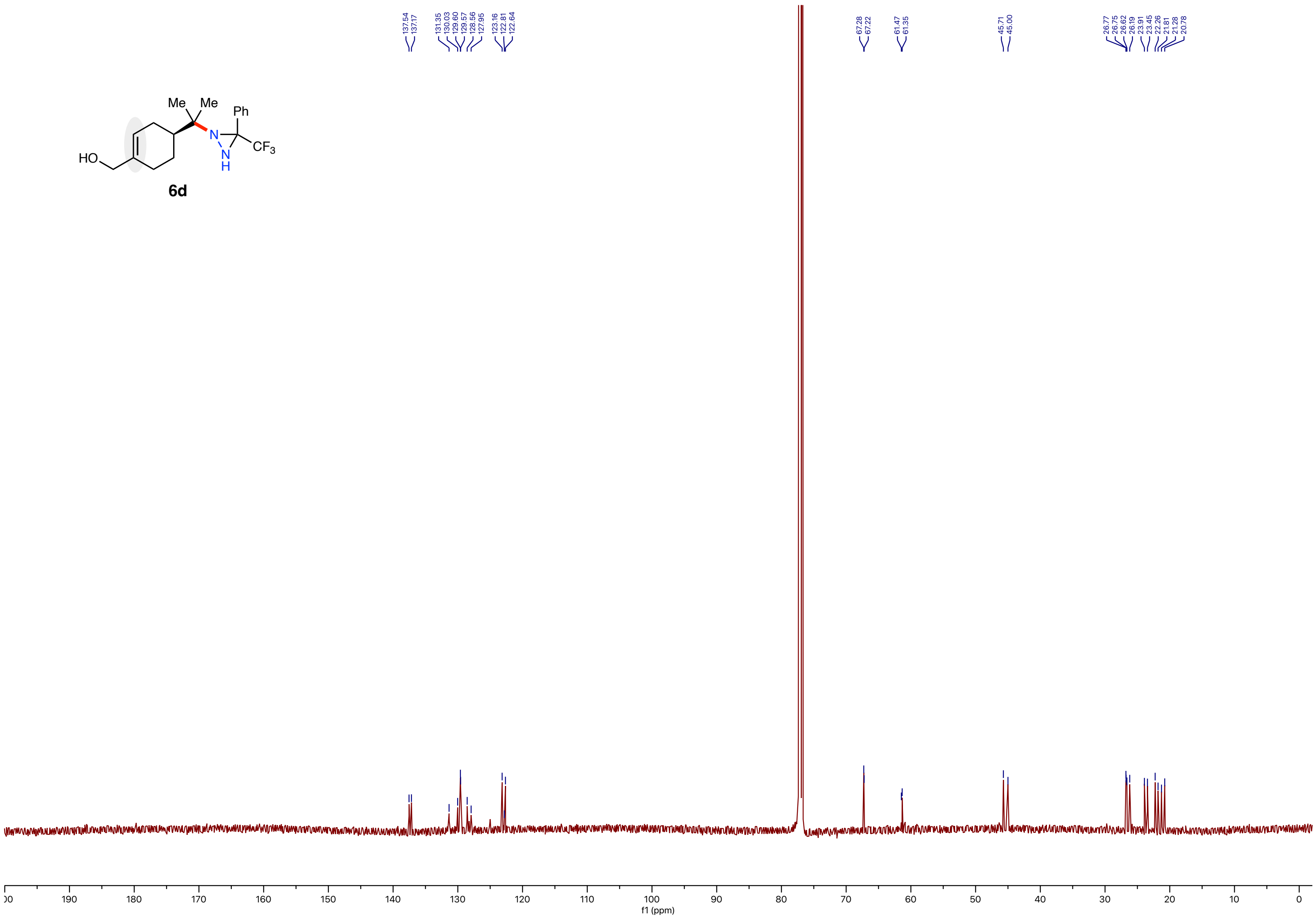

^{19}F NMR of 6d ($CDCl_3$, 471 MHz)

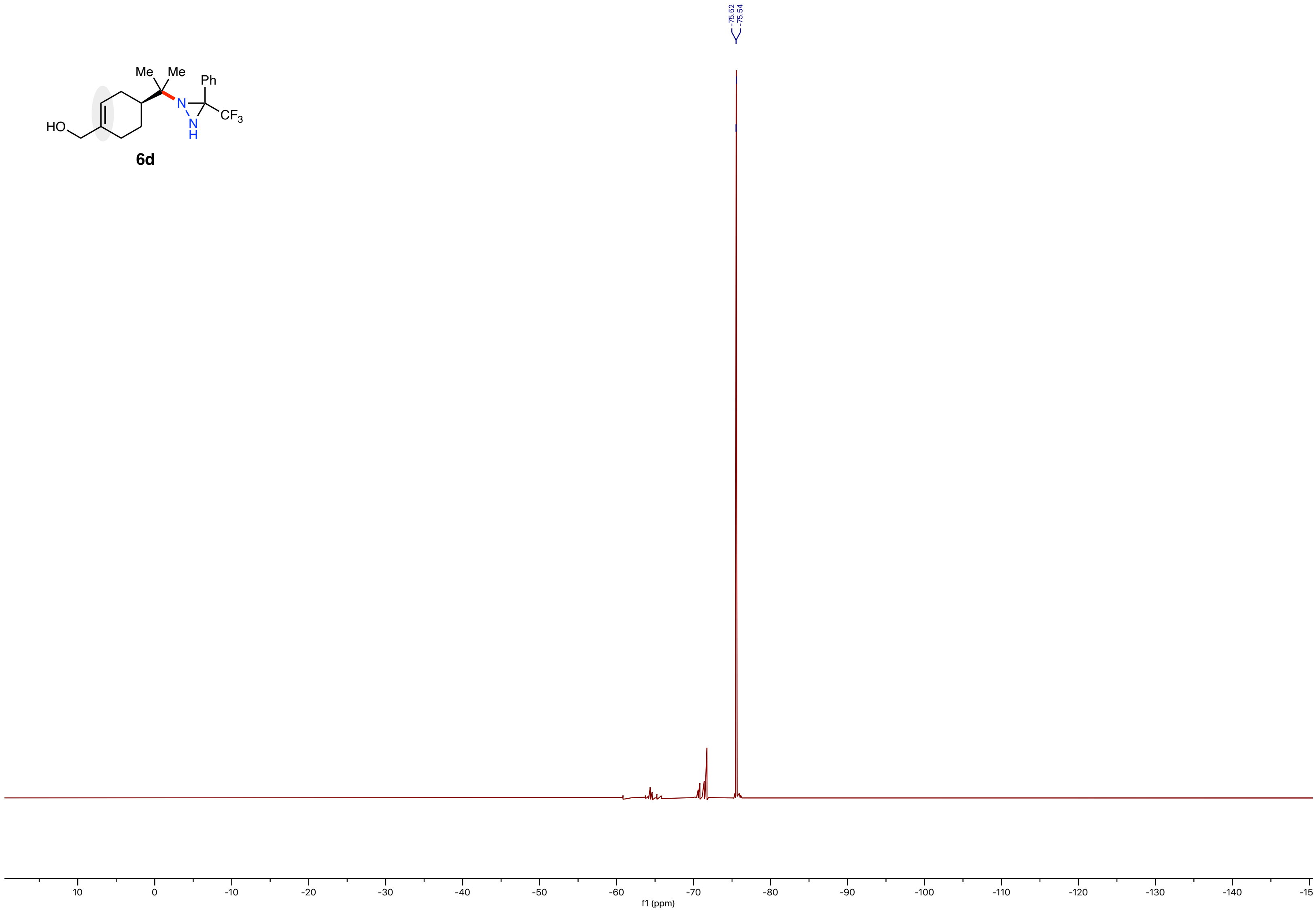

^{1}H NMR of 6e ($CDCl_3$, 500 MHz)

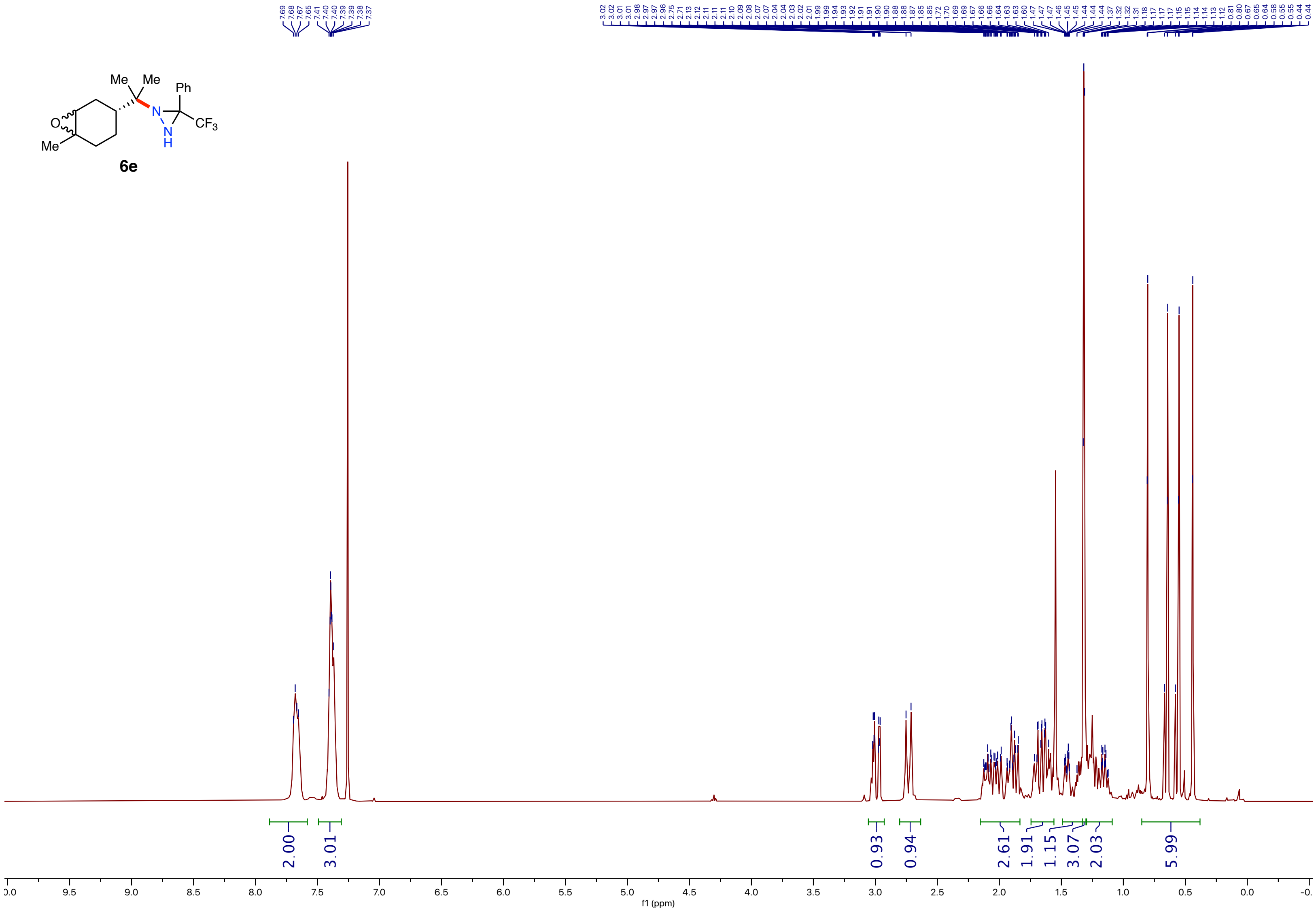

^{13}C NMR of 6e ($CDCl_3$, 126 MHz)

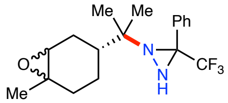

6e

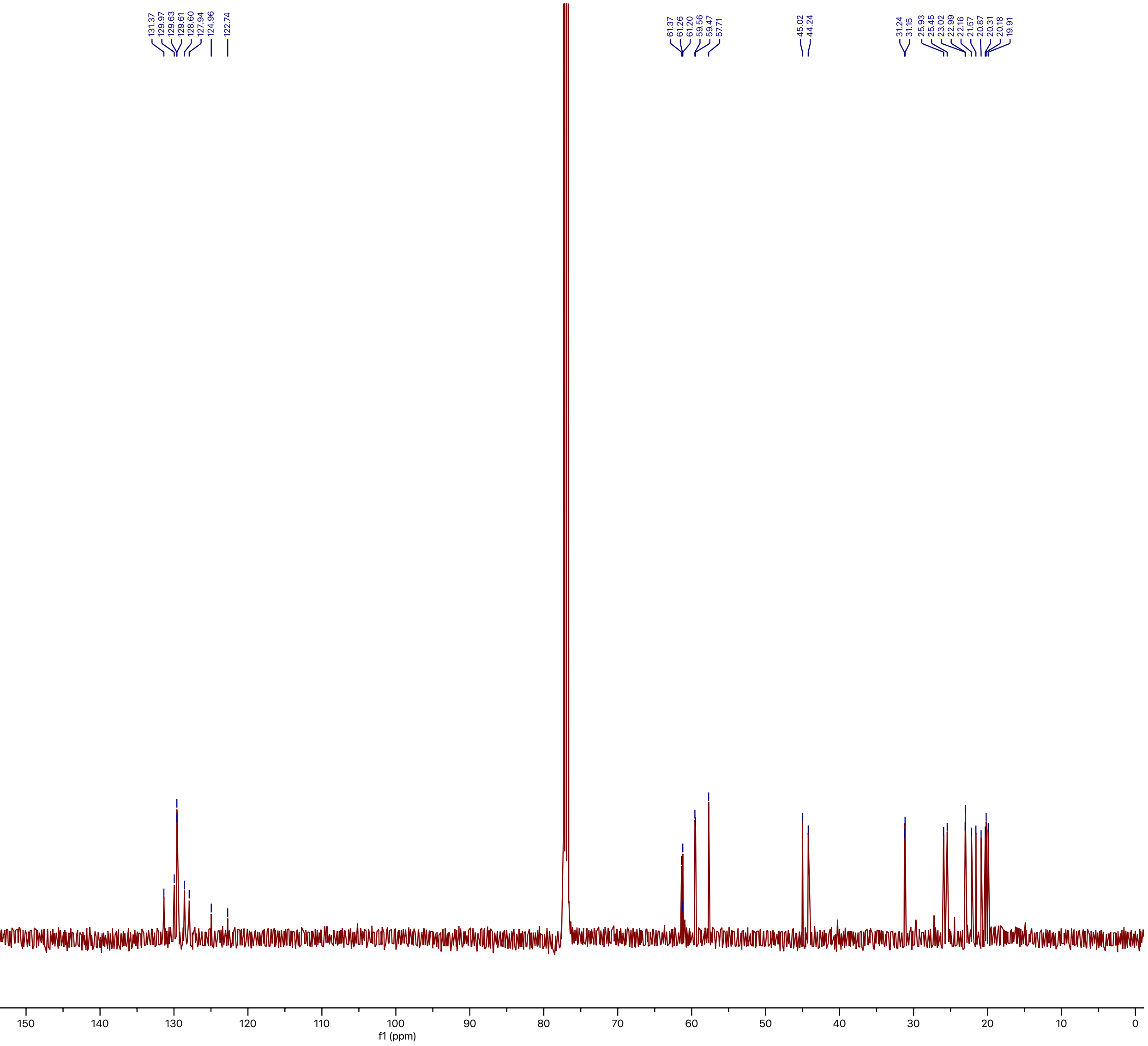

^{19}F NMR of 6e ($CDCl_3$, 471 MHz)

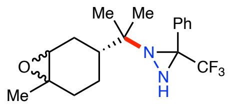

6e

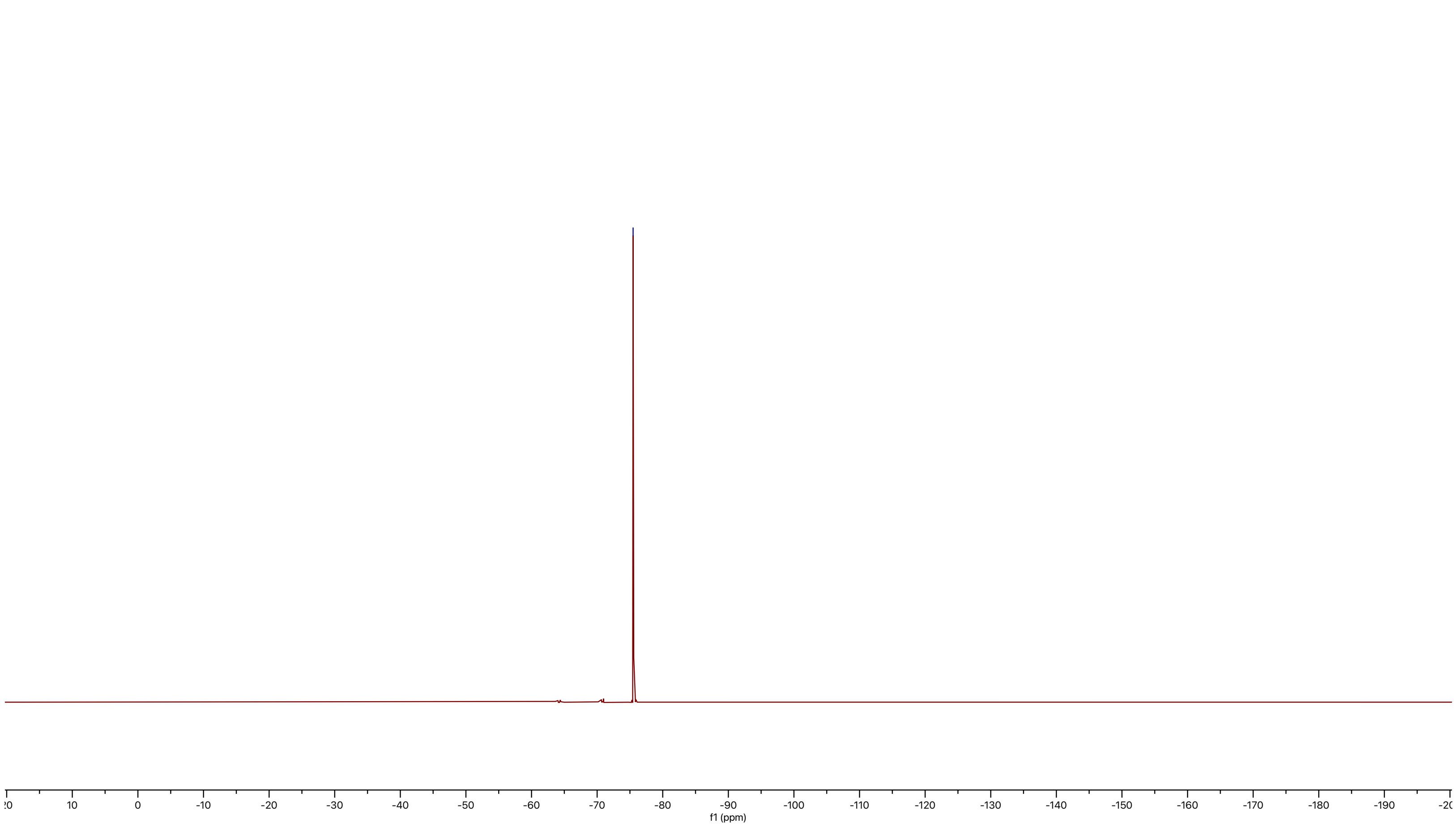

^{1}H NMR of 6f ($CDCl_3$, 500 MHz)

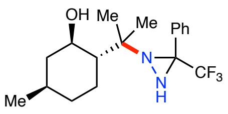

6f

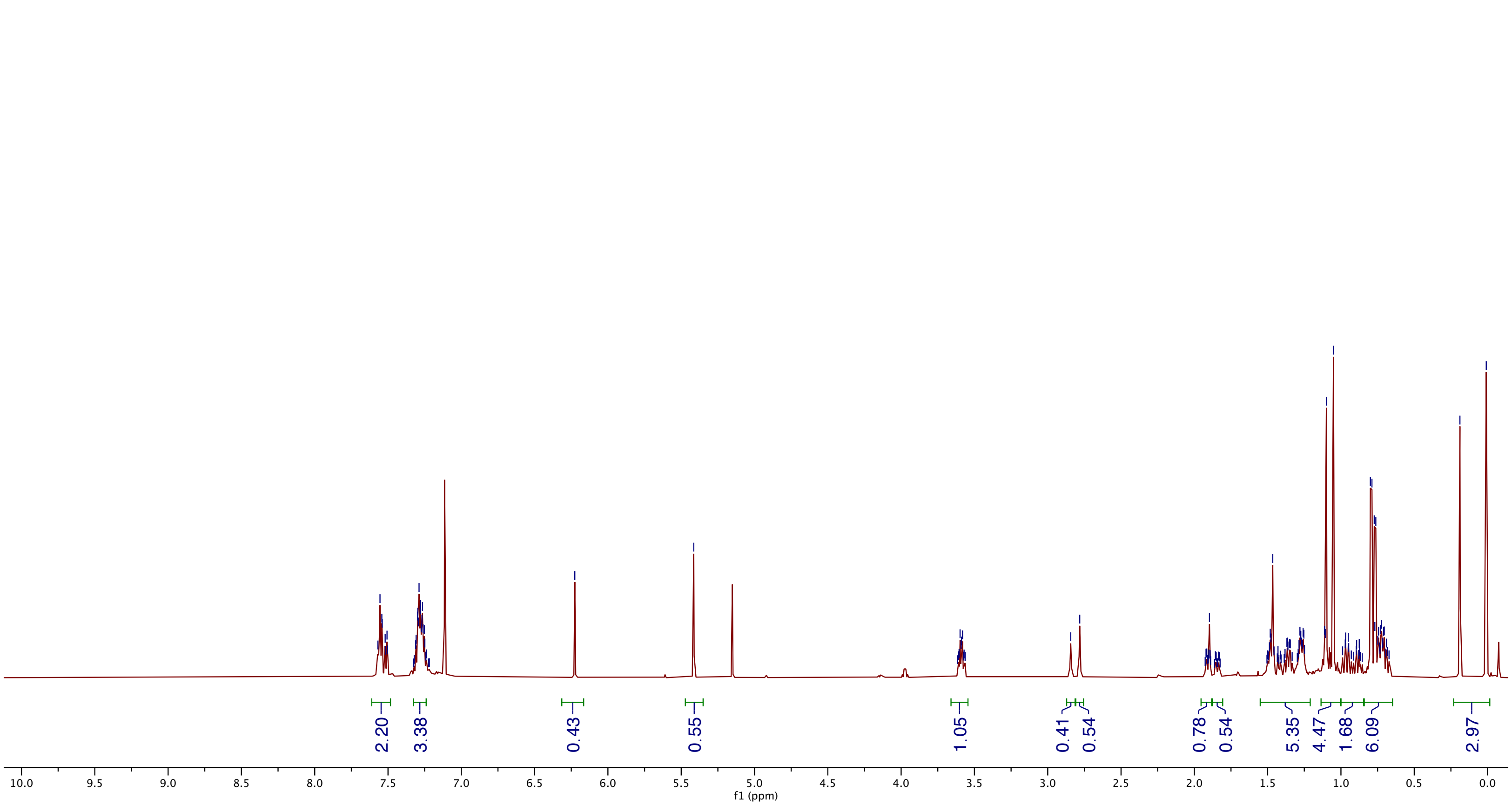

^{13}C NMR of 6f ($CDCl_3$, 126 MHz)

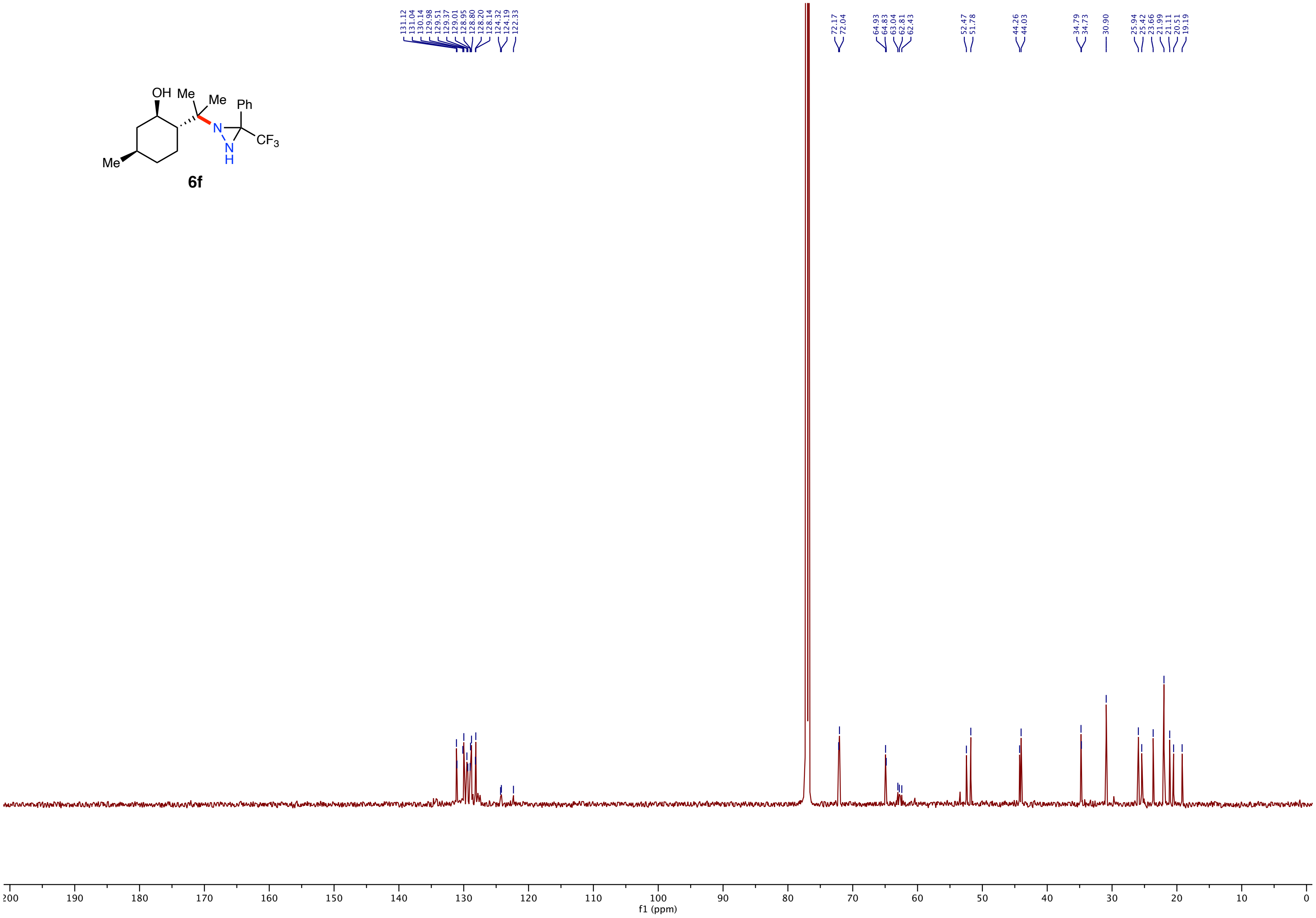

^{19}F NMR of 6f ($CDCl_3$, 471 MHz)

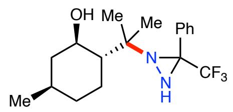

6f

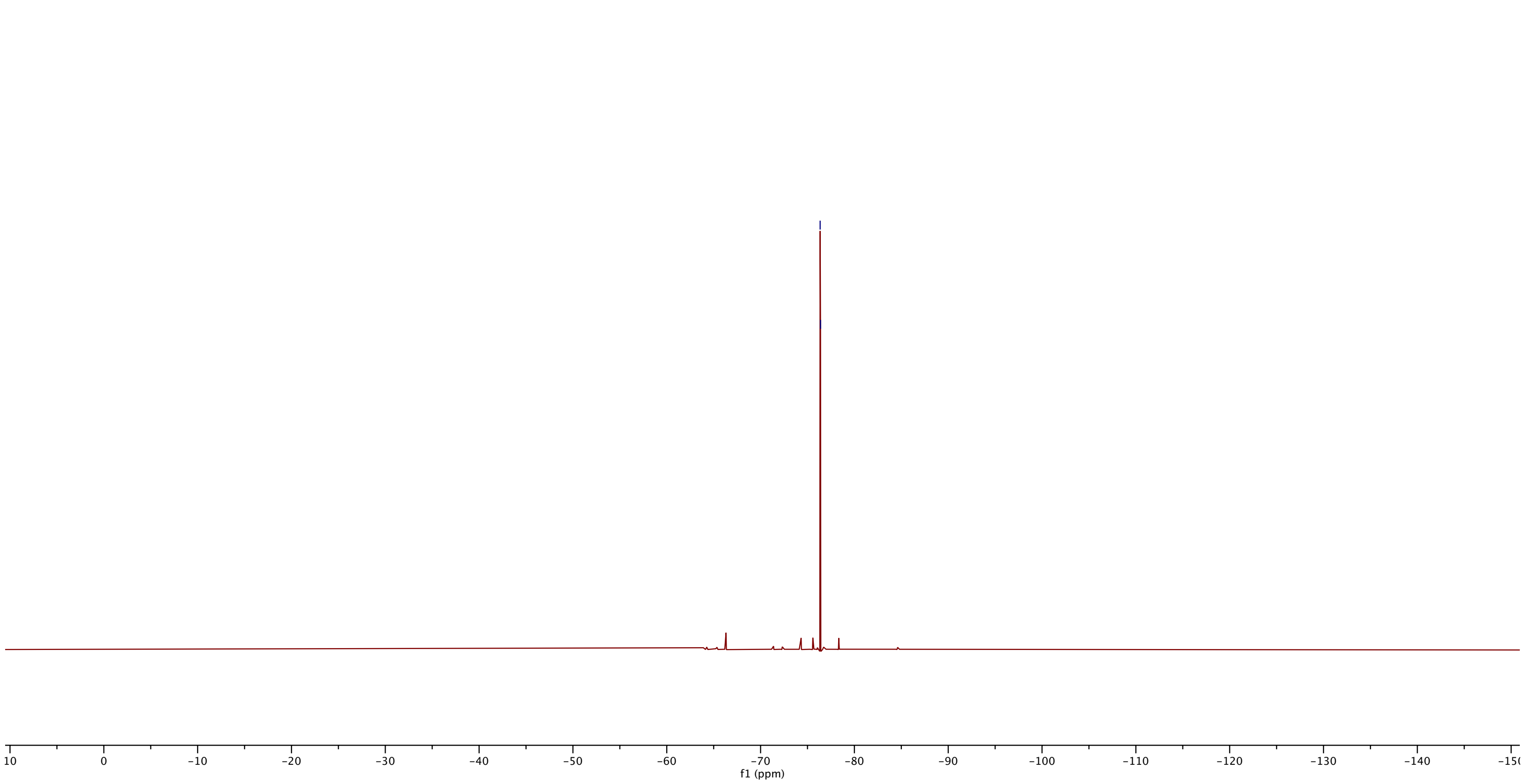

^{1}H NMR of 6g ($CDCl_3$, 500 MHz)

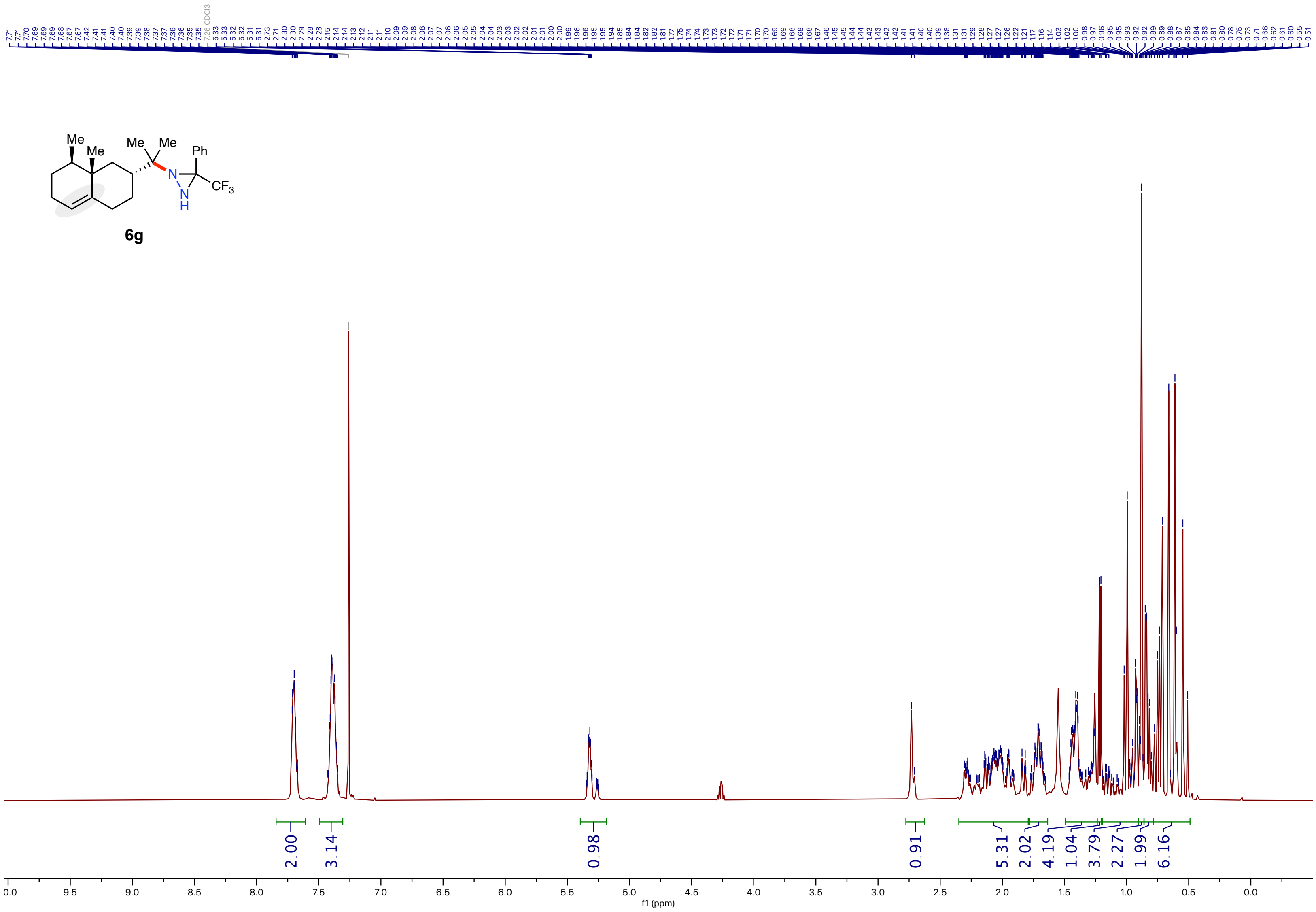

6g

^{13}C NMR of 6g ($CDCl_3$, 126 MHz)

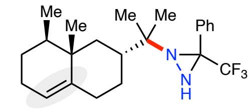

6g

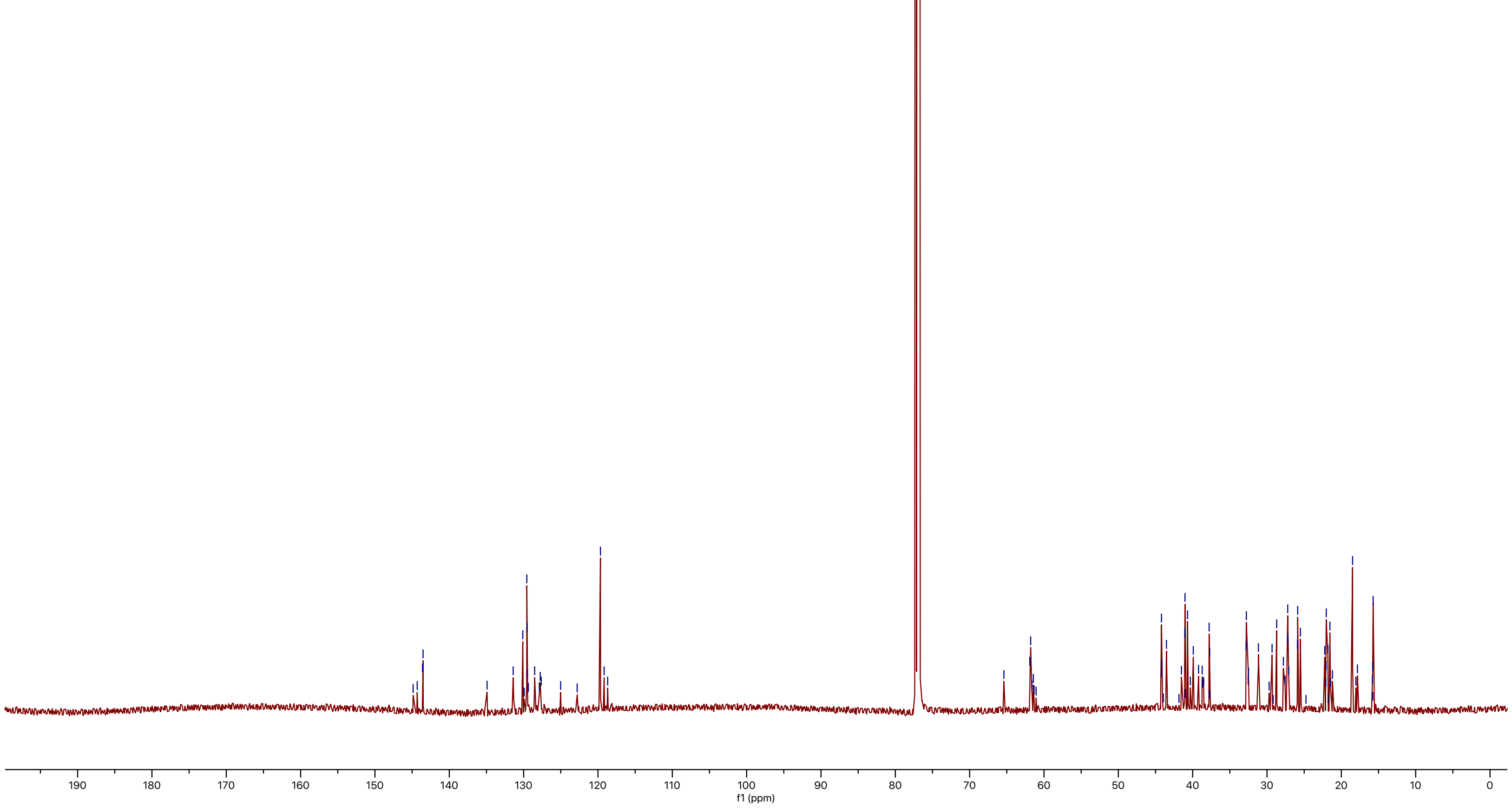

^{19}F NMR of 6g ($CDCl_3$, 126 MHz)

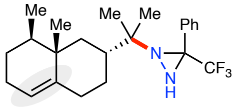

6g

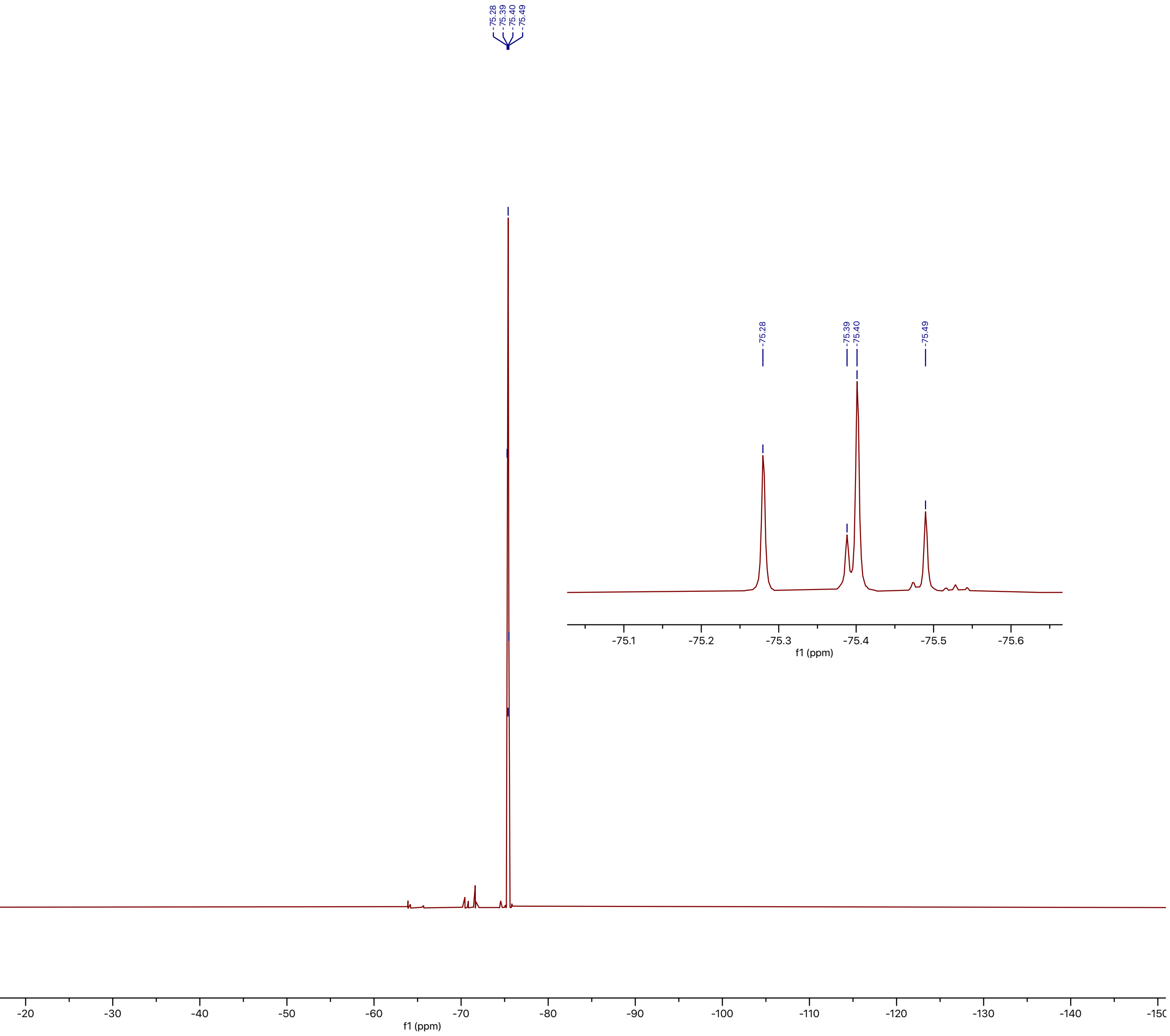

^{1}H NMR of 6h ($CDCl_3$, 500 MHz)

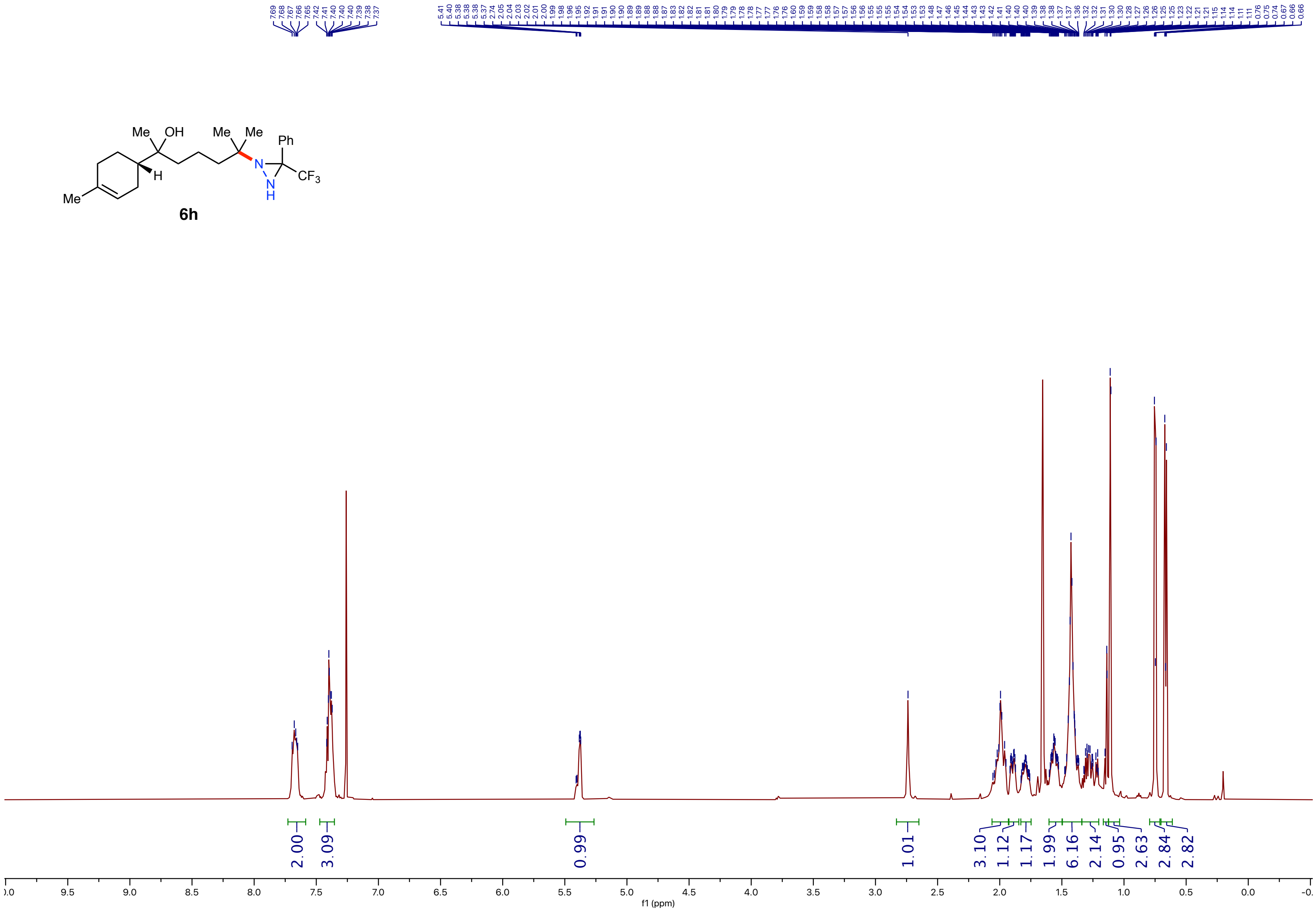

^{13}C NMR of 6h ($CDCl_3$, 126 MHz)

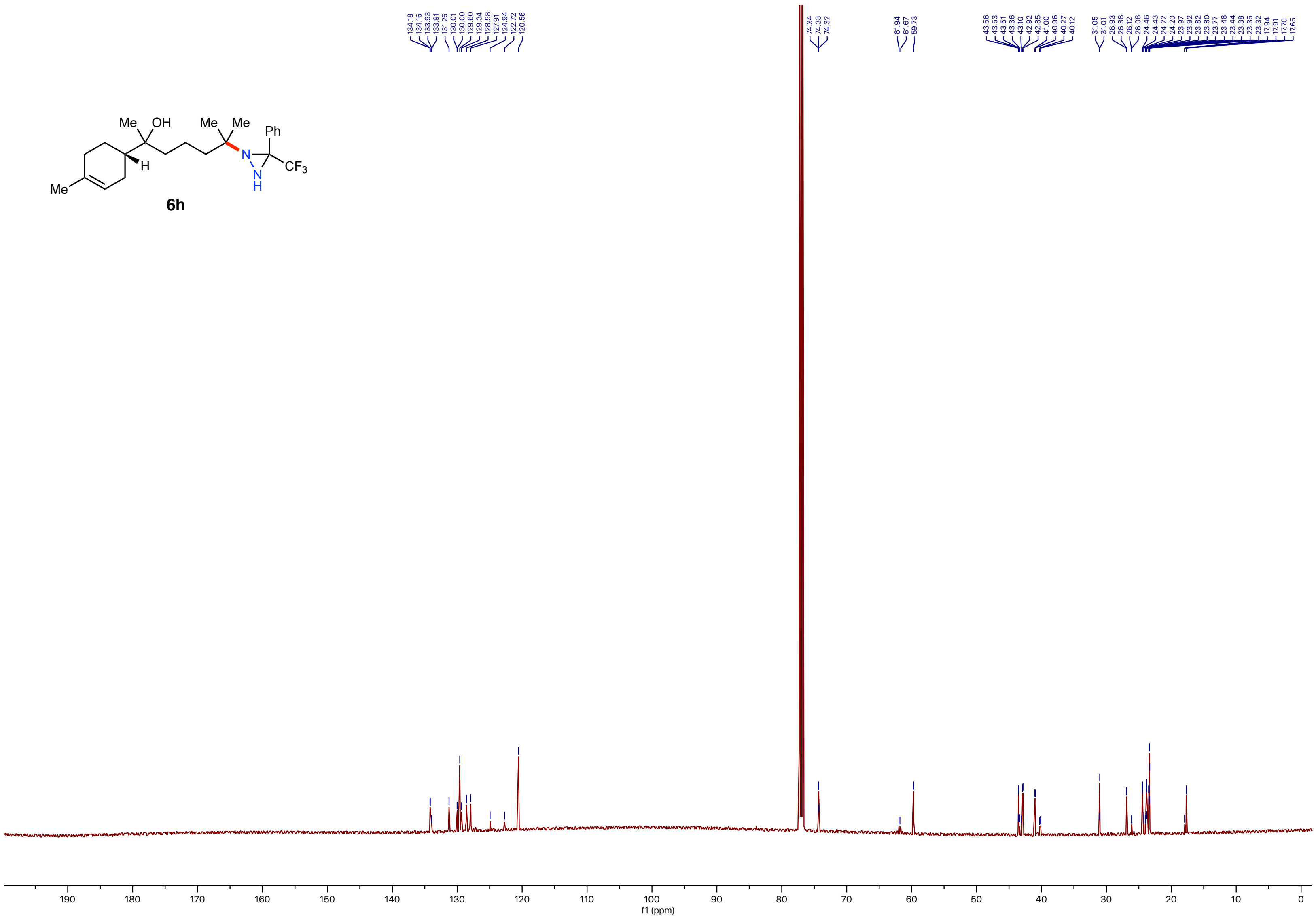

^{19}F NMR of 6h ($CDCl_3$, 471 MHz)

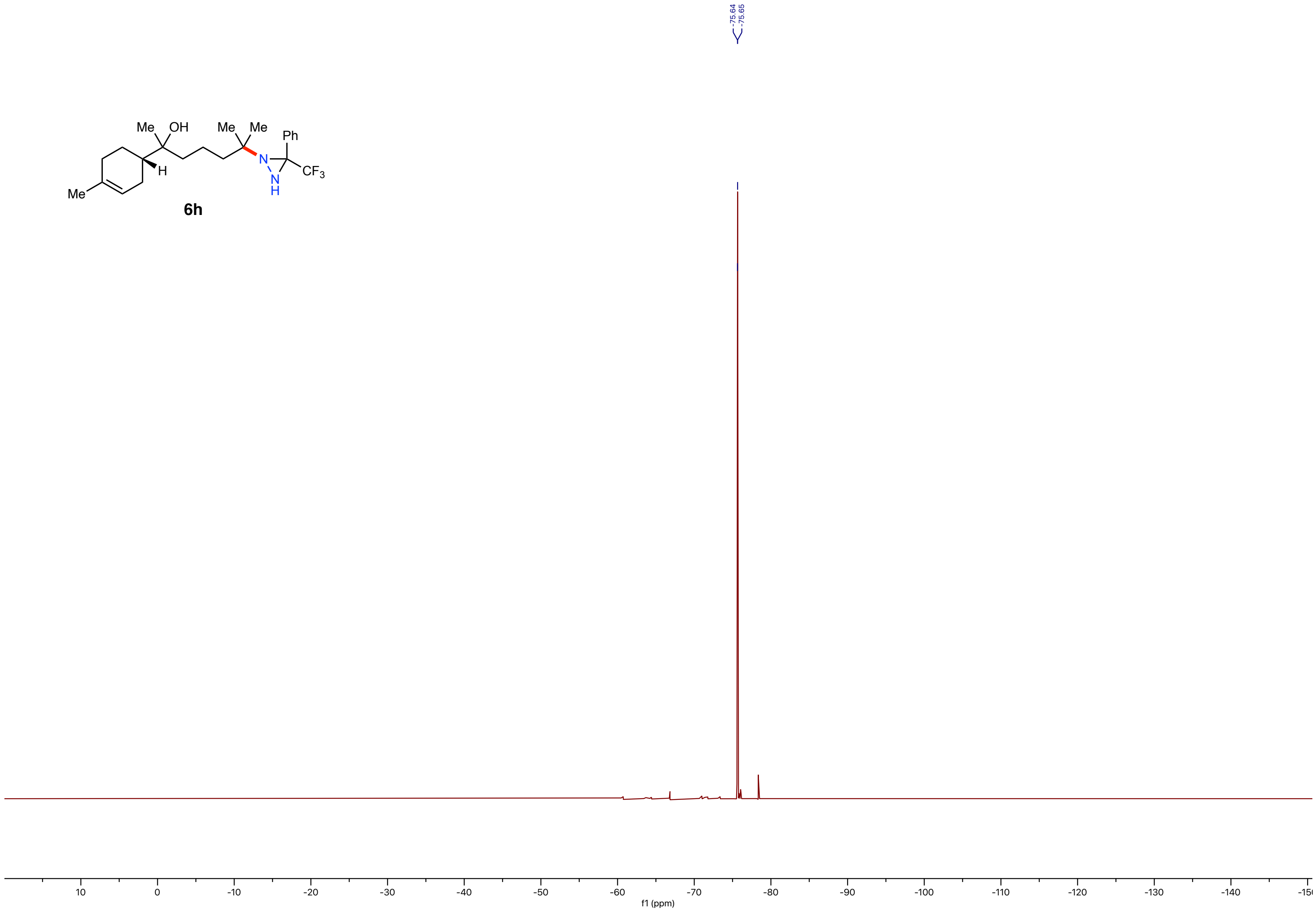

^{1}H NMR of 6i ($CDCl_3$, 500 MHz)

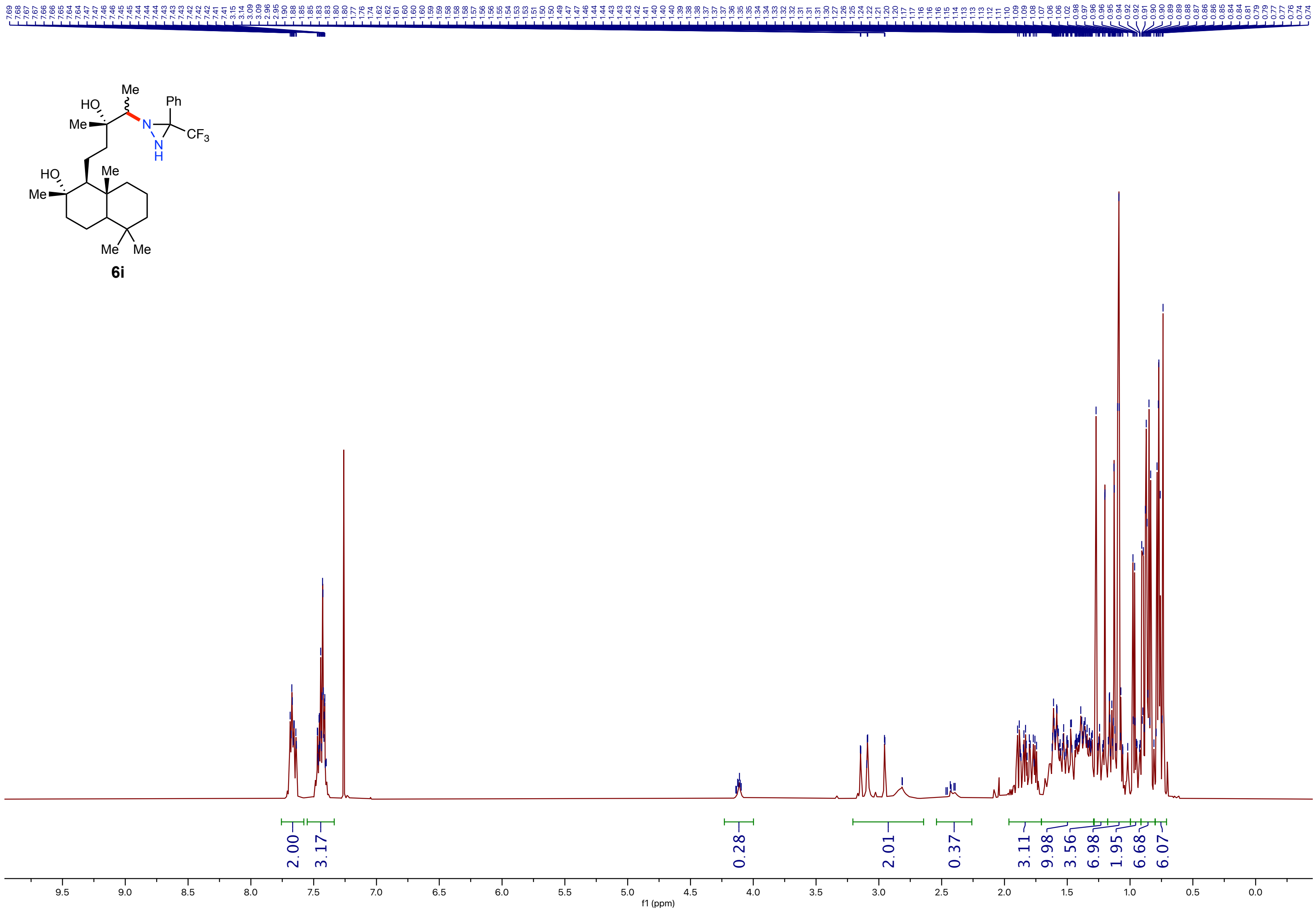

^{13}C NMR of 6i ($CDCl_3$, 126 MHz)

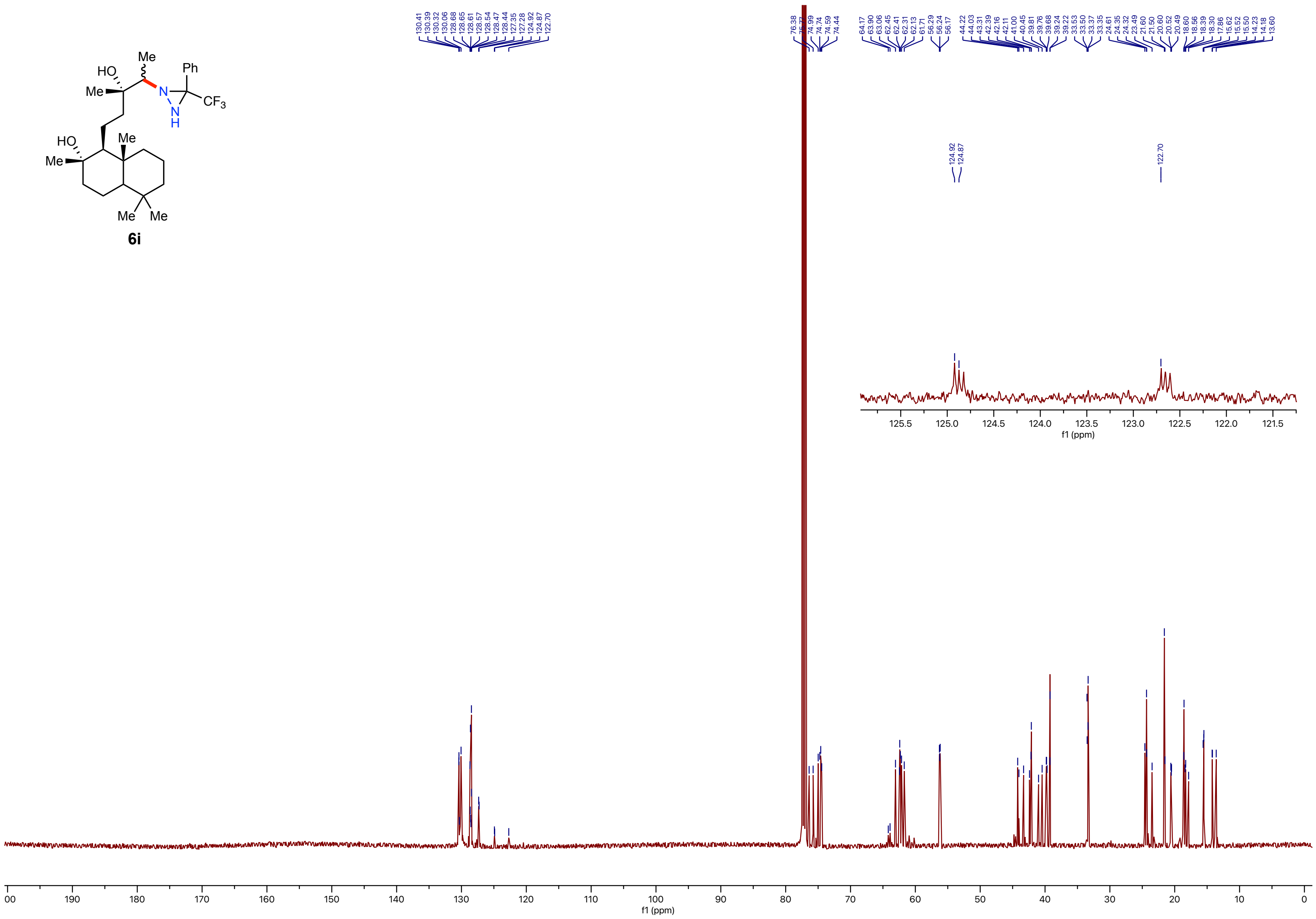

6i

^{19}F NMR of 6i ($CDCl_3$, 471 MHz)

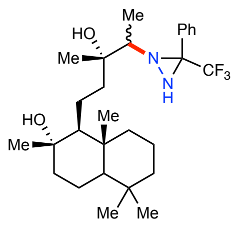

6i

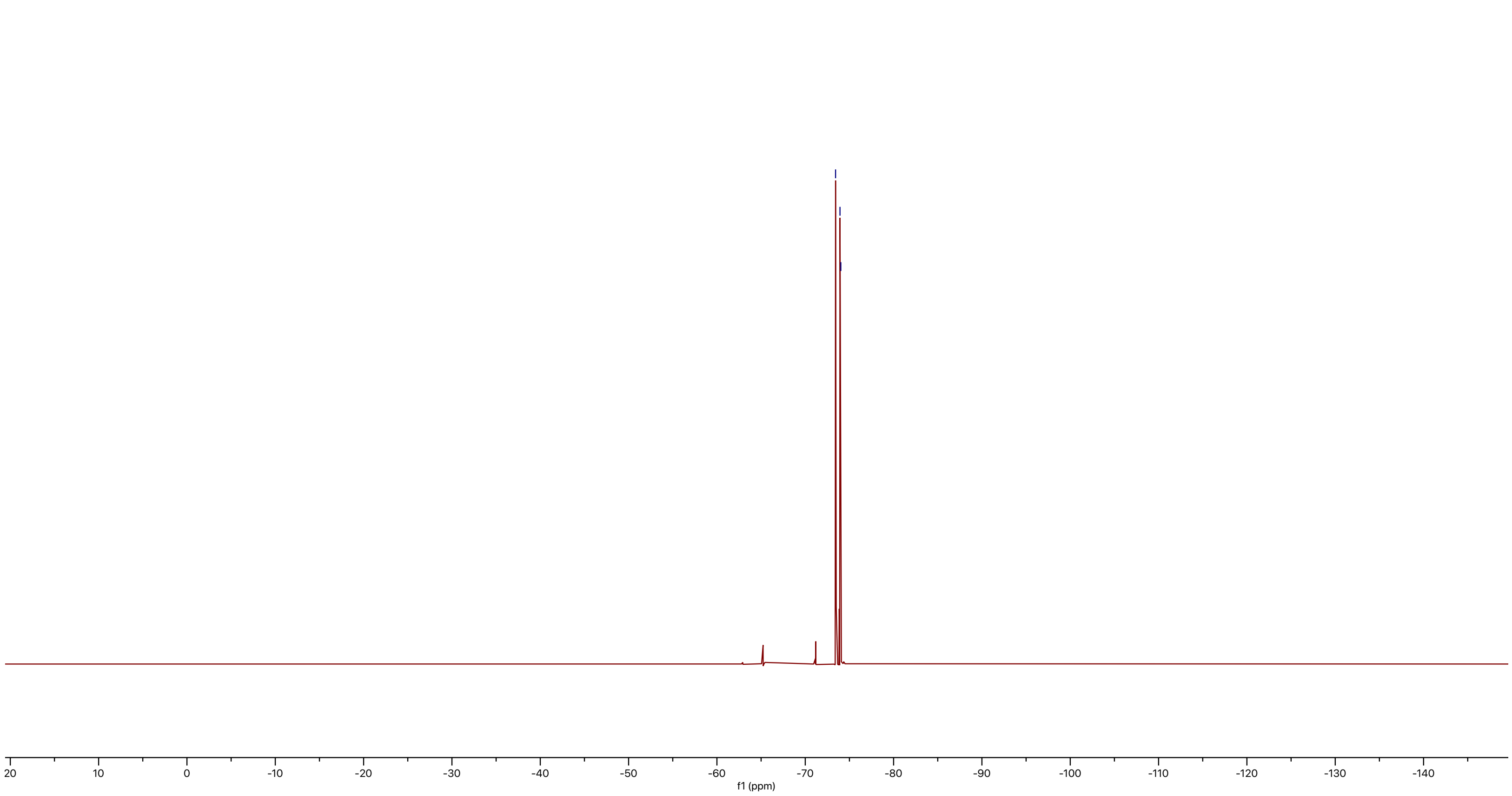

^{1}H NMR of 6j ($CDCl_3$, 500 MHz)

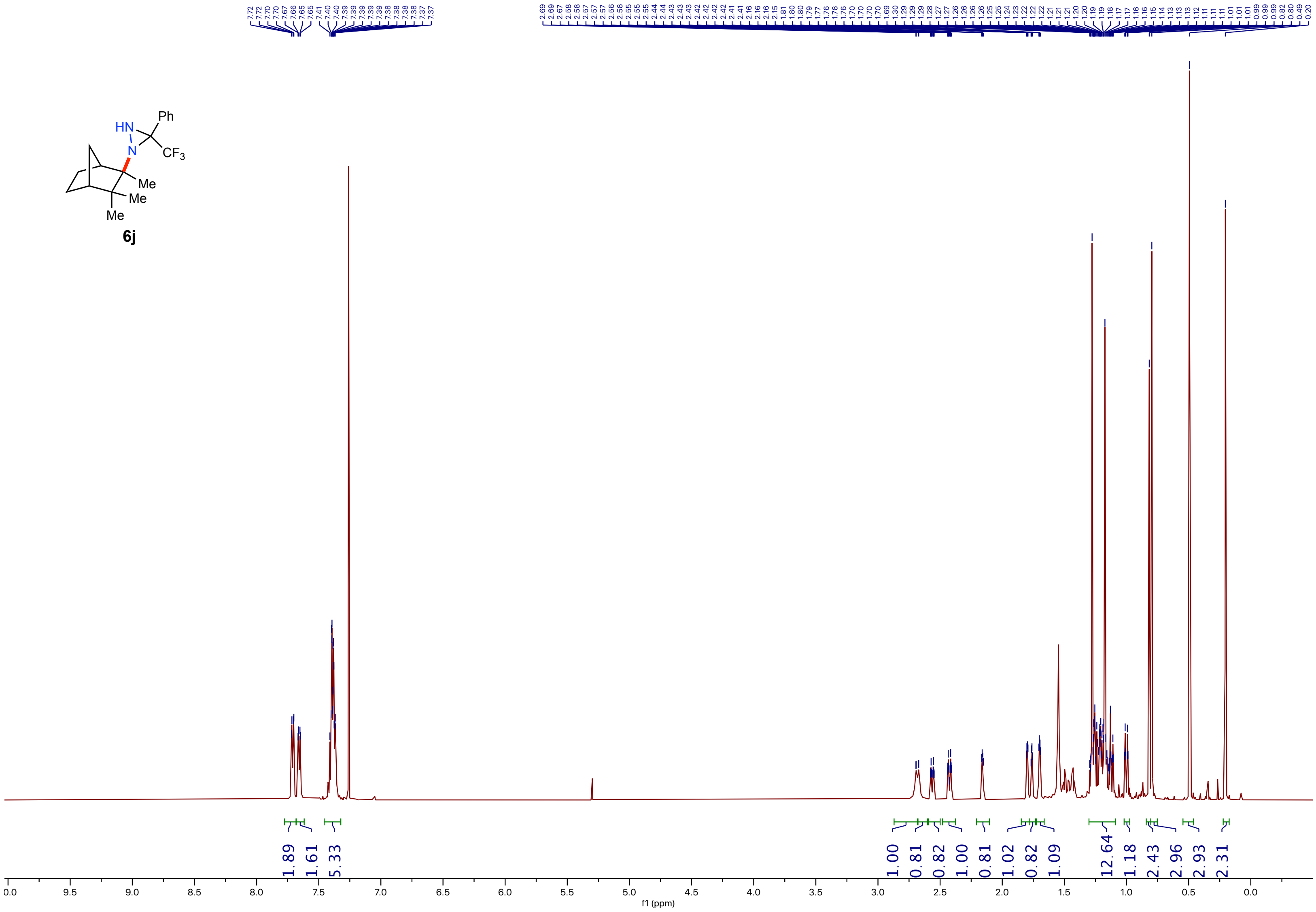

^{13}C NMR of 6j ($CDCl_3$, 126 MHz)

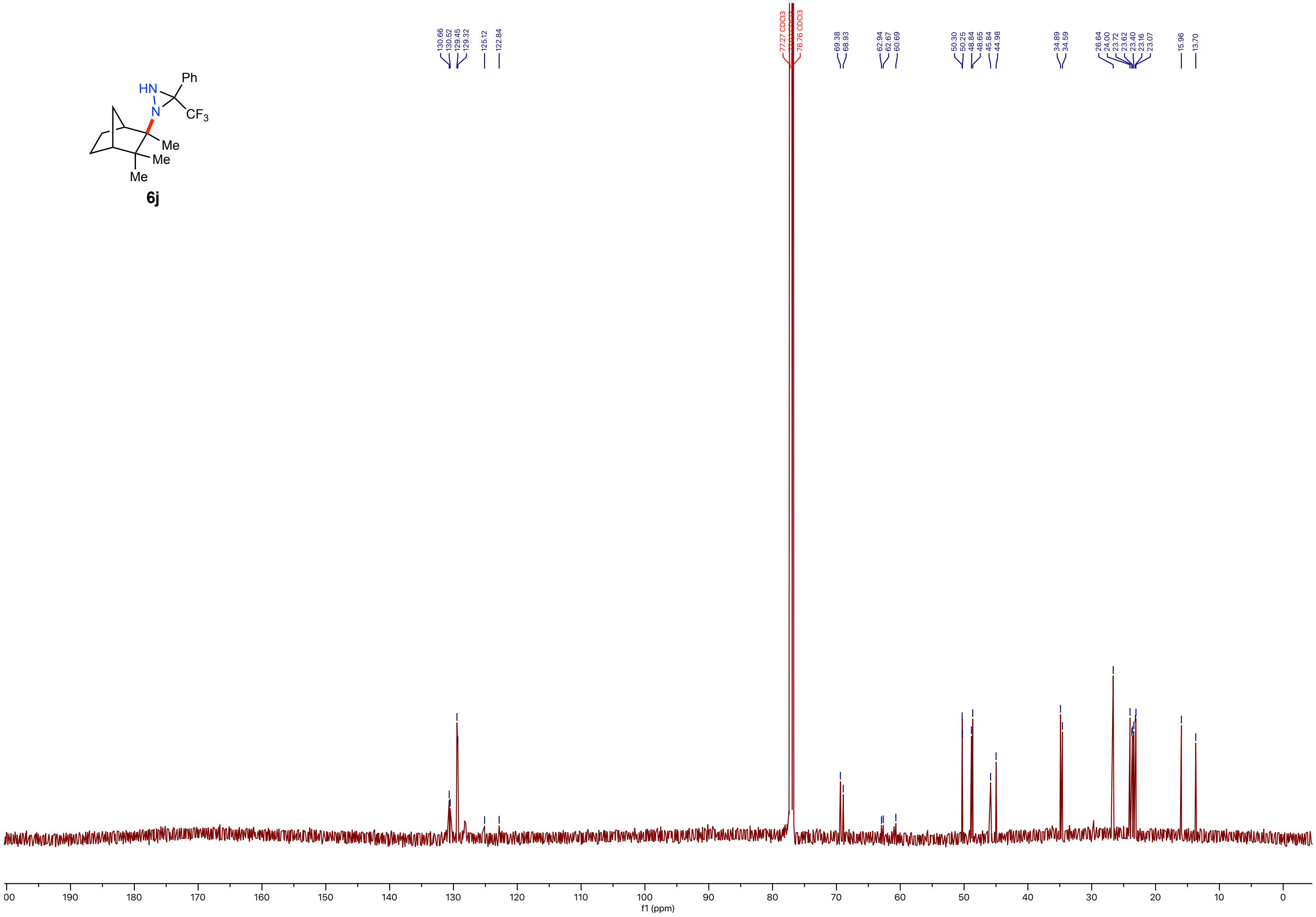

6j

^{19}F NMR of 6j ($CDCl_3$, 471 MHz)

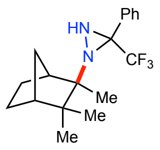

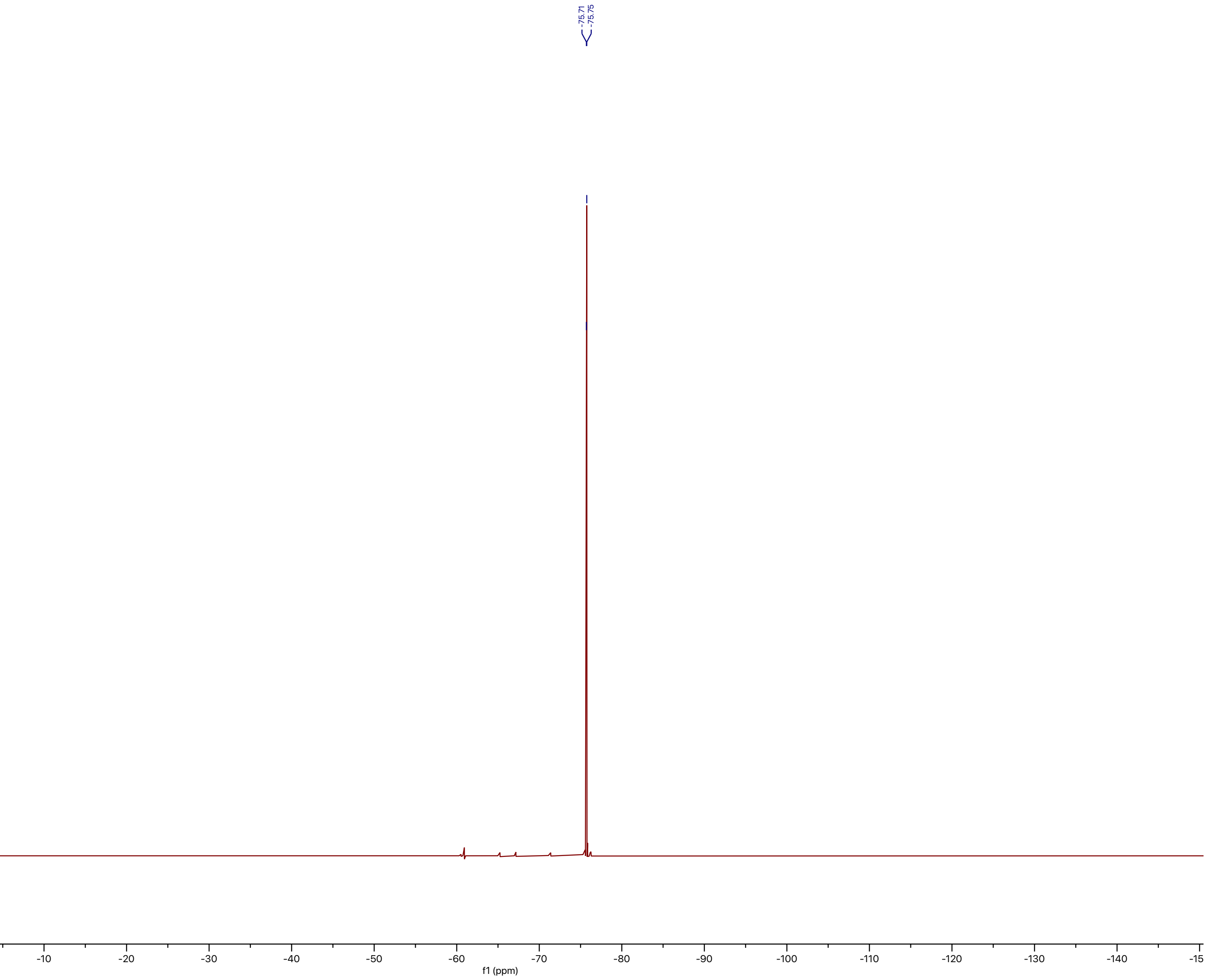

^{1}H NMR of 6k ($CDCl_3$, 500 MHz)

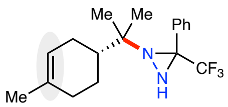

6k

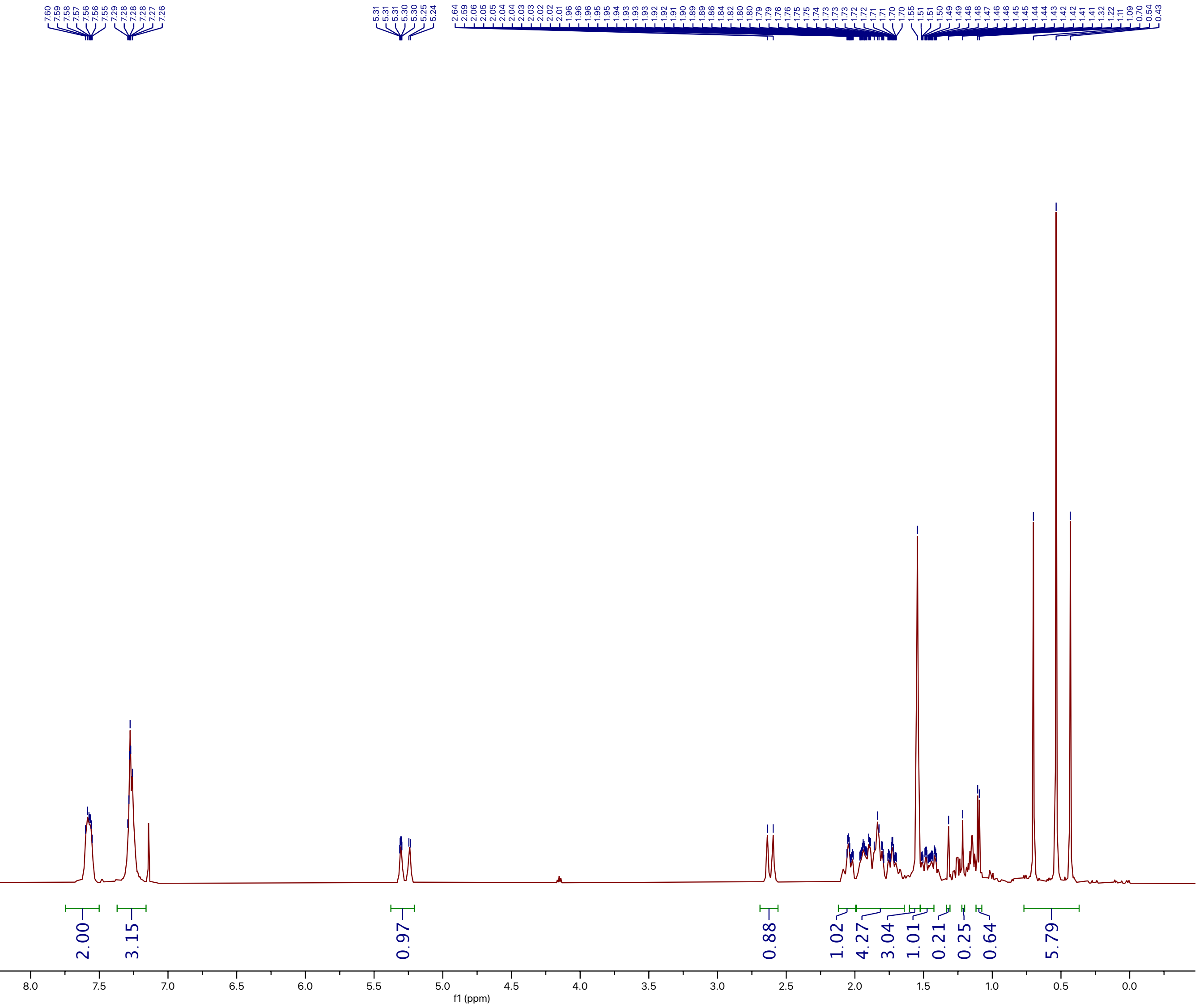

^{13}C NMR of 6k (CDCl$_3$, 126 MHz)

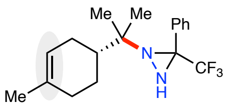

6k

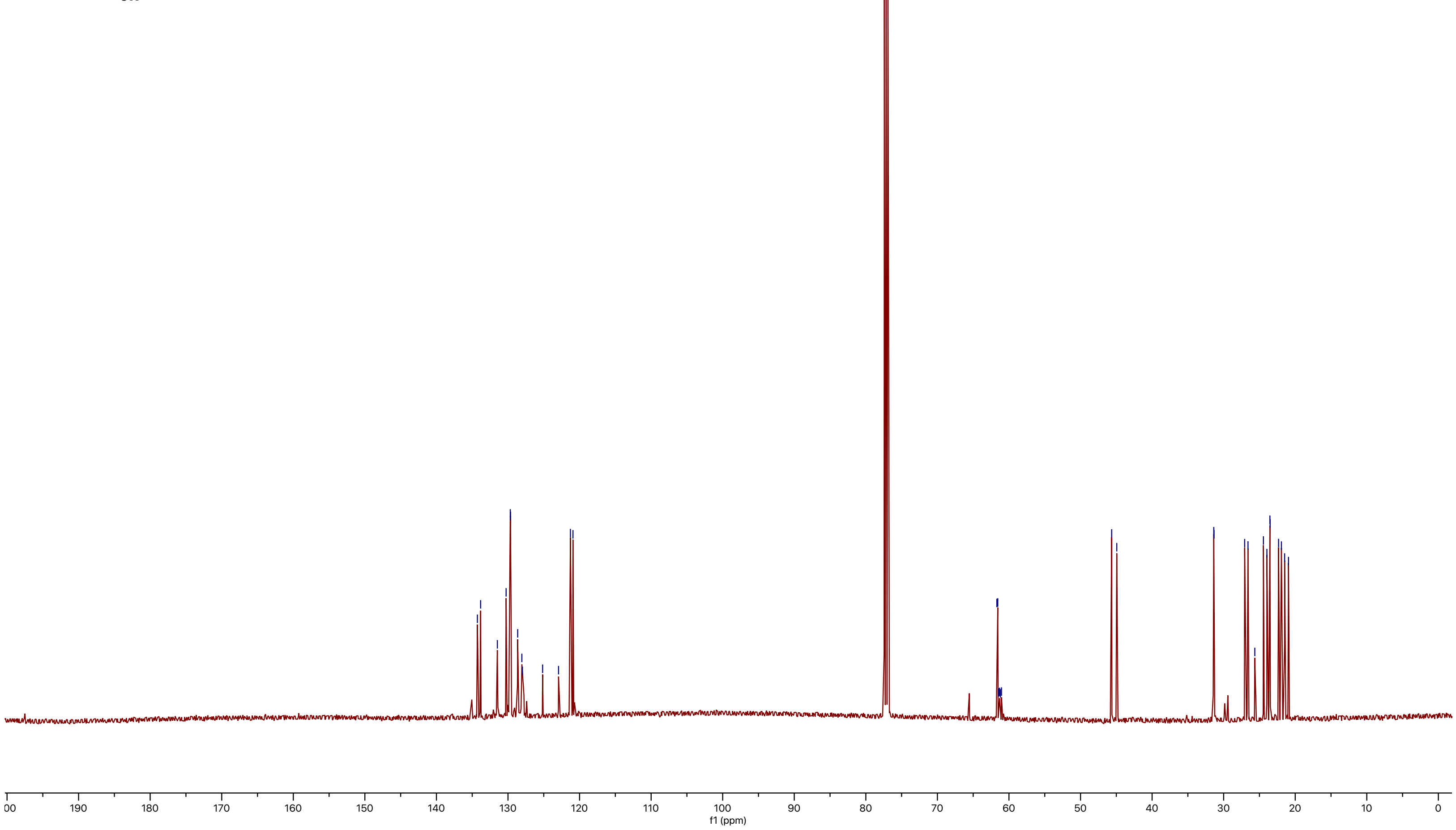

^{19}F NMR of 6k ($CDCl_3$, 471 MHz)

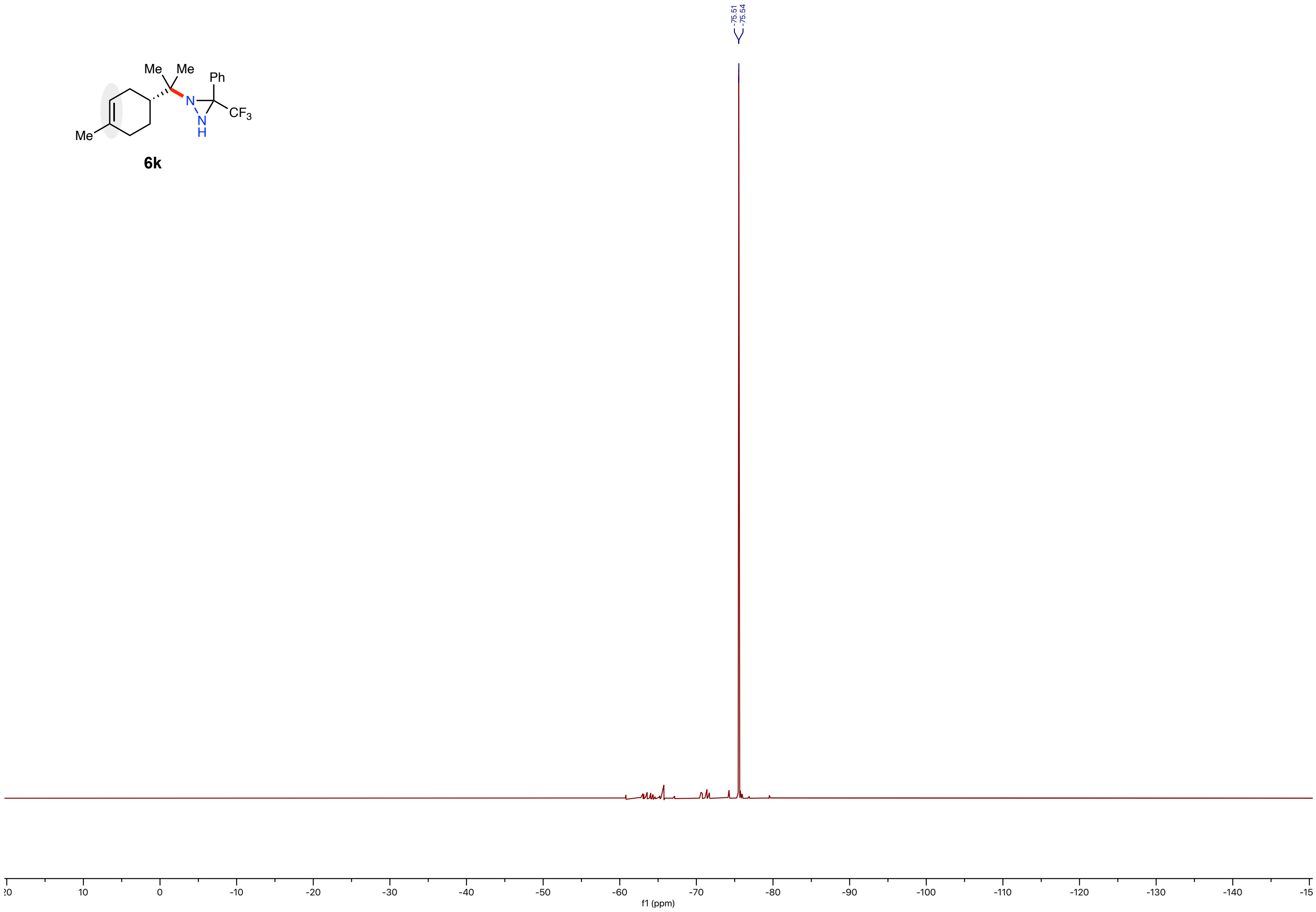

^{1}H NMR of 6l ($CDCl_3$, 500 MHz)

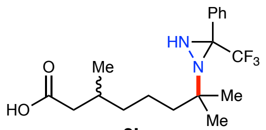

6l

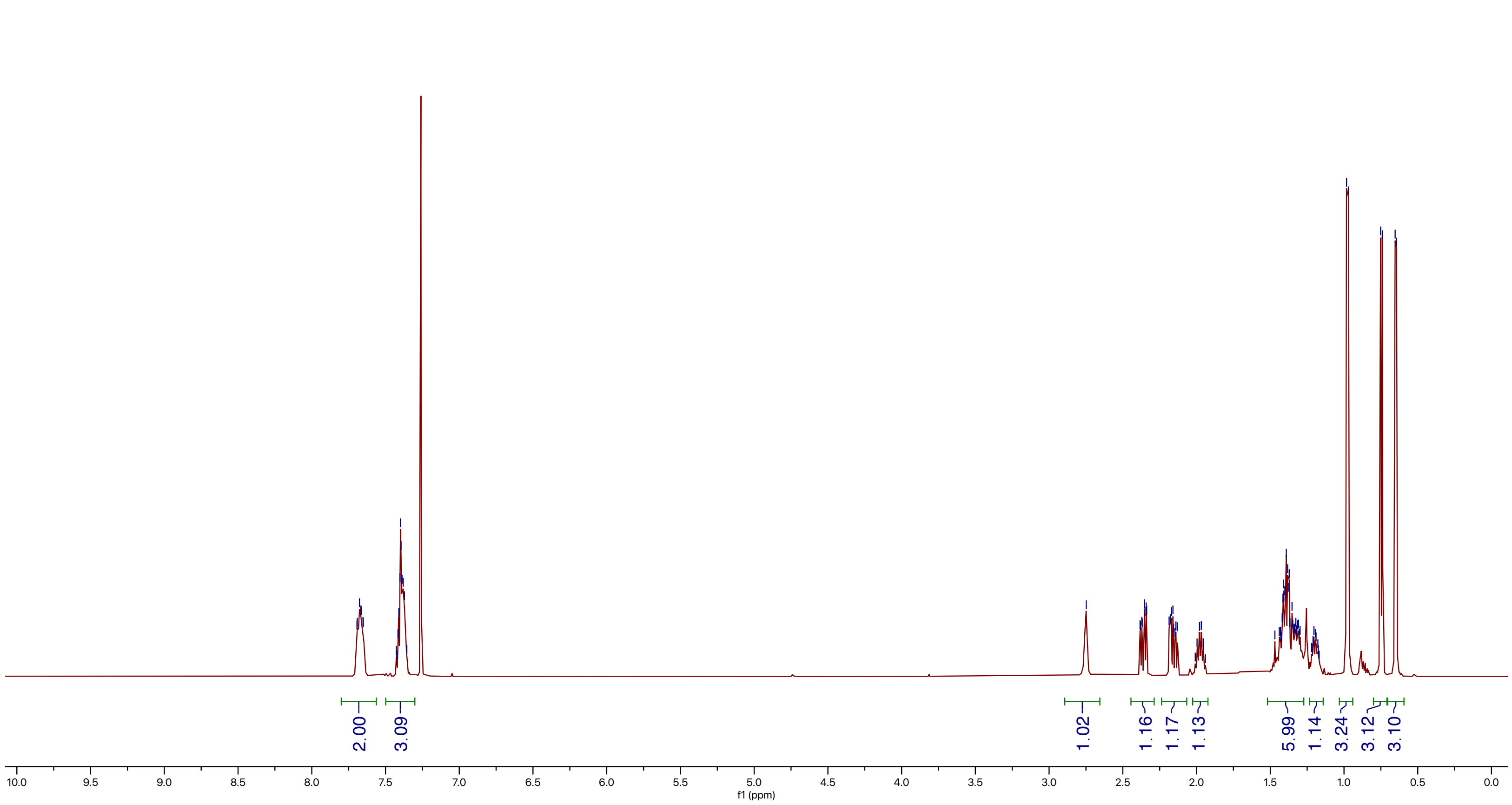

^{13}C NMR of 6l ($CDCl_3$, 126 MHz)

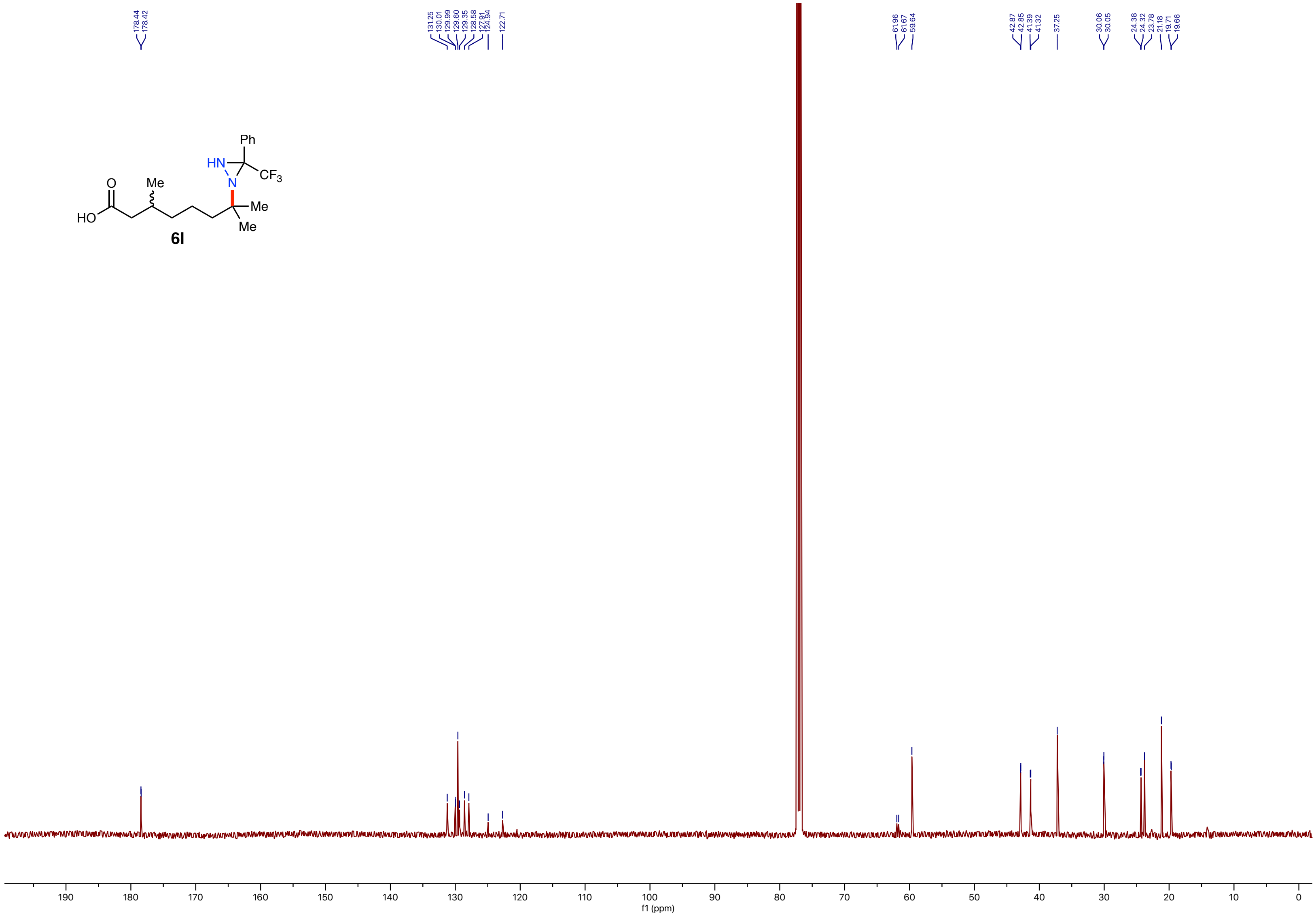

^{19}F NMR of 6l ($CDCl_3$, 471 MHz)

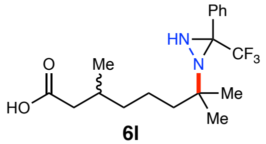

6l

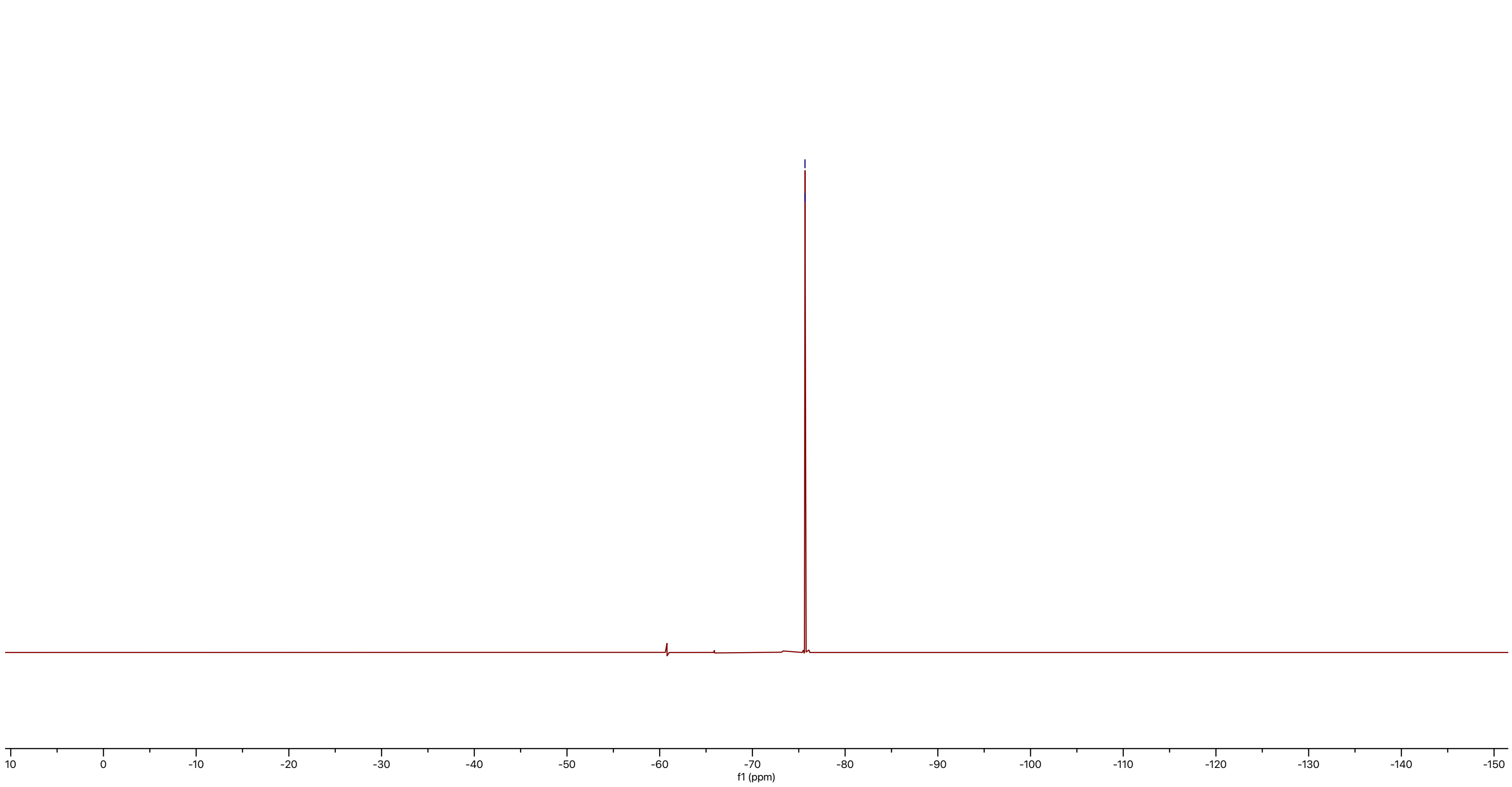

^{1}H NMR of 6m ($CDCl_3$, 500 MHz)

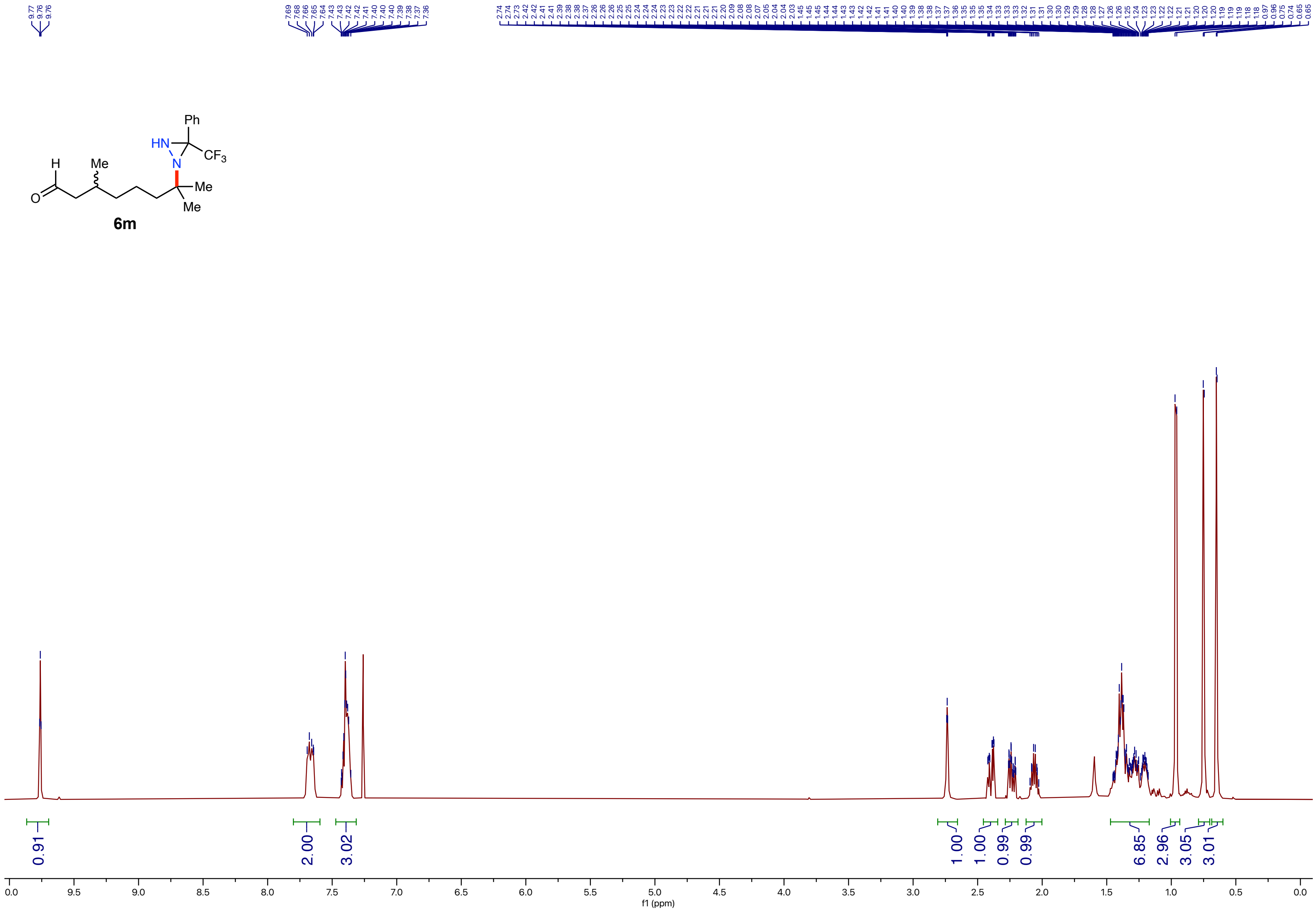

^{13}C NMR of 6m ($CDCl_3$, 126 MHz)

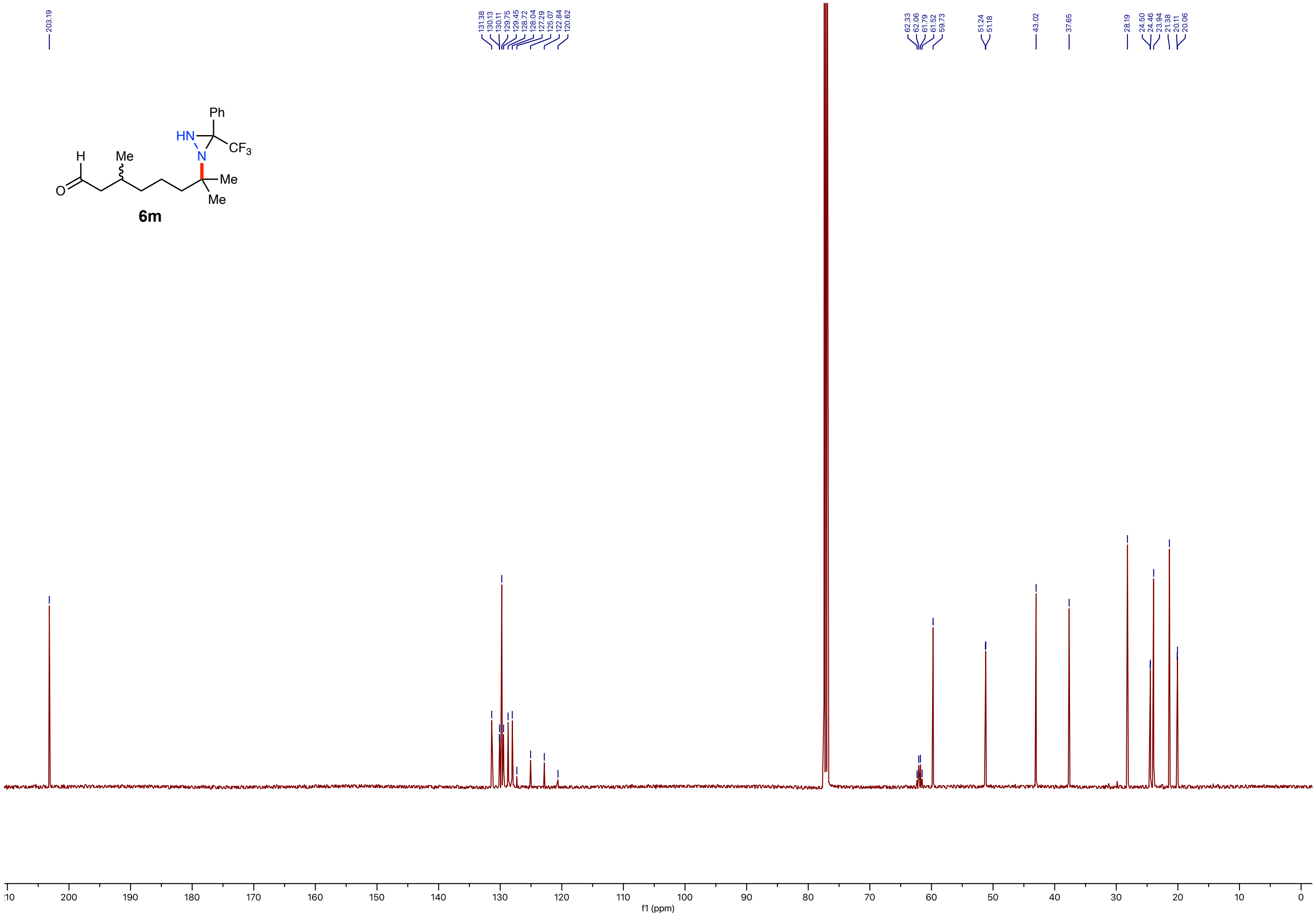

^{19}F NMR of 6m ($CDCl_3$, 471 MHz)

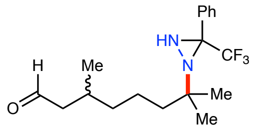

6m

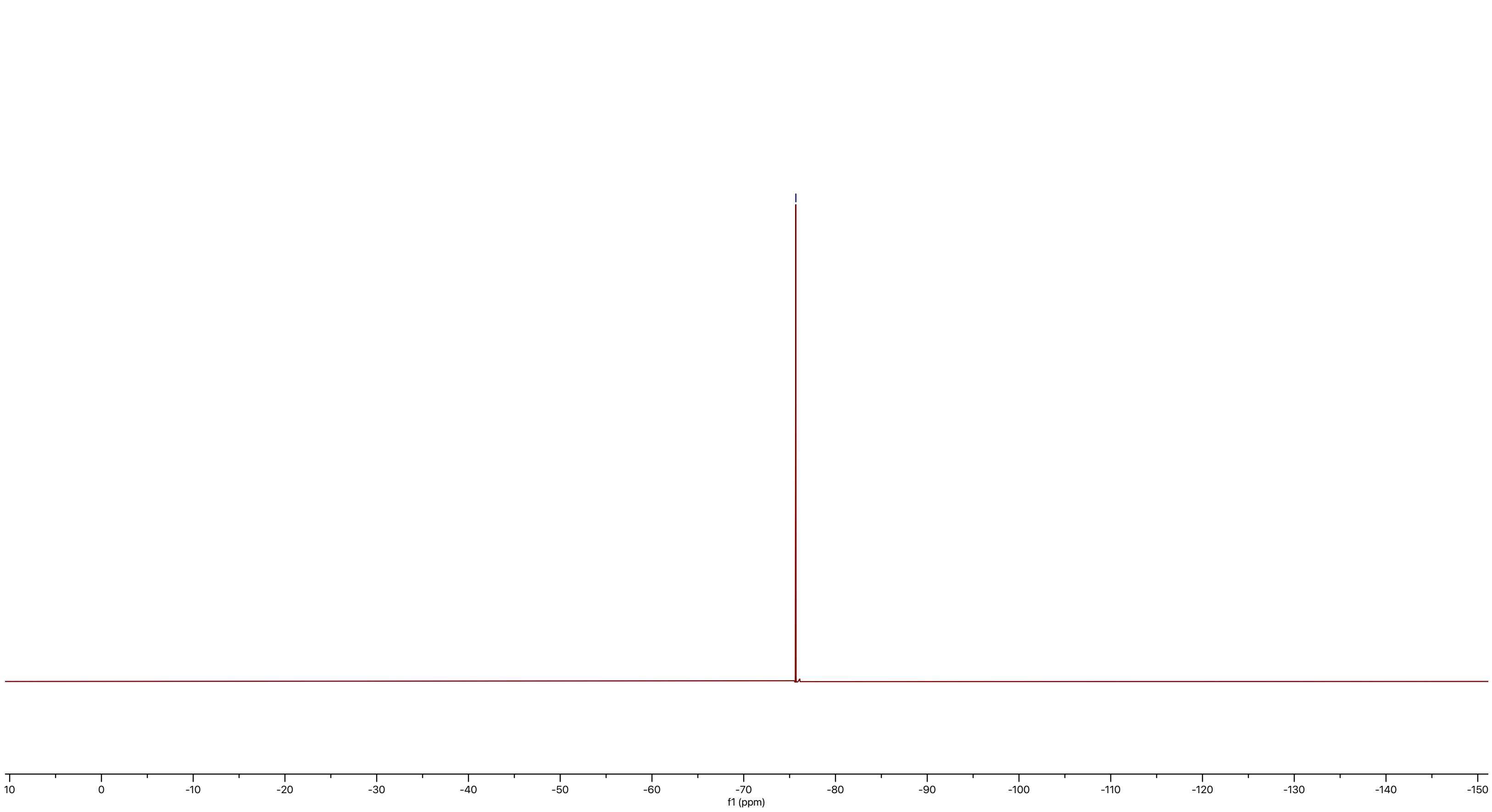

^{1}H NMR of 6n ($CDCl_3$, 500 MHz)

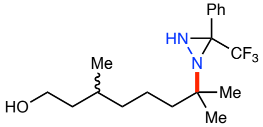

6n

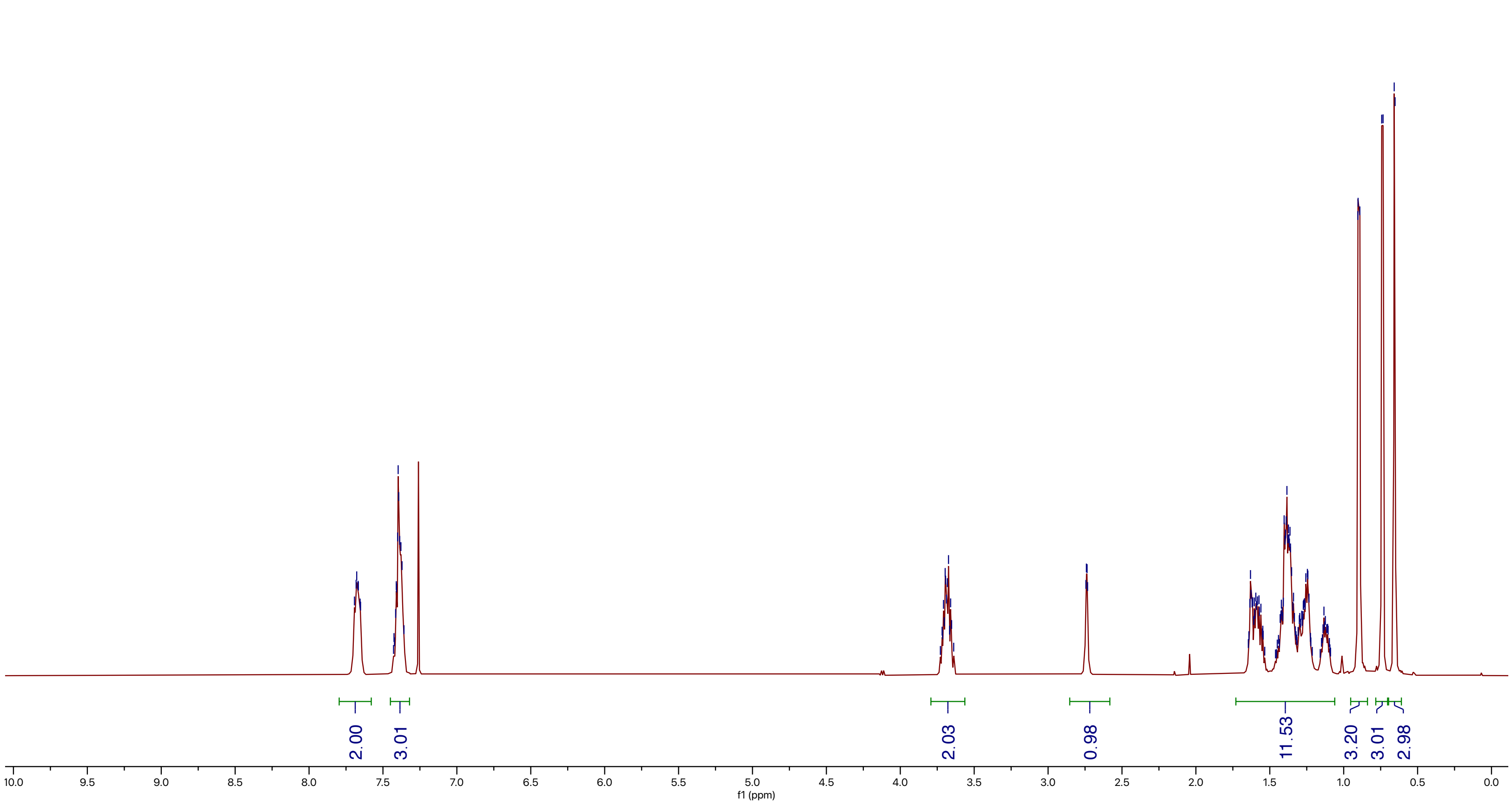

^{13}C NMR of 6n ($CDCl_3$, 126 MHz)

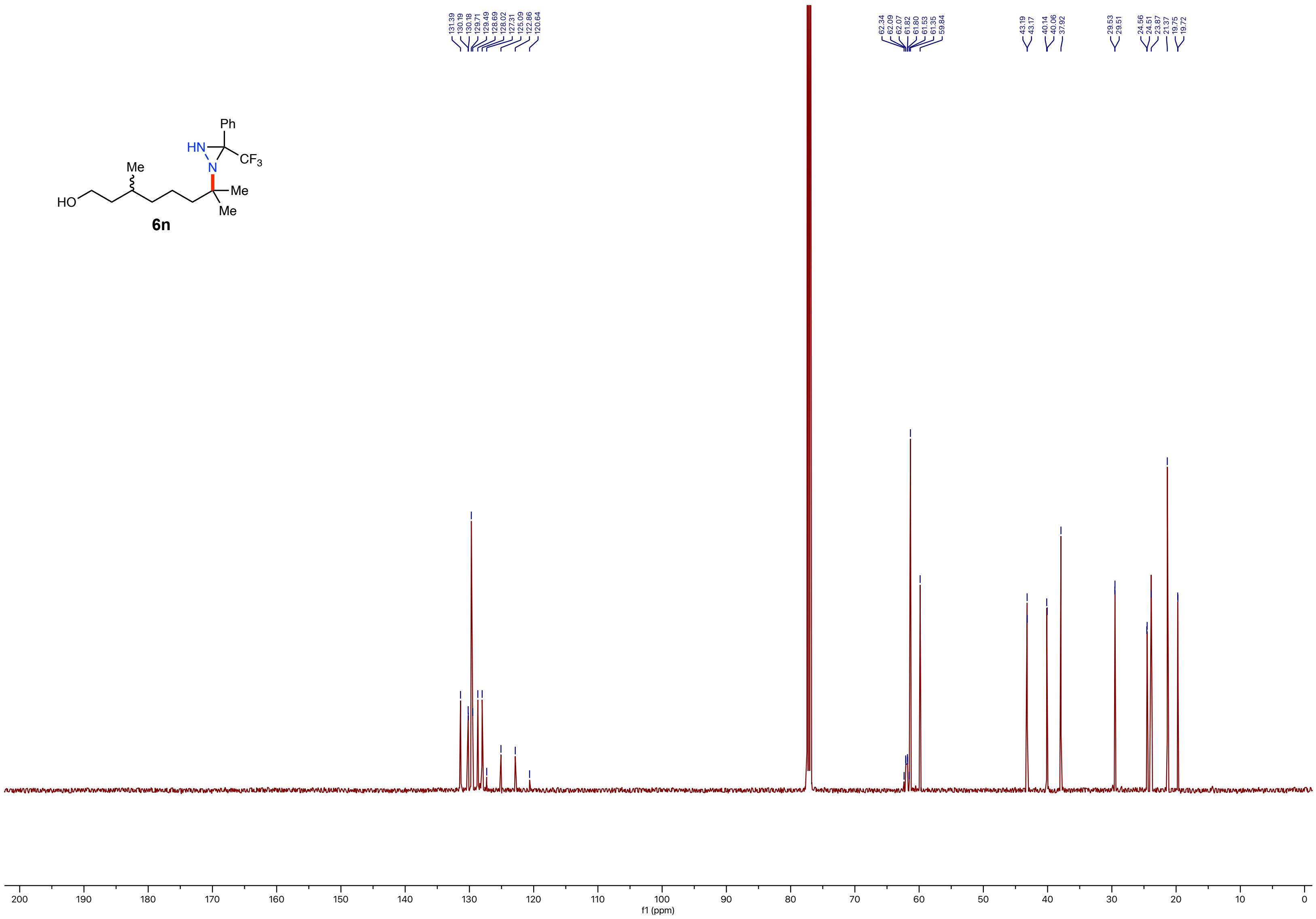

6n

^{19}F NMR of 6n ($CDCl_3$, 471 MHz)

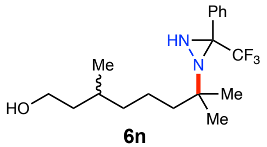

6n

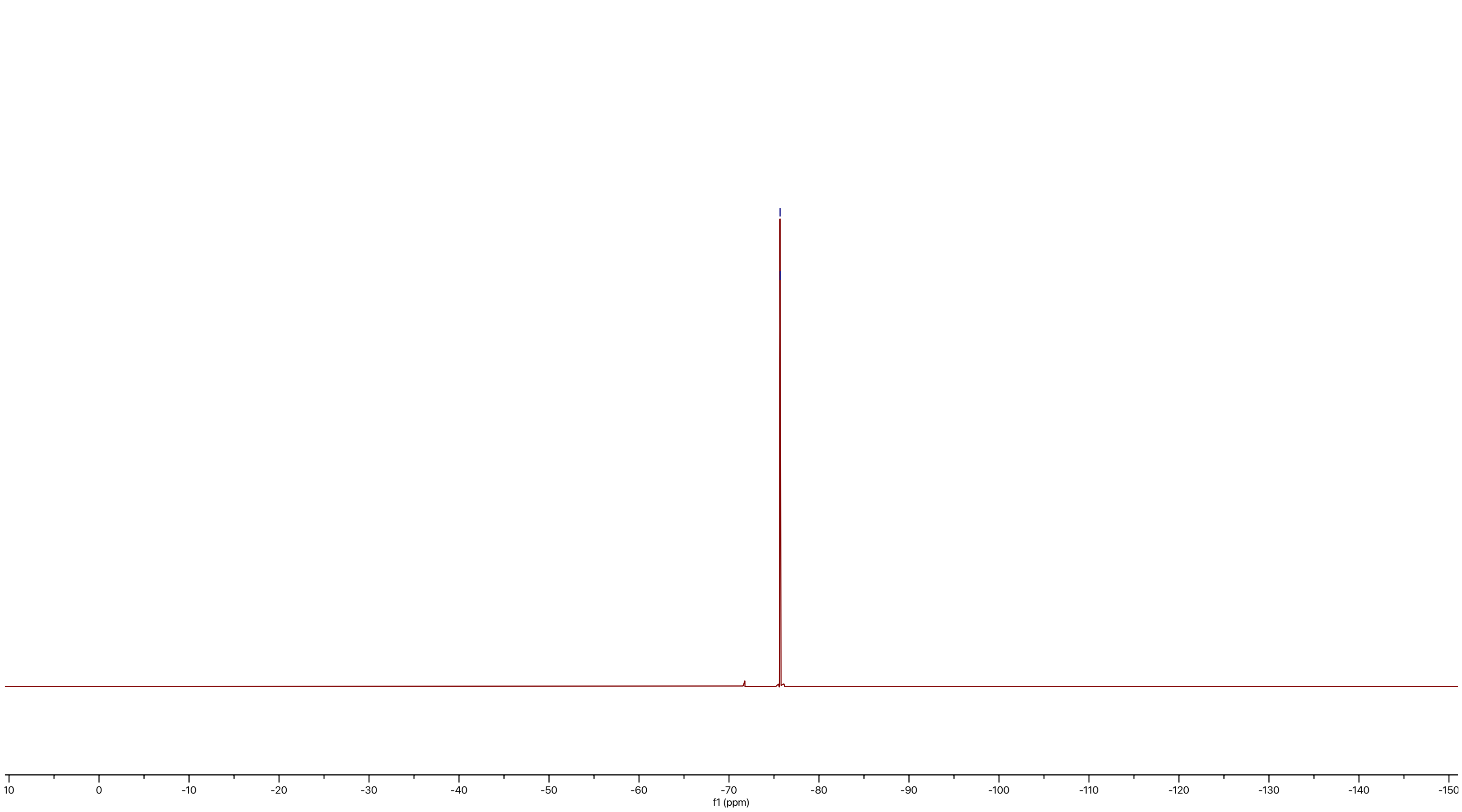

^{1}H NMR of *N*^{1}-(6-methoxyquinolin-8-yl)pentane-1,4-diamine hydrochloride (8) (DMSO, 500 MHz)

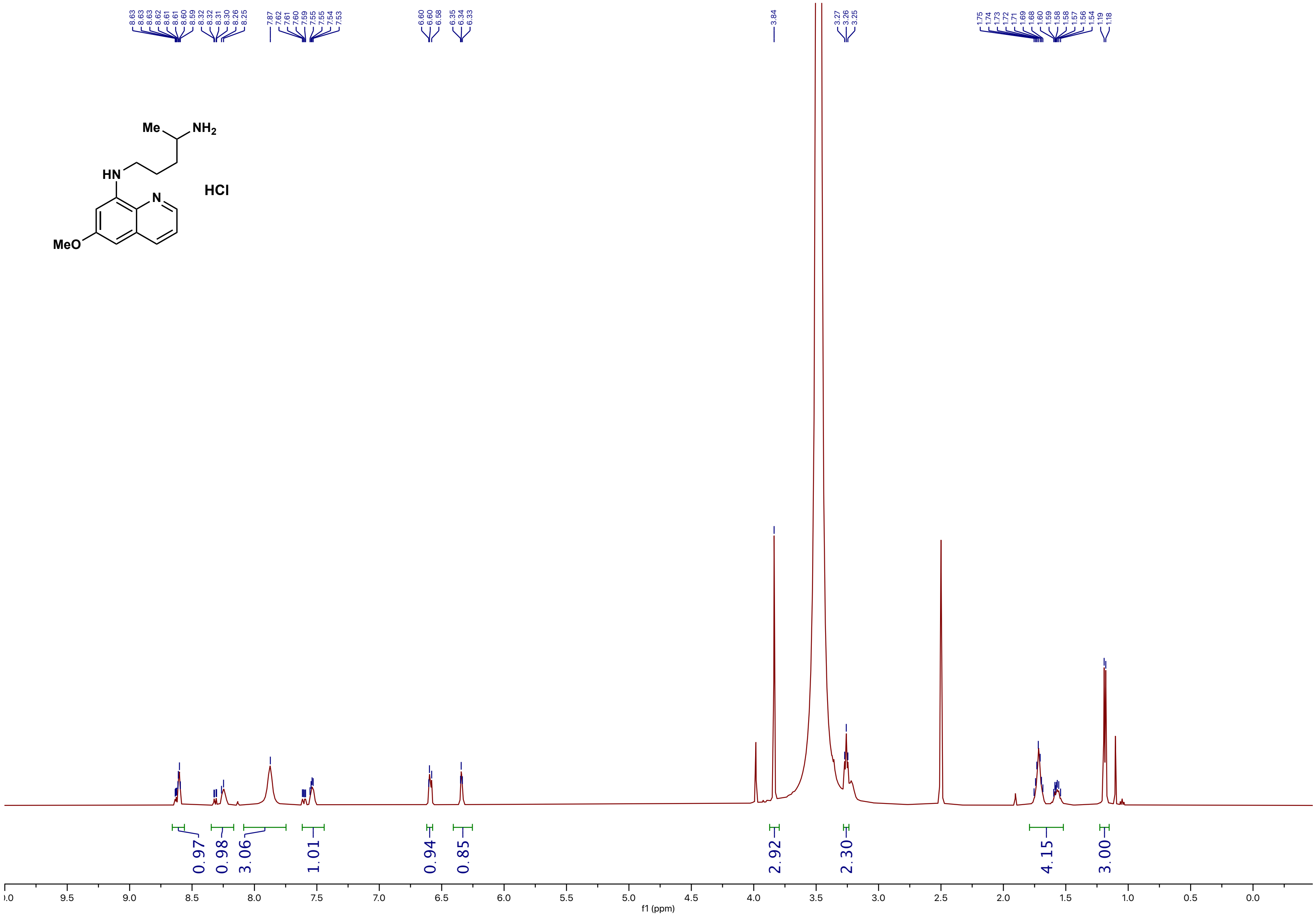

^{13}C NMR of *N*^{1}-(6-methoxyquinolin-8-yl)pentane-1,4-diamine hydrochloride (8) (DMSO, 126 MHz)

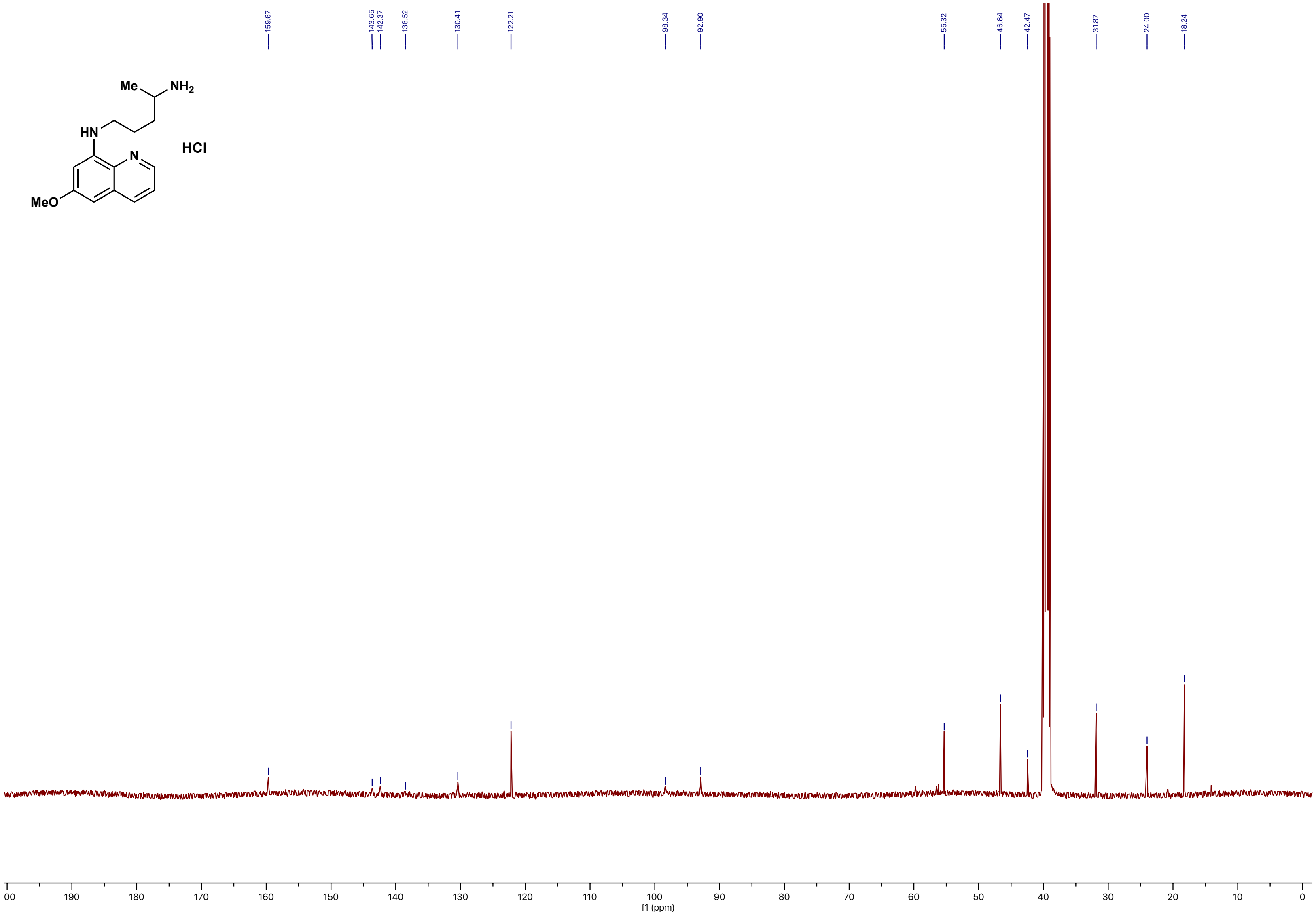

^{1}H NMR of (1*S*,2*R*,4*R*)-2,3,3-trimethylbicyclo[2.2.1]heptan-2-amine ($CDCl_3$, 500 MHz)

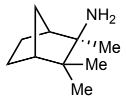

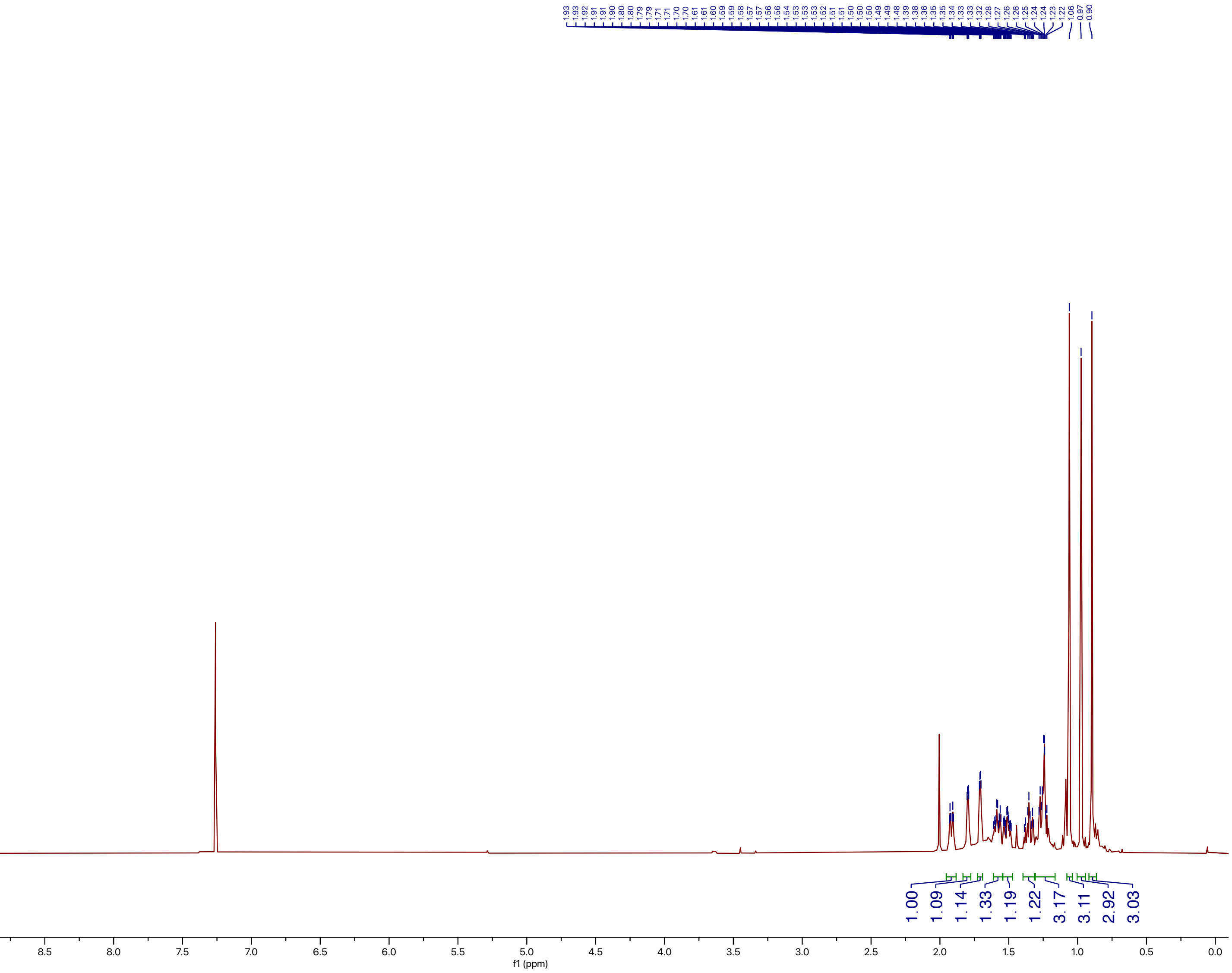

^{13}C NMR of (1*S*,2*R*,4*R*)-2,3,3-trimethylbicyclo[2.2.1]heptan-2-amine ($CDCl_3$, 126 MHz)

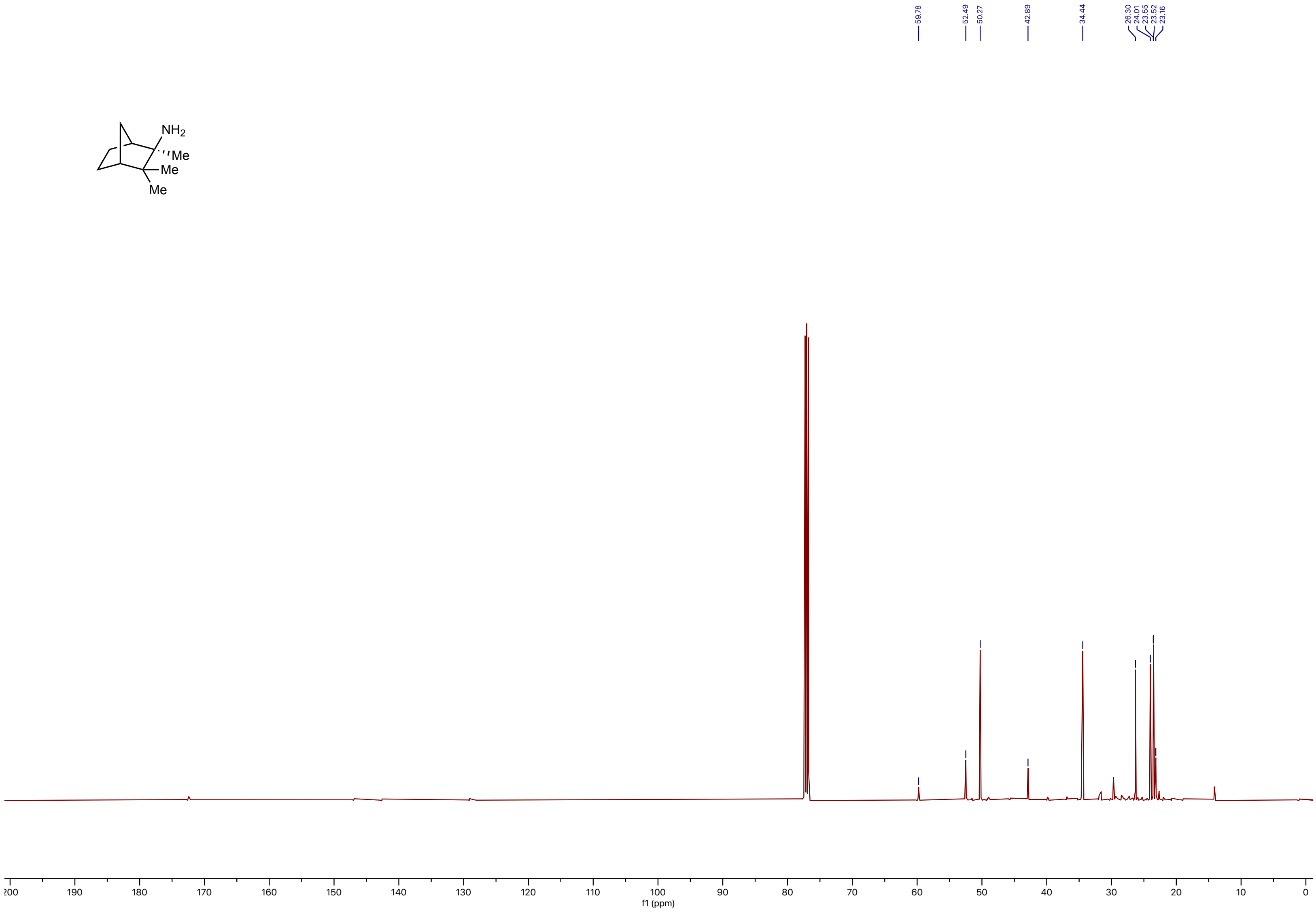

^{1}H NMR of Mecamylamine HCl (10) ($CDCl_3$, 500 MHz)

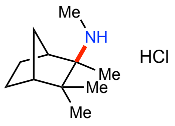

10

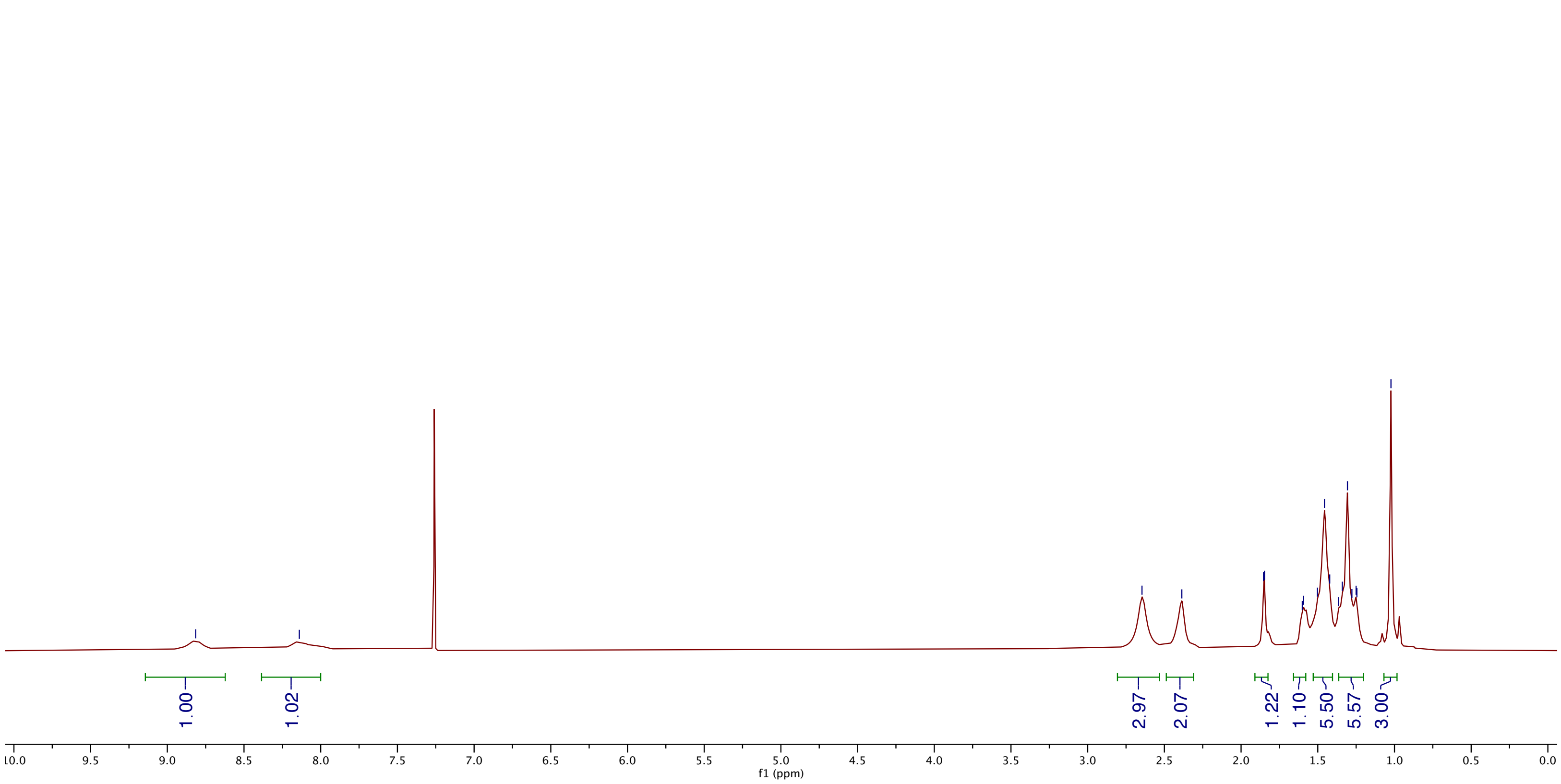

^{13}C NMR of Mecamylamine HCl (10) ($CDCl_3$, 126 MHz)

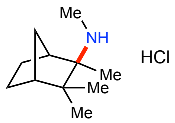

10

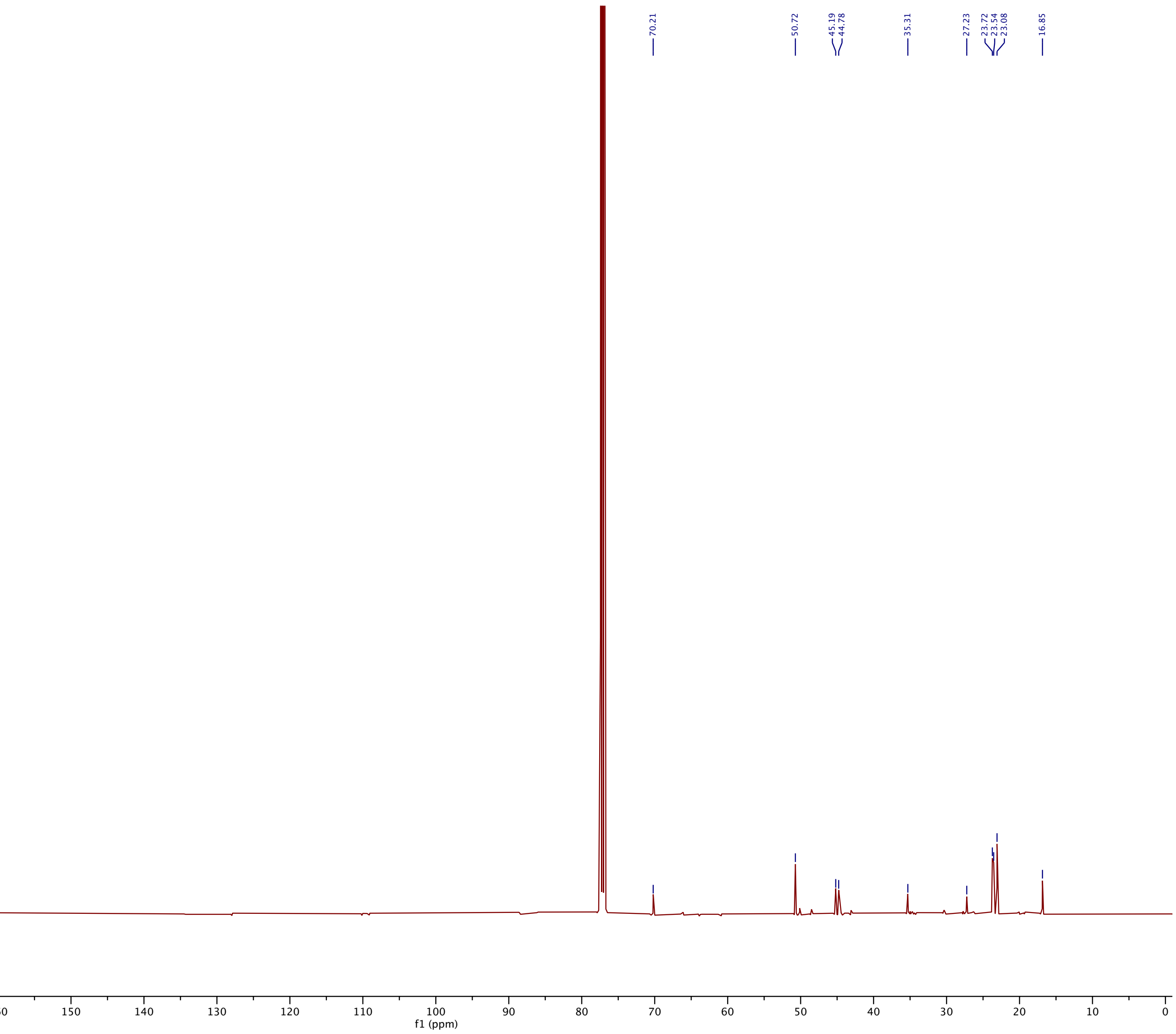

^{1}H NMR of Neramexane hydrochloride (12) (DMSO, 500 MHz)

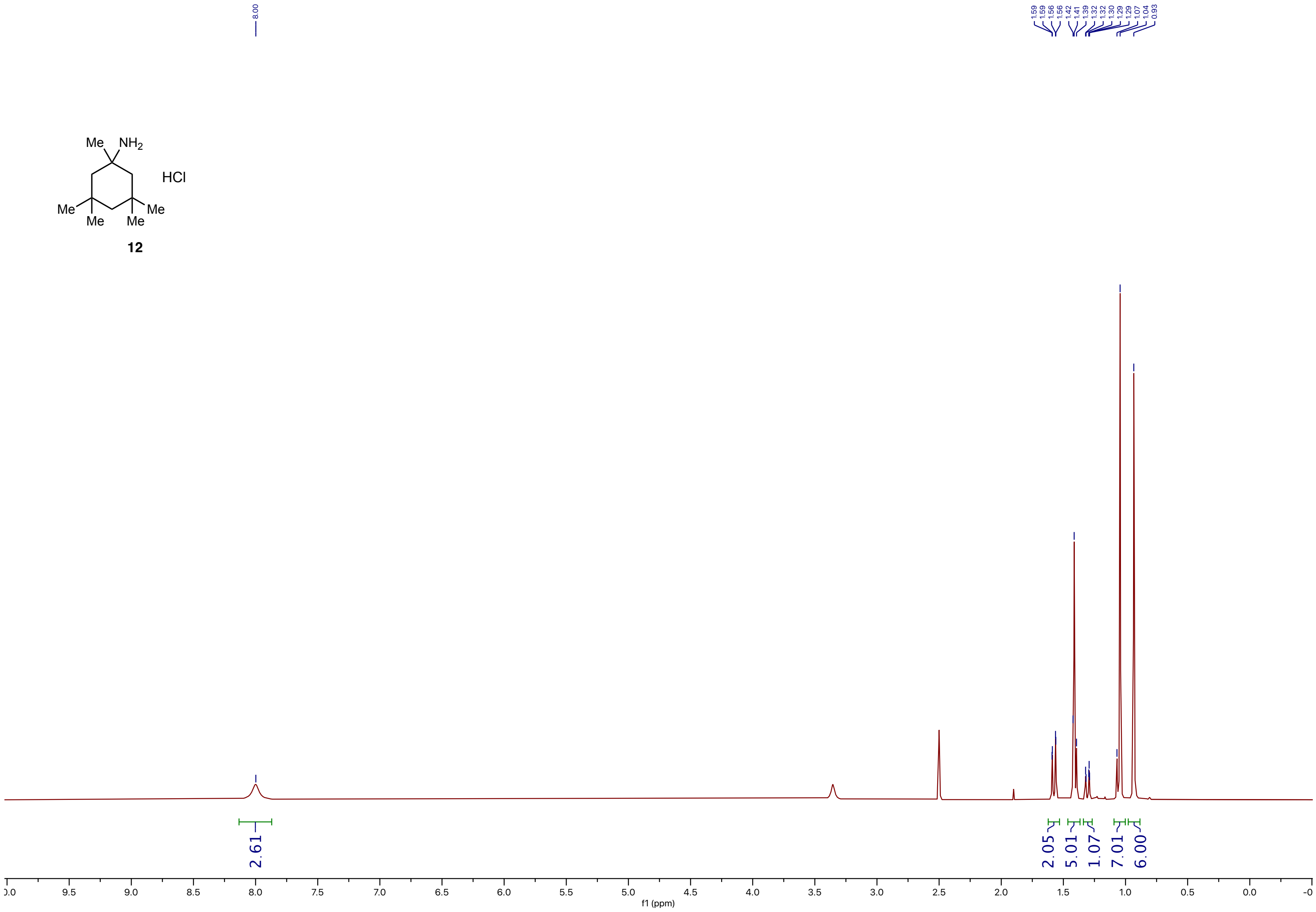

^{13}C NMR of Neramexane hydrochloride (12) (DMSO, 126 MHz)

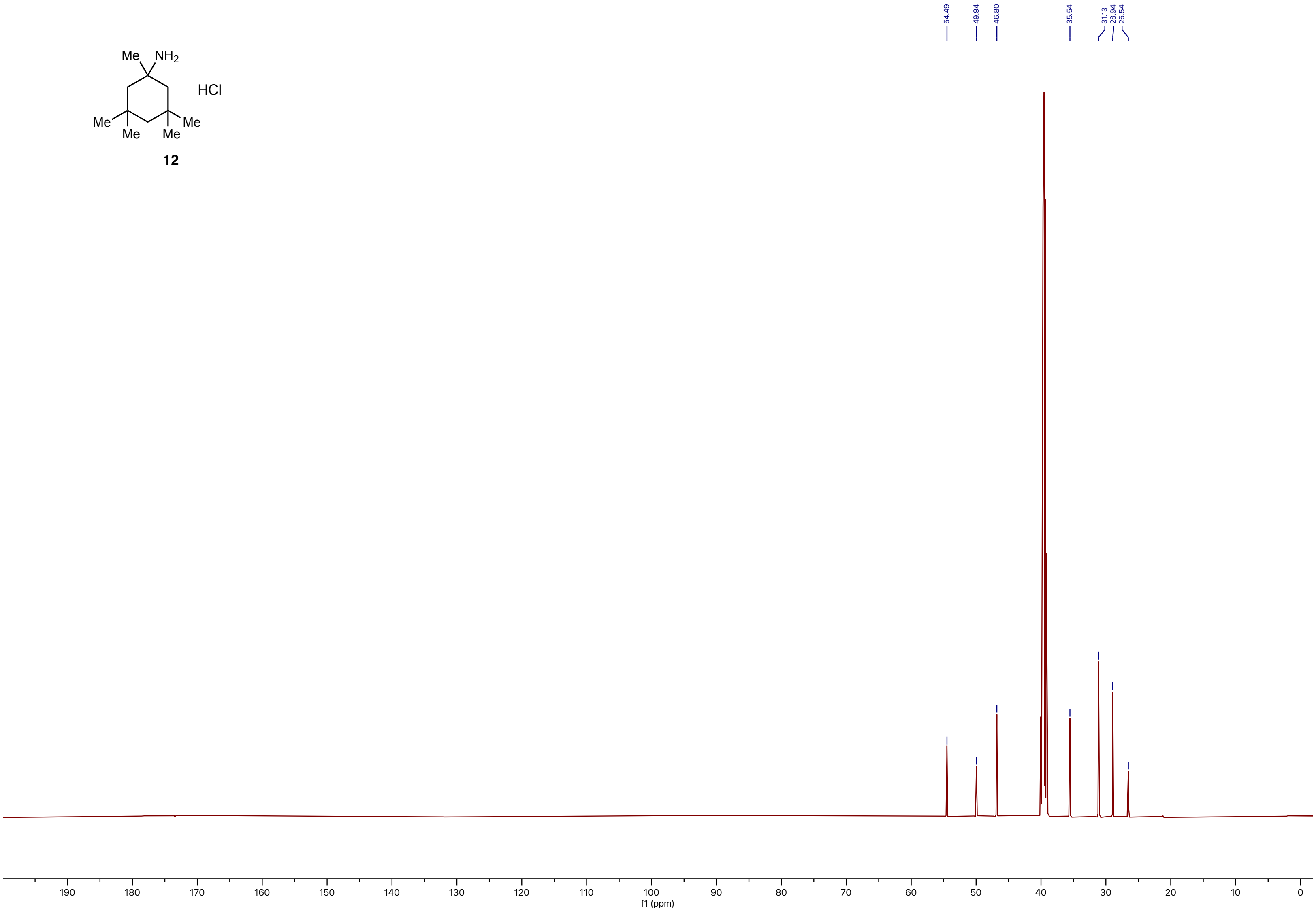

^{1}H NMR of 4-(1-(1H-pyrazol-1-yl)ethyl)-1-tosylpiperidine (15) ($CDCl_3$, 500 MHz)

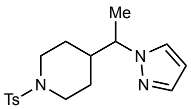

15

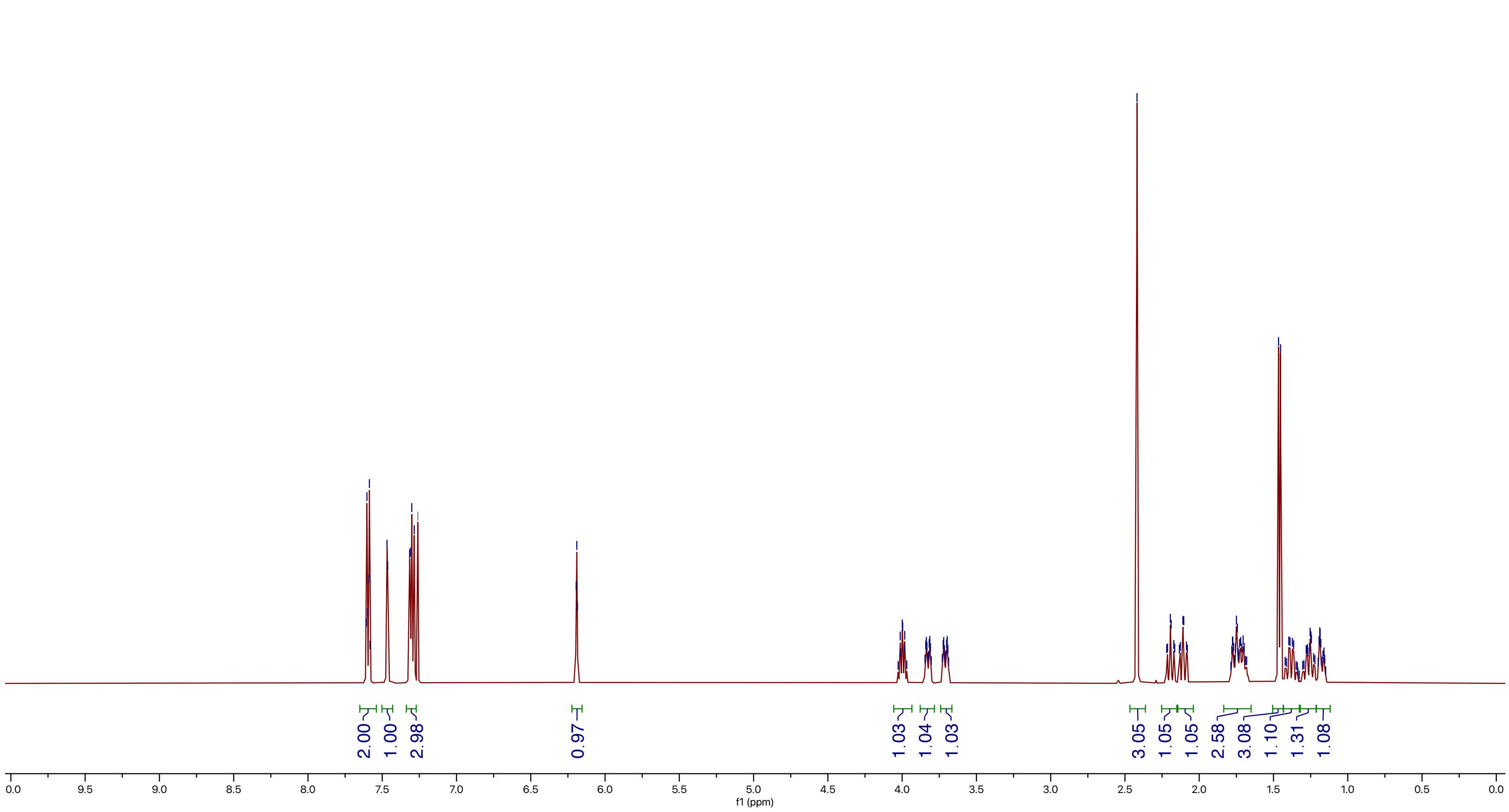

^{13}C NMR of 4-(1-(1H-pyrazol-1-yl)ethyl)-1-tosylpiperidine (15) ($CDCl_3$, 126 MHz)

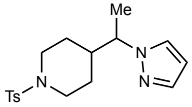

15

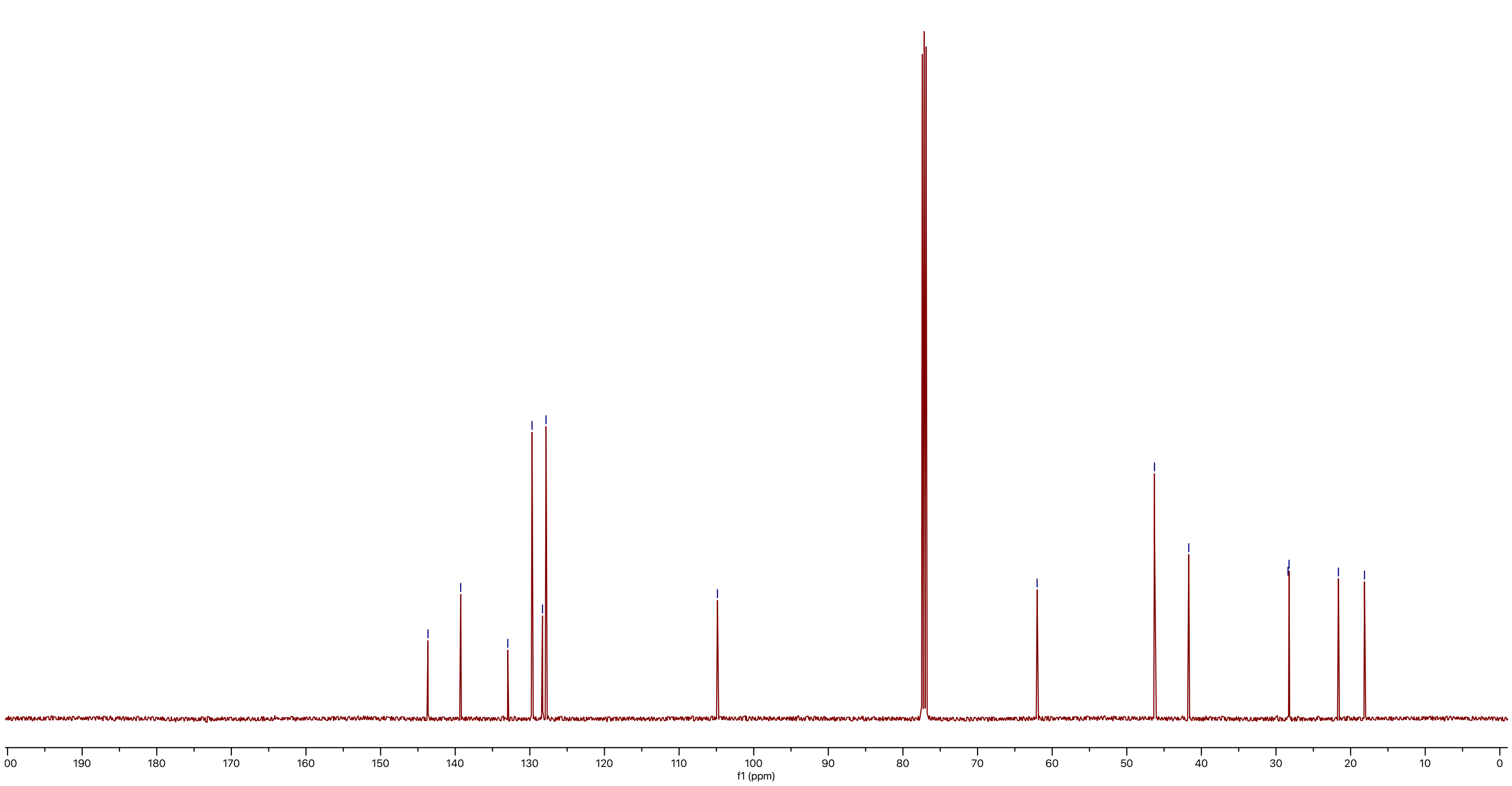

^{1}H NMR of 1-(1-tosylpiperidin-4-yl)ethan-1-amine (16) ($CDCl_3$, 500 MHz)

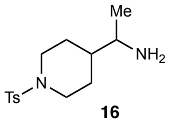

16

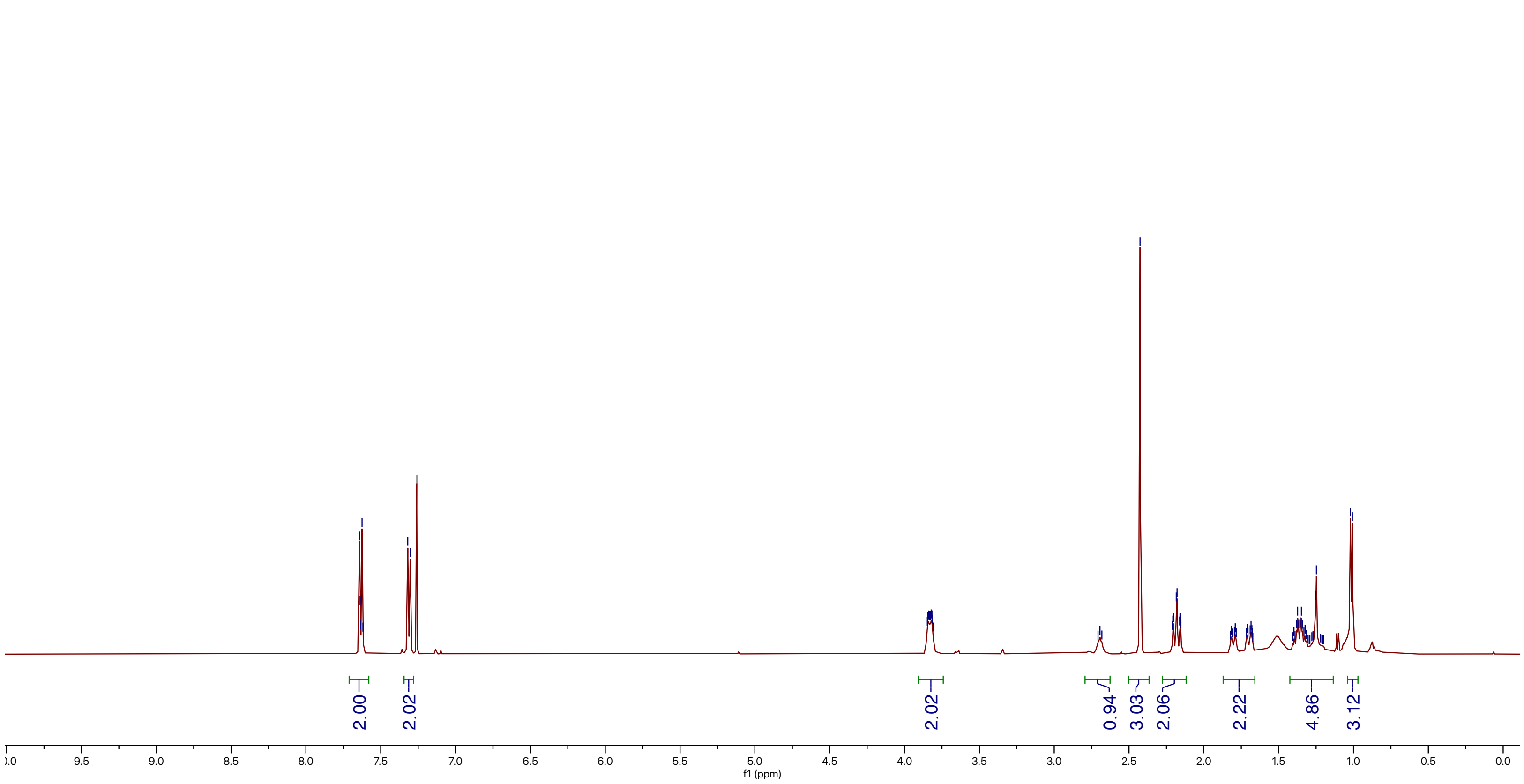

^{13}C NMR of 1-(1-tosylpiperidin-4-yl)ethan-1-amine (16) ($CDCl_3$, 126 MHz)

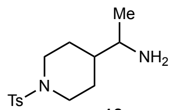

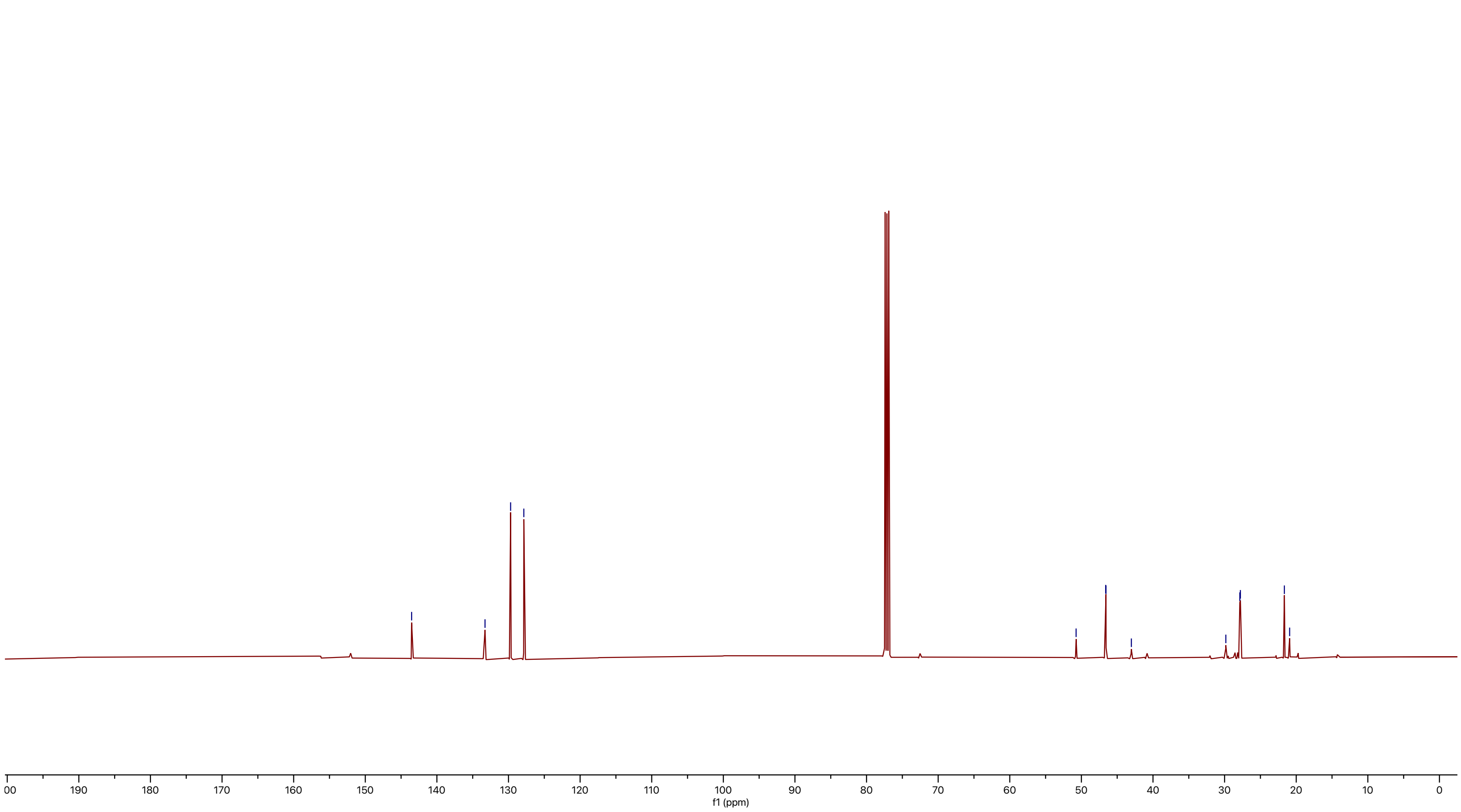

^{1}H NMR of 4-(2-(1*H*-pyrazol-1-yl)propan-2-yl)-1-tosylpiperidine (17) ($CDCl_3$, 500 MHz)

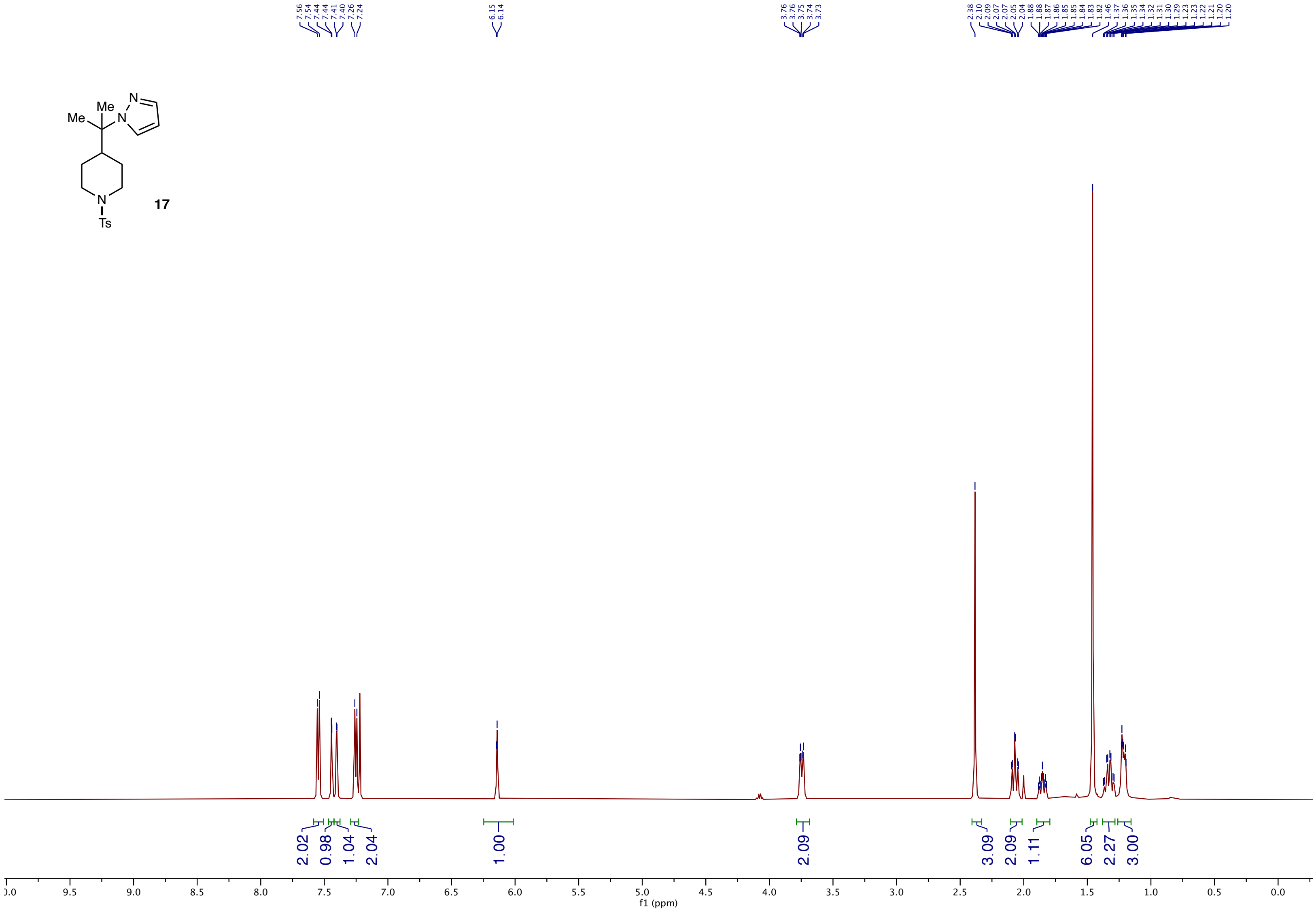

^{13}C NMR of 4-(2-(1*H*-pyrazol-1-yl)propan-2-yl)-1-tosylpiperidine (17) ($CDCl_3$, 126 MHz)

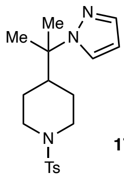

17

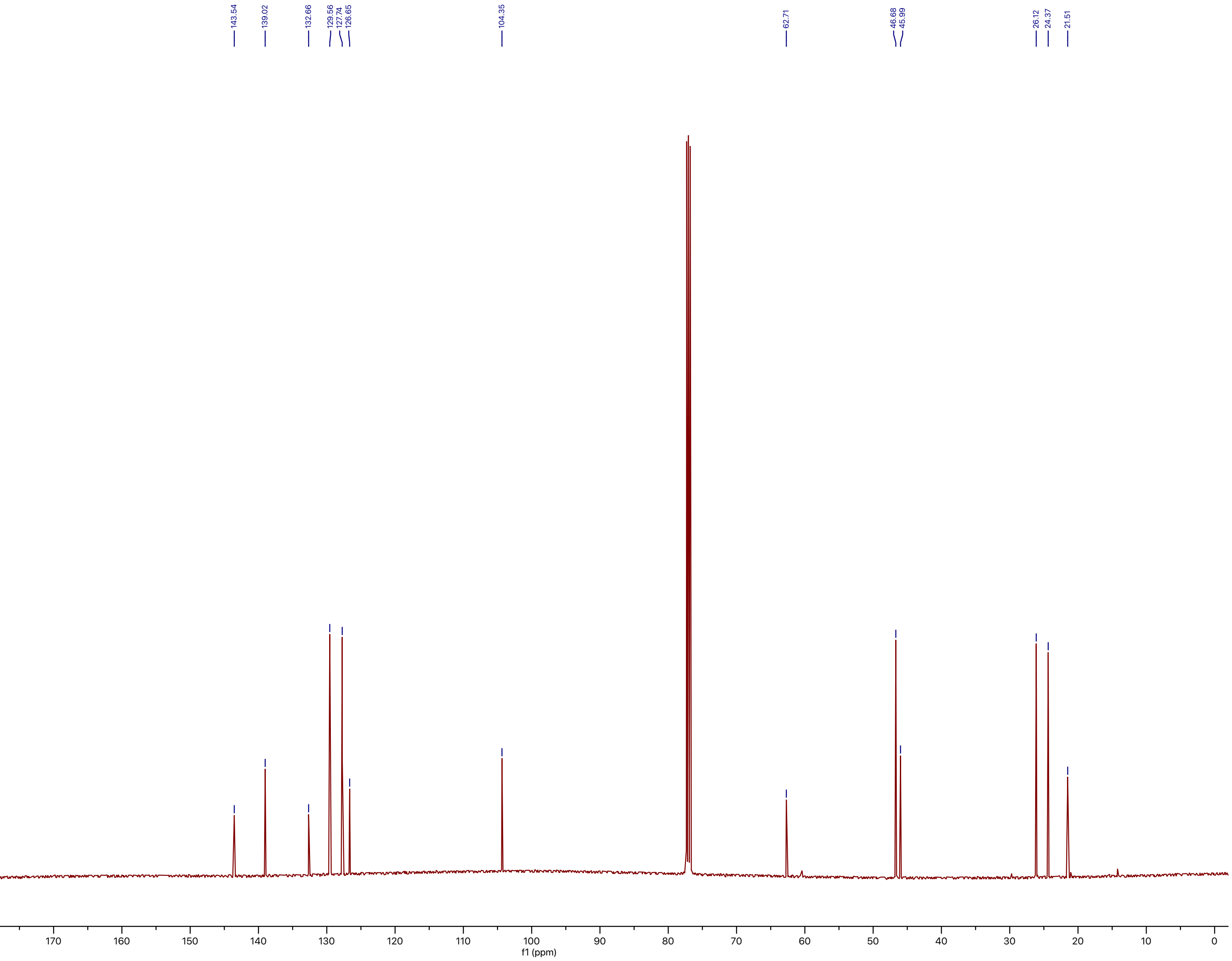

^{1}H NMR of 2-(1-tosylpiperidin-4-yl)propan-2-amine (18) ($CDCl_3$, 500 MHz)

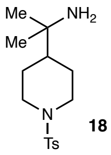

18

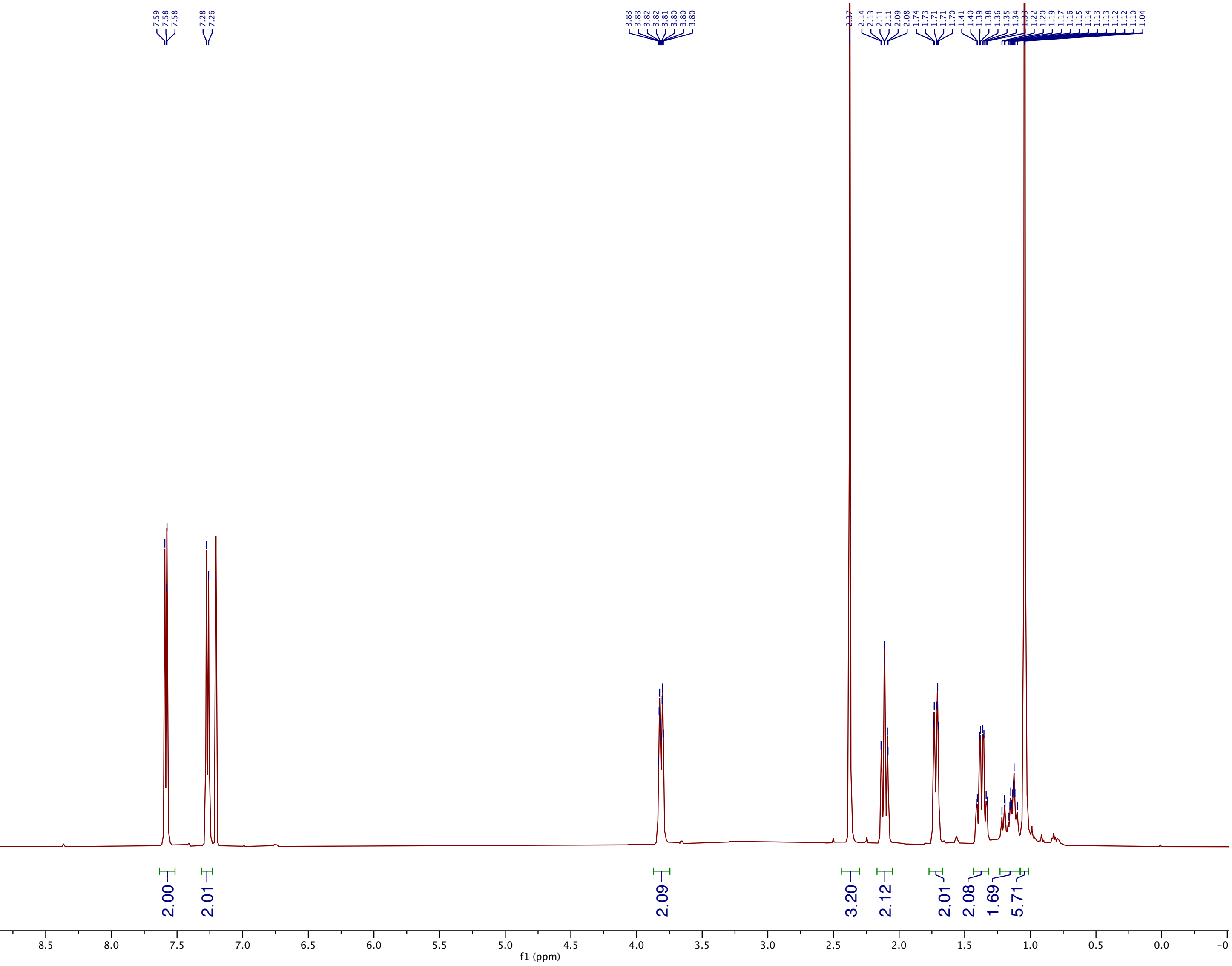

^{13}C NMR of 2-(1-tosylpiperidin-4-yl)propan-2-amine (18) ($CDCl_3$, 126 MHz)

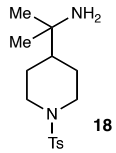

18

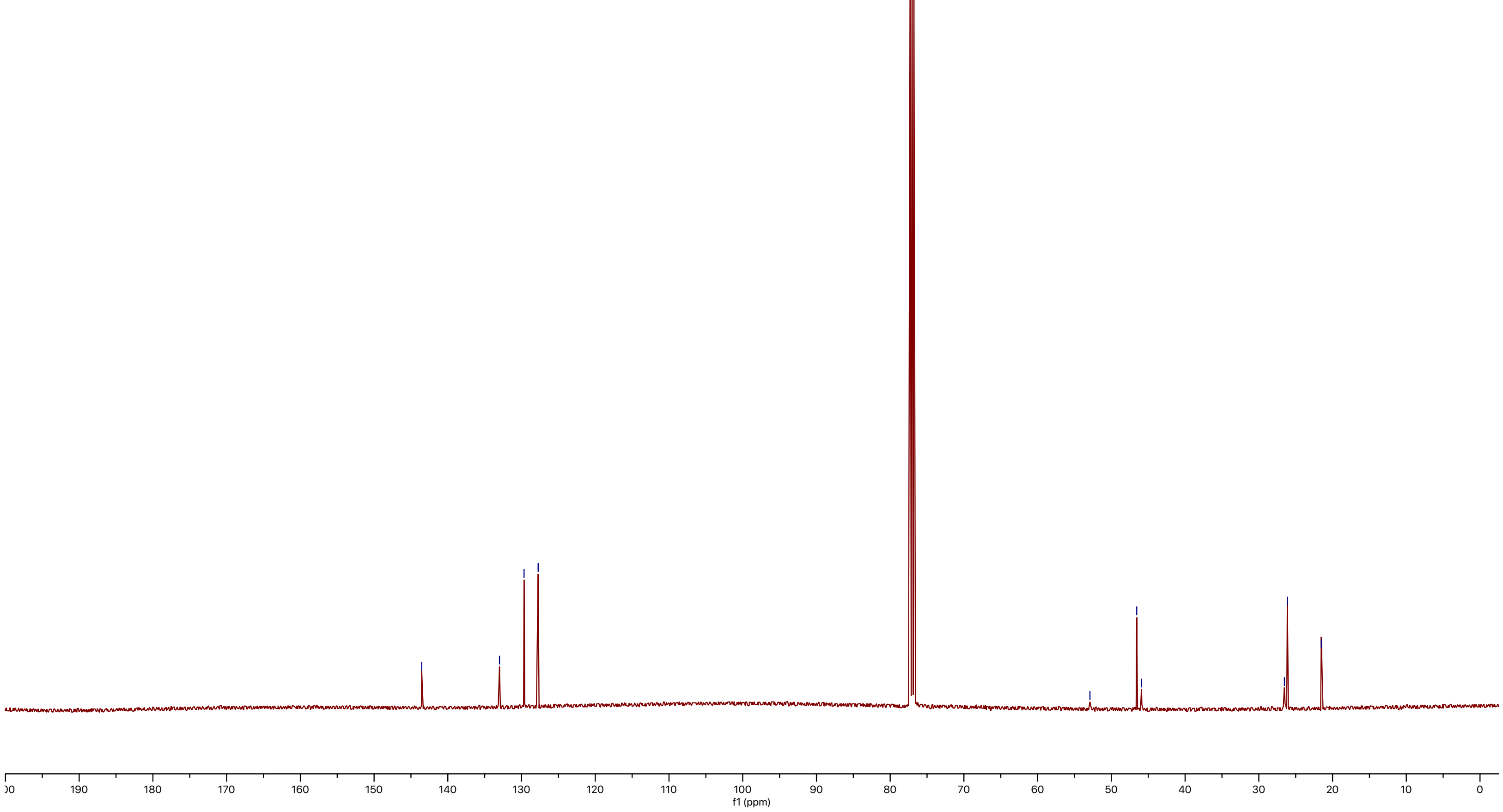

^{1}H NMR of 5-bromo-3-methoxyisobenzofuran-1(3*H*)-one ($CDCl_3$, 500 MHz)

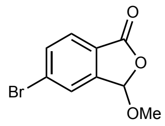

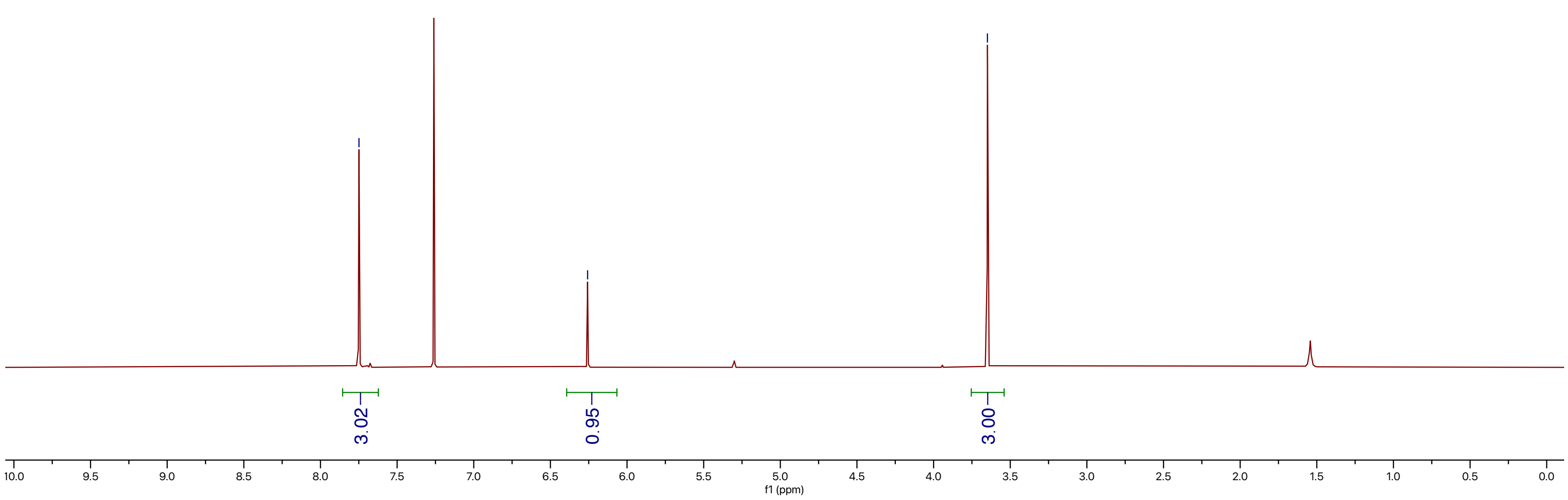

^{13}C NMR of 5-bromo-3-methoxyisobenzofuran-1(3*H*)-one ($CDCl_3$, 126 MHz)

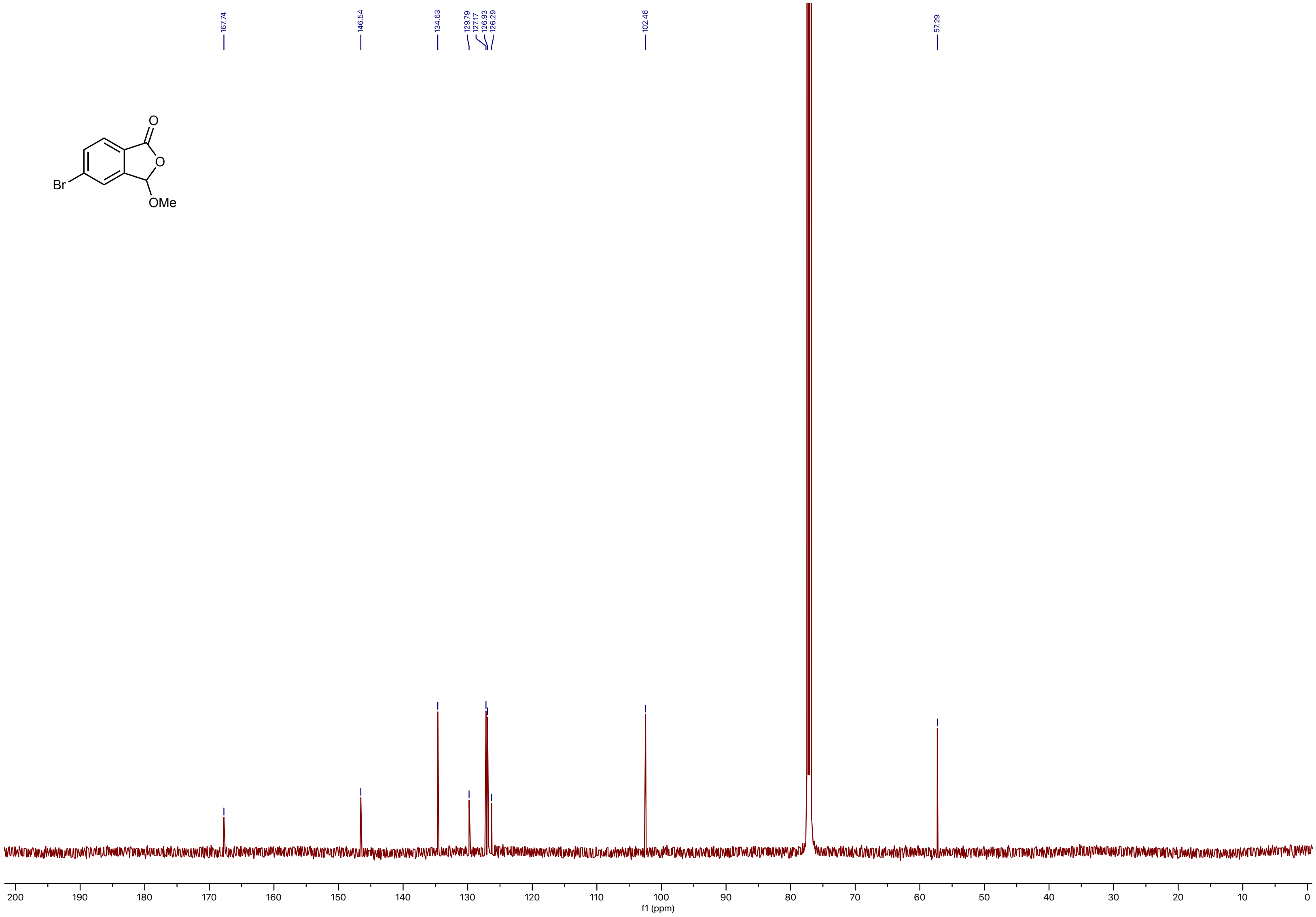

HSQC for 5-bromo-3-methoxyisobenzofuran-1(3*H*)-one

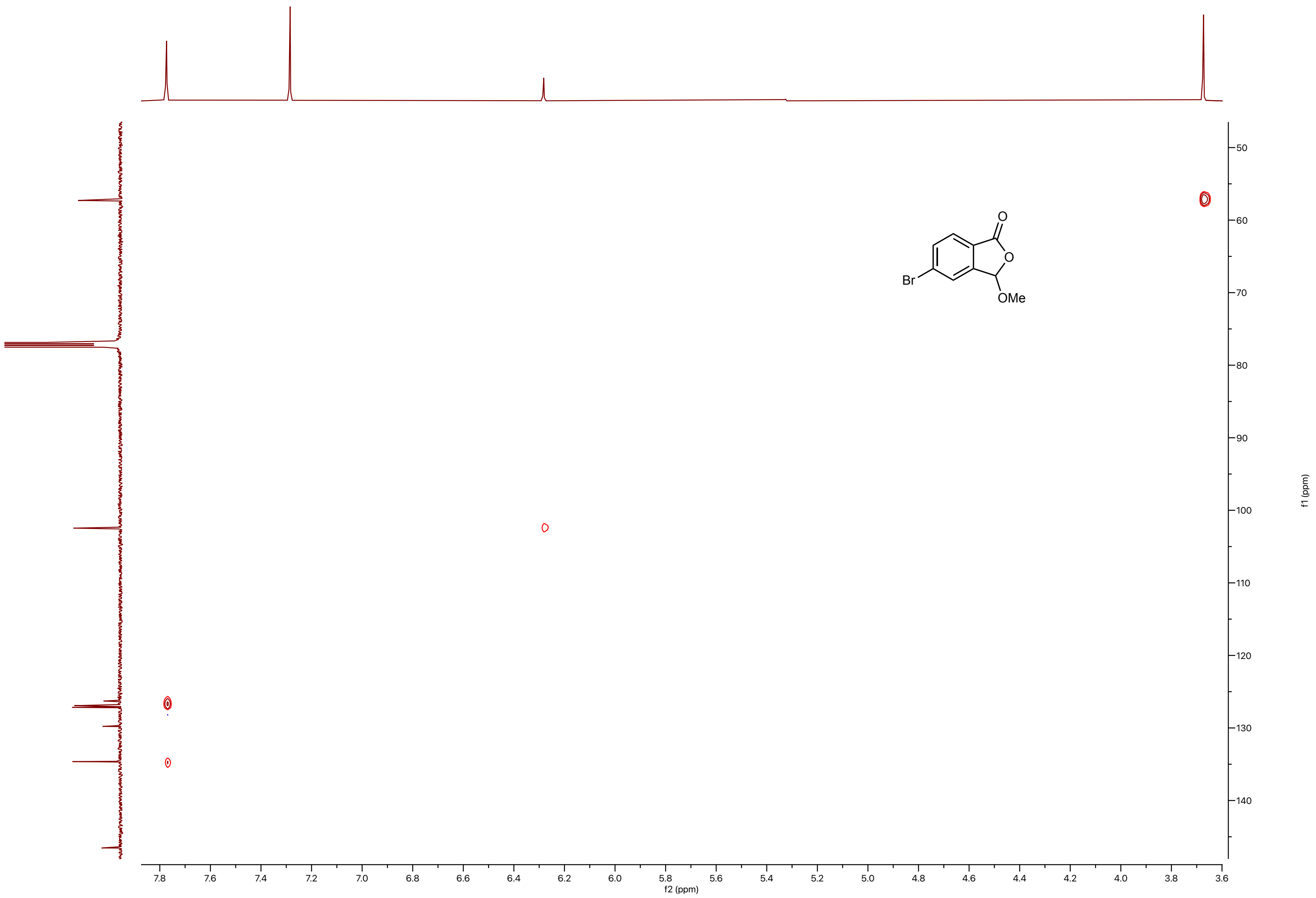

^{1}H NMR of 3-methoxy-5-(4,4,5,5-tetramethyl-1,3,2-dioxaborolan-2-yl)isobenzofuran-1(3*H*)-one ($CDCl_3$, 500 MHz)

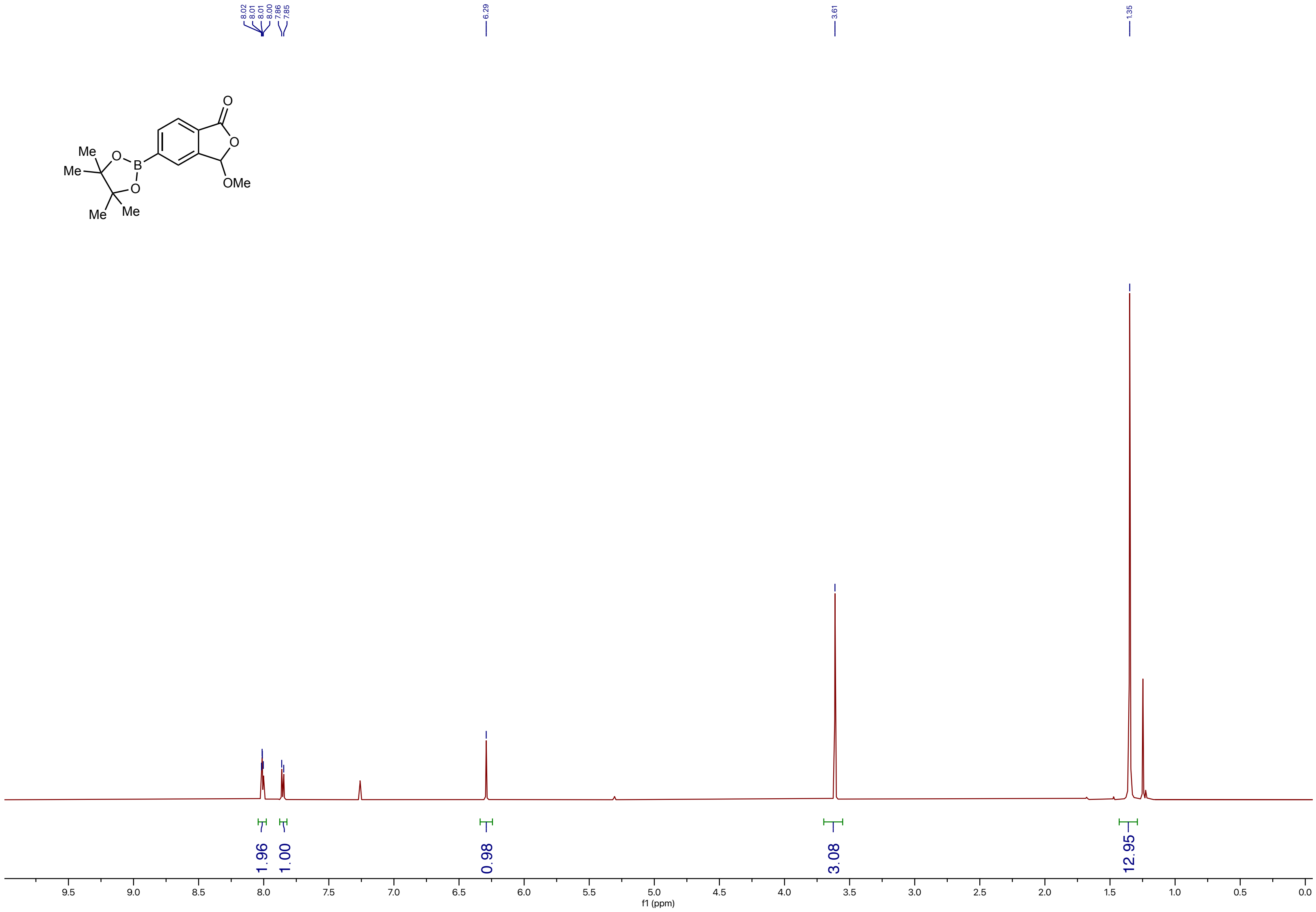

^{13}C NMR of 3-methoxy-5-(4,4,5,5-tetramethyl-1,3,2-dioxaborolan-2-yl)isobenzofuran-1(3*H*)-one ($CDCl_3$, 126 MHz)

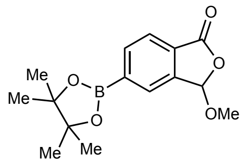

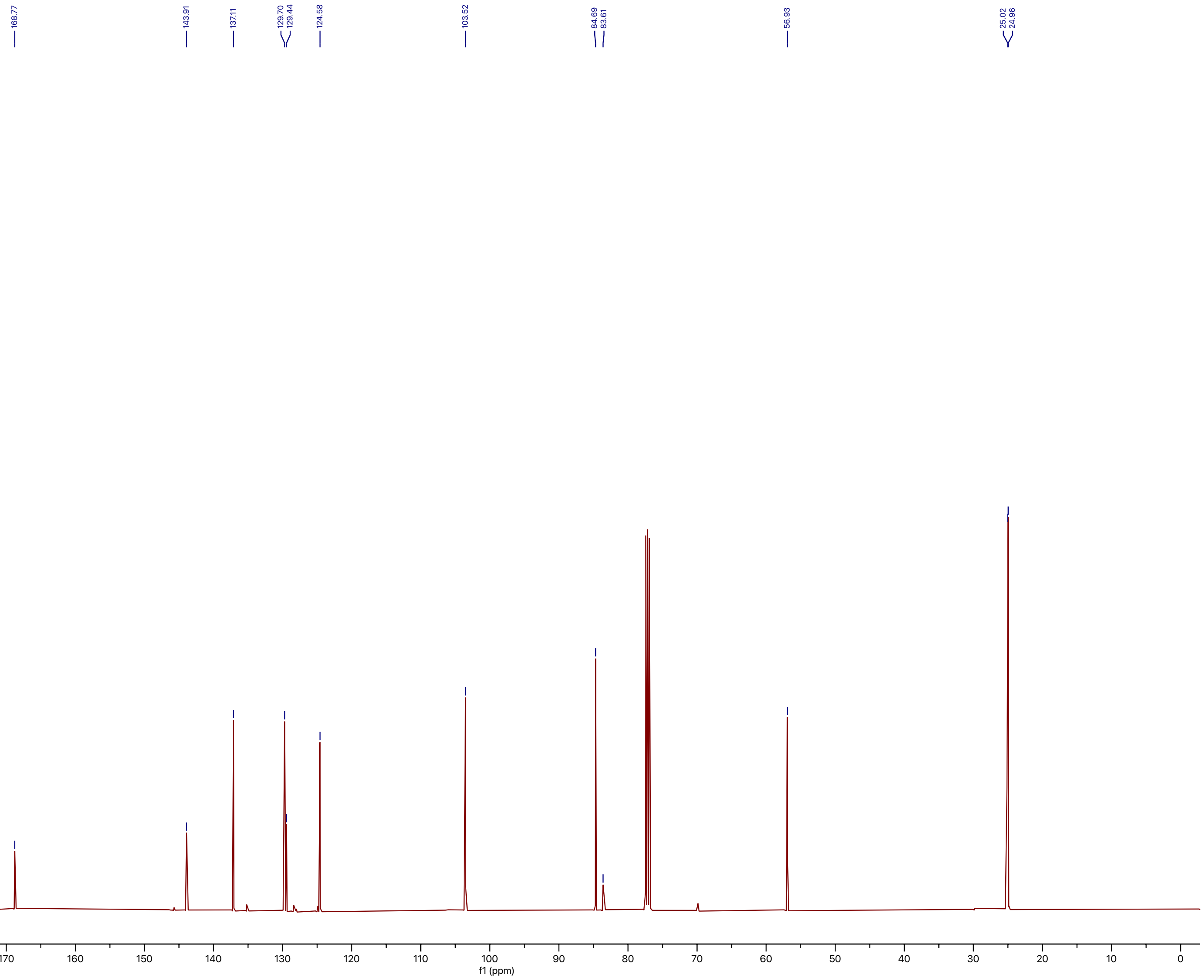

^{1}H NMR of methyl 2-(dimethoxymethyl)-4-(2,8-dimethylimidazo[1,2-*b*]pyridazin-6-yl)benzoate (21) ($CDCl_3$, 500 MHz)

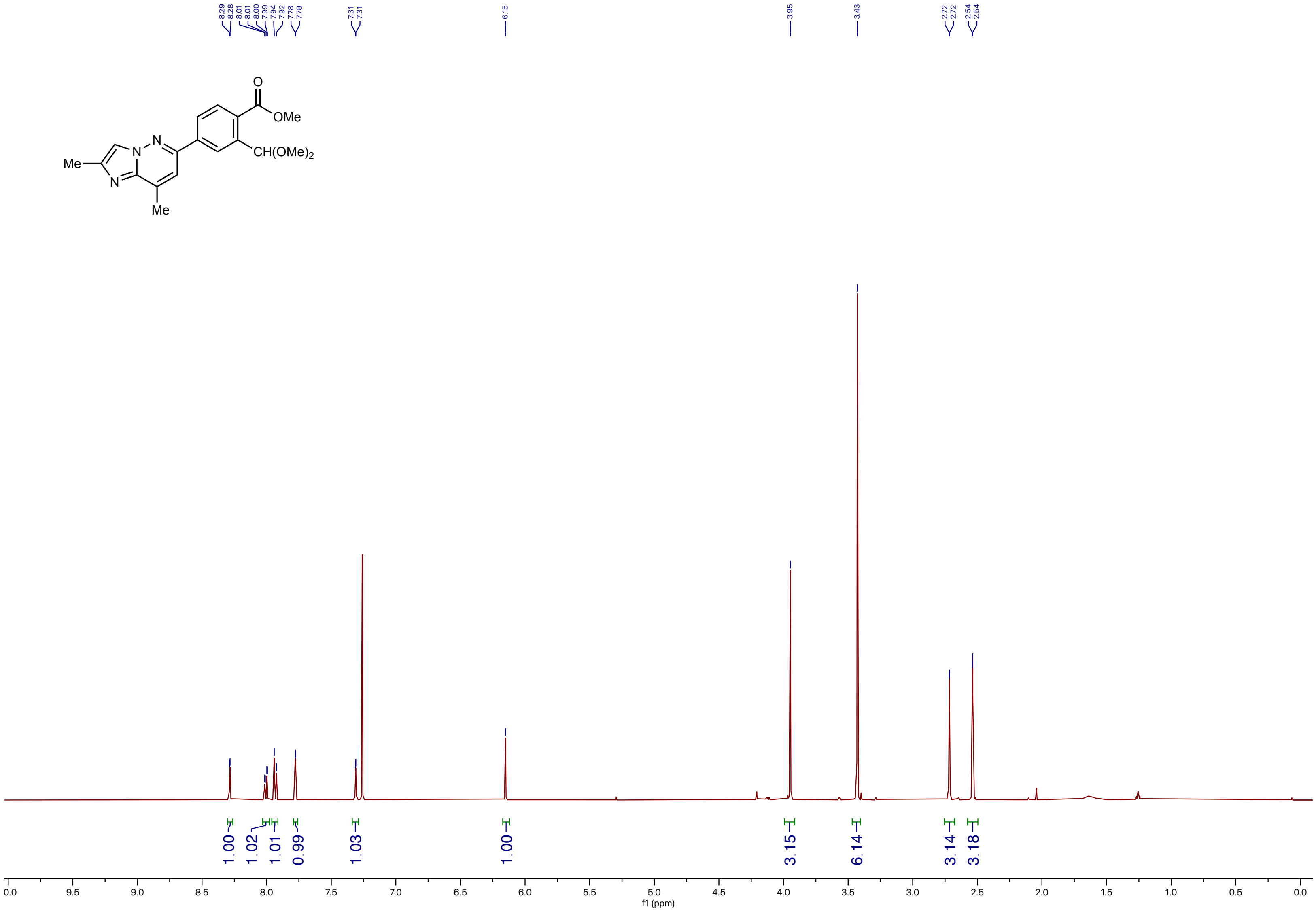

^{13}C NMR of methyl 2-(dimethoxymethyl)-4-(2,8-dimethylimidazo[1,2-*b*]pyridazin-6-yl)benzoate (21) ($CDCl_3$, 126 MHz)

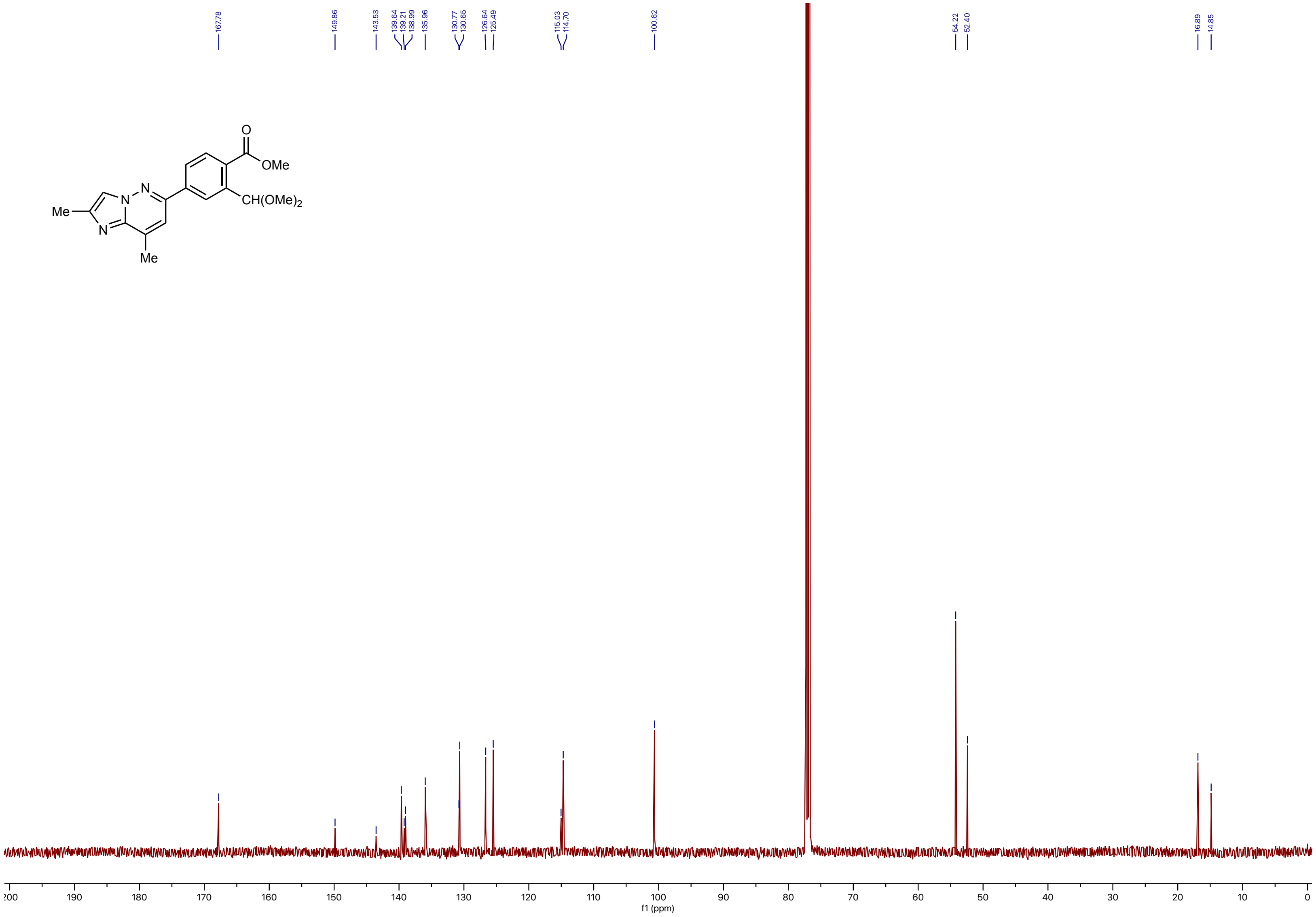

^{1}H NMR of 25 (DMSO, 500 MHz)

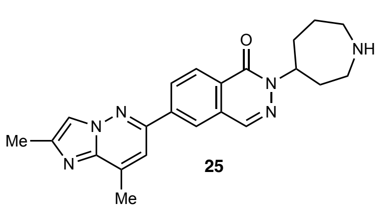

25

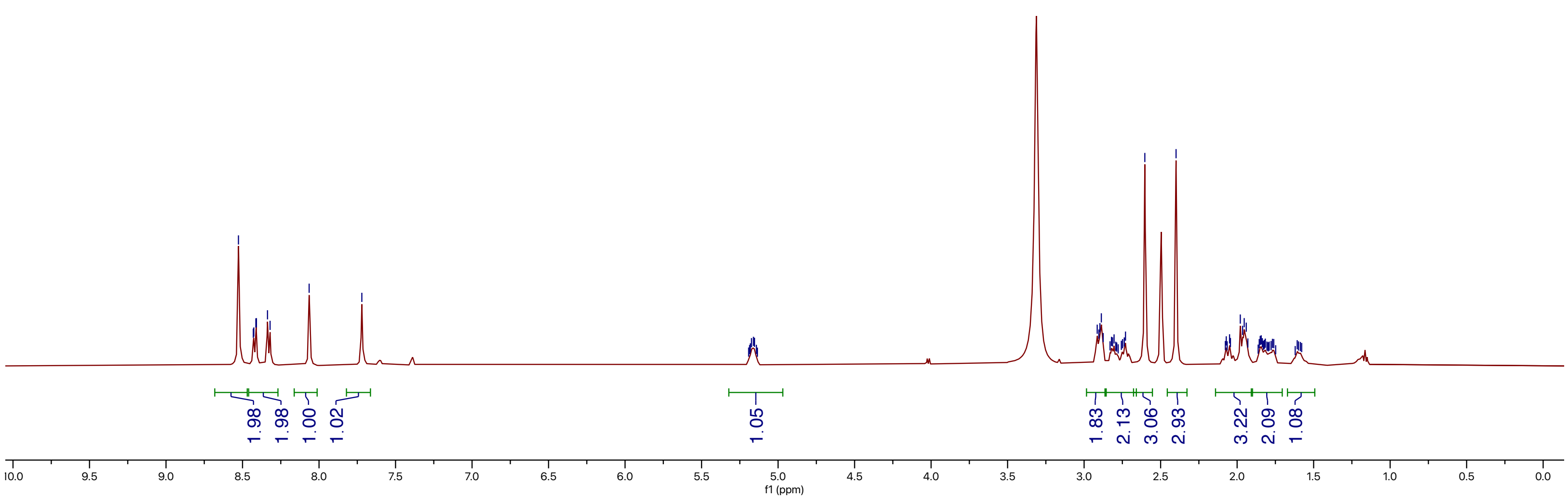

^{13}C NMR of 25 (DMSO, 126 MHz)

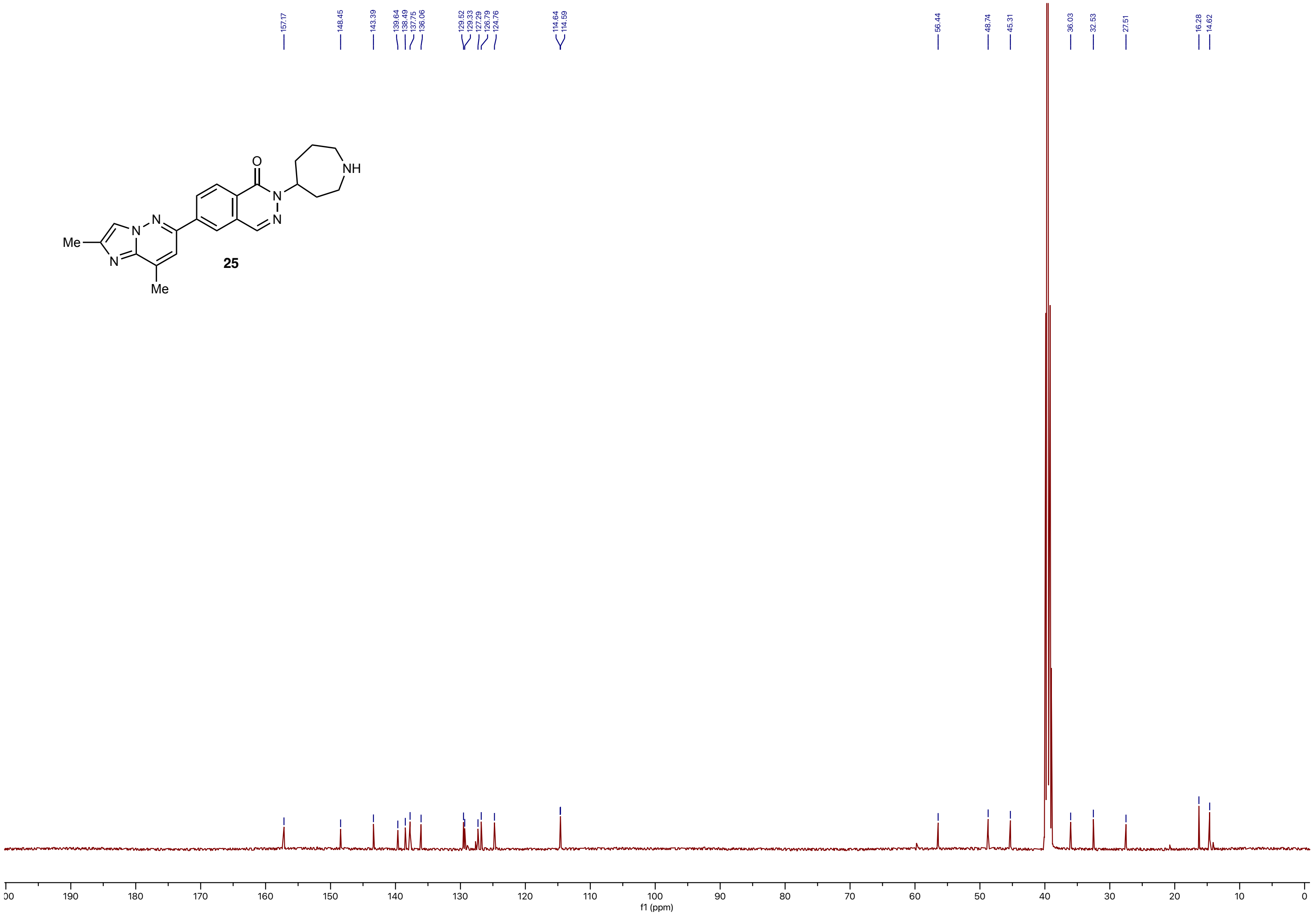

^{1}H NMR of 23 (DMSO, 500 MHz)

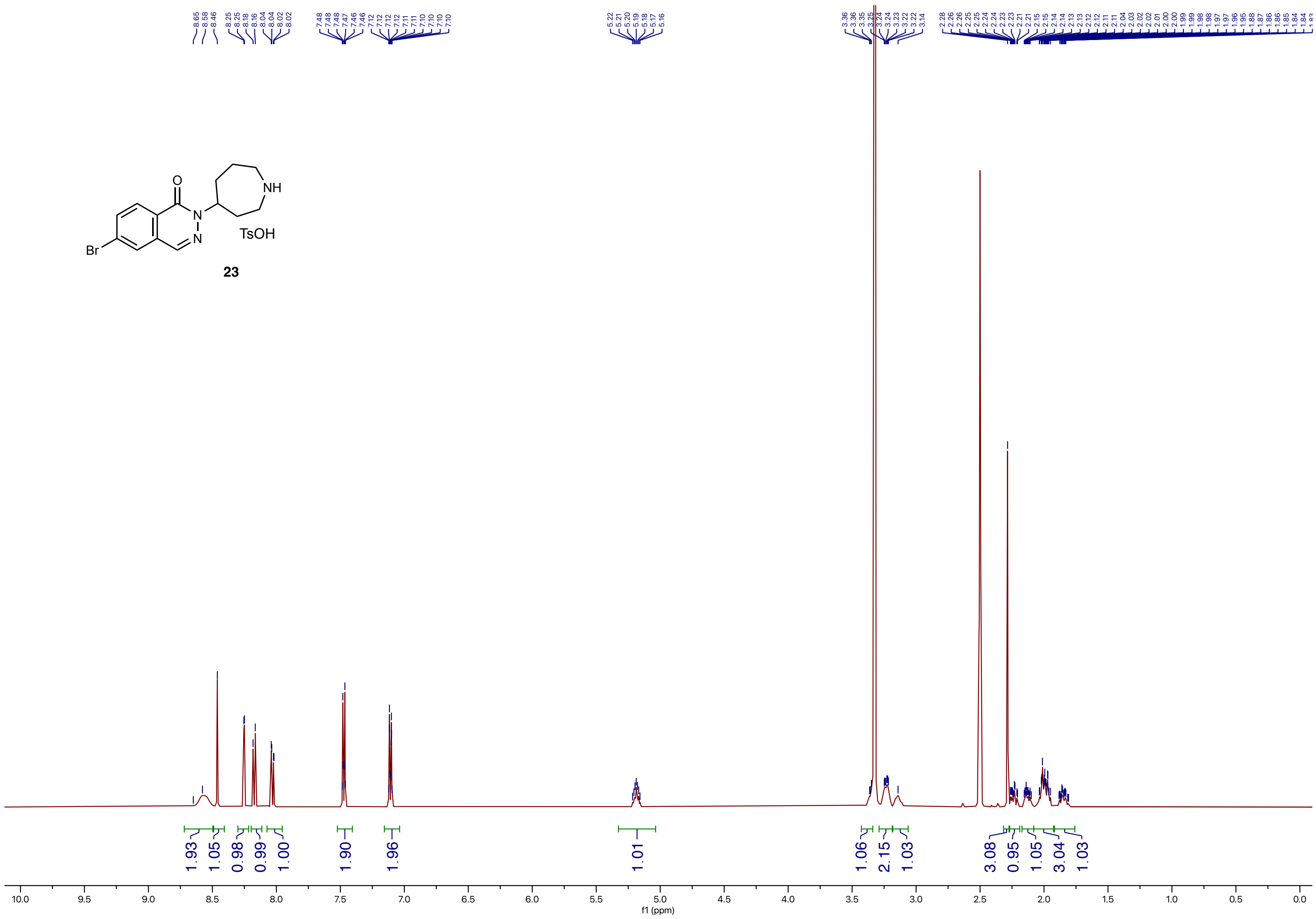

^{13}C NMR of 23 (DMSO, 126 MHz)

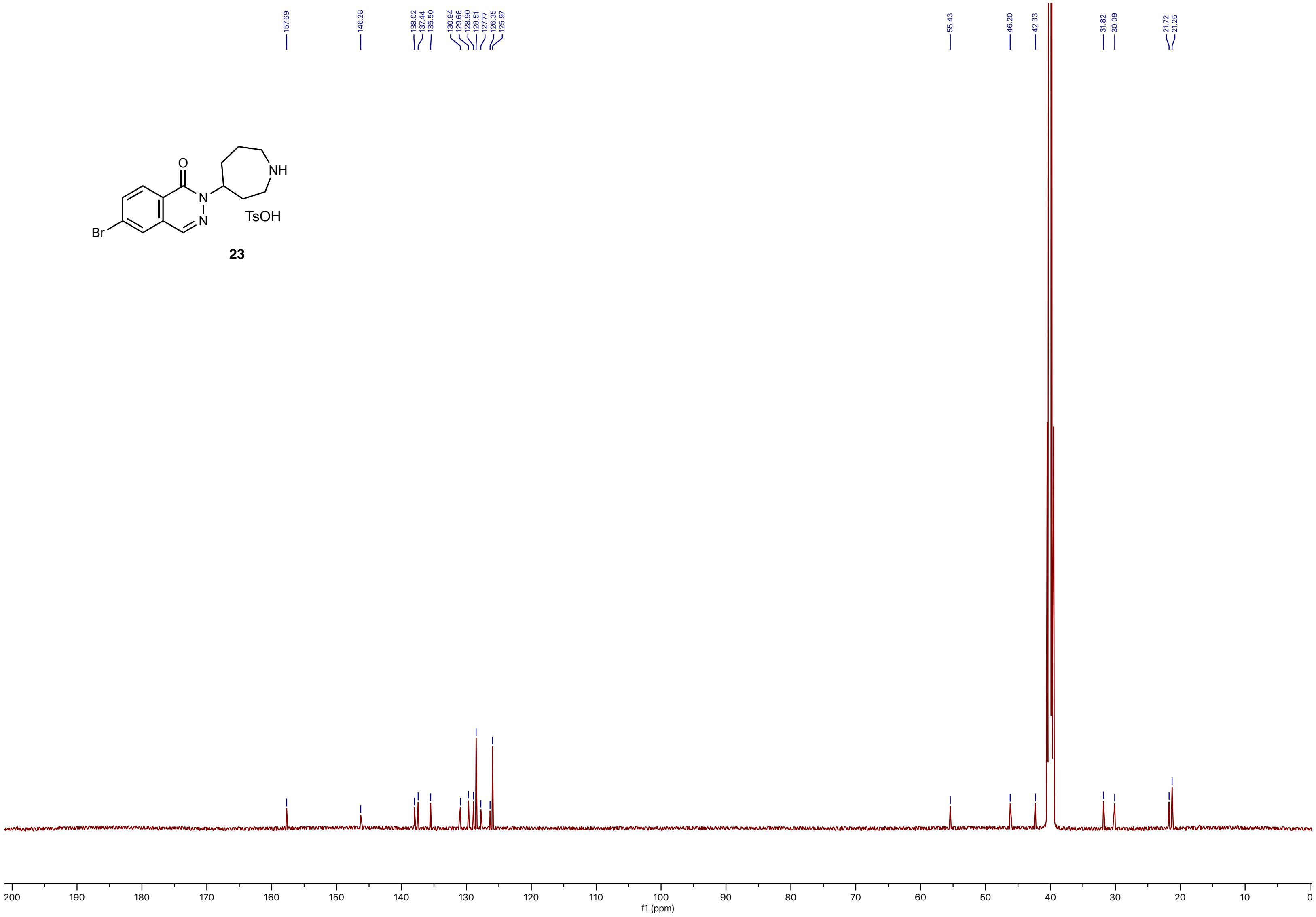

^{1}H NMR of (Amino-^{15}*N*)triphenylphosphonium bromide ($CDCl_3$, 600 MHz)

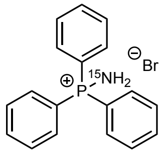

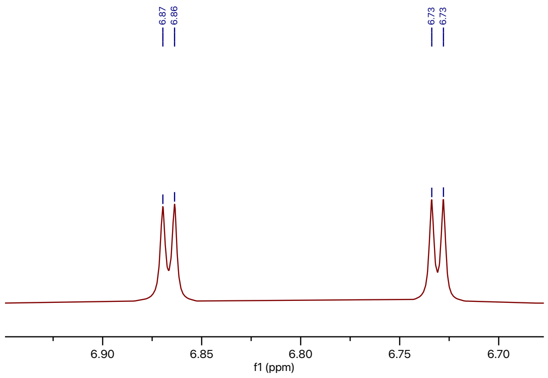

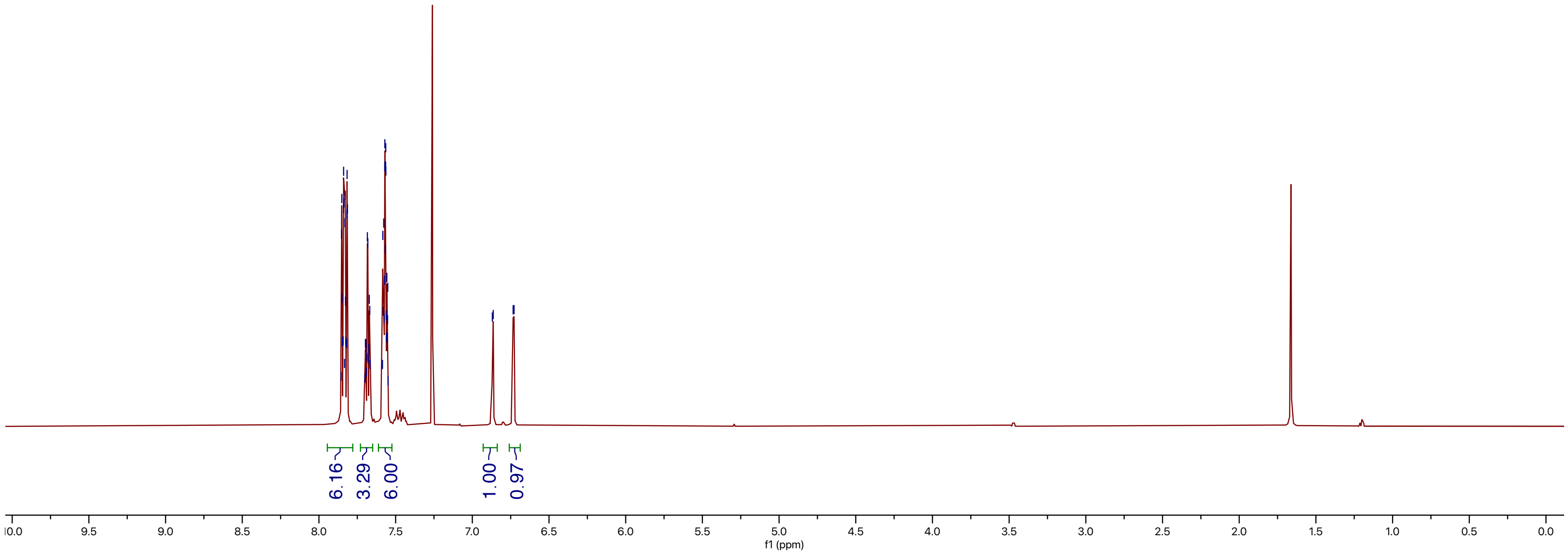

^{13}C NMR of (Amino-^{15}*N*)triphenylphosphonium bromide ($CDCl_3$, 151 MHz)

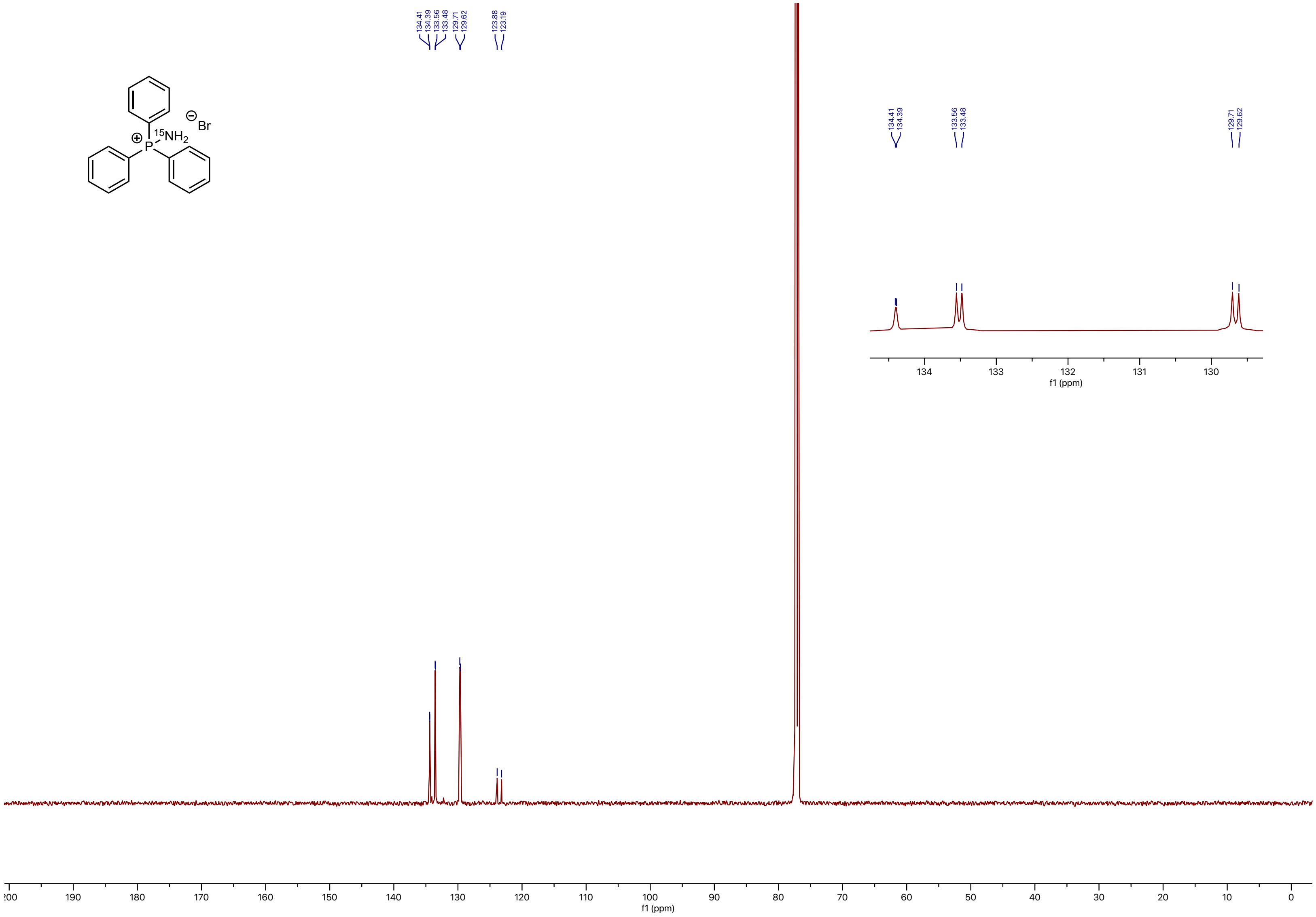

^{31}P NMR of (Amino-^{15}*N*)triphenylphosphonium bromide ($CDCl_3$, 243 MHz)

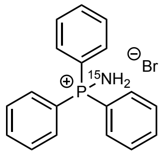

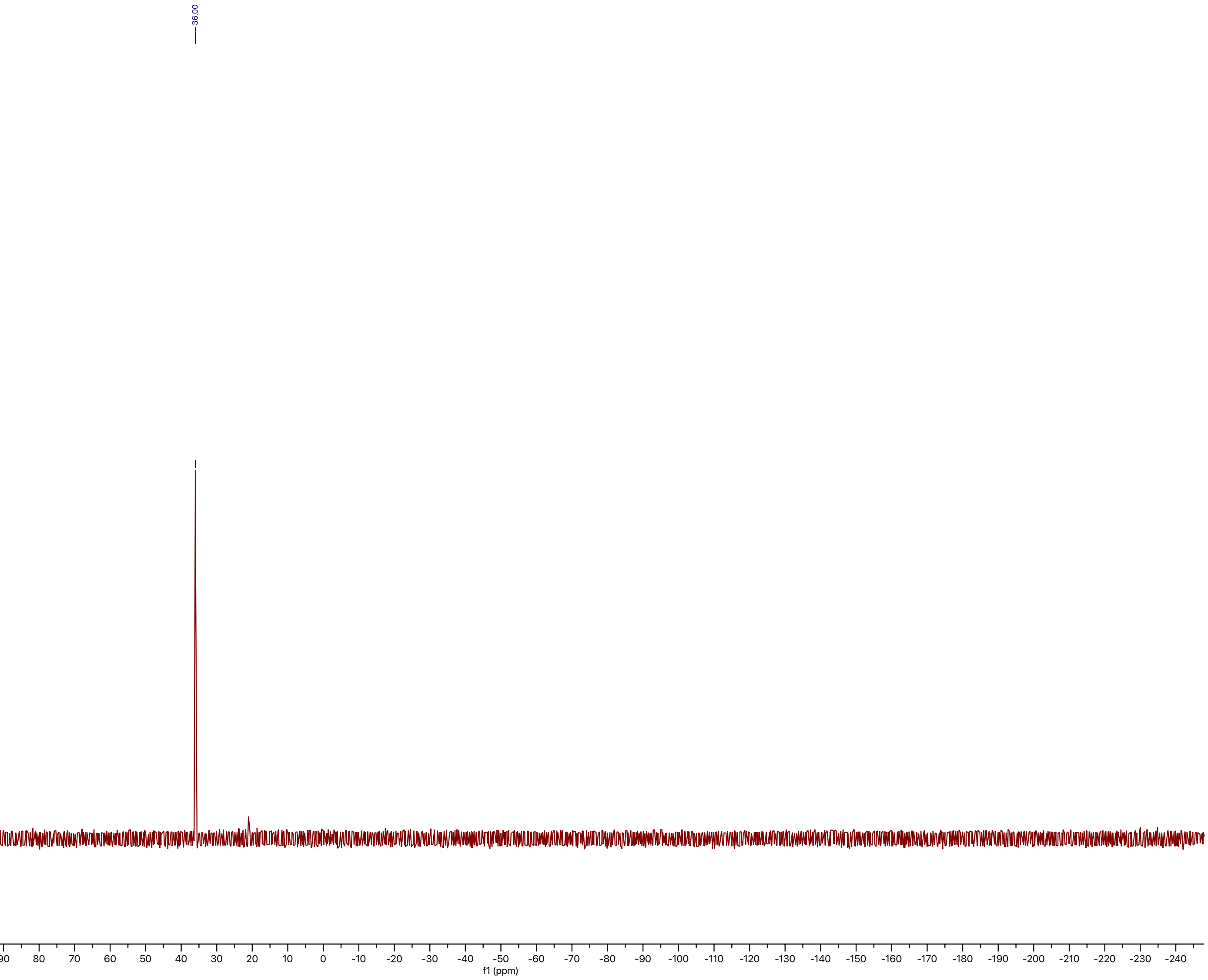

Crude ^{1}H NMR of *N*-chloro-2,2,2-trifluoro-1-phenylethan-1-imine-^{15}*N* (31) (CDCl$_3$, 500 MHz)

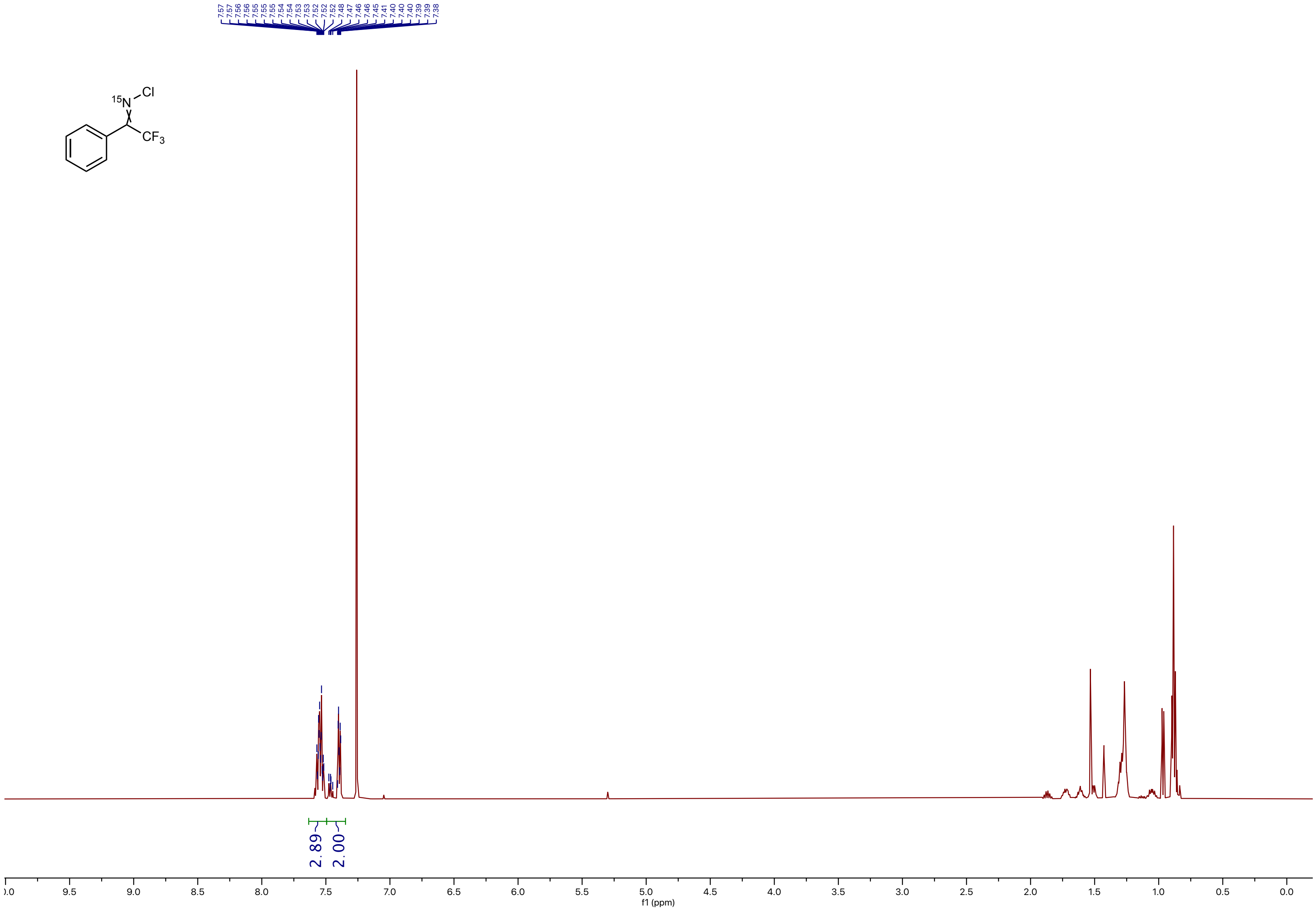

Crude ^{13}C NMR of *N*-chloro-2,2,2-trifluoro-1-phenylethan-1-imine-^{15}N (31) ($CDCl_3$, 126 MHz)

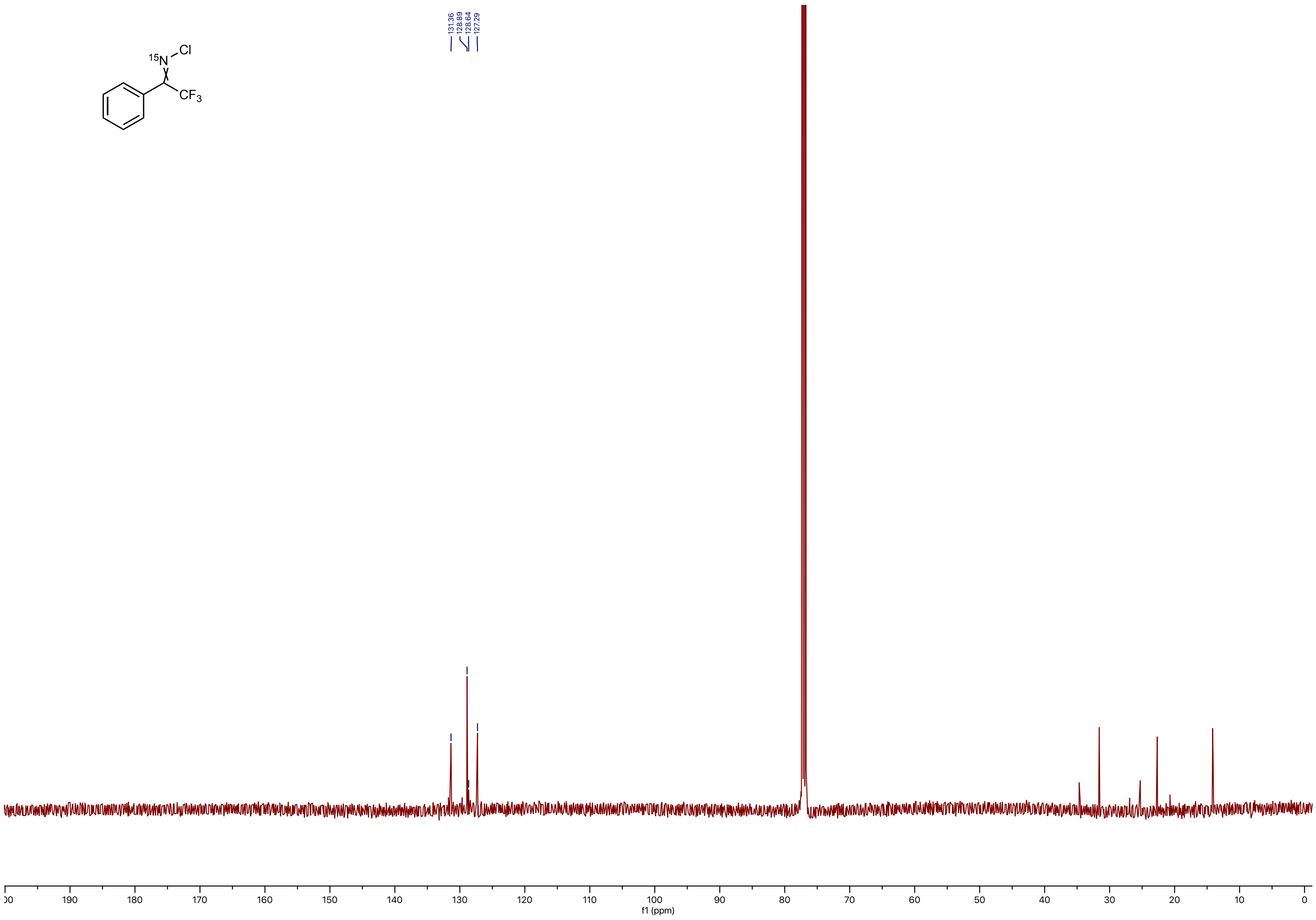

Crude ^{19}F NMR of *N*-chloro-2,2,2,trifluoro-1-phenylethan-1-imine-^{15}*N* (31) ($CDCl_3$, 471 MHz)

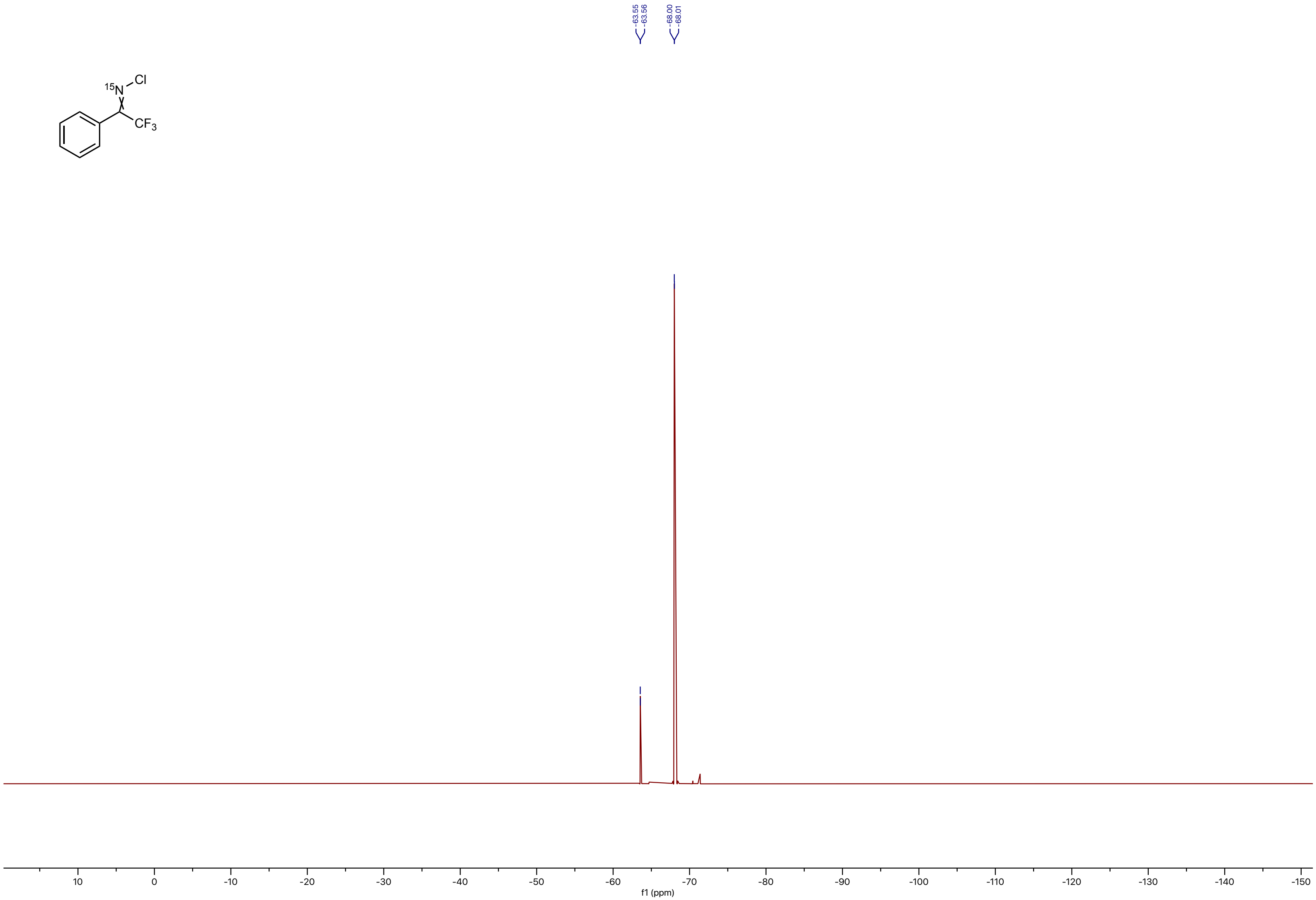

^{1}H NMR of 3-phenyl-3-(trifluoromethyl)diaziridine-1,2-$^{15}N_2$ (32) ($CDCl_3$, 500 MHz)

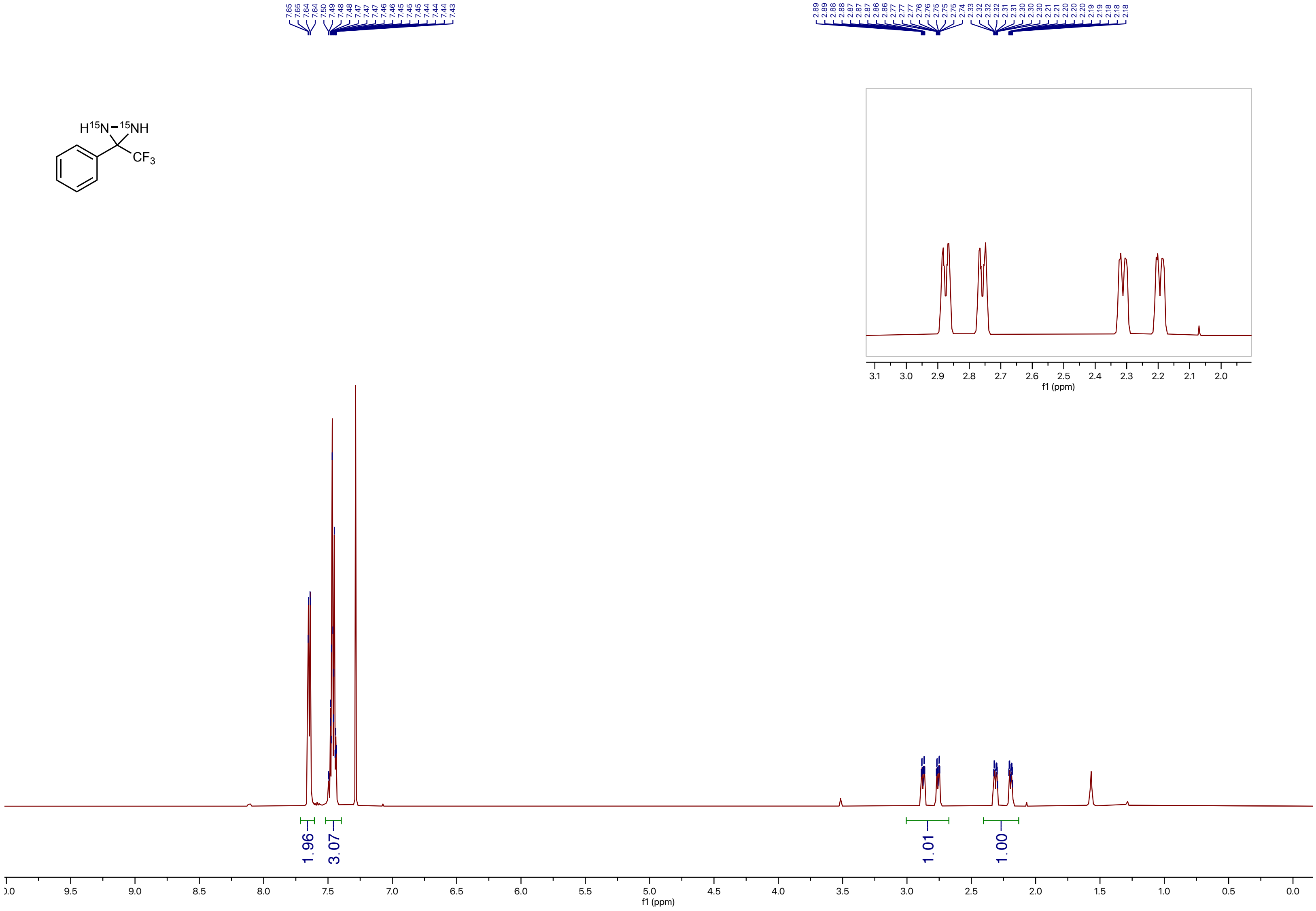

^{13}C NMR of 3-phenyl-3-(trifluoromethyl) diaziridine-1,2-$^{15}N_2$ (32) ($CDCl_3$, 126 MHz)

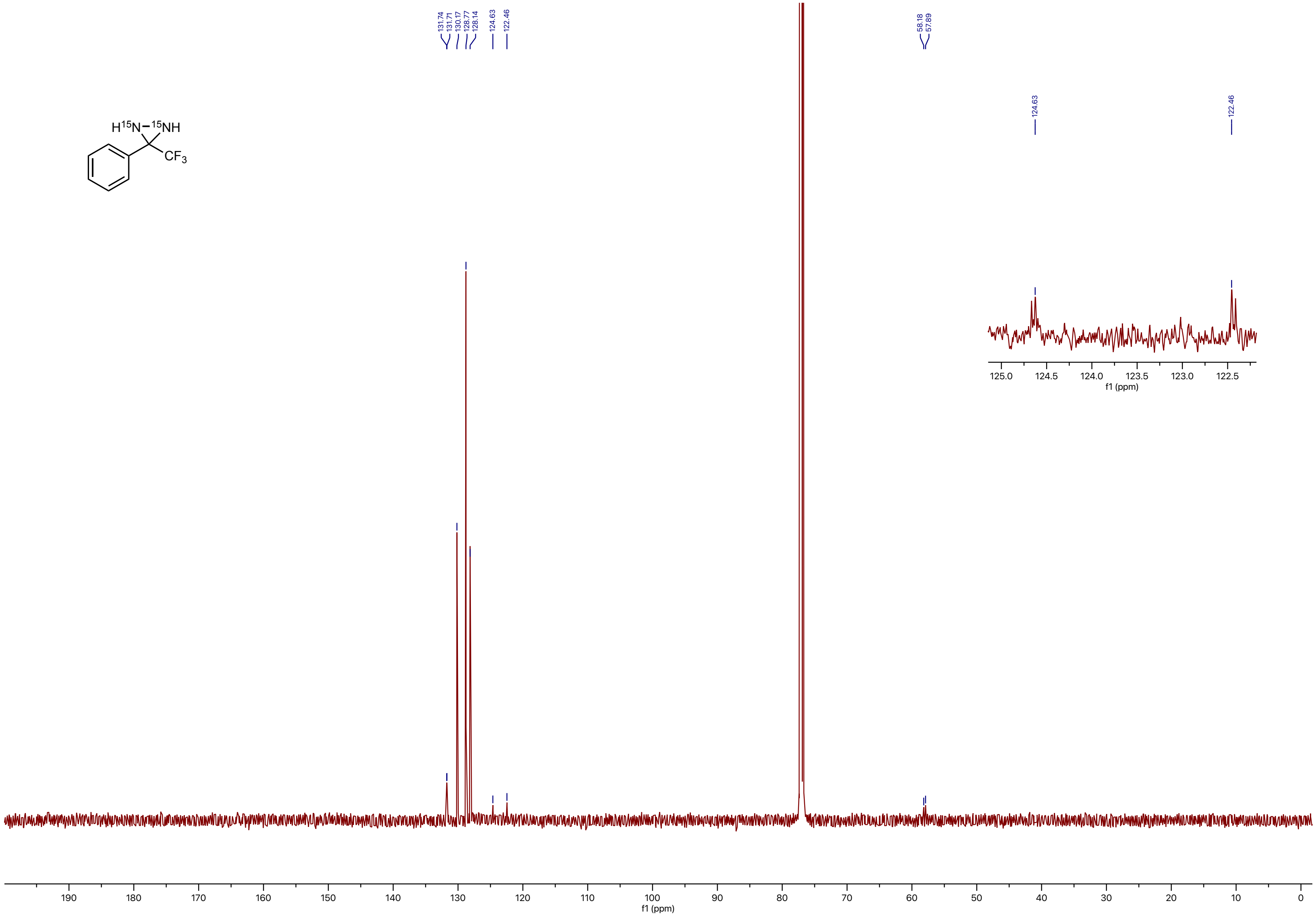

^{19}F NMR of 3-phenyl-3-(trifluoromethyl) diaziridine-1,2-$^{15}N_2$ (32) ($CDCl_3$, 471 MHz)

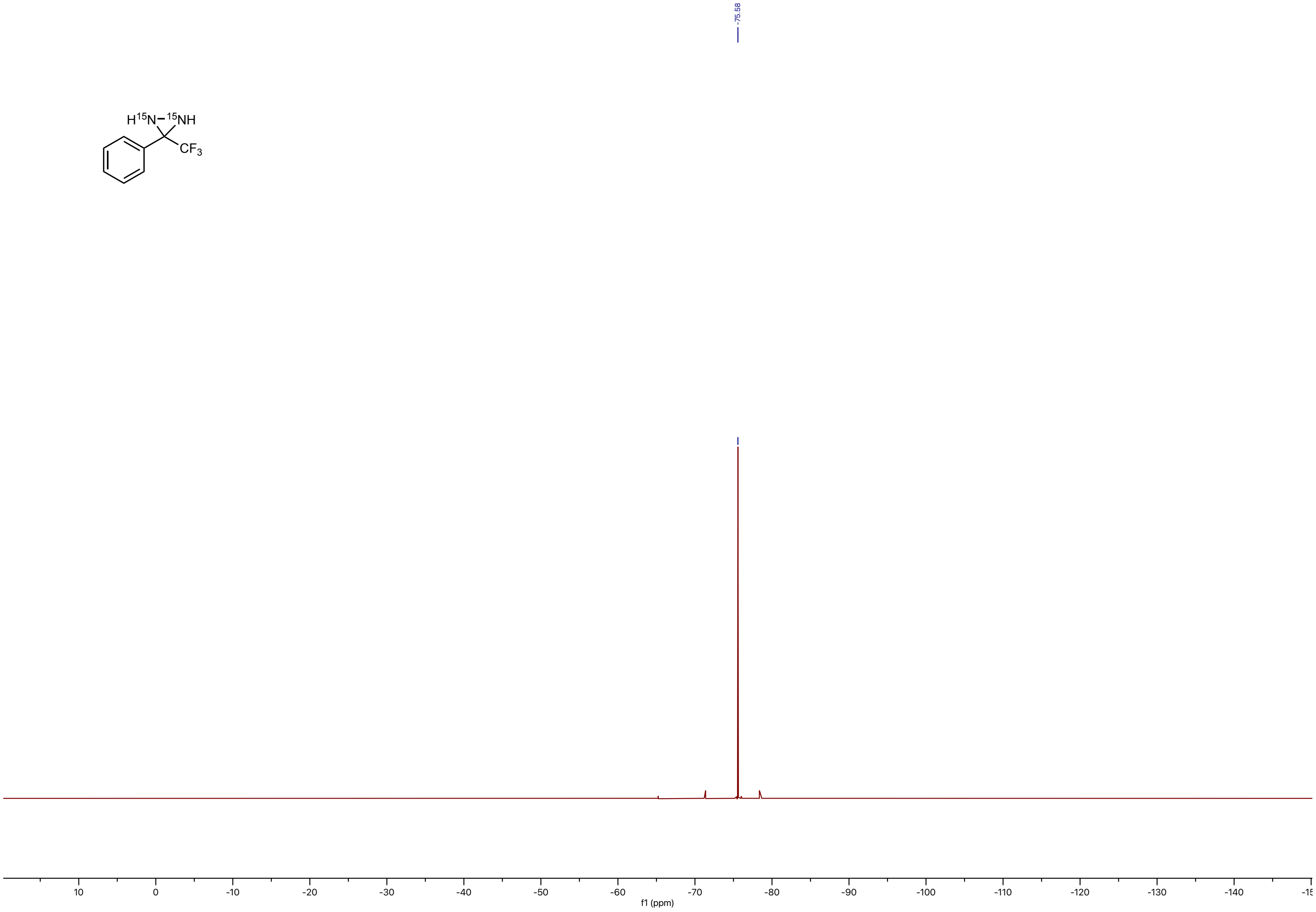

^{1}H NMR of 33 ($CDCl_3$, 500 MHz)

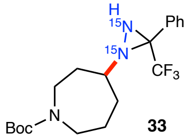

33

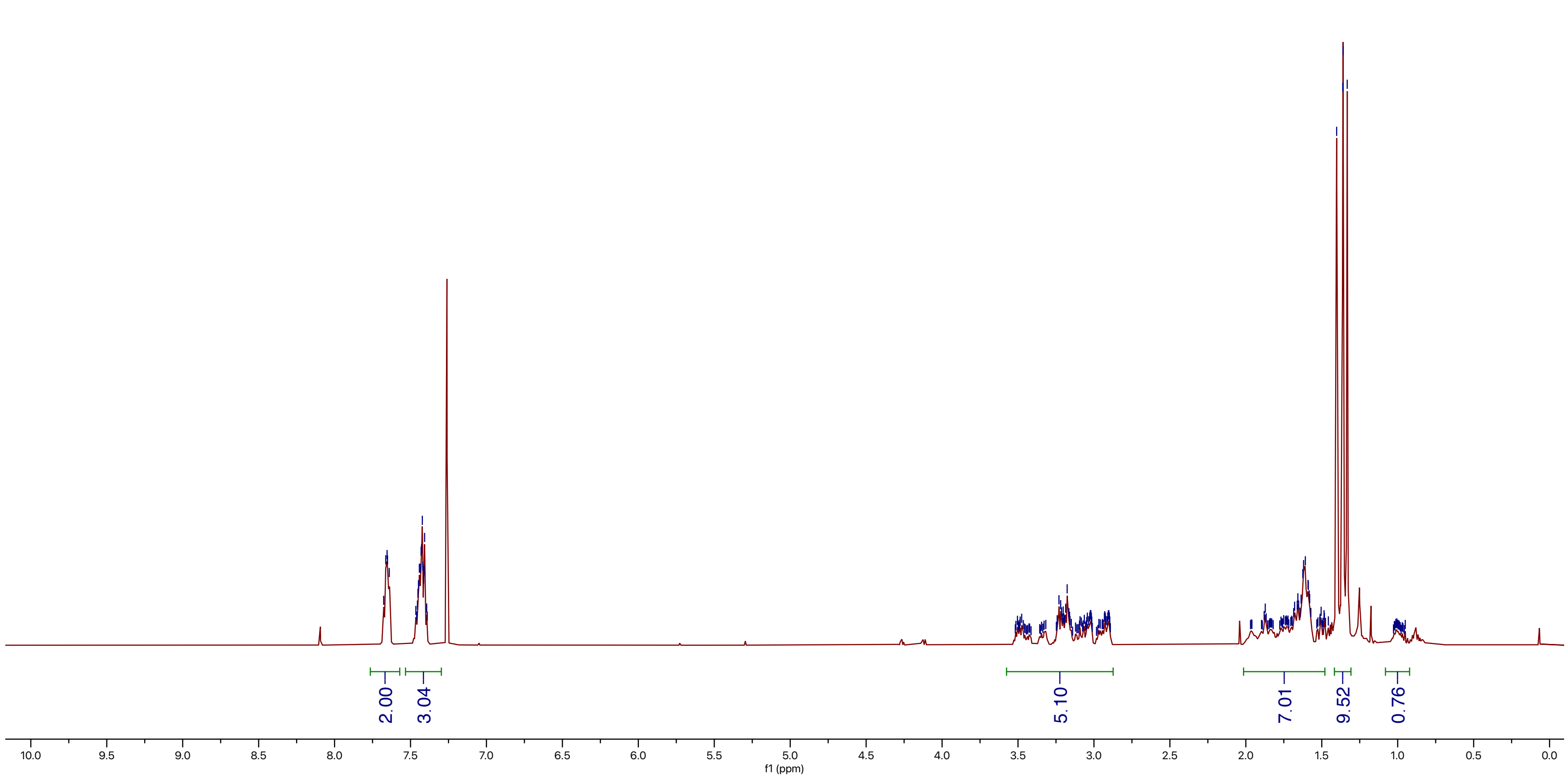

^{13}C NMR of 33 (CDCl$_3$, 126 MHz)

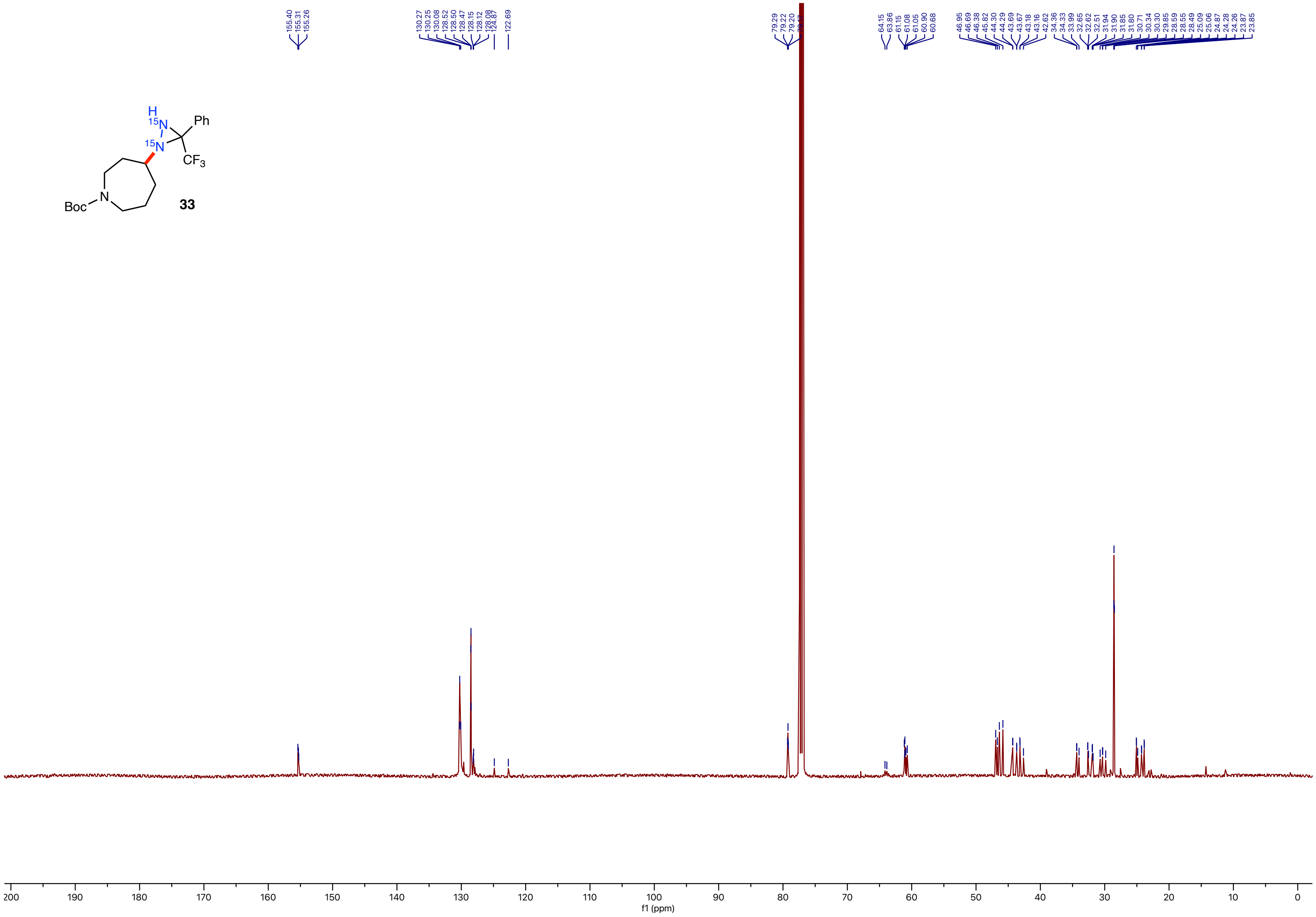

^{19}F NMR of 33 ($CDCl_3$, 471 MHz)

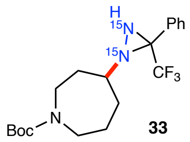

33

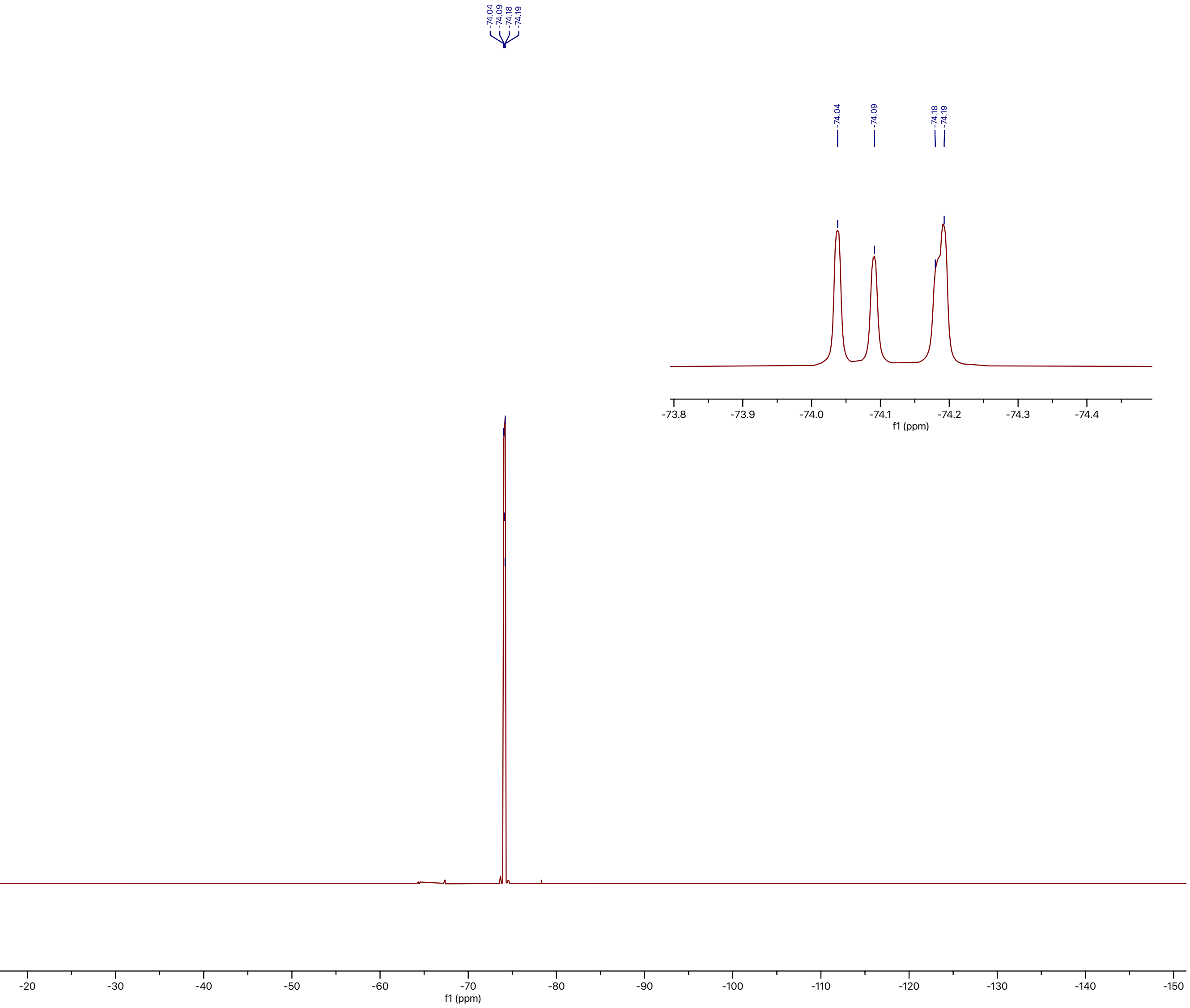

^{1}H NMR of ^{15}N-25 (DMSO, 500 MHz)

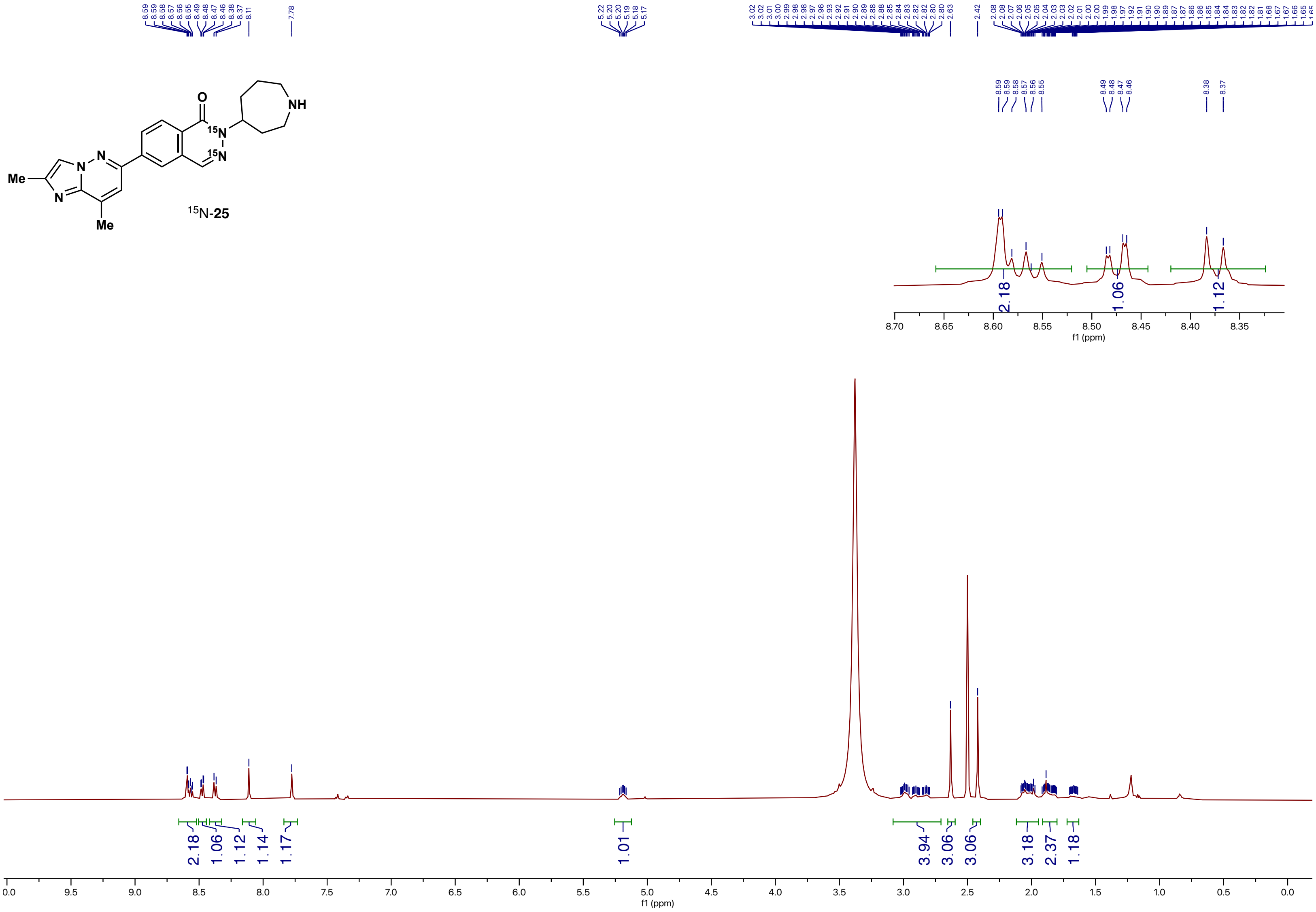

^{13}C NMR of ^{15}N-25 (DMSO, 126 MHz)

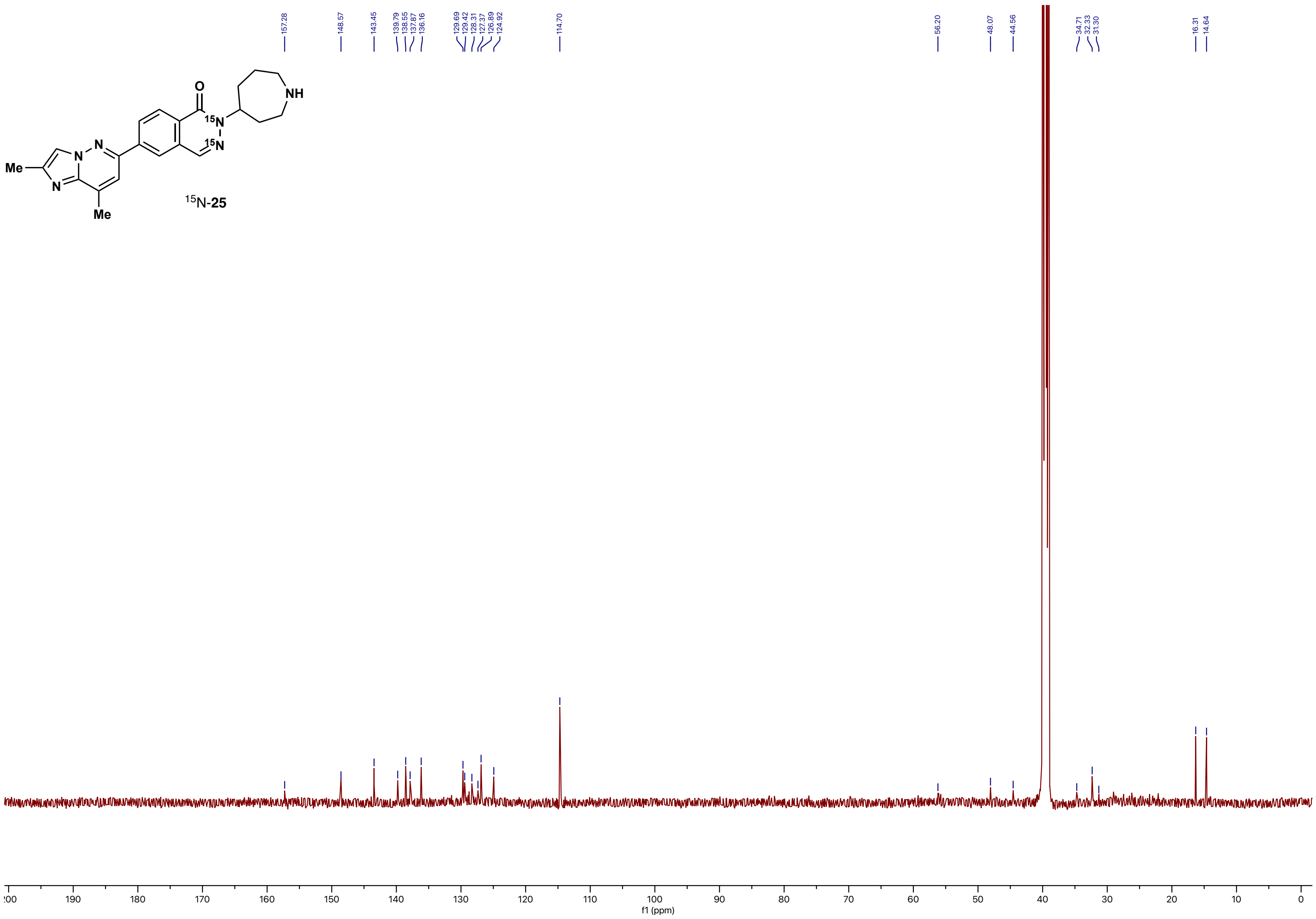

Supplementary Notes:

Troubleshooting:

Question 1: How do I decide which conditions should I use when doing the hydroamination?

Answer: The Mn-catalyzed conditions are best for symmetrical olefins since the reactions are unselective. The Co-catalyzed conditions with DCE/IPA as the solvent system (General Procedure A) work well with most examples. However, if the reaction does not reach 100% conversion or the yields are unsatisfactory, the "IPA only" solvent conditions (General Procedure B) can be used. While these reaction conditions are often a little slower and the reaction profiles not as clean, the conversions are often better.

Question 2: My starting materials are consumed based upon TLC, but the yield is lower than expected.

Answer: We found that the reaction goes through an "intermediate" complex that is difficult to directly monitor. For 1-(but-3-en-1-yloxy)-4-methoxybenzene, the starting material is consumed after ~8 h, but the isolated yield is 60% – 70%. After 16 h, the isolated yield is >90% and with 20 h, it will reach 99%. Letting the reaction run for enough time is crucial for optimal yields. However, excess reaction times (e.g. 48 h) are not necessary and can, for some substrates, result in messier reaction profiles.

Question 3: I ran the hydroamination and after purification my fractions looked clean via TLC, however, the ^{19}FNMR shows an additional peak at approx. –71 ppm. What should I do?

Answer: This peak can be attributed to 2,2,2-trifluoro-1-phenylethan-1-one, and you can remove it by running flash chromatography at a lower polarity.

Supplementary References:

1 Chandrachud, P. P., Wojtas, L. & Lopchuk, J. M. Decarboxylative Amination: Diazirines as Single and Double Electrophilic Nitrogen Transfer Reagents. *J. Am. Chem. Soc.* **142**, 21743-21750 (2020).

2 Bugge, S. *et al.* Structure–activity study leading to identification of a highly active thienopyrimidine based EGFR inhibitor. *Eur. J. Med. Chem.* **75**, 354-374 (2014).

3 Xia, W., Salmeia, K. A., Vagin, S. I. & Rieger, B. Concerning the Deactivation of Cobalt (III)-Based Porphyrin and Salen Catalysts in Epoxide/CO2 Copolymerization. *Chem. Eur. J.* **21**, 4384-4390 (2015).

4 Leduc, A. B. & Kerr, M. A. Total Synthesis of (−)-Allosecurinine. *Angew. Chem. Int. Ed.* **47**, 7945-7948 (2008).

5 Tzur, E. *et al.* Stability and activity of cis-dichloro ruthenium olefin metathesis precatalysts bearing chelating sulfur alkylidenes. *J. Organomet. Chem.* **769**, 24-28 (2014).

6 Green, S. A., Vásquez-Céspedes, S. & Shenvi, R. A. Iron–Nickel Dual-Catalysis: A New Engine for Olefin Functionalization and the Formation of Quaternary Centers. *J. Am. Chem. Soc.* **140**, 11317-11324 (2018).

7 Dixon, D. J., Ley, S. V. & Tate, E. W. The synthesis of mono- and bicyclic ethers via acid catalysed ring-opening cyclisation of tetrahydropyranyl ether derivatives. *J. Chem. Soc. Perkin Trans. 1*, 1829-1836 (2000).

8 Cleary, P. A. & Woerpel, K. A. Metal-Catalyzed Rearrangement of Homoallylic Ethers to Silylmethyl Allylic Silanes in the Presence of a Di-tert-butylsilylene Source. *Org. Lett.* **7**, 5531-5533 (2005).

9 Schevenels, F. T., Shen, M. & Snyder, S. A. Isolable and Readily Handled Halophosphonium Pre-reagents for Hydro- and Deuteriohalogenation. *J. Am. Chem. Soc.* **139**, 6329-6337 (2017).

10 Bernardi, A. *et al.* Computer-assisted design of chiral boron enolates: The role of ate complexes in determining aldol stereoselectivity. *Tetrahedron* **50**, 1227-1242 (1994).

11 Wang, X. *et al.* Optimization of Pan-Pim Kinase Activity and Oral Bioavailability Leading to Diaminopyrazole (GDC-0339) for the Treatment of Multiple Myeloma. *J. Med. Chem.* **62**, 2140-2153 (2019).

12 Nikitas, N. F., Voutyritsa, E., Gkizis, P. L. & Kokotos, C. G. Metal-free Photochemical Atom Transfer Radical Addition (ATRA) of BrCCl3 to Alkenes. *Eur. J. Org. Chem.* **2021**, 96-101 (2021).

13 Soulard, V., Villa, G., Vollmar, D. P. & Renaud, P. Radical Deuteration with D2O: Catalysis and Mechanistic Insights. *J. Am. Chem. Soc.* **140**, 155-158 (2018).

14 Andersen, C. *et al.* Introduction of Cyclopropyl and Cyclobutyl Ring on Alkyl Iodides through Cobalt-Catalyzed Cross-Coupling. *Org. Lett.* **21**, 2285 (2019).

15 Liu, X.-G. *et al.* Decarboxylative Negishi Coupling of Redox-Active Aliphatic Esters by Cobalt Catalysis. *Angew. Chem. Int. Ed.* **57**, 13096-13100 (2018).

16 Kuninobu, Y., Nishi, M. & Kanai, M. 5-Position-selective C–H trifluoromethylation of 8-aminoquinoline derivatives. *Org. Biomol. Chem.* **14**, 8092-8100 (2016).

17 Knez, D. *et al.* Stereoselective Activity of 1-Propargyl-4-styrylpiperidine-like Analogues That Can Discriminate between Monoamine Oxidase Isoforms A and B. *J. Med. Chem.* **63**, 1361-1387 (2020).

18 Xue, C.-B. *et al.* Synthesis and structure–activity relationship of a novel sulfone series of TNF-α converting enzyme inhibitors. *Bioorg. Med. Chem. Lett.* **14**, 4453-4459 (2004).

19 Davison, G. *et al.* Mapping Ligand Interactions of Bromodomains BRD4 and ATAD2 with FragLites and PepLites—Halogenated Probes of Druglike and Peptide-like Molecular Interactions. *J. Med. Chem.* **65**, 15416-15432 (2022).

20 Zhang, Y., Qian, J., Wang, M., Huang, Y. & Hu, P. Visible-Light-Induced Decarboxylative Fluorination of Aliphatic Carboxylic Acids Catalyzed by Iron. *Org. Lett.* **24**, 5972-5976 (2022).

21 Maza, R. J., Royes, J., Carbó, J. J. & Fernández, E. Consecutive borylcupration/C–C coupling of γ-alkenyl aldehydes towards diastereoselective 2-(borylmethyl)cycloalkanols. *Chem. Commun.* **56**, 5973-5976 (2020).

22 Kubota, K., Yamamoto, E. & Ito, H. Copper(I)-Catalyzed Enantioselective Nucleophilic Borylation of Aldehydes: An Efficient Route to Enantiomerically Enriched α-Alkoxyorganoboronate Esters. *J. Am. Chem. Soc.* **137**, 420-424 (2015).

23 Asano, Y., Nagasawa, Y., Yamaguchi, E. & Itoh, A. Aerobic Photooxidative Synthesis of β-Alkoxy Monohydroperoxides Using an Organo Photoredox Catalyst Controlled by a Base. *Chem. – Asian J.* **13**, 409-412 (2018).

24 Li, Z. *et al.* Divinylsulfonamides as Specific Linkers for Stapling Disulfide Bonds in Peptides. *Org. Lett.* **19**, 4972-4975 (2017).

25 Oleinik, I. I., Oleinik, I. V., Ivanchev, S. S. & Tolstikov, G. A. Design of postmetallocene catalytic systems of arylimine type for olefi n polymerization: XV. Synthesis of (N-Aryl)salicylaldimine ligands containing a but-3-enyloxy group and their complexes with titanium(IV) dichloride. *Russ. J. Org. Chem.* **49**, 1150-1156 (2013).

26 Mali, S. M., Bhaisare, R. D. & Gopi, H. N. Thioacids Mediated Selective and Mild N-Acylation of Amines. *J. Org. Chem.* **78**, 5550-5555 (2013).

27 Oh, H., Park, A., Jeong, K.-S., Han, S. B. & Lee, H. Copper-Catalyzed 1,2-Bistrifluoromethylation of Terminal Alkenes. *Adv. Synth. Catal.* **361**, 2136-2140 (2019).

28 Li, S. *et al.* One stone two birds: cobalt-catalyzed in situ generation of isocyanates and benzyl alcohols for the synthesis of N-aryl carbamates. *Org. Biomol. Chem.* **17**, 5891-5896 (2019).

29 Bunker, K. D., Sach, N. W., Huang, Q. & Richardson, P. F. Scalable Synthesis of 1-Bicyclo[1.1.1]pentylamine via a Hydrohydrazination Reaction. *Org. Lett.* **13**, 4746-4748 (2011).

30 Gaspar, B., Waser, J. & Carreira, E. M. Cobalt-Catalyzed Synthesis of Tertiary Azides from α,α-Disubstituted Olefins under Mild Conditions Using Commercially Available Reagents. *Synthesis* **2007**, 3839-3845 (2007).

31 Waser, J., Gaspar, B., Nambu, H. & Carreira, E. M. Hydrazines and Azides via the Metal-Catalyzed Hydrohydrazination and Hydroazidation of Olefins. *J. Am. Chem. Soc.* **128**, 11693-11712 (2006).

32 Waser, J. & Carreira, E. M. Convenient Synthesis of Alkylhydrazides by the Cobalt-Catalyzed Hydrohydrazination Reaction of Olefins and Azodicarboxylates. *J. Am. Chem. Soc.* **126**, 5676-5677 (2004).

33 Zhang, B. *et al.* Cobalt-Catalyzed Markovnikov-Selective Radical Hydroacylation of Unactivated Alkenes with Acylphosphonates. *J. Am. Chem. Soc.* **143**, 4955-4961 (2021).

34 Tokuyasu, T., Kunikawa, S., Masuyama, A. & Nojima, M. Co(III)−Alkyl Complex- and Co(III)−Alkylperoxo Complex-Catalyzed Triethylsilylperoxidation of Alkenes with Molecular Oxygen and Triethylsilane. *Org. Lett.* **4**, 3595-3598 (2002).

35 Guo, X. T., Le; Li, Yujiang; Wang, Qiang; Dong, Lihong. Quinocide and preparation method of hydrochloride thereof. CN105481766B (2016).

36 Mangan, D. *et al.* A new synthesis and preliminary evaluation of some analogues of mecamylamine – a compound with anti-addiction properties. *Org. Biomol. Chem.* **14**, 10787-10798 (2016).

37 Jirgensons, A., Kauss, V., Kalvinsh, I. & Gold, M. R. A Practical Synthesis of tert-Alkylamines via the Ritter Reaction with Chloroacetonitrile. *Synthesis* **2000**, 1709-1712 (2000).

38 Calvo, R. R., Cheung, W. S. & Player, M. R. 4-Piperidinecarboxamide modulators of vanilloid VR1 receptor. US2006116368A1 (2006).

39 Woll, M. *et al.* Compounds for Treating Hunting's Disease. WO2018226622A1 (2018).

40 Wang, B., Chai, X., Zhu, W., Wang, T. & Wu, Q. A general approach to spirolactonized Si-rhodamines. *Chem. Commun* **50**, 14374-14377 (2014).

41 Reynolds, D. *et al.* Compounds and methods for modulating splicing. WO2021207553A1 (2021).
